# Multifunctional Reagents for Quantitative Proteome-Wide Analysis of Protein Modification in Human Cells and Dynamic Profiling of Protein Lipidation During Vertebrate Development**

**DOI:** 10.1002/anie.201500342

**Published:** 2015-03-25

**Authors:** Malgorzata Broncel, Remigiusz A Serwa, Paulina Ciepla, Eberhard Krause, Margaret J Dallman, Anthony I Magee, Edward W Tate

**Affiliations:** Department of Chemistry, Imperial College London,Exhibition Road, London SW7 2AZ (UK); Leibniz-Institut für Molekulare Pharmakologie (FMP)Robert-Roessle-Str. 10, 13125 Berlin (Germany); Department of Life Sciences, Imperial College London, Exhibition RoadLondon SW7 2AZ (UK); National Heart and Lung Institute, Imperial College LondonExhibition Road, London SW7 2AZ (UK)

**Keywords:** capture reagents, lipidation, mass spectrometry, post-translational modification, proteomics

## Abstract

Novel multifunctional reagents were applied in combination with a lipid probe for affinity enrichment of myristoylated proteins and direct detection of lipid-modified tryptic peptides by mass spectrometry. This method enables high-confidence identification of the myristoylated proteome on an unprecedented scale in cell culture, and allowed the first quantitative analysis of dynamic changes in protein lipidation during vertebrate embryonic development.

Although advancements in protein mass spectrometry (MS) have enabled global profiling of numerous post-translational modifications (PTMs), confident high-throughput identification of lipidated proteins remains problematic. Lipidation adversely affects solubility, chromatographic properties, and ionization of modified peptides, and routine proteome-wide detection by MS and assignment of the modification sites remains an unsolved challenge.[1] Furthermore, lipidated proteins are often present at relatively low abundance in cells, and lipid-specific enrichment is required to reduce sample complexity and improve discovery. Enrichment strategies can exploit natural features of the lipid, for example, the use of anti-farnesylpeptide antibodies;[2] indirect analysis through acyl–biotin exchange for *S*-acylation;[3] or more generalizable introduction of a functional handle through metabolic incorporation of a bioorthogonal chemical tag.[4]

Co-translational myristoylation is a specific class of protein lipidation whereby *N*-myristoyl transferases (NMTs) catalyze the covalent and irreversible addition of a tetradecanoyl chain at an N-terminal glycine (Gly), revealed by the action of methionine aminopeptidases during protein synthesis at ribosomes.[5] Metabolic tagging has become the technique of choice for whole-proteome profiling of the cellular myristoylated proteome. As previously demonstrated,[6] metabolic tagging of a cellular proteome with tetradec-13-ynoic acid (YnMyr) and subsequent elaboration through ligation with secondary reporters (capture reagents) enables visualization, enrichment, and MS-based identification of myristoylated proteins. This chemoproteomic methodology delivers greatly improved processing time and sensitivity over traditional radioisotope or direct MS approaches, but there are also limitations. Firstly, as with any affinity enrichment technique, unspecific protein background is often observed when coupled to high-sensitivity MS detection. Secondly, protein fatty acyl transferases that utilize longer acyl-CoAs as substrates may also use YnMyr either natively or following chain extension, which may lead to false positive identifications.[7] Finally, robust and general methods for confirmation of the lipidation site have yet to be established.

To overcome these limitations, we report a portfolio of reagents for the enrichment and visualization of myristoylated proteomes and direct MS detection of lipid modification of protein N termini. The design of these reagents is based on compound **1** (Figure 1 a), a robust tool for enrichment and in-gel fluorescence (igFl) analysis of metabolically tagged proteomes.[8] To make **1** amenable to the detection of lipid-modified peptides, we envisioned the introduction of a linker (Figure S1 in the Supporting Information) to facilitate cleavage as an integral part of a proteomic workflow. In a standard tagging experiment (Figure 1 b), cells or organisms are treated with YnMyr or a myristic acid (Myr) control to metabolically tag proteins via activation by cellular acyl-CoA synthase followed by transfer by NMTs. The cells/organisms are harvested and the metabolically tagged proteins ligated to a capture reagent through copper-catalyzed alkyne azide cycloaddition (CuAAC) and affinity-enriched. The enriched proteomes are visualized by igFl to assess metabolic tagging and enrichment efficiency, or analyzed through on-bead proteolytic digestion, MS analysis, and software-aided protein identification.

**Figure 1:**
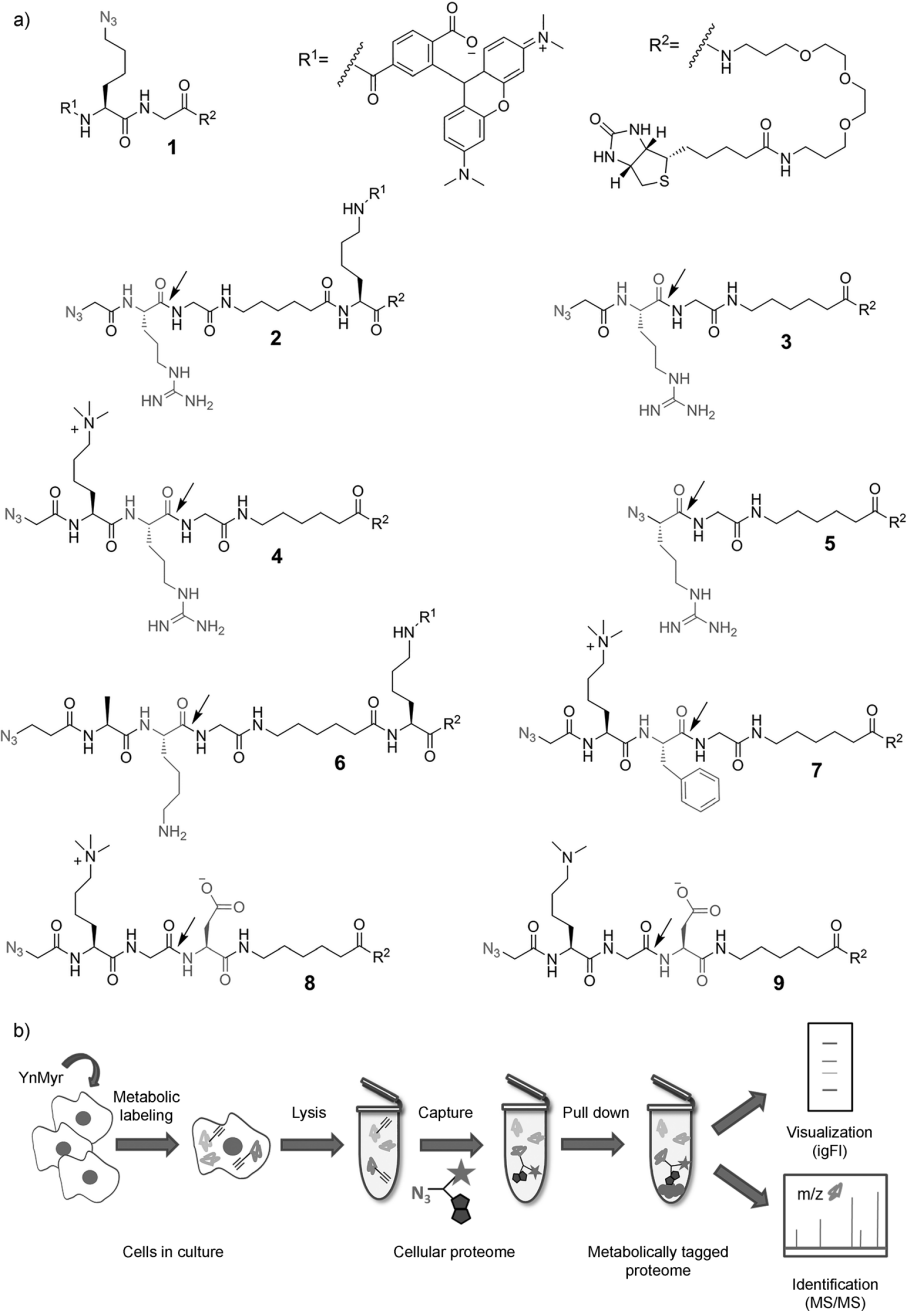
a) Structures of capture reagents 1–9. Protease (trypsin, chymotrypsin, AspN) cleavage sites are indicated by arrows. b) General proteomic workflow; the capture reagent is shown schematically as a cartoon: star=TAMRA (R^1^), fused pentagons=biotin (R^2^).

We synthesized a library of reagents bearing enzymatically cleavable linkers (Figure 1 a, Table S1 in the Supporting Information). Reagents **2**–**5** contain arginine (Arg) residues and reagent **6** contains a lysine (Lys) residue to enable proteolysis by trypsin and simultaneous enhancement of the ionization properties of lipidated peptides. Reagent **7** in turn incorporates phenylalanine (Phe) and reagents **8**–**9** incorporate aspartic acid (Asp) to facilitate cleavage by chymotrypsin and AspN, respectively. These proteases are less commonly used in proteomic workflows, however they may offer complementary protein sequence coverage, thus improving overall modified peptide discovery. Similarly to **1**, compounds **2** and **6** were equipped with the TAMRA fluorophore (R^1^) for fast visualization by igFl. All other reagents lack the fluorophore to reduce bulk and possible steric hindrance, since biotin (contained in R^2^) is sufficient for visualization (e.g., by using streptavidin conjugated to horseradish peroxidase (streptavidin–HRP)). In addition, **5** was equipped with α-azido-arginine to further minimize the size of the cleaved product. Finally, we aimed to enhance the ionization properties of the lipidated peptides still further by incorporating trimethyl (**4**, **7**, **8**) or dimethyl (**9**) lysine residues.

Taking advantage of both the TAMRA and biotin moieties, we first investigated the efficiency of **2**–**9** for the capture and enrichment of myristoylated proteins. HEK293 cells were treated with YnMyr or Myr for 24 h followed by lysis, CuAAC with **1**–**9**, and affinity enrichment on streptavidin-conjugated beads. Captured proteins were eluted by thermal denaturation, resolved by SDS-PAGE, and visualized by igFl or Western Blotting with streptavidin–HRP (Figure S2a and b). We were pleased to observe that the majority of the reagents showed comparable enrichment efficiency to **1** and that there was negligible background signal, as shown by the Myr control. However, the performance of **8** and **9** was clearly diminished, which may indicate lower efficiency of CuAAC, and these reagents were excluded from further analysis. We also observed that despite their bulkier structures, **2** and **6** performed as robustly as **1**, which was demonstrated by two alternative readouts.

We then evaluated the capacity of these reagents to identify proteins with a consensus myristoylation sequence (MG-proteins) and, most importantly, N-terminally myristoylated peptides. Samples were prepared as described above, subjected to CuAAC with **2**–**7**, affinity-enriched, and digested with either trypsin (**2**–**6**) or chymotrypsin (**7**), followed by MS analysis.

Five reagents (**2**–**4** and **6**–**7**) identified 38 lipid-modified peptides with high (>;99 %) confidence (Figure S2c and Table S2 in the Supporting Information). Nearly 90 % of these peptides were discovered with **2**, thus demonstrating its high effectiveness despite the presence of the TAMRA dye and lower predicted net charge compared to **3** and **4**, respectively. Reagent **2** was also superior to **6**, thus suggesting that the proteolysis step and/or the ionization properties of the Arg-containing conjugates were improved compared to Lys since CuAAC and enrichment were similar (Figure S2). These steps also seemed efficient for **5**, however, no lipidated peptides were detected; we speculate that proximity of the triazole to the cleavage site reduces the affinity for trypsin. Regardless of the reagent type, approximately 30 % of all of the proteins identified carried an N-terminal MG motif (Table S3), which is more than four-fold higher than in analysis of the native proteome, where the abundance of MG proteins is approximately 7 %.[9] The remaining 70 % of the detected proteins represent tagging at sites other than the N terminus as a result of the promiscuity of lipid transferases and proteins binding to beads and/or tagged proteins, thus highlighting the utility of methodologies that allow either quantification of NMT inhibition (if reliable inhibitors are available)[6b] or direct detection of lipidated peptides to improve modified protein ID confidence.

Having selected the best performing capture reagent (**2**), we next aimed to maximize the discovery of myristoylated peptides and the corresponding endogenous protein substrates of human NMT. Larger-scale chemical proteomics experiments were undertaken with three commonly used cell lines: HeLa, MCF7, and HEK293. Two complementary software packages were employed for MS data analysis, with primary identification of lipid-modified peptides performed with PEAKS7, and MaxQuant1.5 used as a secondary platform.[10] The latter was also used to quantify protein levels between YnMyr- and Myr-treated samples, with Myr serving as a control for both nonspecific protein identification and reliability of the modified peptide identifications. 81 lipid-modified peptides were detected in PEAKS7 analysis, of which approximately 40 % were common to all three cell lines (Figure 2 a). MaxQuant analysis delivered 62 lipidated peptides, four of which were not observed in PEAKS7.[11] Importantly, each search engine returned only one lipidated peptide common to both YnMyr and Myr control samples, which was assigned as a false positive and excluded from the list of myristoylated peptides (Table S4).

**Figure 2:**
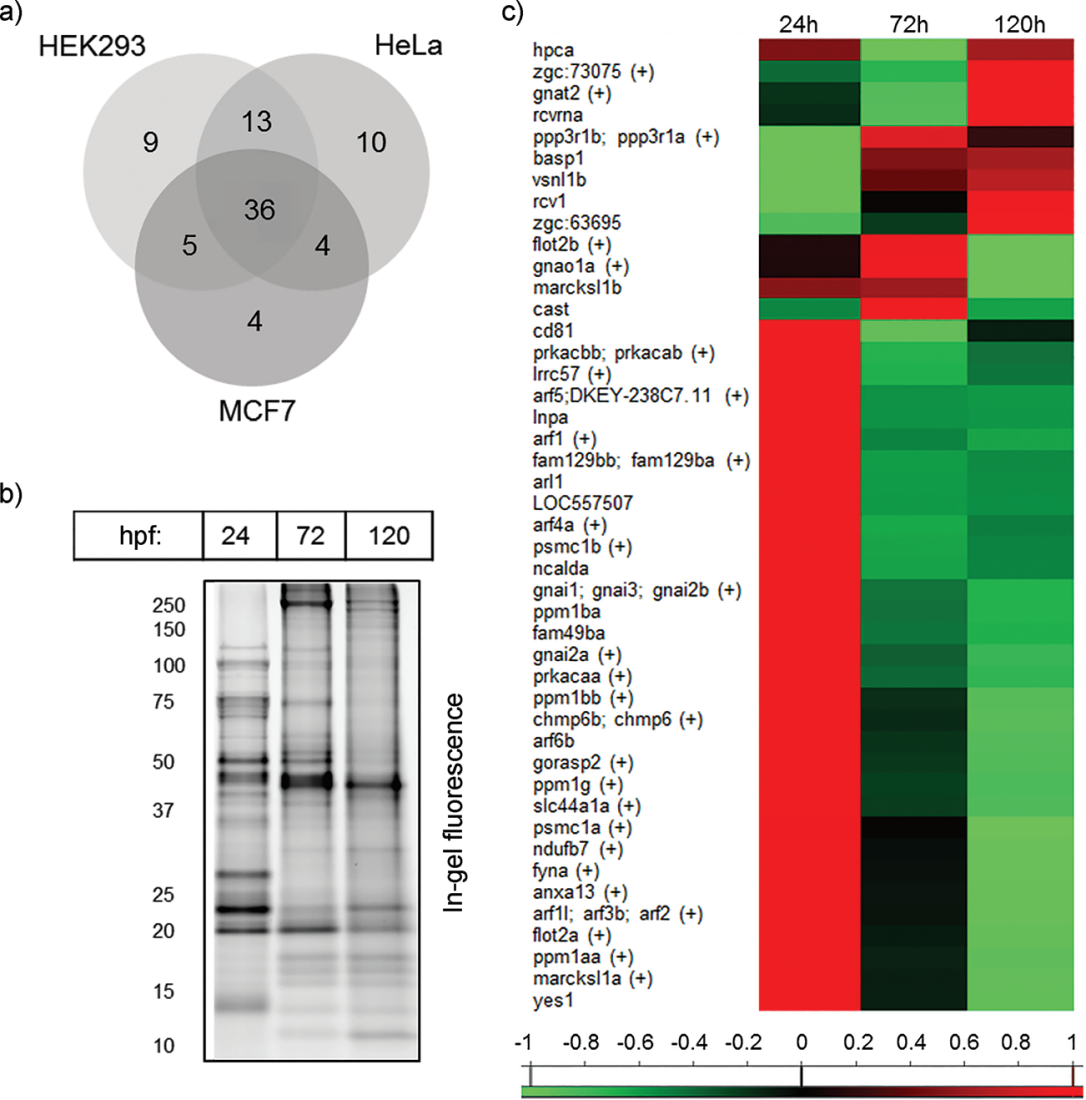
a) Modified peptide discovery in human cells. b) Time-dependent metabolic tagging with YnMyr visualized by igFl. c) Quantitative MS-based analysis of myristoylation in developing zebrafish embryos. Color coding represents normalized levels (log_2_ scale) of myristoylation. Protein targets are reported by gene names with peptide indicated as (+).

Based on statistically significant enrichment of MG proteins pulled down from YnMyr-treated samples compared to Myr controls, we identified 170, 163, and 145 candidate myristoylated proteins for HEK293, HeLa, and MCF7 cells, respectively (Table S5). However, a previous comprehensive study of quantitative dose response to NMT inhibition showed that enrichment is only a simple and indirect measure of N-terminal myristoylation since only 70 out of 169 enriched MG HeLa proteins robustly responded to NMT inhibition.[6b] In agreement with this alternative approach, our dataset shows direct evidence for 87 of the candidate proteins in the three cell lines. Herein, we provide direct proof for myristoylation in Hela cells for 47 previously reported proteins, as well as significantly increased coverage of since direct MS/MS evidence for myristoylated peptides was detected for 69 enriched MG targets in HeLa, 65 in HEK293, and 50 in MCF7 cells (Table S5). This represents the largest directly validated database reported to date for the *N*-myristoylated human proteome.

We next applied **2** to profile the myristoylated proteome for the first time in a developing organism. Following evaluation of YnMyr tagging specificity (Figure S3), *Danio rerio* (zebrafish) embryos were metabolically tagged in specific time windows (0–24, 48–72, and 96–120 hours post-fertilization (hpf)) and processed according to the standard workflow. Chemical proteomics experiments revealed the scope of myristoylation in developing zebrafish; 72 potential myristoylation targets were identified based on statistical analyses of YnMyr/Myr enrichment and 56 lipid-modified peptides were discovered (Tables S6 and S7). Notably, in the absence of validated NMT inhibitors for zebrafish, the methodology presented herein is currently the only approach for confident assignment of myristoylated proteins in this animal model.

We also investigated the dynamic nature of myristoylation through pulsed YnMyr tagging as described above, coupled with visualization by igFl and quantification through triplex dimethyl labeling[12] (Figure 2 b and c, respectively). IgFl analysis indicates development-stage-specific *D. rerio* protein myristoylation profiles that reflect differential protein expression during embryonic development. Further MS-based analysis allowed the quantification of myristoylation dynamics for 54 zebrafish proteins (Figure 2 c), which revealed that myristoylation is most prominent in early development. Several proteins myristoylated within 24 hpf (e.g., prkaca and gnai families) participate in progesterone-mediated maturation, the hedgehog and wnt signaling pathways, melanogenesis, and meiosis, all of which are critical in early development (Table S8).

In summary, the multifunctional capture reagents reported herein enable robust identification of metabolically-tagged myristoylated proteomes, with unprecedented confidence resulting from the combination of chemical-probe-based enrichment and release and direct detection of lipid-modified peptides by MS. Previously reported reagents[13] typically required an extra proteolytic step and their capacity to enable the detection of lipidated peptides has not been demonstrated. Herein, we report the largest database (87 counts) of experimentally validated human proteins that are myristoylated at an endogenous level in living cells. We also present the first profile of myristoylation in a living multicellular organism and the confident identification of over 50 novel targets. Importantly, this work provides the first example of the analysis of any protein lipidation event during vertebrate development. We envision that our reagents will find application in the quantitative and dynamic analysis of other PTMs, and in related workflows such as activity-based protein profiling, both in cells and in multicellular organisms.
