# Supplementary material for: Multifunctional Reagents for Quantitative Proteome-Wide Analysis of Protein Modification in Human Cells and Dynamic Profiling of Protein Lipidation During Vertebrate Development
Source: Angew Chem Int Ed Engl. 2015 Mar 25;54(20):5948–51. doi: 10.1002/anie.201500342 (PMC4471546; doi:10.1002/anie.201500342)
Supplement: Supplementary file 1 [file anie0054-5948-sd1.pdf]

Angewandte Chemie
Eine Zeitschrift der Gesellschaft Deutscher Chemiker

Supporting Information

German Edition: DOI:

Multifunctional Reagents for Quantitative Proteome-Wide Analysis of Protein Modification in Human Cells and Dynamic Profiling of Protein Lipidation During Vertebrate Development**

Malgorzata Broncel, Remigiusz A. Serwa, Paulina Ciepla, Eberhard Krause, Margaret J. Dallman, Anthony I. Magee, and Edward W. Tate**

anie_201500342_sm_miscellaneous_information.pdf

Contents:

1. Supplementary methods

2. Synthetic procedures

3. Supplementary Figures and Tables

4. References

1. Supplementary methods

General:

Culture media and reagents for cell culture were obtained from Sigma Aldrich and Gibco (Life Technologies). All CuAAC reagents ($CuSO_4$, TCEP, TBTA), buffer salts, DTT, iodoacetamide were from Sigma Aldrich. EDTA-free complete protease inhibitor and pronase were obtained from Roche Diagnostics. Absorbance in 96-well plates was measured using a SpectraMax M2/M2e Microplate Reader from Molecular Devices. For proteomics, all buffers were filtered using a 0.2 µm filter. Low binding tubes (Protein LoBind tubes, Eppendorf) were used to carry out the enrichment of proteins for MS-based proteomics. Trypsin and chymotrypsin were sequencing grade from Promega.

1.1 Cell and zebrafish culture

1.1.1 Cells

Cells used herein (Cancer Research UK cell services core facility) were grown in DMEM supplemented with 10 % FBS in a humidified 10 % CO_2-containing atmosphere at 37 °C. Cells were plated 24 h before treatments with YnMyr or Myr control.

1.1.2 Zebrafish

Fish were maintained according to standard practices and all procedures conformed to U.K. Home Office regulations (ASPA 1986) under Animal Project Licence no. PPL 70/7472. Adult zebrafish strains AB were kept at 28 ºC on 14 hours light and 10 hours dark cycle. Embryos were obtained from natural spawnings and were maintained in system water (combination of tap and RO (reverse osmosis) water).

1.2 YnMyr tagging and lysis

1.2.1 Cells

Upon treatment the medium was removed and replaced by fresh culture media supplemented with 20 µM YnMyr or Myr. The cells were then incubated for 24 h, washed with PBS (2x) and lysed on ice using the following lysis buffer: PBS 1x, 0.1 % SDS, 1 % Triton X-100, 1 x EDTA-free complete protease inhibitor. Lysates were kept on ice for 20 min and centrifuged at 17,000 × g for 20 min to remove insoluble material. Supernatants were collected and stored at -80 ºC. Protein concentration was determined using the Bio-Rad DC Protein Assay.

1.2.2 Zebrafish

Strain AB zebrafish embryos were placed in zebrafish water (system water containing 0.0001% methylene blue, 25 mL). The embryos were treated as described in Laughlin et.al.[1] with some adjustments. At approximately 4-5 h post-fertilisation (hpf) the embryos were enzymatically dechorionated by incubation in a 1 mg/mL solution of pronase in zebrafish water at room temperature for approximately 7 min. The solution with embryos was poured into a beaker with zebrafish water (200 mL) and swirled gently to wash embryos. The solution was removed (not completely) and more zebrafish water was added. The cleaning process was repeated (4x) and during this most of the chorions were also removed. Then,

using a Pasteur pipette, the embryos were transferred into 1% agarose-coated wells of a 48-well plate containing 250 µL of feeding solution (zebrafish water containing 20 µM YnMyr or 20 µM Myr as negative control in DMSO). The embryos were kept in the solution for 24 h. For longer time points (72 or 120 hpf) the embryos were placed in zebrafish water for 48 hpf or 96 hpf, respectively. After this time the already hatched embryos were placed in a 48-well plate containing 250 µL of feeding solution (as above) and incubated for additional 24 h. Following metabolic tagging embryos were euthanized (MS-222 solution (250 mg/L)), transferred to 1.5 ml Eppendorf tubes and deyolked using a protocol described previously by Link et al. [2] in a calcium-containing solution. The deyolked embryos were treated as described previously by Hinz et.al. [3] with adjustments. The embryos were washed with 200 µL ice-cold lysis solution (PBS containing 2 x EDTA-free complete protease inhibitor). The solution was removed and replaced by fresh lysis solution (100 µL). Zebrafish embryos were homogenised with a Kimble® Kontes Disposable pellet pestle with cordless motor. SDS was added to the lysate to a final concentration of 1 % and Benzonase® Nuclease (0.5 µL, Sigma Aldrich) was also added. The lysate was vortexed at room temperature (5 min) and heated at 95 ºC (10 min). Lysate was allowed to cool down on ice for 5 min, before addition of more lysis solution (150 µL) and Triton X-100 to a final concentration of 0.2 %. The lysate was centrifuged (16,000 xg, 10 min, 4 ºC) to remove insoluble material. The supernatant was collected and used for further experiments or stored at -80 °C.

72 h YnMyr pulse experiment: Prepared as described above but the embryos were kept in the feeding solution for 72 hpf. Due to a certain degree of deformation of embryos compared to non-treated controls, 72 h pulse was substituted with three shorter (24 h) treatments with YnMyr or Myr (see above), under which conditions the development of embryos appeared unaffected. The samples generated from the 72 h pulse experiment were processed and searched for lipidated peptides; however, protein quantification was not performed.

<u>1.3 CuAAC and enrichment</u>

Lysates were thawed on ice. 100 µg of proteins were taken and diluted to 1 mg / mL using the lysis buffer. A click mixture was prepared by adding reagents in the following order and by vortexing between the addition of each reagent: a capture reagent (1 µL, stock solution 10 mM in water, final concentration 0.1 mM), $CuSO_4$ (2 µL, stock solution 50 mM in water, final concentration 1 mM), TCEP (2 µL, stock solution 50 mM in water, final concentration 1 mM), TBTA (1 µL, stock solution 10 mM in DMSO, final concentration 0.1 mM). Following the addition of the click mixture (6 µL/sample) the samples were vortexed (RT, 1 h), and the reaction was stopped by addition of EDTA (final concentration 10 mM). Subsequently, proteins were precipitated (chloroform/methanol, 0.25:1, relative to the sample volume), the precipitates isolated by centrifugation (17,000 x g, 10 min), washed once with methanol (400 µL) and air dried (10 min). The pellets were then resuspended (final concentration 1 mg/mL, PBS, 0.4 % SDS) and the precipitation step was repeated to remove excess of the capture reagent. Next, samples were added to 15 µL of pre-washed (0.2 % SDS in PBS (3 × 500 µL)) Dynabeads® MyOne™ Streptavidin C1 (Invitrogen) and gently vortexed for 90 min. The supernatant was removed and the beads were washed with 0.2 % SDS in PBS (3 x 500 µL).

1.4 SDS-PAGE, in-gel fluorescence and Western Blotting (WB)

30 µL of 2 % SDS in PBS and 10 µL 4x SLB (Invitrogen) were added to the beads and 7 µL SLB were added to 20 µL of supernatant. The samples were then boiled (10 min), centrifuged (1,000 x g, 2 min) and loaded on a 12 % SDS-PAGE gel (supernatant: 13 µL (~10 µg of proteins); pull-down: 15 µL (~40 µg of proteins). Following electrophoresis (60 min, 130V), the gel was washed with MilliQ (3x), soaked in fixing solution (40 % MeOH, 10 % acetic acid, 50 % water) for 5 min and washed with water (3x). In-gel fluorescence was detected using an Ettan DIGE Imager (GE Healthcare) and the protein loading was checked by Coomassie staining. For WB, proteins were not fixed, instead they were transferred onto PVDF membranes using an iBlot device (Invitrogen) according to manufacturer's instructions. After brief washing with TBS-T (1 x TBS, 0.1 % Tween-20) membranes were blocked (3 % BSA in TBS-T, 1h), washed with TBS-T (3x) and incubated with Streptavidin-HRP in 0.3 % BSA, TBS-T (1h). Following washing (TBS-T, 3x), membranes were treated with Luminata Crescendo Western HRP substrate (Millipore) and chemiluminescence was recorded using a LAS-3000 Imaging System (GE Healthcare).

1.5 CuAAC and sample preparation for MS-based proteomics

1.5.1 Cells

CuAAC reaction was carried out with 0.4 mg (small scale) or 2 mg (large scale) of proteins at 2 mg/mL. Proteins were captured by adding a mixture of respective capture reagent (final concentration 0.1 mM), $CuSO_4$ (final concentration 1 mM), TCEP (final concentration 1 mM) and TBTA (final concentration 0.1 mM). The samples were vortex-mixed at RT for 1 h before the addition of EDTA (final concentration 10 mM), methanol (4 volumes), chloroform (1 volume), and water (3 volumes). The samples were vortex-mixed briefly, centrifuged (10,000 × g, 20 min) and the resulting pellets were resuspended (at 2 mg/mL, 1% SDS in PBS) after which the precipitation step was repeated. Following centrifugation (as above) the pellets were washed with methanol (4 volumes) and dried. Subsequently, the pellets were resuspended in 2 % SDS in PBS and, once completely dissolved, PBS was added to dilute the sample (final concentration 1 mg/mL, 0.4 % SDS). NeutrAvidin agarose resin (Thermo Scientific) was washed with 0.2 % SDS in PBS (3x). Typically 50 µL of bead slurry was used for 1 mg of lysate. The samples were mixed with the beads and the enrichment was carried out on a rotating wheel for 2 h at RT. Following the removal of supernatants the beads were sequentially washed with 1 % SDS in PBS (3x), 4 M urea in 50 mM AMBIC (2x) and 50 mM AMBIC (5x). The samples were reduced (5 mM DTT, 55 °C, 30 min) and cysteines alkylated (10 mM iodoacetamide, RT, 30 min in the dark) with washing the beads after each step (2x, 50 mM AMBIC). Protein digestion was initiated upon addition of trypsin (ca. 1/1000 w/w protease to protein ratio) or chymotrypsin (according to supplier's protocols) and samples were incubated overnight at 37 °C or 25 °C, respectively. The samples were then briefly centrifuged, diluted twice (0.1% TFA, 50 mM AMBIC) and stage-tipped according to a published protocol.[4] Elution from the sorbent (SDC-XC, from 3M) with 70% acetonitrile in water was followed by speed-vac-assisted solvent removal, reconstitution of peptides in 0.5% TFA, 2% acetonitrile in water, and finally sample transfer into LC-MS sample vials. All experiments involved biochemical triplicates.

1.5.2 Zebrafish

Samples were processed as described above for cell-based experiments with the following adjustments. In label free experiments CuAAC reaction was carried out with 1.5 mg (mix of 3 x 0.5 mg proteins from 24, 72 and 120 h tagging) of proteins as described above. For dimethyl labelling experiments CuAAC reaction was carried out with 0.2 mg of proteins (from each 24, 72 and 120 h tagging) as described above. Triplex dimethyl labelling was performed according to a published protocol, [5] utilising the following sets of reagents:

Light Labelling Buffer: A mix of 90% (v/v) PB 7.5, 5% (v/v) of a 4% (v/v) CH_2O solution in water and 5% of a 0.6 M $NaBH_3CN$ solution in water;

Medium Labelling Buffer: A mix of 90% (v/v) PB 7.5, 5% (v/v) of a 4% (v/v) CD_2O solution in water and 5% of a 0.6 M $NaBH_3CN$ solution in water;

Heavy Labelling Buffer: A mix of 90% (v/v) PB 7.5, 5% (v/v) of a 4% (v/v) $^{13}CD_2O$ solution in water and 5% of a 0.6 M $NaBD_3CN$ solution in water.

After isotopic labelling the samples (24, 72 and 120 h) were mixed in 1:1:1 ratio.

All experiments involved biological triplicates.

1.6 LC-MS/MS analysis

The analysis was performed using an Acclaim PepMap RSLC column 50 cm × 75 µm inner diameter (Thermo Fisher Scientific) using a 2 h acetonitrile gradient in 0.1 % aqueous formic acid at a flow rate of 250 nL/min. Easy nLC-1000 was coupled to a Q Exactive mass spectrometer via an easy-spray source (all Thermo Fisher Scientific). The Q Exactive was operated in data-dependent mode with survey scans acquired at a resolution of 75,000 at m/z 200 (transient time 256 ms). Up to 10 of the most abundant isotope patterns with charge +2 or higher from the survey scan were selected with an isolation window of 3.0 m/z and fragmented by HCD with normalized collision energies of 25. The maximum ion injection times for the survey scan and the MS/MS scans (acquired with a resolution of 17 500 at m/z 200) were 20 and 120 ms, respectively. The ion target value for MS was set to 10^6 and for MS/MS to 10^5, and the intensity threshold was set to 8.3×10^2.

1.7 Proteomics data analysis with MaxQuant

The data were processed with MaxQuant[6] (version 1.5.0.25) and the peptides were identified from the MS/MS spectra searched against human complete proteome (uniprot, September 2014) using Andromeda[7] search engine. Cysteine carbamidomethylation was selected as a fixed modification and methionine oxidation as a variable modification. For *in silico* digests of the reference proteome the following peptide bond cleavages were allowed: arginine or lysine followed by any amino acid (a general setting referred to as Trypsin/P); phenylalanine, tyrosine or tryptophan followed by any amino acid (this setting was applied only for chymotrypsin digested samples). Up to two missed cleavages were allowed. The false discovery rate was set to 0.01 for peptides, proteins and sites. Other parameters were used as pre-set in the software. “Unique and razor peptides” mode was selected to allow identification and quantification of proteins in groups (razor peptides are uniquely assigned to protein groups and not to individual proteins). Data were analyzed using Microsoft Office Excel 2007 and Perseus (version 1.5.0.9).

1.7.1 Label-free quantification (LFQ) data analysis

LFQ experiments in MaxQuant were performed using a built-in label-free quantification algorithm[6] enabling the 'Match between runs' option (time window 0.7 minutes). The experiment comprised three replicates treated with YnMyr (and three replicates treated with Myr, where applicable). The LFQ is based on intensities of proteins calculated by MaxQuant from peak intensities and based on the ion currents carried by peptides whose sequences match a specific protein or a protein group to provide an approximation of abundance. The outcome from MaxQuant was uploaded into Perseus.

1.7.1.1 Capture reagents comparison LFQ

Label free intensities were logarithmized (base 2) and replicates were filtered to require 1 (2 or 3) valid value across the three replicates. The data were filtered through a modified fasta file comprising only N-terminal MG proteins.

1.7.1.2 Cell line and zebrafish LFQ

The replicates were grouped together and YnMyr protein group was filtered to require one valid value across the three replicates. Label free intensities were logarithmized (base 2) and empty values were imputed with random numbers from a normal distribution, whose mean and standard deviation were chosen to best simulate low abundance values close to noise level (impute criteria: width 0.3 and down shift 1.8). A modified t-test with permutation based FDR statistics was applied (250 permutations; FDR = 0.01; S0 = 2). The data were filtered through a modified fasta file comprising only MG proteins.

1.7.2 Isotopic labelling data analysis

Triplex dimethyl labelling experiments in MaxQuant were performed using the built-in quantification algorithm[6] enabling the 'Match between runs' (time window 0.7 minutes) and 'Re-quantify' features. Light (+0 Da), medium (+4 Da) and heavy (+8 Da) intensities were selected for a triplex experiment. The outcome from MaxQuant was uploaded into Perseus.

To create the heat map of myristoylation levels in zebrafish development (Figure 2c), several filtering steps in Perseus were taken. The proteins were only included in the analysis if they had N-terminal glycine, contained at least two valid values per time point, and were either associated with a myristoylated peptide or were relatively enriched in samples originated from YnMyr over Myr-treated embryos (LFQ analysis).

1.7.3 Identification of lipid-modified peptides

PTM peptide search in MaxQuant was performed as described in section 1.7 applying the following variable modifications at any N-terminus: +463.2907 (reagent **2**, **3**); +633.4326 (reagent **4**); +406.2692 (reagent **5**); +520.3373 (reagent **6**); +624.3999 (reagent **7**). In addition, the minimum peptide length was reduced to 6 amino acids. MaxQuant utilizes a scoring algorithm when matching experimental MS/MS spectra with a library of theoretical spectra generated from *in silico* digestion of proteins within databases selected for the search.[6,7] The algorithm is used to evaluate the quality of peptide-spectrum matches (PSMs). A number of factors, such as the number of product ions detected or their intensities, contribute to the score. To each PSM, MaxQuant also attributes a Δscore, which is a difference between scores associated with the match to the best peptide candidate and

the second best match within the database; the higher the score and the Δscore, the more reliable the identification. MaxQuant utilizes a predefined FDR value (0.01 for all searches) to generate score thresholds separately for different populations of peptides, e.g. different peptide length, nature and number of modifications, etc. In order to reduce a possibility for a false peptide sequence assignment even further, relatively high Δscore thresholds (30) were applied for all lipid modified peptides in our analysis. See Supplementary Table 2 and 4 for the per-protein Δscore.

1.8 Proteomics data analysis with Peaks Suite

The data were processed with PEAKS7,[8] which as a default performs *de novo* peptide sequencing prior to database searches, in order to improve the accuracy of the results. The software also searches for common PTMs (PEAKS PTM) and point mutations (SPIDER). Samples originating from cell lines and zebrafish experiments were searched against the same reference Uniprot *Homo sapiens* and *Danio rerio* databases that were used in MaxQuant analyses. Trypsin (specific, up to three missed cleavages allowed) was selected for database searches, and no enzyme was chosen in *de novo* searches (up to 5 candidates per spectrum reported). The maximal mass error was set to 5 ppm for precursor ions and 0.01 Da for product ions. Carbamidomethylation was selected as a fixed modification, and methionine oxidation as well as the lipid-derived adduct (+463.2907 Da) to any amino acid at peptide N-terminus were set as variable modifications. The maximal number of modifications per peptide was set as five. The false discovery rate was set to 0.01 for peptides and minimum of 1 unique peptide per protein was required. For N-terminally modified peptides, b_1 ions were required. Proteins were grouped post analysis. Data were analyzed using Microsoft Office Excel 2007 and Perseus (version 1.5.0.9).

2. Synthetic procedures

General:

All reagents, amino acids and solvents for solid phase peptise synthesis (SPPS) were obtained from commercial sources (Sigma-Aldrich, Merck, AGTC Bioproducts) and were used without further purification. 3-Azidopropanoic acid and YnMyr were prepared according to previously published procedures. [9,10] Semi-preparative LC-MS purification was performed on a Waters system (2767 autosampler, 515 pump, 3100 ESI-MS) and HRMS was performed on a Waters LCT Premier Spectrometer operating in W mode ES +.

2.1 SPPS of **2** and **6**:

Biotin-PEG NovaTag™ resin (50.0 µmol, 1 eq) was swollen in DMF (2 mL, 30 min), Fmoc deprotected with 20% v/v piperidine in DMF (2 mL, 10 min x 3) and washed sequentially with DMF, DCM and DMF. Fmoc-Lysine(Mmt)-OH (3 eq), HATU (3 eq) and DIPEA (6 eq) were dissolved in DMF (1 mL), added to the deprotected resin and the reaction was shaken for 2 h, after which time the procedure was repeated. All subsequent couplings (5 eq each), i.e. Fmoc-6-Ahx-OH, Fmoc-Gly-OH, Fmoc-Arg(Pbf)-OH and 2-Azidoacetic acid (in case of **2**) or Fmoc-Lys(Boc)-OH, Fmoc-Ala-OH, and 3-Azidopropanoic acid (in case of **6**), were performed using DIC/HOBt activation (5 eq each, 30 min x 2). Following the removal of Mmt protecting group with 1 % TFA in DCM (10 min x 4) and wash (DCM, DMF), TAMRA (2 eq)

was activated for 10 min in DMF (1 mL) with DIC (2 eq) and HOAt (2 eq) and coupled to the peptidyl resin (2 h x 2).

2.2 SPPS of **3-5** and **7-9**

Fmoc-PEG Biotin NovaTag™ resin (50.0 µmol, 1 eq) was swollen in DMF (2 mL, 30 min), Fmoc deprotected with 20% v/v piperidine in DMF (2 mL, 10 min x 3) and washed sequentially with DMF, DCM and DMF. The couplings of Fmoc-Lys(Me_3Cl)-OH, Fmoc-Lys$(Me)_2$-OH*HCl and N_3-Arg(Pmc)-OH (2 eq) were carried out with DIC/HOBt (2 eq each, 2h x 2). All other amino acids (5 eq) were coupled with DIC/HOBt (5 eq each, 2h).

2.3 Peptide cleavage and purification

Crude products were cleaved from the resin with 95 % TFA, 2.5 % water and 2.5 % triisopropylsilane (3 h) and precipitated with cold TBME. The solids were pelleted by centrifugation (15 min, 4300 rpm, 4 °C) and washed three times with TBME. Pelleted products were dried and purified (≥ 95%) by semi-preparative LC-MS equipped with Waters X Select C18 columns running a gradient of MeOH (0.1% FA) in water (0.1% FA) (5-98 %, 20 min), with detection over 100–600 nm. The products were obtained by lyophilisation as bright pink (in case of **2** and **6**) or off-white amorphous solids. Products characterization and yields are given in Table S1 below.

3. Supplementary Figures and Tables

Figure S1. Mode of action for non-cleavable and cleavable reagents.

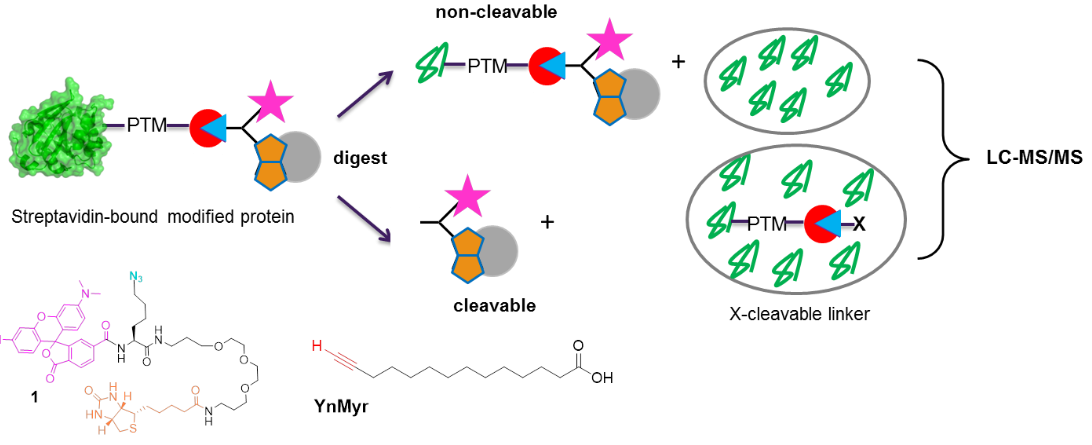

Figure S2. Comparison of the enrichment efficiency by a) in-gel fluorescence and b) WB with streptavidin HRP. First and second lanes in a) represent tagging and supernatant after enrichment on streptavidin beads, respectively. c) Modified peptide discovery per reagent.

a)

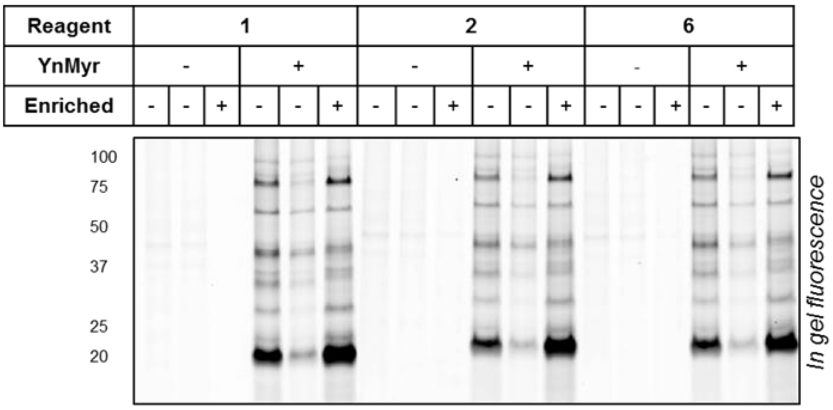

b)

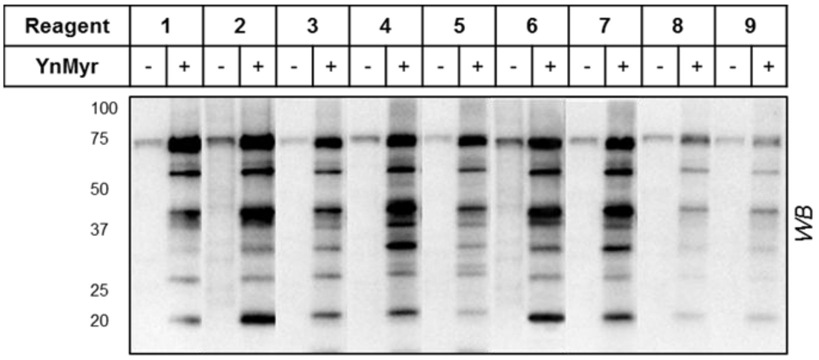

c)

Counts	2	3	4	6	7
total	33	19	9	24	1
7	+	+	+	+	+
6	+	+		+	
2	+		+	+	
5	+			+	
4	+	+			
1		+		+	
9	+				
1		+			
3				+	

Figure S3. Dose-dependent tagging of zebrafish embryos with YnMyr and efficient competition with the natural substrate, Myr. The embryos were pulsed (24 h) with YnMyr (and Myr) 48 hpf.

**YnMyr (µM)**	-	2	10	20	50		-	20	20	20	20	20
**Myr (µM)**	-	-	-	-	-		200	-	20	50	100	200

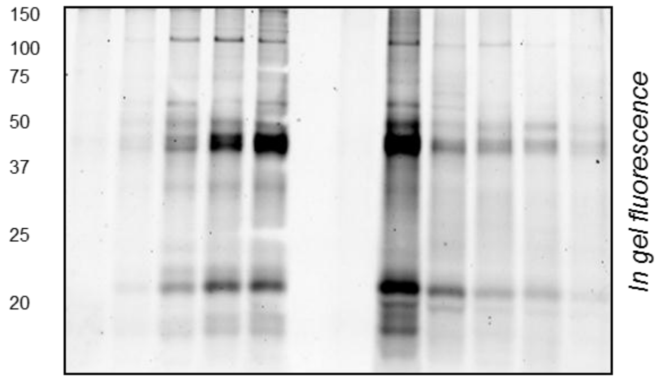

Table S1a. Characterisation of capture reagents **2**-**9**.

Reagent #	Yield [mg]	Yield [%]	Retention time [min]	HRMS		
				calculated	found	m/z
**2**	10.2	14	10.29/10.58*	1396.7202	1396.7368	$[M +H]^+$
**3**	36.3	85	9.44	856.4829	856.4835	$[M +H]^+$
**4**	33.7	66	8.40	514.3200	514.3157	$[M + 2H]^{2+}$
**5**	22.4	56	9.35	799.4614	799.4606	$[M +H]^+$
**6**	12	17	10.47/10.74*	727.3873	727.3892	$[M + 2H]^{2+}$
**7**	26.8	53	10.35	1017.5914	1017.5925	$[M +H]^+$
**8**	25	51	9.53	985.5499	985.5501	$[M +H]^+$
**9**	19.6	40	9.83	971.5350	971.5353	$[M +H]^+$

*TAMRA isotopes

Table S1b. LC traces of capture reagents **2**-**9**.

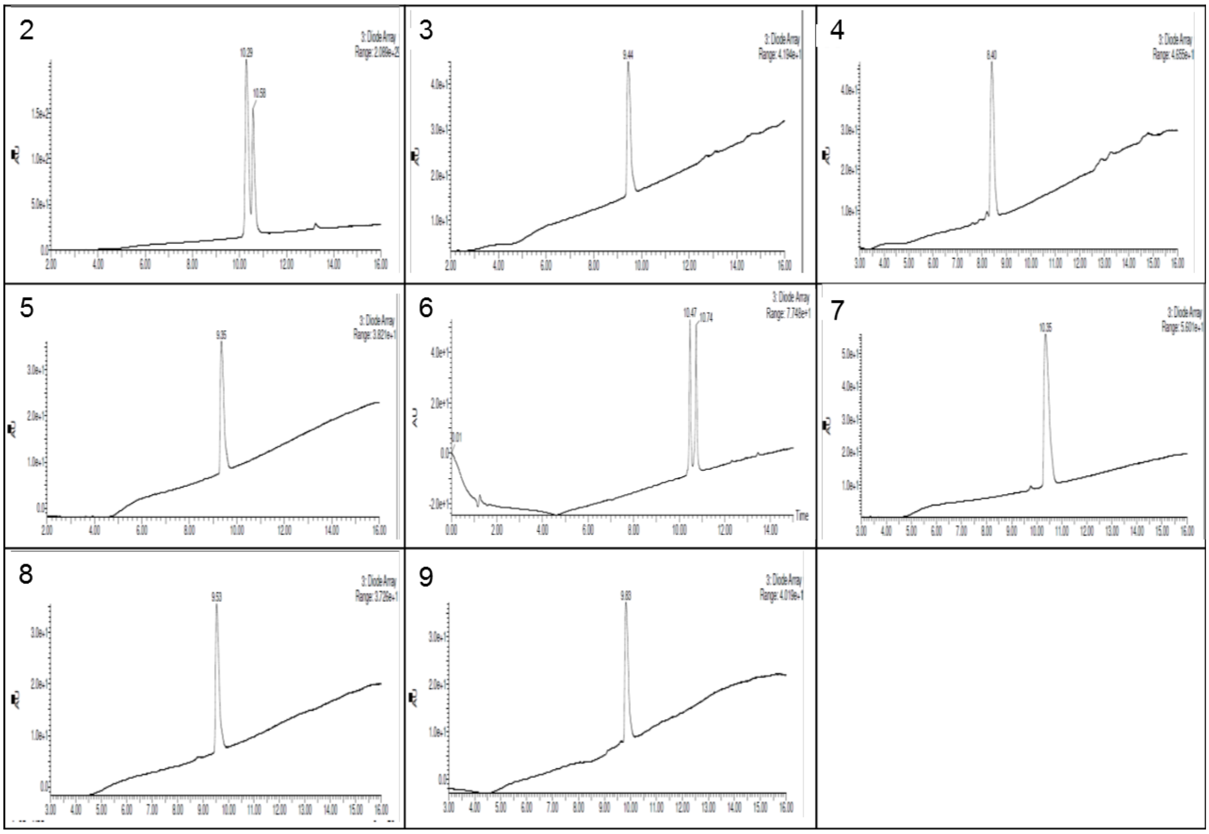

Table S2 : Identification of PTM peptides with reagents 2-4 and 6-7

Sheet 1 : Summary
The table shows sequences of detected PTM peptides, number of appearences within a triplicate, length, mass, and the corresponding protein details per reagent.

Sheets 2-5 : Total number of PTM peptide sequences per reagent
A complete list of PTM peptides found in all replicates and their characterists, e.g. charge, retention time, score, delta score.

PTM peptides are ordered by their sequences, Δ score treshold was set to 30 and minimum peptide length to 6 amino acids.

Reagent 2

#	PTM peptide sequence (# replicates found in)	Length (aa)	Mass	Proteins	Gene Names	Protein Names
1	GAAGSSALAR (3)	10	1322.74188	Q5VT66;Q5VT	MARC1	MOSC domain-containing protein 1, mitochondrial
2	GAFLDKPK (3)	8	1337.78196	P35813;P3581	PPM1A;PPM1E	Protein phosphatase 1A;Protein phosphatase 1B
3	GAGSSTEQR (2)	9	1354.69533	Q02952	AKAP12	A-kinase anchor protein 12
4	GAQFSK (3)	6	1099.61383	P29966	MARCKS	Myristoylated alanine-rich C-kinase substrate
5	GASSSSALAR (3)	10	1368.74736	Q969Z3;Q969Z	MARC2	MOSC domain-containing protein 2, mitochondrial
6	GAYLSQPNTVK (3)	11	1639.90459	O15355	PPM1G	Protein phosphatase 1G
7	GCCSSASSAAQSSK (1)	14	1849.84108	Q8WWI5;Q8W	SLC44A1	Choline transporter-like protein 1
8	GCCYSSENEDSDQDREER (2)	18	2698.09837	Q6IAA8;F5GX1	LAMTOR1	Ragulator complex protein LAMTOR1
9	GCFFSK (1)	6	1207.6172	O75695	RP2	Protein XRP2
10	GCSSSALNK (1)	9	1385.70853	Q6P6B1;Q6P6	C8orf47	Uncharacterized protein C8orf47
11	GCTLSAEDK (3)	9	1442.71876	P63096;C9J2Z	GNAI1;GNAI3	Guanine nucleotide-binding protein G(i) subunit alpha-1;Guanine nucleotide-binding protein G(k) subunit alpha
12	GCTVSAEDK (3)	9	1428.70311	P04899;P0489	GNAI2	Guanine nucleotide-binding protein G(i) subunit alpha-2
13	GCVQCK (2)	6	1213.60598	P06241;P0624	FYN	Tyrosine-protein kinase Fyn
14	GDVLSTHLDDAR (3)	12	1760.91695	Q96TA1	FAM129B	Niban-like protein 1
15	GGLFSR (1)	6	1098.62981	Q9C0E8;Q9C0	LNP;KIAA1715	Protein lunapark
16	GGSASSQLDEGK (2)	12	1597.806	Q9BZQ8	FAM129A	Protein Niban
17	GLTISSLFSR (3)	10	1542.88821	P18085;C9JPM	ARF4	ADP-ribosylation factor 4
18	GLTVSALFSR (1)	10	1512.87765	P84085;C9J1Z	ARF5	ADP-ribosylation factor 5
19	GNAAAAK (3)	7	1064.60908	P17612;K7ENJ	PRKACA	cAMP-dependent protein kinase catalytic subunit alpha
20	GNAATAK (1)	7	1094.61964	P22694;P2269	PRKACB	cAMP-dependent protein kinase catalytic subunit beta
21	GNEASYPLEMCSHFDADEIKR (3)	21	2931.36436	P63098;D3YTA	PPP3R1	Calcineurin subunit B type 1
22	GNIFANLFK (2)	9	1485.84562	P84077	ARF1	ADP-ribosylation factor 1
23	GNIFGNLLK (3)	9	1437.84562	P61204;F5H42	ARF3	ADP-ribosylation factor 3
24	GNSASNIVSPQEALPGR (3)	17	2159.14471	Q9UJ68-5;Q9U	MSRA	Mitochondrial peptide methionine sulfoxide reductase
25	GQQISDQTQLVINK (3)	14	2034.12219	Q8WVD5;E9PL	RNF141	RING finger protein 141
26	GQSQSGGHGPGGGK (3)	14	1672.83936	P62191	PSMC1	26S protease regulatory subunit 4
27	GSENSALK (1)	8	1267.68845	X6RHX1;Q8IZE	SCYL3	Protein-associating with the carboxyl-terminal domain of ezrin
28	GSQHSAAAR (3)	9	1346.71673	Q9BVX2;F8W1	TMEM106C	Transmembrane protein 106C
29	GSQSSK (2)	6	1055.57236	P49006	MARCKSL1	MARCKS-related protein
30	GSSQSVEIPGGGTEGYHVLR (3)	20	2492.27718	Q9H8Y8;F8WF	GORASP2	Golgi reassembly-stacking protein 2
31	GSTLGCHR (1)	8	1349.69864	Q7L9B9	EEPD1	Endonuclease/exonuclease/phosphatase family domain-containing protein 1
32	GTVHAR (2)	6	1102.63596	Q9Y512	SAMM50	Sorting and assembly machinery component 50 homolog
33	GYEESEGHNTPKLKNQR (2)	17	2449.24622	Q8TE76-2;Q8T	MORC4	MORC family CW-type zinc finger protein 4

Reagent 3

#	PTM peptide sequence (# replicates found in)	Length (aa)	Mass	Proteins	Gene Names	Protein Names
1	GAAHSASEEVR (2)	11	1575.81175	Q96BS2-3;Q96	TESC	Calcineurin B homologous protein 3
2	GAGSSTEQR (3)	9	1354.69533	Q02952	AKAP12	A-kinase anchor protein 12
3	GAQFSK (3)	6	1099.61383	P29966	MARCKS	Myristoylated alanine-rich C-kinase substrate
4	GASSSSALAR (3)	10	1368.74736	Q969Z3;Q969Z	MARC2	MOSC domain-containing protein 2, mitochondrial
5	GAYLSQPNTVK (3)	11	1639.90459	O15355	PPM1G	Protein phosphatase 1G
6	GCCYSSENEDSDQDREER (2)	18	2698.09837	Q6IAA8;F5GX1	LAMTOR1	Ragulator complex protein LAMTOR1
7	GCFFSK (1)	6	1207.6172	O75695	RP2	Protein XRP2
8	GCTLSAEDK (3)	9	1442.71876	P63096;C9J2Z	GNAI1;GNAI3	Guanine nucleotide-binding protein G(i) subunit alpha-1;Guanine nucleotide-binding protein G(k) subunit alpha
9	GCTVSAEDK (2)	9	1428.70311	P04899;P0489	GNAI2	Guanine nucleotide-binding protein G(i) subunit alpha-2

10	GDVLSTHLDDAR (3)	12	1760.91695	Q96TA1	FAM129B	Niban-like protein 1
11	GGSASSQLDEGK (1)	12	1597.806	Q9BZQ8	FAM129A	Protein Niban
12	GNAAAAK (3)	7	1064.60908	P17612;K7ENJ	PRKACA	cAMP-dependent protein kinase catalytic subunit alpha
13	GNAATAK (3)	7	1094.61964	P22694;P2269	PRKACB	cAMP-dependent protein kinase catalytic subunit beta
14	GNIFANLFK (2)	9	1485.84562	P84077	ARF1	ADP-ribosylation factor 1
15	GNSALR (1)	6	1079.61998	Q8N9N7;H3BS	LRRC57	Leucine-rich repeat-containing protein 57
16	GQQISDQTQLVINK (2)	14	2034.12219	Q8WVD5;E9PL	RNF141	RING finger protein 141
17	GQSQSGGHGPGGGK (3)	14	1672.83936	P62191	PSMC1	26S protease regulatory subunit 4
18	GSQSSK (2)	6	1055.57236	P49006	MARCKSL1	MARCKS-related protein
19	GSSQSVEIPGGGTEGYHVLR (3)	20	2492.27718	Q9H8Y8;F8WF	GORASP2	Golgi reassembly-stacking protein 2

Reagent 4

#	PTM peptide sequence (# replicates found in)	Length (aa)	Mass	Proteins	Gene Names	Protein Names
1	GAQFSK (3)	6	1269.75574	P29966	MARCKS	Myristoylated alanine-rich C-kinase substrate
2	GAYLSQPNTVK (2)	11	1810.0465	O15355	PPM1G	Protein phosphatase 1G
3	GCTLSAEDK (3)	9	1612.86068	P63096;C9J2Z	GNAI1;GNAI3	Guanine nucleotide-binding protein G(i) subunit alpha-1;Guanine nucleotide-binding protein G(k) subunit alpha
4	GLTISSLFSR (1)	10	1713.03012	P18085;C9JPM	ARF4	ADP-ribosylation factor 4
5	GNAAAAK (3)	7	1234.75099	P17612;K7ENJ	PRKACA	cAMP-dependent protein kinase catalytic subunit alpha
6	GNIFANLFK (1)	9	1655.98753	P84077	ARF1	ADP-ribosylation factor 1
7	GNIFGNLLK (1)	9	1607.98753	P61204;F5H42	ARF3	ADP-ribosylation factor 3
8	GQSQSGGHGPGGGK (1)	14	1842.98128	P62191	PSMC1	26S protease regulatory subunit 4
9	GSQSSK (1)	6	1225.71427	P49006	MARCKSL1	MARCKS-related protein

Reagent 6

#	PTM peptide sequence (# replicates found in)	Length (aa)	Mass	Proteins	Gene Names	Protein Names
1	GAFLDKPK (3)	8	1394.82857	P35813;P3581	PPM1A;PPM1B	Protein phosphatase 1A;Protein phosphatase 1B
2	GAGSSTEQR (3)	9	1411.74194	Q02952	AKAP12	A-kinase anchor protein 12
3	GAQFSK (3)	6	1156.66044	P29966	MARCKS	Myristoylated alanine-rich C-kinase substrate
4	GASSSSALAR (3)	10	1425.79398	Q969Z3;Q969Z	MARC2	MOSC domain-containing protein 2, mitochondrial
5	GAYLSQPNTVK (3)	11	1696.95121	O15355	PPM1G	Protein phosphatase 1G
6	GCCSSASSAAQSSK (1)	14	1906.8877	Q8WWI5;Q8W	SLC44A1	Choline transporter-like protein 1
7	GCCYSSENEDSDQDREER (3)	18	2755.14499	Q6IAA8;F5GX1	LAMTOR1	Ragulator complex protein LAMTOR1
8	GCTLSAEDK (3)	9	1499.76538	P63096;C9J2Z	GNAI1;GNAI3	Guanine nucleotide-binding protein G(i) subunit alpha-1;Guanine nucleotide-binding protein G(k) subunit alpha
9	GDVLSTHLDDAR (3)	12	1817.96356	Q96TA1	FAM129B	Niban-like protein 1
10	GGAVSAGEDNDELIDNLK (1)	18	2336.18597	Q9NV79;Q9NV	PCMTD2	Protein-L-isoaspartate O-methyltransferase domain-containing protein 2
11	GGSASSQLDEGK (2)	12	1654.85261	Q9BZQ8	FAM129A	Protein Niban
12	GLTISSLFSR (3)	10	1599.93483	P18085;C9JPM	ARF4	ADP-ribosylation factor 4
13	GLTVSALFSR (2)	10	1569.92426	P84085;C9J1Z	ARF5	ADP-ribosylation factor 5
14	GNAAAAK (3)	7	1121.65569	P17612;K7ENJ	PRKACA	cAMP-dependent protein kinase catalytic subunit alpha
15	GNEASYPLEMCSHFDADEIKR (2)	21	2988.41098	P63098;D3YTA	PPP3R1	Calcineurin subunit B type 1
16	GNIFANLFK (2)	9	1542.89223	P84077	ARF1	ADP-ribosylation factor 1
17	GNIFGNLLK (2)	9	1494.89223	P61204;F5H42	ARF3	ADP-ribosylation factor 3
18	GNLFGR (1)	6	1182.68733	Q96FZ7	CHMP6	Charged multivesicular body protein 6
19	GNSALR (1)	6	1136.66659	Q8N9N7;H3BS	LRRC57	Leucine-rich repeat-containing protein 57
20	GQSQSGGHGPGGGK (3)	14	1729.88598	P62191	PSMC1	26S protease regulatory subunit 4
21	GSEQSSEAESRPNDLNSSVTPSPAK (1)	25	3093.52145	Q969J3	LOH12CR1	Loss of heterozygosity 12 chromosomal region 1 protein
22	GSQHSAAAR (1)	9	1403.76335	Q9BVX2;F8W1	TMEM106C	Transmembrane protein 106C

23	GSQSSK (1)	6	1112.61897	P49006	MARCKSL1	MARCKS-related protein
24	GSSQSVEIPGGGTEGYHVLR (3)	20	2549.3238	Q9H8Y8;F8WF	GORASP2	Golgi reassembly-stacking protein 2

Reagent 7

#	PTM peptide sequence (# replicates found in)	Length (aa)	Mass	Proteins	Gene Names	Protein Names
1	GNAAAAKKGSEQESVKEF (1)	18	2474.32	P17612;K7ENJ	PRKACA	cAMP-dependent protein kinase catalytic subunit alpha

Reagent 2

Replicate	Sequence	Gene Names	Protein Names	Charge	Length	m/z	Retention time	PEP	MS/MS Count	Score	Delta score	Intensity
1	GAAGSSALAR	MARC1	MOSC domain-containing protein 1, mitochondrial	3	10	441.9212	67.184	0.003083	1	57.532	37.011	2884300
2	GAAGSSALAR	MARC1	MOSC domain-containing protein 1, mitochondrial	3	10	441.9212	66.841	0.003044	1	57.785	37.265	1938300
3	GAAGSSALAR	MARC1	MOSC domain-containing protein 1, mitochondrial	3	10	441.9212	64.647	0.002494	1	67.334	42.335	NaN
1	GAFLDKPK	PPM1A;PPM1B	Protein phosphatase 1A;Protein phosphatase 1B	3	8	446.9346	76.685	0.030026	2	71.379	52.285	13154000
2	GAFLDKPK	PPM1A;PPM1B	Protein phosphatase 1A;Protein phosphatase 1B	3	8	446.9346	76.125	0.035093	1	81.296	56.298	35463000
3	GAFLDKPK	PPM1A;PPM1B	Protein phosphatase 1A;Protein phosphatase 1B	3	8	446.9346	73.532	0.030026	1	71.379	52.977	28569000
2	GAGSSTEQR	AKAP12	A-kinase anchor protein 12	3	9	452.5724	56.747	0.002913	2	83.948	60.424	39805000
3	GAGSSTEQR	AKAP12	A-kinase anchor protein 12	3	9	452.5724	54.691	0.005583	1	91.087	45.693	39660000
2	GAQFSK	MARCKS	Myristoylated alanine-rich C-kinase substrate	2	6	550.8142	68.685	0.035196	1	105.2	55.889	7.41E+08
2	GAQFSK	MARCKS	Myristoylated alanine-rich C-kinase substrate	3	6	367.5452	68.699	0.014209	1	93.593	43.453	2434000
3	GAQFSK	MARCKS	Myristoylated alanine-rich C-kinase substrate	2	6	550.8142	66.35	0.031716	1	103.02	53.706	7.21E+08
3	GAQFSK	MARCKS	Myristoylated alanine-rich C-kinase substrate	3	6	367.5452	66.369	0.038915	1	87.582	32.687	NaN
1	GASSSSALAR	MARC2	MOSC domain-containing protein 2, mitochondrial	3	10	457.2564	64.503	0.001525	1	85.533	67.714	13816000
2	GASSSSALAR	MARC2	MOSC domain-containing protein 2, mitochondrial	3	10	457.2564	64.176	0.005325	1	57.347	45.111	14147000
3	GASSSSALAR	MARC2	MOSC domain-containing protein 2, mitochondrial	3	10	457.2564	61.94	0.001607	1	86.898	69.079	10374000
1	GAYLSQPNTVK	PPM1G	Protein phosphatase 1G	2	11	820.9596	78.544	0.000103	1	94.692	75.034	9226600
1	GAYLSQPNTVK	PPM1G	Protein phosphatase 1G	3	11	547.6421	78.599	5.69E-06	1	104.82	86.489	39582000
2	GAYLSQPNTVK	PPM1G	Protein phosphatase 1G	3	11	547.6421	78.19	3.84E-06	2	108.9	90.568	59611000
2	GAYLSQPNTVK	PPM1G	Protein phosphatase 1G	2	11	820.9596	78.19	1.28E-07	1	120.86	94.413	13343000
3	GAYLSQPNTVK	PPM1G	Protein phosphatase 1G	3	11	547.6421	75.922	7.49E-08	3	119.27	97.246	49443000
3	GAYLSQPNTVK	PPM1G	Protein phosphatase 1G	2	11	820.9596	75.913	0.000509	1	73.632	56.464	11649000
3	GCCSSASSAAQSSK	SLC44A1	Choline transporter-like protein 1	3	14	617.621	53.575	0.000359	1	70.26	67.162	9626400
2	GCCYSSENEDSDQDREER	LAMTOR1	Ragulator complex protein LAMTOR1	4	18	675.5319	55.798	2.77E-07	1	78.441	75.107	36535000
2	GCCYSSENEDSDQDREER	LAMTOR1	Ragulator complex protein LAMTOR1	3	18	900.3734	55.797	0.010038	1	36.637	34.227	28369000
3	GCCYSSENEDSDQDREER	LAMTOR1	Ragulator complex protein LAMTOR1	4	18	675.5319	53.82	2.57E-05	2	47.942	47.058	32144000
3	GCFFSK	RP2	Protein XRP2	2	6	604.8159	84.948	0.033374	1	91.093	72.137	5496900
3	GCSSSALNK	C8orf47	Uncharacterized protein C8orf47	3	9	462.9101	59.484	0.020325	1	46.447	37.534	5065600
1	GCTLSAEDK	GNAI1;GNAI3	Guanine nucleotide-binding protein G(i) subunit alpha-1;Guan	3	9	481.9135	68.781	0.00469	2	63.184	50.147	25780000
1	GCTLSAEDK	GNAI1;GNAI3	Guanine nucleotide-binding protein G(i) subunit alpha-1;Guan	2	9	722.3667	68.785	1.70E-20	1	177.06	120.66	69231000
2	GCTLSAEDK	GNAI1;GNAI3	Guanine nucleotide-binding protein G(i) subunit alpha-1;Guan	3	9	481.9135	68.582	0.005242	2	88.495	71.256	24130000
2	GCTLSAEDK	GNAI1;GNAI3	Guanine nucleotide-binding protein G(i) subunit alpha-1;Guan	2	9	722.3667	68.587	5.93E-09	1	153.97	106.08	59605000
3	GCTLSAEDK	GNAI1;GNAI3	Guanine nucleotide-binding protein G(i) subunit alpha-1;Guan	2	9	722.3667	66.348	3.46E-05	2	137.98	104.08	79279000
3	GCTLSAEDK	GNAI1;GNAI3	Guanine nucleotide-binding protein G(i) subunit alpha-1;Guan	3	9	481.9135	66.349	0.003008	2	81.95	67.281	31563000
1	GCTVSAEDK	GNAI2	Guanine nucleotide-binding protein G(i) subunit alpha-2	2	9	715.3588	63.792	0.00316	1	109.66	50.683	66463000
1	GCTVSAEDK	GNAI2	Guanine nucleotide-binding protein G(i) subunit alpha-2	3	9	477.2416	63.791	0.003008	1	72.325	34.049	19750000
2	GCTVSAEDK	GNAI2	Guanine nucleotide-binding protein G(i) subunit alpha-2	2	9	715.3588	63.62	8.24E-39	1	190.02	108.72	54861000
3	GCTVSAEDK	GNAI2	Guanine nucleotide-binding protein G(i) subunit alpha-2	2	9	715.3588	61.512	0.002547	2	111.46	59.949	56730000
3	GCTVSAEDK	GNAI2	Guanine nucleotide-binding protein G(i) subunit alpha-2	3	9	477.2416	61.527	0.003008	1	72.325	31.601	16127000
2	GCVQCK	FYN	Tyrosine-protein kinase Fyn	2	6	607.8103	62.174	0.032801	1	103.7	31.08	11520000
3	GCVQCK	FYN	Tyrosine-protein kinase Fyn	2	6	607.8103	59.989	0.032801	1	103.7	31.702	11158000
1	GDVLSTHLDDAR	FAM129B	Niban-like protein 1	3	12	587.9796	77.154	1.56E-06	2	107.17	88.831	14723000
2	GDVLSTHLDDAR	FAM129B	Niban-like protein 1	3	12	587.9796	76.796	2.23E-05	2	92.19	80.027	5723100
3	GDVLSTHLDDAR	FAM129B	Niban-like protein 1	3	12	587.9796	74.457	1.35E-26	2	167.71	145.72	7949500
2	GGLFSR	LNP;KIAA1715	Protein lunapark	2	6	550.3222	86.438	0.027732	1	98.03	37.025	6482000
1	GGSASSQLDEGK	FAM129A	Protein Niban	3	12	533.6093	62.063	0.000346	1	69.864	52.95	9933600
2	GGSASSQLDEGK	FAM129A	Protein Niban	3	12	533.6093	61.98	0.001177	1	54.65	45.163	7937800

1	GLTISSLFSR	ARF4	ADP-ribosylation factor 4	3	10	515.3033	101.85	0.001984	1	64.82	55.165	15788000
2	GLTISSLFSR	ARF4	ADP-ribosylation factor 4	3	10	515.3033	100.76	0.001063	1	74.841	63.677	9146500
3	GLTISSLFSR	ARF4	ADP-ribosylation factor 4	3	10	515.3033	99.709	0.001063	1	74.841	61.032	6168900
2	GLTVSALFSR	ARF5	ADP-ribosylation factor 5	3	10	505.2998	98.396	0.017461	1	43.308	31.261	1606800
1	GNAAAAK	PRKACA	cAMP-dependent protein kinase catalytic subunit alpha	2	7	533.3118	58.693	0.023168	2	120.52	70.211	1.64E+08
2	GNAAAAK	PRKACA	cAMP-dependent protein kinase catalytic subunit alpha	2	7	533.3118	58.395	0.028056	2	120.65	70.335	1.08E+08
3	GNAAAAK	PRKACA	cAMP-dependent protein kinase catalytic subunit alpha	2	7	533.3118	56.207	0.023854	1	124.34	74.033	1.23E+08
1	GNAATAK	PRKACB	cAMP-dependent protein kinase catalytic subunit beta	2	7	548.3171	58.624	0.033747	1	98.458	52.107	37646000
1	GNEASYPLEMCSHFDADEIKR	PPP3R1	Calcineurin subunit B type 1	4	21	733.8484	70.792	1.10E-08	1	63.869	61.225	17191000
2	GNEASYPLEMCSHFDADEIKR	PPP3R1	Calcineurin subunit B type 1	4	21	733.8484	70.708	1.48E-08	2	57.806	53.5	17061000
3	GNEASYPLEMCSHFDADEIKR	PPP3R1	Calcineurin subunit B type 1	4	21	733.8484	68.137	4.40E-21	1	98.543	93.903	22346000
3	GNEASYPLEMCSHFDADEIKR	PPP3R1	Calcineurin subunit B type 1	4	21	733.8484	68.803	1.57E-20	1	95.622	84.421	21182000
1	GNIFANLFK	ARF1	ADP-ribosylation factor 1	2	9	743.9301	102.27	4.25E-11	1	161.79	102.81	29042000
1	GNIFANLFK	ARF1	ADP-ribosylation factor 1	3	9	496.2891	102.27	0.004156	1	96.027	69.855	28084000
2	GNIFANLFK	ARF1	ADP-ribosylation factor 1	3	9	496.2891	102.05	0.00622	1	51.066	39.902	65172000
1	GNIFGNLLK	ARF3	ADP-ribosylation factor 3	2	9	719.9301	101.32	0.008381	1	80.165	47.751	5208500
2	GNIFGNLLK	ARF3	ADP-ribosylation factor 3	2	9	719.9301	99.822	0.000475	1	117.89	76.442	9658900
3	GNIFGNLLK	ARF3	ADP-ribosylation factor 3	2	9	719.9301	98.71	0.000475	2	117.89	76.442	15748000
3	GNIFGNLLK	ARF3	ADP-ribosylation factor 3	3	9	480.2891	98.702	0.006196	1	58.23	36.544	8220800
1	GNSASNIVSPQEALPGR	MSRA	Mitochondrial peptide methionine sulfoxide reductase	3	17	720.7222	72.588	0.000148	1	48.623	45.582	5068100
2	GNSASNIVSPQEALPGR	MSRA	Mitochondrial peptide methionine sulfoxide reductase	3	17	720.7222	72.547	0.000109	1	56.817	51.102	5493700
3	GNSASNIVSPQEALPGR	MSRA	Mitochondrial peptide methionine sulfoxide reductase	3	17	720.7222	70.448	6.28E-05	1	69.979	62.468	7206100
1	GQQISDQTQLVINK	RNF141	RING finger protein 141	3	14	679.048	74.782	0.000639	1	56.139	35.903	16338000
2	GQQISDQTQLVINK	RNF141	RING finger protein 141	3	14	679.048	74.869	7.73E-05	1	76.01	44.938	14600000
3	GQQISDQTQLVINK	RNF141	RING finger protein 141	3	14	679.048	72.635	3.16E-06	1	81.703	49.995	19294000
1	GQSQSGGHGPGGGK	PSMC1	26S protease regulatory subunit 4	3	14	558.6204	46.794	1.51E-13	2	125.22	104.52	2.2E+08
2	GQSQSGGHGPGGGK	PSMC1	26S protease regulatory subunit 4	3	14	558.6204	45.701	6.80E-25	2	151.74	134.27	1.53E+08
3	GQSQSGGHGPGGGK	PSMC1	26S protease regulatory subunit 4	3	14	558.6204	43.4	8.36E-22	2	140.31	128.07	1.8E+08
3	GSENSALK	SCYL3	Protein-associating with the carboxyl-terminal domain of ezri	2	8	634.8515	60.576	0.025265	1	77.732	38.94	6497600
1	GSQHSAAAR	TMEM106C	Transmembrane protein 106C	3	9	449.9129	47.001	1.93E-05	1	131.22	114.67	21016000
2	GSQHSAAAR	TMEM106C	Transmembrane protein 106C	3	9	449.9129	46.094	0.01587	1	49.418	37.371	18727000
3	GSQHSAAAR	TMEM106C	Transmembrane protein 106C	3	9	449.9129	43.739	5.35E-05	1	124.74	105.94	16846000
1	GSQSSK	MARCKSL1	MARCKS-related protein	2	6	528.7935	54.081	0.000145	1	158.12	68.443	2.48E+08
3	GSQSSK	MARCKSL1	MARCKS-related protein	2	6	528.7935	51.538	3.10E-14	2	179.16	71.411	1.29E+08
1	GSSQSVEIPGGGTEGYHVLR	GORASP2	Golgi reassembly-stacking protein 2	3	20	831.7663	68.449	1.96E-16	2	95.205	87.291	35321000
1	GSSQSVEIPGGGTEGYHVLR	GORASP2	Golgi reassembly-stacking protein 2	4	20	624.0766	68.45	1.11E-13	2	90.718	86.877	36201000
2	GSSQSVEIPGGGTEGYHVLR	GORASP2	Golgi reassembly-stacking protein 2	3	20	831.7663	68.095	5.63E-45	3	144.17	136.52	32153000
2	GSSQSVEIPGGGTEGYHVLR	GORASP2	Golgi reassembly-stacking protein 2	4	20	624.0766	68.093	2.52E-18	2	99.413	95.435	27167000
3	GSSQSVEIPGGGTEGYHVLR	GORASP2	Golgi reassembly-stacking protein 2	3	20	831.7663	65.825	1.44E-32	2	131.54	120.42	51902000
3	GSSQSVEIPGGGTEGYHVLR	GORASP2	Golgi reassembly-stacking protein 2	4	20	624.0766	65.828	4.75E-26	2	116.6	113.79	40220000
3	GSTLGCHR	EEPD1	Endonuclease/exonuclease/phosphatase family domain-conta	3	8	450.9068	57.497	0.01324	1	67.169	51.73	4516900
2	GTVHAR	SAMM50	Sorting and assembly machinery component 50 homolog	3	6	368.5526	54.235	0.032985	2	112.27	46.936	4403300
3	GTVHAR	SAMM50	Sorting and assembly machinery component 50 homolog	3	6	368.5526	51.88	0.015131	1	91.093	41.782	4278600
2	GYEESEGHNTPKLKNQR	MORC4	MORC family CW-type zinc finger protein 4	3	17	817.4227	42.539	0.000868	1	43.592	31.391	43529000
3	GYEESEGHNTPKLKNQR	MORC4	MORC family CW-type zinc finger protein 4	3	17	817.4227	40.646	0.002407	3	37.77	31.017	51875000

Reagent 3

Replicate	Sequence	Gene Names	Protein Names	Charge	Length	m/z	Retention time	PEP	MS/MS Count	Score	Delta score	Intensity
1	GAAHSASEEVR	TESC	Calcineurin B homologous protein 3	3	11	526.2779	50.495	0.016373	1	48.998	39.641	3340800
2	GAAHSASEEVR	TESC	Calcineurin B homologous protein 3	3	11	526.2779	50.03	0.000681	1	69.65	52.42	4898400
1	GAGSSTEQR	AKAP12	A-kinase anchor protein 12	3	9	452.5724	54.341	0.016578	1	97.857	53.09	18692000
2	GAGSSTEQR	AKAP12	A-kinase anchor protein 12	3	9	452.5724	53.971	0.012178	2	98.582	65.066	22613000
3	GAGSSTEQR	AKAP12	A-kinase anchor protein 12	3	9	452.5724	53.935	0.01217	2	73.233	51.102	7851600
1	GAQFSK	MARCKS	Myristoylated alanine-rich C-kinase substrate	2	6	550.8142	65.962	0.00911	2	143.37	79.696	2.05E+08
2	GAQFSK	MARCKS	Myristoylated alanine-rich C-kinase substrate	2	6	550.8142	65.643	0.010655	1	97.473	52.527	3E+08
3	GAQFSK	MARCKS	Myristoylated alanine-rich C-kinase substrate	2	6	550.8142	65.636	0.00911	2	114.19	62.197	1.17E+08
1	GASSSSALAR	MARC2	MOSC domain-containing protein 2, mitochondrial	3	10	457.2564	61.497	0.014435	1	57.532	39.712	3057400
2	GASSSSALAR	MARC2	MOSC domain-containing protein 2, mitochondrial	3	10	457.2564	61.198	0.005049	1	67.207	52.533	4242400
3	GASSSSALAR	MARC2	MOSC domain-containing protein 2, mitochondrial	3	10	457.2564	61.226	0.016067	1	43.297	35.254	3299900
1	GAYLSQPNTVK	PPM1G	Protein phosphatase 1G	3	11	547.6421	75.66	3.78E-05	2	110.08	88.049	23467000
2	GAYLSQPNTVK	PPM1G	Protein phosphatase 1G	3	11	547.6421	75.3	1.96E-06	2	121.36	101.7	23161000
3	GAYLSQPNTVK	PPM1G	Protein phosphatase 1G	3	11	547.6421	75.325	0.021131	1	45.653	37.253	4710200
1	GCCYSSENEDSDQDREER	LAMTOR1	Ragulator complex protein LAMTOR1	4	18	675.5319	53.655	0.000372	2	43.326	42.514	16076000
2	GCCYSSENEDSDQDREER	LAMTOR1	Ragulator complex protein LAMTOR1	4	18	675.5319	53.217	7.73E-06	1	60.062	56.728	22211000
2	GCFFSK	RP2	Protein XRP2	2	6	604.8159	84.469	0.039222	1	67.377	55.645	2926800
1	GCTLSAEDK	GNAI1;GNAI3	Guanine nucleotide-binding protein G(i) subunit alpha-1;Guanine	2	9	722.3667	66.01	2.21E-05	1	150.61	102.71	21418000
2	GCTLSAEDK	GNAI1;GNAI3	Guanine nucleotide-binding protein G(i) subunit alpha-1;Guanine	2	9	722.3667	65.726	3.25E-05	2	125.97	88.241	27920000
2	GCTLSAEDK	GNAI1;GNAI3	Guanine nucleotide-binding protein G(i) subunit alpha-1;Guanine	3	9	481.9135	65.732	0.019936	2	61.78	44.54	10530000
3	GCTLSAEDK	GNAI1;GNAI3	Guanine nucleotide-binding protein G(i) subunit alpha-1;Guanine	3	9	481.9135	65.696	0.022999	1	60.398	47.39	4791100
3	GCTLSAEDK	GNAI1;GNAI3	Guanine nucleotide-binding protein G(i) subunit alpha-1;Guanine	2	9	722.3667	65.692	2.05E-05	1	134.25	95.944	10894000
1	GCTVSAEDK	GNAI2	Guanine nucleotide-binding protein G(i) subunit alpha-2	2	9	715.3588	61.14	0.00034	1	121.73	62.752	23495000
2	GCTVSAEDK	GNAI2	Guanine nucleotide-binding protein G(i) subunit alpha-2	2	9	715.3588	60.847	1.81E-10	1	168.83	92.763	26304000
1	GDVLSTHLDDAR	FAM129B	Niban-like protein 1	3	12	587.9796	74.389	0.001672	1	70.089	49.883	2790900
2	GDVLSTHLDDAR	FAM129B	Niban-like protein 1	3	12	587.9796	73.94	0.001781	2	66.267	56.262	3283900
3	GDVLSTHLDDAR	FAM129B	Niban-like protein 1	3	12	587.9796	74.138	0.030455	1	39.73	34.632	909010
1	GGSASSQLDEGK	FAM129A	Protein Niban	3	12	533.6093	59.636	0.000453	1	77.597	55.569	3860000
1	GNAAAAK	PRKACA	cAMP-dependent protein kinase catalytic subunit alpha	2	7	533.3118	55.909	0.007815	1	97.602	52.799	36992000
2	GNAAAAK	PRKACA	cAMP-dependent protein kinase catalytic subunit alpha	2	7	533.3118	55.571	0.006646	2	110.63	65.83	37826000
3	GNAAAAK	PRKACA	cAMP-dependent protein kinase catalytic subunit alpha	2	7	533.3118	55.502	0.010475	1	91.906	51.544	21464000
1	GNAATAK	PRKACB	cAMP-dependent protein kinase catalytic subunit beta	2	7	548.3171	55.862	0.01377	1	78.324	40.765	8985900
2	GNAATAK	PRKACB	cAMP-dependent protein kinase catalytic subunit beta	2	7	548.3171	55.511	0.009746	2	93.374	57.568	12651000
3	GNAATAK	PRKACB	cAMP-dependent protein kinase catalytic subunit beta	2	7	548.3171	55.48	0.013248	1	82.452	47.347	6494000
2	GNIFANLFK	ARF1	ADP-ribosylation factor 1	2	9	743.9301	101.93	2.87E-05	1	128.36	60.138	9730100
2	GNIFANLFK	ARF1	ADP-ribosylation factor 1	3	9	496.2891	101.93	0.02003	1	61.679	39.719	9505500
3	GNIFANLFK	ARF1	ADP-ribosylation factor 1	2	9	743.9301	101.92	0.0014	1	115.71	75.363	7863700
2	GNSALR	LRRC57	Leucine-rich repeat-containing protein 57	2	6	540.8173	62.132	0.012436	1	110.9	31.448	22782000
1	GQQISDQTQLVINK	RNF141	RING finger protein 141	3	14	679.048	72.485	0.008866	1	49.298	30.054	7672500
2	GQQISDQTQLVINK	RNF141	RING finger protein 141	3	14	679.048	72.227	2.96E-09	1	96.866	65.158	5117100
1	GQSQSGGHGPGGGK	PSMC1	26S protease regulatory subunit 4	3	14	558.6204	42.915	4.79E-13	1	69.345	69.345	59857000
2	GQSQSGGHGPGGGK	PSMC1	26S protease regulatory subunit 4	3	14	558.6204	42.335	1.21E-11	2	122.66	107.72	1.13E+08
3	GQSQSGGHGPGGGK	PSMC1	26S protease regulatory subunit 4	3	14	558.6204	42.357	4.49E-08	1	60.937	60.937	13022000
2	GSQSSK	MARCKSL1	MARCKS-related protein	2	6	528.7935	50.695	0.026616	1	143.02	43.1	25919000
3	GSQSSK	MARCKSL1	MARCKS-related protein	2	6	528.7935	50.681	0.016832	1	124.37	42.992	13621000

1	GSSQSVEIPGGGTEGYHVLR	GORASP2	Golgi reassembly-stacking protein 2	3	20	831.7663	65.585	1.64E-05	2	72.359	68.609	12427000
1	GSSQSVEIPGGGTEGYHVLR	GORASP2	Golgi reassembly-stacking protein 2	4	20	624.0766	65.581	4.72E-08	2	78.78	74.024	8397600
2	GSSQSVEIPGGGTEGYHVLR	GORASP2	Golgi reassembly-stacking protein 2	4	20	624.0766	65.212	5.75E-24	2	107.72	102.51	24007000
2	GSSQSVEIPGGGTEGYHVLR	GORASP2	Golgi reassembly-stacking protein 2	3	20	831.7663	65.217	4.19E-29	2	126.8	118.78	21380000
3	GSSQSVEIPGGGTEGYHVLR	GORASP2	Golgi reassembly-stacking protein 2	4	20	624.0766	65.433	6.40E-07	2	52.814	48.057	4730500
3	GSSQSVEIPGGGTEGYHVLR	GORASP2	Golgi reassembly-stacking protein 2	3	20	831.7663	65.433	0.001033	2	45.347	41.598	6562900

Reagent 4

Replicate	Sequence	Gene Names	Protein Names	Charge	Length	m/z	Retention time	PEP	MS/MS Count	Score	Delta score	Intensity
1	GAQFSK	MARCKS	Myristoylated alanine-rich C-kinase substrate	3	6	424.2592	53.661	0.013186	3	95.957	43.368	1.98E+08
2	GAQFSK	MARCKS	Myristoylated alanine-rich C-kinase substrate	3	6	424.2592	53.318	0.017324	3	88.627	50.425	4.77E+08
3	GAQFSK	MARCKS	Myristoylated alanine-rich C-kinase substrate	3	6	424.2592	54.199	0.029403	1	74.832	41.14	3.06E+08
1	GAYLSQPNTVK	PPM1G	Protein phosphatase 1G	3	11	604.3561	62.806	0.000401	3	59.198	46.409	8638900
2	GAYLSQPNTVK	PPM1G	Protein phosphatase 1G	4	11	453.5189	62.421	0.001393	2	38.875	32.176	6119700
2	GAYLSQPNTVK	PPM1G	Protein phosphatase 1G	3	11	604.3561	62.47	4.41E-14	2	137.89	104.63	16016000
1	GCTLSAEDK	GNAI1;GNAI3	Guanine nucleotide-binding protein G(i) subunit alpha-1;Guanine nucleo	3	9	538.6275	53.816	0.005217	2	107.57	71.179	22464000
2	GCTLSAEDK	GNAI1;GNAI3	Guanine nucleotide-binding protein G(i) subunit alpha-1;Guanine nucleo	3	9	538.6275	53.574	0.003988	1	109.29	76.398	21673000
3	GCTLSAEDK	GNAI1;GNAI3	Guanine nucleotide-binding protein G(i) subunit alpha-1;Guanine nucleo	3	9	538.6275	54.472	0.00135	2	61.78	30.55	15071000
3	GLTISSLFSR	ARF4	ADP-ribosylation factor 4	3	10	572.0173	86.667	0.000289	2	81.017	67.208	6277500
1	GNAAAAK	PRKACA	cAMP-dependent protein kinase catalytic subunit alpha	3	7	412.5909	42.775	0.014289	4	74.415	44.4	5372700
2	GNAAAAK	PRKACA	cAMP-dependent protein kinase catalytic subunit alpha	3	7	412.5909	42.48	0.005757	5	94.407	54.432	7762300
3	GNAAAAK	PRKACA	cAMP-dependent protein kinase catalytic subunit alpha	3	7	412.5909	43.344	0.015513	2	73.039	44.4	2341700
3	GNIFANLFK	ARF1	ADP-ribosylation factor 1	3	9	553.0031	91.506	0.000839	4	107.57	58.367	8859800
3	GNIFGNLLK	ARF3	ADP-ribosylation factor 3	3	9	537.0031	85.756	0.000271	3	120.15	68.809	13906000
2	GQSQSGGHGPGGGK	PSMC1	26S protease regulatory subunit 4	4	14	461.7526	33.69	1.64E-08	2	77.192	57.051	13187000
2	GSQSSK	MARCKSL1	MARCKS-related protein	3	6	409.5787	38.696	0.011373	2	100.07	38.159	10888000

Reagent 6

Replicate	Sequence	Gene Names	Protein Names	Charge	Length	m/z	Retention time	PEP	MS/MS Count	Score	Delta score	Intensity
1	GAFLDKPK	PPM1A;PPM1B	Protein phosphatase 1A;Protein phosphatase 1B	3	8	465.9501	68.722	0.002959	4	68.214	51.741	7095800
2	GAFLDKPK	PPM1A;PPM1B	Protein phosphatase 1A;Protein phosphatase 1B	3	8	465.9501	68.881	0.002579	4	70.525	49.438	10878000
3	GAFLDKPK	PPM1A;PPM1B	Protein phosphatase 1A;Protein phosphatase 1B	3	8	465.9501	69.024	0.002326	4	73.26	44.111	3400400
1	GAGSSTEQR	AKAP12	A-kinase anchor protein 12	3	9	471.5879	51.115	0.010672	1	98.009	64.006	26232000
2	GAGSSTEQR	AKAP12	A-kinase anchor protein 12	3	9	471.5879	51.08	0.002491	1	86.744	64.613	27576000
3	GAGSSTEQR	AKAP12	A-kinase anchor protein 12	3	9	471.5879	50.955	0.003346	1	66.568	46.873	20428000
1	GAQFSK	MARCKS	Myristoylated alanine-rich C-kinase substrate	3	6	386.5608	62.09	0.036197	1	101.33	46.743	2.02E+08
2	GAQFSK	MARCKS	Myristoylated alanine-rich C-kinase substrate	3	6	386.5608	62.101	0.034845	1	80.555	41.733	2.45E+08
3	GAQFSK	MARCKS	Myristoylated alanine-rich C-kinase substrate	3	6	386.5608	62.127	0.036197	1	101.33	41.438	1.47E+08
3	GAQFSK	MARCKS	Myristoylated alanine-rich C-kinase substrate	2	6	579.3375	62.135	0.042823	1	80.377	34.026	1.96E+08
1	GASSSSALAR	MARC2	MOSC domain-containing protein 2, mitochondrial	3	10	476.2719	58.08	0.008183	1	48.284	37.12	8216300
2	GASSSSALAR	MARC2	MOSC domain-containing protein 2, mitochondrial	3	10	476.2719	58.105	0.007254	1	50.194	35.521	7486400
3	GASSSSALAR	MARC2	MOSC domain-containing protein 2, mitochondrial	3	10	476.2719	58.06	0.008183	1	48.284	36.048	5388900
1	GAYLSQPNTVK	PPM1G	Protein phosphatase 1G	3	11	566.6577	71.485	3.49E-14	3	135.8	122.29	22747000
2	GAYLSQPNTVK	PPM1G	Protein phosphatase 1G	3	11	566.6577	71.562	3.05E-07	4	120.63	103.47	32733000
3	GAYLSQPNTVK	PPM1G	Protein phosphatase 1G	3	11	566.6577	71.584	3.43E-10	3	128.51	112.65	21442000
3	GCCSSASSAAQSSK	SLC44A1	Choline transporter-like protein 1	3	14	636.6365	49.985	0.018988	1	32.152	30.108	6985600
1	GCCYSSENEDSDQDREER	LAMTOR1	Ragulator complex protein LAMTOR1	4	18	689.7935	50.889	0.000663	2	43.37	41.264	21546000
2	GCCYSSENEDSDQDREER	LAMTOR1	Ragulator complex protein LAMTOR1	4	18	689.7935	50.953	0.000352	2	49.537	47.432	28789000
3	GCCYSSENEDSDQDREER	LAMTOR1	Ragulator complex protein LAMTOR1	4	18	689.7935	50.958	4.95E-05	2	58.246	56.737	12452000
1	GCTLSAEDK	GNAI1;GNAI3	Guanine nucleotide-binding protein G(i) subunit alpha-1;Guani	3	9	500.9291	62.331	0.002386	2	80.231	59.531	26695000
1	GCTLSAEDK	GNAI1;GNAI3	Guanine nucleotide-binding protein G(i) subunit alpha-1;Guani	2	9	750.89	62.327	0.003001	1	85.533	57.359	13719000
2	GCTLSAEDK	GNAI1;GNAI3	Guanine nucleotide-binding protein G(i) subunit alpha-1;Guani	2	9	750.89	62.337	0.003542	1	68.557	41.303	15123000
3	GCTLSAEDK	GNAI1;GNAI3	Guanine nucleotide-binding protein G(i) subunit alpha-1;Guani	3	9	500.9291	62.352	0.012242	2	52.49	35.25	24109000
3	GCTLSAEDK	GNAI1;GNAI3	Guanine nucleotide-binding protein G(i) subunit alpha-1;Guani	2	9	750.89	62.352	0.002912	1	80.688	49.194	10382000
1	GDVLSTHLDDAR	FAM129B	Niban-like protein 1	3	12	606.9951	70.548	2.31E-05	2	88.021	70.355	6644000
2	GDVLSTHLDDAR	FAM129B	Niban-like protein 1	3	12	606.9951	70.607	9.33E-06	3	108.56	76.967	9228100
3	GDVLSTHLDDAR	FAM129B	Niban-like protein 1	3	12	606.9951	70.726	0.000265	2	66.393	48.727	6952600
1	GDVLSTHLDDARR	FAM129B	Niban-like protein 1	4	13	494.5234	63.632	0.000714	1	39.73	35.509	3510900
1	GGAVSAGEDNDELIDNLK	PCMTD2	Protein-L-isoaspartate O-methyltransferase domain-containing	3	18	779.7359	75.089	0.00357	1	45.618	40.978	5825900
1	GGSASSQLDEGK	FAM129A	Protein Niban	3	12	552.6248	56.591	0.002435	1	51.463	38.508	4898500
2	GGSASSQLDEGK	FAM129A	Protein Niban	3	12	552.6248	56.633	0.004493	1	40.767	31.66	4582400
1	GLTISSLFSR	ARF4	ADP-ribosylation factor 4	3	10	534.3189	94.997	0.001171	2	101.38	85.751	30050000
2	GLTISSLFSR	ARF4	ADP-ribosylation factor 4	3	10	534.3189	95.013	0.0007	3	93.096	77.466	30223000
3	GLTISSLFSR	ARF4	ADP-ribosylation factor 4	3	10	534.3189	95.147	0.008183	1	48.284	38.629	3386300
1	GLTVSALFSR	ARF5	ADP-ribosylation factor 5	3	10	524.3154	92.429	0.002085	3	93.096	64.138	37270000
2	GLTVSALFSR	ARF5	ADP-ribosylation factor 5	3	10	524.3154	92.497	0.000734	3	112.36	80.863	31694000
1	GNAAAAK	PRKACA	cAMP-dependent protein kinase catalytic subunit alpha	2	7	561.8351	52.341	0.028041	1	81.297	30.987	31383000
2	GNAAAAK	PRKACA	cAMP-dependent protein kinase catalytic subunit alpha	2	7	561.8351	52.293	0.023087	2	86.866	46.891	23988000
3	GNAAAAK	PRKACA	cAMP-dependent protein kinase catalytic subunit alpha	2	7	561.8351	52.162	0.029498	2	76.847	41.419	22065000
1	GNEASYPLEMCSHFDADEIKR	PPP3R1	Calcineurin subunit B type 1	4	21	748.11	66.158	1.60E-05	3	47.016	44.521	12006000
2	GNEASYPLEMCSHFDADEIKR	PPP3R1	Calcineurin subunit B type 1	4	21	748.11	66.425	8.40E-06	2	50.858	48.106	11329000
1	GNIFANLFK	ARF1	ADP-ribosylation factor 1	2	9	772.4534	100.93	9.89E-05	3	124.92	80.573	15728000
2	GNIFANLFK	ARF1	ADP-ribosylation factor 1	3	9	515.3047	100.83	0.002358	3	87.696	76.532	42088000
2	GNIFANLFK	ARF1	ADP-ribosylation factor 1	2	9	772.4534	100.82	3.18E-05	3	126.07	91.681	17712000

1	GNIFGNLLK	ARF3	ADP-ribosylation factor 3	3	9	499.3047	93.804	0.002312	2	78.192	50.018	38256000
1	GNIFGNLLK	ARF3	ADP-ribosylation factor 3	2	9	748.4534	93.799	0.007697	2	105.52	56.726	16612000
2	GNIFGNLLK	ARF3	ADP-ribosylation factor 3	3	9	499.3047	93.85	0.002197	3	85.38	52.117	43529000
2	GNIFGNLLK	ARF3	ADP-ribosylation factor 3	2	9	748.4534	93.848	0.001343	2	115.71	74.266	18330000
2	GNLFGR	CHMP6	Charged multivesicular body protein 6	3	6	395.2364	79.17	0.036985	1	107.15	67.172	3226800
2	GNSALR	LRRC57	Leucine-rich repeat-containing protein 57	3	6	379.8961	58.838	0.035336	1	80.377	30.068	9203300
1	GQSQSGGHGPGGGK	PSMC1	26S protease regulatory subunit 4	3	14	577.6359	39.527	4.68E-08	3	106.54	92.97	47879000
2	GQSQSGGHGPGGGK	PSMC1	26S protease regulatory subunit 4	3	14	577.6359	39.575	1.68E-12	3	124.47	96.645	92659000
3	GQSQSGGHGPGGGK	PSMC1	26S protease regulatory subunit 4	3	14	577.6359	39.427	7.92E-12	4	119.88	104.49	47617000
3	GQSQSGGHGPGGGK	PSMC1	26S protease regulatory subunit 4	3	14	577.6359	40.36	0.000267	1	64.534	41.522	5179400
1	GSEQSSEAESRPNDLNSSVTPSPAK	LOH12CR1	Loss of heterozygosity 12 chromosomal region 1 protein	4	25	774.3876	50.521	1.95E-07	1	37.373	32.958	7658900
3	GSQHSAAAR	TMEM106C	Transmembrane protein 106C	3	9	468.9284	39.445	0.00341	1	66.27	43.199	7072200
2	GSQSSK	MARCKSL1	MARCKS-related protein	3	6	371.8803	47.661	0.036462	1	103.28	32.217	29845000
1	GSSQSVEIPGGGTEGYHVLR	GORASP2	Golgi reassembly-stacking protein 2	4	20	638.3382	62.71	8.42E-16	3	81.878	76.666	41927000
1	GSSQSVEIPGGGTEGYHVLR	GORASP2	Golgi reassembly-stacking protein 2	3	20	850.7819	62.702	3.16E-05	2	57.41	53.775	18560000
2	GSSQSVEIPGGGTEGYHVLR	GORASP2	Golgi reassembly-stacking protein 2	4	20	638.3382	62.853	1.33E-31	2	125.73	118.22	48132000
2	GSSQSVEIPGGGTEGYHVLR	GORASP2	Golgi reassembly-stacking protein 2	3	20	850.7819	62.832	2.80E-05	2	58.123	55.411	26248000
3	GSSQSVEIPGGGTEGYHVLR	GORASP2	Golgi reassembly-stacking protein 2	4	20	638.3382	62.844	6.02E-16	2	84.249	80.613	25132000
3	GSSQSVEIPGGGTEGYHVLR	GORASP2	Golgi reassembly-stacking protein 2	3	20	850.7819	62.834	1.30E-07	1	69.747	66.632	13989000

Reagent 7

Replicate	Sequence	Gene Names	Protein Names	Charge	Length	m/z	Retention time	PEP	MS/MS Count	Score	Delta score	Intensity
1	GNAAAAKKGSEQESVKEF	PRKACA	cAMP-dependent protein kinase catalytic subunit alpha	4	18	619.587	68.268	3.47E-09	1	62.203	57.148	21577000

Table S3 : Label-free quantification of YnMyr-tagged proteins using capture reagents 2-7 in Hek293 cells

Sheet 1 : Summary of protein IDs per reagent
The table is showing a total number of protein IDs, the corresponding number of MG proteins and their percentage based on the number of requested valid values within a triplicate (1, 2 or 3 respectively). In addition a number of proteins for which a PTM peptide was found is indicated per reagent.

Sheets 2-7 : Total protein IDs per reagent 2-7
Cells were treated for 24 h with 20 µM YnMyr. Cells were lysed, tagged proteins were captured with reagent 2-7, enriched, digested and analyzed by LC-MS/MS. The data were analyzed with MaxQuant (version 1.5.0.25) and Perseus (version 1.5.0.9). The data were filtered (1 valid value in any replicate) and were displayed without any additional processing (total protein IDs). The LFQ intensity and number of "razor + unique peptides" are displayed for each replicate (N = 3). In addition PEP, Intensity, MS/MS count, sequence coverage, and molecular weight are shown for each protein ID. Colunm MG proteins shows proteins with the MG motif and column PTM peptides indicates if such peptides were found for a given ID. Protein grouping feature was enabled in MaxQuant, therefore when unique peptides could not be assigned to one unique protein they were assigned instead to a group of closely related proteins. Proteins are ordered by gene names.

Reagent	# valid values within triplicate	# total proteins	# MG proteins	% MG proteins	# proteins with PTM peptide
**2**	1	1238	273	22	34
	2	888	215	24	
	3	762	191	25	
**3**	1	851	207	24	20
	2	753	193	26	
	3	636	168	26	
**4**	1	714	179	25	9
	2	629	165	26	
	3	558	144	26	
**5**	1	646	167	26	0
	2	550	145	26	
	3	455	118	26	
**6**	1	817	207	25	26
	2	756	194	26	
	3	677	180	27	
**7**	1	263	88	33	1
	2	126	47	37	
	3	111	44	40	

Reagent 2

Gene names	log2 LFQ intensity_1	log2 LFQ intensity_2	log2 LFQ intensity_3	St dev log2 LFQ	MG protein	PTM peptide	Razor + unique peptides_1	Razor + unique peptides_2	Razor + unique peptides_3	PEP	Intensity	MS/MS Count	Sequence coverage [%]	Mol. weight [kDa]	
AARS	21.59131	20.80908	20.72476	0.47782538			3	5	5	3.06E-15	77577000	13	5.9	106.81	
ABCC4	NaN	NaN	19.79168	N/A			2	0	2	0.000148	17376000	1	1.5	144.2	
ABHD17A	23.44215	23.19912	23.1715	0.14892832			7	8	8	1.06E-22	2.79E+08	24	33.5	33.989	
ABHD17B	23.88372	23.57034	23.7494	0.15722138			7	6	7	7.99E-26	3.29E+08	22	34	32.214	
ABL1	19.31743	NaN	NaN	N/A	+		1	2	1	2.95E-17	14852000	3	4.4	122.87	
ABL2	23.08309	22.84443	23.02484	0.12443166	+		11	11	9	1.43E-35	2.96E+08	46	12.6	115.82	
ACAA2	21.224	20.54745	21.09828	0.35984698	+		2	5	3	1.25E-15	53733000	8	14.6	41.924	
ACACA	25.52746	25.21786	25.93724	0.36085071			26	29	44	5.68E-188	2.45E+09	314	31	265.55	
ACAD9	20.17035	20.05815	20.23783	0.09076278			2	2	3	2.10E-08	36352000	6	6.1	68.76	
ACADSB	NaN	19.58838	19.28821	0.21225224	+		2	2	2	0.00024	11471000	2	6.4	36.025	
ACAT1	21.52109	20.56207	21.40878	0.52428534			5	5	5	3.69E-18	87102000	17	21.8	45.199	
ACLY	21.80766	21.88632	21.70168	0.09265625			5	6	8	1.93E-18	76021000	16	8.6	119.77	
ACOT7	NaN	20.97946	21.10406	0.0881055			1	2	3	4.67E-09	56625000	11	14.2	27.041	
ACOT9	23.53148	23.46076	23.44834	0.04484758			10	11	9	4.59E-33	3.51E+08	54	31.3	46.354	
ACP1	NaN	NaN	19.47082	N/A			1	1	1	0.002102	8920600	3	12.9	7.5005	
ACSL3;ACSL4	19.9876	NaN	NaN	N/A			2	1	0	0.000638	8663100	2	3.6	80.419	
ACTA1;ACTC1;ACT	24.96816	24.80445	NaN	0.11576045			1	1	1	7.40E-92	9.6E+08	26	28.9	42.051	
ACTG1;ACTB;ACTA	28.549	28.53634	28.66082	0.068507			18	19	19	0	1.28E+10	601	67.5	41.792	
ACTN4;ACTN1	21.19514	21.27048	21.34017	0.07253334	+		6	7	8	5.49E-17	75173000	11	9.9	104.85	
ACTR3	19.44451	19.78692	NaN	0.24212043			2	2	2	1.77E-05	14608000	2	5.3	47.371	
ADH5	NaN	20.36741	NaN	N/A			1	2	1	4.46E-05	14519000	2	11.8	27.547	
ADO	NaN	NaN	18.73085	N/A			1	1	1	0.005285	12113000	3	3.3	29.751	
ADRM1	NaN	NaN	19.57117	N/A			0	0	1	0.008675	20607000	2	3.9	42.153	
ADSL	20.51307	20.60431	20.74337	0.1159745			3	3	3	3.16E-07	41640000	9	6.4	54.889	
AGPAT1	21.97862	22.12603	21.88308	0.12239438			4	4	4	4.89E-11	1.12E+08	30	35.1	18.756	
AHCY	24.59912	24.70556	24.62634	0.05529649			11	14	14	3.52E-54	9E+08	113	34.3	47.716	
AIFM1	20.06487	NaN	NaN	N/A	+		3	3	1	1.14E-08	14523000	3	7.1	66.294	
AIFM2	24.83735	24.79382	24.92741	0.06813217	+		11	13	11	1.32E-58	7.66E+08	64	44.5	40.526	
AIP	NaN	17.7544	NaN	N/A			0	1	1	4.28E-05	9698800	1	9.5	26.595	
AK2	19.91045	NaN	NaN	N/A			2	1	0	3.70E-05	14523000	3	57.1	4.4602	
AKAP12	27.00325	27.11357	27.07674	0.05616602	+	+	33	34	38	0	4.84E+09	455	37.4	191.48	
AKR1A1	NaN	20.00347	19.84419	0.11262797			1	2	2	6.39E-05	13251000	3	5.5	36.573	
AKR1B1	21.36148	21.30218	21.56658	0.13873828			3	3	3	3.17E-08	93513000	7	11.1	35.853	
ALDH18A1	22.95091	22.73622	22.50325	0.2238922			9	8	8	5.75E-27	2.24E+08	48	14.9	87.088	
ALDH2	19.57919	NaN	20.14631	0.4010144			2	1	3	1.13E-08	20215000	8	7	56.381	
ALDH5A1	21.02911	21.00929	21.00168	0.01416068			5	4	5	4.75E-16	45353000	13	9.9	57.214	
ALDH6A1	18.756	19.22553	NaN	0.33200785			2	2	0	0.001351	4354900	2	3.8	56.227	
ALDH7A1	23.04375	22.7111	22.70875	0.19273754	+		6	7	6	1.96E-21	2.11E+08	19	21.5	55.366	
ALDH9A1	21.2727	21.50489	21.40494	0.11646861			4	4	5	1.39E-15	91824000	15	13.2	53.801	
ALDOA	26.77102	26.58821	26.45657	0.15791738			15	17	17	5.06E-187	3.01E+09	221	68.4	39.42	
ALDOC	22.91504	22.60795	22.50678	0.21260913			1	1	1	5.87E-18	2.18E+08	13	8.2	39.455	
ALG1	19.88369	NaN	19.83536	0.03417447			2	1	2	1.93E-05	20183000	8	5.6	52.518	
ALG6	NaN	21.02715	21.43939	0.2914977			1	2	3	2.23E-07	65478000	7	5.5	58.18	
ANKIB1	19.50279	19.67927	NaN	0.1247902	+		2	2	2	1.76E-05	15999000	3	2.5	122	
ANO6	22.45282	22.07222	22.05298	0.22549891			9	8	9	6.48E-20	1.17E+08	21	11.8	103.96	
ANP32A	22.19223	22.27449	22.11375	0.08037741			5	4	5	3.14E-20	1.67E+08	16	29.4	19.997	

ANP32B	21.35901	21.51783	21.29401	0.11514063			2	2	2	5.50E-16	85008000	10	17.9	22.276	
ANXA1	23.96543	23.84479	23.89208	0.0607873			11	12	14	5.45E-105	6.1E+08	89	54.3	38.714	
ANXA11	21.84341	21.46683	21.28048	0.28677198			4	4	3	2.02E-11	1.09E+08	26	8.5	51.242	
ANXA2;ANXA2P2	25.89013	26.05234	25.92443	0.08548834	+		16	18	19	6.34E-165	2.65E+09	241	64.3	38.604	
ANXA4	NaN	19.50411	19.96691	0.32724902			0	2	2	2.23E-05	18401000	2	8.4	27.062	
ANXA5	27.05321	26.548	26.73828	0.25515503			19	19	21	4.45E-256	6.82E+09	647	76.6	35.936	
ANXA6	23.98763	23.4763	23.59946	0.26686535			13	16	13	2.41E-58	6.57E+08	106	42	75.276	
ANXA7	21.08764	21.5326	20.68046	0.42620956			3	4	4	8.89E-12	79585000	13	18.5	37.805	
AP1AR	21.37077	NaN	21.77652	0.28690858	+		2	2	2	1.45E-07	93730000	6	13.6	34.28	
AP1B1	21.6793	21.40925	21.29064	0.19918665			6	6	6	2.23E-21	1.48E+08	32	10.9	101.35	
AP2B1	NaN	NaN	18.97118	N/A			1	1	1	5.76E-18	25786000	2	8.9	98.117	
APEX1	NaN	20.78197	NaN	N/A			1	2	1	0.000179	23148000	6	7.9	27.125	
APH1A	NaN	19.32575	NaN	N/A	+		1	1	0	0.004346	4688200	2	5.7	26.839	
API5	22.91647	22.67373	22.5696	0.1779909			2	4	5	4.29E-71	2.21E+08	36	21.8	50.309	
APMAP	23.04253	23.77666	23.00362	0.43551726			5	5	7	1.73E-21	2.41E+08	24	18.3	46.48	
APRT	23.50191	23.54062	23.4902	0.02638739			4	4	4	6.05E-23	3.19E+08	45	49.4	19.608	
AQP6	NaN	NaN	18.55817	N/A			1	1	1	0.006664	20670000	7	3.2	29.37	
ARCN1	20.47951	20.45262	20.61297	0.08587468			3	2	4	6.23E-10	44299000	10	8	57.21	
ARF1	31.23078	31.25147	31.08879	0.08855697	+	+	13	14	13	0	6.23E+10	761	79.6	20.697	
ARF3	21.09146	21.95908	22.52184	0.72058428	+	+	1	1	1	0	1.97E+08	22	64.6	20.601	
ARF4	28.25917	28.16825	28.01733	0.12215419	+	+	7	7	7	0	7.01E+09	255	86.7	20.511	
ARF5	26.66506	26.83026	26.59534	0.12065055	+	+	6	7	7	0	2.77E+09	142	71.1	20.529	
ARF6	26.37476	26.34216	26.38611	0.02281514	+		7	7	7	2.11E-78	2.14E+09	117	60	20.082	
ARHGDIA	NaN	22.65814	21.85996	0.56439849			2	2	2	3.77E-12	92974000	10	23.3	21.517	
ARL1	27.61786	27.6208	27.36353	0.14769351	+		10	10	10	0	4.38E+09	238	80.5	18.565	
ARL13B	20.26064	20.47694	20.88963	0.31956406			2	3	3	2.82E-06	42032000	11	8.3	37.086	
ARL15	NaN	21.43934	21.25043	0.13357954			1	2	2	1.18E-07	33656000	6	10.3	22.876	
ARL4C;ARL7	21.94102	22.44405	22.34703	0.26686332	+		3	3	2	1.02E-07	1.17E+08	8	16.1	21.487	
ARL5A	NaN	20.14233	NaN	N/A	+		1	3	2	1.15E-21	21895000	8	25.7	20.728	
ARL5B	23.1616	23.38192	23.42073	0.139759	+		3	4	4	2.83E-21	2.38E+08	23	21.2	20.374	
ARL6IP6	NaN	NaN	20.36922	N/A			1	1	1	0.000287	26724000	3	5.3	24.676	
ARMC4	NaN	19.57897	NaN	N/A	+		1	2	0	0.001215	4771200	2	4.6	67.624	
ARPC3	NaN	NaN	18.58545	N/A			0	1	1	0.003624	12205000	5	13.1	9.7261	
ARPC5	NaN	NaN	18.89353	N/A			1	1	1	0.000823	33174000	8	7.9	16.32	
ATAD3B;ATAD3A;A	NaN	21.05742	NaN	N/A			1	2	2	8.46E-09	46550000	10	8.1	67.608	
ATIC	22.26708	22.24899	21.92897	0.19020096			7	7	7	1.03E-21	94879000	16	14	64.523	
ATP11B	21.94732	22.09003	22.07676	0.07884262			6	7	8	4.17E-22	1.1E+08	16	8.1	134.19	
ATP13A3	21.18077	20.76531	20.44758	0.36767897			3	3	2	5.64E-07	33471000	4	2.8	138.04	
ATP1A1	24.70551	24.83735	24.65168	0.09552731	+		19	19	18	3.17E-92	8.85E+08	116	24.8	113	
ATP2A2	23.82813	23.71219	23.83374	0.06861482	+		13	13	14	1.20E-60	4.56E+08	66	21	114.76	
ATP2B1;ATP2B2;A	NaN	19.32661	NaN	N/A	+		0	2	1	0.000224	9407100	3	2.3	106.86	
ATP2C1	NaN	NaN	18.73496	N/A			1	1	1	0.003643	6392500	2	5.1	23.902	
ATP5A1	23.62098	23.735	23.77163	0.07856817			15	14	14	7.15E-77	7.08E+08	92	40.7	59.75	
ATP5B	21.57233	21.79428	22.15653	0.29489447			6	7	7	1.30E-18	1.68E+08	15	24.6	38.138	
ATP5C1	NaN	NaN	20.28568	N/A			1	1	1	0.001031	32460000	3	4	27.512	
ATP5F1	NaN	19.59432	NaN	N/A			0	2	1	4.63E-06	14010000	2	10.3	22.275	
ATP5H	22.01471	21.40572	21.45091	0.33930846			4	5	4	2.04E-20	2.06E+08	18	74.5	15.773	
ATP5J2;PTCD1;ATI	NaN	NaN	19.71783	N/A			1	1	1	0.000567	23534000	6	26.5	5.7407	
ATP5O	22.99514	22.8592	22.87527	0.07428183			5	4	6	2.48E-24	3.62E+08	48	34.7	23.277	
ATP6V0A1	NaN	NaN	19.06113	N/A	+		1	1	1	0.003733	9731800	1	5.1	22.45	

ATP6V0A2	21.17133	21.37124	21.30101	0.10141759	+		4	6	7	3.84E-20	80622000	8	9.2	98.081	
ATP6V0D1	NaN	NaN	19.74077	N/A			2	1	2	1.42E-06	19293000	4	14.7	26.705	
ATP6V1A	20.75768	20.89141	20.64157	0.12502351			3	3	4	1.00E-20	61686000	12	8.9	64.735	
ATP6V1B2	19.88208	20.02756	NaN	0.10286989			2	2	1	2.47E-06	19877000	5	5.1	56.5	
ATP9A	23.70535	23.29095	23.65009	0.22500465			10	10	11	8.96E-41	3.19E+08	59	12.7	118.58	
ATPAF1	NaN	NaN	23.12959	N/A			0	1	1	0.004208	1.48E+08	4	28.9	5.1488	
ATXN10	NaN	NaN	19.87786	N/A			2	2	3	5.06E-09	37702000	7	13.6	46.286	
AUP1	21.89412	21.92665	21.08997	0.473946			3	3	3	4.57E-10	1.02E+08	8	9.4	41.385	
B3GNT1	23.33279	23.46114	23.04795	0.21147637			7	8	8	8.47E-30	2.39E+08	42	33.3	47.119	
B4GALT1	NaN	20.81527	NaN	N/A			0	2	2	1.14E-05	34452000	10	4.9	42.537	
B4GALT7	NaN	NaN	20.19641	N/A			2	1	3	3.28E-07	23726000	8	11.3	37.405	
BAG5	23.17633	23.10362	22.94696	0.11721753			7	9	11	3.83E-39	2.43E+08	31	33.3	51.199	
BASP1	26.27691	26.66099	26.28502	0.219445	+		8	8	7	2.37E-200	1.93E+09	95	57.3	22.693	
BAX	22.16472	21.91703	22.11981	0.13196399			5	5	5	2.50E-44	1.69E+08	36	36	18.129	
BCAM	22.87721	22.89362	22.29205	0.34267769			9	9	10	1.08E-32	1.9E+08	33	21	67.404	
BCAP31	22.30377	22.79392	22.95234	0.33812891	+		5	5	5	5.54E-18	2.15E+08	35	19.5	27.991	
BCL2L12	NaN	20.30006	20.3073	0.00511945	+		1	3	3	7.13E-12	37182000	7	18.6	36.821	
BET1;DKFZp781C0	NaN	20.55178	18.97606	1.1142023			1	2	2	2.61E-16	36720000	9	24.6	13.289	
BET1L	21.77024	21.9712	21.46079	0.25711949			2	3	2	6.35E-10	92362000	11	48.3	6.4651	
BID	NaN	NaN	17.728	N/A			0	1	1	0.000339	2493900	2	15.2	11.263	
BLVRA	NaN	NaN	19.82977	N/A			1	1	2	9.32E-05	12351000	2	6.1	33.428	
BMPR1B;BMPR1A	19.47161	NaN	NaN	N/A	+		2	1	1	1.01E-05	7478500	2	4	56.93	
BRI3	NaN	NaN	19.95943	N/A			1	1	1	0.00011	17947000	2	16.3	10.863	
BRI3BP	NaN	NaN	20.44334	N/A	+		1	1	2	0.000282	24652000	2	9.6	27.835	
BST2	21.09423	NaN	NaN	N/A			2	1	1	2.32E-05	83188000	8	13.7	18.394	
BYSL	NaN	NaN	19.22821	N/A			1	1	2	9.22E-05	15366000	2	3.9	49.601	
BZW1	NaN	20.61369	20.78596	0.12181329			2	3	3	4.91E-10	61522000	18	10.2	40.538	
BZW2	20.40956	20.70438	20.51393	0.14948976			3	3	3	9.60E-08	37898000	9	9.3	39.445	
C11orf48	NaN	22.94535	NaN	N/A	+		1	2	1	6.25E-11	2.61E+08	20	21.2	11.6	
C12orf23	23.33129	22.72542	23.44291	0.38607628			3	5	5	2.14E-28	4.19E+08	61	62.9	11.748	
C1orf21	NaN	NaN	18.83599	N/A	+		0	1	1	0.000642	4379600	2	11.5	10.192	
C1QBP	22.0762	21.93308	22.2009	0.13401553			4	4	4	2.97E-23	1.14E+08	13	26.2	31.362	
C22orf28	NaN	19.90563	NaN	N/A			1	1	2	2.64E-05	10620000	3	4	55.21	
C2orf72	NaN	NaN	17.20103	N/A			1	1	1	0.005316	1478500	2	4.4	30.48	
C8orf47	21.72151	22.49648	22.19316	0.39052	+	+	6	6	5	7.76E-16	94151000	20	21.7	39.935	
C9orf123	24.02977	24.31617	24.35763	0.17852921	+		3	3	2	3.61E-57	6.18E+08	36	50.9	11.829	
C9orf91	20.12994	20.59487	NaN	0.32875516			2	2	2	9.94E-06	16716000	5	6.9	35.653	
CACFD1	NaN	NaN	20.53041	N/A			1	1	1	0.002166	28229000	4	6.9	13.63	
CACNA2D1	NaN	20.2111	NaN	N/A			1	2	2	1.04E-05	20230000	2	2.9	121.89	
CACYBP	21.49653	21.29927	21.19117	0.15483424			3	2	3	9.82E-07	41900000	6	14.6	21.228	
CADM4	NaN	NaN	18.8006	N/A	+		1	1	1	0.002488	4251900	1	4.1	42.785	
CALHM2	21.09275	NaN	NaN	N/A			2	2	2	6.12E-10	51699000	6	11.1	36.174	
CALR	NaN	21.90969	21.76753	0.1005223			1	3	4	6.29E-16	81862000	10	12.9	48.141	
CAND1	24.76865	24.67007	24.85738	0.09369815			24	23	26	2.82E-106	8.89E+08	132	31.7	136.37	
CANX	29.09999	29.15988	29.20818	0.05419837			22	24	23	0	1.84E+10	767	44.9	67.567	
CAP1	21.65705	21.2735	21.21353	0.24063007			4	4	4	9.88E-15	1.11E+08	18	13.7	51.83	
CAPN1	NaN	20.21881	19.7477	0.33312508			1	2	2	7.81E-05	20912000	5	2.5	81.889	
CAPN2	19.96155	19.76861	NaN	0.13642918			3	3	2	7.12E-07	15762000	4	4	79.994	
CAPNS1;CAPNS2	21.11883	21.26385	21.42537	0.15334399	+		3	3	3	2.54E-10	78085000	15	37	11.399	
CAPRIN1	NaN	NaN	20.1673	N/A			2	1	2	4.72E-07	40313000	5	12.9	19.381	

CAPZA2;CAPZA1	NaN	NaN	19.89293	N/A			1	1	1	1.50E-05	18042000	4	6.8	16.698	
CAPZB	22.4303	22.42306	22.4851	0.03392251			4	4	5	2.22E-13	1.62E+08	25	25.4	29.295	
CARS	NaN	19.112	NaN	N/A			0	1	0	0.00187	8354500	2	1.5	82.845	
CAV1	24.18518	24.36866	24.87192	0.35556232			5	5	5	1.42E-67	5.71E+08	75	47.2	20.471	
CAV2	NaN	20.31108	NaN	N/A	+		1	2	1	8.33E-08	55929000	11	18.8	16.828	
CBR1	20.46732	20.61252	20.20574	0.20614603			3	3	1	1.04E-09	40273000	8	23.1	18.762	
CCDC141	NaN	NaN	21.72405	N/A			1	1	1	0.005059	1.06E+08	8	1.7	100.58	
CCNB1	NaN	NaN	23.78497	N/A			1	1	1	0.007642	5.57E+08	8	2.7	44.932	
CCNY	23.9304	24.31997	24.18722	0.19805044	+		11	10	10	1.95E-44	5.2E+08	52	47.5	39.336	
CCNYL1	21.14096	21.52612	21.01883	0.26476628	+		5	4	3	2.56E-33	98915000	19	36.2	40.705	
CCRN4L	20.31363	20.16803	21.02749	0.4599759			2	2	2	1.01E-06	32196000	4	6	48.195	
CCT2	24.22406	24.33865	24.07602	0.13166955			15	15	16	4.36E-88	6.61E+08	84	42.1	57.488	
CCT3	24.26098	24.14569	24.01631	0.1224026			13	14	13	7.39E-80	6.01E+08	89	40	55.674	
CCT4	24.58251	24.47519	24.45375	0.0689884			18	21	20	2.70E-75	7.68E+08	105	55.8	57.924	
CCT5	23.16628	22.99063	23.00235	0.0982033			7	8	10	5.33E-38	2.72E+08	48	22.6	59.67	
CCT6A	23.9679	23.76739	23.6584	0.15698903			10	13	14	5.77E-76	4.41E+08	63	29.6	58.024	
CCT7	23.99248	23.85842	23.81802	0.09132409			11	11	9	4.56E-49	4.72E+08	57	33.1	54.804	
CCT8	25.37723	25.24967	24.93629	0.22690184			19	19	22	6.05E-87	1.16E+09	182	46.5	59.62	
CD151	26.83425	26.96146	26.61105	0.17738273	+		4	4	5	2.39E-50	3.56E+09	181	15.1	28.067	
CD276	26.002	25.71482	25.90502	0.14608988			9	10	8	2.83E-258	1.83E+09	130	37	57.165	
CD44	24.43288	24.78616	24.66145	0.1791664			6	6	5	4.97E-34	7.63E+08	94	33.5	22.683	
CD46	21.25517	21.2401	20.76814	0.27693909			2	2	2	4.53E-06	29560000	3	7.1	36.826	
CD47	NaN	20.78341	20.9959	0.15025312			1	2	3	7.11E-07	55427000	10	9.6	31.742	
CD55	19.64359	20.02797	NaN	0.2717977			2	2	1	0.000402	19435000	4	5.2	35.695	
CD59	NaN	22.61257	NaN	N/A	+		1	2	1	2.62E-06	72878000	5	18.5	11.985	
CD63	27.60562	27.52992	27.56231	0.03798104			7	7	7	4.99E-212	5.65E+09	157	33.5	23.43	
CD81	28.77269	28.52062	28.67084	0.12680612	+		4	5	4	0	1.21E+10	235	50.3	17.963	
CD82	21.14308	NaN	NaN	N/A	+		2	1	1	1.83E-08	19035000	2	13.2	26.818	
CD9	26.392	26.38181	26.15321	0.13502004			6	6	5	4.90E-220	2.91E+09	196	42.8	17.764	
CD97	NaN	NaN	18.06197	N/A	+		1	1	1	0.002139	8942000	1	2.3	81.742	
CDC37	22.20726	22.07741	22.02124	0.09541098			4	4	4	5.76E-15	1.53E+08	26	15.3	44.468	
CDC42	NaN	20.80073	20.74255	0.04113947			1	3	3	6.27E-09	67238000	14	25.7	21.258	
CDCA3	24.90637	24.84833	24.79714	0.05465079	+		9	11	9	3.67E-87	9.61E+08	98	52.2	28.998	
CDK1	23.65556	23.63123	23.51232	0.07664772	+		3	3	5	2.39E-13	3.91E+08	33	26.5	21.738	
CDK4	NaN	NaN	20.27878	N/A			2	1	2	3.63E-11	24100000	5	19.4	18.685	
CECR5	21.47001	21.48925	21.38785	0.05385537			2	5	5	7.30E-13	82416000	13	13.5	43.588	
CERS2	21.51562	NaN	22.50031	0.69628098			4	2	3	2.17E-11	77216000	9	13.8	36.373	
CFL1	25.10641	24.71968	24.98303	0.19754153			7	8	6	1.87E-62	8.73E+08	67	69.1	16.811	
CHCHD3	27.52148	27.69803	27.57658	0.09032904	+		11	12	12	1.22E-222	5.78E+09	281	46.7	26.152	
CHCHD6	24.3181	24.46301	24.44177	0.07825633	+		7	6	7	3.85E-134	8E+08	72	44.7	26.457	
CHIC2	NaN	21.47427	21.60946	0.09559377			1	3	3	1.32E-07	93967000	9	26.3	15.99	
CHMP6	25.76035	25.56424	25.74784	0.10979116	+		6	8	8	5.52E-146	1.43E+09	94	48.3	23.485	
CHP1	25.50109	25.54863	25.67718	0.09109779	+		11	11	9	5.54E-76	1.33E+09	131	67.2	22.456	
CHTOP	NaN	NaN	18.26054	N/A			1	1	1	0.002452	15065000	3	6.4	21.918	
CIB1	NaN	20.8729	20.63662	0.16707519	+		2	3	3	3.23E-07	22664000	4	20.4	21.703	
CIP29;SARNP	NaN	NaN	18.54958	N/A			0	1	1	0.004223	14381000	3	6.7	16.83	
CKAP4	27.74983	27.94943	27.8079	0.10266696			28	31	30	0	5.99E+09	480	57.1	66.022	
CKAP5	20.21395	20.20383	20.24551	0.02173964	+		3	4	4	6.97E-11	43521000	11	2.9	218.52	
CKB	24.35629	24.26999	24.25508	0.05464043			8	11	11	7.38E-86	6.36E+08	80	52	42.644	
CLCC1	20.82446	20.88256	21.19802	0.20101314			3	4	3	1.32E-15	62104000	12	13.2	56.266	

CLDN12	NaN	NaN	20.93891	N/A	+			1	1	1	8.97E-14	68100000	11	6.1	27.11	
CLDND1	22.50511	22.99988	22.48643	0.29119787	+			3	4	4	1.34E-11	1.19E+08	14	33.3	16.049	
CLIC1	24.12668	23.86882	23.88623	0.14411286				7	7	8	4.85E-41	5.76E+08	69	53.1	26.922	
CLIC4	NaN	20.97218	21.02201	0.03523513				1	3	3	1.48E-07	42129000	7	16.6	28.772	
CLN3	22.70369	22.19691	22.33219	0.26240519	+			4	3	4	2.72E-24	1.31E+08	29	21.5	34.568	
CLPTM1	NaN	NaN	19.43659	N/A				0	1	2	3.67E-05	16267000	3	3.4	75.177	
CLTA	20.65237	21.05384	NaN	0.28388216				2	2	1	5.26E-06	74539000	19	7.8	23.662	
CLTC	23.58978	23.55028	23.6841	0.06875598				15	15	18	1.29E-71	4.23E+08	98	15.8	187.89	
CMTM3	NaN	NaN	20.50174	N/A				1	1	1	0.005842	29242000	3	15.3	6.2201	
CNDP2	NaN	19.34655	NaN	N/A				2	2	0	0.000475	13386000	2	3.8	52.878	
CNN3	20.92854	NaN	NaN	N/A				3	1	1	3.75E-11	72237000	18	16.3	31.38	
COPA	20.58985	20.46473	20.54576	0.06346233				3	4	5	3.31E-11	37450000	5	4.4	138.34	
COPB1	NaN	21.41452	21.19802	0.15308862				1	4	4	3.85E-10	75607000	16	4.9	107.14	
COPB2	21.15134	21.11877	21.18186	0.03155055				5	3	2	4.85E-14	61662000	10	6.8	99.045	
COPG1	20.63892	21.09989	21.24528	0.31657122				2	2	3	8.03E-09	72398000	12	4.9	97.717	
COPS3	20.60069	20.77997	20.80444	0.11124609				2	2	2	9.56E-08	55200000	9	9	39.036	
COPS4	NaN	NaN	20.00347	N/A				1	1	2	0.000301	17903000	2	5.4	40.196	
COPS5	NaN	NaN	17.60581	N/A				1	1	1	0.007007	3140800	1	3	37.578	
COPS8	NaN	20.07848	NaN	N/A				2	2	1	2.47E-05	20715000	3	42.2	8.9341	
COPZ1	20.13696	20.72176	20.556	0.30140281	+			2	2	2	2.72E-06	25490000	5	22.8	10.628	
COTL1	20.00415	20.24737	19.79265	0.22754432				2	2	2	0.000375	34272000	4	12	15.945	
COX5A	NaN	NaN	17.40408	N/A				1	2	1	0.000305	16326000	5	23.2	7.771	
COX7C	NaN	NaN	20.33723	N/A				1	1	1	0.004532	39734000	1	16.1	6.3824	
CPD	24.70261	24.65097	24.77173	0.06059049				22	25	24	7.36E-130	9.89E+08	146	25.1	152.93	
CPNE1	19.71734	19.78612	19.25091	0.29118647				2	3	3	6.58E-07	20100000	6	6.2	58.634	
CPNE3	22.13527	21.86355	22.30877	0.22440843				5	4	5	9.04E-14	1.27E+08	22	11.9	60.13	
CPSF6	NaN	NaN	18.66605	N/A				1	0	1	1.72E-08	18861000	3	5.2	52.325	
CRBN	NaN	NaN	18.83692	N/A				1	1	1	0.004879	6985800	1	2	50.474	
CS	24.47098	24.56715	24.59275	0.06420275				7	9	10	3.26E-35	6.56E+08	63	24.5	50.431	
CSE1L	25.28468	25.48542	25.32182	0.10680269				23	21	25	5.92E-151	1.44E+09	186	35.7	107.78	
CSNK1G3;CSNK1G	21.97449	21.82353	22.10246	0.13962282				6	4	5	3.80E-13	57284000	12	13.7	48.897	
CTBP1;CTBP2	NaN	20.81831	20.68509	0.09420077	+			1	3	3	1.52E-07	34808000	7	15.8	18.623	
CTNND1	20.02161	NaN	NaN	N/A				3	2	2	7.18E-06	17401000	3	4.2	92.674	
CTPS1	21.31606	21.17913	21.62511	0.22845856				4	4	3	1.81E-13	54596000	9	9.8	66.69	
CUL1	NaN	20.15165	NaN	N/A				2	2	1	0.000422	26865000	2	2.3	89.677	
CXADR	24.31935	24.61032	24.36279	0.15696161				9	12	12	6.57E-93	7.02E+08	58	40.5	40.029	
CYB561D2	NaN	NaN	18.63687	N/A				1	1	1	0.00011	14038000	2	24	5.212	
CYB5B	22.73896	22.87573	22.52073	0.17905091				2	3	3	1.18E-20	1.48E+08	18	46.4	15.716	
CYB5D2	19.22823	20.62266	20.89711	0.89488531				4	4	3	1.90E-12	31122000	10	24.2	28.689	
CYB5R3	28.66892	28.81467	28.76642	0.07424888	+			17	18	19	0	1.22E+10	574	85.7	31.76	
CYP51A1	NaN	NaN	17.71505	N/A				1	1	1	0.006126	7783500	2	2.7	46.312	
CYSTM1	NaN	23.67971	23.22038	0.32479536				1	2	2	5.15E-08	4.14E+08	33	11.3	10.631	
DAD1	22.42788	22.55766	22.45749	0.0680118				3	2	3	4.48E-11	1.64E+08	21	35.3	9.5541	
DAG1	21.2986	21.31981	NaN	0.01499773				2	2	2	6.91E-07	35846000	5	3.6	97.44	
DAGLB	25.25883	25.16504	25.05076	0.10420301				15	15	15	1.15E-81	1.08E+09	139	24.7	73.731	
DARS2	NaN	NaN	17.90654	N/A				1	1	1	0.00218	8106400	6	1.4	73.562	
DBNL	NaN	NaN	17.43347	N/A				1	1	1	0.003643	3473300	1	3.4	37.1	
DCAF11	21.5582	21.36164	21.95035	0.29972124	+			3	4	5	2.45E-13	86374000	12	12.3	58.846	
DCUN1D3	21.07646	21.13464	21.22764	0.07625539	+			3	3	3	3.21E-09	47330000	8	13.8	34.291	
DDB1	21.14233	21.04534	21.32997	0.14470088				5	5	6	2.28E-15	76194000	19	5.6	121.71	

DDOST	20.48463	20.45814	20.42849	0.02808482	+		2	3	3	7.93E-08	18010000	3	6.8	50.8	
DDT;DDTL	20.44233	20.27696	20.79962	0.26713818			2	2	2	2.18E-11	27019000	3	20.3	12.712	
DDX1	NaN	NaN	19.09728	N/A			0	1	1	0.000103	10095000	2	2.7	82.431	
DDX17	21.5242	21.81538	21.47378	0.18439926			3	4	6	2.65E-21	99665000	25	14	72.371	
DDX39B;DDX39A	24.06628	24.02361	24.23191	0.11003251	+		8	11	11	6.14E-49	5.33E+08	82	29.9	48.991	
DDX3X;DDX3Y	NaN	NaN	19.55675	N/A	+		0	0	2	3.84E-08	18016000	3	5.3	71.354	
DDX46	23.55929	23.18203	23.37119	0.18863025	+		14	12	12	2.70E-41	4.62E+08	87	17.4	117.36	
DDX6	NaN	19.49172	NaN	N/A			1	2	1	0.000248	13843000	3	4.6	54.416	
DECR1	NaN	NaN	18.20065	N/A			0	0	1	0.007291	9593800	1	8.6	15.898	
DEGS1	24.62962	24.6179	24.67374	0.02944497	+		4	4	4	2.35E-38	6.33E+08	55	15.8	37.866	
DEK	NaN	20.06763	20.22341	0.11015309			1	2	2	3.78E-05	26058000	5	6.5	38.704	
DERL1	20.55478	20.20789	NaN	0.24528827			2	2	1	2.45E-05	16495000	4	16.6	17.033	
DESI2	21.39509	21.5134	21.44096	0.05965018	+		2	2	2	1.02E-06	50280000	6	12.4	21.444	
DHCR7	22.24028	22.4234	22.31587	0.09202308	+		4	4	5	3.17E-16	1.4E+08	33	12	54.489	
DHX15	21.58339	21.30858	21.2108	0.19317728			3	5	5	5.54E-17	87879000	18	8.3	90.932	
DHX9	21.50993	21.66952	21.61041	0.08068374	+		5	6	6	6.18E-22	1.04E+08	21	8.3	140.96	
DIRC2	22.57623	22.38488	NaN	0.13530488	+		2	2	2	6.06E-22	2.22E+08	16	10.5	44.145	
DKFZp781B11202;	NaN	19.98926	NaN	N/A			1	2	2	1.41E-08	16844000	5	9.2	45.771	
DNAJC5	25.98383	25.88066	25.90928	0.05326171			6	8	7	9.45E-289	3.38E+09	275	54.5	22.149	
DNAJC7	20.80664	20.72126	20.72318	0.04874936			3	4	5	7.58E-15	47893000	17	20.6	28.704	
DNAJC9	NaN	18.83192	NaN	N/A	+		0	1	0	4.79E-05	15788000	3	7.7	29.909	
DNM1L	21.03059	21.56705	21.40213	0.2747817			5	3	2	9.62E-12	79167000	13	6.6	78.099	
DPM3	NaN	NaN	19.22409	N/A			1	1	1	0.000963	35631000	6	10.9	10.094	
DRG1	20.72642	20.29244	20.29188	0.25072028			2	4	4	5.29E-07	34944000	4	12.3	40.542	
DSC1	NaN	20.42849	NaN	N/A			0	2	1	7.66E-12	22112000	5	4.5	93.834	
DSG2	20.82205	20.99638	21.09603	0.13867595			5	5	5	7.38E-17	87772000	17	6.6	122.29	
DSG4	NaN	NaN	21.83101	N/A			0	0	2	2.14E-10	23450000	3	1.8	113.82	
DSP	NaN	17.31999	20.77844	2.44549345			1	2	13	1.73E-53	1.89E+08	36	9.3	331.77	
DSTN	19.71015	NaN	NaN	N/A			2	1	1	2.06E-07	32626000	4	16.2	16.62	
DUSP22	NaN	NaN	20.63139	N/A	+		1	1	1	0.00099	24038000	4	20.4	6.178	
DUT	21.07555	19.79766	20.80239	0.6729415			2	4	5	5.14E-13	53319000	14	36	17.748	
DYM	24.12518	23.68977	23.81186	0.22459492	+		7	8	10	3.98E-34	3.99E+08	68	18.1	75.935	
EBAG9;PDAF	NaN	NaN	22.11763	N/A			1	2	2	1.61E-08	1.11E+08	3	17	17.132	
EBF2	NaN	NaN	22.07708	N/A			1	1	1	0.000115	1.39E+08	9	2.6	45.967	
EBP	NaN	22.4059	21.9978	0.28857028			1	2	2	3.35E-07	1.46E+08	21	9.6	26.352	
ECE1	20.86408	21.44268	20.9851	0.30517843			3	3	3	1.73E-07	62583000	12	3.3	85.561	
ECHS1	23.08092	22.94799	22.77014	0.15593012			4	6	6	3.25E-21	2.96E+08	41	24.8	31.387	
ECI1;DCI	21.15066	21.03093	20.94135	0.10501629			3	3	2	8.51E-08	34955000	5	11.6	30.895	
ECM29;KIAA0368	NaN	NaN	19.34564	N/A			1	1	3	7.69E-06	6237400	3	1.8	204.29	
EEF1A1P5;EEF1A1	27.99927	28.03821	28.03107	0.02073059	+		13	13	13	2.69E-171	7.65E+09	406	42.6	50.184	
EEF1B2	23.24676	23.25523	23.20189	0.02866535	+		4	3	4	1.50E-38	2.75E+08	28	24.4	24.763	
EEF1D	20.54161	22.38361	22.44465	1.08153064			2	4	5	1.30E-24	1.67E+08	29	31.8	28.821	
EEF1G	25.73104	25.44477	25.46828	0.15892663			15	19	18	1.22E-122	1.47E+09	158	47.6	50.118	
EEF2	27.80156	27.27861	27.58971	0.26304003			31	35	36	2.09E-242	5.11E+09	440	54.8	95.337	
EEPD1	21.07835	21.3839	21.32509	0.16212138	+	+	5	5	3	2.83E-15	64368000	9	10.5	62.402	
EFCAB14	20.824	20.0713	20.82811	0.43576285			3	4	2	2.43E-13	38747000	8	10.7	55.031	
EFNA5	NaN	21.2149	NaN	N/A			0	1	0	0.005964	27244000	2	4.8	26.297	
EFR3A	23.26728	23.03663	22.79352	0.23690731			12	10	11	1.30E-41	2.06E+08	38	21.3	92.923	
EFR3B	20.93531	21.17505	21.16919	0.13675371			4	4	2	1.34E-11	32854000	6	7.3	82.289	
EIF2B1	NaN	20.28387	20.94557	0.46789256			1	3	2	2.31E-13	68937000	17	17.4	33.712	

EIF2S1	NaN	21.05556	20.8634	0.13587764			2	3	3	2.19E-10	52197000	13	14.9	36.112	
EIF2S2	NaN	21.08525	20.33702	0.52907851			1	2	4	3.07E-09	56630000	11	10.8	38.388	
EIF2S3;EIF2S3L	22.21854	22.03634	21.55871	0.34075966			4	6	4	1.56E-16	1.11E+08	18	14	51.109	
EIF3A	21.28664	21.2635	21.06605	0.12123112			6	5	8	3.50E-24	1.03E+08	19	7.2	166.57	
EIF3C;EIF3CL	22.08158	21.80912	22.00007	0.13984529			6	9	7	1.40E-25	1.44E+08	33	11.2	104.1	
EIF3E	22.61988	22.86529	22.77076	0.12377854			8	9	10	1.05E-25	2.32E+08	46	21.1	52.22	
EIF3F	22.19226	22.24991	22.0436	0.10644806			4	6	6	4.00E-26	1.46E+08	26	25.2	37.563	
EIF3H;EIF3S3	20.88047	20.94149	21.08602	0.10556449			2	3	4	7.43E-10	72084000	13	10.8	39.93	
EIF3I	NaN	20.55356	20.42275	0.09249664			2	4	4	1.05E-08	29892000	4	12.6	36.501	
EIF3K	NaN	NaN	20.08926	N/A			1	1	1	0.001691	16252000	5	8	15.875	
EIF3L	21.76612	21.87159	21.78935	0.05541806			5	5	6	3.23E-18	1.11E+08	24	14.2	66.726	
EIF3M	21.60246	21.95284	21.94614	0.20038587			3	5	5	4.22E-15	1.26E+08	22	15.5	42.502	
EIF4A1	25.42793	25.23539	25.53098	0.15003577			17	17	17	4.71E-110	1.2E+09	132	53.2	46.153	
EIF4A3	20.9455	20.44101	19.46802	0.75101785			3	2	2	1.00E-14	45677000	9	16.3	46.871	
EIF4G1	21.67969	21.8933	21.97533	0.15262217			8	7	4	1.14E-32	1.58E+08	31	11.1	154.8	
EIF4G2	20.1322	NaN	20.0174	0.08117586			2	2	3	2.33E-08	29369000	7	4.7	98.149	
EIF4H	20.68825	20.10891	20.35078	0.29098165			2	2	2	0.000101	30653000	2	9.6	25.2	
EIF5	19.94778	20.30775	NaN	0.25453723			3	2	0	3.15E-07	16578000	6	6.3	49.222	
EIF5A	23.3434	23.48837	22.81031	0.35706363			3	3	4	1.03E-21	2.53E+08	32	54.5	16.832	
ELOVL1	20.43125	20.65045	20.81503	0.19253671			3	3	2	3.45E-09	28100000	7	12.5	32.662	
EMC6	18.58589	NaN	NaN	N/A			1	0	0	0.002867	1565300	1	9.1	12.017	
ENDOD1	20.70734	20.31374	20.72334	0.23200184	+		2	2	3	5.36E-10	40164000	7	11.8	55.016	
ENO1	27.14336	26.91479	26.95748	0.12153056			16	16	16	2.03E-225	3.38E+09	290	52.1	47.168	
ERGIC2	24.1741	24.51431	24.29137	0.17281847			7	8	8	3.60E-37	5.08E+08	71	28.4	42.548	
ERGIC3	23.89208	24.09603	23.77555	0.16221502			7	7	6	2.37E-42	3.93E+08	51	26.6	43.222	
ERMP1	NaN	19.85372	NaN	N/A			1	2	0	0.001564	11510000	1	2	93.159	
ERP29	NaN	NaN	20.4109	N/A			1	1	2	1.71E-05	19081000	2	8.4	28.993	
ESD	21.68761	21.70075	21.37984	0.18160317			3	4	4	8.50E-13	96215000	14	25.9	31.462	
ESYT1	NaN	18.61313	18.55255	0.04283653			0	2	2	0.000114	5764100	4	2.3	122.85	
ETF1	NaN	NaN	21.45031	N/A			2	2	4	1.62E-07	39365000	6	10.4	45.462	
ETFA	NaN	19.84925	20.07848	0.16209009			1	2	2	3.84E-09	61038000	8	12.8	30.22	
ETFB	20.88554	21.0189	20.79027	0.1148426			3	3	3	5.55E-12	50676000	8	18	27.843	
EXTL2	NaN	21.67176	21.42762	0.17263305	+		2	3	3	3.14E-08	61811000	4	12	35.967	
EZR;RDX	NaN	21.12692	21.27918	0.10766408			1	2	2	1.04E-18	31924000	5	11.9	69.412	
FABP4	NaN	NaN	24.01708	N/A			1	1	1	0.00802	1.08E+09	7	6.8	14.719	
FAM120A	NaN	NaN	17.17728	N/A	+		0	1	1	0.000381	4307900	2	1.6	121.89	
FAM129A	23.29656	23.04509	23.27597	0.13962252	+	+	12	11	11	1.16E-44	3.02E+08	50	14.7	103.13	
FAM129B	26.20154	26.10558	26.30208	0.0982589	+	+	19	21	21	1.08E-262	2.22E+09	223	42	84.137	
FAM171A2	NaN	19.25153	19.65274	0.28369831			2	2	2	5.46E-06	11363000	2	2.8	87.434	
FAM219B	NaN	20.30173	20.18604	0.08180518			1	2	2	4.66E-07	49572000	8	28.3	11.907	
FAM3C	NaN	NaN	18.39552	N/A			1	1	1	0.003277	5041400	1	11	11.596	
FAM49A	21.47219	21.56723	21.31241	0.12877337	+		2	2	3	4.29E-66	39262000	6	21.1	37.312	
FAM49B	27.49968	27.48478	27.52492	0.02029075	+		17	18	18	0	4.62E+09	348	73.1	36.748	
FAM69B	23.15397	23.39048	23.40237	0.14010764			7	8	7	3.25E-38	2.56E+08	36	29	48.582	
FAM84B	23.22964	23.60037	23.30633	0.19569603	+		6	6	6	2.50E-34	2.83E+08	42	31.6	34.474	
FAM98B	NaN	NaN	19.91684	N/A			0	2	2	0.000504	16451000	1	7.3	37.19	
FARSB	19.39285	19.57786	19.53848	0.09745733			2	3	2	3.71E-06	14464000	4	4.4	66.115	
FAS	20.79741	NaN	NaN	N/A			3	1	0	4.42E-10	35610000	9	9.9	35.386	
FASN	20.53934	20.98155	20.6109	0.23736468			6	6	5	5.08E-18	54301000	13	4.3	273.42	
FBXL20	20.60603	20.78149	20.70159	0.0878464			2	3	2	5.85E-08	23080000	5	7.4	44.98	

FBXO17;SARS2	22.6828	22.74158	22.87789	0.10007965	+		4	7	7	1.82E-23	1.96E+08	26	34.2	31.479	
FDPS	21.35816	21.53474	22.09294	0.38355125			2	2	3	3.96E-08	92928000	14	10.5	40.532	
FEN1	21.38585	21.3303	21.17426	0.10970005	+		4	3	2	1.82E-17	62812000	20	17.9	42.592	
FGD4	NaN	NaN	16.22827	N/A			0	0	1	0.002243	14477000	1	3.4	54.223	
FHL1	NaN	NaN	19.18981	N/A			1	1	1	0.000249	19631000	5	8.5	16.151	
FKBP1A;FKBP12-E:	20.00072	20.0454	NaN	0.03159353	+		2	2	1	3.02E-07	28265000	11	25	11.951	
FKBP4	23.29095	23.52193	23.62399	0.17062808			9	10	10	4.29E-36	3.95E+08	59	33.6	51.804	
FKRP	20.70936	21.11807	21.1213	0.23690675			3	3	3	1.07E-10	34220000	9	8.5	54.567	
FLNA	22.1916	22.30955	22.11385	0.09853574			10	11	13	1.97E-43	1.77E+08	25	7.9	276.55	
FLOT1	24.38621	24.56197	24.33354	0.11961464	+		13	14	15	5.10E-92	6.56E+08	75	49.6	42.08	
FLOT2	26.23951	26.26841	26.31555	0.03838288	+		19	21	21	3.25E-136	2.15E+09	218	51.2	47.064	
FMNL2	20.54151	20.44071	20.16005	0.19767077	+		3	2	3	3.03E-13	43226000	7	5.7	123.32	
FMNL3	21.4501	21.18731	21.65193	0.23297556	+		5	8	7	1.05E-25	91132000	21	9.8	117.21	
FRS2;FRS3	21.23251	21.05152	20.8842	0.1741997	+		3	3	3	2.65E-11	56786000	10	10.4	57.028	
FSCN1	23.58474	23.65611	23.75878	0.08748783			9	9	9	2.27E-45	4.6E+08	88	25.6	52.262	
FUBP1	22.36512	22.07009	22.59466	0.26296546			8	8	7	7.03E-21	1.5E+08	20	17.2	67.56	
FUS	21.41085	21.19328	20.84644	0.28466159			3	3	2	5.66E-08	52552000	8	7	53.354	
FYN	24.0874	24.42265	24.44158	0.19924624	+	+	13	15	16	1.22E-97	5.6E+08	63	44.5	60.761	
G6PD	20.60132	20.30419	20.38543	0.15356577	+		2	3	3	2.32E-07	39799000	9	6	59.256	
GALNT1	21.88718	22.19592	22.2149	0.1839751	+		5	5	4	4.33E-15	1.15E+08	17	12	57.379	
GALNT2	21.14781	21.25822	21.02032	0.11905214			4	5	4	7.30E-12	58308000	13	10.7	60.793	
GANAB	22.86527	22.85375	22.82275	0.02199114			10	10	10	9.90E-45	2.25E+08	45	18.9	96.215	
GAPDH	26.50684	26.20775	26.24244	0.16358769	+		12	13	13	6.55E-212	2.43E+09	195	63.6	36.053	
GARS	NaN	NaN	20.44071	N/A			2	1	2	0.001009	15220000	2	2.6	83.165	
GART	20.96958	21.12591	21.48409	0.26377153			4	4	4	2.50E-09	46445000	12	9.2	46.033	
GDI1	20.23128	20.49658	20.3309	0.13401374			2	2	2	1.45E-19	28235000	6	19.7	50.582	
GDI2	23.28517	23.20836	23.23338	0.03917478			9	10	11	1.48E-35	3.09E+08	38	34.8	50.663	
GHITM	22.34218	22.61538	22.52972	0.13973017			5	4	5	5.38E-21	2.13E+08	25	16	35.282	
GLCE	NaN	NaN	19.40326	N/A			2	1	1	5.74E-05	14233000	2	4.3	62.886	
GLG1	21.94178	22.40603	22.30597	0.24432711			10	10	8	1.40E-34	1.87E+08	25	11.2	134.55	
GLIPR2	NaN	21.75898	21.53583	0.15779088	+		1	2	2	3.20E-10	66801000	12	20.3	14.213	
GLO1	23.88846	23.77072	23.88241	0.06629978			7	8	8	9.91E-26	4.12E+08	45	50.3	19.043	
GLOD4	NaN	NaN	18.84298	N/A			0	1	1	0.007523	3666400	0	11.3	12.704	
GLUD1;GLUD2	21.99807	21.68183	21.84468	0.15814358			6	6	4	5.46E-18	89335000	17	14	61.397	
GMCL1;GMCL1P1	NaN	NaN	20.23515	N/A	+		1	1	2	9.67E-08	25618000	9	4.5	58.684	
GMPS	21.41885	NaN	20.61378	0.56927046			2	1	2	4.76E-06	46202000	11	3.7	65.928	
GNA11	25.46067	25.22148	25.40788	0.12566065			12	14	14	5.04E-89	1.08E+09	90	50.7	42.123	
GNA13	23.48321	23.76415	23.79907	0.17316381			11	12	12	6.67E-45	3.51E+08	67	34.7	44.049	
GNAI1	24.65627	24.60445	24.60484	0.02980635	+	+	5	7	8	3.76E-210	7.19E+08	72	45.2	40.361	
GNAI2	26.47177	26.57529	26.47695	0.05832949	+	+	12	12	12	0	2.35E+09	176	62	40.45	
GNAI3	27.98495	28.00475	27.81244	0.10577876	+	+	15	16	17	2.06E-231	6.9E+09	372	61	40.532	
GNAQ	22.22588	21.81589	21.81816	0.23605527	+		3	3	4	3.81E-40	90141000	10	37.9	42.142	
GNAS	NaN	NaN	20.46692	N/A	+		1	2	2	1.50E-11	31644000	5	7.7	44.179	
GNAZ	20.74616	20.71213	20.97099	0.14066217	+		4	3	3	1.87E-14	39581000	7	16.3	40.923	
GNB1	22.81673	23.02876	23.00563	0.11631489			3	3	2	6.57E-13	2.23E+08	25	12.9	37.377	
GNB2L1	24.27597	24.32045	24.2832	0.02386876			10	9	11	3.02E-46	5.82E+08	60	40.1	35.076	
GNPDA1	NaN	NaN	24.11482	N/A			1	1	2	2.87E-06	1.39E+08	3	14.6	24.047	
GOLGA7	23.68003	23.62578	23.51015	0.08676844			3	4	4	7.22E-29	3.35E+08	66	40.1	15.824	
GOLIM4	25.2097	25.30306	25.26313	0.04684239	+		10	10	11	9.67E-193	1.01E+09	107	17.7	81.879	
GORASP1	22.60729	22.53098	22.77062	0.12242497	+		3	3	3	3.86E-11	1.68E+08	19	16.3	23.906	

GORASP2	26.61625	26.9283	26.55076	0.20174266	+	+	8	10	10	9.70E-171	2.91E+09	148	36.9	47.145	
GOT1	20.83938	20.96846	20.92143	0.06532696			4	5	5	3.10E-14	1.09E+08	14	15.7	46.247	
GOT2	23.13643	23.28249	23.26628	0.08005968			8	10	10	1.98E-40	3.13E+08	46	29.5	47.517	
GPI	24.60246	24.7292	24.51953	0.10559508			9	9	10	1.84E-50	7.16E+08	79	24.9	63.146	
GPM6A;GPM6B	20.45041	NaN	NaN	N/A	+		2	1	1	0.000349	23460000	2	6.7	29.905	
GPR108	NaN	NaN	19.23603	N/A			1	1	1	0.002104	22136000	6	8.7	11.365	
GPRC5A	24.27149	24.26019	24.53053	0.15292325			3	4	4	2.15E-82	5.38E+08	33	14.8	40.251	
GPRC5C	NaN	21.29788	21.22529	0.05132888	+		2	4	3	1.10E-08	54549000	8	10	48.193	
GPS1	NaN	20.76142	NaN	N/A			2	3	2	9.45E-07	22025000	8	7.2	53.371	
GPX8	20.02945	NaN	20.2207	0.13523417			2	1	2	7.60E-06	14313000	3	10	23.881	
GRPEL1	20.23853	20.81167	20.76693	0.31877309			2	2	2	2.01E-05	49739000	4	9.2	24.279	
GSPT1;GSPT2	21.72071	21.90976	21.73169	0.10612052			6	5	6	9.09E-15	1.21E+08	12	10.8	68.6	
GSR	20.93668	20.88643	21.17512	0.1542296			3	4	2	8.52E-12	45372000	8	9.8	47.267	
GSS	NaN	NaN	19.03212	N/A			0	0	1	0.002715	4582500	1	2.5	40.349	
GSTM2;GSTM5;GS	NaN	NaN	19.07428	N/A			0	0	1	0.00632	2353900	1	6.1	17.369	
GSTO1	23.071	22.86495	23.11304	0.13277337			6	4	6	2.11E-18	3.31E+08	41	30.3	27.566	
GSTP1	26.25802	26.34846	26.09489	0.12850975			10	10	9	1.76E-166	2.4E+09	145	57.6	23.356	
H3F3B;H3F3A;HIST	NaN	NaN	23.17813	N/A			0	0	2	3.33E-12	66703000	6	15.2	10.334	
HADH	NaN	20.13546	NaN	N/A	+		2	2	1	0.000796	20344000	3	12.4	34.293	
HADHA	22.60137	22.76622	22.98082	0.19026779			8	9	11	9.30E-34	2.24E+08	39	19.3	82.999	
HADHB	25.0288	25.27633	24.99804	0.15256835			13	14	15	1.54E-60	8.77E+08	87	40.3	48.879	
HARS;HARS2	21.15487	21.11063	21.36175	0.13405084			3	4	3	6.83E-13	68602000	14	11.1	49.623	
HAT1	NaN	NaN	18.91292	N/A			1	1	1	0.000474	13339000	1	3.9	39.786	
HCCS	25.45381	25.3947	25.42525	0.02956058	+		12	12	12	5.27E-112	1.41E+09	172	60.1	30.601	
HDAC2;HDAC1	NaN	NaN	19.79685	N/A			1	1	1	0.002334	19076000	4	20	7.4336	
HDGFRP2	NaN	NaN	18.18394	N/A			1	1	1	0.003806	8788500	4	8.7	19.097	
HEATR2	NaN	NaN	18.13416	N/A			1	1	1	0.00521	2479600	1	4.7	26.361	
HGSNAT	NaN	21.10284	21.17329	0.04981567	+		1	2	2	6.30E-07	44056000	6	4.1	70.495	
HID1	NaN	18.82811	NaN	N/A	+		1	1	0	0.000303	6641400	1	6.8	21.34	
HINT1	22.01206	22.40166	21.98715	0.23246047			2	3	3	9.21E-08	98861000	13	19.8	13.802	
HIST1H1C;HIST1H1	20.75711	20.65665	22.73459	1.171776			3	2	3	1.99E-08	61928000	6	15	21.364	
HIST1H2AJ;HIST1H	NaN	NaN	24.28383	N/A			0	1	3	3.52E-07	83368000	6	27.3	13.936	
HIST1H2BL;HIST1H	NaN	NaN	23.73552	N/A			1	1	5	4.08E-14	2.71E+08	10	35.7	13.952	
HIST1H4A	18.84855	19.3413	25.15943	3.51000108			2	2	5	1.63E-22	2.42E+08	21	50.5	11.367	
HLA-B;HLA-C	23.13078	23.13097	22.98113	0.08645537			4	7	5	2.78E-39	2.6E+08	51	26.8	40.46	
HLA-C	20.19976	20.38869	20.17731	0.11610344			2	2	2	8.95E-30	30057000	10	26.5	40.648	
HLA-C;HLA-H;HLA-	21.02952	NaN	NaN	N/A			2	1	1	2.43E-31	38105000	3	23.8	40.932	
HM13	23.22456	23.5013	23.24458	0.15432163			4	5	5	1.24E-32	3.84E+08	48	22.7	36.813	
HMGB1;HMGB1P1	22.19003	22.05053	21.42757	0.40597304	+		4	4	5	7.82E-22	1.73E+08	33	30.2	24.893	
HMGB2	NaN	NaN	20.64922	N/A	+		0	2	2	5.90E-15	36018000	11	21.5	24.033	
HMGB3	NaN	NaN	19.80799	N/A			1	1	1	0.0012	28662000	4	8.5	17.522	
HMGCS1	21.28195	21.49575	21.43171	0.10972664			5	5	4	6.74E-16	82919000	10	13.1	57.293	
HMOX2	21.46518	21.43237	21.24887	0.11657527			5	5	6	2.07E-19	97839000	13	34.8	32.837	
HNRNPA1;HNRNP	21.91276	22.21827	22.23637	0.18183665			5	5	5	6.42E-25	1.57E+08	24	38.6	29.386	
HNRNPA2B1	21.29418	22.07705	21.81706	0.39872374			2	3	4	1.64E-11	85576000	13	12.6	36.006	
HNRNPC;HNRNPC	NaN	19.36489	NaN	N/A			1	1	0	0.004087	5058100	1	22.2	5.9078	
HNRNPD	23.10026	22.68968	22.99241	0.21285872			4	6	5	4.76E-23	2.25E+08	25	46.8	12.553	
HNRNPF	22.14495	21.70565	21.70594	0.2535463			4	5	6	3.82E-16	1.2E+08	19	18.1	45.671	
HNRNPH1;HNRNP	20.91145	NaN	NaN	N/A	+		2	1	1	8.87E-12	42544000	6	13.1	47.087	
HNRNPK	23.45588	23.71356	23.72328	0.15165543			10	12	11	6.15E-54	4.39E+08	62	37.4	47.557	

HNRNPM	24.82857	24.8616	24.9406	0.05756547			19	20	21	1.10E-68	8.39E+08	106	38.6	73.62	
HNRNPU	22.7145	22.3879	22.38485	0.1894492	+		3	4	5	2.72E-23	1.62E+08	20	8.8	88.979	
HPCAL1	25.17451	25.11791	25.5338	0.22555759	+		10	10	10	1.42E-66	1.03E+09	133	59.6	22.313	
HPCAL4	NaN	NaN	20.15993	N/A	+		1	1	2	0.000212	20746000	3	11.5	22.202	
HPRT1	22.70168	22.62558	22.48005	0.11261294			7	6	7	8.38E-27	2.11E+08	36	42.7	24.579	
HRAS	21.85533	21.97169	21.9218	0.05837654			2	2	2	5.63E-21	84686000	7	31.7	21.298	
HSD17B10	22.15647	21.94142	22.02371	0.10850758			5	6	7	2.36E-16	1.51E+08	25	36.8	26.923	
HSD17B12	22.45302	22.51658	22.58646	0.06674494			7	8	8	8.83E-29	2.14E+08	30	34	34.324	
HSD17B4	21.52387	21.39367	21.52535	0.07560187	+		4	6	4	1.58E-18	86934000	9	11	79.685	
HSDL2	20.60567	21.12117	21.2058	0.32482264			2	3	3	1.50E-08	51117000	10	9.3	37.32	
HSP90AA1	25.8666	25.93735	25.74532	0.0971167	+		13	14	14	0	2.06E+09	195	45.4	84.659	
HSP90AB1	27.43181	27.51781	27.49121	0.04403014			27	24	25	0	5.69E+09	458	51.8	83.263	
HSP90AB2P	NaN	NaN	20.9789	N/A			1	1	1	3.46E-38	38515000	9	18.6	44.348	
HSP90AB4P	NaN	NaN	18.81121	N/A			1	1	1	2.75E-08	7477000	1	4.4	58.264	
HSP90B1	25.04906	25.09188	24.85866	0.12414862			19	18	17	2.90E-86	1.02E+09	112	33.6	92.468	
HSPA1A	28.29903	28.28268	28.21424	0.04498277			26	26	25	0	1.05E+10	729	61.2	70.051	
HSPA4	23.8044	23.65938	23.66003	0.08354033			17	15	15	7.24E-63	4.74E+08	72	29.6	94.33	
HSPA5	24.36786	24.17843	24.33368	0.10095755			11	11	13	1.34E-82	5.09E+08	56	28.7	72.332	
HSPA6;HSPA7	NaN	NaN	24.51147	N/A			1	1	1	1.93E-107	1.14E+09	33	15.7	71.027	
HSPA8	26.3102	26.13087	26.19173	0.09119423			22	22	23	1.19E-192	2.5E+09	271	48.5	70.897	
HSPA9	24.40665	24.22258	24.0721	0.16755581			11	11	10	1.52E-78	6.28E+08	86	26.8	73.68	
HSPB1	21.36592	20.98336	21.32833	0.21085914			3	3	3	2.27E-10	68430000	9	19.4	20.406	
HSPD1	26.93506	26.85234	26.64719	0.14821058			24	24	22	0	3.46E+09	307	55.7	61.054	
HSPH1	21.67784	21.6532	21.43746	0.13224562			4	8	8	1.87E-20	1.12E+08	17	12.8	92.115	
IARS	19.67168	NaN	NaN	N/A	+		2	1	0	0.00031	4753900	2	1.5	131.76	
IARS2	NaN	NaN	18.69088	N/A			0	1	1	0.003594	6824300	1	1.3	106.04	
IDH2	20.23222	20.43847	20.42921	0.1164974			3	3	3	9.00E-09	40406000	9	10.2	36.171	
IDH3A	21.46109	21.10412	21.30123	0.17880863			3	6	4	3.39E-16	93357000	18	20.2	35.786	
IFITM2;IFITM3;IFIT	25.42803	25.79563	25.61186	0.1838			2	2	3	3.84E-81	1.95E+09	79	29.5	14.632	
IGF2BP1	NaN	18.71756	NaN	N/A			1	2	0	0.000119	4294000	3	4.3	63.48	
IGF2R	21.33946	20.98482	20.65631	0.34165828	+		6	6	7	8.09E-19	54818000	14	3.5	274.37	
IGSF8	19.30721	20.13358	19.45314	0.44105584	+		2	2	2	2.85E-07	27471000	5	14.3	32.284	
IKBIP	NaN	NaN	18.3641	N/A			1	1	1	0.004879	9752200	1	2.6	39.309	
ILF2	21.74032	21.56356	21.75169	0.10548797			6	5	5	6.80E-17	1.21E+08	19	21	43.062	
ILF3	22.13261	21.76109	22.2165	0.24237124			5	6	6	2.65E-22	1.37E+08	36	15.1	74.606	
IMMT	21.66605	22.05109	21.56867	0.25510381			6	8	7	1.41E-28	1.48E+08	24	13.9	78.973	
IMPA1	NaN	NaN	19.45982	N/A	+		2	1	2	0.000112	13075000	6	7.2	30.188	
IMPDH2	22.1403	22.14485	22.2219	0.04585478			3	5	4	6.54E-18	1.48E+08	28	21.6	55.804	
IPO4	NaN	20.53659	NaN	N/A			0	2	1	7.67E-05	18362000	4	6.4	33.154	
IPO5	23.28616	22.93957	23.68571	0.37338312			11	10	13	5.23E-51	3.51E+08	46	21.6	123.63	
IPO7	22.94464	22.8506	23.16432	0.16099848			7	8	9	2.80E-41	2.86E+08	44	13.1	119.52	
IPO9	20.95964	21.19707	20.25292	0.49112887			2	2	2	3.91E-10	36722000	5	3.6	115.96	
IQGAP1	20.76652	20.69659	20.62863	0.06894735			4	3	2	8.57E-10	34003000	9	2.5	189.25	
ISYNA1	NaN	20.68055	20.42737	0.17902529			1	3	4	1.02E-08	42013000	8	8.8	44.786	
ITFG3	22.3684	22.1181	21.8926	0.2380077			3	4	4	5.25E-20	1.15E+08	21	12.1	59.659	
ITM2B	23.03399	23.21667	22.76034	0.22967128			5	5	6	1.31E-46	1.93E+08	18	30.8	30.338	
ITM2C	20.69668	20.46364	NaN	0.16478416	+		2	2	1	3.44E-07	27790000	7	11.1	20.056	
JAM3	23.75674	23.6726	23.41957	0.17549327			5	6	6	1.64E-44	4.59E+08	47	27.1	35.02	
JUP	NaN	21.41669	23.07433	1.17212848			0	2	6	1.55E-38	74558000	13	19.1	81.744	
KCNB1	22.47709	22.12256	NaN	0.25069057			1	1	1	0.003318	84060000	3	1	95.877	

KDELR1	19.78424	NaN	20.16742	0.27094918			2	1	2	2.19E-18	46071000	9	20	17.486	
KDELR2	NaN	NaN	17.27712	N/A			1	1	1	2.61E-12	22939000	3	18.4	24.422	
KHSRP	24.50525	24.27555	24.38851	0.11485518			13	12	13	1.86E-50	6.38E+08	93	26.9	73.114	
KIAA0319L	23.09431	22.46326	23.15058	0.38161918			5	7	7	1.09E-29	1.85E+08	20	7	109.75	
KIAA0754	NaN	NaN	19.62599	N/A			1	1	2	7.69E-05	14523000	3	1.8	104.66	
KIAA1279	NaN	NaN	18.42132	N/A			1	1	1	0.008519	5064800	1	1.4	71.813	
KIAA2013	23.03366	23.41893	22.90923	0.26574107			8	7	7	1.22E-30	2.55E+08	30	15.8	69.156	
KIF11	NaN	NaN	18.27708	N/A			1	1	1	0.000354	22103000	2	1.5	119.16	
KIF16B	NaN	NaN	26.20043	N/A			1	1	1	0.003129	3.82E+09	23	1.8	59.06	
KIF5B	19.77734	19.57764	19.58851	0.11229056			2	2	2	6.15E-06	21044000	4	3.8	109.68	
KLB	NaN	NaN	19.61088	N/A			1	1	1	0.006367	58438000	4	0.9	119.81	
KMT2C	NaN	NaN	21.88643	N/A			1	1	1	0.007656	1.65E+08	13	0.2	443.53	
KPNA2	21.71712	22.09976	21.68594	0.23044614			6	6	6	2.04E-17	1.32E+08	21	18.9	57.861	
KPNA4;KPNA3	20.27492	20.32641	NaN	0.03640893			2	2	1	3.57E-08	31298000	8	6	57.886	
KPNA6;KPNA1;KPI	NaN	NaN	19.47649	N/A			0	0	1	0.006069	8686600	2	3.4	35.457	
KPNB1	25.55608	25.71091	25.6719	0.08052782			19	21	21	3.10E-111	1.66E+09	203	35.7	97.169	
KRT121P	NaN	NaN	19.37715	N/A			0	0	1	7.40E-68	7778900	2	46.7	29.117	
KRT18	20.56802	20.49111	19.95886	0.3317331			2	3	3	7.65E-23	23995000	9	12	43.774	
KRTCAP2	NaN	NaN	19.92264	N/A			1	1	1	0.0001	18963000	5	12.5	14.678	
LAMC2	NaN	NaN	21.60128	N/A			0	0	1	0.00701	69572000	3	0.8	121.6	
LAMTOR1	26.66166	26.56764	26.87701	0.15860075	+	+	10	11	11	2.99E-230	3.45E+09	334	92.5	17.745	
LANCL1	20.76685	20.95837	20.81675	0.09935303			4	2	2	7.40E-10	50854000	8	47.6	9.2824	
LANCL2	20.86899	20.94221	20.88941	0.03778444	+		4	3	3	6.60E-12	47151000	15	11.8	50.854	
LAP3	19.69753	NaN	NaN	N/A			2	1	1	2.94E-09	21608000	5	7.2	52.771	
LARS	19.44024	19.46324	20.27549	0.4757313			2	2	2	0.00032	21814000	4	1.9	129.21	
LBR	21.82924	22.26637	22.18404	0.23228714			3	3	2	4.27E-08	1.08E+08	18	4.2	70.702	
LDHA	26.28313	26.22326	26.97093	0.41546434	+		12	13	13	7.36E-182	3.05E+09	215	61.1	36.688	
LDHB	27.47158	27.72117	27.51058	0.1342661			12	16	15	1.15E-180	6.48E+09	312	71.6	36.638	
LEMD3	NaN	NaN	17.94712	N/A			1	0	1	6.41E-05	4670700	2	1.8	99.996	
LEPROTL1	NaN	NaN	21.16705	N/A			1	1	1	1.31E-07	62321000	9	12.3	11.698	
LGALS3	NaN	NaN	18.80117	N/A			0	0	1	0.007645	1947900	1	4.7	24.086	
LHFPL2	NaN	20.99831	20.986	0.00870448			1	2	2	2.52E-15	35884000	7	14.9	24.486	
LMBRD1	23.99196	23.96913	23.64988	0.19125049			3	3	3	4.29E-43	3.9E+08	25	9.9	44.211	
LMBRD2	NaN	20.30251	19.7949	0.35893447			2	2	2	0.000742	51018000	2	2.4	81.171	
LMF2	21.14501	20.85772	20.90124	0.15484043			5	4	3	6.84E-11	62129000	10	12.1	67.155	
LNP;KIAA1715	25.28641	25.5547	25.26621	0.16104557	+	+	14	13	13	1.25E-77	1.19E+09	137	40.4	47.094	
LNPEP	25.02301	24.99386	24.92597	0.04979213			22	22	22	3.65E-109	1.06E+09	132	27.7	117.35	
LOH12CR1	24.12005	24.16216	24.13101	0.0218468	+		10	10	9	7.39E-50	4.99E+08	48	64.4	20.247	
LONP1	NaN	NaN	18.21594	N/A			1	1	1	0.002709	5346600	1	4.1	27.451	
LPAR1	18.29055	19.57302	NaN	0.90684323			2	2	1	3.65E-05	11576000	3	5.5	41.109	
LPHN2;LPHN1;LPH	NaN	NaN	20.67745	N/A			1	2	2	0.000584	53820000	2	3.6	52.311	
LRPPRC	24.073	24.0481	23.89236	0.09789941			21	21	18	2.26E-75	6.2E+08	91	23.1	157.9	
LRRC1	22.40372	22.49526	22.56165	0.07929805			8	7	5	1.12E-54	1.25E+08	19	34.4	59.241	
LRRC15	NaN	NaN	23.58944	N/A			0	0	3	9.78E-07	61378000	4	4.1	64.365	
LRRC57	25.84793	25.61263	25.80693	0.12569777	+		8	11	11	8.54E-69	1.48E+09	128	59	26.754	
LRRC59	21.16638	21.47615	21.58091	0.21554861			2	3	3	2.25E-09	78528000	23	10.4	34.93	
LSR	25.19238	25.08018	25.13269	0.05613828			10	9	9	5.70E-101	1.06E+09	107	29.7	69.428	
LTA4H	21.57939	21.34635	20.93012	0.32891402			8	5	4	2.96E-18	85985000	12	18	69.284	
LTBR	NaN	15.42052	NaN	N/A	+		0	1	0	0.000865	8823000	3	8.8	15.718	
LUC7L2	21.46244	21.18979	21.14837	0.17063294			2	3	3	1.19E-07	99584000	17	7.9	46.513	

LUC7L3	NaN	21.03288	20.84046	0.13606149				2	3	3	2.26E-13	99675000	14	14.6	42.571	
LYN	24.99611	24.97548	24.9061	0.04715397	+			13	16	16	2.96E-104	8.52E+08	94	43.9	58.573	
LYN	NaN	NaN	20.68791	N/A	+			1	1	1	1.15E-91	31136000	4	45.6	56.033	
LYPLA1	22.22143	22.38113	22.18958	0.10264008				4	4	4	2.27E-11	1.05E+08	12	23.4	20.861	
LYPLA2	22.20932	22.12559	22.08463	0.06355579				4	4	4	5.01E-26	1.13E+08	18	22.5	24.737	
LZTS1	20.7733	20.94799	20.73331	0.11416603	+			3	5	5	5.99E-09	46441000	8	7.7	55.611	
LZTS2	NaN	20.62292	19.40277	0.86277634				1	2	2	7.62E-08	19438000	3	5.8	72.759	
M6PR	23.64548	23.84144	23.69115	0.10252898				3	3	4	3.55E-16	3.16E+08	23	22.4	30.993	
MACROD1	NaN	NaN	19.42086	N/A				1	1	1	0.001963	9376600	1	3.1	35.505	
MAGED2	NaN	NaN	19.3283	N/A				1	1	2	0.001288	7467000	1	3.5	55.795	
MAGT1	19.57269	NaN	NaN	N/A				2	1	2	0.000122	9476700	7	5.1	38.036	
MAL2	NaN	NaN	19.29129	N/A				1	1	1	0.006974	13575000	2	6.2	19.125	
MAN1B1	22.43553	22.33181	22.08314	0.18109543				5	5	5	5.03E-21	1.34E+08	19	18.2	46.027	
MAPK3;MAPK1	NaN	19.536	NaN	N/A				1	1	0	0.005069	24264000	7	3.4	30.64	
MAPRE1	23.7599	23.19522	23.34082	0.29316972				7	9	11	8.68E-38	5.38E+08	64	46.3	29.999	
MARC1	NaN	18.8065	NaN	N/A	+	+		1	1	1	3.73E-13	4822600	3	7.4	37.499	
MARC2	24.50803	24.62812	24.65753	0.07920102	+	+		8	11	11	1.70E-45	5.6E+08	58	32.5	38.023	
MARCKS	29.53755	29.74666	29.74108	0.11915158	+	+		11	11	11	0	3.02E+10	411	48.5	31.554	
MARCKSL1	26.105	26.1187	25.40217	0.40979119	+	+		5	6	4	3.32E-200	1.7E+09	76	60	19.529	
MARS	NaN	20.05457	NaN	N/A				2	3	1	6.44E-08	11200000	4	6	71.827	
MAT2A	20.40156	NaN	NaN	N/A				1	1	1	8.55E-06	18507000	5	7.4	32.964	
MAT2B	NaN	NaN	19.67471	N/A				1	2	2	0.000112	10052000	1	6.9	34.583	
MAVS	NaN	19.48318	NaN	N/A				0	1	0	0.000431	10588000	3	3.3	40.467	
MBLAC2	25.86453	25.48898	25.90369	0.22896713				9	10	10	6.01E-35	1.13E+09	41	50.2	31.371	
MBOAT7	20.10725	20.19868	20.13896	0.04642458	+			2	2	2	3.63E-06	30575000	6	6	44.732	
MBP	NaN	20.24377	NaN	N/A	+			2	2	1	1.68E-06	52373000	4	36.7	6.4902	
MCAM	21.1881	20.9959	21.35509	0.17974239	+			3	3	3	1.17E-22	57399000	11	7.1	71.607	
MCCC1	26.21653	25.94438	26.17062	0.14569253				16	22	23	0	3.94E+09	306	52.8	80.472	
MCM2	19.76111	NaN	NaN	N/A				2	1	1	0.00029	8640500	2	2.4	93.998	
MCM3	20.27799	20.46344	20.74862	0.23706959				4	4	3	1.35E-09	51517000	11	4.3	90.98	
MCM4	20.56151	20.48827	20.3552	0.1045909				3	3	4	2.78E-07	36122000	5	4.4	96.557	
MCM5	NaN	NaN	20.42686	N/A				2	2	2	1.00E-04	21283000	2	3.8	77.592	
MCM6	NaN	NaN	19.47566	N/A				0	1	1	0.001181	10086000	1	1.6	92.888	
MCM7	NaN	19.99521	NaN	N/A				1	2	1	7.60E-07	23541000	5	7.4	60.643	
MCMBP	19.25276	19.53958	19.3323	0.14807505				2	2	2	6.01E-05	10076000	4	3.3	72.748	
MCOLN1	21.4498	NaN	20.70066	0.52972197				2	2	3	2.68E-08	37956000	4	5.3	65.022	
MDFIC	20.11197	NaN	21.4995	0.98113187				2	1	2	4.55E-13	77699000	13	27.6	16.46	
MDH1	24.2553	24.47178	24.03431	0.21873887				8	8	8	1.96E-39	6.28E+08	73	32	36.426	
MDH2	25.00093	24.99045	24.66291	0.19220206				13	14	13	7.75E-70	9.66E+08	139	55.6	35.503	
ME2	20.95759	21.09777	21.1739	0.10972407				4	5	5	1.84E-13	55173000	10	8.7	65.443	
METAP1	NaN	NaN	17.59609	N/A				0	1	1	0.005676	2907200	1	3.3	37.812	
METTL7B	21.78437	21.18211	21.29496	0.32014954				2	3	3	1.21E-08	60108000	13	14.3	27.775	
MFSD12	21.52172	21.94988	21.61486	0.22517935	+			3	3	2	1.23E-07	42656000	7	8.5	51.029	
MFSD5	21.51475	21.84399	21.89393	0.20602203				4	5	5	2.87E-16	88261000	16	15.8	49.764	
MGRN1	24.12289	23.93724	24.06456	0.09493743	+			11	11	11	1.33E-44	3.91E+08	63	35.5	58.304	
MGST3	20.29558	20.12893	20.53526	0.20425588				2	2	3	1.07E-08	24071000	6	18.4	16.516	
MICB;MICA	NaN	21.06171	20.62391	0.30957135	+			1	2	2	0.000288	41059000	7	4.4	37.579	
MIF	24.2995	24.14709	24.2097	0.07660816				2	2	2	5.32E-19	5.92E+08	52	17.4	12.476	
MLEC	24.4311	23.96208	23.96614	0.26962444				5	6	6	2.11E-27	4.31E+08	46	53.4	16.729	
MON2	NaN	NaN	18.63091	N/A				0	0	1	0.006005	2535200	1	9.9	10.176	

MORC4	NaN	23.29558	NaN	N/A		+	0	1	0	0.001988	1.88E+08	1	2.6	74.353	
MPDU1	NaN	NaN	20.81088	N/A			1	1	2	8.06E-06	26162000	5	23.8	10.978	
MRPS27	NaN	NaN	19.21537	N/A			2	2	2	0.000448	10672000	2	6.4	34.458	
MRPS36	23.25854	23.37478	23.28164	0.06153645			3	3	3	4.24E-57	3.04E+08	38	56.3	11.466	
MRPS7	NaN	NaN	16.06554	N/A			0	1	1	0.003275	3999300	1	9	18.274	
MSH2	19.56127	19.45659	19.7988	0.17535017	+		2	3	3	2.12E-08	23074000	11	4.1	97.321	
MSN	23.34476	23.16524	23.02283	0.16132109			7	9	9	4.58E-42	2.94E+08	32	17	67.819	
MSRA	22.21671	22.43505	22.3592	0.11085199	+	+	5	4	5	6.79E-21	1.7E+08	22	36.6	23.627	
MT-ATP6	NaN	NaN	20.55112	N/A			0	1	1	0.000641	19408000	3	4.4	24.817	
MTCH2	21.97872	22.20604	22.19505	0.12818855			3	3	3	5.69E-12	1.01E+08	20	18.5	33.331	
MT-CO2	22.31321	NaN	NaN	N/A			3	3	2	1.97E-07	93885000	7	11.9	25.565	
MT-CO3	NaN	NaN	16.40806	N/A			0	0	1	0.000617	15174000	3	8	29.95	
MT-CYB	NaN	NaN	20.64209	N/A			1	1	1	0.000162	45055000	7	4.7	42.717	
MTDH	23.48787	23.53848	23.413	0.06312965			9	10	9	1.77E-46	3.02E+08	38	19.4	63.836	
MTHFD1	22.92314	22.86391	22.74567	0.09035531			12	11	10	2.19E-41	2.5E+08	49	17.3	101.56	
MTHFD1L	19.73114	19.63886	19.34782	0.2000642	+		2	3	4	1.76E-08	20603000	5	4	105.79	
MT-ND4	19.9245	20.63856	20.13696	0.36665685			2	2	2	0.000293	32159000	7	5.4	51.58	
MTPAP	NaN	NaN	18.45125	N/A			1	1	1	0.000611	10026000	2	2.1	66.171	
MTPN	NaN	NaN	19.02256	N/A			1	1	1	0.006909	8822300	2	32.7	5.7044	
MTX2	NaN	NaN	17.34551	N/A			1	1	1	0.006632	5205500	3	4.8	25.717	
MYADM	NaN	NaN	21.04647	N/A			1	2	2	4.14E-06	22680000	5	22.6	15.864	
MYH9	NaN	NaN	19.49865	N/A			2	1	2	2.36E-05	1.04E+08	2	2.2	159.86	
MYL12A;MYL12B;	NaN	21.51946	21.77953	0.18389726			3	2	3	6.59E-17	1.19E+08	23	24	19.794	
MYL6	22.42101	22.1384	21.92586	0.24839994	+		4	3	4	3.51E-13	1.11E+08	20	38.6	16.29	
NAA15	20.81276	21.30997	21.23263	0.26754753			3	5	5	8.65E-13	72237000	12	5.8	101.27	
NADK2	NaN	NaN	18.22459	N/A			0	0	1	0.000506	1660200	1	4.3	31.716	
NAP1L1	NaN	NaN	19.73831	N/A			0	1	1	1.39E-06	10714000	2	10.2	20.966	
NAP1L4	NaN	NaN	19.13149	N/A			1	1	1	0.000822	21524000	9	12.8	8.6016	
NAPA	NaN	NaN	19.2013	N/A			1	1	1	1.63E-05	19525000	4	7.8	29.163	
NARS	21.00662	20.39582	20.93991	0.33505243			3	4	3	1.61E-10	40565000	6	6.9	62.942	
NASP	22.29036	22.74366	22.94833	0.33672323			4	5	5	1.36E-25	3.56E+08	49	7.5	85.237	
NAT14	21.69337	21.88718	21.78871	0.09690921			2	2	2	1.35E-05	53564000	5	10.4	17.73	
NCALD	NaN	20.12943	20.60232	0.33438373	+		1	2	2	2.37E-12	22511000	3	25.9	22.245	
NCAM1	25.2149	25.29677	25.21104	0.04842043			15	17	20	3.03E-90	1.39E+09	177	34.3	94.573	
NCDN	NaN	NaN	18.47405	N/A			1	1	1	0.001447	15716000	1	1.7	77.242	
NCL	22.19691	22.36981	22.29566	0.08674118			4	6	7	5.53E-18	1.41E+08	26	9.7	76.613	
NCR3LG1	21.9102	21.88822	22.0879	0.10949316			6	6	6	8.92E-19	1.1E+08	17	15	50.827	
NCS1	24.13226	24.09055	24.0694	0.03198548	+		9	9	9	4.15E-44	4.79E+08	83	62.6	21.878	
NCSTN	21.78025	21.69379	21.6677	0.05891169			5	4	5	1.28E-15	1.04E+08	16	10.4	50.26	
NDFIP2	20.17377	20.97918	21.17518	0.53071073			3	3	3	4.76E-09	53359000	12	18.9	26.019	
NDUFA4	22.18556	22.18389	22.25816	0.04240594			2	2	3	2.60E-09	1.34E+08	16	46.9	9.3697	
NDUFA5;DKFZp78	NaN	NaN	18.92654	N/A			1	1	1	0.003396	16348000	5	14.3	7.8423	
NDUFAF4	25.75297	25.8896	25.80213	0.06920436	+		10	10	9	6.10E-46	1.41E+09	104	53.1	20.266	
NDUFB3	NaN	NaN	19.41547	N/A			1	2	2	1.50E-05	5945600	3	21.4	11.402	
NDUFB7	24.8833	24.89721	24.7747	0.06707725	+		6	6	6	3.39E-56	9.65E+08	161	51.1	16.402	
NDUFS7	NaN	18.37742	NaN	N/A			0	1	0	0.00387	1439700	1	4.9	19.77	
NEDD8;NEDD8-MI	NaN	NaN	19.08734	N/A			0	0	1	0.000888	10991000	1	28	5.8668	
NELFB	19.88335	20.03295	19.99604	0.07793325			3	3	3	4.34E-06	29454000	5	5.5	65.697	
NME1	NaN	NaN	20.58463	N/A			0	0	1	1.73E-32	41250000	7	52.6	17.149	
NME2;NME1-NME	24.78691	24.8943	24.70504	0.09491633			9	9	9	9.40E-34	7.37E+08	88	58.4	30.137	

NME3	NaN	19.13152	NaN	N/A				1	1	0	0.001245	4356500	1	5.9	19.015	
NMT1	NaN	NaN	19.69032	N/A				2	2	2	0.000521	15194000	2	29.2	8.1691	
NNT	21.53896	21.1988	21.14015	0.21532846				3	5	5	1.55E-14	73856000	13	5.8	113.89	
NOC2L	NaN	NaN	18.15694	N/A				1	1	1	0.003643	3642100	2	1.5	84.918	
NOL3	19.55845	NaN	NaN	N/A	+			1	0	1	4.81E-05	3071600	2	7.2	14.924	
NPC1	24.13798	24.36406	24.46351	0.16681939				10	11	11	9.01E-40	6.55E+08	71	9.3	142.17	
NPEPPS	22.79251	23.14546	22.8738	0.18483367				14	16	10	2.06E-43	2.83E+08	56	20.1	102.99	
NPHP3	19.72112	NaN	19.66513	0.03959091	+			2	1	2	9.28E-05	18713000	3	10	26.341	
NPM1	23.99757	23.57207	23.55882	0.24957543				6	6	6	1.66E-27	5.02E+08	54	26.8	29.464	
NR3C1	19.94364	19.89701	NaN	0.03297239				2	2	1	8.68E-06	22580000	4	4.5	60.601	
NRAS;KRAS	23.39545	23.39349	23.27838	0.06703176				4	5	3	5.52E-21	2.44E+08	20	31.7	21.229	
NRP1	21.23386	21.44915	21.80994	0.29108629				4	5	4	9.80E-18	80978000	15	7.9	101.3	
NSUN2	20.58985	20.42931	20.63298	0.10732719	+			2	3	3	6.40E-09	41635000	8	5.6	82.392	
NUDC	NaN	NaN	19.29206	N/A	+			1	1	1	0.003643	28651000	5	3.3	38.242	
NUDT21	NaN	19.03998	NaN	N/A				0	1	0	0.000426	2278800	1	12.8	26.227	
NUDT8	NaN	21.46688	NaN	N/A				1	3	3	1.35E-09	36967000	6	35.7	15.052	
OAT	22.80266	22.70436	22.44731	0.18348988				6	6	6	9.80E-21	2.17E+08	37	17.8	48.534	
OGFRL1	23.16486	23.40198	23.47494	0.16212067	+			7	8	9	7.51E-42	2.54E+08	28	26.6	51.251	
OLA1	NaN	NaN	19.66164	N/A				1	1	1	0.003718	10470000	2	9.5	12.302	
OSTC	NaN	NaN	19.70536	N/A				1	1	1	0.000559	25982000	3	8.1	16.829	
OTUB1	20.70498	20.52401	20.66735	0.09549216				2	2	2	1.07E-06	28087000	5	12	28.05	
OXCT1	19.52476	19.75217	19.22178	0.26609076				2	2	4	1.07E-09	24866000	6	7.9	56.157	
P2RX4	NaN	20.2543	20.26304	0.00618011				1	2	2	0.000178	15310000	2	5.9	32.138	
P4HB	23.11633	23.09852	23.31382	0.11949448				7	8	9	3.70E-32	2.61E+08	37	24.8	57.116	
PA2G4	24.33974	24.48781	24.27597	0.10867959				11	10	10	5.62E-45	6.59E+08	82	32	43.786	
PABPC1	22.31951	22.37626	22.49936	0.09194219	+			8	8	9	4.81E-22	1.81E+08	24	17.4	61.18	
PAFAH1B2	NaN	NaN	18.08364	N/A				1	1	1	0.004203	5673200	4	5.1	18.357	
PAFAH1B3	NaN	19.94792	NaN	N/A				1	2	0	9.44E-06	8422900	3	11	18.356	
PAFAH2	22.02351	21.45372	21.52248	0.31102509	+			5	5	5	7.61E-15	56447000	5	19.9	44.035	
PAICS	23.48677	22.87429	23.43122	0.33872028				10	11	11	2.21E-45	2.98E+08	50	36.5	47.079	
PAIP1	20.38385	20.21347	20.41854	0.1097622				2	2	2	2.01E-06	32710000	10	6	39.908	
PALD1	22.02658	21.59773	21.84487	0.21525528	+			5	6	8	1.09E-22	91871000	18	10.7	96.753	
PALM	22.62881	22.90962	22.96107	0.17883795				4	4	4	2.48E-13	2.27E+08	31	14.2	42.075	
PARK7	22.26708	21.96698	22.26919	0.17387512				5	6	7	7.71E-28	1.56E+08	18	45.5	19.891	
PARP1	22.36084	22.26043	22.14647	0.10725635				3	3	5	5.75E-18	1.29E+08	10	8.4	113.08	
PC	27.4231	27.29815	27.48085	0.09338706	+			41	42	45	0	9.94E+09	976	54.9	129.63	
PCBP1	24.04076	24.23045	24.08432	0.0993594				9	7	8	2.17E-35	5.24E+08	48	37.4	37.497	
PCBP2	23.46326	23.23682	23.60071	0.18374961	+			5	4	4	1.49E-32	4.02E+08	40	31.9	38.15	
PCCA	26.61863	26.34155	26.68164	0.18092576				27	30	31	2.86E-259	5.69E+09	474	66.1	77.047	
PCCB	21.61122	21.29233	21.28918	0.18502726				3	3	3	5.88E-21	1.18E+08	25	21.8	52.423	
PCDH7	NaN	18.80945	NaN	N/A				1	1	0	0.001128	5061500	1	1.4	102.42	
PCDH9	NaN	NaN	18.66962	N/A				1	1	1	0.000734	14620000	5	1.4	113.71	
PCMTD1	21.28455	21.2217	21.35369	0.06601997	+			5	5	5	8.92E-11	54060000	10	14	40.675	
PCMTD2	25.41625	25.28817	25.35777	0.0641204	+			11	12	13	1.14E-75	1.06E+09	148	41.6	41.071	
PCNA	22.87665	22.80835	22.60121	0.14343353				8	9	8	2.97E-33	2.61E+08	27	47.1	28.768	
PDCD4	20.09339	NaN	19.33366	0.53721023				2	3	2	2.34E-09	23610000	7	8.5	50.576	
PDCD6IP	20.95468	20.81636	21.19551	0.19187072				2	4	4	2.39E-14	74616000	16	7.9	96.022	
PDE12	NaN	NaN	18.32017	N/A				1	1	1	0.006974	4550400	1	2.5	52.175	
PDHA1	19.82864	19.88981	19.75991	0.06498665				2	2	2	0.000108	24946000	6	5	40.188	
PDHB	21.32784	21.33063	21.06072	0.15503348				5	5	5	1.07E-11	72446000	13	15.5	37.514	

PDIA3	23.2199	23.51159	23.16601	0.18592683			9	8	5	6.59E-31	3.27E+08	34	26.2	54.963	
PDIA3	NaN	NaN	20.32564	N/A			0	1	1	1.47E-08	18229000	3	20.3	13.519	
PDIA4	NaN	NaN	19.9218	N/A			1	1	2	1.36E-05	17148000	5	3.6	72.932	
PDIA6	NaN	20.67426	20.31197	0.25617772			1	2	2	8.59E-08	25632000	6	9.2	47.837	
PDYN	NaN	NaN	24.11728	N/A			1	1	1	0.001065	4.86E+08	19	2.8	28.385	
PEBP1	22.57847	23.21928	23.05583	0.33297295			6	7	6	5.73E-31	2.5E+08	25	56.1	21.057	
PEF1	NaN	14.31833	NaN	N/A			0	1	0	0.008668	4789200	1	3.9	30.381	
PFKP	21.15487	21.14227	NaN	0.00890955	+		5	4	1	1.17E-10	54841000	8	6.3	85.315	
PFN1	24.80307	24.79417	24.81685	0.01142717	+		5	7	7	3.43E-39	8.26E+08	89	60	15.054	
PGAM1	24.57086	24.64719	24.5251	0.06167957			8	10	9	1.57E-138	8.34E+08	138	64.2	28.804	
PGAM5	NaN	NaN	20.13596	N/A			1	2	2	0.000119	47466000	2	7.5	28.02	
PGD	22.33658	22.65611	22.31108	0.19226517	+		6	8	5	5.96E-32	1.53E+08	23	31.9	51.872	
PGK1	26.06833	25.83002	25.79786	0.14774976			16	16	17	2.09E-152	2.27E+09	202	60	44.614	
PGLS	21.89737	22.21807	22.07845	0.16079604			6	6	6	3.99E-18	1.36E+08	20	45.7	27.547	
PGM1	NaN	20.01345	NaN	N/A			2	4	3	9.58E-07	33366000	4	6.8	61.448	
PGM2	NaN	23.91971	NaN	N/A			0	2	1	1.84E-05	2.93E+08	4	9.7	21.996	
PGRMC1	21.57358	21.68423	21.63533	0.05544922			3	2	3	3.00E-10	1.07E+08	10	20	21.671	
PHB	23.03167	22.5077	NaN	0.37050274			4	2	1	2.23E-16	1.64E+08	24	30.3	22.27	
PHGDH	24.2722	24.10765	24.09031	0.10038372			9	10	9	9.28E-66	5.38E+08	70	22.1	56.65	
PI4K2A	27.04958	26.76446	27.2787	0.25762769			28	27	27	0	3.57E+09	312	72.7	54.022	
PI4K2B	24.37683	24.40931	24.15653	0.13752864			16	16	13	9.25E-55	5.78E+08	72	38.5	54.744	
PIGU	NaN	NaN	20.44162	N/A			2	2	2	2.64E-05	41338000	3	5.1	47.61	
PIK3R4	23.45526	23.13743	23.40717	0.17131269	+		12	13	12	3.04E-44	3.01E+08	41	13.8	153.1	
PIN4	NaN	NaN	18.45314	N/A			1	1	1	0.002146	4933100	3	13.2	9.5428	
PITPNB	20.17426	20.06907	20.03241	0.07363232	+		2	3	3	1.92E-06	35896000	5	11.8	31.54	
PKM;PKM2	26.63062	26.5852	26.67317	0.0439928			22	21	19	4.64E-254	2.73E+09	244	55.4	57.936	
PKP1	NaN	NaN	21.7862	N/A			0	0	4	1.50E-42	32854000	7	6.2	80.496	
PLGRKT	25.02568	24.46532	24.17387	0.43292161	+		4	4	4	8.84E-31	6.36E+08	58	34	17.201	
PLIN3	21.54312	21.11076	21.22276	0.22439166			5	4	4	6.89E-20	1.37E+08	24	16.8	45.803	
PLP2	26.23776	26.3554	26.2842	0.05925268			3	3	2	7.94E-58	2.08E+09	53	27	16.691	
PLS3	26.28132	26.19888	26.08742	0.09731127			23	24	24	5.61E-154	2.43E+09	264	47.9	70.81	
PLSCR1	25.73487	25.71345	25.66202	0.03744103			7	8	8	3.70E-91	1.96E+09	137	34.7	34.217	
PLSCR3	22.98183	23.06924	23.04255	0.04479543			4	5	6	6.59E-22	2.71E+08	36	26.1	31.648	
PLSCR4	NaN	18.45455	NaN	N/A			0	1	0	0.005516	1518700	1	4	24.797	
PLXNB2	21.36613	21.16288	21.12603	0.12930358			4	4	4	1.67E-11	56497000	9	2.6	205.12	
PMPCA	21.39947	21.67534	21.28427	0.20096063			4	4	4	1.09E-26	71790000	14	9.1	58.252	
PMPCB	21.10194	20.92644	21.19268	0.13535			2	2	3	2.59E-10	61659000	9	6.7	54.366	
PNP	21.12711	20.90257	20.92071	0.12473187			5	6	5	2.36E-15	72023000	13	25.3	32.118	
PODXL	21.95291	22.3584	22.11137	0.20435081			3	3	3	1.04E-07	1.31E+08	10	5.5	55.385	
PPA1	21.67379	21.2972	21.20281	0.24918255			3	3	4	7.87E-12	63727000	8	16.3	32.66	
PPA2	19.70708	19.98579	19.73948	0.15242355			2	2	2	3.48E-05	23105000	8	10.8	25.991	
PPIA	25.38123	25.10161	25.22836	0.14001318			6	7	8	6.20E-36	1.04E+09	80	52.7	18.012	
PPIB	21.4763	21.52922	21.7727	0.15808015			3	4	4	1.24E-12	91776000	14	19	23.742	
PPM1A	26.25187	26.22971	26.56353	0.18666316	+	+	15	15	16	1.82E-170	2.21E+09	201	52.9	42.447	
PPM1B	25.35531	25.4943	25.62238	0.13357213	+	+	11	11	10	3.95E-127	1.2E+09	101	45.5	52.642	
PPM1G	28.42005	28.45544	28.45862	0.02140954	+	+	20	24	23	0	1E+10	538	63.9	59.271	
PPME1	20.24702	20.16693	19.66965	0.31279864			2	2	2	4.05E-05	25290000	2	6.5	42.315	
PPP1CA	21.09229	20.89364	20.94692	0.10282106			2	3	3	1.53E-06	52097000	8	8.5	37.512	
PPP1CB	NaN	NaN	19.26492	N/A			0	1	1	1.92E-06	13606000	6	5.8	37.186	
PPP2CA;PPP2CB	21.26819	21.30302	NaN	0.02462853			3	2	1	8.05E-20	47984000	8	11	35.594	

PPP2R1A	24.49741	24.38001	24.60303	0.11156184			13	15	16	1.89E-64	7.77E+08	100	39.4	65.308	
PPP2R4	NaN	19.79346	NaN	N/A	+		2	2	1	5.20E-05	16093000	2	35.9	7.4004	
PPP3CA	22.76314	23.06457	22.64733	0.21539159			6	6	8	1.52E-21	1.83E+08	26	17.2	57.658	
PPP3R1	25.27746	25.06719	24.98177	0.15217458	+	+	8	7	8	8.28E-69	1.21E+09	105	84.7	19.3	
PPP5C	NaN	19.52277	NaN	N/A			1	2	1	1.30E-05	13386000	10	6.2	42.394	
PRAF2;WDR45	23.54946	23.92388	23.47803	0.23946983			3	4	4	1.65E-12	3.82E+08	38	26.4	19.258	
PRDX1	25.85567	25.85277	25.9324	0.04516053			8	8	10	1.50E-46	1.92E+09	93	53.3	22.11	
PRDX2	23.03736	23.09984	22.94799	0.07632078			3	3	3	2.54E-15	2.63E+08	43	19.2	21.892	
PRDX3	21.53711	22.32773	22.46129	0.49950424			3	4	5	2.50E-16	1.37E+08	20	31.5	25.838	
PRDX4	22.60756	22.6871	22.79869	0.09601182			3	3	4	3.40E-26	1.6E+08	25	35.4	30.54	
PRDX5	20.97365	NaN	NaN	N/A	+		2	1	1	1.18E-05	30105000	11	11.7	17.031	
PRDX6	23.84173	23.71576	23.73511	0.06783643			8	7	8	1.10E-51	4.38E+08	73	50.9	25.035	
PRKAA1	23.56256	23.28701	23.37928	0.14025757			7	8	8	8.35E-25	2.47E+08	38	20	64.009	
PRKAB1	25.16092	24.97802	25.02314	0.09528165	+		7	7	8	1.57E-105	8.52E+08	74	51.5	30.382	
PRKAB2	21.99766	21.8573	22.0418	0.09634099	+		3	4	4	4.10E-17	1.05E+08	18	16.9	30.302	
PRKACA	27.56042	27.45018	27.49709	0.05532343	+	+	16	17	18	1.49E-121	4.92E+09	322	63.2	40.589	
PRKACB	23.77636	23.69104	23.74294	0.04299226	+	+	6	5	6	3.38E-84	4.63E+08	55	62.7	40.622	
PRKACG	NaN	NaN	22.12452	N/A	+		1	1	1	2.06E-28	1.66E+08	16	14.8	40.434	
PRKAG1	21.73389	21.60046	21.73649	0.07779726	+		4	4	3	1.49E-12	70729000	9	20.2	28.285	
PRKAR2A	NaN	20.90301	21.1054	0.14311134			2	3	3	4.02E-09	45683000	4	11.8	43.066	
PRKCZ	NaN	NaN	19.10288	N/A			1	0	1	0.005314	11221000	1	16.4	6.6642	
PRKDC	22.80825	22.69861	22.80802	0.06323439			17	17	16	3.00E-48	2.71E+08	56	5.8	469.08	
PRMT1	22.20225	22.52358	21.97603	0.27514828	+		6	7	6	5.30E-21	1.38E+08	19	25.5	37.709	
PRNP	22.91942	22.80353	23.42291	0.32928312			4	5	5	1.08E-22	1.97E+08	30	21.5	26.885	
PROCR	23.15126	23.08003	23.00551	0.07288119			3	4	4	4.74E-21	1.9E+08	23	20.6	26.671	
PRPF19	22.11175	21.85129	21.71108	0.20332023			4	3	3	4.07E-13	88949000	9	8.7	55.18	
PRPF38B	21.00237	21.7149	21.51586	0.36764745			2	3	3	1.13E-13	95439000	16	10.3	64.467	
PRSS3	NaN	NaN	21.34229	N/A	+		1	1	1	3.56E-16	1.07E+08	0	7.3	19.288	
PSAT1	19.90984	19.79584	19.7698	0.07448186			3	2	3	4.57E-06	28048000	4	7.6	40.422	
PSEN2;PSEN1	22.26262	22.36105	22.22726	0.0693284			2	2	2	1.10E-05	1.44E+08	20	6.1	40.543	
PSMA1	22.20398	21.83384	22.70835	0.43896857			3	4	5	1.52E-13	1.21E+08	13	21.3	29.555	
PSMA3	21.46837	22.17725	21.82042	0.35444269			2	4	4	1.19E-12	93841000	16	15.3	27.647	
PSMA4	22.63323	22.01913	22.04657	0.34690098			2	3	3	4.97E-12	1.39E+08	23	23.2	24.526	
PSMA5	21.4866	21.13057	20.93488	0.27971619			3	3	3	2.90E-11	58355000	10	19.9	26.411	
PSMA6	22.10904	21.89023	21.74534	0.18309771			4	4	4	4.80E-16	1.26E+08	22	27.7	16.645	
PSMA7	21.35369	21.60829	NaN	0.18002939			3	3	2	5.66E-07	70511000	6	12.9	27.887	
PSMB2	22.00268	21.80652	21.59928	0.20172536			3	2	2	2.48E-07	1.12E+08	12	14.4	22.836	
PSMB4	NaN	NaN	19.48318	N/A			1	1	1	0.008412	17624000	2	3.8	29.204	
PSMB5	21.55796	22.16091	21.78784	0.30429556			5	5	6	2.35E-23	1.34E+08	24	30.4	28.48	
PSMB6	21.38911	21.73186	21.43802	0.18538775			2	3	3	6.56E-10	1E+08	16	13	25.357	
PSMC1	28.85127	28.75373	28.87865	0.06566166	+	+	24	26	26	0	1.32E+10	755	63	49.184	
PSMC2	25.19103	25.31268	25.99468	0.43316203			13	14	17	1.29E-82	1.22E+09	91	50.8	48.633	
PSMC3	22.99965	22.75236	22.83453	0.12594236			4	5	6	1.26E-20	2.6E+08	19	19.4	47.352	
PSMC4	21.61903	21.79432	21.78129	0.09765985			4	5	4	2.15E-10	94116000	11	11.6	43.507	
PSMC5	25.49607	25.58964	25.79813	0.15463056			4	3	5	3.54E-25	1.28E+09	61	18.3	44.784	
PSMC6	22.00309	22.2406	22.13214	0.11890365			6	7	7	9.29E-26	1.35E+08	23	24.2	44.172	
PSMD1	22.13427	22.2878	21.44693	0.44778531			5	4	4	6.08E-17	1.38E+08	18	7.3	102.26	
PSMD10	NaN	NaN	16.15181	N/A			0	0	1	0.006557	4249900	2	9.1	11.926	
PSMD11	21.94774	21.82582	21.33887	0.32215614			3	4	5	5.40E-15	95605000	20	14.2	47.463	
PSMD12	21.83256	21.60481	21.64082	0.12242754			4	6	6	3.20E-17	1.09E+08	28	14	52.904	

PSMD13	22.19757	22.29589	22.04883	0.12438452				7	8	6	3.41E-30	1.83E+08	35	24.7	42.945	
PSMD14	NaN	20.2958	20.49989	0.14431342				1	2	2	3.58E-07	39075000	6	6.5	34.577	
PSMD2	24.14406	24.12943	24.24474	0.06277857				18	14	17	5.34E-103	6.21E+08	92	31.1	100.2	
PSMD3	22.0467	21.49443	21.66418	0.28288359				5	5	4	1.43E-17	95938000	13	16	41.183	
PSMD6	NaN	21.05933	21.38274	0.2286854				3	4	3	2.35E-14	79084000	11	20.3	45.531	
PSMD8	21.68817	21.553	21.40567	0.14129361				2	5	5	1.70E-13	1.18E+08	23	29.7	19.781	
PSME2	NaN	NaN	20.33941	N/A				0	1	2	1.64E-05	17704000	3	9.2	26.011	
PSME3	21.20628	NaN	NaN	N/A				2	2	2	1.27E-10	47687000	9	19.7	22.455	
PTBP1	20.728	20.2596	19.62083	0.5557654				2	3	4	3.30E-15	51454000	7	11.5	57.221	
PTCD3	NaN	NaN	17.73635	N/A				1	1	1	0.005711	6686700	3	3.2	31.863	
PTGES3	NaN	21.32811	21.11889	0.14794088				2	2	2	3.72E-06	71886000	9	20.9	16.476	
PTGFRN	26.91718	27.02559	26.80403	0.11078845	+			23	27	24	2.36E-238	3.47E+09	299	43.5	98.555	
PTK7	24.65971	24.51737	24.60043	0.0715003	+			17	20	18	5.17E-82	6.62E+08	83	25	118.39	
PTPLAD1	24.6997	24.61824	24.55497	0.07255526				7	10	10	1.61E-45	6.68E+08	68	29.8	43.159	
PTPN1	NaN	19.95234	20.3811	0.3031791				1	3	2	7.57E-09	25376000	4	9.4	49.966	
PTRH2	21.6352	21.04547	21.23707	0.30083205				3	3	3	8.83E-13	71580000	9	29.1	19.193	
PTTG1IP	23.00422	22.95298	22.66531	0.18268349	+			2	3	3	2.29E-11	2.22E+08	34	22.8	20.324	
PVR	24.04435	24.62126	24.30173	0.28901241				4	5	4	3.01E-21	6.87E+08	43	13.5	39.304	
PYGL	21.01318	20.17901	20.03725	0.5273163				3	3	2	1.24E-06	20650000	6	3.3	93.133	
QARS	NaN	18.82839	NaN	N/A				0	1	1	0.00083	2967300	1	6.7	28.565	
QKI	21.84257	21.80747	21.615	0.12251858				2	3	2	1.21E-07	75631000	12	10.1	35.232	
RAB14	NaN	18.84337	19.34553	0.35508074				0	2	2	0.000455	30018000	4	7.9	23.897	
RAB1A;RAB1B;RAB	22.6502	22.08534	23.28376	0.5995381				4	4	4	1.52E-14	2.11E+08	17	37	19.018	
RAB21	NaN	19.8935	NaN	N/A				1	2	1	0.000247	6726700	1	9.3	24.347	
RAB5C	NaN	NaN	19.05558	N/A				1	1	1	0.000249	18236000	8	44.4	2.803	
RAB6B;RAB6A;RAB	NaN	NaN	22.64674	N/A				1	1	1	2.22E-05	1.38E+08	12	22.4	5.8575	
RAB7A	NaN	21.25321	NaN	N/A				1	3	2	1.57E-06	35824000	10	22.7	17.037	
RAC1;RAC3;RAC2	21.9523	21.97809	21.99473	0.0213788				3	5	4	7.78E-12	1.24E+08	16	27.1	21.45	
RAD23B	21.52081	22.14283	22.03231	0.33185233				4	5	5	2.04E-15	1.52E+08	21	21	43.171	
RAN	24.56581	24.64763	24.74607	0.09025761				5	6	6	1.60E-29	7.34E+08	68	31.5	24.423	
RANBP1	23.40002	23.21926	22.99205	0.20442524	+			2	3	3	1.70E-12	2.34E+08	21	15.4	23.31	
RANGAP1	22.50555	22.32191	22.2573	0.12879322				8	7	10	2.84E-35	2.18E+08	44	21.3	63.541	
RAP1B;RAP1A	NaN	NaN	20.89955	N/A				1	1	2	1.28E-05	42316000	6	43.8	5.351	
RAP2B	25.26853	25.33002	25.11004	0.11349839				8	8	8	1.44E-47	1.13E+09	103	62.3	20.504	
RAP2C	22.81888	22.84078	22.65154	0.10351656				4	4	4	8.60E-42	1.87E+08	24	59	20.745	
RAPGEF3	NaN	NaN	21.40468	N/A				1	1	1	0.007202	1.67E+08	9	1.5	92.15	
RARS	20.94535	20.95383	20.91918	0.01806194				2	2	2	1.70E-11	49153000	9	8.2	75.378	
RBM39	19.41551	20.40624	19.69489	0.51081927				3	3	3	2.95E-09	43638000	11	13.7	36.514	
RCC2	NaN	20.69659	20.75205	0.03921614				2	2	3	5.35E-09	47052000	8	6.5	56.084	
RCE1	19.98398	20.23888	20.02689	0.13647652				2	2	1	4.67E-06	13874000	5	13.1	22.712	
RECQL	NaN	NaN	18.28794	N/A				1	1	1	0.002657	4510700	1	9.2	14.634	
REEP5	23.0941	23.61291	23.81264	0.37088984				3	4	4	2.29E-30	3.68E+08	49	15.9	21.493	
RER1	22.23143	21.93031	22.21632	0.16965813				3	3	3	8.78E-10	1.09E+08	10	23.4	18.388	
RFC2	NaN	NaN	20.07091	N/A				0	2	2	1.32E-08	29878000	8	10	35.243	
RFC3	NaN	NaN	18.90166	N/A				1	1	1	0.000954	8259800	2	5.9	34.756	
RFC4	NaN	NaN	19.39186	N/A				1	2	2	0.000751	21681000	3	6.3	39.681	
RFT1	NaN	18.91649	NaN	N/A	+			1	2	1	2.45E-05	8431100	3	4.2	55.835	
RFTN1	22.32652	22.61002	22.3456	0.15845832	+			5	5	6	1.10E-18	1.28E+08	17	14	63.145	
RFX1	20.34072	NaN	20.49512	0.10917729				3	1	2	1.05E-07	17100000	4	4.2	104.76	
RGS19	22.56062	22.51627	22.45109	0.05509413				3	3	3	1.36E-08	1.78E+08	16	16.1	24.635	

RHBDD2	21.1606	20.80688	20.89164	0.18468062			3	3	3	2.05E-09	71679000	20	11.8	39.202	
RHOA;RHOC	21.05225	21.24609	21.15283	0.09694303			2	2	2	1.34E-13	58289000	13	23.8	21.768	
RHOB	24.25054	24.36986	24.37119	0.06927656			6	5	5	5.78E-22	5.85E+08	52	36.2	22.123	
RNF11	21.58086	22.22993	NaN	0.4589618	+		2	2	1	7.54E-10	50622000	5	18.8	17.444	
RNF141	24.62561	24.68239	24.7517	0.06314868	+	+	8	7	8	2.54E-57	8.38E+08	80	57.4	25.535	
RNF34	NaN	NaN	17.71519	N/A			1	2	1	2.08E-08	5101700	2	6.7	41.64	
RNH1	NaN	NaN	20.14071	N/A			0	1	3	1.43E-08	32885000	5	11.1	49.973	
RNPEP	NaN	18.90578	NaN	N/A			1	2	1	0.000789	5473700	0	3.1	72.595	
ROR1;ROR2	NaN	17.84983	NaN	N/A			0	1	0	0.003064	4098800	1	2.5	43.825	
RP2	25.85818	25.71411	25.68279	0.09354033	+	+	11	11	11	3.04E-38	1.43E+09	115	26.9	39.641	
RPA2	20.18616	20.40748	20.35499	0.11564407	+		2	2	2	0.000263	40789000	9	11.2	19.433	
RPL10	22.78925	23.08011	23.23796	0.22761694	+		6	5	5	7.64E-22	4.6E+08	24	35.5	22.975	
RPL10A	23.40886	23.14798	23.22445	0.13411008			4	4	4	2.39E-14	2.93E+08	30	26.7	24.831	
RPL11	23.19084	22.98973	23.10644	0.10098665			2	3	3	1.67E-78	2.37E+08	24	22	20.124	
RPL12	23.00965	23.03258	22.86024	0.09358615			5	4	6	3.58E-35	2.19E+08	29	54.5	17.818	
RPL13	27.39566	27.73583	27.45944	0.18081987			11	13	13	0	1.11E+10	425	46.9	24.261	
RPL13A	25.34167	25.13864	25.18359	0.10663877			5	5	5	3.89E-25	1.72E+09	144	30	23.577	
RPL14	22.21963	22.21021	21.54118	0.38901248			3	2	3	1.42E-12	1.56E+08	25	27.4	14.558	
RPL15	26.70609	26.47917	26.74766	0.14451507	+		9	10	11	7.79E-90	6.07E+09	363	52.5	24.146	
RPL17	NaN	NaN	21.32411	N/A			1	1	1	0.000356	63377000	5	9.6	17.094	
RPL18	27.40211	27.90139	28.13124	0.37276939	+		11	12	11	6.72E-251	1.45E+10	493	54.3	21.634	
RPL18A	22.97775	23.08464	23.10717	0.06914069			3	3	4	1.25E-17	3.14E+08	33	27.7	16.714	
RPL19	24.75888	24.77228	24.70287	0.03682035	+		3	4	4	2.32E-38	1.15E+09	64	22.3	23.134	
RPL21	21.77812	21.82096	22.02516	0.13201115			2	2	3	3.16E-16	1.13E+08	17	20.6	18.565	
RPL22	22.95757	22.95704	23.2365	0.16119353			2	2	2	8.96E-09	2.62E+08	17	51.1	5.0827	
RPL23	23.7062	24.04076	24.06283	0.19983428			5	4	5	9.67E-32	5.17E+08	48	43.6	14.865	
RPL23A	NaN	NaN	20.22588	N/A			1	1	1	0.001614	28597000	3	18.6	7.9232	
RPL24	23.16013	23.24053	23.16498	0.04508415			4	4	4	1.08E-14	3.21E+08	36	39.7	14.369	
RPL27	22.36054	22.16797	22.33224	0.10397814	+		2	2	2	3.10E-10	1.47E+08	28	20.6	15.798	
RPL27A	25.45435	25.1848	25.4646	0.15866648			5	6	5	2.39E-23	1.8E+09	126	35.8	16.561	
RPL28	26.16492	26.27403	26.24027	0.05586047			9	12	11	7.10E-92	4.38E+09	195	61.3	15.747	
RPL29	24.23936	25.27767	24.79843	0.51966622			2	3	3	1.11E-21	1.78E+09	45	23.3	17.752	
RPL3	24.17516	24.38726	24.29467	0.10633435			9	10	10	4.95E-48	6.26E+08	72	33	46.108	
RPL30	22.02658	22.14995	21.74968	0.20498367			3	3	3	1.65E-14	1.08E+08	13	31.6	12.656	
RPL31	24.50706	24.61712	24.8986	0.20192728			4	4	4	4.45E-157	1.33E+09	79	32.8	14.463	
RPL32	28.755	28.67243	28.84697	0.08731218			11	12	12	2.10E-291	2.9E+10	1376	60.2	15.616	
RPL34	25.53083	26.36179	26.13208	0.42910037			6	7	7	6.28E-37	6.21E+09	189	35.9	13.293	
RPL35	23.46712	23.80577	23.88511	0.22199636			3	4	5	5.74E-39	7.34E+08	61	29.3	14.551	
RPL35A	22.0793	22.16797	22.00963	0.07935976			3	3	3	7.56E-07	1.13E+08	9	20.9	12.538	
RPL36	26.3435	26.71122	26.88658	0.27715971			8	7	8	2.39E-70	5.55E+09	202	43.8	12.254	
RPL36A;RPL36AL	21.17725	21.72205	21.59204	0.28453482			2	2	3	1.62E-08	1.25E+08	26	32.1	12.441	
RPL37	NaN	NaN	22.2173	N/A			1	1	1	5.07E-08	3.05E+08	20	8.5	9.7223	
RPL38	21.1379	20.85818	NaN	0.19779191			2	2	1	1.36E-06	23726000	5	18.8	7.5649	
RPL39P5;RPL39	23.43746	23.38073	24.06357	0.37892446			2	2	2	9.47E-13	5.66E+08	125	19.6	6.3225	
RPL4	25.96188	25.80359	25.54896	0.2083247			12	16	14	1.90E-66	1.96E+09	173	45.9	47.697	
RPL5	23.90325	24.28164	23.91488	0.21518486	+		7	7	7	2.65E-37	4.71E+08	36	35	34.362	
RPL6	25.08087	25.45447	25.22354	0.18852955			9	11	11	2.23E-60	2.27E+09	215	42.7	32.728	
RPL7	24.06118	23.92723	24.07594	0.08192997			7	7	6	4.70E-28	5.22E+08	51	34.7	29.225	
RPL7A	23.52361	23.8811	23.7681	0.18273089			4	6	6	4.33E-24	4.72E+08	43	36.8	29.995	
RPL8	22.90852	23.18165	23.10476	0.14084401	+		4	5	4	2.66E-24	3.7E+08	47	20.2	28.024	

RPL9	22.61279	22.06009	22.22081	0.28429929			5	5	5	7.12E-18	1.7E+08	26	33.3	21.863	
RPLP0;RPLP0P6	24.94633	24.60269	24.84795	0.17697436			9	9	9	5.74E-73	9.1E+08	121	42.3	34.273	
RPLP1	NaN	NaN	20.63148	N/A			1	1	1	9.76E-10	58002000	8	14	11.514	
RPLP2	NaN	NaN	20.16079	N/A			0	1	1	1.46E-05	9037600	1	13	8.9857	
RPN1	21.2111	21.83434	21.3574	0.32590989			3	4	4	6.79E-15	59327000	12	11.5	68.569	
RPS11	22.80477	22.94871	22.90231	0.07346851			5	6	5	3.49E-19	2.43E+08	32	51.3	18.431	
RPS12	22.68759	22.73202	22.54293	0.09887329			3	4	2	2.18E-18	1.77E+08	28	31.8	14.515	
RPS14	22.48323	22.87493	22.68665	0.19589876			4	5	5	3.13E-22	2.63E+08	34	31.8	16.273	
RPS15A	23.82668	23.66243	23.78007	0.08464605			4	5	5	2.46E-16	3.77E+08	32	54	11.477	
RPS16	23.23856	23.07971	23.24962	0.09506581			7	7	7	1.02E-20	2.76E+08	41	42.6	14.419	
RPS17L;RPS17	20.60549	20.23713	20.50096	0.18983409	+		3	3	2	1.09E-09	48831000	10	48.9	15.55	
RPS18	NaN	NaN	19.7412	N/A			1	1	1	0.000462	24741000	5	5.9	17.718	
RPS19	NaN	NaN	19.09375	N/A			1	1	1	0.002234	14018000	4	12.7	8.1894	
RPS2	24.73836	24.67266	24.69291	0.03364584			10	12	9	5.94E-44	8.74E+08	96	42.7	31.324	
RPS20	NaN	23.11851	NaN	N/A			2	3	2	3.61E-13	76676000	8	25.2	13.373	
RPS23	22.7769	22.83686	22.4668	0.19862092	+		2	2	2	3.95E-15	2.64E+08	51	28.7	15.807	
RPS24	20.73603	20.08939	NaN	0.45724353			2	2	1	5.65E-07	31889000	5	20.8	15.069	
RPS25	23.43046	23.36065	23.59263	0.11901489			3	3	2	1.89E-10	3.92E+08	48	28	13.742	
RPS26;RPS26P11	NaN	22.4958	22.71977	0.15837071			0	2	2	8.42E-07	2.47E+08	17	20.9	13.015	
RPS27	NaN	20.74846	20.67891	0.04917928			1	1	1	1.73E-16	33938000	3	38.1	9.461	
RPS27L	22.98755	22.7006	22.75766	0.15190214			2	2	3	1.47E-16	2.12E+08	17	38.1	9.4771	
RPS3	25.33599	25.32089	25.66156	0.19247506			14	13	15	2.23E-58	1.29E+09	114	70.8	26.688	
RPS3A	25.28076	25.43965	25.54951	0.13511835			10	10	11	5.59E-45	1.15E+09	95	52.7	29.945	
RPS4X	24.53937	24.44152	24.28814	0.1266337			8	9	9	3.27E-46	6.52E+08	84	47.1	29.597	
RPS5	25.21519	24.30772	24.01546	0.62560235			5	6	7	2.45E-60	7.69E+08	99	54	22.391	
RPS6	23.68196	23.63312	23.99326	0.19536029			4	3	4	5.45E-46	6.43E+08	66	18.9	28.68	
RPS8	24.15012	23.98963	23.98503	0.09401499	+		7	7	7	3.91E-72	5.6E+08	85	43.3	24.205	
RPS9	26.40246	26.9695	27.31354	0.46006605			12	14	14	2.54E-61	5.61E+09	278	50.5	22.591	
RPSA;RPSAP58	22.47415	22.45603	22.81382	0.2015431			5	6	5	1.00E-33	1.99E+08	22	28.1	29.404	
RQCD1	NaN	NaN	18.43604	N/A			1	1	1	0.00521	17769000	4	3.7	33.631	
RRAS	20.31717	20.20311	20.46004	0.12873393			2	2	2	2.33E-21	27662000	5	22.5	23.48	
RRAS2	24.71109	24.9252	24.8998	0.11697558			9	9	9	3.27E-37	8.28E+08	62	47.5	23.399	
RRM1	22.30126	22.25914	22.28988	0.02178894			7	6	7	1.16E-22	1.53E+08	25	13.5	79.219	
RRM2	NaN	20.11464	19.8437	0.19158351	+		0	2	2	9.50E-06	20192000	4	8.3	33.789	
RSPRY1	21.26505	NaN	21.02418	0.17032081			3	1	3	1.08E-12	45317000	6	11.1	64.18	
RTN3	22.00765	NaN	22.50293	0.35021585			2	1	2	2.06E-09	1.2E+08	25	11	25.609	
RTN4	23.75817	23.79452	23.41519	0.20930355			4	4	4	2.39E-18	3.79E+08	33	20	37.144	
RUVBL1	21.10303	21.39973	21.09378	0.17403154			6	6	7	3.14E-17	79579000	14	18.6	50.227	
RUVBL2	21.87481	21.93901	22.01828	0.07186679			7	7	8	2.07E-20	1.46E+08	19	22.2	51.156	
S100A11	21.27929	21.27202	21.3673	0.05303598			2	3	4	3.09E-09	1.01E+08	12	37.1	11.74	
S1PR2	NaN	NaN	19.57184	N/A	+		1	1	1	0.002354	9510900	3	2.5	38.867	
SAE1	21.25574	20.18834	21.22288	0.60700021			4	4	3	2.38E-10	44714000	8	19.6	29.422	
SAMM50	24.13116	24.02707	24.05076	0.054559	+	+	10	10	10	9.07E-36	4.25E+08	40	32.2	51.976	
SARS	NaN	NaN	19.92236	N/A			1	2	2	0.000107	9118100	2	3.3	58.777	
SCAMP1	24.05358	23.40873	23.86051	0.33096141			4	5	6	4.82E-64	5.7E+08	61	27.2	37.873	
SCAMP2	24.64515	24.96393	25.08788	0.2283981			3	3	3	1.88E-99	1.02E+09	65	13.7	36.648	
SCAMP2	NaN	NaN	18.33852	N/A			1	1	1	6.65E-09	3809100	1	9.4	28.649	
SCAMP3	26.81543	26.80181	26.85139	0.02561511			7	7	8	3.89E-222	3.12E+09	222	38.9	38.287	
SCAMP4	NaN	NaN	23.6856	N/A			1	2	1	5.14E-65	3E+08	23	10.3	22.047	
SCARB1	23.00758	23.4256	22.90562	0.27553456	+		8	7	7	1.53E-27	2.76E+08	34	19.8	53.579	

SCARB2	26.36123	26.3659	26.29822	0.03779914	+		11	11	13	1.66E-132	2.9E+09	170	37.4	54.29	
SCARF2	21.35762	21.51528	21.29541	0.11333547			4	3	4	2.19E-13	87420000	12	5.7	91.815	
SCRIB	23.46551	23.29165	23.32224	0.09281643			13	10	13	9.44E-47	2.73E+08	50	10.9	174.88	
SDHA	20.65918	20.57182	20.25845	0.21071982			2	2	2	6.04E-08	46273000	8	4.5	63.566	
SEC22B	19.92402	19.9607	19.90654	0.02764139			3	2	3	1.29E-07	30106000	6	14.9	24.593	
SEC61A1;SEC61A2	23.19867	23.37172	22.78355	0.30227329	+		6	5	4	1.48E-18	3.22E+08	35	12.4	52.264	
SEC61B	NaN	NaN	19.65239	N/A			0	1	1	0.008032	26099000	2	15.6	9.9743	
SEC62	20.30841	20.09661	19.95886	0.1760774			2	2	2	0.00078	27566000	5	20	9.8703	
SELENBP1	NaN	NaN	20.17451	N/A			0	0	1	0.001828	6616500	2	8.1	13.577	
SEPT11	20.36538	20.42542	20.27855	0.0738411			3	3	2	4.85E-07	38501000	5	9.4	49.005	
SEPT7	20.7786	20.80554	20.99134	0.11583444			2	4	3	3.32E-09	46067000	7	12.9	43.036	
SEPT9	NaN	20.12187	NaN	N/A	+		2	2	1	2.47E-05	12172000	2	3.9	63.502	
SERINC1	25.21312	24.93459	25.16465	0.14880407	+		4	4	3	1.04E-99	1.03E+09	61	10.4	50.494	
SERINC3	22.79215	22.85668	22.26745	0.32317854	+		3	4	3	2.88E-12	1.43E+08	20	10	46.821	
SERINC5	NaN	NaN	21.03476	N/A			2	2	3	2.10E-09	54162000	6	9.4	46.373	
SERPINB6	23.65447	23.45989	23.28292	0.18584454			11	10	9	5.20E-31	2.78E+08	38	39.4	42.621	
SERPINH1	22.10047	21.9124	21.25788	0.44229038			3	5	5	1.58E-28	73732000	13	16.5	46.44	
SF3B1	NaN	NaN	19.42319	N/A			2	2	2	5.21E-06	21608000	4	1.4	145.83	
SF3B3	21.14464	20.73884	20.60612	0.28056254			5	3	3	7.52E-10	26243000	4	4.2	135.58	
SFN	NaN	NaN	24.41596	N/A			1	1	2	2.70E-19	7.2E+08	30	20.8	24.336	
SFPQ	19.96818	20.2731	20.13558	0.15270381			2	2	2	0.000246	26672000	2	3.7	76.149	
SFT2D1	20.60368	NaN	NaN	N/A			1	0	0	0.000548	6339000	1	17	17.804	
SFT2D2	21.82796	21.91265	21.76036	0.07630465			1	1	1	4.92E-08	72172000	10	15.7	11.756	
SFT2D3	23.9558	24.13312	23.8531	0.14165738			4	4	4	6.38E-56	5.28E+08	46	27	21.789	
SFXN1	22.98	22.97628	22.87072	0.06204685			6	7	6	3.46E-56	2.4E+08	40	35.1	35.619	
SGMS2	NaN	NaN	18.6287	N/A			1	1	1	0.000683	13435000	2	2.7	42.28	
SGTA	NaN	20.84076	20.70269	0.09763023			0	2	2	3.08E-10	51623000	9	17.3	34.063	
SHISA2	NaN	21.79364	21.94528	0.10722567			1	2	2	4.51E-12	96252000	9	15.6	31.375	
SHMT2	24.38824	24.42522	24.31015	0.05874617	+		13	12	12	4.41E-48	5.47E+08	84	35.6	53.454	
SKP1	20.68106	20.36003	20.71431	0.19565281			2	2	2	0.00034	24510000	7	14.8	16.034	
SLC11A2	20.95362	NaN	19.827	0.79664064			2	1	2	6.48E-06	24483000	4	4.1	52.78	
SLC16A1	NaN	21.75377	21.63418	0.0845629			1	2	2	1.17E-13	1.03E+08	10	7.2	46.233	
SLC17A5	22.33563	22.68295	22.6174	0.1845365			5	5	5	1.10E-13	1.58E+08	26	9.3	54.639	
SLC19A1	20.65071	21.14476	20.77892	0.25637281			2	2	2	1.35E-05	54581000	9	3.9	64.868	
SLC1A4	21.93366	22.54151	22.6678	0.39251164			5	6	6	4.31E-23	1.36E+08	16	17.7	55.722	
SLC1A5	26.31572	26.44929	26.26912	0.09351803			12	14	14	1.12E-218	2.31E+09	223	35.3	56.598	
SLC23A2	NaN	20.56151	20.33178	0.16244364			1	2	2	1.44E-05	20862000	6	7.1	58.176	
SLC25A1	20.82198	20.91013	22.27318	0.81359871			2	2	3	4.04E-09	46966000	10	11.3	34.012	
SLC25A10	18.95123	NaN	NaN	N/A			1	0	0	0.001778	6981500	3	6.6	26.541	
SLC25A11	21.45282	20.93575	21.20461	0.25860372			4	5	6	5.51E-15	70193000	14	19.9	32.182	
SLC25A13	NaN	20.0466	20.04206	0.00321026			2	3	3	7.10E-07	15816000	4	5.3	62.244	
SLC25A22	NaN	NaN	19.97631	N/A			0	1	2	0.001487	17154000	2	9.6	34.47	
SLC25A3	24.64966	24.33926	24.34245	0.17829578			7	6	6	1.31E-19	5.6E+08	45	19.7	39.958	
SLC25A4	21.8691	NaN	NaN	N/A	+		2	1	1	4.96E-48	59818000	10	36.2	33.064	
SLC25A5	26.72559	26.4883	26.58205	0.11951244			13	14	13	8.87E-68	3.07E+09	207	42.3	32.852	
SLC25A6	23.57819	23.05984	23.3676	0.26068854			4	4	4	6.07E-53	2.81E+08	32	41.9	32.866	
SLC26A11	20.24296	20.66014	20.503	0.21069446			3	3	3	1.35E-12	27998000	8	6.6	65.298	
SLC26A2	NaN	NaN	20.52497	N/A			1	1	2	7.47E-05	17508000	4	2.6	81.661	
SLC27A4	NaN	NaN	19.55392	N/A			1	1	2	0.000581	12615000	2	3	72.063	
SLC29A1	20.56802	NaN	20.81948	0.17780907			2	2	3	4.38E-15	44667000	7	9.4	50.219	

SLC2A1	NaN	NaN	18.9048	N/A			1	1	1	0.000134	11201000	3	3.7	54.083	
SLC2A14;SLC2A3	21.44491	NaN	21.6511	0.14579835	+		3	1	2	1.03E-08	52355000	9	11.4	44.891	
SLC30A1	23.6572	23.91114	23.27085	0.32241876	+		10	8	9	2.42E-49	3.63E+08	36	19.7	55.299	
SLC30A6	NaN	20.41792	NaN	N/A	+		1	2	1	2.61E-05	22853000	4	5.9	42.509	
SLC35B2	25.82529	25.81409	25.87617	0.0330861			11	12	9	1.41E-51	1.81E+09	159	33.7	42.127	
SLC35C1	NaN	21.04953	20.99113	0.04129504			2	3	2	3.47E-06	47410000	5	9.7	38.234	
SLC35E2B;SLC35E	NaN	NaN	20.17731	N/A			1	1	2	2.99E-05	17997000	5	5.4	43.777	
SLC35F6;C2orf18	NaN	NaN	21.59619	N/A	+		0	1	2	1.31E-17	67738000	7	5.7	40.214	
SLC38A1	24.05258	24.0411	23.85728	0.10959294			5	4	5	2.42E-37	4.13E+08	34	10.7	54.047	
SLC38A2	24.88651	25.20086	25.08732	0.15918119			3	4	4	3.92E-116	8.95E+08	80	17.6	56.025	
SLC39A1	NaN	NaN	19.39203	N/A	+		0	1	1	0.000187	13515000	3	5.8	21.844	
SLC39A10	21.04166	21.67068	21.49023	0.32389386			4	3	4	1.34E-08	65422000	11	4.5	94.131	
SLC3A2	21.71725	22.12266	22.06065	0.21837509			4	4	6	8.84E-24	1.36E+08	25	15.7	57.944	
SLC41A3	21.476	22.24638	22.18761	0.42882165			3	3	3	3.59E-19	1.03E+08	17	30.3	16.43	
SLC44A1	26.19197	26.33899	26.14198	0.10240998	+	+	13	14	16	5.52E-105	2.31E+09	198	28.3	73.301	
SLC44A2	22.85102	22.93751	23.07801	0.11456092	+		6	6	6	1.85E-18	2.18E+08	28	8.5	79.845	
SLC44A5	NaN	NaN	18.42337	N/A			0	1	1	0.005285	3213600	2	1.5	67.165	
SLC5A6	22.84791	22.56505	22.8814	0.17378564			3	4	4	7.31E-15	1.66E+08	21	8.5	68.641	
SLC7A1	23.90601	24.07904	23.9757	0.08705863	+		6	7	7	3.35E-89	4.67E+08	33	14.8	67.638	
SLC7A2	23.30257	23.6841	23.81206	0.26505313			8	9	9	8.12E-34	2.85E+08	22	16.1	71.672	
SLC7A5	22.47437	22.18208	22.08197	0.20389263			5	5	4	4.19E-17	1.49E+08	19	11.2	55.01	
SLC9A6	22.0911	22.33282	21.85403	0.23939876			5	4	5	4.50E-22	1.04E+08	16	12.3	72.259	
SMS	22.02347	21.89427	22.14868	0.12721021			4	4	4	2.30E-10	1.38E+08	20	14.7	35.278	
SNAP23	25.54038	26.21971	25.63702	0.36750423			13	13	13	3.56E-114	1.99E+09	175	76.3	23.354	
SND1	NaN	NaN	19.74478	N/A			3	2	3	1.35E-11	24508000	10	6.7	102	
SNED1	NaN	NaN	23.61336	N/A			1	1	1	0.006907	6.58E+08	17	0.5	145.3	
SNRNP200	NaN	NaN	23.92588	N/A			1	1	1	0.002808	1.27E+09	14	0.7	244.5	
SNRNP70	22.0804	23.16375	22.6623	0.54217263			3	4	2	3.76E-20	2.42E+08	22	29.7	51.556	
SNRPD2	NaN	NaN	21.44263	N/A			3	1	2	2.73E-09	58707000	10	31.4	13.527	
SNRPD3	NaN	NaN	19.9137	N/A			0	1	1	8.93E-06	11554000	3	15.8	13.291	
SNRPN;SNRPB	20.15808	20.49931	20.72933	0.28742352			2	2	2	0.000197	41760000	10	8.9	17.546	
SNX1;SNX2	NaN	NaN	19.81447	N/A			1	1	2	0.000132	23513000	5	6.9	35.592	
SORD	18.93143	NaN	NaN	N/A			1	0	0	0.005495	12758000	4	2.7	36.229	
SORT1	21.23643	20.45453	20.78748	0.39238149			5	4	4	7.07E-15	51099000	15	8.1	92.067	
SPCS3	NaN	NaN	18.77738	N/A			1	1	1	0.008234	14453000	1	5	20.313	
SPECC1	NaN	20.84138	21.14152	0.21223103	+		2	4	3	7.44E-09	68971000	8	5.5	79.014	
SPPL2A	21.42859	21.51379	21.26888	0.12432969	+		3	2	3	2.35E-08	79628000	16	6.5	58.143	
SPPL3	20.5861	19.91244	NaN	0.47634955			2	2	2	2.28E-08	21321000	3	24.9	18.478	
SPRY1	NaN	NaN	21.30352	N/A			1	2	3	9.18E-09	53102000	5	22	22.882	
SPRY2	19.57456	19.78093	19.91253	0.17035789			2	2	2	4.22E-06	13509000	3	7.9	34.688	
SPRY4	NaN	NaN	19.21409	N/A			1	1	1	0.007715	11981000	3	11.3	11.482	
SPRYD7	23.25393	23.27838	23.04891	0.12602082			6	8	6	2.07E-33	2.94E+08	38	45.9	21.666	
SRC	27.10169	27.08162	27.11595	0.01724675	+		23	23	21	2.08E-186	3.11E+09	243	51.5	59.834	
SRI	20.57653	20.72217	20.24656	0.24368562			2	3	2	1.09E-06	49049000	8	19.4	17.605	
SRM	NaN	19.80521	NaN	N/A	+		1	2	1	6.71E-07	15213000	5	10.3	33.824	
SRP68	21.04647	21.07998	20.9047	0.09304545	+		4	4	4	1.48E-10	70576000	9	8.2	60.284	
SRP72	18.49654	NaN	NaN	N/A			1	0	0	0.000109	15788000	2	3.1	67.879	
SRPRB	NaN	NaN	19.17957	N/A			1	1	1	0.007938	13979000	3	5	17.502	
SRRM1	NaN	NaN	17.91041	N/A			0	1	1	0.000874	25676000	13	1.5	78.139	
SRRM2	29.20137	29.19112	29.2548	0.03419301			68	72	69	0	3.55E+10	2305	39.3	299.61	

SRSF1	NaN	18.94069	NaN	N/A			1	1	1	0.004223	21609000	6	7	16.34	
SRSF11	21.78505	20.66648	21.80018	0.65021835			3	2	2	2.51E-08	95271000	13	10	42.316	
SSB	NaN	NaN	19.96141	N/A			1	1	1	0.000124	36245000	5	2.7	46.836	
ST13;ST13P5;ST13	23.01177	22.92976	22.88211	0.0655844	+		6	7	7	6.50E-32	3.07E+08	44	19.5	41.331	
STARD3NL	NaN	21.8842	21.86729	0.01195718			1	2	2	3.26E-07	80567000	13	11.2	23.505	
STBD1	21.27304	21.49892	21.59755	0.16636125	+		3	4	3	9.06E-14	70906000	12	16.8	39.007	
STEAP3	NaN	21.01849	NaN	N/A			1	2	2	1.33E-06	27516000	2	6.8	54.471	
STIP1	24.25961	24.36966	24.27128	0.06045083			14	15	13	2.83E-57	6.44E+08	74	30.9	62.639	
STOM	24.98094	24.81411	25.05627	0.12392762			8	10	7	3.73E-83	7.8E+08	70	57.3	31.73	
STRAP	21.26408	21.35762	21.55262	0.14721303			5	4	3	5.78E-13	78375000	12	16.3	38.438	
STT3A	22.83316	22.57471	22.62357	0.13730239			6	7	7	2.44E-24	2.45E+08	32	10.4	80.529	
STT3B	NaN	NaN	21.03329	N/A			2	1	3	5.93E-08	23364000	3	4.1	93.673	
STX10	20.83245	20.95042	21.14638	0.15857138			2	3	3	4.33E-13	45904000	9	70.7	6.3891	
STX11	NaN	18.67038	NaN	N/A			1	1	0	0.002601	3606200	1	3.1	33.195	
STX12	24.62545	24.40957	24.43199	0.11869681			6	6	6	1.30E-55	6.07E+08	74	35.5	31.642	
STX4	NaN	20.03147	NaN	N/A	+		1	2	1	7.74E-05	19896000	2	6.8	33.84	
STX6	21.2798	21.31518	22.09465	0.46058039			3	4	5	1.11E-22	68112000	12	25.1	29.176	
STX7	25.27128	25.24474	25.38739	0.07586713			7	8	6	5.92E-173	1.06E+09	101	46.9	27.4	
STX8	23.56162	23.09897	23.16246	0.25080029			6	8	6	6.42E-28	3E+08	43	45.3	26.906	
SUCLA2	NaN	19.44821	19.15571	0.20682873			0	2	2	8.86E-05	11721000	2	4.7	43.841	
SUMO2;SUMO4;S	NaN	NaN	20.73132	N/A			1	1	1	2.71E-06	83923000	11	16.9	8.1111	
SURF4	23.63699	23.76294	23.47184	0.14598923	+		4	5	5	4.72E-28	4.23E+08	97	39.8	21.127	
SVIP	24.12274	24.70556	24.38265	0.29197695	+		2	2	2	8.99E-31	6.4E+08	63	32.5	8.4426	
SYNCRIP;HNRNPR	NaN	20.69362	20.39812	0.20895005			3	3	3	7.30E-08	31316000	9	6.8	58.735	
SYNGR2	22.17697	22.5595	22.70303	0.27192805			3	4	4	1.06E-10	2.24E+08	27	16.1	24.81	
SYPL1	NaN	NaN	20.52936	N/A			1	1	1	4.58E-05	21166000	5	4.9	24.784	
SYT1	NaN	18.86233	NaN	N/A			1	1	0	0.004705	11150000	2	3.1	47.26	
TAGLN2	25.1388	25.11331	24.96965	0.09119545			11	11	12	3.16E-65	1.47E+09	145	80.4	22.391	
TALDO1	24.2863	24.31638	24.34503	0.0293679			8	8	8	1.04E-38	6.09E+08	81	35	37.54	
TARS	22.72059	23.00304	22.58813	0.21192529			8	10	10	1.24E-26	2.12E+08	45	14.7	83.434	
TBCB;CKAP1	NaN	NaN	18.52252	N/A			1	1	2	2.92E-06	5440900	3	13	19.251	
TCEB1	NaN	18.57171	NaN	N/A			2	1	2	1.75E-08	22664000	9	55.4	6.968	
TCP1	24.53908	24.44436	24.45092	0.0528947			13	15	13	1.25E-68	6.05E+08	99	50.2	60.343	
TEAD1	19.65681	NaN	NaN	N/A			1	1	0	0.000994	3288400	2	3	37.081	
TECR	22.19448	21.61926	21.74119	0.30309976	+		2	2	3	4.07E-10	1.03E+08	10	11.7	36.034	
TELO2	19.55704	19.63701	19.56815	0.04332115			3	2	2	7.29E-08	18633000	5	5.4	91.746	
TESC	22.44362	22.24007	22.51106	0.14107701	+		5	5	5	1.32E-21	1.42E+08	21	34.8	21.521	
TFAM	NaN	NaN	19.7268	N/A			1	1	1	0.000815	22386000	5	4.6	25.567	
TFRC	27.57391	27.46848	27.41869	0.07925463			22	27	30	1.90E-297	5.38E+09	446	51.3	84.87	
TIAM1	NaN	NaN	18.74619	N/A	+		0	0	1	0.000704	1875100	1	6.2	24.957	
TIMM23;TIMM23I	NaN	NaN	19.10303	N/A			0	1	1	0.001122	9262800	3	4.8	21.943	
TKT	24.08286	24.03633	24.28968	0.13486152			11	11	11	3.65E-33	5.03E+08	50	26.2	62.878	
TLDC1	24.75649	24.81519	25.09204	0.17920445	+		11	9	10	5.97E-70	7.67E+08	92	36.4	50.993	
TLN1	21.4247	21.08654	20.76822	0.32828996			5	6	6	2.00E-13	76736000	11	3.7	258.08	
TM9SF2	21.5733	22.33674	22.09819	0.39056716			3	3	3	6.82E-12	1.14E+08	9	6.3	75.775	
TM9SF3	22.38814	22.48827	22.77519	0.20089666			3	3	4	1.12E-13	1.66E+08	27	8.3	67.887	
TM9SF4	21.42378	21.618	NaN	0.13733428			3	3	1	1.53E-11	51369000	9	7.6	74.518	
TMBIM1	NaN	NaN	19.7055	N/A			1	1	1	1.16E-06	26990000	7	13.2	11.535	
TMED1	NaN	20.46334	20.72783	0.18702267			1	2	2	4.19E-06	18775000	7	22	9.6352	
TMED10	NaN	NaN	19.86618	N/A			1	1	1	4.11E-05	25193000	5	7.2	16.904	

TMEM106B	25.03813	25.27991	25.08898	0.12747389	+			8	8	8	5.78E-78	1.07E+09	78	36.9	31.127
TMEM106C	22.93927	22.70599	22.69434	0.13817018	+	+		3	3	3	1.97E-19	1.78E+08	20	14.4	27.875
TMEM115	22.00792	21.61836	21.61441	0.22606147				4	4	4	7.90E-18	1.06E+08	12	14.2	38.197
TMEM167A	NaN	18.92039	NaN	N/A				1	1	0	0.004924	8370700	3	12.5	8.0598
TMEM168	23.308	23.05127	23.1869	0.12843351				9	7	8	2.84E-33	2.24E+08	29	15.5	79.754
TMEM179B	22.69914	23.10796	23.03366	0.21777585				3	2	3	1.01E-15	2.7E+08	32	15.5	23.55
TMEM181	21.98794	22.32257	22.59862	0.30580792	+			3	4	4	8.67E-13	1.33E+08	13	6.5	69.324
TMEM184B	NaN	NaN	20.62756	N/A				1	1	2	1.31E-05	20337000	5	4.7	45.561
TMEM184C	22.89041	22.69164	22.73783	0.10402218				2	2	2	7.65E-76	2.24E+08	13	9.6	50.141
TMEM192	21.96582	21.42967	21.97344	0.31176933				5	4	3	4.37E-16	69731000	12	21.7	30.549
TMEM205	NaN	NaN	19.95262	N/A	+			1	1	1	2.70E-05	22340000	6	14.2	13.389
TMEM219	21.20819	NaN	NaN	N/A	+			2	2	1	2.29E-06	30991000	8	13	24.698
TMEM222	22.29454	21.67805	21.51821	0.40993841				3	3	3	1.01E-12	70079000	9	24.6	19.862
TMEM245	NaN	19.77372	19.70317	0.04988638				1	2	2	2.76E-06	23194000	9	5.4	55.418
TMEM33	22.72798	22.66123	22.8032	0.0710271				6	6	5	1.04E-23	2.05E+08	41	23.5	27.978
TMEM50A	23.22159	23.36266	23.29123	0.07053689				4	4	4	5.78E-18	2.8E+08	32	29.3	17.4
TMEM50B	NaN	NaN	21.82361	N/A				1	1	2	1.31E-08	93251000	14	15.2	16.854
TMEM55A	21.0794	21.41936	21.22335	0.17064306				2	3	3	1.33E-07	65789000	10	11.7	28.081
TMEM55B	23.26499	23.45801	23.54097	0.1416004	+			5	5	6	2.44E-23	3.01E+08	36	26	29.469
TMEM63A	21.52874	22.0309	22.31647	0.39879682				5	4	3	4.58E-14	1.03E+08	16	6.3	92.125
TMEM63B	23.72411	23.8149	23.76	0.0457255				8	9	8	9.97E-31	3.78E+08	50	11.7	94.957
TMEM65	NaN	NaN	20.07601	N/A				1	0	2	1.81E-05	17253000	3	8.8	25.498
TMEM87A	22.12386	22.22285	22.047	0.08815678				5	4	5	1.28E-19	1.19E+08	14	12.8	56.773
TMPO	NaN	20.57385	NaN	N/A				1	2	2	0.000105	30332000	6	8.9	26.866
TMX1	25.70076	25.67673	25.49902	0.11019476				11	14	13	2.61E-59	1.35E+09	138	32.9	31.791
TMX3	22.49059	22.66451	22.40049	0.13420925				5	5	4	2.42E-18	1.65E+08	30	18.1	51.871
TMX4	21.72763	21.58939	21.97767	0.19680433				3	2	2	3.34E-12	1.01E+08	17	13.5	38.952
TNFRSF10A	24.75511	24.21297	24.30883	0.2893299				7	8	8	6.36E-32	5.39E+08	62	19.2	50.089
TNFRSF10B	20.68123	20.30218	NaN	0.26802883				2	2	1	3.11E-05	21496000	4	9.2	28.674
TNPO1	21.95748	22.15054	22.33475	0.1886523				7	10	8	1.34E-30	1.89E+08	41	15.6	101.31
TOMM40	24.09748	23.53457	23.42829	0.35962443	+			3	4	4	2.17E-68	3.4E+08	26	16.6	37.893
TOMM40L	NaN	NaN	19.70892	N/A	+			1	1	1	0.002211	15736000	3	2.9	33.916
TOMM70A	20.38343	20.58939	NaN	0.14563571				2	2	1	5.92E-06	39208000	5	3	67.454
TPBG	NaN	NaN	20.62444	N/A				1	2	2	1.20E-05	51002000	2	4.3	46.031
TPD52L2	21.3819	21.47615	21.49438	0.0603699				2	4	4	2.31E-15	92947000	19	25.2	22.237
TPI1	27.28965	27.13975	27.08841	0.10456525				15	16	16	0	5.69E+09	383	85.9	26.669
TPT1	NaN	22.21294	21.32378	0.62873107	+			0	2	3	3.17E-17	1.15E+08	14	25.6	19.595
TRAM1	NaN	19.64953	NaN	N/A				2	2	1	1.18E-05	9580500	2	7.3	33.435
TRAP1	23.93553	23.87098	23.74109	0.09903285				12	13	12	2.70E-72	4.74E+08	68	24.9	74.267
TRAPPC3	25.1372	25.27512	25.31164	0.09200086	+			6	6	6	2.21E-32	9.66E+08	75	41	15.005
TRIM23	NaN	NaN	23.11978	N/A				1	1	1	0.000171	2.48E+08	31	1.8	61.068
TRIM28	22.56911	22.56714	22.38916	0.10333019				9	8	8	3.54E-41	1.88E+08	37	12.6	88.549
TRIP13	20.52698	20.48424	20.71984	0.12551829				2	2	2	1.04E-05	25313000	9	4.2	48.55
TROVE2	NaN	20.16325	NaN	N/A				1	2	1	6.60E-07	9638800	2	3.7	58.482
TSN	20.88204	20.44779	20.80302	0.23130268				2	2	2	3.71E-07	53435000	12	13.5	21.01
TSPAN13	22.87513	23.24394	23.04555	0.18458168				4	4	5	8.27E-20	2.46E+08	28	26	22.147
TSPAN14	22.43652	22.54083	22.43092	0.06190334				3	3	4	1.18E-11	1.52E+08	20	14.6	28.876
TSPAN15	NaN	NaN	21.76154	N/A				2	2	3	2.00E-07	64881000	6	20	18.219
TSPAN18	NaN	NaN	20.17158	N/A				1	1	1	0.000208	37420000	4	15.9	15.97
TSPAN2	NaN	NaN	19.84443	N/A	+			1	1	1	0.000111	25470000	5	6.4	20.564

TSPAN3	27.17198	27.20686	27.18376	0.01774351	+			6	6	6	4.69E-52	4.32E+09	77	38.6	25.183	
TSPAN31	NaN	NaN	21.76418	N/A				1	1	1	1.80E-12	99217000	12	17.5	13.317	
TSPAN6	25.29404	25.826	25.56416	0.26599074				8	9	9	2.38E-64	1.77E+09	128	37.1	27.563	
TSPAN7	22.02337	21.72841	21.44304	0.29017821	+			2	2	2	1.34E-17	93774000	11	11.2	24.475	
TSPAN9	22.49714	21.51999	22.26959	0.51128943				3	2	2	5.39E-13	93473000	21	20.1	26.779	
TTYH3	23.87998	23.86212	24.04735	0.10217783				6	7	6	2.75E-24	4.84E+08	53	17.1	54.139	
TUBA1B;TUBA4A	28.32135	28.29863	28.30691	0.01149834	+			17	19	19	0	9.77E+09	672	59.9	50.151	
TUBA1C;TUBA1B	23.12978	22.91714	22.73533	0.1974257	+			2	3	3	0	1.88E+08	28	60.1	49.895	
TUBB	25.88914	25.80344	25.66289	0.11422774				5	5	5	0	1.74E+09	133	58.2	47.766	
TUBB2A	20.57773	20.42992	21.46518	0.55993728				3	3	3	0	52888000	15	47	49.906	
TUBB2B	NaN	NaN	19.17319	N/A				1	1	1	0	12931000	2	47	49.953	
TUBB3	NaN	NaN	19.34505	N/A				1	1	1	1.09E-287	7580000	2	32.4	50.432	
TUBB4B;TUBB4A	27.90696	27.62345	27.67828	0.15037663				20	20	19	0	7.07E+09	705	59.6	49.83	
TUBB8	22.16638	22.22832	22.96443	0.44395538				1	1	1	8.60E-137	1.79E+08	8	24.3	49.775	
TUFM	22.70132	22.73862	22.4328	0.16684332				5	4	7	2.31E-26	1.91E+08	34	18.6	49.541	
TUSC2	NaN	NaN	21.15555	N/A	+			1	1	2	3.03E-05	44492000	6	19.1	12.074	
TXNDC17	NaN	20.02702	20.14843	0.08584983				1	2	2	0.000105	14607000	3	15.4	13.941	
TXNDC5	20.80278	20.64421	20.59396	0.10899182				2	5	5	1.91E-12	51511000	12	16	36.177	
TYMS	NaN	19.57723	19.73907	0.11443816				1	2	2	7.72E-05	20331000	5	7.2	31.758	
TYSND1	20.76393	20.45151	20.71624	0.16830655				2	3	3	3.12E-08	25773000	6	6	59.308	
UBA1	26.02813	25.86872	25.82648	0.1063473	+			26	26	27	2.88E-151	1.91E+09	205	37.9	117.85	
UBA2	22.25275	22.01461	22.11248	0.11969744				6	5	5	3.25E-15	87773000	15	12.5	71.223	
UBB;RPS27A;UBC;	25.52979	25.79662	25.63373	0.13449593				4	4	4	4.96E-69	1.89E+09	149	50.5	10.469	
UBE2D3;UBE2D2	21.64601	22.26382	21.98169	0.30929155				2	2	2	1.77E-05	65976000	4	12.2	16.687	
UBE2I	NaN	NaN	21.54066	N/A				2	3	2	2.17E-09	54769000	11	32.9	7.8891	
UBE2K	NaN	20.21608	20.53991	0.22898239				1	2	2	3.48E-05	23733000	4	18	15.81	
UBE2L3	21.2397	20.77024	20.95027	0.23684496				2	3	3	7.75E-06	39457000	5	34.4	14.121	
UBE2M	21.42008	21.68286	21.67271	0.14887258				6	5	5	3.89E-13	1.29E+08	17	35.5	20.9	
UBE2V1;TMEM18	21.24179	20.89652	21.27384	0.20920841				3	3	4	2.16E-10	73551000	16	24.5	16.495	
UBTD1	NaN	NaN	19.43043	N/A	+			1	1	1	2.49E-22	9325600	1	16.3	25.938	
UBTD2	22.12626	22.13163	21.8858	0.14040551	+			3	3	3	7.70E-28	1.23E+08	29	24.4	26.189	
UCHL1	25.34597	25.10025	24.94002	0.20446979				7	7	7	2.42E-73	1.13E+09	117	59.6	24.824	
UMPS	NaN	NaN	19.05992	N/A				1	1	1	0.000702	9635400	4	2.9	37.64	
UNC45A	19.60257	20.16877	NaN	0.40036386				2	2	0	0.000114	10187000	2	3.1	101.67	
UQCR10	NaN	NaN	19.19439	N/A				1	1	1	0.006463	10213000	3	11.3	6.7387	
UQCRC1	21.27577	21.74131	21.38406	0.24361232				4	4	5	1.12E-13	85573000	11	15.4	52.645	
UQCRC2	NaN	NaN	19.78998	N/A				1	1	1	0.000263	28790000	6	3.9	44.634	
UQCRQ	23.91415	23.31479	23.42111	0.31979806	+			3	4	5	2.33E-24	5.59E+08	102	74.4	9.9062	
USMG5	21.3048	NaN	21.3566	0.03662813				2	1	2	1.37E-06	76824000	6	43.1	6.4575	
USP12;USP46	21.45	21.19617	20.7545	0.35195225				3	3	3	1.09E-07	56852000	8	10.5	42.857	
USP14	NaN	NaN	20.32564	N/A				2	2	2	6.12E-08	34911000	3	8.3	51.086	
USP31	NaN	NaN	23.90921	N/A				0	0	1	0.004478	4.53E+08	2	1.6	68.299	
USP32	21.0208	20.33462	21.10987	0.42422263	+			4	5	5	4.55E-12	43917000	8	3.8	181.65	
USP5	NaN	NaN	19.61101	N/A				1	2	2	5.77E-06	15531000	9	2.5	93.307	
USP7	23.86117	23.5412	23.85262	0.18231672				11	12	10	1.18E-32	3.38E+08	40	13.5	117	
USP9X	19.35357	19.70115	19.45385	0.17889719				2	2	2	1.67E-05	22603000	6	0.9	290.46	
VAMP3;VAMP2	25.62299	25.6193	25.55871	0.03609405				3	3	3	1.86E-157	1.28E+09	43	40	11.309	
VAMP4	NaN	NaN	19.69449	N/A				1	1	1	0.000364	22113000	6	10	16.24	
VAMP7	25.29614	25.10045	25.04226	0.13300107				9	8	8	1.13E-47	9.21E+08	82	45.5	24.935	
VANGL1	21.24348	21.5435	21.35332	0.15179221				4	5	5	6.72E-13	69084000	7	12.3	59.747	

VANGL2	NaN	NaN	18.90107	N/A				0	0	1	0.000163	7397500	2	2.3	59.714	
VARS	18.67855	18.53932	18.6225	0.07005416				2	2	2	5.02E-06	9281200	4	2	140.47	
VAT1	21.49692	20.98106	20.91284	0.3193522				4	4	4	3.82E-10	63337000	7	19.6	41.92	
VCL	24.43905	24.63134	24.48173	0.10097869				21	19	18	2.16E-84	7.96E+08	139	29.1	116.72	
VCP	23.15809	22.75729	22.9742	0.20062657				7	8	9	4.53E-46	2.43E+08	50	16.1	89.321	
VDAC1	22.6553	22.49018	22.38013	0.13850039				6	6	6	4.21E-21	1.92E+08	37	23	30.772	
VDAC2	26.42907	26.44142	26.40401	0.01906145				13	13	12	1.60E-124	2.61E+09	161	61.6	31.566	
VDAC3	23.33484	23.27583	23.52886	0.1323821				4	4	5	8.87E-26	2.97E+08	32	22.3	30.658	
VKORC1	NaN	NaN	20.80876	N/A	+			1	1	1	0.000146	47610000	7	14.1	9.8745	
VKORC1L1	20.11159	NaN	NaN	N/A				2	1	1	3.34E-05	12497000	2	11.9	19.835	
VPS35	20.82314	21.01917	21.50998	0.35380583				4	3	5	2.85E-13	52547000	10	6.7	91.706	
WDR1	19.66648	NaN	NaN	N/A				2	1	1	0.00055	8006600	2	3.4	50.693	
XPO1	24.78502	24.1943	24.52402	0.29602545				16	18	15	2.45E-56	7.89E+08	72	22.7	123.38	
XPO5	20.0747	20.01509	20.2362	0.11440078				3	3	3	1.23E-08	34216000	8	2.7	136.31	
XPO7	NaN	20.30408	20.16165	0.10071322				1	2	2	0.000307	21002000	4	1.9	123.91	
XPOT	20.86287	20.61342	20.67349	0.1301912				5	4	3	3.79E-12	29849000	9	5.3	109.96	
XRCC5	24.32313	23.78616	24.25861	0.29317475				12	12	12	8.52E-59	5.16E+08	64	25.3	82.704	
XRCC6	24.04568	23.88278	23.85861	0.10174788				9	8	9	1.79E-51	5.25E+08	71	29.9	69.842	
XXYLT1	20.45924	20.52898	20.32092	0.105897	+			2	2	2	2.54E-06	33447000	7	13.9	21.816	
YARS	21.7213	21.7628	21.79618	0.03751331	+			5	5	7	1.69E-18	1.02E+08	19	14.4	59.143	
YES1	23.67842	24.09764	23.90912	0.20996337	+			7	9	8	6.69E-79	4.32E+08	52	43.3	60.801	
YIPF3	NaN	NaN	19.16671	N/A				1	1	1	0.005285	7855200	1	8.3	12.29	
YIPF6	NaN	NaN	19.00758	N/A				1	1	1	0.000287	6992900	1	5.1	26.256	
YWHAB	24.58095	24.53202	24.39126	0.09847996				6	5	6	1.99E-123	1.15E+09	78	48.4	27.85	
YWHAE	26.07413	26.25443	26.17684	0.09044118				14	13	13	4.56E-90	3.48E+09	215	61.2	29.174	
YWHAG	21.40171	21.39984	21.02439	0.21730799				3	4	4	1.59E-39	1.1E+08	25	35.2	28.302	
YWHAH	21.50698	22.10466	22.23333	0.38759121	+			4	3	3	4.43E-29	2.08E+08	27	26.8	28.218	
YWHAQ	23.29306	23.44872	23.44468	0.08872709				5	5	6	1.08E-99	5.14E+08	81	45.7	27.764	
YWHAZ	25.8285	25.71544	25.6778	0.07843237				8	10	10	3.32E-163	2.28E+09	171	53.1	27.745	
ZDHHC13	24.18662	23.92922	23.89634	0.15895405				7	7	8	6.79E-36	4.11E+08	46	14.8	70.86	
ZDHHC17	21.05715	20.97869	NaN	0.0554796	+			2	2	2	5.99E-11	44400000	6	5.7	72.639	
ZDHHC18	22.10575	21.95227	21.84395	0.13154757				6	6	6	1.32E-22	1.56E+08	24	20.6	42.03	
ZDHHC20	21.74829	21.51634	21.75776	0.13673216				5	4	5	4.93E-15	82370000	14	17.3	42.277	
ZDHHC23	NaN	18.97006	NaN	N/A				1	1	0	0.00185	4323900	1	3	45.35	
ZDHHC24	NaN	20.4997	20.3024	0.13951217	+			1	2	2	3.89E-05	21688000	8	5.6	30.175	
ZDHHC3	22.2674	22.18526	22.54527	0.18866457				2	2	2	3.38E-14	1.21E+08	10	9	34.17	
ZDHHC4	21.89423	21.36062	22.00467	0.34441666				3	3	3	6.89E-07	80415000	8	11.6	39.786	
ZDHHC5	22.53709	22.60905	22.31058	0.15576123				7	7	7	1.03E-23	1.58E+08	29	14.7	71.951	
ZDHHC6	23.64306	23.44922	23.5134	0.09874608	+			7	5	7	1.94E-30	2.79E+08	35	18.8	47.205	
ZDHHC7	NaN	NaN	20.83337	N/A				1	1	1	0.006126	54905000	3	3.6	35.14	
ZMPSTE24	20.61162	21.09513	21.6611	0.52527964	+			2	2	3	2.57E-07	82939000	18	5.5	54.812	
ZNRF2	23.13632	23.34137	23.43466	0.15261896	+			3	4	4	2.14E-28	2.54E+08	27	59.5	24.115	

Reagent 3

Gene names	log2 LFQ intensity_1	log2 LFQ intensity_2	LFQ intensity_3	St dev log2 LFQ	MG protein	PTM peptide	Razor + unique peptides_1	Razor + unique peptides_2	Razor + unique peptides_3	PEP	Intensity	MS/MS Count	Sequence coverage [%]	Mol. weight [kDa]	
AARS	NaN	21.36725	21.4244	0.04041115			3	4	3	3.06E-15	77577000	13	5.9	106.81	
ABCD3	NaN	20.11908	NaN	N/A			2	3	1	1.60E-09	17398000	4	8	67.315	
ABCE1	NaN	20.02702	NaN	N/A			1	2	1	8.66E-06	15140000	3	5.2	47.037	
ABHD17A	22.8265	22.93207	22.88252	0.05281803			7	5	4	1.06E-22	2.79E+08	24	33.5	33.989	
ABHD17B	22.85028	22.78405	22.83447	0.03458937			5	6	4	7.99E-26	3.29E+08	22	34	32.214	
ABL2	23.18575	22.97761	23.159	0.11324027	+		8	7	7	1.43E-35	2.96E+08	46	12.6	115.82	
ACAA2	20.85118	20.85201	NaN	0.0005869	+		2	3	1	1.25E-15	53733000	8	14.6	41.924	
ACACA	26.73389	26.96477	26.9354	0.12568111			51	50	47	5.68E-188	2.45E+09	314	31	265.55	
ACAD9	20.22376	20.30497	20.56393	0.17765649			3	3	2	2.10E-08	36352000	6	6.1	68.76	
ACAT1	21.14694	21.24632	21.02803	0.10929051			2	4	2	3.69E-18	87102000	17	21.8	45.199	
ACLY	21.69897	21.60001	NaN	0.06997529			6	4	2	1.93E-18	76021000	16	8.6	119.77	
ACOT7	21.24278	21.22888	21.23204	0.00728633			3	3	3	4.67E-09	56625000	11	14.2	27.041	
ACOT9	23.55789	23.58302	23.32882	0.14007273			8	7	5	4.59E-33	3.51E+08	54	31.3	46.354	
ACTG1;ACTB;ACT/	28.49045	28.43595	28.42182	0.0362399			18	18	18	0	1.28E+10	601	67.5	41.792	
ACTN4;ACTN1	21.07496	21.34592	20.97463	0.19206843	+		5	4	3	5.49E-17	75173000	11	9.9	104.85	
ACTR1A;ACTR1B	NaN	21.96797	NaN	N/A			1	2	1	5.02E-06	31431000	1	9.9	34.557	
AGPAT1	21.84625	21.31401	22.02398	0.3694421			4	4	3	4.89E-11	1.12E+08	30	35.1	18.756	
AHCY	24.87769	24.91022	24.97176	0.04777471			12	13	9	3.52E-54	9E+08	113	34.3	47.716	
AIFM2	24.51527	24.64091	24.64108	0.07258741	+		9	8	8	1.32E-58	7.66E+08	64	44.5	40.526	
AKAP12	27.35094	27.35892	26.83219	0.30183045	+	+	35	36	29	0	4.84E+09	455	37.4	191.48	
AKR1B1	21.40769	21.35719	21.48331	0.06347557			4	3	4	3.17E-08	93513000	7	11.1	35.853	
ALDH18A1	22.65003	22.77983	22.96888	0.16033988			8	9	8	5.75E-27	2.24E+08	48	14.9	87.088	
ALDH5A1	20.76223	20.97771	20.69277	0.14857497			2	2	2	4.75E-16	45353000	13	9.9	57.214	
ALDH7A1	22.7136	22.52691	22.88049	0.17688237	+		7	7	6	1.96E-21	2.11E+08	19	21.5	55.366	
ALDH9A1	21.39367	21.49106	21.70261	0.15794627			5	5	5	1.39E-15	91824000	15	13.2	53.801	
ALDOA	26.63492	26.66112	26.30226	0.200054			16	17	15	5.06E-187	3.01E+09	221	68.4	39.42	
ALDOC	23.13877	22.36054	23.01362	0.41789511			1	1	1	5.87E-18	2.18E+08	13	8.2	39.455	
ALG6	20.92781	21.64121	21.0424	0.38311092			2	2	2	2.23E-07	65478000	7	5.5	58.18	
ANKS3	NaN	25.34123	NaN	N/A			0	1	0	3.13E-07	3.93E+08	1	4.4	58.028	
ANO6	21.5382	21.57538	21.43756	0.07130348			6	7	6	6.48E-20	1.17E+08	21	11.8	103.96	
ANP32A	22.59785	22.51191	22.28354	0.16244451			4	4	4	3.14E-20	1.67E+08	16	29.4	19.997	
ANP32B	21.3068	21.91885	21.81249	0.32701683			2	2	2	5.50E-16	85008000	10	17.9	22.276	
ANXA1	24.31119	24.15499	24.01979	0.14582606			11	11	9	5.45E-105	6.1E+08	89	54.3	38.714	
ANXA11	22.17646	21.83653	21.77672	0.21560833			4	4	4	2.02E-11	1.09E+08	26	8.5	51.242	
ANXA2;ANXA2P2	26.6064	26.34875	26.73169	0.19524521	+		16	17	17	6.34E-165	2.65E+09	241	64.3	38.604	
ANXA4	20.03026	20.1756	NaN	0.1027709			2	2	1	2.23E-05	18401000	2	8.4	27.062	
ANXA5	27.72247	27.81268	27.66614	0.07391987			20	21	20	4.45E-256	6.82E+09	647	76.6	35.936	
ANXA6	24.60818	24.50015	24.82862	0.16741008			14	16	9	2.41E-58	6.57E+08	106	42	75.276	
ANXA7	21.61464	21.46613	21.59459	0.08058039			4	2	3	8.89E-12	79585000	13	18.5	37.805	
AP1B1	22.53443	22.55366	22.49465	0.03009546			6	6	7	2.23E-21	1.48E+08	32	10.9	101.35	
API5	23.18414	23.13025	23.21693	0.04376593			7	7	6	4.29E-71	2.21E+08	36	21.8	50.309	
APMAP	22.54776	22.20654	22.47117	0.17903731			6	5	5	1.73E-21	2.41E+08	24	18.3	46.48	
APRT	23.43224	23.18001	23.42458	0.14346494			4	4	3	6.05E-23	3.19E+08	45	49.4	19.608	
ARCN1	20.18882	20.28059	20.47684	0.14713447			2	4	2	6.23E-10	44299000	10	8	57.21	
ARF1	30.49686	30.49543	30.7285	0.13415213	+	+	13	13	12	0	6.23E+10	761	79.6	20.697	
ARF3	16.90971	17.4754	NaN	0.40000324	+		1	1	1	0	1.97E+08	22	64.6	20.601	

ARF4	27.40471	27.34359	27.73635	0.21133747	+		7	7	7	0	7.01E+09	255	86.7	20.511	
ARF5	26.26462	25.89264	26.3585	0.24637644	+		6	6	6	0	2.77E+09	142	71.1	20.529	
ARF6	25.82583	25.6002	25.8704	0.14485822	+		7	6	4	2.11E-78	2.14E+09	117	60	20.082	
ARHGDIA	21.6522	NaN	NaN	N/A			2	1	1	3.77E-12	92974000	10	23.3	21.517	
ARL1	26.69073	26.59449	26.63755	0.0482086	+		10	10	10	0	4.38E+09	238	80.5	18.565	
ARL13B	20.39613	20.44748	NaN	0.03630993			2	2	1	2.82E-06	42032000	11	8.3	37.086	
ARL4C;ARL7	22.40052	NaN	21.7942	0.42873298	+		2	1	2	1.02E-07	1.17E+08	8	16.1	21.487	
ARL5A	19.09084	19.10457	NaN	0.00970858	+		2	2	1	1.15E-21	21895000	8	25.7	20.728	
ARL5B	22.48778	22.86859	22.94972	0.24663972	+		3	4	4	2.83E-21	2.38E+08	23	21.2	20.374	
ATAD3B;ATAD3A;	21.81468	21.21502	20.86816	0.47885351			4	3	3	8.46E-09	46550000	10	8.1	67.608	
ATIC	21.61544	NaN	21.84295	0.16087386			3	2	3	1.03E-21	94879000	16	14	64.523	
ATP11B	21.98808	21.80069	22.0693	0.13775766			6	6	6	4.17E-22	1.1E+08	16	8.1	134.19	
ATP13A3	20.98663	NaN	NaN	N/A			2	1	1	5.64E-07	33471000	4	2.8	138.04	
ATP1A1	24.82372	24.66373	24.65168	0.09603798	+		17	17	14	3.17E-92	8.85E+08	116	24.8	113	
ATP2A2	24.00342	23.78875	23.43415	0.28748701	+		12	13	12	1.20E-60	4.56E+08	66	21	114.76	
ATP5A1	23.50676	23.62956	23.29011	0.17187368			10	11	6	7.15E-77	7.08E+08	92	40.7	59.75	
ATP5B	NaN	21.07477	NaN	N/A			2	4	3	1.30E-18	1.68E+08	15	24.6	38.138	
ATP5H	21.9226	21.89115	22.35665	0.2601534			4	6	5	2.04E-20	2.06E+08	18	74.5	15.773	
ATP5O	24.488	24.08245	24.32038	0.20378827			6	6	4	2.48E-24	3.62E+08	48	34.7	23.277	
ATP6V0A2	21.12048	21.1384	21.19653	0.03975723	+		4	3	3	3.84E-20	80622000	8	9.2	98.081	
ATP6V1A	20.45312	20.58564	20.82221	0.18697341			4	5	2	1.00E-20	61686000	12	8.9	64.735	
ATP6V1B2	20.0474	NaN	NaN	N/A			2	1	1	2.47E-06	19877000	5	5.1	56.5	
ATP9A	23.34123	23.01732	23.55368	0.2701033			9	7	8	8.96E-41	3.19E+08	59	12.7	118.58	
ATXN10	20.64059	NaN	19.93805	0.4967708			2	2	2	5.06E-09	37702000	7	13.6	46.286	
B3GNT1	22.68185	22.8675	22.22126	0.33272491			7	7	5	8.47E-30	2.39E+08	42	33.3	47.119	
B4GALT3	NaN	NaN	19.11383	N/A			0	0	1	0.001285	4268500	1	3.8	43.927	
BAG5	23.17824	23.11119	22.76737	0.22042461			7	5	5	3.83E-39	2.43E+08	31	33.3	51.199	
BASP1	22.5383	21.98384	20.59295	1.00219655	+		4	6	5	2.37E-200	1.93E+09	95	57.3	22.693	
BAX	22.47929	22.17439	22.21892	0.16469136			4	4	4	2.50E-44	1.69E+08	36	36	18.129	
BCAM	22.18915	22.31088	22.3684	0.09152168			7	7	5	1.08E-32	1.9E+08	33	21	67.404	
BCAP31	22.18568	22.13693	21.86624	0.17209084	+		3	3	3	5.54E-18	2.15E+08	35	19.5	27.991	
BET1;DKFZp781C(	NaN	20.0099	NaN	N/A			1	1	0	2.61E-16	36720000	9	24.6	13.289	
BET1L	21.70755	21.69136	21.66965	0.01901688			2	3	3	6.35E-10	92362000	11	48.3	6.4651	
BZW1	20.96219	NaN	21.22764	0.1877015			3	2	2	4.91E-10	61522000	18	10.2	40.538	
BZW2	20.51999	20.71507	20.80554	0.14593368			3	3	2	9.60E-08	37898000	9	9.3	39.445	
C11orf48	23.95987	NaN	22.80599	0.81591637	+		2	1	2	6.25E-11	2.61E+08	20	21.2	11.6	
C12orf23	24.08829	23.58623	23.74632	0.256462			5	5	4	2.14E-28	4.19E+08	61	62.9	11.748	
C1QBP	21.85977	21.95472	21.76979	0.09247613			3	3	2	2.97E-23	1.14E+08	13	26.2	31.362	
C8orf47	21.48989	21.58623	21.72671	0.11909362	+		4	3	2	7.76E-16	94151000	20	21.7	39.935	
C9orf123	24.27021	24.37729	23.2158	0.64191194	+		2	2	3	3.61E-57	6.18E+08	36	50.9	11.829	
CACNA2D1	20.00758	NaN	NaN	N/A			3	2	1	1.04E-05	20230000	2	2.9	121.89	
CALHM2	20.81816	NaN	20.60937	0.14763682			3	3	3	6.12E-10	51699000	6	11.1	36.174	
CALR	22.03265	21.06118	NaN	0.68693302			2	2	0	6.29E-16	81862000	10	12.9	48.141	
CAND1	24.94985	24.86547	24.96433	0.05339			23	28	20	2.82E-106	8.89E+08	132	31.7	136.37	
CANX	28.80953	28.94748	28.66041	0.14357121			23	21	21	0	1.84E+10	767	44.9	67.567	
CAP1	21.21508	21.50412	21.78704	0.28598546			4	5	3	9.88E-15	1.11E+08	18	13.7	51.83	
CAPN1	19.69941	19.63506	19.60223	0.04943461			2	2	2	7.81E-05	20912000	5	2.5	81.889	
CAPNS1;CAPNS2	NaN	21.50062	21.36041	0.09914344	+		1	2	2	2.54E-10	78085000	15	37	11.399	
CAPZB	22.40754	22.66824	22.58594	0.13326936			5	4	5	2.22E-13	1.62E+08	25	25.4	29.295	
CAV1	24.1277	24.44164	23.15971	0.66819502			4	6	5	1.42E-67	5.71E+08	75	47.2	20.471	

CAV2	19.78711	19.96508	NaN	0.12584379	+		2	2	0	8.33E-08	55929000	11	18.8	16.828	
CBR1	20.2143	20.11248	20.13458	0.05355838			2	2	1	1.04E-09	40273000	8	23.1	18.762	
CCNY	23.88017	23.79531	24.15592	0.18853773	+		8	7	8	1.95E-44	5.2E+08	52	47.5	39.336	
CCNYL1	NaN	21.76138	21.44733	0.22206688	+		3	4	3	2.56E-33	98915000	19	36.2	40.705	
CCT2	24.36312	24.24496	24.45689	0.10619865			12	14	8	4.36E-88	6.61E+08	84	42.1	57.488	
CCT3	24.09241	24.37465	24.14102	0.15088921			13	13	12	7.39E-80	6.01E+08	89	40	55.674	
CCT4	24.62249	24.60399	24.4693	0.08361701			18	17	13	2.70E-75	7.68E+08	105	55.8	57.924	
CCT5	23.16537	23.42522	22.84692	0.28964441			8	8	6	5.33E-38	2.72E+08	48	22.6	59.67	
CCT6A	23.85852	23.92107	23.78586	0.06766797			12	14	10	5.77E-76	4.41E+08	63	29.6	58.024	
CCT7	23.98311	23.90426	23.94163	0.03944285			11	11	10	4.56E-49	4.72E+08	57	33.1	54.804	
CCT8	25.33269	25.24981	25.20486	0.06484611			21	20	17	6.05E-87	1.16E+09	182	46.5	59.62	
CD151	26.64389	26.85828	26.46627	0.1962922	+		4	5	4	2.39E-50	3.56E+09	181	15.1	28.067	
CD276	25.83853	25.46423	25.6958	0.18889901			9	8	8	2.83E-258	1.83E+09	130	37	57.165	
CD44	24.82624	24.56314	24.25523	0.28579789			4	6	6	4.97E-34	7.63E+08	94	33.5	22.683	
CD47	NaN	20.93222	21.14115	0.14773582			1	2	2	7.11E-07	55427000	10	9.6	31.742	
CD55	19.54442	NaN	19.61705	0.05135717			2	1	2	0.000402	19435000	4	5.2	35.695	
CD63	27.42822	27.36661	27.61182	0.1275622			7	7	7	4.99E-212	5.65E+09	157	33.5	23.43	
CD81	28.60498	28.46342	28.59477	0.07894756	+		4	4	4	0	1.21E+10	235	50.3	17.963	
CD9	25.90649	26.5204	25.8122	0.38456108			5	5	6	4.90E-220	2.91E+09	196	42.8	17.764	
CDC37	22.41478	22.01223	22.47415	0.25131036			4	3	4	5.76E-15	1.53E+08	26	15.3	44.468	
CDC42	21.07901	21.18683	21.9365	0.46706876			3	2	2	6.27E-09	67238000	14	25.7	21.258	
CDCA3	24.4443	24.71675	24.79853	0.18547051	+		9	11	8	3.67E-87	9.61E+08	98	52.2	28.998	
CDK1	23.0429	23.24128	22.94508	0.1509182	+		4	3	2	2.39E-13	3.91E+08	33	26.5	21.738	
CECR5	21.36169	21.08939	21.2543	0.13715879			3	5	3	7.30E-13	82416000	13	13.5	43.588	
CERS2	NaN	20.58564	19.92606	0.46639349			1	2	3	2.17E-11	77216000	9	13.8	36.373	
CFL1	24.57756	25.11977	24.36045	0.39108722			6	7	7	1.87E-62	8.73E+08	67	69.1	16.811	
CHCHD3	27.19376	27.68966	27.57636	0.25985139	+		12	12	12	1.22E-222	5.78E+09	281	46.7	26.152	
CHCHD6	24.5357	24.44006	24.66633	0.113585	+		7	5	7	3.85E-134	8E+08	72	44.7	26.457	
CHIC2	NaN	NaN	21.40795	N/A			2	1	3	1.32E-07	93967000	9	26.3	15.99	
CHMP6	25.47976	25.34962	25.40005	0.06561668	+		8	7	5	5.52E-146	1.43E+09	94	48.3	23.485	
CHP1	25.24245	24.94271	25.53002	0.29367601	+		9	10	9	5.54E-76	1.33E+09	131	67.2	22.456	
CKAP4	27.43365	27.4239	27.28436	0.08352043			26	25	26	0	5.99E+09	480	57.1	66.022	
CKAP5	20.63795	20.59678	20.65377	0.02941967	+		2	3	4	6.97E-11	43521000	11	2.9	218.52	
CKB	24.30709	24.02276	24.39395	0.1941521			8	9	9	7.38E-86	6.36E+08	80	52	42.644	
CLCC1	21.20932	21.25862	21.03772	0.11595535			3	2	3	1.32E-15	62104000	12	13.2	56.266	
CLIC1	23.9917	23.98024	24.33845	0.20358508			7	6	6	4.85E-41	5.76E+08	69	53.1	26.922	
CLIC4	21.20717	NaN	NaN	N/A			3	1	1	1.48E-07	42129000	7	16.6	28.772	
CLN3	21.61509	22.01998	21.51538	0.26723871	+		4	4	4	2.72E-24	1.31E+08	29	21.5	34.568	
CLTA	21.0351	NaN	21.12022	0.06018893			2	1	2	5.26E-06	74539000	19	7.8	23.662	
CLTC	23.90903	23.90591	23.72328	0.10635359			13	15	10	1.29E-71	4.23E+08	98	15.8	187.89	
CNN3	21.09764	21.43771	20.99576	0.23142563			2	2	2	3.75E-11	72237000	18	16.3	31.38	
CNP	19.16121	NaN	NaN	N/A			2	1	0	0.001165	8208600	2	9.7	20.245	
COPA	20.72109	20.86793	NaN	0.10383156			3	5	3	3.31E-11	37450000	5	4.4	138.34	
COPB1	21.1781	21.15277	21.25632	0.05397929			4	4	3	3.85E-10	75607000	16	4.9	107.14	
COPB2	21.0428	20.92325	21.08557	0.0841321			4	3	3	4.85E-14	61662000	10	6.8	99.045	
COPG1	21.47931	20.73032	21.60155	0.47169382			2	3	3	8.03E-09	72398000	12	4.9	97.717	
COPS3	20.63962	21.39503	20.63121	0.43858408			2	3	2	9.56E-08	55200000	9	9	39.036	
COPS4	NaN	20.52736	NaN	N/A			1	2	0	0.000301	17903000	2	5.4	40.196	
COTL1	20.08602	20.10393	20.17109	0.04484815			2	2	2	0.000375	34272000	4	12	15.945	
CPD	25.12214	25.10566	25.03095	0.04859491			18	23	16	7.36E-130	9.89E+08	146	25.1	152.93	

CPNE3	21.90591	22.31788	21.87755	0.2464461				4	5	5	9.04E-14	1.27E+08	22	11.9	60.13	
CS	24.26549	24.06833	24.24598	0.10863719				6	7	6	3.26E-35	6.56E+08	63	24.5	50.431	
CSE1L	25.7474	25.82114	25.60436	0.11022069				21	17	18	5.92E-151	1.44E+09	186	35.7	107.78	
CTBP1;CTBP2	20.92361	20.82873	NaN	0.06709029	+			3	3	1	1.52E-07	34808000	7	15.8	18.623	
CTNNA1	20.20658	20.51634	20.47348	0.16784112				2	2	2	1.59E-07	30602000	5	5.4	88.676	
CTPS1	21.0711	20.96522	NaN	0.07486847				3	3	2	1.81E-13	54596000	9	9.8	66.69	
CXADR	24.49112	24.08651	24.43129	0.21838885				8	11	11	6.57E-93	7.02E+08	58	40.5	40.029	
CYB5B	22.10124	NaN	NaN	N/A				2	1	1	1.18E-20	1.48E+08	18	46.4	15.716	
CYB5D2	NaN	19.82515	NaN	N/A				1	2	1	1.90E-12	31122000	10	24.2	28.689	
CYB5R3	28.23457	28.08015	28.23434	0.08908811	+			17	19	19	0	1.22E+10	574	85.7	31.76	
CYSTM1	23.93751	23.37478	23.998	0.34368766				2	2	2	5.15E-08	4.14E+08	33	11.3	10.631	
DAD1	22.41434	22.26072	22.33211	0.07687372				2	3	2	4.48E-11	1.64E+08	21	35.3	9.5541	
DAGLB	25.127	25.06932	25.01474	0.05613713				14	13	10	1.15E-81	1.08E+09	139	24.7	73.731	
DCAF11	21.84946	21.2383	21.37268	0.3211681	+			4	3	4	2.45E-13	86374000	12	12.3	58.846	
DCUN1D3	21.05019	20.59878	NaN	0.31919507	+			2	2	1	3.21E-09	47330000	8	13.8	34.291	
DDB1	21.70544	21.12559	21.38358	0.29051068				3	3	2	2.28E-15	76194000	19	5.6	121.71	
DDX17	21.49502	21.52061	21.3374	0.09921762				5	5	5	2.65E-21	99665000	25	14	72.371	
DDX39B;DDX39A	24.14756	23.99929	24.12361	0.07959591	+			12	11	9	6.14E-49	5.33E+08	82	29.9	48.991	
DDX3X;DDX3Y	NaN	19.51414	NaN	N/A	+			2	2	2	3.84E-08	18016000	3	5.3	71.354	
DDX46	24.08034	23.78955	24.13837	0.18690537	+			12	15	12	2.70E-41	4.62E+08	87	17.4	117.36	
DEGS1	23.98181	23.84585	24.14864	0.15165704	+			3	4	3	2.35E-38	6.33E+08	55	15.8	37.866	
DEK	20.52334	NaN	NaN	N/A				2	1	1	3.78E-05	26058000	5	6.5	38.704	
DHCR7	21.55361	22.00748	22.0485	0.2746503	+			5	3	3	3.17E-16	1.4E+08	33	12	54.489	
DHX15	21.43578	21.39707	21.49194	0.04770172				4	6	4	5.54E-17	87879000	18	8.3	90.932	
DHX9	22.12978	22.13949	21.93593	0.11482507	+			5	6	5	6.18E-22	1.04E+08	21	8.3	140.96	
DIRC2	22.90146	22.84759	22.92038	0.03776755	+			2	2	2	6.06E-22	2.22E+08	16	10.5	44.145	
DLAT	NaN	20.5364	NaN	N/A				0	2	1	7.86E-06	32341000	5	4.8	57.586	
DNAJC5	25.68	26.05571	26.24273	0.286589				8	8	8	9.45E-289	3.38E+09	275	54.5	22.149	
DNAJC7	20.66014	20.60304	20.73463	0.06598623				5	4	2	7.58E-15	47893000	17	20.6	28.704	
DNM1L	21.44622	21.32449	20.9636	0.25099198				4	2	2	9.62E-12	79167000	13	6.6	78.099	
DSG2	21.08135	21.35805	20.46114	0.45928765				6	5	5	7.38E-17	87772000	17	6.6	122.29	
DSP	18.4756	17.18534	NaN	0.9123516				2	2	1	1.73E-53	1.89E+08	36	9.3	331.77	
DTYMK	20.61711	NaN	NaN	N/A				2	1	1	0.001026	29337000	3	9.4	19.368	
DUT	21.12105	20.83167	21.40847	0.28840056				4	4	3	5.14E-13	53319000	14	36	17.748	
DYM	23.67627	23.70388	23.65009	0.02689817	+			9	7	9	3.98E-34	3.99E+08	68	18.1	75.935	
EBP	20.08861	22.01764	NaN	1.36403019				2	2	1	3.35E-07	1.46E+08	21	9.6	26.352	
ECE1	20.79551	21.06927	20.63573	0.21925294				3	3	3	1.73E-07	62583000	12	3.3	85.561	
ECHS1	23.2201	23.13871	23.34571	0.10428423				5	5	5	3.25E-21	2.96E+08	41	24.8	31.387	
EEF1A1P5;EEF1A1	27.67607	27.9467	27.44324	0.25196639	+			13	13	13	2.69E-171	7.65E+09	406	42.6	50.184	
EEF1B2	23.05087	23.11834	22.78973	0.17355635	+			4	4	4	1.50E-38	2.75E+08	28	24.4	24.763	
EEF1D	22.34695	21.84069	22.12209	0.25365566				5	4	5	1.30E-24	1.67E+08	29	31.8	28.821	
EEF1G	25.20546	25.34093	25.0984	0.12154201				14	18	11	1.22E-122	1.47E+09	158	47.6	50.118	
EEF2	27.41627	27.5842	27.46389	0.08654733				32	35	33	2.09E-242	5.11E+09	440	54.8	95.337	
EEPD1	NaN	21.18876	NaN	N/A	+			2	2	1	2.83E-15	64368000	9	10.5	62.402	
EFCAB14	20.70092	NaN	21.00539	0.2152928				2	1	2	2.43E-13	38747000	8	10.7	55.031	
EFR3A	22.06651	22.33664	22.05745	0.15863972				7	7	3	1.30E-41	2.06E+08	38	21.3	92.923	
EIF2B1	21.01665	21.49804	21.49404	0.27678317				3	2	2	2.31E-13	68937000	17	17.4	33.712	
EIF2S1	20.61027	20.86884	20.66839	0.13565679				4	4	3	2.19E-10	52197000	13	14.9	36.112	
EIF2S2	21.20198	20.8946	21.32295	0.22083173				3	4	3	3.07E-09	56630000	11	10.8	38.388	
EIF2S3;EIF2S3L	21.2498	22.0158	21.06691	0.50342113				3	4	3	1.56E-16	1.11E+08	18	14	51.109	

EIF3A	21.23479	21.74841	21.74102	0.29442852			3	7	3	3.50E-24	1.03E+08	19	7.2	166.57	
EIF3C;EIF3CL	22.10479	22.20464	21.945	0.13096806			7	7	5	1.40E-25	1.44E+08	33	11.2	104.1	
EIF3D	NaN	20.56737	NaN	N/A			0	2	0	3.30E-05	14240000	2	3.8	58.14	
EIF3E	22.65101	22.77772	22.31152	0.24105716			8	9	6	1.05E-25	2.32E+08	46	21.1	52.22	
EIF3F	22.49277	22.11499	21.8395	0.32796701			6	6	4	4.00E-26	1.46E+08	26	25.2	37.563	
EIF3H;EIF3S3	21.46199	21.08388	21.22347	0.19119987			2	2	2	7.43E-10	72084000	13	10.8	39.93	
EIF3I	20.39383	20.56104	NaN	0.11823532			3	3	1	1.05E-08	29892000	4	12.6	36.501	
EIF3L	21.62484	21.68183	21.85205	0.11821385			4	4	3	3.23E-18	1.11E+08	24	14.2	66.726	
EIF3M	22.35931	22.01318	22.02995	0.19517736			3	5	2	4.22E-15	1.26E+08	22	15.5	42.502	
EIF4A1	24.96961	25.18548	24.91762	0.14203978			15	16	12	4.71E-110	1.2E+09	132	53.2	46.153	
EIF4A2	NaN	NaN	19.30581	N/A	+		0	0	1	1.19E-72	14591000	1	32.6	41.29	
EIF4A3	20.61773	20.68834	20.15734	0.28835907			2	2	3	1.00E-14	45677000	9	16.3	46.871	
EIF4G1	22.36506	22.45779	22.66314	0.15254462			8	9	8	1.14E-32	1.58E+08	31	11.1	154.8	
EIF4G2	20.35681	20.39467	NaN	0.02677106			3	3	1	2.33E-08	29369000	7	4.7	98.149	
EIF5	NaN	20.24551	NaN	N/A			1	2	1	3.15E-07	16578000	6	6.3	49.222	
EIF5A	22.51244	23.03572	23.1494	0.33972124			3	5	4	1.03E-21	2.53E+08	32	54.5	16.832	
ENDOD1	20.88725	20.50028	NaN	0.27362911	+		4	3	2	5.36E-10	40164000	7	11.8	55.016	
ENO1	26.79042	26.834	26.76066	0.03688638			14	17	15	2.03E-225	3.38E+09	290	52.1	47.168	
EPHB2;EPHA7;EPI	NaN	19.83906	NaN	N/A	+		2	2	0	2.63E-07	23842000	8	4.3	105.56	
ERGIC2	23.6381	24.19933	23.42188	0.40128101			7	7	4	3.60E-37	5.08E+08	71	28.4	42.548	
ERGIC3	23.1739	23.29165	23.3243	0.07911094			5	4	4	2.37E-42	3.93E+08	51	26.6	43.222	
ERLIN2;ERLIN1	NaN	18.91523	NaN	N/A			2	2	1	0.001193	9476600	2	9.6	22.315	
ESD	21.50116	21.97358	20.80971	0.58535988			2	3	2	8.50E-13	96215000	14	25.9	31.462	
ETF1	NaN	20.51046	NaN	N/A			1	3	2	1.62E-07	39365000	6	10.4	45.462	
ETFB	20.90845	NaN	21.0023	0.06636197			4	2	4	5.55E-12	50676000	8	18	27.843	
EXTL2	21.70498	NaN	NaN	N/A	+		2	0	0	3.14E-08	61811000	4	12	35.967	
FAM129A	23.08809	23.08503	23.2372	0.0869855	+	+	6	8	7	1.16E-44	3.02E+08	50	14.7	103.13	
FAM129B	26.32306	26.30819	26.36918	0.03180134	+	+	20	21	19	1.08E-262	2.22E+09	223	42	84.137	
FAM49B	26.9587	26.80489	26.96499	0.09067257	+		16	16	16	0	4.62E+09	348	73.1	36.748	
FAM69B	22.53353	23.32292	22.59787	0.43836319			8	7	6	3.25E-38	2.56E+08	36	29	48.582	
FAM84B	22.87736	23.19877	22.57619	0.31134483	+		4	4	5	2.50E-34	2.83E+08	42	31.6	34.474	
FAS	NaN	20.57589	20.28376	0.2065671			1	2	2	4.42E-10	35610000	9	9.9	35.386	
FASN	NaN	20.54246	21.26671	0.51212209			2	2	3	5.08E-18	54301000	13	4.3	273.42	
FBXO17;SARS2	22.4968	22.33579	22.36349	0.08608433	+		4	4	3	1.82E-23	1.96E+08	26	34.2	31.479	
FDPS	22.42355	21.75935	21.02695	0.69857748			3	3	3	3.96E-08	92928000	14	10.5	40.532	
FEN1	20.93783	21.00874	21.04613	0.05500777	+		3	3	2	1.82E-17	62812000	20	17.9	42.592	
FKBP1A;FKBP12-E	20.08057	20.07051	19.9869	0.05142293	+		2	2	2	3.02E-07	28265000	11	25	11.951	
FKBP4	23.69328	23.4716	23.56395	0.11135289			8	9	8	4.29E-36	3.95E+08	59	33.6	51.804	
FKRP	NaN	NaN	20.76344	N/A			1	1	2	1.07E-10	34220000	9	8.5	54.567	
FLNA	22.46321	22.4234	22.17755	0.15471947			8	10	6	1.97E-43	1.77E+08	25	7.9	276.55	
FLOT1	24.25184	24.20978	24.1266	0.06373515	+		11	13	9	5.10E-92	6.56E+08	75	49.6	42.08	
FLOT2	26.19011	25.88453	25.78529	0.21099247	+		20	21	18	3.25E-136	2.15E+09	218	51.2	47.064	
FMNL2	20.1432	20.88718	20.84161	0.41700507	+		3	3	3	3.03E-13	43226000	7	5.7	123.32	
FMNL3	21.50072	21.30374	21.57168	0.1388214	+		7	9	4	1.05E-25	91132000	21	9.8	117.21	
FRS2;FRS3	20.93848	21.17078	NaN	0.16426091	+		3	3	2	2.65E-11	56786000	10	10.4	57.028	
FSCN1	23.70356	24.0269	23.79392	0.16682994			8	10	9	2.27E-45	4.6E+08	88	25.6	52.262	
FUBP1	22.06372	21.93891	22.16048	0.11108053			7	7	7	7.03E-21	1.5E+08	20	17.2	67.56	
FUS	20.58389	20.70691	20.48099	0.11310922			2	3	2	5.66E-08	52552000	8	7	53.354	
FYN	23.92687	24.03002	24.1673	0.12061807	+		13	11	8	1.22E-97	5.6E+08	63	44.5	60.761	
G6PD	20.42388	20.27753	20.84092	0.29233228	+		2	3	2	2.32E-07	39799000	9	6	59.256	

GALNT1	21.72517	21.79213	21.5588	0.12014263	+		5	5	5	4.33E-15	1.15E+08	17	12	57.379	
GALNT2	21.07881	NaN	21.71871	0.45247763			4	2	3	7.30E-12	58308000	13	10.7	60.793	
GANAB	22.41586	22.68528	21.77969	0.46500764			8	8	8	9.90E-45	2.25E+08	45	18.9	96.215	
GAPDH	26.08564	26.12384	25.93652	0.09898215	+		11	14	12	6.55E-212	2.43E+09	195	63.6	36.053	
GART	NaN	21.26081	NaN	N/A			2	2	1	2.50E-09	46445000	12	9.2	46.033	
GCN1L1	19.02212	NaN	NaN	N/A			2	1	0	0.000554	8830000	1	0.8	292.75	
GDI2	23.321	23.50749	22.99208	0.26096435			6	9	8	1.48E-35	3.09E+08	38	34.8	50.663	
GHITM	22.67521	22.39799	22.20392	0.23686436			4	4	4	5.38E-21	2.13E+08	25	16	35.282	
GLG1	22.02689	22.64037	22.07048	0.34230406			8	8	6	1.40E-34	1.87E+08	25	11.2	134.55	
GLIPR2	21.40572	21.02012	21.14482	0.19676816	+		2	2	2	3.20E-10	66801000	12	20.3	14.213	
GLO1	23.50227	23.70134	23.21546	0.24425677			8	6	6	9.91E-26	4.12E+08	45	50.3	19.043	
GLUD1;GLUD2	21.30747	21.83669	NaN	0.37421505			4	4	1	5.46E-18	89335000	17	14	61.397	
GMPS	21.1168	20.60467	NaN	0.3621306			2	2	1	4.76E-06	46202000	11	3.7	65.928	
GNA11	25.1617	24.92958	24.93841	0.13153966			11	12	10	5.04E-89	1.08E+09	90	50.7	42.123	
GNA13	23.27341	23.18956	22.71034	0.30379007			6	7	4	6.67E-45	3.51E+08	67	34.7	44.049	
GNAI1	24.53433	24.52468	24.42592	0.05999915	+	+	8	7	8	3.76E-210	7.19E+08	72	45.2	40.361	
GNAI2	26.06604	26.01097	25.89322	0.08828412	+	+	10	11	10	0	2.35E+09	176	62	40.45	
GNAI3	27.38385	27.35641	27.35018	0.01791385	+	+	16	17	12	2.06E-231	6.9E+09	372	61	40.532	
GNAQ	22.0552	NaN	21.83754	0.15390886	+		3	0	3	3.81E-40	90141000	10	37.9	42.142	
GNB1	22.78269	22.91187	22.75965	0.08204597			3	3	2	6.57E-13	2.23E+08	25	12.9	37.377	
GNB2L1	23.96508	24.11092	23.81499	0.14797009			9	10	7	3.02E-46	5.82E+08	60	40.1	35.076	
GNG5	20.15314	NaN	NaN	N/A			2	1	1	0.000146	43599000	6	23.5	7.3184	
GOLGA7	23.24076	23.13737	23.25035	0.06264442			4	4	3	7.22E-29	3.35E+08	66	40.1	15.824	
GOLIM4	25.032	25.0941	24.79506	0.1578089	+		8	11	6	9.67E-193	1.01E+09	107	17.7	81.879	
GORASP1	22.94621	22.26919	22.45365	0.34999927	+		3	2	2	3.86E-11	1.68E+08	19	16.3	23.906	
GORASP2	26.16687	26.09104	26.05418	0.05745706	+	+	10	8	9	9.70E-171	2.91E+09	148	36.9	47.145	
GOT1	20.90889	20.62756	20.7423	0.1414591			4	4	4	3.10E-14	1.09E+08	14	15.7	46.247	
GOT2	23.18332	22.89417	23.1432	0.15664891			7	7	5	1.98E-40	3.13E+08	46	29.5	47.517	
GPI	24.40763	24.70593	24.42892	0.1664185			8	9	9	1.84E-50	7.16E+08	79	24.9	63.146	
GPRC5A	23.40432	23.50349	23.04552	0.24093867			2	4	2	2.15E-82	5.38E+08	33	14.8	40.251	
GPRC5C	20.82803	20.68286	20.67168	0.08722064	+		3	3	2	1.10E-08	54549000	8	10	48.193	
GRPEL1	NaN	20.69532	NaN	N/A			1	2	1	2.01E-05	49739000	4	9.2	24.279	
GSPT1;GSPT2	22.11445	21.997	22.13392	0.07407279			6	5	4	9.09E-15	1.21E+08	12	10.8	68.6	
GSR	21.04827	20.97834	NaN	0.04944798			3	3	1	8.52E-12	45372000	8	9.8	47.267	
GSTO1	23.08724	23.46812	23.49387	0.22769885			6	5	5	2.11E-18	3.31E+08	41	30.3	27.566	
GSTP1	26.46456	25.98433	25.78469	0.349452			7	6	6	1.76E-166	2.4E+09	145	57.6	23.356	
HADHA	22.73657	22.82879	22.46556	0.18880633			10	10	5	9.30E-34	2.24E+08	39	19.3	82.999	
HADHB	24.79025	24.55993	24.66253	0.11538808			11	9	11	1.54E-60	8.77E+08	87	40.3	48.879	
HARS;HARS2	NaN	21.07874	21.27276	0.13719286			2	3	3	6.83E-13	68602000	14	11.1	49.623	
HCCS	25.28814	25.39869	25.26882	0.07007232	+		12	11	11	5.27E-112	1.41E+09	172	60.1	30.601	
HGSNAT	21.12016	NaN	20.93919	0.12796511	+		2	1	2	6.30E-07	44056000	6	4.1	70.495	
HINT1	21.82753	21.72912	NaN	0.06958638			2	2	1	9.21E-08	98861000	13	19.8	13.802	
HIST1H1C;HIST1H	20.78756	20.77064	NaN	0.01196425			2	2	0	1.99E-08	61928000	6	15	21.364	
HIST1H4A	19.15301	19.05804	20.28636	0.6834071			3	3	5	1.63E-22	2.42E+08	21	50.5	11.367	
HLA-B;HLA-C	23.08508	22.81628	23.10855	0.16239152			6	5	4	2.78E-39	2.6E+08	51	26.8	40.46	
HLA-C	20.16484	19.9693	20.01577	0.10215774			2	2	2	8.95E-30	30057000	10	26.5	40.648	
HM13	23.58681	23.35178	23.40133	0.12389319			5	5	5	1.24E-32	3.84E+08	48	22.7	36.813	
HMGB1;HMGB1P	21.13684	22.03022	21.90987	0.48480019	+		4	6	2	7.82E-22	1.73E+08	33	30.2	24.893	
HMGB2	20.77145	NaN	20.32762	0.3138352	+		2	1	2	5.90E-15	36018000	11	21.5	24.033	
HMGCS1	21.5484	21.48094	21.33522	0.10895785			4	3	3	6.74E-16	82919000	10	13.1	57.293	

HMOX2	21.90874	21.76971	21.97484	0.10470344			5	4	2	2.07E-19	97839000	13	34.8	32.837	
HNRNPA1;HNRNP	21.89045	22.22882	NaN	0.23926372			6	6	1	6.42E-25	1.57E+08	24	38.6	29.386	
HNRNPA2B1	NaN	21.33293	NaN	N/A			1	2	0	1.64E-11	85576000	13	12.6	36.006	
HNRNPAB	20.55347	20.23034	20.33484	0.16489003			2	2	2	1.05E-07	27971000	6	12.5	30.302	
HNRNPD	22.06842	22.32243	21.43787	0.45544132			4	4	4	4.76E-23	2.25E+08	25	46.8	12.553	
HNRNPF	21.73471	21.62729	21.51041	0.11218324			4	4	4	3.82E-16	1.2E+08	19	18.1	45.671	
HNRNPK	23.3073	23.50045	23.26256	0.12642537			10	11	10	6.15E-54	4.39E+08	62	37.4	47.557	
HNRNPM	24.56947	24.68581	24.53979	0.077177			19	20	16	1.10E-68	8.39E+08	106	38.6	73.62	
HNRNPU	21.90562	22.24554	21.94417	0.18612522	+		3	5	4	2.72E-23	1.62E+08	20	8.8	88.979	
HPCAL1	24.79917	25.06899	24.83321	0.14694316	+		9	8	10	1.42E-66	1.03E+09	133	59.6	22.313	
HPRT1	22.47689	22.44433	22.66785	0.12075255			4	6	3	8.38E-27	2.11E+08	36	42.7	24.579	
HSD17B10	22.61018	21.64073	22.20317	0.48679722			5	4	4	2.36E-16	1.51E+08	25	36.8	26.923	
HSD17B12	22.15909	22.07048	22.27881	0.10455142			4	7	5	8.83E-29	2.14E+08	30	34	34.324	
HSD17B4	21.56964	21.3252	21.37523	0.12913118	+		4	4	3	1.58E-18	86934000	9	11	79.685	
HSP90AA1	26.00288	26.05209	26.03633	0.02512935	+		14	16	14	0	2.06E+09	195	45.4	84.659	
HSP90AB1	27.57413	27.3765	27.49831	0.09970286			24	27	25	0	5.69E+09	458	51.8	83.263	
HSP90B1	25.09619	25.11486	25.02521	0.0473002			19	15	16	2.90E-86	1.02E+09	112	33.6	92.468	
HSPA1A	28.44269	28.14919	28.26521	0.14781861			26	26	25	0	1.05E+10	729	61.2	70.051	
HSPA4	23.92705	23.88883	23.87989	0.02504914			13	13	9	7.24E-63	4.74E+08	72	29.6	94.33	
HSPA4L	19.68594	NaN	NaN	N/A			2	2	2	4.67E-12	16792000	3	6.4	94.511	
HSPA5	24.16008	23.84326	23.27909	0.44624472			7	9	7	1.34E-82	5.09E+08	56	28.7	72.332	
HSPA8	26.40523	26.14903	26.11799	0.15764345			20	23	20	1.19E-192	2.5E+09	271	48.5	70.897	
HSPA9	24.27391	24.27767	24.09071	0.10687252			9	12	7	1.52E-78	6.28E+08	86	26.8	73.68	
HSPB1	20.94278	20.35165	20.77209	0.30423115			2	2	2	2.27E-10	68430000	9	19.4	20.406	
HSPD1	26.65922	26.83328	26.85007	0.10567443			21	23	19	0	3.46E+09	307	55.7	61.054	
HSPH1	21.97865	21.53198	NaN	0.31584339			5	7	2	1.87E-20	1.12E+08	17	12.8	92.115	
IDH2	20.15091	20.4707	NaN	0.22612568			2	2	1	9.00E-09	40406000	9	10.2	36.171	
IDH3A	21.30145	21.28161	21.8711	0.33476191			3	4	3	3.39E-16	93357000	18	20.2	35.786	
IFITM2;IFITM3;IFI	25.36052	25.43275	25.58182	0.11285147			3	3	3	3.84E-81	1.95E+09	79	29.5	14.632	
IGF2R	20.97673	21.08167	NaN	0.07420379	+		6	4	1	8.09E-19	54818000	14	3.5	274.37	
ILF2	21.53393	21.85958	21.6778	0.16319235			4	4	3	6.80E-17	1.21E+08	19	21	43.062	
ILF3	22.40158	21.95052	21.58628	0.40841972			5	6	5	2.65E-22	1.37E+08	36	15.1	74.606	
IMMT	21.9989	22.07878	22.38514	0.20388665			6	7	4	1.41E-28	1.48E+08	24	13.9	78.973	
IMPDH2	22.07045	22.0821	22.1626	0.050179			4	6	3	6.54E-18	1.48E+08	28	21.6	55.804	
IPO4	20.3738	20.21845	NaN	0.10984904			2	2	1	7.67E-05	18362000	4	6.4	33.154	
IPO5	23.2051	23.34462	23.16148	0.09566329			13	11	9	5.23E-51	3.51E+08	46	21.6	123.63	
IPO7	23.57288	23.17688	23.01006	0.28908224			5	7	5	2.80E-41	2.86E+08	44	13.1	119.52	
IPO9	20.41204	20.18822	20.50562	0.1630927			2	1	1	3.91E-10	36722000	5	3.6	115.96	
ISYNA1	20.60784	20.69812	20.49872	0.09984823			2	2	2	1.02E-08	42013000	8	8.8	44.786	
ITFG3	22.02591	22.24138	21.96948	0.1434929			3	4	4	5.25E-20	1.15E+08	21	12.1	59.659	
ITM2B	NaN	22.24362	22.53882	0.20873792			0	2	2	1.31E-46	1.93E+08	18	30.8	30.338	
ITM2C	NaN	20.154	NaN	N/A	+		1	2	0	3.44E-07	27790000	7	11.1	20.056	
JAM3	23.93022	23.79193	23.89199	0.07141155			6	6	4	1.64E-44	4.59E+08	47	27.1	35.02	
KHSRP	24.348	24.14763	24.47048	0.16298344			12	13	14	1.86E-50	6.38E+08	93	26.9	73.114	
KIAA0319L	21.99328	22.00299	22.31022	0.18024776			6	5	5	1.09E-29	1.85E+08	20	7	109.75	
KIAA2013	23.08592	23.23482	NaN	0.1052882			4	5	1	1.22E-30	2.55E+08	30	15.8	69.156	
KLC2;KLC1;KLC4	NaN	20.27571	NaN	N/A			1	2	0	0.000497	25631000	1	3.6	57.174	
KPNA2	22.4738	21.94074	21.79348	0.35792778			5	4	3	2.04E-17	1.32E+08	21	18.9	57.861	
KPNB1	25.71128	25.71295	25.80858	0.05570035			21	19	17	3.10E-111	1.66E+09	203	35.7	97.169	
KPRP	NaN	15.80196	NaN	N/A			0	1	0	0.000143	2078200	1	1.9	64.135	

LAMTOR1	26.55375	26.62965	26.54865	0.04536485	+	+	11	11	11	2.99E-230	3.45E+09	334	92.5	17.745	
LANCL1	NaN	20.87965	NaN	N/A			1	2	1	7.40E-10	50854000	8	47.6	9.2824	
LANCL2	20.80507	20.62221	20.95276	0.16558654	+		2	2	2	6.60E-12	47151000	15	11.8	50.854	
LARS	19.58202	19.77108	NaN	0.13368561			2	2	1	0.00032	21814000	4	1.9	129.21	
LBR	21.38406	NaN	21.43405	0.03534827			2	1	3	4.27E-08	1.08E+08	18	4.2	70.702	
LDHA	26.51539	26.47333	26.31114	0.10785229	+		10	13	9	7.36E-182	3.05E+09	215	61.1	36.688	
LDHB	27.27985	27.47212	27.26033	0.1170497			15	15	14	1.15E-180	6.48E+09	312	71.6	36.638	
LHFPL2	NaN	20.98287	NaN	N/A			1	2	0	2.52E-15	35884000	7	14.9	24.486	
LMAN2	20.90441	20.71557	20.85339	0.09768823			2	2	2	1.24E-08	52113000	8	8.4	40.228	
LMBRD1	23.308	23.73469	23.28884	0.25206272			3	3	2	4.29E-43	3.9E+08	25	9.9	44.211	
LMCD1	NaN	24.05838	NaN	N/A	+		0	1	0	0.00056	1.12E+08	2	5.4	22.897	
LMF2	20.96733	20.72492	NaN	0.17140975			3	2	2	6.84E-11	62129000	10	12.1	67.155	
LNP;KIAA1715	24.97657	25.05982	25.23655	0.13276148	+		10	11	10	1.25E-77	1.19E+09	137	40.4	47.094	
LNPEP	25.09136	25.03712	25.18703	0.07590316			19	19	17	3.65E-109	1.06E+09	132	27.7	117.35	
LOH12CR1	23.87173	23.72359	23.77012	0.07575739	+		8	7	8	7.39E-50	4.99E+08	48	64.4	20.247	
LRPPRC	24.53611	24.28115	24.41028	0.12748356			18	18	15	2.26E-75	6.2E+08	91	23.1	157.9	
LRRC1	21.77056	21.53782	21.48409	0.15227162			2	5	2	1.12E-54	1.25E+08	19	34.4	59.241	
LRRC57	25.87533	25.44793	25.71597	0.21599067	+	+	8	11	10	8.54E-69	1.48E+09	128	59	26.754	
LRRC59	21.42409	21.41988	20.92752	0.28548726			3	3	2	2.25E-09	78528000	23	10.4	34.93	
LSR	25.18616	24.92981	25.03884	0.12865072			8	10	8	5.70E-101	1.06E+09	107	29.7	69.428	
LTA4H	21.04647	21.41957	NaN	0.26382154			2	4	1	2.96E-18	85985000	12	18	69.284	
LUC7L2	21.9268	21.74123	21.44627	0.24233128			3	3	2	1.19E-07	99584000	17	7.9	46.513	
LUC7L3	22.30265	21.50286	21.37284	0.50350701			2	2	3	2.26E-13	99675000	14	14.6	42.571	
LYN	24.76096	24.49222	24.49881	0.15329016	+		13	12	12	2.96E-104	8.52E+08	94	43.9	58.573	
LYPLA1	21.59104	21.47843	21.33265	0.12954936			3	3	3	2.27E-11	1.05E+08	12	23.4	20.861	
LYPLA2	21.87815	21.69502	22.03184	0.1686243			2	2	2	5.01E-26	1.13E+08	18	22.5	24.737	
LZTS1	20.35638	20.23257	NaN	0.08754689	+		3	3	1	5.99E-09	46441000	8	7.7	55.611	
LZTS2	19.51343	19.23802	NaN	0.19474428			2	2	0	7.62E-08	19438000	3	5.8	72.759	
M6PR	23.10789	23.13745	22.81264	0.17960507			4	4	3	3.55E-16	3.16E+08	23	22.4	30.993	
MAN1B1	22.03473	22.41826	NaN	0.27119666			3	2	1	5.03E-21	1.34E+08	19	18.2	46.027	
MAPRE1	23.8968	24.13273	23.61448	0.25947081			6	9	5	8.68E-38	5.38E+08	64	46.3	29.999	
MARC2	24.11434	23.98442	23.87155	0.12149474	+	+	9	10	7	1.70E-45	5.6E+08	58	32.5	38.023	
MARCKS	29.71103	29.64721	29.87523	0.11763485	+	+	10	11	12	0	3.02E+10	411	48.5	31.554	
MARCKSL1	25.17646	24.8769	25.59309	0.35968617	+	+	5	5	5	3.32E-200	1.7E+09	76	60	19.529	
MBLAC2	25.39365	25.12558	25.40376	0.1577698			11	9	7	6.01E-35	1.13E+09	41	50.2	31.371	
MCCC1	27.11852	27.25413	27.03496	0.1106103			22	25	16	0	3.94E+09	306	52.8	80.472	
MCM3	20.61693	20.52162	20.49999	0.06221845			2	3	2	1.35E-09	51517000	11	4.3	90.98	
MCM4	20.66926	20.78732	20.70312	0.06079241			3	2	3	2.78E-07	36122000	5	4.4	96.557	
MCM7	20.07509	20.84468	NaN	0.54418231			2	3	1	7.60E-07	23541000	5	7.4	60.643	
MDFIC	21.91232	21.75283	21.872	0.08292994			3	2	2	4.55E-13	77699000	13	27.6	16.46	
MDH1	24.1757	24.03364	23.92352	0.12642667			7	9	5	1.96E-39	6.28E+08	73	32	36.426	
MDH2	24.6111	24.60603	24.62717	0.01103668			12	11	12	7.75E-70	9.66E+08	139	55.6	35.503	
ME2	20.81495	20.9143	20.46084	0.23835907			2	4	4	1.84E-13	55173000	10	8.7	65.443	
METTL7B	20.38184	21.62663	20.12792	0.80209197			3	3	3	1.21E-08	60108000	13	14.3	27.775	
MFSD5	21.2105	21.00601	21.50683	0.25180955			5	4	4	2.87E-16	88261000	16	15.8	49.764	
MGRN1	23.59616	23.5451	23.24952	0.18714253	+		8	7	6	1.33E-44	3.91E+08	63	35.5	58.304	
MGST3	20.07078	19.86178	NaN	0.14778532			2	2	0	1.07E-08	24071000	6	18.4	16.516	
MICB;MICA	NaN	20.52545	20.77876	0.17911722	+		1	2	2	0.000288	41059000	7	4.4	37.579	
MIF	24.09184	24.07169	24.56558	0.27951236			2	2	2	5.32E-19	5.92E+08	52	17.4	12.476	
MLEC	23.47346	23.60874	23.91004	0.22348917			4	5	4	2.11E-27	4.31E+08	46	53.4	16.729	

MRPS36	23.28715	23.15925	23.18139	0.06835418			3	3	3	4.24E-57	3.04E+08	38	56.3	11.466	
MSH2	19.62772	19.84227	NaN	0.15170976	+		2	3	0	2.12E-08	23074000	11	4.1	97.321	
MSN	22.97979	23.34096	22.3895	0.48030584			8	8	5	4.58E-42	2.94E+08	32	17	67.819	
MSRA	22.16389	21.7402	21.53103	0.3224327	+		4	5	4	6.79E-21	1.7E+08	22	36.6	23.627	
MTCH2	NaN	21.81714	21.05146	0.54141752			2	3	3	5.69E-12	1.01E+08	20	18.5	33.331	
MTDH	22.74012	23.25051	22.8011	0.27874297			7	6	7	1.77E-46	3.02E+08	38	19.4	63.836	
MTHFD1	22.69903	22.51263	22.89178	0.18958386			8	8	6	2.19E-41	2.5E+08	49	17.3	101.56	
MTHFD1L	19.44829	19.49191	19.48211	0.02288572	+		3	2	2	1.76E-08	20603000	5	4	105.79	
MYBBP1A	NaN	18.21077	NaN	N/A			0	3	0	2.16E-07	8901700	4	3.4	140.13	
MYL12A;MYL12B;	22.40351	22.11009	22.52174	0.21194707			3	3	3	6.59E-17	1.19E+08	23	24	19.794	
MYL6	21.82373	21.8789	21.38701	0.26948218	+		3	4	2	3.51E-13	1.11E+08	20	38.6	16.29	
NAA15	21.22052	21.11121	21.52688	0.2154788			5	4	5	8.65E-13	72237000	12	5.8	101.27	
NASP	23.92307	23.33238	23.25868	0.36417954			4	3	5	1.36E-25	3.56E+08	49	7.5	85.237	
NCAM1	25.21937	25.29049	25.28016	0.03842783			18	19	13	3.03E-90	1.39E+09	177	34.3	94.573	
NCL	21.74833	21.77896	21.57847	0.1080022			4	6	4	5.53E-18	1.41E+08	26	9.7	76.613	
NCR3LG1	21.78033	21.72912	21.82978	0.05033256			3	2	3	8.92E-19	1.1E+08	17	15	50.827	
NCS1	23.91068	23.72359	23.63567	0.14045349	+		7	6	7	4.15E-44	4.79E+08	83	62.6	21.878	
NCSTN	21.59614	21.32031	21.44116	0.13826648			4	4	4	1.28E-15	1.04E+08	16	10.4	50.26	
NDFIP2	20.38469	21.38448	21.4867	0.60888628			2	2	2	4.76E-09	53359000	12	18.9	26.019	
NDUFA4	22.46954	21.80786	NaN	0.46787841			2	2	1	2.60E-09	1.34E+08	16	46.9	9.3697	
NDUFAF4	25.46501	25.22781	25.30424	0.12107324	+		9	9	8	6.10E-46	1.41E+09	104	53.1	20.266	
NDUFB7	24.96772	24.91871	25.12498	0.10776518	+		4	5	5	3.39E-56	9.65E+08	161	51.1	16.402	
NELFB	NaN	19.92437	NaN	N/A			3	2	1	4.34E-06	29454000	5	5.5	65.697	
NME2;NME1-NM	24.60772	24.41461	24.35683	0.13138734			7	8	4	9.40E-34	7.37E+08	88	58.4	30.137	
NNT	21.30307	21.58742	NaN	0.20106581			3	2	1	1.55E-14	73856000	13	5.8	113.89	
NPC1	24.43256	24.55877	24.21623	0.17323456			9	8	7	9.01E-40	6.55E+08	71	9.3	142.17	
NPEPPS	22.8067	23.14951	23.22776	0.22395434			9	9	10	2.06E-43	2.83E+08	56	20.1	102.99	
NPM1	23.5643	23.89532	23.55929	0.19257704			5	6	4	1.66E-27	5.02E+08	54	26.8	29.464	
NR3C1	NaN	20.17085	NaN	N/A			1	2	1	8.68E-06	22580000	4	4.5	60.601	
NRAS;KRAS	23.10842	22.48707	22.62199	0.3268265			3	3	3	5.52E-21	2.44E+08	20	31.7	21.229	
NRP1	21.4268	21.52683	21.51922	0.05568568			2	2	3	9.80E-18	80978000	15	7.9	101.3	
NSUN2	20.18544	20.47981	NaN	0.20815102	+		2	2	1	6.40E-09	41635000	8	5.6	82.392	
OAT	22.71324	22.67709	22.74351	0.03325335			4	4	4	9.80E-21	2.17E+08	37	17.8	48.534	
OGFRL1	22.46051	22.85523	22.33293	0.27229819	+		6	6	4	7.51E-42	2.54E+08	28	26.6	51.251	
OXCT1	19.93431	NaN	20.14557	0.14938338			2	1	2	1.07E-09	24866000	6	7.9	56.157	
P4HB	22.93627	22.85424	22.77844	0.07893549			7	8	8	3.70E-32	2.61E+08	37	24.8	57.116	
PA2G4	24.20479	24.33361	24.21438	0.07176623			11	10	8	5.62E-45	6.59E+08	82	32	43.786	
PABPC1	22.11248	21.85399	21.81398	0.16202887	+		7	7	5	4.81E-22	1.81E+08	24	17.4	61.18	
PAICS	22.83499	23.20008	22.70639	0.25611146			7	7	5	2.21E-45	2.98E+08	50	36.5	47.079	
PAIP1	20.77483	20.60955	NaN	0.11687061			2	2	1	2.01E-06	32710000	10	6	39.908	
PALD1	21.53583	21.78509	21.60032	0.12937636	+		5	5	4	1.09E-22	91871000	18	10.7	96.753	
PALM	23.01611	22.64467	23.05179	0.22545784			4	3	4	2.48E-13	2.27E+08	31	14.2	42.075	
PARK7	22.28543	22.09957	22.11826	0.10233854			6	6	3	7.71E-28	1.56E+08	18	45.5	19.891	
PARP1	22.03825	22.1784	22.10894	0.0700759			6	6	6	5.75E-18	1.29E+08	10	8.4	113.08	
PC	28.65243	28.52533	28.41498	0.11882342	+		45	49	45	0	9.94E+09	976	54.9	129.63	
PCBP1	24.10821	24.10677	23.60456	0.29036766			8	6	6	2.17E-35	5.24E+08	48	37.4	37.497	
PCBP2	23.80114	23.82532	23.65469	0.0923281	+		4	3	4	1.49E-32	4.02E+08	40	31.9	38.15	
PCCA	27.93601	27.67647	27.62024	0.16844065			30	35	28	2.86E-259	5.69E+09	474	66.1	77.047	
PCCB	22.43637	22.42632	21.92009	0.29521598			6	8	7	5.88E-21	1.18E+08	25	21.8	52.423	
PCMTD1	21.12515	20.81065	NaN	0.22238508	+		4	4	2	8.92E-11	54060000	10	14	40.675	

PCMTD2	24.95819	24.81836	25.00971	0.0990131	+			13	14	13	1.14E-75	1.06E+09	148	41.6	41.071
PCNA	22.79346	22.60277	22.86325	0.13483518				3	4	3	2.97E-33	2.61E+08	27	47.1	28.768
PDCD6IP	21.34061	21.25396	21.26046	0.04826057				4	4	4	2.39E-14	74616000	16	7.9	96.022
PDHB	21.0097	21.4494	21.04833	0.24347673				3	3	3	1.07E-11	72446000	13	15.5	37.514
PDIA3	22.71181	23.02695	22.87431	0.15759571				8	4	7	6.59E-31	3.27E+08	34	26.2	54.963
PDIA6	NaN	20.0675	NaN	N/A				1	2	0	8.59E-08	25632000	6	9.2	47.837
PEBP1	22.67357	22.56781	22.22111	0.23668056				5	5	3	5.73E-31	2.5E+08	25	56.1	21.057
PFN1	24.73071	24.61229	24.73645	0.0700856	+			6	7	5	3.43E-39	8.26E+08	89	60	15.054
PGAM1	24.22133	24.74227	24.07194	0.35190864				9	10	5	1.57E-138	8.34E+08	138	64.2	28.804
PGD	22.35617	22.35646	21.86026	0.28639752	+			5	6	4	5.96E-32	1.53E+08	23	31.9	51.872
PGK1	26.1034	26.09319	26.20613	0.06246751				19	18	16	2.09E-152	2.27E+09	202	60	44.614
PGLS	22.14482	21.76397	21.99175	0.19164241				4	5	3	3.99E-18	1.36E+08	20	45.7	27.547
PGM1	20.25534	20.24748	20.33385	0.04775873				2	2	2	9.58E-07	33366000	4	6.8	61.448
PGRMC1	21.63462	21.82854	NaN	0.13712215				2	3	2	3.00E-10	1.07E+08	10	20	21.671
PHB	22.63622	22.35837	22.49882	0.13892779				3	2	3	2.23E-16	1.64E+08	24	30.3	22.27
PHGDH	24.19895	24.16906	24.06924	0.06792413				7	8	7	9.28E-66	5.38E+08	70	22.1	56.65
PI4K2A	27.26919	26.49091	26.61034	0.41913932				28	27	26	0	3.57E+09	312	72.7	54.022
PI4K2B	24.27227	24.18858	23.93822	0.17381674				12	13	10	9.25E-55	5.78E+08	72	38.5	54.744
PIK3R4	23.23397	23.03108	23.25638	0.12411463	+			10	13	10	3.04E-44	3.01E+08	41	13.8	153.1
PITPNB	20.1149	20.09816	20.29064	0.10662499	+			3	3	3	1.92E-06	35896000	5	11.8	31.54
PKM;PKM2	26.5402	26.36446	26.25969	0.14174341				21	20	18	4.64E-254	2.73E+09	244	55.4	57.936
PLGRKT	22.48446	23.8906	22.72067	0.75296758	+			4	4	3	8.84E-31	6.36E+08	58	34	17.201
PLIN3	22.66798	21.95649	22.43025	0.36221129				4	5	4	6.89E-20	1.37E+08	24	16.8	45.803
PLP2	25.79788	25.87509	25.73099	0.07211156				2	2	2	7.94E-58	2.08E+09	53	27	16.691
PLS3	26.24465	26.2498	26.11064	0.07889942				25	23	22	5.61E-154	2.43E+09	264	47.9	70.81
PLSCR1	26.10781	25.9748	25.81321	0.14753087				8	8	5	3.70E-91	1.96E+09	137	34.7	34.217
PLSCR3	22.82613	23.18395	23.17965	0.20535743				3	5	5	6.59E-22	2.71E+08	36	26.1	31.648
PLXNB2	20.82462	20.78676	20.6909	0.06892455				3	4	3	1.67E-11	56497000	9	2.6	205.12
PMPCA	20.81425	20.96014	21.30558	0.25232844				4	4	2	1.09E-26	71790000	14	9.1	58.252
PMPCB	20.7854	21.01951	20.71238	0.16045158				3	3	3	2.59E-10	61659000	9	6.7	54.366
PNP	20.88032	20.97687	21.13057	0.12620793				3	4	4	2.36E-15	72023000	13	25.3	32.118
PODXL	22.31951	22.15227	22.29019	0.08930361				2	2	2	1.04E-07	1.31E+08	10	5.5	55.385
POM121C;POM12	NaN	19.20624	NaN	N/A				1	2	0	1.40E-06	12679000	6	2.9	125.06
PPA1	NaN	20.94278	NaN	N/A				2	3	1	7.87E-12	63727000	8	16.3	32.66
PPA2	NaN	19.60138	NaN	N/A				1	2	1	3.48E-05	23105000	8	10.8	25.991
PPIA	25.13073	25.01112	24.92289	0.10431407				7	8	8	6.20E-36	1.04E+09	80	52.7	18.012
PPIB	21.9206	21.64698	NaN	0.19347856				2	2	1	1.24E-12	91776000	14	19	23.742
PPM1A	26.02462	26.00462	25.73727	0.16044004	+			14	15	12	1.82E-170	2.21E+09	201	52.9	42.447
PPM1B	25.35971	25.0005	25.15151	0.18036217	+			10	10	8	3.95E-127	1.2E+09	101	45.5	52.642
PPM1G	28.19437	28.03669	28.17612	0.08625231	+	+		23	23	21	0	1E+10	538	63.9	59.271
PPME1	19.9502	20.29064	NaN	0.24072743				2	2	1	4.05E-05	25290000	2	6.5	42.315
PPP1CA	21.19802	21.10847	NaN	0.06332141				3	2	1	1.53E-06	52097000	8	8.5	37.512
PPP2CA;PPP2CB	21.38653	NaN	21.34147	0.03186223				2	2	2	8.05E-20	47984000	8	11	35.594
PPP2R1A	24.67573	24.73309	24.61583	0.05863458				14	16	13	1.89E-64	7.77E+08	100	39.4	65.308
PPP3CA	22.40702	22.24045	22.5971	0.1784541				3	5	6	1.52E-21	1.83E+08	26	17.2	57.658
PPP3R1	25.30187	24.98377	25.02918	0.17205111	+			8	7	8	8.28E-69	1.21E+09	105	84.7	19.3
PRAF2;WDR45	23.59696	23.19934	23.63057	0.23985781				4	3	3	1.65E-12	3.82E+08	38	26.4	19.258
PRDX1	25.8699	25.76517	25.55839	0.15851647				7	10	7	1.50E-46	1.92E+09	93	53.3	22.11
PRDX2	22.97713	22.99025	22.87733	0.06175638				3	3	3	2.54E-15	2.63E+08	43	19.2	21.892
PRDX3	21.87234	21.96993	21.89008	0.05198483				3	5	4	2.50E-16	1.37E+08	20	31.5	25.838

PRDX4	21.50887	21.97935	22.24945	0.37478086			2	3	3	3.40E-26	1.6E+08	25	35.4	30.54	
PRDX6	23.78666	23.8348	23.90371	0.05883133			6	7	7	1.10E-51	4.38E+08	73	50.9	25.035	
PRKAA1	22.42949	23.22383	22.38184	0.47296824			5	7	5	8.35E-25	2.47E+08	38	20	64.009	
PRKAB1	24.36546	24.62171	24.55227	0.13252921	+		6	8	5	1.57E-105	8.52E+08	74	51.5	30.382	
PRKAB2	NaN	21.50707	21.72267	0.15245222	+		1	4	2	4.10E-17	1.05E+08	18	16.9	30.302	
PRKACA	26.73815	27.29667	26.47139	0.42114775	+	+	18	20	19	1.49E-121	4.92E+09	322	63.2	40.589	
PRKACB	23.58417	23.52109	23.27994	0.16056582	+	+	3	5	5	3.38E-84	4.63E+08	55	62.7	40.622	
PRKAG1	21.04267	20.49267	21.11299	0.33966695	+		4	4	2	1.49E-12	70729000	9	20.2	28.285	
PRKDC	23.01647	22.96608	23.04565	0.04025338			15	17	13	3.00E-48	2.71E+08	56	5.8	469.08	
PRMT1	21.93503	21.95504	21.94396	0.01002423	+		4	4	3	5.30E-21	1.38E+08	19	25.5	37.709	
PRMT5	NaN	NaN	18.47548	N/A			1	1	1	0.002947	5935100	2	5.9	19.617	
PRNP	22.11366	22.45704	22.28605	0.17169048			5	5	3	1.08E-22	1.97E+08	30	21.5	26.885	
PROCR	22.56929	22.48277	22.37727	0.09616621			2	4	2	4.74E-21	1.9E+08	23	20.6	26.671	
PRPF19	21.47576	21.10412	21.15505	0.20147997			3	4	4	4.07E-13	88949000	9	8.7	55.18	
PRPF38B	21.91207	21.4892	22.14759	0.33360808			2	4	3	1.13E-13	95439000	16	10.3	64.467	
PRPS1;PRPS1L1;P	NaN	20.61081	NaN	N/A			3	3	2	2.39E-07	36451000	4	8.5	34.834	
PSAT1	20.12414	NaN	NaN	N/A			2	1	1	4.57E-06	28048000	4	7.6	40.422	
PSMA1	21.03349	21.10374	20.8783	0.11535611			4	4	3	1.52E-13	1.21E+08	13	21.3	29.555	
PSMA3	21.77322	21.59149	21.602	0.10202332			3	3	2	1.19E-12	93841000	16	15.3	27.647	
PSMA4	22.28602	21.78061	22.42275	0.33824994			3	3	2	4.97E-12	1.39E+08	23	23.2	24.526	
PSMA6	21.79289	21.74989	NaN	0.03040559			3	4	1	4.80E-16	1.26E+08	22	27.7	16.645	
PSMA7	NaN	21.52133	NaN	N/A			1	2	1	5.66E-07	70511000	6	12.9	27.887	
PSMB2	21.57464	21.70434	21.46094	0.12178762			2	2	2	2.48E-07	1.12E+08	12	14.4	22.836	
PSMB5	22.18858	22.00802	21.67189	0.26221933			4	5	3	2.35E-23	1.34E+08	24	30.4	28.48	
PSMB6	21.23754	21.38837	21.02817	0.18089109			3	3	3	6.56E-10	1E+08	16	13	25.357	
PSMC1	28.50476	28.52641	28.4817	0.02235871	+	+	24	24	22	0	1.32E+10	755	63	49.184	
PSMC2	24.98872	25.46781	25.1208	0.24744976			15	15	11	1.29E-82	1.22E+09	91	50.8	48.633	
PSMC3	23.16013	23.13444	NaN	0.01816557			3	2	1	1.26E-20	2.6E+08	19	19.4	47.352	
PSMC4	21.7336	21.84579	NaN	0.07933031			3	4	2	2.15E-10	94116000	11	11.6	43.507	
PSMC5	25.27494	25.49286	24.82556	0.3402746			5	5	5	3.54E-25	1.28E+09	61	18.3	44.784	
PSMC6	22.0879	22.10364	21.5838	0.29569077			5	5	5	9.29E-26	1.35E+08	23	24.2	44.172	
PSMD1	21.818	21.73281	22.30744	0.31010979			5	4	4	6.08E-17	1.38E+08	18	7.3	102.26	
PSMD11	21.38232	21.40624	21.2064	0.10912992			4	6	5	5.40E-15	95605000	20	14.2	47.463	
PSMD12	21.85559	21.89874	NaN	0.03051166			5	5	1	3.20E-17	1.09E+08	28	14	52.904	
PSMD13	22.59439	22.40606	22.34754	0.12898818			5	6	5	3.41E-30	1.83E+08	35	24.7	42.945	
PSMD14	20.33047	20.16852	20.07012	0.13146133			2	2	2	3.58E-07	39075000	6	6.5	34.577	
PSMD2	24.25897	24.32649	24.13915	0.09487892			10	15	11	5.34E-103	6.21E+08	92	31.1	100.2	
PSMD3	21.51031	21.17408	21.07666	0.22752055			4	4	3	1.43E-17	95938000	13	16	41.183	
PSMD6	21.18544	21.41194	21.29502	0.11326982			2	2	3	2.35E-14	79084000	11	20.3	45.531	
PSMD8	21.91437	22.09934	21.94839	0.09845233			4	4	3	1.70E-13	1.18E+08	23	29.7	19.781	
PSME1	NaN	21.48871	NaN	N/A			2	2	2	1.33E-10	44860000	7	22.3	26.87	
PSME2	20.33473	NaN	20.35057	0.01120057			2	1	2	1.64E-05	17704000	3	9.2	26.011	
PTBP1	NaN	20.80601	NaN	N/A			1	2	1	3.30E-15	51454000	7	11.5	57.221	
PTGES3	NaN	20.95731	NaN	N/A			2	3	1	3.72E-06	71886000	9	20.9	16.476	
PTGFRN	26.77906	26.81115	26.67492	0.07121975	+		26	23	23	2.36E-238	3.47E+09	299	43.5	98.555	
PTK7	24.24067	24.37106	23.98198	0.19803422	+		13	15	12	5.17E-82	6.62E+08	83	25	118.39	
PTPLAD1	24.28891	24.29207	24.30612	0.00916126			8	8	6	1.61E-45	6.68E+08	68	29.8	43.159	
PTPN1	19.92768	NaN	NaN	N/A			3	1	1	7.57E-09	25376000	4	9.4	49.966	
PTRH2	21.15925	21.08868	21.61009	0.28287371			3	3	2	8.83E-13	71580000	9	29.1	19.193	
PTTG1IP	22.73581	23.32347	22.83584	0.31441302	+		2	2	1	2.29E-11	2.22E+08	34	22.8	20.324	

PVR	24.22155	24.54728	24.25544	0.17908059				5	5	5	3.01E-21	6.87E+08	43	13.5	39.304	
QKI	NaN	21.35805	NaN	N/A				1	3	0	1.21E-07	75631000	12	10.1	35.232	
RAB14	20.0592	19.99079	20.15078	0.08027414				2	2	2	0.000455	30018000	4	7.9	23.897	
RAB1A;RAB1B;RA	NaN	NaN	21.9578	N/A				1	1	2	1.52E-14	2.11E+08	17	37	19.018	
RAC1;RAC3;RAC2	21.97998	21.64768	21.90628	0.1745131				4	4	3	7.78E-12	1.24E+08	16	27.1	21.45	
RAD23B	21.98933	22.39239	22.40663	0.23692454				3	5	2	2.04E-15	1.52E+08	21	21	43.171	
RAN	24.51593	24.43479	24.74227	0.15935152				5	6	5	1.60E-29	7.34E+08	68	31.5	24.423	
RANBP1	23.17954	22.75448	23.20561	0.25326992	+			3	3	3	1.70E-12	2.34E+08	21	15.4	23.31	
RANGAP1	22.87243	22.86437	23.28856	0.24261296				7	7	6	2.84E-35	2.18E+08	44	21.3	63.541	
RAP1B;RAP1A	20.6784	20.69132	NaN	0.00913582				2	2	0	1.28E-05	42316000	6	43.8	5.351	
RAP2B	25.04414	24.98828	24.95011	0.04729152				8	8	8	1.44E-47	1.13E+09	103	62.3	20.504	
RAP2C	22.9304	22.53585	22.3853	0.28150459				4	5	4	8.60E-42	1.87E+08	24	59	20.745	
RARS	20.87658	20.81511	NaN	0.04346585				3	4	1	1.70E-11	49153000	9	8.2	75.378	
RBM39	NaN	19.91444	NaN	N/A				1	2	2	2.95E-09	43638000	11	13.7	36.514	
RCC2	20.36698	20.57764	NaN	0.14895911				2	3	1	5.35E-09	47052000	8	6.5	56.084	
REEP5	22.44748	23.73159	NaN	0.90800289				3	3	1	2.29E-30	3.68E+08	49	15.9	21.493	
RER1	22.37029	22.11873	21.7565	0.30855339				2	2	2	8.78E-10	1.09E+08	10	23.4	18.388	
RFC2	20.3538	20.50368	20.55777	0.10566733				3	2	3	1.32E-08	29878000	8	10	35.243	
RFTN1	22.0157	21.95759	21.89301	0.06137343	+			6	6	4	1.10E-18	1.28E+08	17	14	63.145	
RGS19	22.57277	22.44081	23.0818	0.3384753				2	3	3	1.36E-08	1.78E+08	16	16.1	24.635	
RHBDD2	20.78117	21.17396	20.34191	0.41624122				2	1	1	2.05E-09	71679000	20	11.8	39.202	
RHOB	24.40613	23.91543	24.47772	0.30607234				5	4	3	5.78E-22	5.85E+08	52	36.2	22.123	
RNF141	24.73293	24.54775	24.67325	0.09451947	+	+		5	7	6	2.54E-57	8.38E+08	80	57.4	25.535	
RNH1	20.5682	21.18604	20.20156	0.49755268				2	2	2	1.43E-08	32885000	5	11.1	49.973	
RP2	25.20944	25.00697	25.37921	0.18635923	+	+		11	11	10	3.04E-38	1.43E+09	115	26.9	39.641	
RPA2	20.45513	20.19304	20.48601	0.1609742	+			2	2	2	0.000263	40789000	9	11.2	19.433	
RPL10	23.93571	24.08829	24.16315	0.11591206	+			4	4	4	7.64E-22	4.6E+08	24	35.5	22.975	
RPL10A	23.10719	23.11081	22.89946	0.12099151				4	5	4	2.39E-14	2.93E+08	30	26.7	24.831	
RPL11	NaN	23.03461	23.31507	0.19831517				1	2	2	1.67E-78	2.37E+08	24	22	20.124	
RPL12	22.7444	22.81673	22.26994	0.29701942				4	4	3	3.58E-35	2.19E+08	29	54.5	17.818	
RPL13	28.14788	28.37476	27.93208	0.22136311				13	13	11	0	1.11E+10	425	46.9	24.261	
RPL13A	26.0239	25.73138	25.64532	0.19845112				5	7	5	3.89E-25	1.72E+09	144	30	23.577	
RPL14	22.22326	21.98315	21.85098	0.18873002				2	2	2	1.42E-12	1.56E+08	25	27.4	14.558	
RPL15	27.27764	27.15788	26.70859	0.30000607	+			11	14	11	7.79E-90	6.07E+09	363	52.5	24.146	
RPL18	28.70853	28.61695	28.4243	0.14507907	+			11	10	11	6.72E-251	1.45E+10	493	54.3	21.634	
RPL18A	NaN	23.13254	23.29095	0.11201279				1	2	2	1.25E-17	3.14E+08	33	27.7	16.714	
RPL19	25.29127	25.13289	25.16806	0.08316836	+			5	5	4	2.32E-38	1.15E+09	64	22.3	23.134	
RPL21	NaN	22.09249	NaN	N/A				0	3	1	3.16E-16	1.13E+08	17	20.6	18.565	
RPL22	23.01728	23.14344	22.99967	0.07841797				2	2	2	8.96E-09	2.62E+08	17	51.1	5.0827	
RPL23	23.95109	24.05639	24.1168	0.08386225				4	4	4	9.67E-32	5.17E+08	48	43.6	14.865	
RPL24	23.32773	23.17598	22.9145	0.20902906				5	4	5	1.08E-14	3.21E+08	36	39.7	14.369	
RPL27	22.46849	22.05976	22.47719	0.23853152	+			3	3	2	3.10E-10	1.47E+08	28	20.6	15.798	
RPL27A	25.92504	25.78457	25.47318	0.23125491				6	5	5	2.39E-23	1.8E+09	126	35.8	16.561	
RPL28	27.05941	27.16863	26.84062	0.16702726				11	13	11	7.10E-92	4.38E+09	195	61.3	15.747	
RPL29	26.71764	25.80784	25.61714	0.58810469				2	4	2	1.11E-21	1.78E+09	45	23.3	17.752	
RPL3	24.07128	24.10941	24.0375	0.03597692				9	9	8	4.95E-48	6.26E+08	72	33	46.108	
RPL30	21.98527	21.32734	21.46478	0.34705222				2	3	2	1.65E-14	1.08E+08	13	31.6	12.656	
RPL31	25.73694	25.17706	24.95952	0.40107587				4	4	4	4.45E-157	1.33E+09	79	32.8	14.463	
RPL32	29.07664	29.49379	28.977	0.2741697				11	12	10	2.10E-291	2.9E+10	1376	60.2	15.616	
RPL34	27.27302	27.49335	27.11247	0.19122029				9	9	9	6.28E-37	6.21E+09	189	35.9	13.293	

RPL35	24.368	24.62428	24.45995	0.12983232			4	5	2	5.74E-39	7.34E+08	61	29.3	14.551	
RPL35A	NaN	22.18135	NaN	N/A			0	2	1	7.56E-07	1.13E+08	9	20.9	12.538	
RPL36	27.70101	27.64506	26.99992	0.38962871			8	7	8	2.39E-70	5.55E+09	202	43.8	12.254	
RPL36A;RPL36AL	21.93632	22.16484	21.91823	0.13745613			2	3	3	1.62E-08	1.25E+08	26	32.1	12.441	
RPL39P5;RPL39	23.63434	24.34503	23.95456	0.3559232			2	2	2	9.47E-13	5.66E+08	125	19.6	6.3225	
RPL4	25.74509	25.61944	26.02162	0.20575289			11	13	11	1.90E-66	1.96E+09	173	45.9	47.697	
RPL5	23.05545	23.89375	23.53517	0.42060627	+		5	5	4	2.65E-37	4.71E+08	36	35	34.362	
RPL6	25.51087	25.61672	26.52019	0.5547055			11	8	8	2.23E-60	2.27E+09	215	42.7	32.728	
RPL7	23.78746	23.79025	24.14577	0.20606969			7	7	6	4.70E-28	5.22E+08	51	34.7	29.225	
RPL7A	23.58417	23.60671	23.49314	0.06012855			5	6	4	4.33E-24	4.72E+08	43	36.8	29.995	
RPL8	22.85766	23.1233	23.00311	0.13302002	+		4	3	2	2.66E-24	3.7E+08	47	20.2	28.024	
RPL9	22.2338	21.97271	21.86333	0.19034179			4	3	3	7.12E-18	1.7E+08	26	33.3	21.863	
RPLP0;RPLP0P6	24.79084	24.65622	24.79233	0.07815657			8	8	7	5.74E-73	9.1E+08	121	42.3	34.273	
RPRD1B	20.02743	19.88124	20.41967	0.27842731			2	3	2	8.37E-09	38774000	6	11.7	36.899	
RPS11	22.35114	22.80674	22.64159	0.23065381			5	5	5	3.49E-19	2.43E+08	32	51.3	18.431	
RPS12	22.30025	22.60666	22.79444	0.2494568			2	3	2	2.18E-18	1.77E+08	28	31.8	14.515	
RPS14	22.75839	22.77177	23.00703	0.13985			3	3	2	3.13E-22	2.63E+08	34	31.8	16.273	
RPS15A	23.39113	23.65053	23.55906	0.13156469			4	4	3	2.46E-16	3.77E+08	32	54	11.477	
RPS16	22.74353	22.75156	22.08871	0.38039975			6	7	3	1.02E-20	2.76E+08	41	42.6	14.419	
RPS17L;RPS17	20.59587	20.39142	20.5976	0.11854183	+		2	3	2	1.09E-09	48831000	10	48.9	15.55	
RPS2	24.5559	24.7571	24.6151	0.10340058			10	9	9	5.94E-44	8.74E+08	96	42.7	31.324	
RPS23	22.5271	23.00546	22.55105	0.26953365	+		2	2	3	3.95E-15	2.64E+08	51	28.7	15.807	
RPS25	23.65633	23.594	23.54898	0.0539071			3	3	4	1.89E-10	3.92E+08	48	28	13.742	
RPS26;RPS26P11	22.41261	22.66659	22.69825	0.15657714			2	2	2	8.42E-07	2.47E+08	17	20.9	13.015	
RPS27	20.51999	NaN	NaN	N/A			1	1	1	1.73E-16	33938000	3	38.1	9.461	
RPS27L	NaN	22.8581	22.97885	0.08538314			1	2	2	1.47E-16	2.12E+08	17	38.1	9.4771	
RPS3	25.29284	25.14965	25.17653	0.0761073			12	12	13	2.23E-58	1.29E+09	114	70.8	26.688	
RPS3A	25.23184	25.19494	25.1476	0.04222768			9	9	11	5.59E-45	1.15E+09	95	52.7	29.945	
RPS4X	24.14663	24.28397	24.49351	0.17468783			9	8	7	3.27E-46	6.52E+08	84	47.1	29.597	
RPS5	24.40639	24.2868	24.40295	0.06807401			7	7	8	2.45E-60	7.69E+08	99	54	22.391	
RPS6	24.20411	23.95802	23.9444	0.14617061			4	3	3	5.45E-46	6.43E+08	66	18.9	28.68	
RPS8	23.93885	23.93472	23.78706	0.08646843	+		6	7	6	3.91E-72	5.6E+08	85	43.3	24.205	
RPS9	27.29632	27.66133	27.11178	0.2796702			13	14	14	2.54E-61	5.61E+09	278	50.5	22.591	
RPSA;RPSAP58	22.81987	22.29249	22.58589	0.26424732			4	4	2	1.00E-33	1.99E+08	22	28.1	29.404	
RRAS2	24.6049	24.46401	24.59958	0.07985144			8	8	8	3.27E-37	8.28E+08	62	47.5	23.399	
RRM1	22.29095	22.36514	22.05715	0.16074018			4	5	4	1.16E-22	1.53E+08	25	13.5	79.219	
RRM2	NaN	NaN	19.98676	N/A	+		1	1	2	9.50E-06	20192000	4	8.3	33.789	
RSPRY1	21.00546	21.17725	NaN	0.12147387			2	3	1	1.08E-12	45317000	6	11.1	64.18	
RTN4	24.02935	23.29221	23.12855	0.4798612			4	4	3	2.39E-18	3.79E+08	33	20	37.144	
RUVBL1	21.15808	21.37109	21.4173	0.13826522			4	4	3	3.14E-17	79579000	14	18.6	50.227	
RUVBL2	22.09992	22.32649	22.31711	0.1281883			8	8	7	2.07E-20	1.46E+08	19	22.2	51.156	
S100A11	21.79919	NaN	NaN	N/A			2	1	2	3.09E-09	1.01E+08	12	37.1	11.74	
SAE1	20.79875	NaN	NaN	N/A			2	1	1	2.38E-10	44714000	8	19.6	29.422	
SAMM50	23.88269	23.57957	23.68603	0.15378048	+		8	11	6	9.07E-36	4.25E+08	40	32.2	51.976	
SAR1B;SAR1A	NaN	20.40582	NaN	N/A			1	1	0	0.002902	17899000	4	22	5.7567	
SCAMP1	23.5715	24.26506	24.64801	0.54567236			6	6	4	4.82E-64	5.7E+08	61	27.2	37.873	
SCAMP2	24.80671	24.92538	24.64801	0.13916559			3	3	2	1.88E-99	1.02E+09	65	13.7	36.648	
SCAMP3	26.92071	26.54753	26.72051	0.18675538			8	7	8	3.89E-222	3.12E+09	222	38.9	38.287	
SCARB1	22.78425	22.76747	23.27853	0.29033791	+		5	4	6	1.53E-27	2.76E+08	34	19.8	53.579	
SCARB2	26.15462	25.98869	26.27487	0.14369633	+		13	10	11	1.66E-132	2.9E+09	170	37.4	54.29	

SCARF2	21.29793	21.17615	21.28782	0.06758053				3	4	3	2.19E-13	87420000	12	5.7	91.815	
SCP2	20.36698	20.12048	NaN	0.17430182	+			2	2	2	0.000469	24811000	2	16.4	15.079	
SCRIB	23.20824	23.03508	22.71678	0.24927636				11	13	10	9.44E-47	2.73E+08	50	10.9	174.88	
SDHA	20.51749	20.60946	20.79559	0.14168184				2	2	2	6.04E-08	46273000	8	4.5	63.566	
SEC22B	20.20407	20.06671	NaN	0.09712819				2	2	1	1.29E-07	30106000	6	14.9	24.593	
SEC61A1;SEC61A2	23.37199	23.13363	23.50434	0.18786428	+			6	5	5	1.48E-18	3.22E+08	35	12.4	52.264	
SEC62	20.10776	19.9648	NaN	0.10108799				2	2	1	0.00078	27566000	5	20	9.8703	
SEPT11	20.86121	NaN	NaN	N/A				2	1	0	4.85E-07	38501000	5	9.4	49.005	
SEPT7	NaN	20.82462	NaN	N/A				2	2	2	3.32E-09	46067000	7	12.9	43.036	
SERINC1	25.25389	24.84757	25.01171	0.20440525	+			3	3	3	1.04E-99	1.03E+09	61	10.4	50.494	
SERINC3	21.9502	20.94163	21.50969	0.50562744	+			3	3	3	2.88E-12	1.43E+08	20	10	46.821	
SERPINB6	22.13806	22.70988	23.18195	0.52273871				7	6	5	5.20E-31	2.78E+08	38	39.4	42.621	
SERPINH1	20.27969	20.51229	19.87422	0.32291433				4	4	3	1.58E-28	73732000	13	16.5	46.44	
SF3A3	NaN	20.1795	NaN	N/A				1	2	0	0.000314	16303000	0	4.2	58.848	
SFN	NaN	NaN	23.3199	N/A				1	1	2	2.70E-19	7.2E+08	30	20.8	24.336	
SFPQ	19.53623	20.01754	NaN	0.34033756				2	2	0	0.000246	26672000	2	3.7	76.149	
SFT2D2	21.53012	NaN	NaN	N/A				1	1	1	4.92E-08	72172000	10	15.7	11.756	
SFT2D3	24.09997	24.10701	24.09909	0.00434094				4	3	3	6.38E-56	5.28E+08	46	27	21.789	
SFXN1	23.2165	22.85085	22.9767	0.18576068				5	5	3	3.46E-56	2.4E+08	40	35.1	35.619	
SGTA	NaN	20.78963	NaN	N/A				2	4	1	3.08E-10	51623000	9	17.3	34.063	
SHISA2	21.72659	21.59751	21.58761	0.07754042				2	2	3	4.51E-12	96252000	9	15.6	31.375	
SHMT2	24.40009	24.14235	24.13915	0.14973857	+			11	13	10	4.41E-48	5.47E+08	84	35.6	53.454	
SLC16A1	NaN	21.93837	NaN	N/A				1	2	1	1.17E-13	1.03E+08	10	7.2	46.233	
SLC17A5	22.27213	22.36432	21.79118	0.3077611				5	5	4	1.10E-13	1.58E+08	26	9.3	54.639	
SLC19A1	20.7778	NaN	20.78883	0.00779939				2	1	2	1.35E-05	54581000	9	3.9	64.868	
SLC1A4	21.72733	22.25903	21.75348	0.29971362				4	3	4	4.31E-23	1.36E+08	16	17.7	55.722	
SLC1A5	26.41437	25.95987	25.92345	0.27352608				14	12	14	1.12E-218	2.31E+09	223	35.3	56.598	
SLC25A11	21.29081	21.17255	NaN	0.08362245				3	5	1	5.51E-15	70193000	14	19.9	32.182	
SLC25A22	NaN	19.75369	19.68685	0.04726302				1	2	2	0.001487	17154000	2	9.6	34.47	
SLC25A24	19.70477	19.88728	NaN	0.12905406				3	3	1	3.71E-10	35974000	6	11.1	51.354	
SLC25A3	23.97518	23.75776	23.68688	0.15022889				3	4	3	1.31E-19	5.6E+08	45	19.7	39.958	
SLC25A4	NaN	21.41895	NaN	N/A	+			1	2	1	4.96E-48	59818000	10	36.2	33.064	
SLC25A5	26.64828	26.55139	26.54413	0.05814866				12	12	10	8.87E-68	3.07E+09	207	42.3	32.852	
SLC25A6	23.11953	22.96971	23.44164	0.24114961				3	3	3	6.07E-53	2.81E+08	32	41.9	32.866	
SLC26A11	19.66404	19.9869	20.05616	0.20928196				3	2	2	1.35E-12	27998000	8	6.6	65.298	
SLC27A4	NaN	19.62788	NaN	N/A				1	2	1	0.000581	12615000	2	3	72.063	
SLC29A1	NaN	20.70995	20.67788	0.02267691				1	2	2	4.38E-15	44667000	7	9.4	50.219	
SLC30A1	23.59058	23.46998	23.15945	0.22242838	+			5	7	7	2.42E-49	3.63E+08	36	19.7	55.299	
SLC35B2	25.65051	25.73629	25.63699	0.05385395				12	12	11	1.41E-51	1.81E+09	159	33.7	42.127	
SLC35F6;C2orf18	21.46004	21.34158	21.39869	0.05924265	+			2	2	2	1.31E-17	67738000	7	5.7	40.214	
SLC38A1	23.38034	23.62299	23.79193	0.20689211				5	6	4	2.42E-37	4.13E+08	34	10.7	54.047	
SLC38A2	24.89333	24.74571	24.43619	0.23329926				4	5	4	3.92E-116	8.95E+08	80	17.6	56.025	
SLC39A10	NaN	21.12016	20.27299	0.59903965				2	3	4	1.34E-08	65422000	11	4.5	94.131	
SLC39A14	21.53312	21.11401	21.16208	0.22935947				2	2	2	7.43E-09	41097000	5	21.3	17	
SLC3A2	21.97879	21.94131	21.97649	0.02100664				6	6	3	8.84E-24	1.36E+08	25	15.7	57.944	
SLC41A3	21.06046	21.00881	21.06723	0.03195427				2	3	2	3.59E-19	1.03E+08	17	30.3	16.43	
SLC44A1	26.04005	25.99389	26.14079	0.07512091	+			14	16	15	5.52E-105	2.31E+09	198	28.3	73.301	
SLC44A2	22.86576	22.71994	22.7648	0.07468692	+			4	4	3	1.85E-18	2.18E+08	28	8.5	79.845	
SLC5A6	22.59318	22.3306	22.14635	0.22455637				3	3	2	7.31E-15	1.66E+08	21	8.5	68.641	
SLC7A1	23.77545	23.64702	23.74817	0.06766312	+			6	5	7	3.35E-89	4.67E+08	33	14.8	67.638	

SLC7A2	23.2815	23.05192	22.87542	0.20361737			6	6	6	8.12E-34	2.85E+08	22	16.1	71.672	
SLC7A5	21.89992	22.24394	22.07865	0.17205375			4	2	4	4.19E-17	1.49E+08	19	11.2	55.01	
SLC9A6	21.30975	21.60046	21.09526	0.25355647			4	4	4	4.50E-22	1.04E+08	16	12.3	72.259	
SMS	22.46453	22.16091	22.03701	0.21996578			4	3	2	2.30E-10	1.38E+08	20	14.7	35.278	
SNAP23	25.84045	25.94481	25.94985	0.06175863			12	12	10	3.56E-114	1.99E+09	175	76.3	23.354	
SNRNP70	22.97281	23.31036	22.81898	0.25134902			6	5	6	3.76E-20	2.42E+08	22	29.7	51.556	
SNRPD2	NaN	20.96607	NaN	N/A			3	4	3	2.73E-09	58707000	10	31.4	13.527	
SNX5	NaN	NaN	19.30332	N/A			0	1	1	2.74E-05	7600600	0	4.8	26.304	
SORT1	NaN	NaN	20.7666	N/A			3	1	2	7.07E-15	51099000	15	8.1	92.067	
SPAG1	NaN	19.21582	NaN	N/A			1	2	2	1.89E-06	29277000	7	3.2	103.64	
SPCS1	NaN	NaN	21.05013	N/A			1	2	2	1.23E-11	55094000	7	31.4	11.805	
SPECC1	21.08654	21.53716	21.54567	0.26265667	+		2	3	3	7.44E-09	68971000	8	5.5	79.014	
SPPL2A	21.03181	21.39011	21.45804	0.22900706	+		3	3	3	2.35E-08	79628000	16	6.5	58.143	
SPRY1	21.29143	20.96543	NaN	0.23051681			3	3	2	9.18E-09	53102000	5	22	22.882	
SPRYD7	22.72121	22.84757	22.69878	0.08021681			6	6	5	2.07E-33	2.94E+08	38	45.9	21.666	
SRC	26.71148	26.60978	26.20762	0.26644276	+		17	20	17	2.08E-186	3.11E+09	243	51.5	59.834	
SRI	20.62257	20.73876	20.62203	0.06723875			2	2	2	1.09E-06	49049000	8	19.4	17.605	
SRP68	21.27804	21.44091	21.44516	0.09528361	+		3	4	2	1.48E-10	70576000	9	8.2	60.284	
SRRM2	30.12989	30.07278	30.08765	0.02962793			77	75	75	0	3.55E+10	2305	39.3	299.61	
SRSF11	21.37629	21.17402	21.44278	0.13997977			2	2	2	2.51E-08	95271000	13	10	42.316	
SRSF6;SRSF5;SRSF	NaN	NaN	21.28658	N/A			1	1	2	1.98E-05	42883000	5	5.4	38.418	
ST13;ST13P5;ST1	23.2266	23.23352	23.52755	0.17179078	+		5	5	5	6.50E-32	3.07E+08	44	19.5	41.331	
STBD1	NaN	21.16987	NaN	N/A	+		1	2	1	9.06E-14	70906000	12	16.8	39.007	
STIP1	24.19378	24.16974	24.49552	0.18154777			11	10	9	2.83E-57	6.44E+08	74	30.9	62.639	
STOM	24.37132	24.616	24.18495	0.21618132			9	7	8	3.73E-83	7.8E+08	70	57.3	31.73	
STRAP	20.6651	21.1815	21.22899	0.31275555			4	4	2	5.78E-13	78375000	12	16.3	38.438	
STT3A	22.65401	22.64537	22.59941	0.02934885			5	6	6	2.44E-24	2.45E+08	32	10.4	80.529	
STUB1	NaN	NaN	20.67452	N/A			0	1	2	1.55E-05	16689000	2	7.6	34.856	
STX10	20.87493	20.20848	20.67917	0.34254602			3	3	3	4.33E-13	45904000	9	70.7	6.3891	
STX12	23.92651	23.69083	24.18601	0.24768547			6	5	5	1.30E-55	6.07E+08	74	35.5	31.642	
STX4	NaN	20.29041	NaN	N/A	+		1	2	1	7.74E-05	19896000	2	6.8	33.84	
STX6	20.99755	21.15845	21.30597	0.15425836			3	2	4	1.11E-22	68112000	12	25.1	29.176	
STX7	24.93405	24.77555	25.05565	0.14045451			8	8	7	5.92E-173	1.06E+09	101	46.9	27.4	
STX8	22.87802	22.5807	23.18215	0.30073143			4	7	3	6.42E-28	3E+08	43	45.3	26.906	
SUCLA2	19.31882	19.18899	NaN	0.09180367			2	2	0	8.86E-05	11721000	2	4.7	43.841	
SURF4	23.21242	22.85681	23.8468	0.50149388	+		4	4	3	4.72E-28	4.23E+08	97	39.8	21.127	
SVIP	24.34658	24.12715	24.3908	0.14119505	+		2	2	2	8.99E-31	6.4E+08	63	32.5	8.4426	
SYNCRIP;HNRNPR	19.92024	20.0466	NaN	0.08935001			3	2	1	7.30E-08	31316000	9	6.8	58.735	
SYNGR2	22.77378	22.39132	NaN	0.27044006			4	3	1	1.06E-10	2.24E+08	27	16.1	24.81	
TAGLN2	25.36493	25.2382	25.58245	0.17410892			9	10	9	3.16E-65	1.47E+09	145	80.4	22.391	
TALDO1	24.17737	24.51147	23.91762	0.29769971			8	9	9	1.04E-38	6.09E+08	81	35	37.54	
TARS	22.66965	22.65147	22.53512	0.07299104			8	8	8	1.24E-26	2.12E+08	45	14.7	83.434	
TCP1	24.66682	24.27085	24.33674	0.21216603			14	16	12	1.25E-68	6.05E+08	99	50.2	60.343	
TECR	21.7681	21.94685	21.95123	0.10448871	+		3	4	3	4.07E-10	1.03E+08	10	11.7	36.034	
TESC	22.03258	21.66427	22.28082	0.31021747	+	+	4	3	3	1.32E-21	1.42E+08	21	34.8	21.521	
TFRC	27.63437	27.49098	27.44876	0.09729191			28	29	28	1.90E-297	5.38E+09	446	51.3	84.87	
TKT	23.74314	23.95536	23.54392	0.20575423			8	9	6	3.65E-33	5.03E+08	50	26.2	62.878	
TLCD1	19.36027	NaN	NaN	N/A	+		2	1	1	0.000198	20241000	4	10.9	28.548	
TLDC1	24.24104	24.26263	24.30765	0.03398485	+		9	9	7	5.97E-70	7.67E+08	92	36.4	50.993	
TLN1	21.65652	21.21703	21.66206	0.25535395			6	5	4	2.00E-13	76736000	11	3.7	258.08	

TM9SF3	22.43665	22.19541	22.41284	0.13294075			4	4	4	1.12E-13	1.66E+08	27	8.3	67.887	
TM9SF4	21.47234	21.20288	NaN	0.19053699			3	3	1	1.53E-11	51369000	9	7.6	74.518	
TMEM106B	25.35269	24.99434	25.03842	0.19541554	+		9	9	6	5.78E-78	1.07E+09	78	36.9	31.127	
TMEM106C	22.27168	22.40963	22.43122	0.08655378	+		3	2	2	1.97E-19	1.78E+08	20	14.4	27.875	
TMEM115	21.75128	22.54692	22.62391	0.48312414			3	2	3	7.90E-18	1.06E+08	12	14.2	38.197	
TMEM168	22.52946	22.81382	22.47954	0.18032181			6	6	6	2.84E-33	2.24E+08	29	15.5	79.754	
TMEM179B	23.28743	23.16153	23.30925	0.07973719			3	3	3	1.01E-15	2.7E+08	32	15.5	23.55	
TMEM181	22.11474	21.93837	21.43731	0.35144438	+		2	3	3	8.67E-13	1.33E+08	13	6.5	69.324	
TMEM184B	19.89097	NaN	NaN	N/A			2	1	0	1.31E-05	20337000	5	4.7	45.561	
TMEM184C	NaN	23.22201	22.74618	0.33646262			1	3	3	7.65E-76	2.24E+08	13	9.6	50.141	
TMEM192	20.97449	20.69905	20.6664	0.1692398			2	2	2	4.37E-16	69731000	12	21.7	30.549	
TMEM222	NaN	NaN	21.42568	N/A			1	1	2	1.01E-12	70079000	9	24.6	19.862	
TMEM33	22.25874	23.00974	22.59687	0.37611934			4	3	3	1.04E-23	2.05E+08	41	23.5	27.978	
TMEM50A	23.28954	22.76709	23.1733	0.27430892			3	2	3	5.78E-18	2.8E+08	32	29.3	17.4	
TMEM50B	21.92484	21.49585	21.96109	0.25877749			2	2	2	1.31E-08	93251000	14	15.2	16.854	
TMEM55A	21.20848	21.32789	21.12868	0.10025917			2	2	2	1.33E-07	65789000	10	11.7	28.081	
TMEM55B	23.15392	23.03455	23.10583	0.06005925	+		5	5	5	2.44E-23	3.01E+08	36	26	29.469	
TMEM59	20.86431	21.05953	20.34191	0.37103258			3	3	2	5.39E-11	58491000	3	19.8	21.86	
TMEM63A	21.69417	21.90547	21.43466	0.23581603			3	4	2	4.58E-14	1.03E+08	16	6.3	92.125	
TMEM63B	23.77475	23.45501	23.88558	0.22357232			8	6	7	9.97E-31	3.78E+08	50	11.7	94.957	
TMEM87A	22.02722	21.84533	21.79551	0.12196689			5	5	5	1.28E-19	1.19E+08	14	12.8	56.773	
TMPO	19.93919	20.08563	19.65097	0.22115031			2	2	2	0.000105	30332000	6	8.9	26.866	
TMX1	25.02568	25.07194	25.01482	0.03033321			10	11	9	2.61E-59	1.35E+09	138	32.9	31.791	
TMX3	22.01828	22.05063	22.07685	0.02933842			3	3	3	2.42E-18	1.65E+08	30	18.1	51.871	
TMX4	21.59314	21.66014	21.51581	0.07222659			2	3	2	3.34E-12	1.01E+08	17	13.5	38.952	
TNFRSF10A	24.05887	24.0912	23.67357	0.23234892			6	5	5	6.36E-32	5.39E+08	62	19.2	50.089	
TNPO1	22.38995	22.26734	22.44106	0.08927867			7	6	4	1.34E-30	1.89E+08	41	15.6	101.31	
TOMM40	23.21676	23.08936	23.03026	0.09531161	+		4	3	4	2.17E-68	3.4E+08	26	16.6	37.893	
TOMM70A	20.7778	20.43441	NaN	0.2428134			2	2	1	5.92E-06	39208000	5	3	67.454	
TPD52L2	21.97155	21.79697	21.46732	0.25605832			4	4	5	2.31E-15	92947000	19	25.2	22.237	
TPI1	27.27782	27.24744	27.80888	0.31574318			14	16	14	0	5.69E+09	383	85.9	26.669	
TPT1	22.40927	21.52526	21.63635	0.48152881	+		2	2	3	3.17E-17	1.15E+08	14	25.6	19.595	
TRAP1	23.83123	23.82929	23.69604	0.07749802			10	12	6	2.70E-72	4.74E+08	68	24.9	74.267	
TRAPPC3	24.76436	24.61448	24.82002	0.10630831	+		5	6	5	2.21E-32	9.66E+08	75	41	15.005	
TRIM28	22.2575	21.94892	22.15505	0.15716615			5	5	3	3.54E-41	1.88E+08	37	12.6	88.549	
TSN	21.08537	21.0418	NaN	0.03080864			2	2	1	3.71E-07	53435000	12	13.5	21.01	
TSPAN13	22.8341	23.47321	22.84518	0.36583376			5	5	3	8.27E-20	2.46E+08	28	26	22.147	
TSPAN14	22.1084	22.21721	22.64729	0.28495931			3	2	2	1.18E-11	1.52E+08	20	14.6	28.876	
TSPAN3	27.18952	26.82953	27.58276	0.37673729	+		5	5	4	4.69E-52	4.32E+09	77	38.6	25.183	
TSPAN6	25.44922	25.52752	25.46008	0.04242048			7	7	7	2.38E-64	1.77E+09	128	37.1	27.563	
TSPAN7	21.67508	21.36169	21.46034	0.16023856	+		2	2	2	1.34E-17	93774000	11	11.2	24.475	
TSPAN9	21.07783	21.84015	NaN	0.53904164			2	2	1	5.39E-13	93473000	21	20.1	26.779	
TTYH3	23.93238	23.8374	23.60964	0.16585984			5	5	6	2.75E-24	4.84E+08	53	17.1	54.139	
TUBA1B;TUBA4A	28.02902	27.80514	27.99647	0.12096069	+		18	18	16	0	9.77E+09	672	59.9	50.151	
TUBA1C;TUBA1B	NaN	22.42145	NaN	N/A	+		1	2	1	0	1.88E+08	28	60.1	49.895	
TUBB	25.59146	25.5697	25.62656	0.02868962			5	5	5	0	1.74E+09	133	58.2	47.766	
TUBB2A	20.38585	20.81941	NaN	0.30657322			3	3	1	0	52888000	15	47	49.906	
TUBB4B;TUBB4A	27.73789	27.49396	27.71371	0.13439778			19	19	20	0	7.07E+09	705	59.6	49.83	
TUBB8	NaN	22.63318	NaN	N/A			1	1	1	8.60E-137	1.79E+08	8	24.3	49.775	
TUFM	22.35574	22.332	22.66856	0.18783529			7	6	3	2.31E-26	1.91E+08	34	18.6	49.541	

TUSC2	NaN	20.17646	NaN	N/A	+		2	2	1	3.03E-05	44492000	6	19.1	12.074	
TXN	20.001	NaN	NaN	N/A			2	1	2	3.84E-08	60190000	14	36.5	9.4519	
TXNDC5	20.23397	20.90485	NaN	0.4743838			3	3	1	1.91E-12	51511000	12	16	36.177	
TYMS	19.08124	19.23292	NaN	0.10725396			2	2	1	7.72E-05	20331000	5	7.2	31.758	
UBA1	25.93429	25.7892	25.52949	0.20508674	+		22	23	18	2.88E-151	1.91E+09	205	37.9	117.85	
UBB;RPS27A;UBC	25.61944	25.39333	25.80137	0.20441824			4	4	4	4.96E-69	1.89E+09	149	50.5	10.469	
UBE2I	NaN	21.35219	NaN	N/A			0	2	0	2.17E-09	54769000	11	32.9	7.8891	
UBE2K	NaN	NaN	20.40145	N/A			0	1	2	3.48E-05	23733000	4	18	15.81	
UBE2L3	NaN	20.66986	NaN	N/A			0	2	1	7.75E-06	39457000	5	34.4	14.121	
UBE2M	22.11851	22.44726	22.22864	0.16733195			5	6	4	3.89E-13	1.29E+08	17	35.5	20.9	
UBE2V1;TMEM18	21.3721	21.65661	NaN	0.20117895			3	2	1	2.16E-10	73551000	16	24.5	16.495	
UBTD2	21.88907	21.78333	22.29112	0.2679166	+		3	3	3	7.70E-28	1.23E+08	29	24.4	26.189	
UCHL1	24.70852	24.81245	24.55818	0.12783896			7	7	7	2.42E-73	1.13E+09	117	59.6	24.824	
UQCRC1	21.45704	21.48075	NaN	0.0167655			3	4	1	1.12E-13	85573000	11	15.4	52.645	
UQCRQ	23.88558	24.40425	23.84709	0.31116108	+		4	4	4	2.33E-24	5.59E+08	102	74.4	9.9062	
USMG5	21.50145	21.36661	21.26287	0.11962736			2	2	2	1.37E-06	76824000	6	43.1	6.4575	
USP12;USP46	20.61144	20.6796	20.77233	0.08075707			2	2	2	1.09E-07	56852000	8	10.5	42.857	
USP32	20.22635	20.42316	20.65753	0.21586248	+		3	4	3	4.55E-12	43917000	8	3.8	181.65	
USP7	22.95507	22.95227	22.55265	0.23153324			6	7	4	1.18E-32	3.38E+08	40	13.5	117	
USP9X	19.74751	19.57352	20.19063	0.31818564			2	2	2	1.67E-05	22603000	6	0.9	290.46	
VAMP3;VAMP2	25.22218	24.61313	25.26112	0.36339814			2	2	2	1.86E-157	1.28E+09	43	40	11.309	
VAMP7	24.99088	24.58658	24.83016	0.20356024			7	8	9	1.13E-47	9.21E+08	82	45.5	24.935	
VANGL1	21.14868	NaN	NaN	N/A			2	1	1	6.72E-13	69084000	7	12.3	59.747	
VAT1	20.89052	21.04247	NaN	0.10744488			2	2	1	3.82E-10	63337000	7	19.6	41.92	
VCL	24.73872	24.56964	24.70372	0.08924731			17	17	19	2.16E-84	7.96E+08	139	29.1	116.72	
VCP	23.02332	22.8369	22.88407	0.09692608			8	9	8	4.53E-46	2.43E+08	50	16.1	89.321	
VDAC1	22.15249	22.43296	22.47516	0.17538538			3	3	2	4.21E-21	1.92E+08	37	23	30.772	
VDAC2	26.56571	26.27647	26.19378	0.1952901			11	12	10	1.60E-124	2.61E+09	161	61.6	31.566	
VDAC3	23.52385	23.05636	22.85083	0.34490226			3	4	3	8.87E-26	2.97E+08	32	22.3	30.658	
VPS35	NaN	21.44273	21.22199	0.15608675			2	3	3	2.85E-13	52547000	10	6.7	91.706	
XPO1	24.43288	24.35414	24.30313	0.065367			13	17	13	2.45E-56	7.89E+08	72	22.7	123.38	
XPO5	20.08343	19.89352	20.3888	0.24987294			3	3	2	1.23E-08	34216000	8	2.7	136.31	
XPOT	20.58912	NaN	NaN	N/A			2	1	1	3.79E-12	29849000	9	5.3	109.96	
XRCC5	23.9693	23.94655	23.81695	0.08218297			12	15	8	8.52E-59	5.16E+08	64	25.3	82.704	
XRCC6	24.06044	23.75379	24.16745	0.21470909			7	8	7	1.79E-51	5.25E+08	71	29.9	69.842	
XXYLT1	20.16251	19.97071	20.04367	0.09681025	+		2	2	2	2.54E-06	33447000	7	13.9	21.816	
YARS	21.73322	21.85426	NaN	0.0855882	+		6	6	3	1.69E-18	1.02E+08	19	14.4	59.143	
YES1	23.28263	23.39192	23.4665	0.09247942	+		7	4	4	6.69E-79	4.32E+08	52	43.3	60.801	
YWHAB	25.08772	24.86755	24.85771	0.13004887			6	5	6	1.99E-123	1.15E+09	78	48.4	27.85	
YWHAE	26.54391	26.60159	26.52953	0.03813662			11	13	12	4.56E-90	3.48E+09	215	61.2	29.174	
YWHAG	21.51225	21.0995	21.39514	0.21271277			4	4	3	1.59E-39	1.1E+08	25	35.2	28.302	
YWHAH	22.48333	23.08434	22.51756	0.33754612	+		4	3	4	4.43E-29	2.08E+08	27	26.8	28.218	
YWHAQ	23.84307	23.83942	23.30883	0.30739536			6	6	6	1.08E-99	5.14E+08	81	45.7	27.764	
YWHAZ	25.89502	25.64064	25.98826	0.17992709			10	10	10	3.32E-163	2.28E+09	171	53.1	27.745	
ZDHHC13	23.92289	23.55133	23.61807	0.19808512			7	6	6	6.79E-36	4.11E+08	46	14.8	70.86	
ZDHHC17	21.17329	21.16017	NaN	0.00927724	+		2	3	1	5.99E-11	44400000	6	5.7	72.639	
ZDHHC18	22.03725	21.90628	22.07058	0.08685094			6	4	4	1.32E-22	1.56E+08	24	20.6	42.03	
ZDHHC20	21.71716	20.76991	20.88658	0.51652002			2	3	2	4.93E-15	82370000	14	17.3	42.277	
ZDHHC3	21.65368	22.21184	21.71607	0.30583846			2	2	2	3.38E-14	1.21E+08	10	9	34.17	
ZDHHC4	21.36341	21.10981	21.40083	0.15832764			3	3	3	6.89E-07	80415000	8	11.6	39.786	

ZDHHC5	21.96406	22.15774	22.15041	0.10976641			4	5	2	1.03E-23	1.58E+08	29	14.7	71.951	
ZDHHC6	22.97636	23.0969	22.68166	0.21362047	+		6	7	5	1.94E-30	2.79E+08	35	18.8	47.205	
ZMPSTE24	21.81874	21.48055	21.58646	0.17298526	+		2	3	2	2.57E-07	82939000	18	5.5	54.812	
ZNF341	NaN	28.60925	NaN	N/A			0	1	0	0.001916	1.34E+09	2	3	92.046	
ZNRF2	23.09405	21.67767	22.48355	0.7104324	+		3	5	3	2.14E-28	2.54E+08	27	59.5	24.115	

Reagent 4

Gene names	log2 LFQ intensity_1	log2 LFQ intensity_2	log2 LFQ intensity_3	St dev log2 LFQ	MG protein	PTM peptide	Razor + unique peptides_1	Razor + unique peptides_2	Razor + unique peptides_3	PEP	Intensity	MS/MS Count	Sequence coverage [%]	Mol. weight [kDa]	
AARS	21.5467	21.31772	21.2204	0.167518513			3	3	2	3.06E-15	77577000	13	5.9	106.81	
AASDHPPT	20.66101	20.51893	20.5721	0.071785286			2	2	2	5.46E-06	19014000	7	7.4	35.776	
ABHD17A	22.73647	22.85102	NaN	0.080999082			2	2	1	1.06E-22	2.79E+08	24	33.5	33.989	
ABHD17B	22.40971	22.44675	22.1763	0.146626164			4	5	3	7.99E-26	3.29E+08	22	34	32.214	
ABL2	22.63034	22.60098	22.56486	0.032798106	+		6	7	6	1.43E-35	2.96E+08	46	12.6	115.82	
ACAA2	20.66414	20.98628	20.82524	0.161070001	+		2	2	2	1.25E-15	53733000	8	14.6	41.924	
ACACA	27.35649	26.70649	27.01967	0.325071639			49	48	50	5.68E-188	2.45E+09	314	31	265.55	
ACAT1	NaN	21.04847	21.06178	0.009411591			1	2	2	3.69E-18	87102000	17	21.8	45.199	
ACOT7	20.99362	NaN	20.82135	0.121813285			3	1	2	4.67E-09	56625000	11	14.2	27.041	
ACOT9	23.21898	23.02591	23.0148	0.114810663			7	7	8	4.59E-33	3.51E+08	54	31.3	46.354	
ACTG1;ACTB;ACTA1	28.44273	28.36995	28.56747	0.099892564			18	18	18	0	1.28E+10	601	67.5	41.792	
ACTN4;ACTN1	20.59478	20.66752	20.93603	0.179740688	+		2	2	3	5.49E-17	75173000	11	9.9	104.85	
AGPAT1	20.99673	21.3224	21.20156	0.164630183			3	4	2	4.89E-11	1.12E+08	30	35.1	18.756	
AHCY	24.41306	24.49186	24.21823	0.140855449			7	10	9	3.52E-54	9E+08	113	34.3	47.716	
AIFM2	24.15846	23.90784	23.82007	0.175604551	+		7	7	5	1.32E-58	7.66E+08	64	44.5	40.526	
AKAP12	27.55976	27.21854	26.43598	0.576152863	+		31	27	24	0	4.84E+09	455	37.4	191.48	
AKR1B1	21.45734	21.35526	21.24853	0.104413629			3	3	3	3.17E-08	93513000	7	11.1	35.853	
ALDH18A1	22.55796	22.4265	22.91304	0.251689196			4	6	5	5.75E-27	2.24E+08	48	14.9	87.088	
ALDH2	NaN	19.03757	NaN	N/A			1	2	1	1.13E-08	20215000	8	7	56.381	
ALDH7A1	22.65318	22.72642	22.73442	0.04477357	+		4	6	5	1.96E-21	2.11E+08	19	21.5	55.366	
ALDH9A1	21.22517	21.19352	21.38858	0.10468441			4	4	3	1.39E-15	91824000	15	13.2	53.801	
ALDOA	25.92608	26.21653	25.9268	0.167483926			16	16	14	5.06E-187	3.01E+09	221	68.4	39.42	
ALDOC	22.87938	23.01364	22.38798	0.329380768			1	1	1	5.87E-18	2.18E+08	13	8.2	39.455	
ANO6	21.03308	20.97694	21.35176	0.20215451			2	3	3	6.48E-20	1.17E+08	21	11.8	103.96	
ANP32A	22.40328	22.33514	22.77129	0.234627805			5	4	4	3.14E-20	1.67E+08	16	29.4	19.997	
ANP32B	NaN	21.63281	22.04896	0.294262487			1	2	2	5.50E-16	85008000	10	17.9	22.276	
ANXA1	23.51737	23.60298	23.2967	0.158025188			7	9	8	5.45E-105	6.1E+08	89	54.3	38.714	
ANXA11	21.86869	21.94521	21.87594	0.042241776			4	4	4	2.02E-11	1.09E+08	26	8.5	51.242	
ANXA2;ANXA2P2	27.01744	27.04469	26.46421	0.327557381	+		18	17	17	6.34E-165	2.65E+09	241	64.3	38.604	
ANXA5	28.51559	28.52373	29.08507	0.326464987			20	20	20	4.45E-256	6.82E+09	647	76.6	35.936	
ANXA6	25.06562	24.9003	24.71356	0.176138569			16	17	13	2.41E-58	6.57E+08	106	42	75.276	
ANXA7	21.20425	21.31877	20.93984	0.194343092			2	2	2	8.89E-12	79585000	13	18.5	37.805	
AP1B1	22.6904	22.54387	22.21525	0.243320662			3	3	3	2.23E-21	1.48E+08	32	10.9	101.35	
API5	23.12755	23.09934	22.5591	0.320361895			5	6	5	4.29E-71	2.21E+08	36	21.8	50.309	
APMAP	22.18226	22.21454	21.93096	0.155247815			4	5	4	1.73E-21	2.41E+08	24	18.3	46.48	
APRT	23.1001	23.43505	22.9087	0.266417525			5	4	5	6.05E-23	3.19E+08	45	49.4	19.608	
ARCN1	20.30952	20.25165	20.38922	0.069073067			2	3	2	6.23E-10	44299000	10	8	57.21	
ARF1	30.23729	30.24943	30.24774	0.00657569	+	+	12	12	14	0	6.23E+10	761	79.6	20.697	
ARF4	27.13956	27.04573	27.0379	0.056568739	+	+	7	6	7	0	7.01E+09	255	86.7	20.511	
ARF5	25.72489	25.52883	25.87963	0.175805115	+		5	6	6	0	2.77E+09	142	71.1	20.529	
ARF6	25.1493	25.03158	25.01517	0.073164362	+		5	5	5	2.11E-78	2.14E+09	117	60	20.082	
ARL1	26.2727	26.15021	25.8009	0.244818476	+		10	10	8	0	4.38E+09	238	80.5	18.565	
ARL13B	19.79149	20.31241	NaN	0.368346064			2	2	1	2.82E-06	42032000	11	8.3	37.086	
ARL5B	21.90496	22.01716	21.81445	0.101548219	+		2	3	2	2.83E-21	2.38E+08	23	21.2	20.374	
ATP1A1	24.64438	24.42291	24.37836	0.142478246	+		15	13	9	3.17E-92	8.85E+08	116	24.8	113	
ATP2A2	23.41338	23.58978	23.1888	0.200971846	+		10	12	8	1.20E-60	4.56E+08	66	21	114.76	

ATP5A1	22.95161	23.01456	22.78662	0.117715084			9	9	10	7.15E-77	7.08E+08	92	40.7	59.75	
ATP5B	NaN	21.6787	NaN	N/A			1	3	2	1.30E-18	1.68E+08	15	24.6	38.138	
ATP5H	22.95709	23.41351	22.71404	0.355117539			4	3	3	2.04E-20	2.06E+08	18	74.5	15.773	
ATP5O	24.13242	23.69445	23.53469	0.309467858			5	6	5	2.48E-24	3.62E+08	48	34.7	23.277	
ATP6V0A2	21.03241	21.24824	NaN	0.152614857	+		2	2	1	3.84E-20	80622000	8	9.2	98.081	
ATP6V1A	20.56867	NaN	NaN	N/A			3	2	2	1.00E-20	61686000	12	8.9	64.735	
ATP9A	22.75277	22.89245	22.99335	0.120809801			7	8	6	8.96E-41	3.19E+08	59	12.7	118.58	
ATXN10	NaN	NaN	20.63768	N/A			1	1	2	5.06E-09	37702000	7	13.6	46.286	
AUP1	21.49795	21.64733	21.57736	0.074739697			2	2	2	4.57E-10	1.02E+08	8	9.4	41.385	
B3GNT1	21.53602	21.86582	22.28139	0.37350656			2	4	2	8.47E-30	2.39E+08	42	33.3	47.119	
B4GALT1	NaN	NaN	21.53212	N/A			1	1	2	1.14E-05	34452000	10	4.9	42.537	
BAG5	22.20386	22.3118	22.11984	0.096228067			4	5	5	3.83E-39	2.43E+08	31	33.3	51.199	
BAG6	NaN	21.43013	NaN	N/A			2	4	2	4.22E-10	44693000	9	6.4	118.69	
BASP1	22.17767	20.04514	23.34015	1.671134089	+		5	2	4	2.37E-200	1.93E+09	95	57.3	22.693	
BAX	22.48591	22.67381	21.85175	0.430745083			3	3	3	2.50E-44	1.69E+08	36	36	18.129	
BCAM	21.81378	22.01274	21.38611	0.320195739			3	3	3	1.08E-32	1.9E+08	33	21	67.404	
BCAP31	22.50312	22.51617	22.04059	0.270887627	+		3	3	3	5.54E-18	2.15E+08	35	19.5	27.991	
BET1L	20.94056	21.12849	20.91254	0.117428849			2	2	2	6.35E-10	92362000	11	48.3	6.4651	
BZW1	21.14886	21.54269	20.83653	0.353862978			2	3	2	4.91E-10	61522000	18	10.2	40.538	
C11orf48	24.38271	23.44708	24.29796	0.517458989	+		2	2	2	6.25E-11	2.61E+08	20	21.2	11.6	
C12orf23	24.69694	24.52803	24.6705	0.090854612			5	5	5	2.14E-28	4.19E+08	61	62.9	11.748	
C1QBP	NaN	22.08005	NaN	N/A			1	3	0	2.97E-23	1.14E+08	13	26.2	31.362	
C9orf123	23.77314	24.5254	24.08302	0.378069832	+		3	3	3	3.61E-57	6.18E+08	36	50.9	11.829	
CALR	NaN	21.69451	NaN	N/A			1	2	0	6.29E-16	81862000	10	12.9	48.141	
CAND1	24.80114	24.7902	24.62064	0.101201554			22	23	25	2.82E-106	8.89E+08	132	31.7	136.37	
CANX	28.25885	28.39705	28.05217	0.173569427			20	21	20	0	1.84E+10	767	44.9	67.567	
CAP1	21.51696	21.72862	21.68594	0.11193434			2	2	2	9.88E-15	1.11E+08	18	13.7	51.83	
CAPNS1;CAPNS2	20.99728	21.15956	20.89652	0.132713612	+		2	2	2	2.54E-10	78085000	15	37	11.399	
CAPZB	22.12348	22.08469	22.11248	0.019991449			4	3	5	2.22E-13	1.62E+08	25	25.4	29.295	
CAV1	23.79184	24.17805	24.09394	0.203099756			5	5	5	1.42E-67	5.71E+08	75	47.2	20.471	
CAV2	NaN	21.15493	NaN	N/A	+		0	2	1	8.33E-08	55929000	11	18.8	16.828	
CBR1	20.00292	20.06737	20.14058	0.068876438			1	2	1	1.04E-09	40273000	8	23.1	18.762	
CCNY	23.4837	23.1369	24.05184	0.461910616	+		7	7	8	1.95E-44	5.2E+08	52	47.5	39.336	
CCT2	24.38515	24.12068	24.25465	0.132238794			9	9	11	4.36E-88	6.61E+08	84	42.1	57.488	
CCT3	23.72349	23.8641	24.28263	0.290853936			7	8	5	7.39E-80	6.01E+08	89	40	55.674	
CCT4	24.47846	24.03691	24.70229	0.338574663			11	14	16	2.70E-75	7.68E+08	105	55.8	57.924	
CCT5	22.5413	22.70126	22.54538	0.091197974			8	7	7	5.33E-38	2.72E+08	48	22.6	59.67	
CCT6A	23.62956	23.47246	23.56628	0.079043196			8	11	10	5.77E-76	4.41E+08	63	29.6	58.024	
CCT7	23.67109	23.56186	23.89393	0.169243091			9	10	8	4.56E-49	4.72E+08	57	33.1	54.804	
CCT8	25.07851	24.83562	24.87942	0.12945451			19	19	21	6.05E-87	1.16E+09	182	46.5	59.62	
CD151	26.63034	26.96576	26.48843	0.245115263	+		5	5	5	2.39E-50	3.56E+09	181	15.1	28.067	
CD276	25.92302	25.72915	25.5786	0.172663457			7	8	7	2.83E-258	1.83E+09	130	37	57.165	
CD44	24.14647	24.33381	23.74088	0.303085661			5	5	5	4.97E-34	7.63E+08	94	33.5	22.683	
CD47	21.09513	20.84445	21.19424	0.180285098			2	2	2	7.11E-07	55427000	10	9.6	31.742	
CD55	NaN	19.30084	NaN	N/A			1	2	1	0.000402	19435000	4	5.2	35.695	
CD63	27.02865	27.57766	26.68779	0.448974041			7	7	5	4.99E-212	5.65E+09	157	33.5	23.43	
CD81	28.53282	28.68852	28.4287	0.13076053	+		4	4	4	0	1.21E+10	235	50.3	17.963	
CD9	25.74963	25.98713	26.21386	0.232135821			5	5	4	4.90E-220	2.91E+09	196	42.8	17.764	
CDC37	22.39354	22.21611	22.10946	0.143502075			3	3	2	5.76E-15	1.53E+08	26	15.3	44.468	
CDC42	21.22529	21.67896	21.21886	0.263802269			2	2	2	6.27E-09	67238000	14	25.7	21.258	

CDCA3	24.84652	24.69943	24.5994	0.124304574	+		7	10	5	3.67E-87	9.61E+08	98	52.2	28.998	
CDK1	22.89047	NaN	22.72723	0.115428111	+		2	1	2	2.39E-13	3.91E+08	33	26.5	21.738	
CECR5	21.25931	21.50799	21.19105	0.166809339			4	2	3	7.30E-13	82416000	13	13.5	43.588	
CERS2	20.88569	20.5871	20.74575	0.149392667			2	3	2	2.17E-11	77216000	9	13.8	36.373	
CFL1	24.32752	24.47544	24.52558	0.102974195			6	7	6	1.87E-62	8.73E+08	67	69.1	16.811	
CHCHD3	26.95216	27.0358	27.01478	0.043510065	+		14	13	14	1.22E-222	5.78E+09	281	46.7	26.152	
CHCHD6	24.47562	25.17486	24.5087	0.39450391	+		5	5	4	3.85E-134	8E+08	72	44.7	26.457	
CHMP6	24.78122	24.89924	24.69136	0.104257401	+		5	5	6	5.52E-146	1.43E+09	94	48.3	23.485	
CHP1	25.36453	25.49433	24.94802	0.285418767	+		8	8	9	5.54E-76	1.33E+09	131	67.2	22.456	
CKAP4	26.92954	26.86124	26.60696	0.169990718			25	25	21	0	5.99E+09	480	57.1	66.022	
CKAP5	20.72592	NaN	20.79003	0.045332616	+		2	1	2	6.97E-11	43521000	11	2.9	218.52	
CKB	24.14398	23.91123	24.01163	0.116739914			6	10	9	7.38E-86	6.36E+08	80	52	42.644	
CLCC1	NaN	21.34294	NaN	N/A			2	3	2	1.32E-15	62104000	12	13.2	56.266	
CLDND1	21.11763	20.86718	NaN	0.177094893	+		2	2	1	1.34E-11	1.19E+08	14	33.3	16.049	
CLIC1	24.0891	24.58015	24.4545	0.255093181			5	6	5	4.85E-41	5.76E+08	69	53.1	26.922	
CLN3	21.67344	21.22176	22.3894	0.588783593	+		3	3	3	2.72E-24	1.31E+08	29	21.5	34.568	
CLTA	21.98562	22.02489	21.55539	0.260470803			2	2	2	5.26E-06	74539000	19	7.8	23.662	
CLTC	23.7006	23.73407	23.44076	0.160555194			15	12	10	1.29E-71	4.23E+08	98	15.8	187.89	
CNN3	21.45914	21.52927	21.19724	0.175001785			3	2	2	3.75E-11	72237000	18	16.3	31.38	
COPB1	NaN	20.95837	21.213	0.1800506			1	3	2	3.85E-10	75607000	16	4.9	107.14	
COPB2	NaN	20.84376	NaN	N/A			1	4	3	4.85E-14	61662000	10	6.8	99.045	
COPG1	21.41875	21.51615	21.69302	0.139040635			2	2	2	8.03E-09	72398000	12	4.9	97.717	
COTL1	20.42859	NaN	20.39728	0.022139513			2	1	2	0.000375	34272000	4	12	15.945	
CPD	24.81617	24.83215	24.38357	0.254500209			22	18	17	7.36E-130	9.89E+08	146	25.1	152.93	
CPNE3	21.66635	21.6497	21.65399	0.008644808			4	5	4	9.04E-14	1.27E+08	22	11.9	60.13	
CS	23.90757	23.91944	23.77726	0.078884668			5	7	6	3.26E-35	6.56E+08	63	24.5	50.431	
CSE1L	26.00425	25.80909	25.08018	0.487049378			19	18	18	5.92E-151	1.44E+09	186	35.7	107.78	
CTNNA1	20.20943	20.37708	20.08822	0.145050845			2	2	2	1.59E-07	30602000	5	5.4	88.676	
CXADR	23.98581	23.8968	23.55918	0.22506414			9	10	8	6.57E-93	7.02E+08	58	40.5	40.029	
CYB5R3	27.71221	27.64849	27.66099	0.033763829	+		17	19	17	0	1.22E+10	574	85.7	31.76	
CYSTM1	NaN	24.21074	23.92588	0.201426438			1	2	2	5.15E-08	4.14E+08	33	11.3	10.631	
DAD1	22.26942	22.58474	22.06891	0.260035751			3	3	2	4.48E-11	1.64E+08	21	35.3	9.5541	
DAGLB	24.62076	24.77831	24.42137	0.178878235			10	12	8	1.15E-81	1.08E+09	139	24.7	73.731	
DCAF11	21.01964	20.41256	NaN	0.429270385	+		3	2	1	2.45E-13	86374000	12	12.3	58.846	
DDB1	NaN	20.91612	20.65325	0.18587716			1	4	3	2.28E-15	76194000	19	5.6	121.71	
DDX17	21.46797	21.35391	21.93012	0.305125731			4	4	4	2.65E-21	99665000	25	14	72.371	
DDX39B;DDX39A	23.85034	23.71083	23.75073	0.071853093	+		10	10	7	6.14E-49	5.33E+08	82	29.9	48.991	
DDX46	24.23001	24.12747	24.62717	0.263928844	+		14	14	14	2.70E-41	4.62E+08	87	17.4	117.36	
DEGS1	23.35474	23.47853	23.62723	0.136434633	+		3	3	3	2.35E-38	6.33E+08	55	15.8	37.866	
DHCR7	22.33331	21.87455	21.7905	0.292166572	+		4	5	3	3.17E-16	1.4E+08	33	12	54.489	
DHX15	21.58742	21.2948	21.54755	0.158691863			3	6	4	5.54E-17	87879000	18	8.3	90.932	
DHX9	21.94967	NaN	NaN	N/A	+		4	2	3	6.18E-22	1.04E+08	21	8.3	140.96	
DIRC2	22.74593	22.67502	NaN	0.050140942	+		2	2	1	6.06E-22	2.22E+08	16	10.5	44.145	
DLAT	NaN	20.80049	NaN	N/A			1	2	0	7.86E-06	32341000	5	4.8	57.586	
DNAJC5	26.19695	26.20277	25.9862	0.123390977			8	8	7	9.45E-289	3.38E+09	275	54.5	22.149	
DNAJC7	20.62908	NaN	NaN	N/A			2	1	1	7.58E-15	47893000	17	20.6	28.704	
DNM1L	22.10399	NaN	NaN	N/A			2	1	1	9.62E-12	79167000	13	6.6	78.099	
DSG2	21.26247	21.53886	20.99811	0.270397302			2	2	2	7.38E-17	87772000	17	6.6	122.29	
DSG4	NaN	22.43568	NaN	N/A			0	2	0	2.14E-10	23450000	3	1.8	113.82	
DSP	NaN	20.89586	19.12098	1.255029684			1	12	3	1.73E-53	1.89E+08	36	9.3	331.77	

DTYMK	NaN	20.61126	20.47417	0.096937269			1	2	2	0.001026	29337000	3	9.4	19.368	
DUT	NaN	20.62578	NaN	N/A			1	2	1	5.14E-13	53319000	14	36	17.748	
DYM	23.26556	22.99745	23.67799	0.34281097	+		9	9	8	3.98E-34	3.99E+08	68	18.1	75.935	
EBP	21.12187	22.44094	21.18701	0.743474874			2	2	2	3.35E-07	1.46E+08	21	9.6	26.352	
ECE1	21.05126	20.636	20.22294	0.414160487			2	2	2	1.73E-07	62583000	12	3.3	85.561	
ECHS1	23.25609	23.56697	23.26026	0.178295068			5	4	5	3.25E-21	2.96E+08	41	24.8	31.387	
EEF1A1P5;EEF1A1	27.28666	27.49747	27.34571	0.108749602	+		13	14	13	2.69E-171	7.65E+09	406	42.6	50.184	
EEF1B2	22.82613	23.13011	22.59703	0.267415076	+		4	3	3	1.50E-38	2.75E+08	28	24.4	24.763	
EEF1D	22.34838	22.46226	22.29942	0.083548989			5	5	5	1.30E-24	1.67E+08	29	31.8	28.821	
EEF1G	24.87919	24.97557	25.06727	0.094049704			10	11	12	1.22E-122	1.47E+09	158	47.6	50.118	
EEF2	26.79872	26.98087	26.71398	0.136375561			35	36	33	2.09E-242	5.11E+09	440	54.8	95.337	
EFCAB14	NaN	20.99879	NaN	N/A			1	2	1	2.43E-13	38747000	8	10.7	55.031	
EFR3A	NaN	20.81167	22.34082	1.081272334			1	3	2	1.30E-41	2.06E+08	38	21.3	92.923	
EIF2B1	21.57464	21.73748	22.02834	0.229840559			2	2	2	2.31E-13	68937000	17	17.4	33.712	
EIF2S1	20.75564	20.32904	20.80002	0.260057463			3	3	2	2.19E-10	52197000	13	14.9	36.112	
EIF2S2	21.1684	21.41287	21.13402	0.15204434			2	2	2	3.07E-09	56630000	11	10.8	38.388	
EIF2S3;EIF2S3L	20.91174	21.48773	20.83345	0.357299156			2	2	2	1.56E-16	1.11E+08	18	14	51.109	
EIF3A	22.13712	21.88379	21.60833	0.264472168			4	4	3	3.50E-24	1.03E+08	19	7.2	166.57	
EIF3B	19.64873	NaN	NaN	N/A			2	1	1	1.04E-07	27529000	6	4.5	92.48	
EIF3C;EIF3CL	22.14985	21.86431	22.10981	0.154599775			4	4	3	1.40E-25	1.44E+08	33	11.2	104.1	
EIF3E	22.62165	22.7868	22.50074	0.143599022			6	7	6	1.05E-25	2.32E+08	46	21.1	52.22	
EIF3F	22.28023	21.76288	22.20652	0.27985134			3	4	4	4.00E-26	1.46E+08	26	25.2	37.563	
EIF3H;EIF3S3	21.49091	21.41787	21.40052	0.047969115			2	2	2	7.43E-10	72084000	13	10.8	39.93	
EIF3L	21.72696	21.74296	21.47338	0.151235023			4	4	2	3.23E-18	1.11E+08	24	14.2	66.726	
EIF3M	NaN	21.79626	NaN	N/A			1	3	1	4.22E-15	1.26E+08	22	15.5	42.502	
EIF4A1	24.86406	24.85766	24.73273	0.074045069			17	14	16	4.71E-110	1.2E+09	132	53.2	46.153	
EIF4A3	20.58674	NaN	20.16005	0.301715392			2	1	3	1.00E-14	45677000	9	16.3	46.871	
EIF4G1	22.46538	22.50913	22.57801	0.056780328			6	6	6	1.14E-32	1.58E+08	31	11.1	154.8	
EIF4G2	NaN	20.10712	NaN	N/A			1	2	2	2.33E-08	29369000	7	4.7	98.149	
EIF5A	23.12594	23.19426	23.91397	0.436585499			4	4	4	1.03E-21	2.53E+08	32	54.5	16.832	
ENDOD1	NaN	20.6734	NaN	N/A	+		2	3	2	5.36E-10	40164000	7	11.8	55.016	
ENO1	26.12497	26.51703	26.15518	0.218158624			16	17	15	2.03E-225	3.38E+09	290	52.1	47.168	
ERGIC2	23.05398	22.93702	23.24987	0.15807575			6	8	6	3.60E-37	5.08E+08	71	28.4	42.548	
ERGIC3	22.90736	22.59703	22.3036	0.301919418			3	3	2	2.37E-42	3.93E+08	51	26.6	43.222	
ESD	20.67081	20.68509	21.41947	0.428176307			2	2	2	8.50E-13	96215000	14	25.9	31.462	
ETF1	NaN	19.87769	NaN	N/A			2	2	1	1.62E-07	39365000	6	10.4	45.462	
FABP5	19.11617	18.80035	NaN	0.223318464	+		2	2	1	8.64E-06	21856000	5	14.8	15.164	
FAM129A	22.74218	23.07369	22.40338	0.335161607	+		4	5	4	1.16E-44	3.02E+08	50	14.7	103.13	
FAM129B	26.094	26.17102	26.07486	0.0509005	+		21	18	21	1.08E-262	2.22E+09	223	42	84.137	
FAM49B	26.23641	26.28494	26.08471	0.104451028	+		17	14	15	0	4.62E+09	348	73.1	36.748	
FAM69B	22.38002	22.39503	22.0462	0.197206939			5	7	5	3.25E-38	2.56E+08	36	29	48.582	
FAM84B	22.44693	23.31465	22.24264	0.569192094	+		4	4	5	2.50E-34	2.83E+08	42	31.6	34.474	
FASN	21.17706	20.9693	21.07692	0.103902439			4	3	4	5.08E-18	54301000	13	4.3	273.42	
FBXO17;SARS2	21.90588	22.11712	21.66145	0.228036368	+		2	3	3	1.82E-23	1.96E+08	26	34.2	31.479	
FDPS	20.59623	21.10553	21.43217	0.421282934			2	3	3	3.96E-08	92928000	14	10.5	40.532	
FKBP4	23.61302	23.55812	23.16789	0.242704958			7	7	7	4.29E-36	3.95E+08	59	33.6	51.804	
FLNA	22.26588	21.88446	22.4868	0.304713072			5	6	7	1.97E-43	1.77E+08	25	7.9	276.55	
FLOT1	23.66644	24.022	23.31935	0.351333508	+		7	11	11	5.10E-92	6.56E+08	75	49.6	42.08	
FLOT2	25.29488	25.62394	25.15831	0.23935439	+		16	17	14	3.25E-136	2.15E+09	218	51.2	47.064	
FMNL2	20.61144	20.27708	20.38522	0.170619634	+		3	2	2	3.03E-13	43226000	7	5.7	123.32	

FMNL3	20.91802	21.13965	NaN	0.156716076	+		2	5	1	1.05E-25	91132000	21	9.8	117.21	
FSCN1	23.47556	23.32182	23.22712	0.125383752			10	11	11	2.27E-45	4.6E+08	88	25.6	52.262	
FUBP1	21.97698	22.02151	21.74324	0.149472128			5	5	5	7.03E-21	1.5E+08	20	17.2	67.56	
FYN	23.02935	23.38245	23.90067	0.438259839	+		7	6	6	1.22E-97	5.6E+08	63	44.5	60.761	
GALNT1	21.37045	21.01522	21.50242	0.251981571	+		4	5	2	4.33E-15	1.15E+08	17	12	57.379	
GALNT2	20.84713	20.76951	20.74509	0.05328127			3	3	3	7.30E-12	58308000	13	10.7	60.793	
GANAB	21.91214	22.16085	21.53929	0.312839315			5	4	2	9.90E-45	2.25E+08	45	18.9	96.215	
GAPDH	25.96691	25.9329	25.90093	0.032995256	+		11	11	13	6.55E-212	2.43E+09	195	63.6	36.053	
GDI2	23.04382	22.95316	23.32224	0.192335201			6	6	6	1.48E-35	3.09E+08	38	34.8	50.663	
GHITM	22.39207	22.41903	22.31802	0.052302429			2	3	3	5.38E-21	2.13E+08	25	16	35.282	
GLG1	22.22873	22.5653	22.21824	0.19741667			6	6	4	1.40E-34	1.87E+08	25	11.2	134.55	
GLIPR2	20.80955	20.07392	20.51884	0.370499124	+		2	2	2	3.20E-10	66801000	12	20.3	14.213	
GLO1	23.08648	23.34746	22.87057	0.238799693			5	5	4	9.91E-26	4.12E+08	45	50.3	19.043	
GLRX3	21.65583	21.63143	NaN	0.017253405			3	3	2	6.61E-07	49239000	6	9.3	37.432	
GMPS	20.74649	20.54331	20.43746	0.157048759			2	2	2	4.76E-06	46202000	11	3.7	65.928	
GNA11	24.18397	24.292	23.93912	0.180806664			8	9	7	5.04E-89	1.08E+09	90	50.7	42.123	
GNA13	22.87704	22.23395	22.67042	0.328319619			5	7	4	6.67E-45	3.51E+08	67	34.7	44.049	
GNAI1	24.1795	24.24271	24.40652	0.117166051	+	+	7	7	7	3.76E-210	7.19E+08	72	45.2	40.361	
GNAI2	25.76709	25.54966	25.27863	0.244719648	+		8	9	9	0	2.35E+09	176	62	40.45	
GNAI3	27.23087	26.68016	27.06116	0.282029546	+	+	15	15	16	2.06E-231	6.9E+09	372	61	40.532	
GNAQ	NaN	21.87931	NaN	N/A	+		0	2	0	3.81E-40	90141000	10	37.9	42.142	
GNB1	22.86147	22.41532	22.5935	0.224575843			3	4	3	6.57E-13	2.23E+08	25	12.9	37.377	
GNB2L1	23.46426	23.58681	23.72588	0.130896901			5	6	7	3.02E-46	5.82E+08	60	40.1	35.076	
GOLGA7	23.3573	23.3672	22.92635	0.251715658			3	5	4	7.22E-29	3.35E+08	66	40.1	15.824	
GOLIM4	24.50749	24.3571	23.78646	0.380379508	+		8	8	6	9.67E-193	1.01E+09	107	17.7	81.879	
GORASP1	22.20311	21.75165	21.53588	0.340482182	+		3	3	2	3.86E-11	1.68E+08	19	16.3	23.906	
GORASP2	25.90731	25.86769	25.6992	0.110505201	+		7	7	5	9.70E-171	2.91E+09	148	36.9	47.145	
GOT1	20.5093	20.41844	20.43675	0.048052572			2	3	2	3.10E-14	1.09E+08	14	15.7	46.247	
GOT2	23.046	22.60598	22.82854	0.220014926			4	5	3	1.98E-40	3.13E+08	46	29.5	47.517	
GPI	23.7678	24.01402	23.14029	0.450517702			6	7	5	1.84E-50	7.16E+08	79	24.9	63.146	
GPRC5A	22.95584	23.05016	23.03934	0.05161651			2	3	2	2.15E-82	5.38E+08	33	14.8	40.251	
GPRC5C	20.1006	20.46692	NaN	0.259027356	+		2	2	1	1.10E-08	54549000	8	10	48.193	
GRPEL1	20.83954	21.0254	NaN	0.131422866			2	2	1	2.01E-05	49739000	4	9.2	24.279	
GSPT1;GSPT2	21.60644	21.48508	21.50315	0.065477221			4	4	3	9.09E-15	1.21E+08	12	10.8	68.6	
GSTO1	23.64845	23.26069	23.52456	0.19804626			5	5	5	2.11E-18	3.31E+08	41	30.3	27.566	
GSTP1	25.65494	25.76258	25.75346	0.059687707			6	6	5	1.76E-166	2.4E+09	145	57.6	23.356	
H3F3B;H3F3A;HIST2	NaN	23.43632	NaN	N/A			1	2	0	3.33E-12	66703000	6	15.2	10.334	
HADHA	22.35614	22.39189	22.26722	0.064196882			7	7	7	9.30E-34	2.24E+08	39	19.3	82.999	
HADHB	23.9686	23.78566	23.69519	0.13928681			10	10	9	1.54E-60	8.77E+08	87	40.3	48.879	
HARS;HARS2	21.03066	20.99452	20.78891	0.130399792			3	4	3	6.83E-13	68602000	14	11.1	49.623	
HCCS	25.01992	25.4087	24.75527	0.328674174	+		10	9	8	5.27E-112	1.41E+09	172	60.1	30.601	
HIST1H1C;HIST1H1E	21.41251	22.03093	NaN	0.437288976			2	3	1	1.99E-08	61928000	6	15	21.364	
HIST1H2AJ;HIST1H2	NaN	22.66841	NaN	N/A			0	2	0	3.52E-07	83368000	6	27.3	13.936	
HIST1H2BL;HIST1H2	NaN	25.57759	NaN	N/A			1	3	1	4.08E-14	2.71E+08	10	35.7	13.952	
HIST1H4A	19.53463	25.19884	20.25557	3.083259847			2	4	3	1.63E-22	2.42E+08	21	50.5	11.367	
HLA-B;HLA-C	22.40715	22.39186	21.92984	0.271268963			4	5	4	2.78E-39	2.6E+08	51	26.8	40.46	
HLA-C	19.64755	19.87442	19.50576	0.185959044			2	2	2	8.95E-30	30057000	10	26.5	40.648	
HM13	23.3243	23.27142	23.1146	0.109058774			3	2	3	1.24E-32	3.84E+08	48	22.7	36.813	
HMGB1;HMGB1P1	22.71676	22.5932	22.15178	0.297018824	+		3	4	4	7.82E-22	1.73E+08	33	30.2	24.893	
HMGB2	NaN	20.85878	NaN	N/A	+		1	2	1	5.90E-15	36018000	11	21.5	24.033	

HMOX2	NaN	22.0232	21.60205	0.297798021			2	5	3	2.07E-19	97839000	13	34.8	32.837	
HNRNPA1;HNRNPA1	21.9903	21.9903	22.17075	0.104182856			3	2	2	6.42E-25	1.57E+08	24	38.6	29.386	
HNRNPA2B1	21.13965	21.02161	20.54472	0.314986565			2	2	3	1.64E-11	85576000	13	12.6	36.006	
HNRNPD	21.75197	22.16192	21.75026	0.237179918			3	4	3	4.76E-23	2.25E+08	25	46.8	12.553	
HNRNPF	22.05169	21.39921	21.97127	0.355773844			2	4	4	3.82E-16	1.2E+08	19	18.1	45.671	
HNRNPK	23.86098	23.53552	23.18616	0.337480531			7	11	9	6.15E-54	4.39E+08	62	37.4	47.557	
HNRNPM	24.32231	24.48904	24.53279	0.111066636			14	15	12	1.10E-68	8.39E+08	106	38.6	73.62	
HNRNPU	21.64935	21.66644	NaN	0.012084455	+		3	4	2	2.72E-23	1.62E+08	20	8.8	88.979	
HPCAL1	24.39087	24.43071	24.3245	0.053654398	+		9	8	9	1.42E-66	1.03E+09	133	59.6	22.313	
HPRT1	22.5598	22.27603	21.78421	0.392418181			4	6	3	8.38E-27	2.11E+08	36	42.7	24.579	
HRAS	NaN	20.80931	NaN	N/A			2	2	2	5.63E-21	84686000	7	31.7	21.298	
HSD17B10	21.78285	22.12654	22.1021	0.191764044			3	4	3	2.36E-16	1.51E+08	25	36.8	26.923	
HSD17B12	22.01454	22.12566	22.10121	0.058391132			3	4	4	8.83E-29	2.14E+08	30	34	34.324	
HSD17B4	20.88755	NaN	20.83268	0.038798949	+		2	2	3	1.58E-18	86934000	9	11	79.685	
HSP90AA1	26.16825	25.60071	25.85412	0.284310844	+		12	13	16	0	2.06E+09	195	45.4	84.659	
HSP90AB1	27.30287	27.32517	27.28648	0.019420085			23	25	25	0	5.69E+09	458	51.8	83.263	
HSP90B1	24.78995	24.82953	24.80592	0.019912514			13	13	13	2.90E-86	1.02E+09	112	33.6	92.468	
HSPA1A	28.32539	28.10335	28.04557	0.147726957			27	27	27	0	1.05E+10	729	61.2	70.051	
HSPA4	23.64592	23.42445	23.3362	0.159563729			10	10	6	7.24E-63	4.74E+08	72	29.6	94.33	
HSPA5	23.41957	23.35057	23.142	0.144515022			8	8	9	1.34E-82	5.09E+08	56	28.7	72.332	
HSPA8	26.16634	26.07739	25.8749	0.149360624			22	22	22	1.19E-192	2.5E+09	271	48.5	70.897	
HSPA9	24.315	24.26134	24.11712	0.102335476			10	10	8	1.52E-78	6.28E+08	86	26.8	73.68	
HSPB1	21.16632	NaN	NaN	N/A			2	0	0	2.27E-10	68430000	9	19.4	20.406	
HSPD1	26.40887	26.49196	26.02654	0.248225688			21	22	20	0	3.46E+09	307	55.7	61.054	
HSPH1	21.76365	22.06553	21.89685	0.151287098			5	6	4	1.87E-20	1.12E+08	17	12.8	92.115	
IDH3A	21.58192	21.60327	21.58229	0.012221019			3	2	3	3.39E-16	93357000	18	20.2	35.786	
IFITM2;IFITM3;IFITM	25.65998	25.44158	24.68715	0.510434683			3	3	3	3.84E-81	1.95E+09	79	29.5	14.632	
IGF2R	20.86197	NaN	NaN	N/A	+		2	1	2	8.09E-19	54818000	14	3.5	274.37	
ILF2	21.86045	21.86302	21.40919	0.261280137			3	3	3	6.80E-17	1.21E+08	19	21	43.062	
ILF3	21.52272	21.83298	22.20795	0.343123865			7	6	4	2.65E-22	1.37E+08	36	15.1	74.606	
IMMT	22.47892	22.34367	22.48033	0.078496822			4	7	4	1.41E-28	1.48E+08	24	13.9	78.973	
IMPDH2	21.98036	21.83453	22.12852	0.146996539			2	3	2	6.54E-18	1.48E+08	28	21.6	55.804	
IPO5	23.90279	23.22888	23.12196	0.423336364			8	11	7	5.23E-51	3.51E+08	46	21.6	123.63	
IPO7	23.11073	22.97615	23.11218	0.078121742			4	7	5	2.80E-41	2.86E+08	44	13.1	119.52	
IPO9	NaN	20.1061	20.8319	0.513218102			1	1	1	3.91E-10	36722000	5	3.6	115.96	
ISYNA1	NaN	20.56904	NaN	N/A			1	2	1	1.02E-08	42013000	8	8.8	44.786	
ITFG3	21.52931	21.6937	NaN	0.116241284			2	3	2	5.25E-20	1.15E+08	21	12.1	59.659	
ITM2B	22.0494	21.99549	22.02269	0.026955371			2	2	3	1.31E-46	1.93E+08	18	30.8	30.338	
JAM3	24.05457	24.11386	23.97885	0.067671415			3	6	4	1.64E-44	4.59E+08	47	27.1	35.02	
JUP	NaN	22.32638	NaN	N/A			0	5	0	1.55E-38	74558000	13	19.1	81.744	
KHSRP	23.76284	23.72119	23.7191	0.02467211			13	11	11	1.86E-50	6.38E+08	93	26.9	73.114	
KIAA0319L	22.0252	21.80203	21.634	0.196246601			3	3	3	1.09E-29	1.85E+08	20	7	109.75	
KIAA2013	22.23908	21.96155	22.38814	0.216495116			5	4	3	1.22E-30	2.55E+08	30	15.8	69.156	
KPNA2	21.79776	21.87852	21.80872	0.043807037			4	4	4	2.04E-17	1.32E+08	21	18.9	57.861	
KPNB1	25.70849	25.60538	25.44288	0.133907054			21	19	18	3.10E-111	1.66E+09	203	35.7	97.169	
KRT18	NaN	NaN	20.07874	N/A			1	0	2	7.65E-23	23995000	9	12	43.774	
LAMTOR1	26.70028	26.53295	26.45731	0.124335004	+		10	10	10	2.99E-230	3.45E+09	334	92.5	17.745	
LBR	20.99023	21.35122	20.93171	0.227202923			3	3	3	4.27E-08	1.08E+08	18	4.2	70.702	
LDHA	26.43314	26.27469	26.4508	0.096981966	+		10	11	13	7.36E-182	3.05E+09	215	61.1	36.688	
LDHB	27.3864	27.46638	27.35456	0.057611195			13	13	17	1.15E-180	6.48E+09	312	71.6	36.638	

LMAN2	20.69558	20.59414	20.60277	0.05624092			2	2	2	1.24E-08	52113000	8	8.4	40.228	
LMBRD1	23.15598	22.67031	23.21284	0.298174225			3	2	2	4.29E-43	3.9E+08	25	9.9	44.211	
LNP;KIAA1715	24.28968	24.33436	24.01759	0.171450918	+		8	8	8	1.25E-77	1.19E+09	137	40.4	47.094	
LNPEP	24.85695	24.78182	24.5856	0.140105691			14	17	13	3.65E-109	1.06E+09	132	27.7	117.35	
LOH12CR1	23.31451	23.28489	22.78951	0.294930412	+		6	8	4	7.39E-50	4.99E+08	48	64.4	20.247	
LRPPRC	24.64713	24.33531	24.29789	0.191746598			16	19	17	2.26E-75	6.2E+08	91	23.1	157.9	
LRRC1	NaN	NaN	22.07848	N/A			1	1	2	1.12E-54	1.25E+08	19	34.4	59.241	
LRRC15	NaN	22.91978	NaN	N/A			0	3	0	9.78E-07	61378000	4	4.1	64.365	
LRRC57	25.28602	25.26857	24.83928	0.253038546	+		10	10	11	8.54E-69	1.48E+09	128	59	26.754	
LRRC59	20.80152	21.12149	20.95851	0.159994344			3	3	3	2.25E-09	78528000	23	10.4	34.93	
LSR	24.41319	24.87404	24.10685	0.386179452			8	9	9	5.70E-101	1.06E+09	107	29.7	69.428	
LTA4H	20.84107	20.98794	20.81135	0.094549902			3	2	3	2.96E-18	85985000	12	18	69.284	
LUC7L2	22.18728	21.74579	22.86246	0.562395671			2	2	2	1.19E-07	99584000	17	7.9	46.513	
LUC7L3	22.48761	22.03691	22.35372	0.231453954			4	4	4	2.26E-13	99675000	14	14.6	42.571	
LYN	23.77384	23.86335	23.67939	0.091991054	+		9	12	10	2.96E-104	8.52E+08	94	43.9	58.573	
LYPLA1	20.96712	21.03765	21.2454	0.144668761			3	3	2	2.27E-11	1.05E+08	12	23.4	20.861	
LYPLA2	NaN	NaN	21.24208	N/A			1	1	2	5.01E-26	1.13E+08	18	22.5	24.737	
LZTS1	20.47605	NaN	NaN	N/A	+		2	1	1	5.99E-09	46441000	8	7.7	55.611	
LZTS2	NaN	19.56783	NaN	N/A			1	2	1	7.62E-08	19438000	3	5.8	72.759	
M6PR	22.80564	22.80725	22.84921	0.024703504			4	4	4	3.55E-16	3.16E+08	23	22.4	30.993	
MAN1B1	NaN	21.177	NaN	N/A			0	2	0	5.03E-21	1.34E+08	19	18.2	46.027	
MAPRE1	24.24227	24.32121	24.2527	0.042883425			7	7	6	8.68E-38	5.38E+08	64	46.3	29.999	
MARC2	23.16352	23.54828	23.06837	0.254102141	+		4	7	4	1.70E-45	5.6E+08	58	32.5	38.023	
MARCKS	29.69067	30.27175	29.85525	0.299503142	+	+	12	12	11	0	3.02E+10	411	48.5	31.554	
MARCKSL1	25.3305	25.64576	25.30946	0.188383138	+	+	6	6	6	3.32E-200	1.7E+09	76	60	19.529	
MBLAC2	24.59109	24.94168	24.02471	0.462697459			7	8	6	6.01E-35	1.13E+09	41	50.2	31.371	
MCCC1	27.40852	27.23663	27.67061	0.218546708			22	23	23	0	3.94E+09	306	52.8	80.472	
MCM3	20.41833	20.57616	20.56532	0.088160719			2	2	2	1.35E-09	51517000	11	4.3	90.98	
MDFIC	21.61234	21.79297	22.13918	0.267721543			4	4	4	4.55E-13	77699000	13	27.6	16.46	
MDH1	24.0831	23.90242	23.86533	0.116508014			8	9	7	1.96E-39	6.28E+08	73	32	36.426	
MDH2	24.36573	24.53178	24.58915	0.116031904			9	13	10	7.75E-70	9.66E+08	139	55.6	35.503	
ME2	20.39707	20.46683	20.09507	0.197600588			2	2	2	1.84E-13	55173000	10	8.7	65.443	
METTL7B	NaN	19.72699	NaN	N/A			1	2	0	1.21E-08	60108000	13	14.3	27.775	
MFSD5	21.3024	20.86778	NaN	0.307322749			2	2	1	2.87E-16	88261000	16	15.8	49.764	
MGRN1	22.41516	22.65355	22.08774	0.284070004	+		6	7	4	1.33E-44	3.91E+08	63	35.5	58.304	
MICB;MICA	20.4914	20.66284	NaN	0.121226387	+		2	2	1	0.000288	41059000	7	4.4	37.579	
MIF	24.26978	24.03246	23.80912	0.230365352			2	3	3	5.32E-19	5.92E+08	52	17.4	12.476	
MLEC	22.83268	22.65903	22.29499	0.274405412			4	5	3	2.11E-27	4.31E+08	46	53.4	16.729	
MRPS36	22.5093	22.90685	22.58715	0.210679344			3	2	2	4.24E-57	3.04E+08	38	56.3	11.466	
MSH2	19.44016	19.52726	NaN	0.061589001	+		2	2	1	2.12E-08	23074000	11	4.1	97.321	
MSN	23.0423	22.60474	22.93898	0.228710207			7	6	5	4.58E-42	2.94E+08	32	17	67.819	
MSRA	22.1466	22.1249	22.17146	0.023297865	+		2	2	2	6.79E-21	1.7E+08	22	36.6	23.627	
MTCH2	21.71121	21.82985	20.84337	0.538572884			2	2	3	5.69E-12	1.01E+08	20	18.5	33.331	
MTDH	22.47766	22.44443	22.26344	0.115290807			6	4	4	1.77E-46	3.02E+08	38	19.4	63.836	
MTHFD1	22.27216	22.51412	22.42514	0.122382571			3	4	4	2.19E-41	2.5E+08	49	17.3	101.56	
MT-ND4	20.68492	NaN	NaN	N/A			2	1	1	0.000293	32159000	7	5.4	51.58	
MYL12A;MYL12B;M	22.65342	22.58506	22.14793	0.274249238			3	3	3	6.59E-17	1.19E+08	23	24	19.794	
MYL6	21.6594	21.36778	20.99016	0.335539681	+		3	4	3	3.51E-13	1.11E+08	20	38.6	16.29	
NAA15	21.0551	20.84315	20.95624	0.106054585			3	3	3	8.65E-13	72237000	12	5.8	101.27	
NASP	23.95961	23.81646	23.59946	0.181332542			4	4	3	1.36E-25	3.56E+08	49	7.5	85.237	

NCAM1	25.18775	25.32673	25.33671	0.083270766			13	15	15	3.03E-90	1.39E+09	177	34.3	94.573	
NCL	21.59587	22.05956	21.82466	0.231851709			5	4	5	5.53E-18	1.41E+08	26	9.7	76.613	
NCR3LG1	NaN	21.75185	NaN	N/A			0	3	2	8.92E-19	1.1E+08	17	15	50.827	
NCS1	22.97348	23.23134	22.56797	0.334412394	+		8	8	7	4.15E-44	4.79E+08	83	62.6	21.878	
NCSTN	21.87639	21.33074	21.14942	0.37839491			3	3	2	1.28E-15	1.04E+08	16	10.4	50.26	
NDFIP2	21.26522	NaN	NaN	N/A			2	0	1	4.76E-09	53359000	12	18.9	26.019	
NDUFA4	22.13518	NaN	NaN	N/A			3	1	1	2.60E-09	1.34E+08	16	46.9	9.3697	
NDUFAF4	24.81982	24.24829	24.38416	0.29858188	+		7	8	9	6.10E-46	1.41E+09	104	53.1	20.266	
NDUFB7	24.70678	24.59548	24.08432	0.331945951	+		4	4	4	3.39E-56	9.65E+08	161	51.1	16.402	
NME2;NME1-NME2	23.87624	23.76577	23.69519	0.091254461			8	8	5	9.40E-34	7.37E+08	88	58.4	30.137	
NNT	21.23438	20.80821	20.77563	0.255973269			3	3	3	1.55E-14	73856000	13	5.8	113.89	
NPC1	23.98537	24.07765	23.63755	0.232085602			7	7	7	9.01E-40	6.55E+08	71	9.3	142.17	
NPEPPS	22.35135	22.9281	22.47398	0.303837581			5	6	5	2.06E-43	2.83E+08	56	20.1	102.99	
NPM1	23.66405	23.5134	24.369	0.45674597			4	5	3	1.66E-27	5.02E+08	54	26.8	29.464	
NRAS;KRAS	NaN	22.43303	22.33172	0.071636988			1	3	2	5.52E-21	2.44E+08	20	31.7	21.229	
NSUN2	NaN	20.42583	NaN	N/A	+		1	3	2	6.40E-09	41635000	8	5.6	82.392	
OAT	22.26439	22.42481	22.20559	0.113467635			6	5	4	9.80E-21	2.17E+08	37	17.8	48.534	
OGFRL1	22.6874	22.45184	22.09146	0.300140729	+		4	6	4	7.51E-42	2.54E+08	28	26.6	51.251	
OXCT1	NaN	20.1818	NaN	N/A			0	2	1	1.07E-09	24866000	6	7.9	56.157	
P4HB	22.47946	22.91592	22.86922	0.239649424			6	8	7	3.70E-32	2.61E+08	37	24.8	57.116	
PA2G4	24.20903	24.15182	24.19167	0.029332508			10	9	10	5.62E-45	6.59E+08	82	32	43.786	
PABPC1	21.78848	22.4383	21.95925	0.336877161	+		2	4	3	4.81E-22	1.81E+08	24	17.4	61.18	
PAICS	22.20508	22.45992	22.18961	0.151794951			7	6	6	2.21E-45	2.98E+08	50	36.5	47.079	
PALD1	21.50906	NaN	21.02898	0.339467824	+		3	1	2	1.09E-22	91871000	18	10.7	96.753	
PALM	22.8618	23.06276	22.59585	0.234207626			5	4	4	2.48E-13	2.27E+08	31	14.2	42.075	
PARK7	NaN	22.14821	NaN	N/A			1	2	1	7.71E-28	1.56E+08	18	45.5	19.891	
PARP1	22.41769	22.12641	22.50819	0.199495304			6	6	5	5.75E-18	1.29E+08	10	8.4	113.08	
PC	28.96419	28.7673	29.27304	0.254927095	+		46	47	47	0	9.94E+09	976	54.9	129.63	
PCBP1	23.41596	23.1876	23.13677	0.148704985			7	7	7	2.17E-35	5.24E+08	48	37.4	37.497	
PCBP2	23.849	24.17074	23.70788	0.237230852	+		4	4	4	1.49E-32	4.02E+08	40	31.9	38.15	
PCCA	28.19882	28.12642	28.1378	0.038933072			33	32	33	2.86E-259	5.69E+09	474	66.1	77.047	
PCCB	22.84337	22.43133	23.07524	0.326129			6	7	8	5.88E-21	1.18E+08	25	21.8	52.423	
PCMTD2	24.9349	24.90647	24.85875	0.038480051	+		12	12	12	1.14E-75	1.06E+09	148	41.6	41.071	
PCNA	22.7705	22.86268	22.74753	0.060943			3	3	2	2.97E-33	2.61E+08	27	47.1	28.768	
PDCD6IP	21.28574	21.31899	21.20234	0.060094849			2	5	3	2.39E-14	74616000	16	7.9	96.022	
PDHA1	19.91124	19.8396	19.94006	0.051728608			2	2	2	0.000108	24946000	6	5	40.188	
PDHB	20.42152	20.4491	20.58206	0.085841045			3	3	2	1.07E-11	72446000	13	15.5	37.514	
PDIA3	23.21055	22.65447	22.94306	0.278106711			8	7	7	6.59E-31	3.27E+08	34	26.2	54.963	
PEBP1	22.76241	23.08937	23.0257	0.173338943			3	4	5	5.73E-31	2.5E+08	25	56.1	21.057	
PFN1	24.48376	24.50379	24.20926	0.164569829	+		6	6	6	3.43E-39	8.26E+08	89	60	15.054	
PGAM1	24.95797	25.17001	25.14406	0.115660316			10	9	10	1.57E-138	8.34E+08	138	64.2	28.804	
PGD	21.47719	22.19769	NaN	0.509470436	+		4	4	3	5.96E-32	1.53E+08	23	31.9	51.872	
PGK1	26.19088	25.99914	26.0669	0.097233984			18	16	16	2.09E-152	2.27E+09	202	60	44.614	
PGLS	21.16846	21.62885	21.38516	0.230326818			3	5	3	3.99E-18	1.36E+08	20	45.7	27.547	
PGM1	20.23222	20.22459	NaN	0.005395225			3	3	2	9.58E-07	33366000	4	6.8	61.448	
PGRMC1	21.7043	21.71704	NaN	0.00900854			2	3	1	3.00E-10	1.07E+08	10	20	21.671	
PHB	22.43222	22.75073	22.48854	0.16998243			5	5	5	2.23E-16	1.64E+08	24	30.3	22.27	
PHGDH	23.63533	23.65611	23.7496	0.060868467			9	9	9	9.28E-66	5.38E+08	70	22.1	56.65	
PI4K2A	26.20687	26.32021	26.25483	0.056892679			24	25	24	0	3.57E+09	312	72.7	54.022	
PI4K2B	23.6936	23.54439	23.52409	0.092564711			12	10	12	9.25E-55	5.78E+08	72	38.5	54.744	

PIK3R4	22.72763	22.59339	23.0665	0.243819034	+		6	6	3	3.04E-44	3.01E+08	41	13.8	153.1	
PITPNB	20.255	20.10137	20.08835	0.092685777	+		3	3	2	1.92E-06	35896000	5	11.8	31.54	
PKM;PKM2	25.79739	25.90097	25.62165	0.141204949			16	17	17	4.64E-254	2.73E+09	244	55.4	57.936	
PKP1	NaN	22.48662	NaN	N/A			0	4	0	1.50E-42	32854000	7	6.2	80.496	
PLGRKT	23.02254	23.00067	22.84088	0.099172826	+		3	5	3	8.84E-31	6.36E+08	58	34	17.201	
PLIN3	22.4066	22.14482	22.26273	0.131104356			5	5	4	6.89E-20	1.37E+08	24	16.8	45.803	
PLP2	25.51923	25.36986	NaN	0.10562054			2	2	1	7.94E-58	2.08E+09	53	27	16.691	
PLS3	26.34792	26.05957	26.16942	0.145530635			21	23	19	5.61E-154	2.43E+09	264	47.9	70.81	
PLSCR1	26.00811	26.07012	26.23484	0.11717822			8	7	7	3.70E-91	1.96E+09	137	34.7	34.217	
PLSCR3	22.5455	23.11948	22.9068	0.290179115			3	4	3	6.59E-22	2.71E+08	36	26.1	31.648	
PMPCA	20.64421	20.25315	21.94013	0.883009824			3	2	2	1.09E-26	71790000	14	9.1	58.252	
PMPCB	20.66232	20.53212	NaN	0.092065303			3	3	1	2.59E-10	61659000	9	6.7	54.366	
PNP	20.96888	20.9754	NaN	0.004610336			2	3	1	2.36E-15	72023000	13	25.3	32.118	
PODXL	21.85905	22.01706	21.91254	0.080366629			3	3	2	1.04E-07	1.31E+08	10	5.5	55.385	
PPIA	24.68153	24.89176	24.48603	0.202909559			8	8	9	6.20E-36	1.04E+09	80	52.7	18.012	
PPM1A	25.23089	25.29502	24.9444	0.186692064	+		12	11	11	1.82E-170	2.21E+09	201	52.9	42.447	
PPM1B	24.26849	24.45218	24.32999	0.093501036	+		9	9	10	3.95E-127	1.2E+09	101	45.5	52.642	
PPM1G	27.40308	28.01223	28.03423	0.358212703	+	+	21	21	20	0	1E+10	538	63.9	59.271	
PPP2CA;PPP2CB	NaN	NaN	20.75792	N/A			1	0	2	8.05E-20	47984000	8	11	35.594	
PPP2R1A	24.44613	24.43638	24.5335	0.053480329			15	14	13	1.89E-64	7.77E+08	100	39.4	65.308	
PPP3CA	22.18068	22.01638	22.13182	0.084368421			5	4	2	1.52E-21	1.83E+08	26	17.2	57.658	
PPP3R1	25.4746	25.6376	25.62277	0.090132567	+		6	7	5	8.28E-69	1.21E+09	105	84.7	19.3	
PRAF2;WDR45	23.63001	23.32361	22.90159	0.365736138			3	2	3	1.65E-12	3.82E+08	38	26.4	19.258	
PRDX1	25.36215	25.2263	25.21456	0.08203237			7	7	7	1.50E-46	1.92E+09	93	53.3	22.11	
PRDX2	23.06566	22.57328	22.8872	0.24927622			3	3	3	2.54E-15	2.63E+08	43	19.2	21.892	
PRDX3	21.81143	21.90595	21.74526	0.080760732			3	3	3	2.50E-16	1.37E+08	20	31.5	25.838	
PRDX4	NaN	22.01628	NaN	N/A			0	2	1	3.40E-26	1.6E+08	25	35.4	30.54	
PRDX6	23.48333	23.6513	23.15495	0.252458138			7	6	7	1.10E-51	4.38E+08	73	50.9	25.035	
PRKAA1	22.02313	22.1278	21.49565	0.338822614			6	5	4	8.35E-25	2.47E+08	38	20	64.009	
PRKAB1	23.84278	24.00958	23.56558	0.224275902	+		5	7	5	1.57E-105	8.52E+08	74	51.5	30.382	
PRKAB2	NaN	21.42414	NaN	N/A	+		0	2	0	4.10E-17	1.05E+08	18	16.9	30.302	
PRKACA	26.50793	26.73286	26.71463	0.124933801	+	+	16	18	16	1.49E-121	4.92E+09	322	63.2	40.589	
PRKACB	23.31257	23.18064	23.10835	0.103551257	+		3	4	4	3.38E-84	4.63E+08	55	62.7	40.622	
PRKAG1	20.47368	20.6256	NaN	0.107423662	+		2	3	1	1.49E-12	70729000	9	20.2	28.285	
PRKDC	23.17126	23.26628	23.24721	0.050267398			11	12	9	3.00E-48	2.71E+08	56	5.8	469.08	
PRMT1	21.57191	21.27367	21.39451	0.150011201	+		3	3	3	5.30E-21	1.38E+08	19	25.5	37.709	
PRNP	21.67206	21.80869	21.78193	0.072405423			5	5	5	1.08E-22	1.97E+08	30	21.5	26.885	
PROCR	21.82683	21.787	21.54948	0.14995844			2	2	2	4.74E-21	1.9E+08	23	20.6	26.671	
PRPF19	20.97561	21.12111	21.0189	0.074711844			3	2	3	4.07E-13	88949000	9	8.7	55.18	
PRPF38B	21.99656	21.53241	22.71624	0.596493643			6	6	6	1.13E-13	95439000	16	10.3	64.467	
PSAT1	19.68464	19.77163	19.86737	0.091399909			2	2	2	4.57E-06	28048000	4	7.6	40.422	
PSMA1	20.99009	21.11165	20.65377	0.237185606			2	3	3	1.52E-13	1.21E+08	13	21.3	29.555	
PSMA4	NaN	21.91335	21.556	0.252684608			1	3	3	4.97E-12	1.39E+08	23	23.2	24.526	
PSMA6	21.34527	21.40707	NaN	0.043699199			2	2	1	4.80E-16	1.26E+08	22	27.7	16.645	
PSMA7	NaN	21.18271	NaN	N/A			1	2	1	5.66E-07	70511000	6	12.9	27.887	
PSMB2	22.02151	21.35111	21.79737	0.341277719			2	3	2	2.48E-07	1.12E+08	12	14.4	22.836	
PSMB5	22.10926	21.88293	22.10716	0.130069707			3	3	4	2.35E-23	1.34E+08	24	30.4	28.48	
PSMB6	21.48109	21.29961	21.33865	0.095523353			3	3	3	6.56E-10	1E+08	16	13	25.357	
PSMC1	28.09828	28.216	27.99253	0.111788418	+	+	20	22	18	0	1.32E+10	755	63	49.184	
PSMC2	24.93575	24.51171	24.39956	0.282809494			12	9	9	1.29E-82	1.22E+09	91	50.8	48.633	

PSMC3	NaN	23.64955	NaN	N/A			1	3	1	1.26E-20	2.6E+08	19	19.4	47.352	
PSMC5	24.65562	24.65464	24.43409	0.127618444			4	4	5	3.54E-25	1.28E+09	61	18.3	44.784	
PSMC6	21.59914	21.76583	21.61396	0.09225841			4	3	3	9.29E-26	1.35E+08	23	24.2	44.172	
PSMD1	22.19093	22.20762	22.0464	0.088656045			4	4	4	6.08E-17	1.38E+08	18	7.3	102.26	
PSMD11	20.90991	21.60598	21.49297	0.37355135			4	3	3	5.40E-15	95605000	20	14.2	47.463	
PSMD12	21.70075	21.74411	21.66432	0.039945126			3	5	4	3.20E-17	1.09E+08	28	14	52.904	
PSMD13	22.49722	22.28774	22.76332	0.238351077			5	5	4	3.41E-30	1.83E+08	35	24.7	42.945	
PSMD2	23.85681	24.22207	23.86193	0.20942059			11	6	8	5.34E-103	6.21E+08	92	31.1	100.2	
PSMD3	21.13308	21.05457	20.90514	0.115794206			2	2	2	1.43E-17	95938000	13	16	41.183	
PSMD6	21.08317	21.02736	21.02695	0.032340925			3	3	3	2.35E-14	79084000	11	20.3	45.531	
PSMD8	21.49541	21.40109	21.08861	0.212926534			3	3	2	1.70E-13	1.18E+08	23	29.7	19.781	
PTBP1	NaN	NaN	21.78991	N/A			1	1	2	3.30E-15	51454000	7	11.5	57.221	
PTGFRN	26.48115	26.59477	25.84967	0.401424651	+		24	23	19	2.36E-238	3.47E+09	299	43.5	98.555	
PTK7	23.78826	23.41725	23.62589	0.185985253	+		10	11	7	5.17E-82	6.62E+08	83	25	118.39	
PTPLAD1	23.93778	24.07447	23.69264	0.193464865			8	8	6	1.61E-45	6.68E+08	68	29.8	43.159	
PTRH2	NaN	21.19135	NaN	N/A			0	2	1	8.83E-13	71580000	9	29.1	19.193	
PTTG1IP	22.73502	22.67876	22.42603	0.164576457	+		2	2	2	2.29E-11	2.22E+08	34	22.8	20.324	
PVR	24.08164	24.40152	23.94235	0.235429398			4	4	4	3.01E-21	6.87E+08	43	13.5	39.304	
QKI	21.03322	20.82687	NaN	0.145911484			3	2	1	1.21E-07	75631000	12	10.1	35.232	
RAB14	20.62203	21.07946	20.38258	0.35407632			2	2	2	0.000455	30018000	4	7.9	23.897	
RAB1A;RAB1B;RAB1	23.43517	23.36426	22.12225	0.73839646			4	3	4	1.52E-14	2.11E+08	17	37	19.018	
RAD23B	22.77177	22.80835	22.36033	0.248777977			7	6	6	2.04E-15	1.52E+08	21	21	43.171	
RAN	24.06694	24.15383	23.73965	0.21840852			4	6	6	1.60E-29	7.34E+08	68	31.5	24.423	
RANBP1	22.3648	22.71194	22.61403	0.178982374	+		2	3	3	1.70E-12	2.34E+08	21	15.4	23.31	
RANGAP1	23.13348	23.03676	22.65659	0.252093917			3	6	3	2.84E-35	2.18E+08	44	21.3	63.541	
RAP1B;RAP1A	NaN	20.66865	NaN	N/A			1	2	1	1.28E-05	42316000	6	43.8	5.351	
RAP2B	24.7902	24.70941	25.00826	0.154590055			8	7	7	1.44E-47	1.13E+09	103	62.3	20.504	
RAP2C	21.73562	21.65036	22.63232	0.543995343			3	5	4	8.60E-42	1.87E+08	24	59	20.745	
RARS	20.62533	20.75271	20.88629	0.130492275			3	4	3	1.70E-11	49153000	9	8.2	75.378	
RBM39	21.61778	21.30524	NaN	0.220999153			2	2	2	2.95E-09	43638000	11	13.7	36.514	
RCC2	NaN	20.80294	20.72991	0.051640008			1	2	2	5.35E-09	47052000	8	6.5	56.084	
REEP5	23.71544	23.40574	23.2099	0.254898047			3	3	3	2.29E-30	3.68E+08	49	15.9	21.493	
RFTN1	20.86287	21.17548	20.74952	0.220610953	+		2	2	2	1.10E-18	1.28E+08	17	14	63.145	
RGS19	22.27338	22.52891	22.19217	0.175729182			3	3	3	1.36E-08	1.78E+08	16	16.1	24.635	
RHBDD2	20.91903	20.85483	NaN	0.045396255			1	1	1	2.05E-09	71679000	20	11.8	39.202	
RHOB	23.92823	23.84384	23.49363	0.230450962			3	3	2	5.78E-22	5.85E+08	52	36.2	22.123	
RNF141	24.46283	24.49899	24.1934	0.166975706	+		6	5	3	2.54E-57	8.38E+08	80	57.4	25.535	
RNH1	20.27458	20.32389	20.14196	0.09409044			2	3	2	1.43E-08	32885000	5	11.1	49.973	
RP2	24.93197	24.86547	24.73846	0.098319132	+		8	8	6	3.04E-38	1.43E+09	115	26.9	39.641	
RPL10	24.31334	24.47246	24.10933	0.182026853	+		5	5	5	7.64E-22	4.6E+08	24	35.5	22.975	
RPL10A	23.07791	22.55145	22.58628	0.294412782			5	3	3	2.39E-14	2.93E+08	30	26.7	24.831	
RPL11	NaN	22.85015	NaN	N/A			1	2	1	1.67E-78	2.37E+08	24	22	20.124	
RPL12	22.63547	22.46456	22.60781	0.091738638			5	3	2	3.58E-35	2.19E+08	29	54.5	17.818	
RPL13	29.25298	29.08699	29.20939	0.086056695			14	13	12	0	1.11E+10	425	46.9	24.261	
RPL13A	26.55855	26.10749	25.93575	0.321670015			7	8	8	3.89E-25	1.72E+09	144	30	23.577	
RPL14	22.44035	22.6504	22.42065	0.127340852			2	2	2	1.42E-12	1.56E+08	25	27.4	14.558	
RPL15	28.27644	28.34672	28.40202	0.062938733	+		16	15	16	7.79E-90	6.07E+09	363	52.5	24.146	
RPL18	29.78343	29.55525	30.00491	0.224838319	+		11	10	11	6.72E-251	1.45E+10	493	54.3	21.634	
RPL18A	23.75644	24.182	23.83123	0.227205652			4	4	4	1.25E-17	3.14E+08	33	27.7	16.714	
RPL19	25.78541	25.52677	25.69572	0.131328499	+		5	5	5	2.32E-38	1.15E+09	64	22.3	23.134	

RPL21	21.89785	22.08845	21.63041	0.230091705			2	2	2	3.16E-16	1.13E+08	17	20.6	18.565	
RPL22	23.05356	NaN	23.27867	0.159176808			2	2	2	8.96E-09	2.62E+08	17	51.1	5.0827	
RPL23	24.27434	23.97106	24.48536	0.258525527			5	5	5	9.67E-32	5.17E+08	48	43.6	14.865	
RPL24	24.1059	23.83904	23.38034	0.366982575			5	5	5	1.08E-14	3.21E+08	36	39.7	14.369	
RPL27	21.97243	22.00371	21.68003	0.178533349	+		2	3	2	3.10E-10	1.47E+08	28	20.6	15.798	
RPL27A	26.12904	26.2434	26.14334	0.062309313			6	6	6	2.39E-23	1.8E+09	126	35.8	16.561	
RPL28	28.12687	28.07158	28.40868	0.180790058			13	12	14	7.10E-92	4.38E+09	195	61.3	15.747	
RPL29	26.37627	26.50749	27.1776	0.429805357			4	4	4	1.11E-21	1.78E+09	45	23.3	17.752	
RPL3	24.44253	24.03507	24.47376	0.244761067			9	12	11	4.95E-48	6.26E+08	72	33	46.108	
RPL30	NaN	21.97589	21.39399	0.411465436			1	2	2	1.65E-14	1.08E+08	13	31.6	12.656	
RPL31	26.18422	25.92554	26.51473	0.29532385			4	4	4	4.45E-157	1.33E+09	79	32.8	14.463	
RPL32	30.38772	30.13443	30.62401	0.244839187			14	14	14	2.10E-291	2.9E+10	1376	60.2	15.616	
RPL34	28.41458	28.61618	28.8441	0.21489436			8	9	9	6.28E-37	6.21E+09	189	35.9	13.293	
RPL35	25.66373	25.34065	25.83461	0.250857577			5	5	5	5.74E-39	7.34E+08	61	29.3	14.551	
RPL36	27.93534	27.71921	27.97087	0.136202859			8	8	8	2.39E-70	5.55E+09	202	43.8	12.254	
RPL36A;RPL36AL	22.49531	22.40735	22.08216	0.217630582			3	3	2	1.62E-08	1.25E+08	26	32.1	12.441	
RPL4	26.31418	26.24561	26.38002	0.067209621			11	11	13	1.90E-66	1.96E+09	173	45.9	47.697	
RPL5	23.53267	23.094	23.66384	0.298427703	+		7	5	5	2.65E-37	4.71E+08	36	35	34.362	
RPL6	26.73828	26.18641	26.99399	0.412741557			11	12	11	2.23E-60	2.27E+09	215	42.7	32.728	
RPL7	23.94092	23.99412	24.1817	0.12648551			7	8	7	4.70E-28	5.22E+08	51	34.7	29.225	
RPL7A	23.68763	24.05192	24.0017	0.197428982			4	6	6	4.33E-24	4.72E+08	43	36.8	29.995	
RPL8	23.56046	23.7123	23.81206	0.126695174	+		3	3	4	2.66E-24	3.7E+08	47	20.2	28.024	
RPL9	22.16223	22.0451	21.77704	0.197461784			3	3	2	7.12E-18	1.7E+08	26	33.3	21.863	
RPLP0;RPLP0P6	24.79838	24.64075	24.75522	0.081458971			8	8	7	5.74E-73	9.1E+08	121	42.3	34.273	
RPRD1B	NaN	19.78788	NaN	N/A			1	3	0	8.37E-09	38774000	6	11.7	36.899	
RPS11	22.84044	22.5127	23.00299	0.249739963			5	5	4	3.49E-19	2.43E+08	32	51.3	18.431	
RPS12	22.17919	22.26979	22.06026	0.105083717			3	3	3	2.18E-18	1.77E+08	28	31.8	14.515	
RPS14	23.02687	22.89639	22.90062	0.07414174			3	3	3	3.13E-22	2.63E+08	34	31.8	16.273	
RPS15A	22.96443	23.50591	22.79513	0.37127512			3	3	3	2.46E-16	3.77E+08	32	54	11.477	
RPS16	22.38358	22.73403	22.63697	0.180943057			4	5	4	1.02E-20	2.76E+08	41	42.6	14.419	
RPS17L;RPS17	20.40925	19.95376	21.24412	0.654409147	+		2	4	2	1.09E-09	48831000	10	48.9	15.55	
RPS2	24.98494	24.64031	25.11339	0.244633715			9	9	8	5.94E-44	8.74E+08	96	42.7	31.324	
RPS23	23.72057	23.6608	23.321	0.215519789	+		4	3	4	3.95E-15	2.64E+08	51	28.7	15.807	
RPS25	23.34286	23.82736	23.39911	0.264985023			3	3	3	1.89E-10	3.92E+08	48	28	13.742	
RPS26;RPS26P11	23.57612	23.1333	24.06973	0.468444506			2	2	2	8.42E-07	2.47E+08	17	20.9	13.015	
RPS27L	NaN	23.02276	22.98357	0.027711515			1	2	2	1.47E-16	2.12E+08	17	38.1	9.4771	
RPS3	24.96614	25.08712	24.66828	0.215554976			12	12	12	2.23E-58	1.29E+09	114	70.8	26.688	
RPS3A	24.83292	24.77158	24.85296	0.042400671			11	10	10	5.59E-45	1.15E+09	95	52.7	29.945	
RPS4X	24.56442	23.94449	24.46538	0.333028657			9	12	11	3.27E-46	6.52E+08	84	47.1	29.597	
RPS5	24.69078	24.4095	24.90421	0.248129263			9	10	9	2.45E-60	7.69E+08	99	54	22.391	
RPS6	25.0712	24.75103	24.37577	0.348078483			4	4	4	5.45E-46	6.43E+08	66	18.9	28.68	
RPS8	24.57738	24.41596	24.36092	0.112502407	+		6	7	7	3.91E-72	5.6E+08	85	43.3	24.205	
RPS9	28.0921	28.05481	27.94754	0.075049973			15	15	14	2.54E-61	5.61E+09	278	50.5	22.591	
RPSA;RPSAP58	22.56209	22.34072	21.74139	0.424607587			2	3	2	1.00E-33	1.99E+08	22	28.1	29.404	
RRAS2	24.30952	24.64323	24.10109	0.273471878			7	8	6	3.27E-37	8.28E+08	62	47.5	23.399	
RRM1	22.03708	21.74698	21.95308	0.149271129			6	5	5	1.16E-22	1.53E+08	25	13.5	79.219	
RTN4	22.8823	22.82161	22.55272	0.175408136			4	4	3	2.39E-18	3.79E+08	33	20	37.144	
RUVBL1	21.38295	NaN	NaN	N/A			2	2	1	3.14E-17	79579000	14	18.6	50.227	
RUVBL2	22.36338	21.45954	22.15187	0.472755055			5	4	4	2.07E-20	1.46E+08	19	22.2	51.156	
S100A11	21.85049	21.67848	21.65193	0.107794879			2	2	2	3.09E-09	1.01E+08	12	37.1	11.74	

SAE1	NaN	20.1801	NaN	N/A			1	2	1	2.38E-10	44714000	8	19.6	29.422	
SAMM50	22.64456	22.80709	22.67168	0.087070243	+		7	8	5	9.07E-36	4.25E+08	40	32.2	51.976	
SCAMP1	24.21193	23.99602	23.39571	0.422927163			3	3	4	4.82E-64	5.7E+08	61	27.2	37.873	
SCAMP2	24.84995	24.78247	23.97631	0.486088871			2	3	2	1.88E-99	1.02E+09	65	13.7	36.648	
SCAMP3	26.31972	26.34498	25.71602	0.356062363			8	7	7	3.89E-222	3.12E+09	222	38.9	38.287	
SCARB1	23.13237	23.01415	22.37981	0.404703545	+		4	5	4	1.53E-27	2.76E+08	34	19.8	53.579	
SCARB2	26.14894	26.02663	26.04623	0.06569277	+		9	9	9	1.66E-132	2.9E+09	170	37.4	54.29	
SCARF2	20.98252	21.25742	NaN	0.194383654			2	2	1	2.19E-13	87420000	12	5.7	91.815	
SCRIB	22.17408	22.31973	22.09313	0.114829138			3	7	3	9.44E-47	2.73E+08	50	10.9	174.88	
SCYL3	19.74347	19.92809	NaN	0.130546054	+		3	2	1	2.18E-19	95085000	13	14.5	65.298	
SEC22B	20.22553	NaN	NaN	N/A			2	1	0	1.29E-07	30106000	6	14.9	24.593	
SEC61A1;SEC61A2	22.8928	22.90163	23.36986	0.272917431	+		4	5	4	1.48E-18	3.22E+08	35	12.4	52.264	
SEPT11	20.17889	NaN	NaN	N/A			2	1	1	4.85E-07	38501000	5	9.4	49.005	
SERBP1	20.84973	21.56881	22.0479	0.603077472			2	2	2	3.69E-17	57828000	12	12.9	42.426	
SERINC1	24.69631	24.6392	24.32965	0.197282501	+		2	3	3	1.04E-99	1.03E+09	61	10.4	50.494	
SERINC3	21.64015	21.04527	NaN	0.420643682	+		2	2	1	2.88E-12	1.43E+08	20	10	46.821	
SERPINB6	22.89665	22.42882	21.88774	0.504897987			5	4	3	5.20E-31	2.78E+08	38	39.4	42.621	
SFN	NaN	24.7543	NaN	N/A			1	2	1	2.70E-19	7.2E+08	30	20.8	24.336	
SFT2D3	23.72463	23.81705	23.27056	0.292509926			3	3	3	6.38E-56	5.28E+08	46	27	21.789	
SFXN1	22.52528	22.3165	22.226	0.153486078			5	5	5	3.46E-56	2.4E+08	40	35.1	35.619	
SGTA	20.86265	21.58573	NaN	0.511294771			5	4	3	3.08E-10	51623000	9	17.3	34.063	
SHISA2	21.65905	21.32861	20.78572	0.44095076			2	2	3	4.51E-12	96252000	9	15.6	31.375	
SHMT2	23.56906	23.66308	23.55086	0.060227802	+		10	12	13	4.41E-48	5.47E+08	84	35.6	53.454	
SLC17A5	21.14351	21.67077	21.01427	0.347778266			2	4	4	1.10E-13	1.58E+08	26	9.3	54.639	
SLC19A1	20.49023	20.60277	NaN	0.079577797			2	2	1	1.35E-05	54581000	9	3.9	64.868	
SLC1A4	21.27048	21.72476	NaN	0.321224469			3	2	1	4.31E-23	1.36E+08	16	17.7	55.722	
SLC1A5	25.77009	25.70572	25.61223	0.079376378			14	13	14	1.12E-218	2.31E+09	223	35.3	56.598	
SLC25A22	19.53856	NaN	NaN	N/A			2	1	1	0.001487	17154000	2	9.6	34.47	
SLC25A3	23.76719	23.31451	23.50397	0.227339336			3	3	3	1.31E-19	5.6E+08	45	19.7	39.958	
SLC25A5	26.24251	26.3036	26.21188	0.046695366			13	9	11	8.87E-68	3.07E+09	207	42.3	32.852	
SLC25A6	22.7253	22.54508	22.76497	0.117192555			2	3	2	6.07E-53	2.81E+08	32	41.9	32.866	
SLC29A1	20.67702	NaN	NaN	N/A			3	2	1	4.38E-15	44667000	7	9.4	50.219	
SLC30A1	22.49133	23.13336	22.57362	0.349352504	+		3	4	3	2.42E-49	3.63E+08	36	19.7	55.299	
SLC35B2	25.32701	25.47411	25.27615	0.102805084			12	12	10	1.41E-51	1.81E+09	159	33.7	42.127	
SLC35F6;C2orf18	20.89178	NaN	NaN	N/A	+		2	1	1	1.31E-17	67738000	7	5.7	40.214	
SLC38A1	23.15153	23.21801	22.7966	0.226561722			6	4	3	2.42E-37	4.13E+08	34	10.7	54.047	
SLC38A2	24.39185	24.20015	24.20643	0.108910441			3	4	4	3.92E-116	8.95E+08	80	17.6	56.025	
SLC39A10	20.67745	20.71188	NaN	0.024345686			2	2	1	1.34E-08	65422000	11	4.5	94.131	
SLC3A2	21.53702	21.65101	21.55079	0.062219211			2	4	4	8.84E-24	1.36E+08	25	15.7	57.944	
SLC41A3	20.82966	21.44733	20.72542	0.390200003			2	2	2	3.59E-19	1.03E+08	17	30.3	16.43	
SLC44A1	26.10677	26.09307	25.60215	0.287469268	+		14	11	11	5.52E-105	2.31E+09	198	28.3	73.301	
SLC44A2	22.47358	22.6034	22.10053	0.261054838	+		2	2	2	1.85E-18	2.18E+08	28	8.5	79.845	
SLC5A6	22.11899	22.16036	21.28901	0.491567069			2	2	2	7.31E-15	1.66E+08	21	8.5	68.641	
SLC7A1	23.74622	23.05466	23.95509	0.471284786	+		4	4	4	3.35E-89	4.67E+08	33	14.8	67.638	
SLC7A2	22.54135	22.46628	22.46029	0.045170249			5	6	5	8.12E-34	2.85E+08	22	16.1	71.672	
SLC7A5	21.80751	21.68102	21.51941	0.144406326			3	3	2	4.19E-17	1.49E+08	19	11.2	55.01	
SLC9A6	20.79471	20.79535	21.38785	0.342264936			4	4	3	4.50E-22	1.04E+08	16	12.3	72.259	
SMS	21.91605	22.1452	NaN	0.162033519			4	3	1	2.30E-10	1.38E+08	20	14.7	35.278	
SNAP23	25.89034	25.9478	25.55418	0.21261934			10	10	9	3.56E-114	1.99E+09	175	76.3	23.354	
SNRNP70	23.21132	23.00043	23.74622	0.384446643			8	6	7	3.76E-20	2.42E+08	22	29.7	51.556	

SORT1	20.65779	20.52554	NaN	0.093514872			2	2	1	7.07E-15	51099000	15	8.1	92.067	
SPECC1	21.15128	21.60562	20.86559	0.373204147	+		2	3	2	7.44E-09	68971000	8	5.5	79.014	
SPRYD7	22.83809	22.83571	22.67437	0.093844285			3	3	4	2.07E-33	2.94E+08	38	45.9	21.666	
SRC	25.69602	26.11914	25.16081	0.480256297	+		16	18	15	2.08E-186	3.11E+09	243	51.5	59.834	
SRI	20.8336	20.69217	NaN	0.100006112			2	2	1	1.09E-06	49049000	8	19.4	17.605	
SRP68	21.15283	20.82563	21.02249	0.164723109	+		3	3	2	1.48E-10	70576000	9	8.2	60.284	
SRRM2	30.86938	30.67051	31.12355	0.227081816			83	85	87	0	3.55E+10	2305	39.3	299.61	
SRSF11	21.69515	21.83329	22.36113	0.351479512			2	2	2	2.51E-08	95271000	13	10	42.316	
SRSF6;SRSF5;SRSF4	21.31269	21.32323	21.28771	0.018242635			2	2	2	1.98E-05	42883000	5	5.4	38.418	
SRSF7	20.31052	NaN	20.59341	0.200033437			2	2	2	0.000164	15109000	4	15.9	15.257	
ST13;ST13P5;ST13P	22.87867	23.38706	22.31995	0.53375278	+		3	4	3	6.50E-32	3.07E+08	44	19.5	41.331	
STARD3NL	21.19418	21.68007	NaN	0.343576114			2	2	1	3.26E-07	80567000	13	11.2	23.505	
STBD1	20.96289	20.40125	NaN	0.397139453	+		2	2	1	9.06E-14	70906000	12	16.8	39.007	
STIP1	23.9805	24.29817	24.00736	0.17616571			8	8	9	2.83E-57	6.44E+08	74	30.9	62.639	
STOM	23.88036	24.15244	24.01368	0.136049064			7	6	6	3.73E-83	7.8E+08	70	57.3	31.73	
STRAP	20.88985	21.194	20.90507	0.171376494			2	3	2	5.78E-13	78375000	12	16.3	38.438	
STT3A	22.45	22.37851	22.63305	0.131281335			2	3	2	2.44E-24	2.45E+08	32	10.4	80.529	
STX10	NaN	NaN	20.11057	N/A			1	1	2	4.33E-13	45904000	9	70.7	6.3891	
STX12	24.01818	23.97588	23.72213	0.160116601			5	5	5	1.30E-55	6.07E+08	74	35.5	31.642	
STX6	NaN	NaN	20.4342	N/A			1	1	2	1.11E-22	68112000	12	25.1	29.176	
STX7	24.86627	24.97723	24.45325	0.276119745			8	8	7	5.92E-173	1.06E+09	101	46.9	27.4	
STX8	22.37348	22.68983	22.376	0.18192166			3	3	4	6.42E-28	3E+08	43	45.3	26.906	
SURF4	23.50519	23.15173	23.13408	0.209351429	+		5	6	6	4.72E-28	4.23E+08	97	39.8	21.127	
SVIP	24.09941	23.9331	23.88837	0.11120377	+		2	2	2	8.99E-31	6.4E+08	63	32.5	8.4426	
SYNGR2	22.62983	22.53203	21.5898	0.574314753			2	2	2	1.06E-10	2.24E+08	27	16.1	24.81	
TAGLN2	25.64293	25.57542	25.1694	0.256138147			9	8	7	3.16E-65	1.47E+09	145	80.4	22.391	
TALDO1	23.796	23.89587	23.47494	0.219937721			9	9	9	1.04E-38	6.09E+08	81	35	37.54	
TARS	22.50809	22.63218	22.35652	0.138058097			4	5	5	1.24E-26	2.12E+08	45	14.7	83.434	
TCP1	23.98572	23.93283	23.84163	0.072888865			13	12	12	1.25E-68	6.05E+08	99	50.2	60.343	
TECR	21.46503	21.8077	21.89667	0.227907686	+		4	3	3	4.07E-10	1.03E+08	10	11.7	36.034	
TFRC	27.27435	27.14822	26.81897	0.235118865			27	27	23	1.90E-297	5.38E+09	446	51.3	84.87	
TKT	23.84518	23.50724	23.36626	0.246117577			8	8	8	3.65E-33	5.03E+08	50	26.2	62.878	
TLDC1	23.7683	24.19077	23.8375	0.226594069	+		6	6	6	5.97E-70	7.67E+08	92	36.4	50.993	
TLN1	21.59104	21.45322	NaN	0.097453457			3	4	2	2.00E-13	76736000	11	3.7	258.08	
TM9SF3	22.3473	22.38943	21.32025	0.60549601			3	3	2	1.12E-13	1.66E+08	27	8.3	67.887	
TMEM106B	24.93107	24.70893	24.67239	0.139998032	+		6	7	5	5.78E-78	1.07E+09	78	36.9	31.127	
TMEM106C	21.86985	21.77559	21.63865	0.116254714	+		1	1	1	1.97E-19	1.78E+08	20	14.4	27.875	
TMEM168	NaN	21.86676	NaN	N/A			2	3	2	2.84E-33	2.24E+08	29	15.5	79.754	
TMEM179B	23.8532	NaN	NaN	N/A			2	1	1	1.01E-15	2.7E+08	32	15.5	23.55	
TMEM181	21.47689	21.47269	21.29569	0.103424755	+		3	3	3	8.67E-13	1.33E+08	13	6.5	69.324	
TMEM184C	22.12512	NaN	NaN	N/A			2	1	1	7.65E-76	2.24E+08	13	9.6	50.141	
TMEM33	22.63157	22.15317	22.36744	0.239632653			3	4	3	1.04E-23	2.05E+08	41	23.5	27.978	
TMEM50A	22.48581	22.19292	22.23479	0.158402804			3	3	3	5.78E-18	2.8E+08	32	29.3	17.4	
TMEM50B	21.83522	21.8916	21.56686	0.173518424			2	2	2	1.31E-08	93251000	14	15.2	16.854	
TMEM55B	22.53312	22.88655	22.20249	0.342093322	+		3	4	3	2.44E-23	3.01E+08	36	26	29.469	
TMEM59	20.96388	20.93711	20.58985	0.208648261			3	3	2	5.39E-11	58491000	3	19.8	21.86	
TMEM63A	NaN	20.90903	NaN	N/A			1	2	0	4.58E-14	1.03E+08	16	6.3	92.125	
TMEM63B	23.18905	23.26084	23.00405	0.132488936			9	7	6	9.97E-31	3.78E+08	50	11.7	94.957	
TMEM97	22.36228	22.31603	22.20066	0.083236935	+		2	2	2	0.000116	1.76E+08	14	10.2	20.848	
TMPO	19.66628	19.82665	19.65314	0.096606519			2	2	2	0.000105	30332000	6	8.9	26.866	

TMX1	24.85201	24.56092	24.82803	0.161583921			8	8	8	2.61E-59	1.35E+09	138	32.9	31.791	
TMX3	22.01458	21.7523	21.58779	0.215253356			2	2	2	2.42E-18	1.65E+08	30	18.1	51.871	
TMX4	21.28269	21.42168	21.22441	0.101349421			2	2	2	3.34E-12	1.01E+08	17	13.5	38.952	
TNFRSF10A	23.78327	24.08942	23.61863	0.238913295			5	4	5	6.36E-32	5.39E+08	62	19.2	50.089	
TNPO1	22.34055	22.21807	22.73944	0.272623446			3	5	2	1.34E-30	1.89E+08	41	15.6	101.31	
TOMM40	23.8798	23.54486	23.12179	0.37985791	+		3	4	3	2.17E-68	3.4E+08	26	16.6	37.893	
TPD52L2	21.90544	21.84698	21.47966	0.230806615			3	4	3	2.31E-15	92947000	19	25.2	22.237	
TPI1	27.56507	27.54672	27.47437	0.047954379			14	15	13	0	5.69E+09	383	85.9	26.669	
TPT1	22.36565	23.14397	NaN	0.55035535	+		3	3	2	3.17E-17	1.15E+08	14	25.6	19.595	
TRAP1	23.02808	23.31036	22.91621	0.203121933			7	8	6	2.70E-72	4.74E+08	68	24.9	74.267	
TRAPPC3	24.51497	24.63683	24.14538	0.255919821	+		5	6	5	2.21E-32	9.66E+08	75	41	15.005	
TRIM28	22.59789	22.11721	22.02901	0.306174503			7	9	3	3.54E-41	1.88E+08	37	12.6	88.549	
TSPAN13	22.70712	22.96543	22.83472	0.12915812			5	5	4	8.27E-20	2.46E+08	28	26	22.147	
TSPAN14	22.42793	NaN	NaN	N/A			2	1	1	1.18E-11	1.52E+08	20	14.6	28.876	
TSPAN3	27.03999	26.82189	27.2076	0.193404984	+		5	4	4	4.69E-52	4.32E+09	77	38.6	25.183	
TSPAN6	25.4551	25.2738	24.95074	0.255478787			6	7	6	2.38E-64	1.77E+09	128	37.1	27.563	
TSPAN7	21.54844	21.43476	NaN	0.080383899	+		2	2	1	1.34E-17	93774000	11	11.2	24.475	
TSPAN9	NaN	20.6172	20.93862	0.227278262			1	2	2	5.39E-13	93473000	21	20.1	26.779	
TTYH3	23.72962	24.06866	23.4419	0.313729984			4	5	6	2.75E-24	4.84E+08	53	17.1	54.139	
TUBA1B;TUBA4A	27.79576	27.73234	27.83635	0.052420932	+		17	17	16	0	9.77E+09	672	59.9	50.151	
TUBA1C;TUBA1B	22.80322	NaN	NaN	N/A	+		2	1	1	0	1.88E+08	28	60.1	49.895	
TUBB	25.34282	25.45673	25.88423	0.285440165			5	5	5	0	1.74E+09	133	58.2	47.766	
TUBB4B;TUBB4A	27.5618	27.34181	27.36302	0.121352759			19	20	20	0	7.07E+09	705	59.6	49.83	
TUFM	22.14722	22.11191	21.99935	0.077224874			5	5	5	2.31E-26	1.91E+08	34	18.6	49.541	
TXNDC5	NaN	20.66709	20.68252	0.010910658			1	2	2	1.91E-12	51511000	12	16	36.177	
TYMS	NaN	19.28229	NaN	N/A			0	2	1	7.72E-05	20331000	5	7.2	31.758	
UBA1	25.60809	25.40305	25.5433	0.104808721	+		19	21	18	2.88E-151	1.91E+09	205	37.9	117.85	
UBB;RPS27A;UBC;U	25.92764	25.72151	25.40662	0.262395099			4	4	4	4.96E-69	1.89E+09	149	50.5	10.469	
UBE2M	22.04564	22.16971	22.09	0.062868724			4	3	3	3.89E-13	1.29E+08	17	35.5	20.9	
UBE2V1;TMEM189;	20.56458	NaN	NaN	N/A			2	1	1	2.16E-10	73551000	16	24.5	16.495	
UBTD2	NaN	21.8913	NaN	N/A	+		1	2	1	7.70E-28	1.23E+08	29	24.4	26.189	
UCHL1	24.50755	24.61549	24.29207	0.164662873			8	7	8	2.42E-73	1.13E+09	117	59.6	24.824	
UQCRC1	21.59828	21.45779	21.4208	0.093634796			3	3	3	1.12E-13	85573000	11	15.4	52.645	
UQCRQ	24.77821	24.75389	25.14351	0.218265624	+		6	5	6	2.33E-24	5.59E+08	102	74.4	9.9062	
USMG5	NaN	21.32312	20.89208	0.304791307			1	2	2	1.37E-06	76824000	6	43.1	6.4575	
USP12;USP46	20.61827	20.34375	NaN	0.194114954			2	2	1	1.09E-07	56852000	8	10.5	42.857	
USP7	22.36044	22.32762	22.3689	0.021805054			3	4	4	1.18E-32	3.38E+08	40	13.5	117	
VAMP3;VAMP2	24.28023	24.56715	23.53505	0.532736134			2	2	2	1.86E-157	1.28E+09	43	40	11.309	
VAMP7	24.41803	24.12959	23.53112	0.452396088			8	7	7	1.13E-47	9.21E+08	82	45.5	24.935	
VANGL1	21.4051	NaN	NaN	N/A			3	1	1	6.72E-13	69084000	7	12.3	59.747	
VCL	24.4179	24.66666	24.08902	0.289744589			18	23	19	2.16E-84	7.96E+08	139	29.1	116.72	
VCP	22.72447	22.61045	22.62934	0.061110705			6	5	7	4.53E-46	2.43E+08	50	16.1	89.321	
VDAC1	22.48974	22.05874	21.99669	0.268548414			3	2	2	4.21E-21	1.92E+08	37	23	30.772	
VDAC2	26.38103	26.3181	26.06743	0.165901876			11	11	9	1.60E-124	2.61E+09	161	61.6	31.566	
VDAC3	22.44445	22.76294	22.37925	0.205306655			4	3	3	8.87E-26	2.97E+08	32	22.3	30.658	
XPO1	23.90215	23.97588	23.77686	0.100616978			11	13	10	2.45E-56	7.89E+08	72	22.7	123.38	
XPO5	20.33276	20.00662	20.74813	0.371648718			3	2	2	1.23E-08	34216000	8	2.7	136.31	
XRCC5	22.77866	22.76711	23.33783	0.326222268			7	6	4	8.52E-59	5.16E+08	64	25.3	82.704	
XRCC6	23.9136	24.05838	23.53017	0.27294249			5	8	9	1.79E-51	5.25E+08	71	29.9	69.842	
XXYLT1	NaN	19.84681	NaN	N/A	+		1	2	1	2.54E-06	33447000	7	13.9	21.816	

YARS	21.74981	21.60883	21.54816	0.103456055	+		5	5	6	1.69E-18	1.02E+08	19	14.4	59.143	
YES1	22.71597	22.46064	23.63279	0.616399214	+		5	5	3	6.69E-79	4.32E+08	52	43.3	60.801	
YWHAB	24.74381	24.82343	25.01039	0.136844366			6	6	7	1.99E-123	1.15E+09	78	48.4	27.85	
YWHAE	26.80403	26.68953	26.71816	0.059586889			13	13	13	4.56E-90	3.48E+09	215	61.2	29.174	
YWHAG	21.17505	21.7331	22.43934	0.633590818			3	2	2	1.59E-39	1.1E+08	25	35.2	28.302	
YWHAH	23.04984	22.41586	22.83982	0.322950234	+		4	3	4	4.43E-29	2.08E+08	27	26.8	28.218	
YWHAQ	23.85795	24.17204	24.1931	0.187715021			7	6	7	1.08E-99	5.14E+08	81	45.7	27.764	
YWHAZ	26.07765	26.31993	26.54463	0.233545145			8	10	8	3.32E-163	2.28E+09	171	53.1	27.745	
ZDHHC13	22.93789	22.85664	22.36997	0.307132539			7	7	6	6.79E-36	4.11E+08	46	14.8	70.86	
ZDHHC17	20.86673	NaN	NaN	N/A	+		3	1	1	5.99E-11	44400000	6	5.7	72.639	
ZDHHC18	21.93876	22.39679	21.78856	0.316832041			3	3	3	1.32E-22	1.56E+08	24	20.6	42.03	
ZDHHC20	NaN	20.90896	NaN	N/A			2	3	1	4.93E-15	82370000	14	17.3	42.277	
ZDHHC3	21.27515	21.03423	21.02783	0.14097907			2	2	2	3.38E-14	1.21E+08	10	9	34.17	
ZDHHC4	NaN	NaN	20.93452	N/A			1	1	2	6.89E-07	80415000	8	11.6	39.786	
ZDHHC5	NaN	21.50988	NaN	N/A			1	2	1	1.03E-23	1.58E+08	29	14.7	71.951	
ZDHHC6	21.88013	22.18782	21.43451	0.378753719	+		4	3	3	1.94E-30	2.79E+08	35	18.8	47.205	
ZMPSTE24	21.49209	21.28105	21.54062	0.138003398	+		3	3	2	2.57E-07	82939000	18	5.5	54.812	
ZNRF2	22.36161	22.68631	22.65596	0.179347491	+		3	3	3	2.14E-28	2.54E+08	27	59.5	24.115	

Reagent 5

Gene names	log2 LFQ intensity_1	log2 LFQ intensity_2	log2 LFQ intensity_3	St dev log2 LFQ	MG protein	PTM peptide	Razor + unique peptides_1	Razor + unique peptides_2	Razor + unique peptides_3	PEP	Intensity	MS/MS Count	Sequence coverage [%]	Mol. weight [kDa]	
AARS	NaN	21.26808	21.47502	0.146328677			1	2	3	3.06E-15	77577000	13	5.9	106.81	
ABHD17A	NaN	22.45866	22.41527	0.030681363			1	4	2	1.06E-22	2.79E+08	24	33.5	33.989	
ABHD17B	22.00614	22.16457	22.22417	0.112686368			4	2	3	7.99E-26	3.29E+08	22	34	32.214	
ABL2	22.26828	22.39352	22.29793	0.065449253	+		4	5	6	1.43E-35	2.96E+08	46	12.6	115.82	
ACAA2	NaN	NaN	20.4399	N/A	+		1	0	2	1.25E-15	53733000	8	14.6	41.924	
ACACA	27.63416	27.31562	27.13751	0.251612177			45	48	46	5.68E-188	2.45E+09	314	31	265.55	
ACAT1	NaN	NaN	20.79543	N/A			1	1	2	3.69E-18	87102000	17	21.8	45.199	
ACAT2	NaN	NaN	18.19027	N/A	+		0	0	1	0.002185	6737700	3	2.5	41.35	
ACOT7	21.1943	20.74985	21.02039	0.22396889			3	3	3	4.67E-09	56625000	11	14.2	27.041	
ACOT9	23.03361	23.12389	23.20561	0.086035493			7	6	5	4.59E-33	3.51E+08	54	31.3	46.354	
ACTG1;ACTB;ACTA	28.38142	28.05264	28.32126	0.17505819			16	18	17	0	1.28E+10	601	67.5	41.792	
AGPAT1	21.53919	NaN	21.06362	0.336278772			2	1	2	4.89E-11	1.12E+08	30	35.1	18.756	
AHCY	24.56924	24.63666	25.01954	0.242869183			8	7	8	3.52E-54	9E+08	113	34.3	47.716	
AIFM2	24.27299	23.39336	23.90169	0.441590311	+		8	9	8	1.32E-58	7.66E+08	64	44.5	40.526	
AKAP12	26.52067	26.35464	27.12711	0.406621309	+		24	23	29	0	4.84E+09	455	37.4	191.48	
AKR1B1	21.49809	21.52774	21.52678	0.016848146			3	3	4	3.17E-08	93513000	7	11.1	35.853	
ALDH18A1	22.58394	22.57886	22.30327	0.160598518			4	4	5	5.75E-27	2.24E+08	48	14.9	87.088	
ALDH7A1	22.78471	NaN	22.58486	0.14131529	+		7	2	5	1.96E-21	2.11E+08	19	21.5	55.366	
ALDH9A1	21.36896	21.41369	21.10732	0.165488589			3	3	3	1.39E-15	91824000	15	13.2	53.801	
ALDOA	26.22505	26.45138	26.68565	0.230311406			13	13	15	5.06E-187	3.01E+09	221	68.4	39.42	
ALDOC	23.84575	22.95511	22.80312	0.563237352			1	1	1	5.87E-18	2.18E+08	13	8.2	39.455	
ANO6	NaN	20.74763	21.16932	0.298179859			1	3	4	6.48E-20	1.17E+08	21	11.8	103.96	
ANP32A	22.58706	22.63478	22.59835	0.024939284			3	3	3	3.14E-20	1.67E+08	16	29.4	19.997	
ANP32B	21.85924	NaN	NaN	N/A			2	1	1	5.50E-16	85008000	10	17.9	22.276	
ANXA1	24.57144	24.69339	24.71816	0.078540981			7	7	7	5.45E-105	6.1E+08	89	54.3	38.714	
ANXA11	22.25029	22.21685	22.57064	0.195324397			4	4	4	2.02E-11	1.09E+08	26	8.5	51.242	
ANXA2;ANXA2P2	26.96774	27.2238	27.77165	0.410685937	+		16	16	18	6.34E-165	2.65E+09	241	64.3	38.604	
ANXA5	27.97738	28.10149	28.43801	0.238337645			17	19	21	4.45E-256	6.82E+09	647	76.6	35.936	
ANXA6	25.40048	25.16894	25.26799	0.116171765			13	16	17	2.41E-58	6.57E+08	106	42	75.276	
ANXA7	21.85616	22.02503	21.73603	0.145183386			3	4	2	8.89E-12	79585000	13	18.5	37.805	
AP1B1	22.64992	22.7367	23.17426	0.281046247			4	4	4	2.23E-21	1.48E+08	32	10.9	101.35	
API5	23.59161	23.19417	23.80035	0.307946189			6	6	6	4.29E-71	2.21E+08	36	21.8	50.309	
APMAP	22.34652	22.23935	22.57582	0.17188948			4	3	3	1.73E-21	2.41E+08	24	18.3	46.48	
APRT	23.5242	23.14579	23.66134	0.267018511			5	5	6	6.05E-23	3.19E+08	45	49.4	19.608	
ARCN1	20.17146	NaN	20.62623	0.321570951			2	1	2	6.23E-10	44299000	10	8	57.21	
ARF1	29.96761	29.69151	29.73473	0.148510534	+		11	11	11	0	6.23E+10	761	79.6	20.697	
ARF4	27.09637	26.63006	26.83074	0.233907665	+		6	6	6	0	7.01E+09	255	86.7	20.511	
ARF5	25.54569	25.25436	25.35646	0.147820596	+		4	4	5	0	2.77E+09	142	71.1	20.529	
ARF6	25.06554	24.64653	25.0827	0.247018257	+		4	5	5	2.11E-78	2.14E+09	117	60	20.082	
ARHGDIA	22.01798	22.03083	NaN	0.009086322			2	2	1	3.77E-12	92974000	10	23.3	21.517	
ARL1	26.34053	25.50679	25.84967	0.419053024	+		8	10	9	0	4.38E+09	238	80.5	18.565	
ARL4C;ARL7	21.45648	NaN	21.43375	0.016072537	+		2	1	2	1.02E-07	1.17E+08	8	16.1	21.487	
ARL5B	22.07705	NaN	21.65984	0.29501202	+		2	0	2	2.83E-21	2.38E+08	23	21.2	20.374	
ATP1A1	24.44878	24.36393	24.48567	0.062424651	+		12	11	10	3.17E-92	8.85E+08	116	24.8	113	
ATP2A2	23.18643	23.30243	23.33715	0.078928234	+		5	8	10	1.20E-60	4.56E+08	66	21	114.76	
ATP5A1	22.98398	23.63744	23.07465	0.354015914			7	7	8	7.15E-77	7.08E+08	92	40.7	59.75	

ATP5H	23.02185	22.61876	22.84493	0.20204583				2	3	4	2.04E-20	2.06E+08	18	74.5	15.773	
ATP5O	23.71816	23.01686	23.32965	0.351330636				4	4	5	2.48E-24	3.62E+08	48	34.7	23.277	
ATP6V0A2	21.13815	NaN	21.09623	0.029641916	+			2	0	2	3.84E-20	80622000	8	9.2	98.081	
ATP6V1A	20.90764	21.03228	NaN	0.088133789				2	3	1	1.00E-20	61686000	12	8.9	64.735	
ATP9A	22.71517	21.88301	22.31711	0.416210051				6	4	3	8.96E-41	3.19E+08	59	12.7	118.58	
AUP1	21.63883	21.32718	21.31556	0.183377679				2	2	2	4.57E-10	1.02E+08	8	9.4	41.385	
B3GNT1	22.22565	21.25084	21.38953	0.527349679				3	2	3	8.47E-30	2.39E+08	42	33.3	47.119	
BAG5	22.31821	21.6001	22.32575	0.416794663				4	2	4	3.83E-39	2.43E+08	31	33.3	51.199	
BASP1	22.19877	21.15808	20.65674	0.786578455	+			4	5	4	2.37E-200	1.93E+09	95	57.3	22.693	
BAX	22.60738	22.58818	22.8757	0.16074411				3	4	3	2.50E-44	1.69E+08	36	36	18.129	
BCAM	21.56207	NaN	21.95234	0.275962563				3	1	3	1.08E-32	1.9E+08	33	21	67.404	
BCAP31	22.51145	22.11423	22.34879	0.199691595	+			2	4	3	5.54E-18	2.15E+08	35	19.5	27.991	
BZW1	21.93797	21.51388	21.64447	0.217197424				3	2	2	4.91E-10	61522000	18	10.2	40.538	
C12orf23	24.92854	24.59679	24.23213	0.348334577				5	5	5	2.14E-28	4.19E+08	61	62.9	11.748	
C9orf123	NaN	24.09611	23.75256	0.242926535	+			1	2	2	3.61E-57	6.18E+08	36	50.9	11.829	
CAND1	25.00851	24.38337	24.82527	0.321364935				14	18	21	2.82E-106	8.89E+08	132	31.7	136.37	
CANX	28.15967	27.89145	27.9961	0.135184279				19	20	19	0	1.84E+10	767	44.9	67.567	
CAP1	21.71217	22.33813	22.36183	0.368430393				5	5	3	9.88E-15	1.11E+08	18	13.7	51.83	
CAPNS1;CAPNS2	21.12774	NaN	21.47848	0.248010632	+			2	1	2	2.54E-10	78085000	15	37	11.399	
CAPZB	22.10358	22.21457	22.25531	0.078528679				3	5	4	2.22E-13	1.62E+08	25	25.4	29.295	
CAV1	23.30897	23.20819	23.40976	0.100785				5	5	5	1.42E-67	5.71E+08	75	47.2	20.471	
CAV2	20.57126	NaN	20.4207	0.106461997	+			2	1	2	8.33E-08	55929000	11	18.8	16.828	
CCNY	23.13402	22.77557	22.93688	0.17952321	+			8	5	7	1.95E-44	5.2E+08	52	47.5	39.336	
CCT2	24.23936	24.02039	24.09225	0.111619198				9	9	10	4.36E-88	6.61E+08	84	42.1	57.488	
CCT3	23.56162	23.8905	23.95784	0.212009048				6	8	7	7.39E-80	6.01E+08	89	40	55.674	
CCT4	24.45732	24.34442	24.61684	0.136873236				14	13	14	2.70E-75	7.68E+08	105	55.8	57.924	
CCT5	22.43855	22.72862	22.82376	0.200656825				4	6	5	5.33E-38	2.72E+08	48	22.6	59.67	
CCT6A	23.55309	23.738	23.69996	0.097646938				7	11	11	5.77E-76	4.41E+08	63	29.6	58.024	
CCT7	24.194	24.02935	24.00702	0.102119025				9	8	10	4.56E-49	4.72E+08	57	33.1	54.804	
CCT8	25.29502	25.40028	25.5103	0.10764877				17	17	18	6.05E-87	1.16E+09	182	46.5	59.62	
CD151	26.06236	26.16312	26.38052	0.162604389	+			4	5	5	2.39E-50	3.56E+09	181	15.1	28.067	
CD276	24.94905	24.8347	25.56567	0.393194881				8	8	7	2.83E-258	1.83E+09	130	37	57.165	
CD44	23.55215	23.84566	24.15646	0.302196221				5	5	5	4.97E-34	7.63E+08	94	33.5	22.683	
CD47	NaN	20.69583	21.24417	0.387734932				1	2	2	7.11E-07	55427000	10	9.6	31.742	
CD63	25.85928	27.19639	26.84518	0.69320637				6	7	6	4.99E-212	5.65E+09	157	33.5	23.43	
CD81	28.29544	27.67761	27.98114	0.308930645	+			4	4	4	0	1.21E+10	235	50.3	17.963	
CD9	25.11398	25.50739	25.61695	0.264497524				4	5	5	4.90E-220	2.91E+09	196	42.8	17.764	
CDC37	22.73302	22.45839	22.3524	0.196437904				3	3	3	5.76E-15	1.53E+08	26	15.3	44.468	
CDC42	NaN	21.15103	NaN	N/A				1	2	1	6.27E-09	67238000	14	25.7	21.258	
CDCA3	23.46688	24.19099	24.18223	0.415559393	+			6	9	8	3.67E-87	9.61E+08	98	52.2	28.998	
CECR5	21.48773	21.19334	21.20658	0.166275921				3	3	2	7.30E-13	82416000	13	13.5	43.588	
CFL1	24.18911	24.41015	24.6142	0.212601581				5	4	7	1.87E-62	8.73E+08	67	69.1	16.811	
CHCHD3	26.60385	26.69538	26.89655	0.149733318	+			11	11	12	1.22E-222	5.78E+09	281	46.7	26.152	
CHCHD6	24.5657	23.65611	24.2325	0.460181421	+			4	4	4	3.85E-134	8E+08	72	44.7	26.457	
CHMP6	24.91789	24.40814	24.8924	0.287228872	+			3	4	5	5.52E-146	1.43E+09	94	48.3	23.485	
CHP1	25.08962	24.46389	24.45588	0.36359973	+			9	6	8	5.54E-76	1.33E+09	131	67.2	22.456	
CKAP4	26.64072	26.26617	26.64113	0.216364997				17	20	21	0	5.99E+09	480	57.1	66.022	
CKAP5	20.80986	20.53867	NaN	0.191760288	+			2	3	1	6.97E-11	43521000	11	2.9	218.52	
CKB	24.56471	23.58898	23.82046	0.509826782				4	6	7	7.38E-86	6.36E+08	80	52	42.644	
CLIC1	24.06242	23.98007	24.22339	0.123758829				5	6	6	4.85E-41	5.76E+08	69	53.1	26.922	

CLN3	NaN	21.48473	20.94399	0.382360921	+		1	3	2	2.72E-24	1.31E+08	29	21.5	34.568	
CLTA	21.40764	22.16119	22.26144	0.466701565			2	2	2	5.26E-06	74539000	19	7.8	23.662	
CLTC	23.80696	23.79203	23.50033	0.172884235			9	10	12	1.29E-71	4.23E+08	98	15.8	187.89	
CNN3	22.06894	23.24724	23.27796	0.689331073			4	3	4	3.75E-11	72237000	18	16.3	31.38	
COPB1	21.15703	20.97484	21.21715	0.126175636			3	2	2	3.85E-10	75607000	16	4.9	107.14	
COPG1	21.87586	21.08706	21.28218	0.410839222			2	3	3	8.03E-09	72398000	12	4.9	97.717	
COTL1	20.23526	20.18253	20.12477	0.055264079			2	2	2	0.000375	34272000	4	12	15.945	
CPD	24.48505	24.32079	24.39218	0.082363743			11	15	14	7.36E-130	9.89E+08	146	25.1	152.93	
CPNE3	21.71423	21.5254	21.84724	0.161724774			4	4	5	9.04E-14	1.27E+08	22	11.9	60.13	
CS	24.14523	23.75205	23.75062	0.227416508			7	5	7	3.26E-35	6.56E+08	63	24.5	50.431	
CSE1L	25.38627	25.36152	26.07089	0.402600485			13	14	17	5.92E-151	1.44E+09	186	35.7	107.78	
CTNNA1	NaN	NaN	20.67736	N/A			1	1	2	1.59E-07	30602000	5	5.4	88.676	
CXADR	23.43835	23.41738	23.64273	0.124494677			6	7	4	6.57E-93	7.02E+08	58	40.5	40.029	
CYB5R3	27.42238	27.09436	27.30808	0.166502177	+		14	13	16	0	1.22E+10	574	85.7	31.76	
CYSTM1	NaN	NaN	23.95305	N/A			1	1	2	5.15E-08	4.14E+08	33	11.3	10.631	
DAD1	22.14115	22.00439	22.24452	0.120451284			3	3	3	4.48E-11	1.64E+08	21	35.3	9.5541	
DAGLB	24.69885	23.99084	24.11728	0.377599633			7	10	10	1.15E-81	1.08E+09	139	24.7	73.731	
DCAF11	NaN	21.12351	20.9113	0.15005513	+		0	2	2	2.45E-13	86374000	12	12.3	58.846	
DDB1	21.26968	NaN	21.16466	0.074260354			3	1	3	2.28E-15	76194000	19	5.6	121.71	
DDX17	21.79229	21.75711	21.70024	0.046448954			3	5	5	2.65E-21	99665000	25	14	72.371	
DDX39B;DDX39A	24.16867	23.64251	23.69848	0.288979706	+		5	7	9	6.14E-49	5.33E+08	82	29.9	48.991	
DDX46	25.01971	24.89832	24.78027	0.119723882	+		13	15	15	2.70E-41	4.62E+08	87	17.4	117.36	
DEGS1	23.49558	23.0673	23.42714	0.230069778	+		2	3	3	2.35E-38	6.33E+08	55	15.8	37.866	
DEK	21.1128	20.82314	NaN	0.20482055			2	2	1	3.78E-05	26058000	5	6.5	38.704	
DHCR7	21.62819	21.55866	21.41064	0.111109813	+		3	2	4	3.17E-16	1.4E+08	33	12	54.489	
DHX15	21.6063	21.33353	NaN	0.192877517			3	5	2	5.54E-17	87879000	18	8.3	90.932	
DIRC2	22.83239	22.44922	22.41235	0.232598455	+		1	2	2	6.06E-22	2.22E+08	16	10.5	44.145	
DNAJC5	25.85645	25.38564	25.58451	0.236348153			7	7	7	9.45E-289	3.38E+09	275	54.5	22.149	
DNAJC7	20.89674	NaN	NaN	N/A			2	0	0	7.58E-15	47893000	17	20.6	28.704	
DNM1L	NaN	NaN	21.57496	N/A			2	1	2	9.62E-12	79167000	13	6.6	78.099	
DSC1	NaN	23.66416	NaN	N/A			0	3	0	7.66E-12	22112000	5	4.5	93.834	
DSG1	NaN	22.21437	NaN	N/A			0	2	0	3.60E-07	5950200	2	2.4	113.75	
DSG2	21.23602	NaN	NaN	N/A			2	1	1	7.38E-17	87772000	17	6.6	122.29	
DSP	NaN	26.76307	18.05197	6.159677882			1	24	2	1.73E-53	1.89E+08	36	9.3	331.77	
DYM	22.8212	22.72057	22.88891	0.084704778	+		7	6	6	3.98E-34	3.99E+08	68	18.1	75.935	
EBP	NaN	20.7882	21.01414	0.159763706			1	2	2	3.35E-07	1.46E+08	21	9.6	26.352	
ECE1	NaN	20.54915	NaN	N/A			1	2	1	1.73E-07	62583000	12	3.3	85.561	
ECHS1	23.46413	23.11826	23.45751	0.197804804			4	4	5	3.25E-21	2.96E+08	41	24.8	31.387	
EEF1A1P5;EEF1A1	27.15045	27.44277	27.60414	0.229973142	+		11	12	12	2.69E-171	7.65E+09	406	42.6	50.184	
EEF1B2	22.84345	22.44544	23.11507	0.3367971	+		3	3	3	1.50E-38	2.75E+08	28	24.4	24.763	
EEF1D	22.01794	22.32312	22.42242	0.210791917			4	2	4	1.30E-24	1.67E+08	29	31.8	28.821	
EEF1G	25.01572	25.10298	25.11132	0.052951587			8	9	10	1.22E-122	1.47E+09	158	47.6	50.118	
EEF2	27.05383	26.92818	27.09134	0.085455747			30	31	30	2.09E-242	5.11E+09	440	54.8	95.337	
EFR3A	NaN	20.66978	NaN	N/A			1	2	1	1.30E-41	2.06E+08	38	21.3	92.923	
EIF2B1	21.10393	21.5355	21.52602	0.246475997			2	2	2	2.31E-13	68937000	17	17.4	33.712	
EIF2S1	20.53886	20.9036	20.62096	0.191337854			3	2	2	2.19E-10	52197000	13	14.9	36.112	
EIF2S2	20.76975	20.62961	NaN	0.099093944			2	3	1	3.07E-09	56630000	11	10.8	38.388	
EIF2S3;EIF2S3L	20.85354	20.85506	21.01168	0.090866564			2	2	2	1.56E-16	1.11E+08	18	14	51.109	
EIF3A	NaN	NaN	21.61831	N/A			3	2	5	3.50E-24	1.03E+08	19	7.2	166.57	
EIF3C;EIF3CL	22.19893	22.03292	22.26433	0.119294501			4	5	4	1.40E-25	1.44E+08	33	11.2	104.1	

EIF3E	22.31429	22.82792	23.10374	0.400650253			5	5	5	1.05E-25	2.32E+08	46	21.1	52.22	
EIF3F	21.72467	21.7733	21.54952	0.117700105			3	2	3	4.00E-26	1.46E+08	26	25.2	37.563	
EIF3H;EIF3S3	21.31457	NaN	NaN	N/A			2	1	1	7.43E-10	72084000	13	10.8	39.93	
EIF3L	NaN	21.49751	NaN	N/A			2	2	2	3.23E-18	1.11E+08	24	14.2	66.726	
EIF3M	22.28737	22.0961	22.18698	0.095674395			2	2	5	4.22E-15	1.26E+08	22	15.5	42.502	
EIF4A1	25.00457	24.90862	24.9136	0.054016577			12	11	12	4.71E-110	1.2E+09	132	53.2	46.153	
EIF4A3	NaN	20.57819	20.60865	0.021538473			1	2	2	1.00E-14	45677000	9	16.3	46.871	
EIF4G1	22.67872	22.45884	22.65846	0.12152217			8	7	7	1.14E-32	1.58E+08	31	11.1	154.8	
EIF5A	23.10681	22.83024	22.85861	0.152150727			3	3	4	1.03E-21	2.53E+08	32	54.5	16.832	
ENO1	26.16509	25.85377	25.98266	0.156425424			15	16	14	2.03E-225	3.38E+09	290	52.1	47.168	
ERGIC2	22.09979	22.78498	22.94449	0.448784816			4	6	8	3.60E-37	5.08E+08	71	28.4	42.548	
ERGIC3	22.8326	22.60304	22.73521	0.115218282			2	3	3	2.37E-42	3.93E+08	51	26.6	43.222	
ESD	21.01971	21.14277	20.95319	0.096184935			2	2	2	8.50E-13	96215000	14	25.9	31.462	
ETF1	20.21181	20.07601	20.23806	0.086977876			2	2	3	1.62E-07	39365000	6	10.4	45.462	
FABP5	NaN	23.16716	19.16022	2.833334446	+		0	2	2	8.64E-06	21856000	5	14.8	15.164	
FAM129A	22.39055	22.23911	22.26528	0.080943926	+		3	4	4	1.16E-44	3.02E+08	50	14.7	103.13	
FAM129B	25.59949	25.4359	25.40503	0.104506249	+		16	17	16	1.08E-262	2.22E+09	223	42	84.137	
FAM219B	20.95738	21.06553	NaN	0.076473598			2	2	1	4.66E-07	49572000	8	28.3	11.907	
FAM49B	26.06174	25.76337	26.00234	0.157934416	+		12	15	15	0	4.62E+09	348	73.1	36.748	
FAM69B	NaN	NaN	21.59368	N/A			1	0	4	3.25E-38	2.56E+08	36	29	48.582	
FAM84B	21.24307	21.98801	22.36856	0.57249187	+		2	4	5	2.50E-34	2.83E+08	42	31.6	34.474	
FASN	NaN	20.77258	20.82073	0.034047192			2	2	4	5.08E-18	54301000	13	4.3	273.42	
FBXO17;SARS2	21.49047	21.68949	NaN	0.140728392	+		3	2	1	1.82E-23	1.96E+08	26	34.2	31.479	
FDPS	21.78919	21.2052	21.36842	0.301311711			3	2	2	3.96E-08	92928000	14	10.5	40.532	
FKBP4	23.19016	23.00364	23.47531	0.237547477			4	5	6	4.29E-36	3.95E+08	59	33.6	51.804	
FLNA	22.60892	22.27989	22.13207	0.244096093			6	7	6	1.97E-43	1.77E+08	25	7.9	276.55	
FLOT1	23.20546	23.31382	23.34001	0.071334316	+		8	4	8	5.10E-92	6.56E+08	75	49.6	42.08	
FLOT2	25.33064	25.24423	25.24902	0.048565174	+		13	13	13	3.25E-136	2.15E+09	218	51.2	47.064	
FMNL3	NaN	21.09822	NaN	N/A	+		1	2	1	1.05E-25	91132000	21	9.8	117.21	
FSCN1	24.77097	24.47488	24.38528	0.201847395			8	9	10	2.27E-45	4.6E+08	88	25.6	52.262	
FUBP1	21.84648	NaN	21.76426	0.05813832			5	2	5	7.03E-21	1.5E+08	20	17.2	67.56	
FYN	23.47914	22.83731	22.98362	0.33637589	+		4	4	5	1.22E-97	5.6E+08	63	44.5	60.761	
G6PD	20.54019	NaN	NaN	N/A	+		2	1	1	2.32E-07	39799000	9	6	59.256	
GALNT1	NaN	NaN	21.27299	N/A	+		1	1	4	4.33E-15	1.15E+08	17	12	57.379	
GANAB	21.39456	21.4978	22.38105	0.542210227			2	3	2	9.90E-45	2.25E+08	45	18.9	96.215	
GAPDH	25.8505	25.94626	25.79206	0.077849054	+		10	10	10	6.55E-212	2.43E+09	195	63.6	36.053	
GART	NaN	21.8994	NaN	N/A			1	2	1	2.50E-09	46445000	12	9.2	46.033	
GDI1	NaN	NaN	20.72925	N/A			1	0	2	1.45E-19	28235000	6	19.7	50.582	
GDI2	22.42201	23.14277	23.04377	0.390700541			5	7	7	1.48E-35	3.09E+08	38	34.8	50.663	
GHITM	21.91317	22.376	22.1586	0.23155642			3	3	3	5.38E-21	2.13E+08	25	16	35.282	
GLG1	22.02069	21.98235	22.03594	0.02761161			3	2	6	1.40E-34	1.87E+08	25	11.2	134.55	
GLIPR2	20.43807	20.37518	20.16754	0.141572127	+		2	2	2	3.20E-10	66801000	12	20.3	14.213	
GLO1	23.26757	23.44316	23.32704	0.08930508			6	6	4	9.91E-26	4.12E+08	45	50.3	19.043	
GNA11	24.16362	24.06233	24.10406	0.050905878			9	6	7	5.04E-89	1.08E+09	90	50.7	42.123	
GNA13	21.72871	22.03721	22.38764	0.329687271			2	4	4	6.67E-45	3.51E+08	67	34.7	44.049	
GNAI1	23.74386	23.55192	23.31893	0.21279521	+		5	7	8	3.76E-210	7.19E+08	72	45.2	40.361	
GNAI2	25.24231	24.98824	25.14775	0.128411188	+		7	7	8	0	2.35E+09	176	62	40.45	
GNAI3	26.83171	26.68539	26.87021	0.097510875	+		9	10	12	2.06E-231	6.9E+09	372	61	40.532	
GNB1	22.09934	22.29609	22.50703	0.203886154			2	2	2	6.57E-13	2.23E+08	25	12.9	37.377	
GNB2L1	23.95198	23.88157	23.95376	0.041174694			6	7	5	3.02E-46	5.82E+08	60	40.1	35.076	

GOLGA7	21.82345	22.58233	22.42598	0.400704916			3	3	3	7.22E-29	3.35E+08	66	40.1	15.824	
GOLIM4	24.05499	24.33674	24.34327	0.164585875	+		5	7	8	9.67E-193	1.01E+09	107	17.7	81.879	
GORASP1	NaN	21.65075	22.05686	0.287163135	+		1	3	3	3.86E-11	1.68E+08	19	16.3	23.906	
GORASP2	25.56494	25.19163	25.3502	0.187357977	+		6	5	6	9.70E-171	2.91E+09	148	36.9	47.145	
GOT1	20.55234	20.80947	20.20323	0.304280727			2	3	2	3.10E-14	1.09E+08	14	15.7	46.247	
GOT2	23.09913	22.5836	23.2118	0.334938041			5	4	4	1.98E-40	3.13E+08	46	29.5	47.517	
GPI	23.85158	23.37	24.16997	0.40274961			6	6	9	1.84E-50	7.16E+08	79	24.9	63.146	
GPRASP1	NaN	23.01721	NaN	N/A			1	2	1	0.001634	68847000	1	1.5	156.86	
GRPEL1	NaN	21.03611	21.33014	0.207910607			1	2	2	2.01E-05	49739000	4	9.2	24.279	
GSPT1;GSPT2	NaN	21.83237	21.83148	0.000629325			4	3	4	9.09E-15	1.21E+08	12	10.8	68.6	
GSTO1	23.30577	23.29768	23.41041	0.062879555			4	4	5	2.11E-18	3.31E+08	41	30.3	27.566	
GSTP1	25.70643	25.64045	25.81648	0.088929676			5	6	6	1.76E-166	2.4E+09	145	57.6	23.356	
HADHA	22.12408	22.62346	22.70062	0.31297828			4	7	7	9.30E-34	2.24E+08	39	19.3	82.999	
HADHB	23.94029	23.94065	24.03909	0.056938568			9	7	9	1.54E-60	8.77E+08	87	40.3	48.879	
HARS;HARS2	20.78101	NaN	21.10987	0.232539136			3	1	3	6.83E-13	68602000	14	11.1	49.623	
HCCS	25.15306	24.62667	25.06225	0.281384323	+		8	9	9	5.27E-112	1.41E+09	172	60.1	30.601	
HINT1	NaN	NaN	21.04527	N/A			1	1	2	9.21E-08	98861000	13	19.8	13.802	
HIST1H1C;HIST1H	NaN	NaN	21.65158	N/A			1	1	3	1.99E-08	61928000	6	15	21.364	
HIST1H4A	19.75199	20.30095	19.76723	0.312635671			3	3	3	1.63E-22	2.42E+08	21	50.5	11.367	
HLA-B;HLA-C	22.53555	22.23108	22.38908	0.152271382			5	4	4	2.78E-39	2.6E+08	51	26.8	40.46	
HLA-C	NaN	NaN	19.87343	N/A			0	0	2	8.95E-30	30057000	10	26.5	40.648	
HM13	22.47289	23.02425	23.08164	0.336122008			3	4	3	1.24E-32	3.84E+08	48	22.7	36.813	
HMGB1;HMGB1P1	NaN	NaN	22.54033	N/A	+		1	1	4	7.82E-22	1.73E+08	33	30.2	24.893	
HMGCS1	21.24121	21.27287	21.244	0.017529102			2	4	2	6.74E-16	82919000	10	13.1	57.293	
HNRNPA1;HNRNP	NaN	21.70138	21.70168	0.000212132			1	3	3	6.42E-25	1.57E+08	24	38.6	29.386	
HNRNPAB	NaN	20.15239	NaN	N/A			1	2	1	1.05E-07	27971000	6	12.5	30.302	
HNRNPD	NaN	22.07228	NaN	N/A			2	4	3	4.76E-23	2.25E+08	25	46.8	12.553	
HNRNPF	NaN	NaN	21.25816	N/A			1	1	4	3.82E-16	1.2E+08	19	18.1	45.671	
HNRNPK	23.40899	23.04828	23.45814	0.223797768			7	7	8	6.15E-54	4.39E+08	62	37.4	47.557	
HNRNPM	24.00873	24.06653	24.06727	0.033586503			10	17	15	1.10E-68	8.39E+08	106	38.6	73.62	
HNRNPU	21.91689	21.46518	21.81913	0.237655053	+		3	2	5	2.72E-23	1.62E+08	20	8.8	88.979	
HPCAL1	23.48922	23.67325	23.93012	0.221450539	+		5	6	6	1.42E-66	1.03E+09	133	59.6	22.313	
HPRT1	22.47018	21.89855	22.45129	0.324715054			3	3	3	8.38E-27	2.11E+08	36	42.7	24.579	
HSD17B10	22.3904	22.19355	22.25124	0.10119582			4	4	4	2.36E-16	1.51E+08	25	36.8	26.923	
HSD17B12	21.76272	21.878	21.73376	0.076303492			2	2	3	8.83E-29	2.14E+08	30	34	34.324	
HSD17B4	21.18882	NaN	NaN	N/A	+		3	1	2	1.58E-18	86934000	9	11	79.685	
HSP90AA1	25.81846	25.87697	26.0648	0.128703098	+		13	12	14	0	2.06E+09	195	45.4	84.659	
HSP90AB1	27.26177	26.71816	27.3189	0.3315781			23	23	24	0	5.69E+09	458	51.8	83.263	
HSP90B1	25.16046	25.01214	25.10749	0.075162342			15	13	13	2.90E-86	1.02E+09	112	33.6	92.468	
HSPA1A	28.34261	27.97043	28.19268	0.187257407			25	26	26	0	1.05E+10	729	61.2	70.051	
HSPA4	24.23199	23.87961	24.00727	0.178403951			9	10	9	7.24E-63	4.74E+08	72	29.6	94.33	
HSPA5	22.96846	23.29992	23.21539	0.172233116			4	6	6	1.34E-82	5.09E+08	56	28.7	72.332	
HSPA8	26.09834	26.09927	26.3287	0.132730755			16	19	17	1.19E-192	2.5E+09	271	48.5	70.897	
HSPA9	24.47073	24.40944	24.7062	0.156667952			7	9	10	1.52E-78	6.28E+08	86	26.8	73.68	
HSPB1	NaN	21.88573	NaN	N/A			0	2	1	2.27E-10	68430000	9	19.4	20.406	
HSPD1	26.90644	26.70965	26.92965	0.120875286			20	19	21	0	3.46E+09	307	55.7	61.054	
HSPH1	22.20502	21.76405	22.10217	0.230708257			2	5	6	1.87E-20	1.12E+08	17	12.8	92.115	
IDH2	20.50591	NaN	NaN	N/A			2	1	0	9.00E-09	40406000	9	10.2	36.171	
IDH3A	21.97872	21.43043	20.94099	0.519143042			3	2	3	3.39E-16	93357000	18	20.2	35.786	
IFITM2;IFITM3;IFI	25.36349	25.68501	25.5054	0.161127957			3	2	3	3.84E-81	1.95E+09	79	29.5	14.632	

ILF2	21.55829	21.18259	21.5637	0.218488974			2	2	2	6.80E-17	1.21E+08	19	21	43.062	
ILF3	NaN	22.24403	21.746	0.35216039			1	2	7	2.65E-22	1.37E+08	36	15.1	74.606	
IMMT	22.14174	22.01011	22.04933	0.067582386			4	3	4	1.41E-28	1.48E+08	24	13.9	78.973	
IMPDH2	NaN	21.96409	22.12786	0.115802878			1	2	3	6.54E-18	1.48E+08	28	21.6	55.804	
IPO5	22.79917	23.24834	23.51268	0.360722849			4	5	10	5.23E-51	3.51E+08	46	21.6	123.63	
IPO7	22.90237	22.49104	22.84766	0.223369417			4	3	4	2.80E-41	2.86E+08	44	13.1	119.52	
ISYNA1	NaN	20.56644	20.33789	0.161609255			0	2	2	1.02E-08	42013000	8	8.8	44.786	
ITM2B	22.17646	NaN	NaN	N/A			2	0	1	1.31E-46	1.93E+08	18	30.8	30.338	
JAM3	24.06258	23.81646	23.8147	0.142608232			4	2	4	1.64E-44	4.59E+08	47	27.1	35.02	
JUP	NaN	24.24525	NaN	N/A			0	4	1	1.55E-38	74558000	13	19.1	81.744	
KHSRP	23.95917	23.80164	23.97115	0.094598151			9	7	10	1.86E-50	6.38E+08	93	26.9	73.114	
KIAA0319L	NaN	21.62002	21.49346	0.089491434			1	3	3	1.09E-29	1.85E+08	20	7	109.75	
KPNA2	21.85137	21.94303	21.91867	0.047476758			4	4	4	2.04E-17	1.32E+08	21	18.9	57.861	
KPNB1	25.81441	25.9423	25.95587	0.078050121			15	17	16	3.10E-111	1.66E+09	203	35.7	97.169	
LAMTOR1	26.4724	25.89442	26.21795	0.289677218	+		8	10	10	2.99E-230	3.45E+09	334	92.5	17.745	
LBR	NaN	21.13984	21.23122	0.064615418			1	2	3	4.27E-08	1.08E+08	18	4.2	70.702	
LDHA	26.21593	26.11353	26.23403	0.064978997	+		8	10	12	7.36E-182	3.05E+09	215	61.1	36.688	
LDHB	27.03957	27.21827	27.41965	0.190152746			11	12	12	1.15E-180	6.48E+09	312	71.6	36.638	
LMAN2	20.4801	NaN	NaN	N/A			2	1	1	1.24E-08	52113000	8	8.4	40.228	
LMBRD1	22.45746	22.59641	22.8681	0.208865095			2	2	2	4.29E-43	3.9E+08	25	9.9	44.211	
LNP;KIAA1715	24.34848	24.14981	24.24547	0.099357657	+		6	6	8	1.25E-77	1.19E+09	137	40.4	47.094	
LNPEP	24.3057	24.30633	24.52886	0.128660006			8	10	10	3.65E-109	1.06E+09	132	27.7	117.35	
LOH12CR1	22.90275	23.10436	23.13469	0.126070526	+		4	5	3	7.39E-50	4.99E+08	48	64.4	20.247	
LRPPRC	24.7006	24.22612	24.68405	0.269290753			13	15	19	2.26E-75	6.2E+08	91	23.1	157.9	
LRRC1	NaN	20.8137	NaN	N/A			0	3	1	1.12E-54	1.25E+08	19	34.4	59.241	
LRRC57	25.10921	24.69339	25.06114	0.227470533	+		7	7	9	8.54E-69	1.48E+09	128	59	26.754	
LRRC59	20.71129	NaN	20.93589	0.158816183			2	1	3	2.25E-09	78528000	23	10.4	34.93	
LSR	23.91196	24.23345	24.52271	0.305516702			5	8	6	5.70E-101	1.06E+09	107	29.7	69.428	
LTA4H	NaN	21.22629	21.349	0.086769073			1	3	2	2.96E-18	85985000	12	18	69.284	
LUC7L2	22.59243	22.24629	22.59717	0.201226299			2	3	2	1.19E-07	99584000	17	7.9	46.513	
LUC7L3	NaN	22.96941	22.67833	0.205824642			3	4	4	2.26E-13	99675000	14	14.6	42.571	
LYN	23.57819	23.51159	23.78706	0.143727727	+		6	11	10	2.96E-104	8.52E+08	94	43.9	58.573	
LYPLA1	20.91794	21.03342	20.98301	0.057894881			2	3	3	2.27E-11	1.05E+08	12	23.4	20.861	
M6PR	NaN	22.51538	22.6086	0.065916494			2	3	4	3.55E-16	3.16E+08	23	22.4	30.993	
MAPRE1	24.05912	24.3096	25.0566	0.518927673			8	7	5	8.68E-38	5.38E+08	64	46.3	29.999	
MARC2	22.95434	22.0231	23.07858	0.576871093	+		3	2	8	1.70E-45	5.6E+08	58	32.5	38.023	
MARCKS	28.8869	28.95662	28.97528	0.046583435	+		10	10	10	0	3.02E+10	411	48.5	31.554	
MARCKSL1	25.2797	24.32965	24.85343	0.475858278	+		6	4	6	3.32E-200	1.7E+09	76	60	19.529	
MBLAC2	26.07439	25.51021	23.91953	1.117436318			2	3	5	6.01E-35	1.13E+09	41	50.2	31.371	
MCCC1	28.07867	27.7431	27.93764	0.168494558			22	23	24	0	3.94E+09	306	52.8	80.472	
MCM3	20.19568	20.46812	20.43033	0.147598702			2	3	2	1.35E-09	51517000	11	4.3	90.98	
MCM4	NaN	20.45322	NaN	N/A			1	2	1	2.78E-07	36122000	5	4.4	96.557	
MDFIC	21.78959	20.98183	21.0547	0.446812706			2	3	4	4.55E-13	77699000	13	27.6	16.46	
MDH1	24.1536	24.12479	23.97841	0.093940279			6	8	8	1.96E-39	6.28E+08	73	32	36.426	
MDH2	24.60071	24.48536	24.6841	0.09979738			12	10	10	7.75E-70	9.66E+08	139	55.6	35.503	
METTL7B	NaN	NaN	19.74269	N/A			0	1	2	1.21E-08	60108000	13	14.3	27.775	
MGRN1	21.603	NaN	22.31091	0.500567961	+		4	1	4	1.33E-44	3.91E+08	63	35.5	58.304	
MICB;MICA	NaN	NaN	20.39435	N/A	+		0	1	2	0.000288	41059000	7	4.4	37.579	
MIF	24.53931	23.54687	23.94601	0.499375407			2	2	2	5.32E-19	5.92E+08	52	17.4	12.476	
MLEC	22.91936	22.30933	22.46329	0.317238114			4	3	2	2.11E-27	4.31E+08	46	53.4	16.729	

MPDU1	NaN	NaN	19.6377	N/A			0	1	2	8.06E-06	26162000	5	23.8	10.978	
MRPS36	21.81804	22.09832	22.93359	0.580328162			2	3	2	4.24E-57	3.04E+08	38	56.3	11.466	
MSH2	NaN	19.22955	19.60746	0.267222724	+		1	2	3	2.12E-08	23074000	11	4.1	97.321	
MSN	22.46069	22.57073	22.67601	0.107668769			5	6	2	4.58E-42	2.94E+08	32	17	67.819	
MSRA	22.19451	21.93578	21.36116	0.426536747	+		2	2	3	6.79E-21	1.7E+08	22	36.6	23.627	
MTDH	21.71523	21.79058	21.98343	0.138323284			3	3	2	1.77E-46	3.02E+08	38	19.4	63.836	
MTHFD1	22.54272	22.27671	22.78361	0.253553716			2	3	5	2.19E-41	2.5E+08	49	17.3	101.56	
MYL12A;MYL12B;	23.01451	22.59796	22.7872	0.208564745			3	3	3	6.59E-17	1.19E+08	23	24	19.794	
NAA15	21.39535	NaN	21.16023	0.166254946			3	1	4	8.65E-13	72237000	12	5.8	101.27	
NASP	24.28666	24.15345	24.60179	0.230239215			3	5	4	1.36E-25	3.56E+08	49	7.5	85.237	
NCAM1	24.2717	24.67842	24.74212	0.255203783			10	13	12	3.03E-90	1.39E+09	177	34.3	94.573	
NCL	NaN	21.46448	21.853	0.274725127			2	5	4	5.53E-18	1.41E+08	26	9.7	76.613	
NCS1	22.18026	22.38044	22.29238	0.100330695	+		4	4	4	4.15E-44	4.79E+08	83	62.6	21.878	
NCSTN	20.97162	21.5364	21.47887	0.310802379			3	2	3	1.28E-15	1.04E+08	16	10.4	50.26	
NDUFA4	22.56221	22.20093	22.60702	0.222650796			3	2	2	2.60E-09	1.34E+08	16	46.9	9.3697	
NDUFAF4	23.93733	24.52773	24.71565	0.406133826	+		9	6	5	6.10E-46	1.41E+09	104	53.1	20.266	
NDUFB7	24.36366	24.06924	24.28376	0.152253066	+		5	4	5	3.39E-56	9.65E+08	161	51.1	16.402	
NME2;NME1-NME	22.09973	23.87296	24.17691	1.121859096			2	5	6	9.40E-34	7.37E+08	88	58.4	30.137	
NPC1	23.58302	23.50373	24.15561	0.355690349			7	6	5	9.01E-40	6.55E+08	71	9.3	142.17	
NPEPPS	22.90435	22.30805	22.72096	0.305423272			4	4	5	2.06E-43	2.83E+08	56	20.1	102.99	
NPM1	23.77394	24.18382	24.37451	0.306879079			4	3	5	1.66E-27	5.02E+08	54	26.8	29.464	
NRAS;KRAS	22.4948	NaN	NaN	N/A			2	1	1	5.52E-21	2.44E+08	20	31.7	21.229	
NSUN2	NaN	NaN	20.53193	N/A	+		1	0	2	6.40E-09	41635000	8	5.6	82.392	
OAT	22.54191	22.63321	22.80747	0.134922417			4	3	5	9.80E-21	2.17E+08	37	17.8	48.534	
OGFRL1	NaN	22.14666	22.52927	0.270546126	+		2	4	5	7.51E-42	2.54E+08	28	26.6	51.251	
P4HB	22.6154	22.45871	22.44567	0.094454637			5	5	7	3.70E-32	2.61E+08	37	24.8	57.116	
PA2G4	24.17226	24.14895	24.05225	0.063635218			6	8	9	5.62E-45	6.59E+08	82	32	43.786	
PABPC1	NaN	22.06408	22.18195	0.083346676	+		1	2	3	4.81E-22	1.81E+08	24	17.4	61.18	
PAICS	22.2067	21.63272	22.094	0.304119924			3	3	4	2.21E-45	2.98E+08	50	36.5	47.079	
PALM	22.58116	22.4997	22.20565	0.197530122			3	3	4	2.48E-13	2.27E+08	31	14.2	42.075	
PARP1	21.83125	21.60359	22.107	0.25208754			5	5	5	5.75E-18	1.29E+08	10	8.4	113.08	
PC	29.44308	29.18787	29.09751	0.179218534	+		44	46	44	0	9.94E+09	976	54.9	129.63	
PCBP1	23.37398	23.5248	23.2078	0.158562009			5	6	6	2.17E-35	5.24E+08	48	37.4	37.497	
PCBP2	NaN	23.50191	NaN	N/A	+		1	2	1	1.49E-32	4.02E+08	40	31.9	38.15	
PCCA	28.56056	28.52515	28.39473	0.087333492			31	32	34	2.86E-259	5.69E+09	474	66.1	77.047	
PCCB	23.00705	22.69729	22.66659	0.188328949			7	8	7	5.88E-21	1.18E+08	25	21.8	52.423	
PCMTD2	24.20113	24.00359	24.36152	0.179286032	+		9	10	11	1.14E-75	1.06E+09	148	41.6	41.071	
PCNA	22.39702	22.5087	22.52203	0.068650821			3	3	3	2.97E-33	2.61E+08	27	47.1	28.768	
PDCD6IP	21.47586	21.21857	21.63272	0.209094646			3	4	4	2.39E-14	74616000	16	7.9	96.022	
PDHA1	NaN	19.67112	20.17572	0.356806082			1	2	2	0.000108	24946000	6	5	40.188	
PDHB	NaN	NaN	20.7921	N/A			1	1	2	1.07E-11	72446000	13	15.5	37.514	
PDIA3	22.33593	22.11499	22.65873	0.273455515			8	7	5	6.59E-31	3.27E+08	34	26.2	54.963	
PEBP1	21.63378	22.44288	21.88844	0.413702437			2	2	2	5.73E-31	2.5E+08	25	56.1	21.057	
PFDN2	NaN	20.72941	NaN	N/A			1	2	1	1.10E-05	19221000	2	14.9	16.648	
PFN1	24.52049	24.3152	24.50791	0.115064753	+		5	6	6	3.43E-39	8.26E+08	89	60	15.054	
PGAM1	24.20322	24.32306	24.87375	0.357591849			5	10	9	1.57E-138	8.34E+08	138	64.2	28.804	
PGD	NaN	NaN	22.26264	N/A	+		3	3	5	5.96E-32	1.53E+08	23	31.9	51.872	
PGK1	26.16429	26.0206	26.36416	0.172543862			15	16	16	2.09E-152	2.27E+09	202	60	44.614	
PGLS	NaN	21.28585	21.67784	0.277178787			1	3	4	3.99E-18	1.36E+08	20	45.7	27.547	
PGM1	NaN	20.06224	NaN	N/A			1	3	1	9.58E-07	33366000	4	6.8	61.448	

PGRMC1	21.53592	NaN	21.94049	0.28607419			2	1	2	3.00E-10	1.07E+08	10	20	21.671	
PHB	NaN	22.58339	22.62703	0.03085814			1	4	5	2.23E-16	1.64E+08	24	30.3	22.27	
PHGDH	24.01461	23.84345	24.07316	0.119366754			9	9	10	9.28E-66	5.38E+08	70	22.1	56.65	
PI4K2A	26.11118	25.85743	25.80391	0.164148462			21	23	21	0	3.57E+09	312	72.7	54.022	
PI4K2B	23.32677	23.26198	23.40561	0.071929441			9	6	10	9.25E-55	5.78E+08	72	38.5	54.744	
PIK3R4	22.87931	NaN	22.5443	0.236887843	+		5	1	6	3.04E-44	3.01E+08	41	13.8	153.1	
PITPNB	NaN	20.32476	20.20323	0.085934687	+		1	2	3	1.92E-06	35896000	5	11.8	31.54	
PKM;PKM2	25.93015	25.72507	26.14221	0.208579733			13	18	18	4.64E-254	2.73E+09	244	55.4	57.936	
PKP1	NaN	22.40104	NaN	N/A			0	4	1	1.50E-42	32854000	7	6.2	80.496	
PLGRKT	21.95479	22.57685	21.97292	0.354028903	+		2	3	4	8.84E-31	6.36E+08	58	34	17.201	
PLIN3	22.76624	22.80721	23.21598	0.248675666			5	5	5	6.89E-20	1.37E+08	24	16.8	45.803	
PLP2	24.74422	24.87746	25.49823	0.402417563			2	2	2	7.94E-58	2.08E+09	53	27	16.691	
PLS3	26.23451	26.07323	26.36097	0.144220709			18	23	22	5.61E-154	2.43E+09	264	47.9	70.81	
PLSCR1	25.38472	25.41012	25.54265	0.084804919			5	6	7	3.70E-91	1.96E+09	137	34.7	34.217	
PLSCR3	22.60198	22.48171	22.33301	0.134735187			2	3	5	6.59E-22	2.71E+08	36	26.1	31.648	
PMPCA	NaN	20.86219	20.55272	0.218828336			1	3	4	1.09E-26	71790000	14	9.1	58.252	
PMPCB	20.52745	20.75743	20.69285	0.118616003			3	2	2	2.59E-10	61659000	9	6.7	54.366	
PNP	NaN	20.81378	20.86627	0.037116035			1	4	2	2.36E-15	72023000	13	25.3	32.118	
PODXL	21.73182	21.92557	NaN	0.137001939			3	3	1	1.04E-07	1.31E+08	10	5.5	55.385	
PPIA	24.9593	24.41963	24.8147	0.279354367			6	7	6	6.20E-36	1.04E+09	80	52.7	18.012	
PPM1A	25.08521	25.17146	25.29158	0.103647201	+		7	9	10	1.82E-170	2.21E+09	201	52.9	42.447	
PPM1B	24.37305	23.76668	24.20382	0.312893685	+		6	6	8	3.95E-127	1.2E+09	101	45.5	52.642	
PPM1G	27.27755	27.22223	27.36928	0.074272469	+		19	17	19	0	1E+10	538	63.9	59.271	
PPP2R1A	24.76213	24.86188	24.99873	0.118783799			12	12	14	1.89E-64	7.77E+08	100	39.4	65.308	
PPP3CA	21.56584	NaN	21.89	0.229215734			3	1	3	1.52E-21	1.83E+08	26	17.2	57.658	
PPP3R1	24.56483	24.1876	24.19227	0.216458324	+		5	6	5	8.28E-69	1.21E+09	105	84.7	19.3	
PRAF2;WDR45	22.62765	22.83682	23.09076	0.231915389			2	2	2	1.65E-12	3.82E+08	38	26.4	19.258	
PRDX1	25.7357	25.44632	25.79342	0.185988706			7	6	8	1.50E-46	1.92E+09	93	53.3	22.11	
PRDX2	22.59805	23.19028	22.85523	0.296967009			3	3	3	2.54E-15	2.63E+08	43	19.2	21.892	
PRDX3	NaN	21.31213	21.79622	0.342303322			1	4	4	2.50E-16	1.37E+08	20	31.5	25.838	
PRDX6	23.69104	23.02582	23.57184	0.354698024			6	6	6	1.10E-51	4.38E+08	73	50.9	25.035	
PRKAA1	21.87474	22.01604	21.98409	0.074098937			3	4	3	8.35E-25	2.47E+08	38	20	64.009	
PRKAB1	23.73417	23.26814	23.25436	0.273127408	+		4	4	2	1.57E-105	8.52E+08	74	51.5	30.382	
PRKACA	25.95602	26.26082	26.50771	0.276351096	+		12	15	16	1.49E-121	4.92E+09	322	63.2	40.589	
PRKACB	22.26018	22.46643	22.7177	0.229128867	+		2	2	2	3.38E-84	4.63E+08	55	62.7	40.622	
PRKAG1	NaN	NaN	20.78979	N/A	+		0	1	2	1.49E-12	70729000	9	20.2	28.285	
PRKDC	22.93141	22.95061	23.19147	0.144921463			9	10	11	3.00E-48	2.71E+08	56	5.8	469.08	
PRMT1	21.51287	NaN	21.47477	0.026940768	+		3	2	3	5.30E-21	1.38E+08	19	25.5	37.709	
PRNP	21.60273	21.59769	21.12635	0.273594804			2	4	3	1.08E-22	1.97E+08	30	21.5	26.885	
PROCR	21.26591	NaN	21.36922	0.073051202			2	1	2	4.74E-21	1.9E+08	23	20.6	26.671	
PRPF19	21.34365	21.27469	21.26882	0.041612233			3	3	3	4.07E-13	88949000	9	8.7	55.18	
PRPF38B	22.6709	22.32199	22.58484	0.181766624			3	4	4	1.13E-13	95439000	16	10.3	64.467	
PSAT1	NaN	NaN	19.6627	N/A			1	1	2	4.57E-06	28048000	4	7.6	40.422	
PSMA1	21.06618	NaN	21.16987	0.073319902			2	1	2	1.52E-13	1.21E+08	13	21.3	29.555	
PSMA3	21.56486	21.47502	NaN	0.063526473			3	3	0	1.19E-12	93841000	16	15.3	27.647	
PSMA4	22.17368	22.13977	22.01202	0.085248564			2	2	2	4.97E-12	1.39E+08	23	23.2	24.526	
PSMA6	21.23964	21.4835	NaN	0.17243506			2	2	1	4.80E-16	1.26E+08	22	27.7	16.645	
PSMA7	NaN	NaN	21.30697	N/A			1	1	2	5.66E-07	70511000	6	12.9	27.887	
PSMB2	21.51937	21.30279	21.58431	0.147409639			2	3	3	2.48E-07	1.12E+08	12	14.4	22.836	
PSMB5	21.17505	21.43069	21.19322	0.142638213			2	3	3	2.35E-23	1.34E+08	24	30.4	28.48	

PSMB6	21.07405	21.31849	21.46349	0.196824553			3	3	3	6.56E-10	1E+08	16	13	25.357	
PSMC1	27.67438	27.56034	27.78588	0.112772384	+		18	20	19	0	1.32E+10	755	63	49.184	
PSMC2	24.56494	24.01504	24.16883	0.28370892			7	9	10	1.29E-82	1.22E+09	91	50.8	48.633	
PSMC5	24.71156	24.36626	24.66471	0.187305194			3	2	3	3.54E-25	1.28E+09	61	18.3	44.784	
PSMC6	21.62823	21.68974	21.37888	0.164617153			2	2	2	9.29E-26	1.35E+08	23	24.2	44.172	
PSMD1	22.25514	22.12105	22.04016	0.108581552			4	2	4	6.08E-17	1.38E+08	18	7.3	102.26	
PSMD11	21.14283	21.13952	21.54778	0.23475934			2	2	4	5.40E-15	95605000	20	14.2	47.463	
PSMD12	NaN	21.72966	21.64417	0.060450559			1	2	3	3.20E-17	1.09E+08	28	14	52.904	
PSMD13	22.67157	22.40844	22.19629	0.238095253			3	5	4	3.41E-30	1.83E+08	35	24.7	42.945	
PSMD14	NaN	NaN	20.25488	N/A			1	1	2	3.58E-07	39075000	6	6.5	34.577	
PSMD2	23.53967	23.75501	24.00488	0.232818483			7	8	6	5.34E-103	6.21E+08	92	31.1	100.2	
PSMD3	20.82244	NaN	20.99535	0.122265834			2	1	3	1.43E-17	95938000	13	16	41.183	
PSMD6	NaN	NaN	21.45327	N/A			2	2	3	2.35E-14	79084000	11	20.3	45.531	
PSMD8	21.6163	21.94935	21.98284	0.202647253			2	2	3	1.70E-13	1.18E+08	23	29.7	19.781	
PTGES3	NaN	NaN	21.76288	N/A			1	1	2	3.72E-06	71886000	9	20.9	16.476	
PTGFRN	26.02931	26.03959	26.39617	0.208902383	+		17	20	21	2.36E-238	3.47E+09	299	43.5	98.555	
PTK7	22.91231	22.76939	23.57092	0.427520682	+		6	5	9	5.17E-82	6.62E+08	83	25	118.39	
PTPLAD1	23.68389	23.55918	23.61785	0.062391285			5	6	6	1.61E-45	6.68E+08	68	29.8	43.159	
PTTG1IP	22.24086	NaN	NaN	N/A	+		1	1	1	2.29E-11	2.22E+08	34	22.8	20.324	
PVR	24.09039	24.0147	23.92298	0.083832812			3	3	4	3.01E-21	6.87E+08	43	13.5	39.304	
QKI	NaN	NaN	21.12029	N/A			0	0	2	1.21E-07	75631000	12	10.1	35.232	
RAB14	20.51826	20.07091	20.13646	0.241588516			2	2	2	0.000455	30018000	4	7.9	23.897	
RAB1A;RAB1B;RAI	NaN	NaN	22.40888	N/A			1	2	2	1.52E-14	2.11E+08	17	37	19.018	
RAC1;RAC3;RAC2	NaN	NaN	21.86922	N/A			2	1	2	7.78E-12	1.24E+08	16	27.1	21.45	
RAD23B	22.63103	23.40523	23.89125	0.63557789			6	5	6	2.04E-15	1.52E+08	21	21	43.171	
RAN	24.1418	24.36058	24.53902	0.198951104			3	5	6	1.60E-29	7.34E+08	68	31.5	24.423	
RANBP1	23.15917	22.48603	22.42721	0.406682246	+		2	3	2	1.70E-12	2.34E+08	21	15.4	23.31	
RANGAP1	22.12288	22.43527	22.56906	0.22897011			5	5	4	2.84E-35	2.18E+08	44	21.3	63.541	
RAP2B	24.66899	24.08367	24.25883	0.300419638			6	8	7	1.44E-47	1.13E+09	103	62.3	20.504	
RAP2C	NaN	21.59131	21.74148	0.106186225			1	2	2	8.60E-42	1.87E+08	24	59	20.745	
RBM39	NaN	NaN	20.5243	N/A			2	2	2	2.95E-09	43638000	11	13.7	36.514	
REEP5	23.4073	23.22874	23.43403	0.111611044			3	2	3	2.29E-30	3.68E+08	49	15.9	21.493	
RFTN1	NaN	NaN	21.08044	N/A	+		1	1	3	1.10E-18	1.28E+08	17	14	63.145	
RGS19	NaN	22.25148	22.0046	0.174570522			2	3	3	1.36E-08	1.78E+08	16	16.1	24.635	
RHBDD2	20.29637	19.7344	NaN	0.397372798			2	2	1	2.05E-09	71679000	20	11.8	39.202	
RHOB	23.00828	23.25436	23.55204	0.272287742			3	2	3	5.78E-22	5.85E+08	52	36.2	22.123	
RNF141	23.81558	23.84288	23.97989	0.088048099	+		4	5	5	2.54E-57	8.38E+08	80	57.4	25.535	
RNH1	20.17779	21.10534	20.38469	0.486910847			2	3	2	1.43E-08	32885000	5	11.1	49.973	
RP2	24.73071	24.33994	24.44291	0.202539236	+		7	6	6	3.04E-38	1.43E+09	115	26.9	39.641	
RPA2	20.34787	20.44364	20.4914	0.073091007	+		2	2	2	0.000263	40789000	9	11.2	19.433	
RPL10	24.39944	24.38864	24.3843	0.007796315	+		5	5	5	7.64E-22	4.6E+08	24	35.5	22.975	
RPL10A	22.89169	22.69748	23.317	0.316862523			4	3	4	2.39E-14	2.93E+08	30	26.7	24.831	
RPL11	22.59457	NaN	NaN	N/A			2	1	1	1.67E-78	2.37E+08	24	22	20.124	
RPL12	22.13602	NaN	NaN	N/A			2	1	1	3.58E-35	2.19E+08	29	54.5	17.818	
RPL13	29.45662	29.1632	29.52526	0.192307995			11	13	12	0	1.11E+10	425	46.9	24.261	
RPL13A	26.42044	26.57053	26.44788	0.079919735			5	5	7	3.89E-25	1.72E+09	144	30	23.577	
RPL14	22.50901	22.35539	22.63832	0.141638957			2	2	3	1.42E-12	1.56E+08	25	27.4	14.558	
RPL15	28.31825	28.30674	28.32693	0.010128002	+		11	15	16	7.79E-90	6.07E+09	363	52.5	24.146	
RPL18	29.6968	29.64561	29.63029	0.034829821	+		10	9	11	6.72E-251	1.45E+10	493	54.3	21.634	
RPL18A	23.22657	23.86646	24.0017	0.414040172			3	4	4	1.25E-17	3.14E+08	33	27.7	16.714	

RPL19	26.20006	25.83425	25.78447	0.226939806	+			4	5	5	2.32E-38	1.15E+09	64	22.3	23.134	
RPL23	24.63887	23.89514	24.26835	0.371865811				5	5	5	9.67E-32	5.17E+08	48	43.6	14.865	
RPL24	23.35313	23.48971	24.1035	0.39967641				5	5	4	1.08E-14	3.21E+08	36	39.7	14.369	
RPL27	21.47531	21.69038	21.77901	0.156175154	+			2	3	2	3.10E-10	1.47E+08	28	20.6	15.798	
RPL27A	26.69259	26.59861	26.56366	0.066679192				4	5	5	2.39E-23	1.8E+09	126	35.8	16.561	
RPL28	28.24277	27.79712	27.74964	0.272040276				13	13	12	7.10E-92	4.38E+09	195	61.3	15.747	
RPL29	26.57199	26.68846	26.57168	0.067333654				3	3	4	1.11E-21	1.78E+09	45	23.3	17.752	
RPL3	23.70546	24.5041	24.27711	0.411528458				6	10	11	4.95E-48	6.26E+08	72	33	46.108	
RPL31	26.529	26.22643	26.19972	0.182887646				4	4	4	4.45E-157	1.33E+09	79	32.8	14.463	
RPL32	30.21755	30.25564	30.19108	0.03245382				11	11	12	2.10E-291	2.9E+10	1376	60.2	15.616	
RPL34	28.83379	28.34079	28.85546	0.291090993				9	9	9	6.28E-37	6.21E+09	189	35.9	13.293	
RPL35	25.04589	25.36603	25.19978	0.160109761				3	4	4	5.74E-39	7.34E+08	61	29.3	14.551	
RPL36	28.43821	28.23119	28.24186	0.11656504				8	8	8	2.39E-70	5.55E+09	202	43.8	12.254	
RPL36A;RPL36AL	22.7303	22.64685	22.70948	0.043435569				2	3	2	1.62E-08	1.25E+08	26	32.1	12.441	
RPL4	25.92841	26.24447	26.29511	0.198715552				10	10	12	1.90E-66	1.96E+09	173	45.9	47.697	
RPL5	23.08808	23.08153	23.30703	0.128343455	+			5	4	5	2.65E-37	4.71E+08	36	35	34.362	
RPL6	26.17503	26.59747	26.48378	0.218596863				10	10	11	2.23E-60	2.27E+09	215	42.7	32.728	
RPL7	24.16131	24.18101	23.95003	0.128048877				5	5	6	4.70E-28	5.22E+08	51	34.7	29.225	
RPL7A	22.89141	23.99352	23.85861	0.601154861				5	5	6	4.33E-24	4.72E+08	43	36.8	29.995	
RPL8	22.52803	23.60433	23.53564	0.602552614	+			4	3	3	2.66E-24	3.7E+08	47	20.2	28.024	
RPL9	NaN	NaN	22.3365	N/A				1	1	2	7.12E-18	1.7E+08	26	33.3	21.863	
RPLP0;RPLP0P6	24.78132	24.77821	25.04972	0.155866349				8	6	8	5.74E-73	9.1E+08	121	42.3	34.273	
RPRD1B	21.57893	22.63243	22.99996	0.737593702				2	3	3	8.37E-09	38774000	6	11.7	36.899	
RPS11	22.07725	22.61394	22.77169	0.364043984				4	6	6	3.49E-19	2.43E+08	32	51.3	18.431	
RPS12	NaN	22.16106	NaN	N/A				1	2	2	2.18E-18	1.77E+08	28	31.8	14.515	
RPS14	NaN	22.82108	23.09171	0.191364308				1	3	3	3.13E-22	2.63E+08	34	31.8	16.273	
RPS15A	23.29123	23.328	23.48198	0.101199008				3	3	4	2.46E-16	3.77E+08	32	54	11.477	
RPS16	22.4902	22.1717	22.22738	0.170106273				4	5	3	1.02E-20	2.76E+08	41	42.6	14.419	
RPS17L;RPS17	20.72775	20.21229	20.36271	0.265072125	+			3	2	3	1.09E-09	48831000	10	48.9	15.55	
RPS2	24.57184	24.75974	24.92927	0.178793659				8	9	9	5.94E-44	8.74E+08	96	42.7	31.324	
RPS23	23.951	23.67012	23.28884	0.332346175	+			2	3	4	3.95E-15	2.64E+08	51	28.7	15.807	
RPS25	24.17035	24.26964	24.81622	0.347792145				3	3	4	1.89E-10	3.92E+08	48	28	13.742	
RPS26;RPS26P11	23.47073	23.40523	23.44417	0.032944416				2	2	2	8.42E-07	2.47E+08	17	20.9	13.015	
RPS3	24.95642	24.71775	25.03192	0.163995126				12	10	10	2.23E-58	1.29E+09	114	70.8	26.688	
RPS3A	25.02141	24.83108	25.07472	0.128080648				9	11	11	5.59E-45	1.15E+09	95	52.7	29.945	
RPS4X	24.18533	24.23637	24.32697	0.071734849				10	10	9	3.27E-46	6.52E+08	84	47.1	29.597	
RPS5	24.91966	24.8805	24.82026	0.050071155				8	10	10	2.45E-60	7.69E+08	99	54	22.391	
RPS6	25.32062	24.7682	25.03061	0.276324889				4	4	4	5.45E-46	6.43E+08	66	18.9	28.68	
RPS8	24.83985	24.33599	24.49344	0.257767754	+			6	7	7	3.91E-72	5.6E+08	85	43.3	24.205	
RPS9	28.16101	28.4556	28.33083	0.147867989				15	13	15	2.54E-61	5.61E+09	278	50.5	22.591	
RRAS2	24.05308	23.60693	23.59958	0.259732585				4	4	6	3.27E-37	8.28E+08	62	47.5	23.399	
RRM1	22.21573	21.78053	22.04293	0.219131863				5	6	4	1.16E-22	1.53E+08	25	13.5	79.219	
RTN4	22.85929	22.59858	22.8064	0.13781401				2	3	3	2.39E-18	3.79E+08	33	20	37.144	
RUVBL1	NaN	20.78021	NaN	N/A				1	2	2	3.14E-17	79579000	14	18.6	50.227	
RUVBL2	NaN	22.16561	22.0553	0.078000949				2	4	4	2.07E-20	1.46E+08	19	22.2	51.156	
S100A11	22.32688	NaN	22.34516	0.012925912				2	1	2	3.09E-09	1.01E+08	12	37.1	11.74	
SAMM50	23.15748	22.76733	22.67913	0.254563347	+			6	7	7	9.07E-36	4.25E+08	40	32.2	51.976	
SCAMP1	23.73107	23.9581	24.22376	0.246597274				2	3	4	4.82E-64	5.7E+08	61	27.2	37.873	
SCAMP2	24.17912	24.47556	23.81909	0.328747911				2	3	2	1.88E-99	1.02E+09	65	13.7	36.648	
SCAMP3	26.3431	25.86698	26.08962	0.23822641				6	5	7	3.89E-222	3.12E+09	222	38.9	38.287	

SCARB1	22.55677	22.46361	22.97158	0.27042548	+		4	4	4	1.53E-27	2.76E+08	34	19.8	53.579	
SCARB2	25.94996	25.55151	25.83949	0.205709546	+		7	10	10	1.66E-132	2.9E+09	170	37.4	54.29	
SCARF2	NaN	20.98496	NaN	N/A			1	2	1	2.19E-13	87420000	12	5.7	91.815	
SCRIB	22.09439	21.84575	22.01913	0.127505909			4	4	6	9.44E-47	2.73E+08	50	10.9	174.88	
SDHA	NaN	NaN	20.66848	N/A			0	1	2	6.04E-08	46273000	8	4.5	63.566	
SEC61A1;SEC61A2	22.65075	22.81427	23.02024	0.185150971	+		4	5	4	1.48E-18	3.22E+08	35	12.4	52.264	
SERBP1	NaN	NaN	20.58141	N/A			1	1	2	3.69E-17	57828000	12	12.9	42.426	
SERINC1	24.37551	24.13891	24.35198	0.130340609	+		3	3	2	1.04E-99	1.03E+09	61	10.4	50.494	
SERPINB6	22.0352	22.23882	NaN	0.143981083			2	3	2	5.20E-31	2.78E+08	38	39.4	42.621	
SFT2D3	NaN	23.40185	23.57762	0.124288159			1	1	3	6.38E-56	5.28E+08	46	27	21.789	
SFXN1	22.32053	22.29603	22.44683	0.08092443			3	4	4	3.46E-56	2.4E+08	40	35.1	35.619	
SGTA	NaN	NaN	21.43634	N/A			3	3	5	3.08E-10	51623000	9	17.3	34.063	
SHISA2	21.33838	21.19069	21.41488	0.113963256			2	2	2	4.51E-12	96252000	9	15.6	31.375	
SHMT2	23.60479	23.55403	23.66546	0.055788397	+		9	11	10	4.41E-48	5.47E+08	84	35.6	53.454	
SLC17A5	20.95291	20.90925	21.33101	0.231929337			2	2	4	1.10E-13	1.58E+08	26	9.3	54.639	
SLC19A1	NaN	NaN	20.4801	N/A			1	1	2	1.35E-05	54581000	9	3.9	64.868	
SLC1A4	NaN	NaN	21.48335	N/A			0	0	2	4.31E-23	1.36E+08	16	17.7	55.722	
SLC1A5	25.29249	24.94731	25.23739	0.185441657			12	12	12	1.12E-218	2.31E+09	223	35.3	56.598	
SLC25A24	NaN	NaN	19.90038	N/A			1	1	2	3.71E-10	35974000	6	11.1	51.354	
SLC25A3	23.46551	23.39074	23.70841	0.166084914			2	3	4	1.31E-19	5.6E+08	45	19.7	39.958	
SLC25A4	21.40484	NaN	21.44985	0.031826876	+		2	1	2	4.96E-48	59818000	10	36.2	33.064	
SLC25A5	26.47877	26.23469	26.48364	0.14234633			10	11	10	8.87E-68	3.07E+09	207	42.3	32.852	
SLC25A6	22.90434	22.86705	22.96277	0.0482475			2	2	3	6.07E-53	2.81E+08	32	41.9	32.866	
SLC30A1	22.51605	22.48417	22.58568	0.051911706	+		3	3	3	2.42E-49	3.63E+08	36	19.7	55.299	
SLC35B2	24.61751	25.11064	24.98559	0.256351837			8	8	10	1.41E-51	1.81E+09	159	33.7	42.127	
SLC35F6;C2orf18	NaN	NaN	20.8848	N/A	+		1	1	2	1.31E-17	67738000	7	5.7	40.214	
SLC38A1	22.44761	22.88842	22.99683	0.290891959			4	6	6	2.42E-37	4.13E+08	34	10.7	54.047	
SLC38A2	24.18117	24.05879	24.1453	0.062911977			3	3	3	3.92E-116	8.95E+08	80	17.6	56.025	
SLC39A10	NaN	21.64289	20.45262	0.841647988			0	2	2	1.34E-08	65422000	11	4.5	94.131	
SLC3A2	21.39864	21.43262	21.59003	0.102113238			2	2	3	8.84E-24	1.36E+08	25	15.7	57.944	
SLC41A3	NaN	20.69608	20.88852	0.136075629			1	2	3	3.59E-19	1.03E+08	17	30.3	16.43	
SLC44A1	25.52286	25.47299	25.63184	0.081237286	+		12	13	14	5.52E-105	2.31E+09	198	28.3	73.301	
SLC44A2	21.63471	NaN	22.40497	0.544656069	+		3	1	2	1.85E-18	2.18E+08	28	8.5	79.845	
SLC5A6	21.71812	20.99955	21.50799	0.369461025			2	2	2	7.31E-15	1.66E+08	21	8.5	68.641	
SLC7A1	23.23687	23.50506	23.42969	0.138314898	+		4	3	3	3.35E-89	4.67E+08	33	14.8	67.638	
SLC7A2	22.44314	22.3136	22.2577	0.09512572			6	5	5	8.12E-34	2.85E+08	22	16.1	71.672	
SLC7A5	21.57999	21.57205	21.85018	0.158336128			2	2	3	4.19E-17	1.49E+08	19	11.2	55.01	
SLC9A6	NaN	21.10891	20.61045	0.352464446			1	2	3	4.50E-22	1.04E+08	16	12.3	72.259	
SMS	21.77712	21.9355	22.26616	0.249526345			2	2	3	2.30E-10	1.38E+08	20	14.7	35.278	
SNAP23	25.24133	25.23856	25.29288	0.030593403			6	10	11	3.56E-114	1.99E+09	175	76.3	23.354	
SNRNP70	24.15182	23.49936	24.46712	0.493571602			7	6	8	3.76E-20	2.42E+08	22	29.7	51.556	
SPECC1	21.07555	NaN	21.23526	0.112932024	+		2	1	2	7.44E-09	68971000	8	5.5	79.014	
SPRYD7	NaN	21.8278	21.83067	0.002029396			1	2	3	2.07E-33	2.94E+08	38	45.9	21.666	
SRC	25.5934	25.31167	25.53041	0.147866336	+		12	15	17	2.08E-186	3.11E+09	243	51.5	59.834	
SRP68	21.24336	21.26865	21.11553	0.082083116	+		3	3	3	1.48E-10	70576000	9	8.2	60.284	
SRRM2	31.0181	30.9652	30.92408	0.047132835			80	81	82	0	3.55E+10	2305	39.3	299.61	
SRSF11	22.87217	21.87553	22.01628	0.539390017			2	1	2	2.51E-08	95271000	13	10	42.316	
SRSF6;SRSF5;SRSF	21.56853	21.60137	21.64262	0.037124467			2	2	2	1.98E-05	42883000	5	5.4	38.418	
ST13;ST13P5;ST13	23.51497	23.38943	23.23042	0.142602696	+		3	3	5	6.50E-32	3.07E+08	44	19.5	41.331	
STBD1	NaN	NaN	20.53222	N/A	+		1	1	2	9.06E-14	70906000	12	16.8	39.007	

STIP1	23.98224	24.14857	24.11251	0.087498824			9	9	9	2.83E-57	6.44E+08	74	30.9	62.639	
STOM	23.32978	23.55719	23.57092	0.135432837			4	5	6	3.73E-83	7.8E+08	70	57.3	31.73	
STRAP	NaN	NaN	21.44753	N/A			1	0	4	5.78E-13	78375000	12	16.3	38.438	
STT3A	22.49174	22.46124	22.60184	0.073960147			3	3	3	2.44E-24	2.45E+08	32	10.4	80.529	
STX12	23.80469	23.36453	23.01356	0.396402036			3	4	5	1.30E-55	6.07E+08	74	35.5	31.642	
STX7	24.27469	24.21911	24.61341	0.213421674			7	6	7	5.92E-173	1.06E+09	101	46.9	27.4	
STX8	22.52437	22.08002	22.18722	0.231879597			3	3	2	6.42E-28	3E+08	43	45.3	26.906	
SURF4	22.61365	23.179	22.82441	0.285708029	+		4	3	4	4.72E-28	4.23E+08	97	39.8	21.127	
SVIP	23.46326	23.89273	23.81695	0.229231944	+		2	2	2	8.99E-31	6.4E+08	63	32.5	8.4426	
SYNGR2	NaN	22.31285	23.43135	0.790898935			1	2	2	1.06E-10	2.24E+08	27	16.1	24.81	
TAGLN2	26.33929	26.25888	26.54898	0.149774093			9	10	10	3.16E-65	1.47E+09	145	80.4	22.391	
TALDO1	23.6165	23.93822	24.0253	0.215330802			6	8	9	1.04E-38	6.09E+08	81	35	37.54	
TARS	22.37297	22.14451	22.23354	0.115152824			4	3	4	1.24E-26	2.12E+08	45	14.7	83.434	
TCP1	23.47778	23.80075	24.10653	0.314414162			6	8	11	1.25E-68	6.05E+08	99	50.2	60.343	
TECR	21.32273	21.26471	NaN	0.041026335	+		3	4	2	4.07E-10	1.03E+08	10	11.7	36.034	
TFRC	26.94213	26.82651	26.86715	0.058653779			24	21	26	1.90E-297	5.38E+09	446	51.3	84.87	
TKT	23.31879	23.36946	23.7964	0.262347265			4	5	8	3.65E-33	5.03E+08	50	26.2	62.878	
TLDC1	23.34218	NaN	23.61941	0.196031213	+		4	1	5	5.97E-70	7.67E+08	92	36.4	50.993	
TLN1	21.49316	21.6829	21.49707	0.108435345			4	4	4	2.00E-13	76736000	11	3.7	258.08	
TM9SF3	22.32691	21.76749	22.35552	0.331549028			3	2	2	1.12E-13	1.66E+08	27	8.3	67.887	
TMEM106B	24.39054	24.35998	24.20777	0.097900179	+		3	5	7	5.78E-78	1.07E+09	78	36.9	31.127	
TMEM106C	NaN	21.43314	NaN	N/A	+		1	1	1	1.97E-19	1.78E+08	20	14.4	27.875	
TMEM168	NaN	NaN	22.40844	N/A			1	2	3	2.84E-33	2.24E+08	29	15.5	79.754	
TMEM181	21.07477	21.59104	21.43538	0.264828347	+		3	3	3	8.67E-13	1.33E+08	13	6.5	69.324	
TMEM184C	NaN	22.74355	NaN	N/A			1	2	1	7.65E-76	2.24E+08	13	9.6	50.141	
TMEM222	NaN	20.95681	NaN	N/A			1	2	0	1.01E-12	70079000	9	24.6	19.862	
TMEM33	21.82361	22.12304	22.25465	0.220897789			2	3	3	1.04E-23	2.05E+08	41	23.5	27.978	
TMEM50A	21.41998	21.76288	22.05427	0.317493398			2	3	3	5.78E-18	2.8E+08	32	29.3	17.4	
TMEM55A	NaN	20.6118	NaN	N/A			0	2	1	1.33E-07	65789000	10	11.7	28.081	
TMEM55B	22.28057	22.43632	22.08274	0.177206841	+		2	3	3	2.44E-23	3.01E+08	36	26	29.469	
TMEM59	NaN	NaN	20.80813	N/A			0	1	2	5.39E-11	58491000	3	19.8	21.86	
TMEM63B	23.21753	23.05363	23.07685	0.08868788			6	4	6	9.97E-31	3.78E+08	50	11.7	94.957	
TMEM87A	21.51003	NaN	NaN	N/A			2	0	1	1.28E-19	1.19E+08	14	12.8	56.773	
TMEM97	NaN	21.54614	22.02884	0.341320443	+		1	2	2	0.000116	1.76E+08	14	10.2	20.848	
TMPO	19.73978	20.02134	19.97533	0.151039044			2	2	2	0.000105	30332000	6	8.9	26.866	
TMX1	24.26592	24.26213	24.55268	0.166665815			9	5	9	2.61E-59	1.35E+09	138	32.9	31.791	
TMX3	20.60937	21.59846	21.64553	0.585112832			2	2	2	2.42E-18	1.65E+08	30	18.1	51.871	
TMX4	NaN	NaN	21.55267	N/A			1	1	2	3.34E-12	1.01E+08	17	13.5	38.952	
TNFRSF10A	23.05504	23.18477	23.44658	0.199448373			2	5	4	6.36E-32	5.39E+08	62	19.2	50.089	
TNFRSF10D	NaN	18.03569	NaN	N/A	+		0	1	0	0.00835	3899900	2	3.1	41.823	
TNPO1	22.77525	22.04387	22.24977	0.377147351			3	3	4	1.34E-30	1.89E+08	41	15.6	101.31	
TOMM40	22.86622	22.27793	22.76472	0.314470952	+		4	3	4	2.17E-68	3.4E+08	26	16.6	37.893	
TPD52L2	21.51046	21.49941	21.72096	0.124844406			4	2	5	2.31E-15	92947000	19	25.2	22.237	
TPI1	28.22633	27.85436	28.20983	0.210155836			13	15	13	0	5.69E+09	383	85.9	26.669	
TPT1	21.86876	22.22688	22.52449	0.328329986	+		3	3	3	3.17E-17	1.15E+08	14	25.6	19.595	
TRAP1	23.83403	24.05043	23.96402	0.108928913			9	9	7	2.70E-72	4.74E+08	68	24.9	74.267	
TRAPPC3	23.87333	23.78616	23.90848	0.062976601	+		5	3	3	2.21E-32	9.66E+08	75	41	15.005	
TRIM28	22.51744	22.22547	22.0628	0.230364038			2	4	4	3.54E-41	1.88E+08	37	12.6	88.549	
TSN	21.20311	21.10476	21.90161	0.434462291			2	2	2	3.71E-07	53435000	12	13.5	21.01	
TSPAN13	22.06102	22.43909	22.36517	0.200378107			3	3	4	8.27E-20	2.46E+08	28	26	22.147	

TSPAN14	NaN	21.92383	NaN	N/A			1	2	1	1.18E-11	1.52E+08	20	14.6	28.876	
TSPAN3	26.78756	26.36936	26.46535	0.219060343	+		3	4	4	4.69E-52	4.32E+09	77	38.6	25.183	
TSPAN6	24.04951	24.65873	25.07769	0.517015571			5	6	7	2.38E-64	1.77E+09	128	37.1	27.563	
TSPAN7	NaN	NaN	21.37348	N/A	+		1	1	2	1.34E-17	93774000	11	11.2	24.475	
TSPAN9	NaN	20.38585	NaN	N/A			1	2	1	5.39E-13	93473000	21	20.1	26.779	
TTYH3	22.94213	23.10165	23.14934	0.10851801			3	3	3	2.75E-24	4.84E+08	53	17.1	54.139	
TUBA1B;TUBA4A	27.95577	27.32629	27.56326	0.317926604	+		16	17	17	0	9.77E+09	672	59.9	50.151	
TUBA1C;TUBA1B	22.74649	NaN	NaN	N/A	+		2	1	1	0	1.88E+08	28	60.1	49.895	
TUBB	25.63691	24.98989	25.32433	0.32357154			5	5	5	0	1.74E+09	133	58.2	47.766	
TUBB4B;TUBB4A	27.60654	27.03601	27.23818	0.289271003			18	19	19	0	7.07E+09	705	59.6	49.83	
TUFM	22.22279	22.15329	22.23608	0.044461703			3	4	3	2.31E-26	1.91E+08	34	18.6	49.541	
UBA1	25.45955	25.23714	25.50325	0.142706243	+		15	20	19	2.88E-151	1.91E+09	205	37.9	117.85	
UBB;RPS27A;UBC;	25.77037	25.06106	25.10725	0.396858986			4	3	3	4.96E-69	1.89E+09	149	50.5	10.469	
UBE2M	21.92575	NaN	NaN	N/A			3	2	3	3.89E-13	1.29E+08	17	35.5	20.9	
UBE2V1;TMEM18	21.54736	NaN	22.17316	0.442507424			2	0	2	2.16E-10	73551000	16	24.5	16.495	
UBTD2	NaN	NaN	21.33456	N/A	+		1	1	2	7.70E-28	1.23E+08	29	24.4	26.189	
UCHL1	24.39545	24.07153	24.49601	0.22181815			4	6	6	2.42E-73	1.13E+09	117	59.6	24.824	
UQCRC1	NaN	21.45262	21.5582	0.074656334			1	2	3	1.12E-13	85573000	11	15.4	52.645	
UQCRQ	25.40142	25.1517	24.85676	0.272642684	+		5	5	6	2.33E-24	5.59E+08	102	74.4	9.9062	
USMG5	21.56978	NaN	NaN	N/A			2	1	1	1.37E-06	76824000	6	43.1	6.4575	
USP7	21.49453	NaN	21.91619	0.298158645			3	1	3	1.18E-32	3.38E+08	40	13.5	117	
VAMP3;VAMP2	24.06184	23.93202	24.0029	0.065001449			2	2	2	1.86E-157	1.28E+09	43	40	11.309	
VAMP7	23.3688	23.33402	23.87361	0.30199342			3	5	5	1.13E-47	9.21E+08	82	45.5	24.935	
VAT1	NaN	NaN	21.49008	N/A			1	1	2	3.82E-10	63337000	7	19.6	41.92	
VCL	24.83875	24.94481	25.23575	0.205549591			17	18	21	2.16E-84	7.96E+08	139	29.1	116.72	
VCP	22.59316	22.4093	22.562	0.098397777			5	6	5	4.53E-46	2.43E+08	50	16.1	89.321	
VDAC1	NaN	21.80621	21.78584	0.014403765			1	2	2	4.21E-21	1.92E+08	37	23	30.772	
VDAC2	25.74514	25.76451	25.90933	0.089727723			9	10	11	1.60E-124	2.61E+09	161	61.6	31.566	
VDAC3	22.57307	22.5113	22.53709	0.031024768			4	3	3	8.87E-26	2.97E+08	32	22.3	30.658	
XPO1	24.01052	23.86514	24.09144	0.114669875			9	11	11	2.45E-56	7.89E+08	72	22.7	123.38	
XPO5	19.88768	NaN	19.96508	0.054730065			2	1	3	1.23E-08	34216000	8	2.7	136.31	
XRCC5	23.20555	23.06992	23.06676	0.079233985			4	5	7	8.52E-59	5.16E+08	64	25.3	82.704	
XRCC6	24.48136	23.9729	23.87961	0.323866654			6	6	9	1.79E-51	5.25E+08	71	29.9	69.842	
XXYLT1	NaN	19.87917	NaN	N/A	+		0	2	0	2.54E-06	33447000	7	13.9	21.816	
YARS	21.70485	NaN	22.1004	0.279696087	+		5	3	5	1.69E-18	1.02E+08	19	14.4	59.143	
YES1	22.40554	22.27821	22.43469	0.083215225	+		2	4	3	6.69E-79	4.32E+08	52	43.3	60.801	
YWHAB	26.5952	26.64031	27.08355	0.269871059			6	6	5	1.99E-123	1.15E+09	78	48.4	27.85	
YWHAE	27.57744	27.68251	28.11332	0.283961363			13	13	14	4.56E-90	3.48E+09	215	61.2	29.174	
YWHAG	21.55215	21.26717	21.74288	0.239406049			2	3	3	1.59E-39	1.1E+08	25	35.2	28.302	
YWHAH	23.35272	24.22905	24.55128	0.620259692	+		4	4	4	4.43E-29	2.08E+08	27	26.8	28.218	
YWHAQ	24.51473	24.64548	24.81167	0.148822065			6	7	6	1.08E-99	5.14E+08	81	45.7	27.764	
YWHAZ	27.27017	27.03086	27.20686	0.123998274			10	10	10	3.32E-163	2.28E+09	171	53.1	27.745	
ZDHHC13	22.92018	22.6539	22.65668	0.152940629			5	5	6	6.79E-36	4.11E+08	46	14.8	70.86	
ZDHHC18	21.52659	21.75723	21.63338	0.11542511			2	3	3	1.32E-22	1.56E+08	24	20.6	42.03	
ZDHHC3	21.05292	21.0625	21.40302	0.199422356			2	2	2	3.38E-14	1.21E+08	10	9	34.17	
ZDHHC4	20.79773	NaN	20.66275	0.095445273			2	1	2	6.89E-07	80415000	8	11.6	39.786	
ZDHHC5	NaN	NaN	21.1795	N/A			1	1	2	1.03E-23	1.58E+08	29	14.7	71.951	
ZDHHC6	21.91641	21.58344	21.682	0.171041487	+		3	2	2	1.94E-30	2.79E+08	35	18.8	47.205	
ZMPSTE24	20.8472	20.91225	21.18961	0.181844567	+		2	3	2	2.57E-07	82939000	18	5.5	54.812	
ZNRF2	21.92477	22.12398	22.40494	0.241242055	+		3	3	4	2.14E-28	2.54E+08	27	59.5	24.115	

Reagent 6

Gene names	log2 LFQ intensity_1	log2 LFQ intensity_2	log2 LFQ intensity_3	St dev log2 LFQ	MG protein	PTM peptide	Razor + unique peptides_1	Razor + unique peptides_2	Razor + unique peptides_3	PEP	Intensity	MS/MS Count	Sequence coverage [%]	Mol. weight [kDa]
AARS	21.24731	21.00161	21.16116	0.124663891			3	3	3	3.06E-15	77577000	13	5.9	106.81
AASDHPPT	21.07738	20.90095	21.11528	0.114383349			2	2	2	5.46E-06	19014000	7	7.4	35.776
ABHD17A	22.52621	22.91563	22.77394	0.197101558			5	4	4	1.06E-22	278670000	24	33.5	33.989
ABHD17B	22.52979	23.28616	22.73194	0.391603543			6	6	4	7.99E-26	328720000	22	34	32.214
ABL2	22.7265	22.36842	22.81357	0.235924068	+		6	7	6	1.43E-35	296370000	46	12.6	115.82
ACAA2	20.8762	NaN	20.72092	0.109799541	+		4	2	2	1.25E-15	53733000	8	14.6	41.924
ACACA	25.37478	25.58222	26.17236	0.413809624			42	39	45	5.68E-188	2448300000	314	31	265.55
ACAD9	NaN	NaN	20.0868	N/A			2	1	2	2.10E-08	36352000	6	6.1	68.76
ACAT1	21.37231	21.20943	21.03825	0.167047184			3	3	3	3.69E-18	87102000	17	21.8	45.199
ACOT7	20.69914	20.80333	20.40634	0.205826973			3	3	2	4.67E-09	56625000	11	14.2	27.041
ACOT9	23.20105	22.97376	23.16142	0.121413588			8	6	9	4.59E-33	351240000	54	31.3	46.354
ACTA1;ACTC1;ACTG2	24.59463	NaN	24.55824	0.025731616			1	1	1	7.40E-92	960290000	26	28.9	42.051
ACTG1;ACTB;ACTA1	28.40832	28.4603	28.46346	0.030963219			18	19	19	0	12810000000	601	67.5	41.792
ACTN4;ACTN1	20.84957	20.76782	20.839	0.044462306	+		4	3	4	5.49E-17	75173000	11	9.9	104.85
ADSL	20.89075	20.38459	20.44506	0.276433881			2	3	2	3.16E-07	41640000	9	6.4	54.889
AGPAT1	21.86967	21.84196	21.38179	0.274028942			4	4	4	4.89E-11	112140000	30	35.1	18.756
AHCY	24.41886	24.55848	24.68571	0.133472931			11	11	12	3.52E-54	900180000	113	34.3	47.716
AIFM2	24.22288	24.2103	24.39506	0.103231509	+		9	10	9	1.32E-58	766490000	64	44.5	40.526
AKAP12	27.58398	26.97465	27.16921	0.311226283	+	+	31	30	29	0	4841000000	455	37.4	191.48
AKR1B1	21.44607	21.06454	21.48281	0.231612018			4	4	3	3.17E-08	93513000	7	11.1	35.853
ALDH18A1	22.4226	22.41803	22.46104	0.023623359			7	8	8	5.75E-27	224330000	48	14.9	87.088
ALDH7A1	22.71509	22.33366	22.62941	0.200124116	+		8	8	7	1.96E-21	210600000	19	21.5	55.366
ALDH9A1	21.37756	21.36672	21.49409	0.070616173			4	3	4	1.39E-15	91824000	15	13.2	53.801
ALDOA	26.55935	26.53484	26.7599	0.1234727			18	18	17	5.06E-187	3005900000	221	68.4	39.42
ALDOC	22.56918	22.63194	22.66117	0.04700243			1	1	1	5.87E-18	218100000	13	8.2	39.455
ALG6	21.13515	21.07907	21.18404	0.052526024			2	2	2	2.23E-07	65478000	7	5.5	58.18
ANO6	21.03221	21.31678	21.42157	0.201478799			5	6	6	6.48E-20	116640000	21	11.8	103.96
ANP32A	22.18047	22.38516	22.47843	0.152412516			5	4	4	3.14E-20	166760000	16	29.4	19.997
ANP32B	NaN	NaN	21.58843	N/A			1	1	2	5.50E-16	85008000	10	17.9	22.276
ANXA1	23.97404	24.1779	24.01945	0.107025986			10	10	10	5.45E-105	609870000	89	54.3	38.714
ANXA11	21.48045	21.60291	21.60458	0.071189299			4	4	4	2.02E-11	109060000	26	8.5	51.242
ANXA2;ANXA2P2	26.01585	26.04078	25.81252	0.125211302	+		15	17	15	6.34E-165	2646200000	241	64.3	38.604
ANXA5	27.52664	27.7497	27.58484	0.115702077			20	21	20	4.45E-256	6819100000	647	76.6	35.936
ANXA6	24.13727	24.29691	24.07529	0.114340044			16	16	14	2.41E-58	657030000	106	42	75.276
ANXA7	20.90492	21.4805	21.23105	0.288640034			2	2	2	8.89E-12	79585000	13	18.5	37.805
AP1B1	21.05722	21.37618	21.79543	0.370238673			4	4	4	2.23E-21	148030000	32	10.9	101.35
API5	22.5637	22.58463	22.61128	0.023847235			5	4	4	4.29E-71	220970000	36	21.8	50.309
APMAP	22.37472	22.8341	22.3824	0.263034173			6	6	4	1.73E-21	241240000	24	18.3	46.48
APRT	23.17446	23.21459	22.9149	0.162683694			6	6	6	6.05E-23	318610000	45	49.4	19.608
ARCN1	20.09944	20.36688	20.6082	0.254491727			3	3	2	6.23E-10	44299000	10	8	57.21
ARF1	30.80093	31.03525	30.53157	0.252043057	+	+	14	14	12	0	62286000000	761	79.6	20.697
ARF3	24.62171	24.88911	20.85163	2.257810544	+	+	1	1	1	0	196600000	22	64.6	20.601
ARF4	27.58778	27.60215	27.38022	0.1241911	+	+	8	8	7	0	7011100000	255	86.7	20.511
ARF5	26.18601	26.14322	25.95525	0.122755776	+	+	6	6	6	0	2770000000	142	71.1	20.529
ARF6	25.58342	26.01395	25.48815	0.280148203	+		5	6	6	2.11E-78	2141900000	117	60	20.082
ARHGDIA	21.76272	22.00782	21.9572	0.129395302			2	2	2	3.77E-12	92974000	10	23.3	21.517

ARL1	26.7128	26.8447	26.42349	0.215451367	+		10	10	10	0	4384500000	238	80.5	18.565
ARL13B	20.39759	20.43145	20.50164	0.053071553			2	2	2	2.82E-06	42032000	11	8.3	37.086
ARL4C;ARL7	21.37756	NaN	NaN	N/A	+		2	1	1	1.02E-07	117080000	8	16.1	21.487
ARL5B	22.69564	22.46581	22.27611	0.210084642	+		3	3	3	2.83E-21	238040000	23	21.2	20.374
ATAD3B;ATAD3A;AT	NaN	20.64992	NaN	N/A			1	2	2	8.46E-09	46550000	10	8.1	67.608
ATP11B	21.75581	21.79432	21.99445	0.12811719			4	4	4	4.17E-22	109950000	16	8.1	134.19
ATP1A1	24.62461	24.4295	24.55081	0.098514354	+		16	16	15	3.17E-92	884810000	116	24.8	113
ATP2A2	23.83586	23.29712	23.61684	0.27093401	+		11	10	13	1.20E-60	456360000	66	21	114.76
ATP5A1	23.46051	23.28136	23.34516	0.090802703			13	13	11	7.15E-77	707660000	92	40.7	59.75
ATP5B	NaN	21.93427	21.75467	0.126996378			2	5	5	1.30E-18	167800000	15	24.6	38.138
ATP5H	22.39804	22.54995	22.4382	0.078716301			6	5	3	2.04E-20	206050000	18	74.5	15.773
ATP5O	23.00654	22.79822	23.17295	0.187755197			6	5	5	2.48E-24	362490000	48	34.7	23.277
ATP6V0A2	21.08492	NaN	NaN	N/A	+		2	1	1	3.84E-20	80622000	8	9.2	98.081
ATP6V1A	21.23614	21.12931	21.35794	0.114396653			3	3	3	1.00E-20	61686000	12	8.9	64.735
ATP9A	23.13366	22.95761	23.29544	0.168965223			8	8	9	8.96E-41	318510000	59	12.7	118.58
ATXN10	20.03899	21.12187	NaN	0.765711791			2	2	1	5.06E-09	37702000	7	13.6	46.286
AUP1	21.50882	21.42941	21.54099	0.057432502			2	2	2	4.57E-10	102350000	8	9.4	41.385
B3GNT1	22.37151	23.12693	22.35181	0.441938623			6	6	5	8.47E-30	239460000	42	33.3	47.119
B4GALT1	21.8297	NaN	NaN	N/A			2	1	1	1.14E-05	34452000	10	4.9	42.537
BAG5	22.28368	22.29846	22.48254	0.110791993			5	5	4	3.83E-39	242750000	31	33.3	51.199
BAG6	21.21727	21.20479	NaN	0.008824693			4	3	2	4.22E-10	44693000	9	6.4	118.69
BANF1	NaN	21.59423	NaN	N/A			1	2	1	4.19E-07	28886000	2	40.4	10.058
BASP1	27.40714	26.74906	26.77493	0.37269917	+		7	7	7	2.37E-200	1931800000	95	57.3	22.693
BAX	21.89767	22.1684	22.00192	0.136551813			3	3	3	2.50E-44	169410000	36	36	18.129
BCAM	22.24629	22.52714	22.1622	0.191106015			4	5	4	1.08E-32	189940000	33	21	67.404
BCAP31	23.35662	22.87558	22.98111	0.252831902	+		3	3	3	5.54E-18	215440000	35	19.5	27.991
BET1;DKFZp781C042	21.18253	21.24795	NaN	0.046258926			2	2	2	2.61E-16	36720000	9	24.6	13.289
BET1L	20.97021	20.84889	21.06802	0.109774994			2	2	2	6.35E-10	92362000	11	48.3	6.4651
BZW1	20.6321	20.6808	20.8155	0.095001175			3	2	2	4.91E-10	61522000	18	10.2	40.538
C11orf48	21.09029	21.14345	21.47625	0.20918371	+		2	2	2	6.25E-11	261450000	20	21.2	11.6
C12orf23	23.28376	23.33006	23.43377	0.07681412			5	5	5	2.14E-28	418710000	61	62.9	11.748
C1QBP	21.73517	21.79773	21.83055	0.048456596			3	3	3	2.97E-23	114340000	13	26.2	31.362
C9orf123	23.8099	23.72244	24.51551	0.434836128	+		3	3	3	3.61E-57	618140000	36	50.9	11.829
CALR	22.13094	22.03144	22.02022	0.060944041			4	3	3	6.29E-16	81862000	10	12.9	48.141
CAND1	24.6142	24.55941	24.56326	0.030582267			23	22	23	2.82E-106	888800000	132	31.7	136.37
CANX	28.83065	29.05961	28.57084	0.244547211			21	22	21	0	18403000000	767	44.9	67.567
CAP1	22.09181	22.16791	22.04727	0.061004141			3	3	3	9.88E-15	110850000	18	13.7	51.83
CAPNS1;CAPNS2	20.13609	NaN	20.98572	0.600779134	+		2	1	2	2.54E-10	78085000	15	37	11.399
CAPZB	21.99528	22.30405	22.07398	0.160449789			5	5	4	2.22E-13	162060000	25	25.4	29.295
CAV1	24.28001	24.43841	24.6133	0.166712975			6	6	5	1.42E-67	570630000	75	47.2	20.471
CAV2	21.69613	21.57385	21.60173	0.064084648	+		2	2	2	8.33E-08	55929000	11	18.8	16.828
CBR1	20.39749	20.13069	20.11452	0.158910735			2	2	1	1.04E-09	40273000	8	23.1	18.762
CCNY	24.06956	24.14639	23.77646	0.195217257	+		9	9	7	1.95E-44	520030000	52	47.5	39.336
CCRN4L	NaN	20.36549	20.1614	0.144313423			1	2	2	1.01E-06	32196000	4	6	48.195
CCT2	24.2844	24.20516	24.09257	0.096396952			12	10	10	4.36E-88	661110000	84	42.1	57.488
CCT3	24.10677	23.84986	23.80025	0.164528828			13	10	10	7.39E-80	600610000	89	40	55.674
CCT4	24.61891	24.32306	24.4412	0.148921191			17	17	18	2.70E-75	768400000	105	55.8	57.924
CCT5	23.16765	22.79893	23.04818	0.188128095			9	9	9	5.33E-38	271770000	48	22.6	59.67
CCT6A	23.55824	23.70451	23.52492	0.095531586			12	12	10	5.77E-76	440960000	63	29.6	58.024
CCT7	23.81225	24.01801	23.46126	0.281514274			10	10	9	4.56E-49	471770000	57	33.1	54.804

CCT8	25.04339	24.9266	24.91957	0.069547007			20	22	21	6.05E-87	1160500000	182	46.5	59.62
CD151	26.83461	26.62115	27.02115	0.200150919	+		5	5	5	2.39E-50	3563900000	181	15.1	28.067
CD276	25.71675	25.61378	25.78743	0.087323924			8	8	8	2.83E-258	1834300000	130	37	57.165
CD44	24.54822	24.33286	24.46214	0.108399734			6	6	6	4.97E-34	762720000	94	33.5	22.683
CD47	21.21904	20.63848	21.2366	0.340368869			2	2	2	7.11E-07	55427000	10	9.6	31.742
CD55	19.51497	19.5141	19.40433	0.063628373			2	2	2	0.00040181	19435000	4	5.2	35.695
CD59	21.67103	21.30991	21.49219	0.180562731	+		2	2	2	2.62E-06	72878000	5	18.5	11.985
CD63	27.21651	27.2943	27.38599	0.084834948			7	7	7	4.99E-212	5647000000	157	33.5	23.43
CD81	28.43416	28.56129	28.56435	0.074297641	+		4	4	4	0	12126000000	235	50.3	17.963
CD9	26.53069	26.55172	26.30788	0.13512001			5	5	5	4.90E-220	2909400000	196	42.8	17.764
CDC37	22.19412	21.97194	22.06319	0.111678989			3	3	3	5.76E-15	153190000	26	15.3	44.468
CDC42	22.06326	21.74641	21.77535	0.175177821			2	3	2	6.27E-09	67238000	14	25.7	21.258
CDCA3	25.05122	24.95775	25.01844	0.047424405	+		11	11	11	3.67E-87	961420000	98	52.2	28.998
CDK1	22.59298	22.93432	23.1604	0.2856544	+		4	2	3	2.39E-13	391480000	33	26.5	21.738
CECR5	21.73215	NaN	NaN	N/A			4	2	1	7.30E-13	82416000	13	13.5	43.588
CERS2	21.1838	20.35864	20.38953	0.467744241			3	3	3	2.17E-11	77216000	9	13.8	36.373
CFL1	24.81113	25.15252	24.53919	0.307319645			8	7	7	1.87E-62	872620000	67	69.1	16.811
CHCHD3	27.70859	27.51187	27.19442	0.259436585	+		11	12	13	1.22E-222	5776900000	281	46.7	26.152
CHCHD6	25.01742	24.54745	25.02703	0.274153585	+		8	7	7	3.85E-134	799910000	72	44.7	26.457
CHIC2	21.35832	21.17682	21.72812	0.280958473			2	2	2	1.32E-07	93967000	9	26.3	15.99
CHMP6	25.39146	25.16932	25.19141	0.122375209	+	+	8	7	6	5.52E-146	1432900000	94	48.3	23.485
CHP1	25.28849	25.39548	25.37126	0.056101606	+		9	9	9	5.54E-76	1331100000	131	67.2	22.456
CKAP4	27.41764	27.45725	27.04989	0.224629764			27	28	26	0	5985400000	480	57.1	66.022
CKAP5	20.36282	NaN	20.40354	0.028793388	+		3	1	3	6.97E-11	43521000	11	2.9	218.52
CKB	24.11862	24.35481	24.2012	0.119861868			10	10	7	7.38E-86	636120000	80	52	42.644
CLCC1	20.83399	20.83584	20.824	0.006369304			5	3	4	1.32E-15	62104000	12	13.2	56.266
CLDND1	21.27242	21.18948	21.36666	0.088650036	+		3	3	3	1.34E-11	118680000	14	33.3	16.049
CLIC1	24.03808	23.86098	24.10077	0.124360826			8	7	7	4.85E-41	575700000	69	53.1	26.922
CLN3	22.4967	22.11772	22.1328	0.214583495	+		3	4	4	2.72E-24	131010000	29	21.5	34.568
CLTA	21.45197	21.56454	NaN	0.07959901			2	2	1	5.26E-06	74539000	19	7.8	23.662
CLTC	23.42316	23.224	23.58909	0.182796872			15	13	15	1.29E-71	423020000	98	15.8	187.89
CNDP2	19.50951	NaN	NaN	N/A			2	2	2	0.00047512	13386000	2	3.8	52.878
CNN3	21.14221	20.64271	21.2962	0.341629026			3	3	2	3.75E-11	72237000	18	16.3	31.38
COPB1	21.01107	21.13965	20.96367	0.09105725			2	2	2	3.85E-10	75607000	16	4.9	107.14
COPB2	20.98919	21.20413	20.75026	0.227040644			3	4	4	4.85E-14	61662000	10	6.8	99.045
COPG1	21.3175	NaN	21.11769	0.141287006			2	1	2	8.03E-09	72398000	12	4.9	97.717
COPS3	21.36517	NaN	NaN	N/A			2	2	2	9.56E-08	55200000	9	9	39.036
COTL1	20.2885	20.26282	20.3673	0.054444119			2	2	2	0.00037477	34272000	4	12	15.945
COX4I1	NaN	20.72359	NaN	N/A			2	2	0	6.14E-11	21778000	2	18.9	19.576
CPD	24.88879	24.87408	24.73257	0.086261379			21	22	20	7.36E-130	989290000	146	25.1	152.93
CPNE1	20.14445	NaN	NaN	N/A			2	2	1	6.58E-07	20100000	6	6.2	58.634
CPNE3	21.71888	22.18135	22.16962	0.263686253			4	4	5	9.04E-14	127430000	22	11.9	60.13
CS	24.50221	24.4224	24.52964	0.055711248			7	7	7	3.26E-35	656200000	63	24.5	50.431
CSE1L	25.14005	25.15484	25.05238	0.055381743			18	21	18	5.92E-151	1439800000	186	35.7	107.78
CTNNA1	19.97421	NaN	NaN	N/A			2	1	1	1.59E-07	30602000	5	5.4	88.676
CUL1	NaN	NaN	20.21312	N/A			1	2	2	0.00042201	26865000	2	2.3	89.677
CUL4B	19.86455	20.1566	NaN	0.206510535			2	3	3	1.36E-06	21928000	4	4.2	84.016
CXADR	24.69158	24.28799	24.32121	0.224039577			8	8	8	6.57E-93	701510000	58	40.5	40.029
CYB5D2	19.7899	19.65803	19.71487	0.066143762			2	2	2	1.90E-12	31122000	10	24.2	28.689
CYB5R3	27.93703	28.09411	27.86207	0.118417123	+		21	19	19	0	12158000000	574	85.7	31.76

CYSTM1	23.96429	23.31174	NaN	0.46142253			2	2	1	5.15E-08	413970000	33	11.3	10.631
DAD1	21.40432	22.42742	22.47776	0.605742129			2	2	2	4.48E-11	163690000	21	35.3	9.5541
DAGLB	24.90394	24.91031	25.06513	0.091279813			14	14	16	1.15E-81	1083400000	139	24.7	73.731
DCAF11	20.91401	21.36981	20.59432	0.389730693	+		3	4	4	2.45E-13	86374000	12	12.3	58.846
DCUN1D3	20.5793	NaN	NaN	N/A	+		2	1	1	3.21E-09	47330000	8	13.8	34.291
DDB1	20.94728	21.07267	NaN	0.088664119			3	3	1	2.28E-15	76194000	19	5.6	121.71
DDX17	21.54745	21.6321	21.62154	0.046127476			5	5	5	2.65E-21	99665000	25	14	72.371
DDX39B;DDX39A	24.00376	24.22052	23.96367	0.138181058	+		10	10	10	6.14E-49	533340000	82	29.9	48.991
DDX46	23.33906	23.48038	23.85728	0.267885834	+		15	14	15	2.70E-41	462040000	87	17.4	117.36
DEGS1	23.64592	23.93984	24.04585	0.207191977	+		3	3	3	2.35E-38	633410000	55	15.8	37.866
DESI2	NaN	21.07483	NaN	N/A	+		1	2	0	1.02E-06	50280000	6	12.4	21.444
DHCR7	22.45025	21.72709	22.30742	0.383002111	+		5	4	5	3.17E-16	139650000	33	12	54.489
DHX15	21.57076	21.45628	21.51749	0.057285873			5	5	6	5.54E-17	87879000	18	8.3	90.932
DHX9	21.63626	21.74944	22.23119	0.315920475	+		5	4	5	6.18E-22	103660000	21	8.3	140.96
DIRC2	22.69496	22.89624	22.87819	0.111364771	+		2	2	2	6.06E-22	222070000	16	10.5	44.145
DLAT	20.3868	20.72692	20.57985	0.170577207			2	2	2	7.86E-06	32341000	5	4.8	57.586
DNAJC5	26.33518	26.20049	26.63367	0.221691448			8	8	8	9.45E-289	3383700000	275	54.5	22.149
DNAJC7	21.03053	NaN	NaN	N/A			2	1	1	7.58E-15	47893000	17	20.6	28.704
DNM1L	21.01141	21.32378	21.39126	0.202655122			3	2	3	9.62E-12	79167000	13	6.6	78.099
DRG1	20.32937	NaN	NaN	N/A			3	2	3	5.29E-07	34944000	4	12.3	40.542
DSG2	21.47353	20.5695	21.35526	0.491371687			3	2	3	7.38E-17	87772000	17	6.6	122.29
DSTN	NaN	20.81386	21.07045	0.181436529			2	2	2	2.06E-07	32626000	4	16.2	16.62
DTYMK	20.37316	20.52726	20.50261	0.082776542			2	2	2	0.0010263	29337000	3	9.4	19.368
DUT	21.22858	NaN	NaN	N/A			3	2	1	5.14E-13	53319000	14	36	17.748
DYM	23.35797	23.62734	23.5708	0.142040826	+		8	9	9	3.98E-34	399220000	68	18.1	75.935
EBP	22.55086	22.56284	23.09504	0.310781872			2	2	2	3.35E-07	146250000	21	9.6	26.352
ECE1	20.96691	20.88189	20.96944	0.049832727			2	2	2	1.73E-07	62583000	12	3.3	85.561
ECHS1	23.32663	22.96927	23.22286	0.183839727			4	4	4	3.25E-21	296080000	41	24.8	31.387
EEF1A1P5;EEF1A1	27.76528	27.96939	27.83237	0.104032399	+		15	15	12	2.69E-171	7650700000	406	42.6	50.184
EEF1B2	22.81319	22.88435	22.91401	0.051813984	+		3	3	3	1.50E-38	274690000	28	24.4	24.763
EEF1D	23.02866	22.81147	22.82429	0.12186259			6	6	5	1.30E-24	167220000	29	31.8	28.821
EEF1G	25.33272	25.69548	25.28606	0.224126716			19	19	18	1.22E-122	1467700000	158	47.6	50.118
EEF2	27.48408	26.96708	27.27719	0.260211672			39	37	34	2.09E-242	5105400000	440	54.8	95.337
EEPD1	21.33876	21.74608	21.15802	0.301217279	+		4	4	3	2.83E-15	64368000	9	10.5	62.402
EFR3A	21.69876	22.0111	21.66084	0.19221354			5	5	3	1.30E-41	205560000	38	21.3	92.923
EIF2B1	21.33293	20.51046	21.38137	0.489436336			3	2	2	2.31E-13	68937000	17	17.4	33.712
EIF2S1	20.48827	20.73463	20.84069	0.180804604			3	3	3	2.19E-10	52197000	13	14.9	36.112
EIF2S2	20.85148	20.32136	20.72084	0.276187827			3	3	3	3.07E-09	56630000	11	10.8	38.388
EIF2S3;EIF2S3L	21.36592	21.12351	21.09706	0.14818227			4	3	3	1.56E-16	110650000	18	14	51.109
EIF3A	21.33533	21.6348	21.76656	0.220983518			5	5	4	3.50E-24	103400000	19	7.2	166.57
EIF3B	19.67965	19.65083	NaN	0.020378817			2	2	1	1.04E-07	27529000	6	4.5	92.48
EIF3C;EIF3CL	22.20275	21.96677	22.17862	0.129839157			7	6	4	1.40E-25	144150000	33	11.2	104.1
EIF3E	22.47117	22.57288	22.64042	0.085197944			7	7	7	1.05E-25	231570000	46	21.1	52.22
EIF3F	22.0002	22.17469	21.82151	0.176594162			6	7	5	4.00E-26	146360000	26	25.2	37.563
EIF3H;EIF3S3	21.42992	20.78245	21.23987	0.332808619			3	3	3	7.43E-10	72084000	13	10.8	39.93
EIF3L	21.6383	21.29238	21.69638	0.218422348			7	4	5	3.23E-18	111060000	24	14.2	66.726
EIF3M	21.55483	21.86106	21.53673	0.182251828			3	2	2	4.22E-15	125960000	22	15.5	42.502
EIF4A1	25.2989	25.38584	25.04114	0.179265572			17	17	17	4.71E-110	1204100000	132	53.2	46.153
EIF4A3	21.59778	20.56728	20.76409	0.547068466			3	3	3	1.00E-14	45677000	9	16.3	46.871
EIF4G1	22.36896	21.96	22.5679	0.309937578			7	9	6	1.14E-32	157570000	31	11.1	154.8

EIF4G2	19.55766	20.0155	19.82645	0.23007442			3	3	3	2.33E-08	29369000	7	4.7	98.149
EIF5A	23.5438	23.70155	23.22233	0.244226613			4	5	5	1.03E-21	253130000	32	54.5	16.832
ENDOD1	20.17792	NaN	20.32575	0.104531595	+		3	2	3	5.36E-10	40164000	7	11.8	55.016
ENO1	26.6047	26.85769	26.50609	0.181360797			17	16	15	2.03E-225	3376800000	290	52.1	47.168
ERGIC2	23.77896	23.9785	23.57565	0.20142794			8	8	7	3.60E-37	508100000	71	28.4	42.548
ERGIC3	23.29025	23.41015	23.43237	0.076450237			3	3	3	2.37E-42	393460000	51	26.6	43.222
ESD	21.71347	21.35143	21.41483	0.193338378			3	4	3	8.50E-13	96215000	14	25.9	31.462
ETF1	19.69057	20.43614	20.22376	0.384116186			3	3	3	1.62E-07	39365000	6	10.4	45.462
EXTL2	21.37039	NaN	NaN	N/A	+		2	1	0	3.14E-08	61811000	4	12	35.967
FABP5	19.05155	NaN	19.24109	0.134025019	+		2	1	2	8.64E-06	21856000	5	14.8	15.164
FAM129A	23.31824	22.86316	23.04713	0.228926262	+	+	7	6	7	1.16E-44	302130000	50	14.7	103.13
FAM129B	26.00654	25.91582	25.96038	0.045362351	+	+	22	21	21	1.08E-262	2216800000	223	42	84.137
FAM219B	21.1943	21.30864	21.15443	0.080045796			2	2	2	4.66E-07	49572000	8	28.3	11.907
FAM49B	26.56576	26.88983	26.37815	0.258854946	+		17	18	16	0	4624100000	348	73.1	36.748
FAM69B	23.16106	23.0377	22.77686	0.196156755			8	6	7	3.25E-38	255700000	36	29	48.582
FAM84B	23.15195	22.69066	23.08915	0.250175466	+		6	6	5	2.50E-34	283030000	42	31.6	34.474
FAS	21.18302	NaN	20.12502	0.748118974			2	1	2	4.42E-10	35610000	9	9.9	35.386
FASN	20.64421	20.40499	20.64913	0.139555696			4	3	4	5.08E-18	54301000	13	4.3	273.42
FBXO17;SARS2	22.33617	22.32696	22.226	0.061121702	+		5	4	4	1.82E-23	196460000	26	34.2	31.479
FDPS	21.30864	21.44874	21.02025	0.218479779			2	2	2	3.96E-08	92928000	14	10.5	40.532
FEN1	21.36217	21.15752	21.03059	0.167301197	+		2	2	2	1.82E-17	62812000	20	17.9	42.592
FKBP1A;FKBP12-Exip	20.26888	NaN	20.00881	0.183897261	+		2	1	2	3.02E-07	28265000	11	25	11.951
FKBP4	23.46438	23.5745	23.74386	0.140782512			11	10	9	4.29E-36	395380000	59	33.6	51.804
FLNA	22.72752	22.49617	22.23611	0.245844739			7	11	9	1.97E-43	177120000	25	7.9	276.55
FLOT1	24.12849	24.06907	24.18926	0.060096264	+		11	13	12	5.10E-92	655540000	75	49.6	42.08
FLOT2	25.97978	25.92379	25.7463	0.121895099	+		20	21	18	3.25E-136	2154400000	218	51.2	47.064
FMNL2	20.645	20.34278	20.60023	0.163106194	+		3	2	3	3.03E-13	43226000	7	5.7	123.32
FMNL3	21.10961	21.65228	21.31047	0.274368835	+		4	4	3	1.05E-25	91132000	21	9.8	117.21
FRS2;FRS3	20.92426	20.74222	20.70421	0.117618964	+		3	3	3	2.65E-11	56786000	10	10.4	57.028
FSCN1	24.25429	24.11807	24.12242	0.077421474			12	10	9	2.27E-45	460430000	88	25.6	52.262
FUBP1	22.5863	21.97782	22.45224	0.319711936			8	7	8	7.03E-21	150470000	20	17.2	67.56
FUS	21.11172	NaN	20.90691	0.14482254			2	1	2	5.66E-08	52552000	8	7	53.354
FYN	23.87202	23.66308	23.6279	0.131964724	+		12	11	10	1.22E-97	560360000	63	44.5	60.761
G6PD	20.49736	NaN	NaN	N/A	+		2	1	1	2.32E-07	39799000	9	6	59.256
GALNT1	21.58623	21.44703	21.69638	0.124956716	+		5	5	5	4.33E-15	115110000	17	12	57.379
GALNT2	21.01788	NaN	20.78037	0.167944932			4	3	3	7.30E-12	58308000	13	10.7	60.793
GANAB	22.8839	22.58779	23.10612	0.260041295			7	5	7	9.90E-45	225350000	45	18.9	96.215
GAPDH	26.18029	26.49989	26.14598	0.195180952	+		13	13	11	6.55E-212	2426000000	195	63.6	36.053
GDI1	19.78655	20.2498	NaN	0.327567216			2	2	1	1.45E-19	28235000	6	19.7	50.582
GDI2	23.36145	23.1212	23.22513	0.120488346			8	6	5	1.48E-35	308750000	38	34.8	50.663
GHITM	22.85369	22.37806	22.83642	0.269757928			3	3	3	5.38E-21	212760000	25	16	35.282
GLG1	22.61794	22.17919	22.50026	0.227096195			7	8	7	1.40E-34	187470000	25	11.2	134.55
GLIPR2	21.33467	20.67994	20.94306	0.329459633	+		2	2	2	3.20E-10	66801000	12	20.3	14.213
GLO1	23.44985	23.67152	23.40989	0.140940086			6	6	6	9.91E-26	411620000	45	50.3	19.043
GLRX3	21.4707	20.71825	21.31612	0.397392442			3	3	3	6.61E-07	49239000	6	9.3	37.432
GLUD1;GLUD2	21.28557	21.8182	21.1004	0.372652538			3	3	3	5.46E-18	89335000	17	14	61.397
GMPS	20.66544	20.52363	20.32169	0.17274929			2	2	2	4.76E-06	46202000	11	3.7	65.928
GNA11	24.82673	24.6002	24.80809	0.125752099			11	11	10	5.04E-89	1080700000	90	50.7	42.123
GNA13	23.27895	23.05122	22.80597	0.236544075			9	6	8	6.67E-45	350970000	67	34.7	44.049
GNAI1	24.71597	24.25227	24.53777	0.233909947	+	+	7	8	8	3.76E-210	718810000	72	45.2	40.361

GNAI2	25.87188	25.97598	25.63666	0.17383101	+		12	11	10	0	2345300000	176	62	40.45
GNAI3	27.62554	27.3413	27.04822	0.28867128	+	+	17	17	16	2.06E-231	6898300000	372	61	40.532
GNAQ	21.57667	21.44455	NaN	0.093422948	+		2	3	1	3.81E-40	90141000	10	37.9	42.142
GNAZ	20.58802	20.51778	20.5682	0.03621387	+		2	2	2	1.87E-14	39581000	7	16.3	40.923
GNB1	22.55058	22.53995	23.08088	0.309283137			3	3	4	6.57E-13	223170000	25	12.9	37.377
GNB2L1	24.11569	23.95305	23.97079	0.089221155			10	10	9	3.02E-46	582190000	60	40.1	35.076
GOLGA7	23.32553	23.5451	23.59844	0.144646701			5	5	5	7.22E-29	334760000	66	40.1	15.824
GOLIM4	24.40613	24.86505	24.8532	0.261603891	+		9	9	9	9.67E-193	1014800000	107	17.7	81.879
GORASP1	21.99683	22.00251	21.99773	0.003052889	+		3	3	3	3.86E-11	168090000	19	16.3	23.906
GORASP2	26.56466	26.68966	26.28498	0.207208351	+	+	10	8	10	9.70E-171	2913400000	148	36.9	47.145
GOT1	20.68037	20.66899	20.29367	0.220049804			4	4	3	3.10E-14	109400000	14	15.7	46.247
GOT2	22.94737	23.08417	23.21762	0.13512846			6	4	4	1.98E-40	313110000	46	29.5	47.517
GPI	24.42053	24.26578	24.11902	0.150772644			9	8	9	1.84E-50	716340000	79	24.9	63.146
GPRC5A	24.18752	23.99886	24.06299	0.095927901			4	4	3	2.15E-82	537640000	33	14.8	40.251
GPRC5C	20.67926	20.88338	20.63962	0.130802167	+		3	3	3	1.10E-08	54549000	8	10	48.193
GRPEL1	20.82594	NaN	20.84483	0.013357247			2	1	2	2.01E-05	49739000	4	9.2	24.279
GSPT1;GSPT2	21.64126	21.7882	21.78437	0.083752119			3	3	3	9.09E-15	120980000	12	10.8	68.6
GSTO1	23.48161	23.42458	23.35192	0.065001785			5	5	5	2.11E-18	331180000	41	30.3	27.566
GSTP1	26.04245	26.33444	26.02542	0.17370547			6	6	6	1.76E-166	2396600000	145	57.6	23.356
HADHA	22.55696	22.64564	22.51198	0.068010218			9	6	6	9.30E-34	224200000	39	19.3	82.999
HADHB	24.25306	24.35897	24.41164	0.080765786			12	12	10	1.54E-60	876650000	87	40.3	48.879
HARS;HARS2	21.13846	20.8524	20.98001	0.143306803			4	4	4	6.83E-13	68602000	14	11.1	49.623
HCCS	25.41361	25.47513	25.72213	0.163288242	+		13	12	12	5.27E-112	1409600000	172	60.1	30.601
HGSNAT	NaN	20.93316	21.24406	0.219839498	+		1	2	2	6.30E-07	44056000	6	4.1	70.495
HIST1H4A	19.14549	20.46004	19.75375	0.657883918			2	2	3	1.63E-22	241560000	21	50.5	11.367
HK1	19.461	20.07025	20.1121	0.364432937	+		2	2	2	1.56E-07	25223000	5	4.4	101.08
HLA-B;HLA-C	22.61603	22.71295	21.97351	0.401869994			5	6	4	2.78E-39	259800000	51	26.8	40.46
HLA-C	NaN	19.81257	19.8576	0.031841018			2	2	2	8.95E-30	30057000	10	26.5	40.648
HM13	23.54227	23.01173	23.33129	0.267115408			3	4	4	1.24E-32	383680000	48	22.7	36.813
HMGB1;HMGB1P1	22.71259	22.41227	22.54272	0.150590572	+		7	6	6	7.82E-22	173180000	33	30.2	24.893
HMGB2	20.95497	20.78668	20.85064	0.084948175	+		2	2	2	5.90E-15	36018000	11	21.5	24.033
HMOX2	21.75858	21.53763	21.56611	0.120190622			3	3	3	2.07E-19	97839000	13	34.8	32.837
HNRNPA1;HNRNPA1	22.82106	22.10124	22.64544	0.375308064			4	8	4	6.42E-25	156960000	24	38.6	29.386
HNRNPA2B1	21.31307	21.27725	21.61814	0.18733071			4	4	3	1.64E-11	85576000	13	12.6	36.006
HNRNPAB	NaN	20.22764	20.82361	0.421414428			1	2	2	1.05E-07	27971000	6	12.5	30.302
HNRNPD	23.05872	22.1742	22.26161	0.487408195			5	5	5	4.76E-23	224730000	25	46.8	12.553
HNRNPF	22.51263	21.78469	21.73616	0.434963114			5	5	6	3.82E-16	120010000	19	18.1	45.671
HNRNPH1;HNRNPH2	20.89704	20.98468	21.0732	0.088080366	+		2	2	2	8.87E-12	42544000	6	13.1	47.087
HNRNPK	24.11791	23.82677	23.84403	0.163335372			10	10	10	6.15E-54	439310000	62	37.4	47.557
HNRNPM	24.68992	24.41409	24.50972	0.14005911			18	16	15	1.10E-68	838730000	106	38.6	73.62
HNRNPU	22.49753	22.44415	22.2554	0.127215646	+		4	4	4	2.72E-23	161940000	20	8.8	88.979
HPCAL1	24.77027	24.91118	24.63832	0.136454516	+		11	10	9	1.42E-66	1026100000	133	59.6	22.313
HPRT1	22.73463	22.78443	22.53193	0.133743274			6	6	5	8.38E-27	210780000	36	42.7	24.579
HRAS	20.87658	20.75792	20.9234	0.085299412			2	2	2	5.63E-21	84686000	7	31.7	21.298
HSD17B10	21.71565	21.78859	21.91196	0.099228706			4	3	4	2.36E-16	150590000	25	36.8	26.923
HSD17B12	22.51331	22.32545	22.1757	0.169163114			6	6	6	8.83E-29	214080000	30	34	34.324
HSD17B4	21.25113	21.17225	21.37857	0.104108077	+		3	4	2	1.58E-18	86934000	9	11	79.685
HSP90AA1	26.13283	26.09537	25.94521	0.099291273	+		17	15	16	0	2064600000	195	45.4	84.659
HSP90AB1	27.31691	27.32199	27.18348	0.078543397			29	27	26	0	5693500000	458	51.8	83.263
HSP90B1	24.94065	24.9888	24.80405	0.09583888			16	16	15	2.90E-86	1018400000	112	33.6	92.468

HSPA1A	28.20494	28.328	28.02707	0.151294616			27	27	26	0	10483000000	729	61.2	70.051
HSPA4	23.51437	23.82026	23.3159	0.254079398			14	14	12	7.24E-63	473980000	72	29.6	94.33
HSPA4L	19.32562	NaN	NaN	N/A			2	1	1	4.67E-12	16792000	3	6.4	94.511
HSPA5	23.78237	23.80784	23.80892	0.015026586			8	10	10	1.34E-82	509340000	56	28.7	72.332
HSPA8	26.09774	26.16191	25.98657	0.088713653			23	23	23	1.19E-192	2503100000	271	48.5	70.897
HSPA9	24.09362	23.93193	24.24866	0.158376635			11	11	11	1.52E-78	628080000	86	26.8	73.68
HSPB1	21.11915	21.08971	21.37915	0.159291257			3	2	2	2.27E-10	68430000	9	19.4	20.406
HSPD1	26.5216	26.50756	26.41555	0.057604346			25	23	21	0	3457800000	307	55.7	61.054
HSPH1	21.53692	21.60494	21.37698	0.117027978			8	6	6	1.87E-20	112350000	17	12.8	92.115
IDH2	NaN	20.87575	21.01236	0.096597857			1	2	2	9.00E-09	40406000	9	10.2	36.171
IDH3A	21.45378	21.23327	21.39299	0.113893636			3	3	3	3.39E-16	93357000	18	20.2	35.786
IFITM2;IFITM3;IFITM	24.92066	25.61158	25.73472	0.438791444			3	3	3	3.84E-81	1950500000	79	29.5	14.632
IGF2R	20.73463	20.76919	NaN	0.02443761	+		4	4	3	8.09E-19	54818000	14	3.5	274.37
ILF2	21.58715	22.03016	21.52741	0.274646527			4	3	4	6.80E-17	120860000	19	21	43.062
ILF3	22.7708	22.10428	22.14149	0.374536285			9	8	7	2.65E-22	136700000	36	15.1	74.606
IMMT	22.30502	21.69238	22.15768	0.319776207			6	6	5	1.41E-28	147690000	24	13.9	78.973
IMPDH2	22.05444	21.93211	22.11455	0.092971496			5	6	4	6.54E-18	147610000	28	21.6	55.804
IPO5	23.56581	23.71827	23.64141	0.076230868			9	7	6	5.23E-51	350980000	46	21.6	123.63
IPO7	22.89223	22.97207	23.00891	0.059645946			9	7	7	2.80E-41	285860000	44	13.1	119.52
IPO9	NaN	21.74698	NaN	N/A			1	2	0	3.91E-10	36722000	5	3.6	115.96
ISYNA1	20.65298	20.58793	NaN	0.045997296			2	2	1	1.02E-08	42013000	8	8.8	44.786
ITFG3	21.56653	21.777	21.8535	0.148605547			4	4	4	5.25E-20	114540000	21	12.1	59.659
ITM2B	22.70959	22.24377	22.58396	0.241004981			5	4	3	1.31E-46	192810000	18	30.8	30.338
JAM3	24.2126	23.89708	24.21948	0.184183769			7	7	5	1.64E-44	459360000	47	27.1	35.02
KATNAL2	NaN	NaN	20.54085	N/A			1	1	1	0.0014024	10899000	3	9.4	13.999
KDELR1	20.16054	NaN	NaN	N/A			2	1	1	2.19E-18	46071000	9	20	17.486
KHSRP	24.08845	24.25768	24.26799	0.100813111			13	11	11	1.86E-50	638440000	93	26.9	73.114
KIAA0319L	22.61894	22.16039	22.06523	0.296062576			4	4	3	1.09E-29	185400000	20	7	109.75
KIAA0754	19.6241	NaN	NaN	N/A			2	2	2	7.69E-05	14523000	3	1.8	104.66
KIAA2013	23.33552	23.22897	22.65095	0.368351306			5	4	7	1.22E-30	255300000	30	15.8	69.156
KPNA2	21.51196	22.10306	21.91072	0.301497215			4	4	4	2.04E-17	132160000	21	18.9	57.861
KPNB1	25.55415	25.40915	25.51036	0.074370725			17	16	19	3.10E-111	1655100000	203	35.7	97.169
KRTAP11-1	NaN	19.92511	NaN	N/A			0	1	0	0.00012288	3470600	2	6.7	17.085
LAMTOR1	26.63159	26.98869	26.68016	0.193679381	+	+	10	11	10	2.99E-230	3445100000	334	92.5	17.745
LANCL2	20.45473	20.20263	20.77225	0.285435429	+		3	3	3	6.60E-12	47151000	15	11.8	50.854
LARS	19.54642	NaN	NaN	N/A			2	1	1	0.00032032	21814000	4	1.9	129.21
LBR	21.40104	21.70383	21.77503	0.198586566			3	3	3	4.27E-08	108180000	18	4.2	70.702
LDHA	26.95016	27.06661	26.53218	0.281034787	+		14	14	13	7.36E-182	3054500000	215	61.1	36.688
LDHB	27.81311	27.85757	27.79501	0.032192274			17	18	15	1.15E-180	6478300000	312	71.6	36.638
LMAN2	20.43522	20.71095	20.80081	0.190507174			3	2	3	1.24E-08	52113000	8	8.4	40.228
LMBRD1	23.5013	23.08378	23.46438	0.231135745			3	2	2	4.29E-43	389790000	25	9.9	44.211
LNP;KIAA1715	24.57594	24.83682	24.50827	0.173485209	+		12	12	11	1.25E-77	1185000000	137	40.4	47.094
LNPEP	24.86179	24.6666	24.66324	0.113675362			20	21	17	3.65E-109	1061600000	132	27.7	117.35
LOH12CR1	23.64405	23.40873	23.7168	0.161025459	+	+	7	7	7	7.39E-50	499240000	48	64.4	20.247
LRPPRC	23.97771	23.78556	24.09321	0.155408277			16	15	15	2.26E-75	620490000	91	23.1	157.9
LRRC1	21.61459	21.70826	21.34006	0.191360058			3	4	3	1.12E-54	125160000	19	34.4	59.241
LRRC57	25.25274	25.33118	25.19674	0.067531409	+	+	11	11	10	8.54E-69	1483700000	128	59	26.754
LRRC59	21.12729	21.50838	21.54501	0.23132277			3	3	3	2.25E-09	78528000	23	10.4	34.93
LSR	24.92565	24.98555	24.86339	0.061083799			11	12	12	5.70E-101	1062900000	107	29.7	69.428
LTA4H	21.50978	21.13615	21.10661	0.224728739			5	5	4	2.96E-18	85985000	12	18	69.284

LUC7L2	NaN	21.06224	21.76685	0.498234509			1	2	2	1.19E-07	99584000	17	7.9	46.513
LUC7L3	21.89497	21.66353	21.79317	0.115998738			3	3	3	2.26E-13	99675000	14	14.6	42.571
LYN	24.2527	24.49753	24.20434	0.157183966	+		14	14	12	2.96E-104	852380000	94	43.9	58.573
LYPLA1	21.32509	21.10559	21.13558	0.119019389			3	3	3	2.27E-11	104500000	12	23.4	20.861
LYPLA2	NaN	21.84617	NaN	N/A			1	2	1	5.01E-26	112620000	18	22.5	24.737
LZTS1	20.58536	20.19976	20.38732	0.192823734	+		2	2	3	5.99E-09	46441000	8	7.7	55.611
LZTS2	19.84417	NaN	19.79638	0.033792633			2	1	3	7.62E-08	19438000	3	5.8	72.759
M6PR	23.33361	23.41235	23.34584	0.042373617			4	4	4	3.55E-16	316110000	23	22.4	30.993
MAN1B1	21.51615	22.44139	21.58233	0.516144833			4	4	4	5.03E-21	134040000	19	18.2	46.027
MAPRE1	23.74807	23.84785	24.02504	0.14027635			7	6	7	8.68E-38	537520000	64	46.3	29.999
MARC2	23.70883	23.50142	23.65992	0.10842305	+	+	10	9	9	1.70E-45	559900000	58	32.5	38.023
MARCKS	30.51644	29.9421	30.39958	0.303537654	+	+	12	12	12	0	30248000000	411	48.5	31.554
MARCKSL1	25.91232	25.80437	25.66319	0.124933817	+	+	5	6	5	3.32E-200	1698800000	76	60	19.529
MBLAC2	25.57831	25.29369	25.09672	0.242120718			8	9	8	6.01E-35	1133700000	41	50.2	31.371
MBOAT7	20.09636	NaN	NaN	N/A	+		2	1	1	3.63E-06	30575000	6	6	44.732
MCAM	21.05019	21.12666	NaN	0.054072456	+		2	2	1	1.17E-22	57399000	11	7.1	71.607
MCCC1	26.26917	26.04621	26.26012	0.126194659			23	20	24	0	3941200000	306	52.8	80.472
MCM3	20.76044	20.37071	20.47447	0.201839231			3	2	3	1.35E-09	51517000	11	4.3	90.98
MCM7	20.08537	NaN	19.93848	0.103866915			2	1	2	7.60E-07	23541000	5	7.4	60.643
MDFIC	22.0427	21.25799	21.95209	0.429292978			2	4	4	4.55E-13	77699000	13	27.6	16.46
MDH1	24.07079	24.41377	24.18185	0.175003091			8	9	8	1.96E-39	627720000	73	32	36.426
MDH2	24.73454	25.02627	24.71937	0.172975977			13	13	13	7.75E-70	965600000	139	55.6	35.503
ME2	20.24098	20.31584	20.42532	0.092710235			3	3	3	1.84E-13	55173000	10	8.7	65.443
METTL7B	20.54312	20.29266	20.38153	0.126977305			3	2	3	1.21E-08	60108000	13	14.3	27.775
MFSD5	20.98176	21.26751	20.94671	0.175970739			2	2	3	2.87E-16	88261000	16	15.8	49.764
MGRN1	23.18766	23.36666	22.8966	0.237245768	+		8	7	7	1.33E-44	390700000	63	35.5	58.304
MICB;MICA	20.60168	20.3418	20.67003	0.173178265	+		2	2	2	0.00028761	41059000	7	4.4	37.579
MIF	23.84747	24.00479	23.95172	0.080035482			2	2	2	5.32E-19	592000000	52	17.4	12.476
MLEC	23.38851	23.66112	22.89512	0.38826664			6	4	5	2.11E-27	431480000	46	53.4	16.729
MPDU1	19.86885	21.07816	19.89711	0.690182151			2	2	2	8.06E-06	26162000	5	23.8	10.978
MRPS36	23.23327	23.13163	22.88515	0.179011459			4	4	3	4.24E-57	303520000	38	56.3	11.466
MSH2	19.58503	NaN	19.59155	0.004610336	+		3	3	3	2.12E-08	23074000	11	4.1	97.321
MSN	22.84653	22.82353	22.9018	0.040228443			9	6	8	4.58E-42	294030000	32	17	67.819
MSRA	22.4659	22.21588	22.06556	0.202228509	+		3	3	3	6.79E-21	170100000	22	36.6	23.627
MTCH2	22.11207	21.50959	21.74111	0.303917473			4	3	4	5.69E-12	101210000	20	18.5	33.331
MTDH	23.3254	23.59343	22.87202	0.364651864			7	6	6	1.77E-46	302410000	38	19.4	63.836
MTHFD1	22.4878	22.77032	22.901	0.211198585			5	8	6	2.19E-41	250460000	49	17.3	101.56
MTHFD1L	19.36102	19.32052	19.16455	0.103736326	+		2	2	2	1.76E-08	20603000	5	4	105.79
MT-ND4	21.20419	20.76239	NaN	0.312399776			2	2	1	0.00029301	32159000	7	5.4	51.58
MYBBP1A	19.42767	19.62884	19.55544	0.101802179			3	2	2	2.16E-07	8901700	4	3.4	140.13
MYL12A;MYL12B;MY	21.81429	21.61473	21.57939	0.126656438			3	2	3	6.59E-17	118510000	23	24	19.794
MYL6	21.38126	21.69362	21.48905	0.158659141	+		4	4	4	3.51E-13	111070000	20	38.6	16.29
NAA15	20.9351	21.03503	20.88874	0.074761865			3	4	4	8.65E-13	72237000	12	5.8	101.27
NASP	23.50179	23.27668	23.46239	0.120218604			4	4	4	1.36E-25	355570000	49	7.5	85.237
NCAM1	25.6605	25.35904	25.38423	0.167251199			19	18	16	3.03E-90	1391000000	177	34.3	94.573
NCL	22.46991	22.39069	22.32466	0.072724746			5	6	5	5.53E-18	141170000	26	9.7	76.613
NCR3LG1	21.62275	21.55558	21.65997	0.052906221			4	3	4	8.92E-19	109650000	17	15	50.827
NCS1	23.82007	24.0611	23.68035	0.192608282	+		8	8	8	4.15E-44	479280000	83	62.6	21.878
NCSTN	21.49165	21.49404	21.39367	0.057271182			3	3	3	1.28E-15	103880000	16	10.4	50.26
NDFIP2	21.02317	20.72866	NaN	0.208250018			2	2	1	4.76E-09	53359000	12	18.9	26.019

NDUFA4	22.06434	22.29698	22.47623	0.206520905			4	4	3	2.60E-09	133990000	16	46.9	9.3697
NDUFAF4	25.51114	25.37918	25.06007	0.231915494	+		9	10	9	6.10E-46	1411000000	104	53.1	20.266
NDUFB7	24.6299	24.58256	24.51244	0.059097013	+		4	4	4	3.39E-56	964630000	161	51.1	16.402
NDUFV1	NaN	18.58377	NaN	N/A	+		1	1	0	0.00016646	1536000	2	13.9	15.642
NELFB	20.08693	NaN	NaN	N/A			3	3	2	4.34E-06	29454000	5	5.5	65.697
NME2;NME1-NME2;	24.33906	24.46663	24.46488	0.073152626			8	7	8	9.40E-34	736760000	88	58.4	30.137
NNT	21.0794	21.18471	21.00601	0.089823882			4	4	4	1.55E-14	73856000	13	5.8	113.89
NPC1	24.21764	24.15962	24.30424	0.072779146			8	9	8	9.01E-40	655480000	71	9.3	142.17
NPEPPS	22.86119	22.91885	23.15379	0.154992602			8	8	11	2.06E-43	283220000	56	20.1	102.99
NPM1	24.35743	23.9304	24.15754	0.213659859			5	6	5	1.66E-27	501850000	54	26.8	29.464
NR3C1	19.88533	19.66839	20.0084	0.172151091			2	2	2	8.68E-06	22580000	4	4.5	60.601
NRAS;KRAS	22.32042	22.36904	22.60709	0.15341197			3	4	3	5.52E-21	243830000	20	31.7	21.229
NRP1	21.32795	NaN	21.49531	0.118341391			3	2	2	9.80E-18	80978000	15	7.9	101.3
NSUN2	20.42029	20.39665	20.57404	0.096319887	+		3	3	3	6.40E-09	41635000	8	5.6	82.392
OAT	22.61955	22.54484	22.86047	0.164947632			6	6	6	9.80E-21	217160000	37	17.8	48.534
OGFRL1	22.70028	22.49551	22.78125	0.147271994	+		7	7	6	7.51E-42	254250000	28	26.6	51.251
OXCT1	20.29278	20.01141	20.06237	0.149919293			3	3	3	1.07E-09	24866000	6	7.9	56.157
P4HB	23.32375	23.19119	22.71942	0.317635492			8	9	7	3.70E-32	260700000	37	24.8	57.116
PA2G4	24.45092	24.43581	24.40886	0.021305939			8	9	8	5.62E-45	658510000	82	32	43.786
PABPC1	22.05702	22.19683	22.37523	0.159494514	+		6	6	3	4.81E-22	181350000	24	17.4	61.18
PAFAH2	NaN	20.95298	NaN	N/A	+		1	3	1	7.61E-15	56447000	5	19.9	44.035
PAICS	22.9171	23.5128	23.09897	0.305284145			7	7	7	2.21E-45	297540000	50	36.5	47.079
PAIP1	NaN	19.0149	NaN	N/A			1	2	1	2.01E-06	32710000	10	6	39.908
PALD1	21.34186	NaN	NaN	N/A	+		4	1	1	1.09E-22	91871000	18	10.7	96.753
PALM	22.99384	22.61975	23.06313	0.238512843			4	4	4	2.48E-13	226710000	31	14.2	42.075
PARK7	22.41243	22.3739	22.50882	0.069497002			3	4	5	7.71E-28	155670000	18	45.5	19.891
PARP1	22.39757	22.11801	22.2	0.143706912			5	4	4	5.75E-18	129390000	10	8.4	113.08
PC	27.66506	27.65915	27.88221	0.127112033	+		45	46	45	0	9941500000	976	54.9	129.63
PCBP1	23.99749	23.40912	23.69381	0.294236072			8	9	7	2.17E-35	524400000	48	37.4	37.497
PCBP2	23.52325	23.89735	23.51593	0.218130545	+		3	4	4	1.49E-32	401650000	40	31.9	38.15
PCCA	27.01457	26.88495	27.19141	0.153835118			32	31	29	2.86E-259	5692500000	474	66.1	77.047
PCCB	21.82131	20.87912	22.46389	0.797091275			3	5	4	5.88E-21	117820000	25	21.8	52.423
PCMTD2	25.03091	25.04343	24.97552	0.036139938	+	+	15	14	14	1.14E-75	1063100000	148	41.6	41.071
PCNA	22.7243	22.91628	21.74144	0.630227357			4	5	2	2.97E-33	260800000	27	47.1	28.768
PDCD6IP	20.92976	21.58426	20.91459	0.382330199			4	5	3	2.39E-14	74616000	16	7.9	96.022
PDHA1	19.93747	19.81226	19.88284	0.062774089			2	2	2	0.00010839	24946000	6	5	40.188
PDHB	20.682	20.59021	20.9655	0.195635817			4	3	4	1.07E-11	72446000	13	15.5	37.514
PDIA3	23.36853	23.34082	23.34706	0.014535833			9	8	9	6.59E-31	326690000	34	26.2	54.963
PDLIM1	NaN	NaN	20.76247	N/A			2	2	2	0.00018065	11887000	1	6.1	36.071
PEBP1	23.0469	22.45181	23.2467	0.413501765			3	5	3	5.73E-31	250460000	25	56.1	21.057
PFKP	21.4156	21.70763	21.50359	0.149808813	+		4	2	3	1.17E-10	54841000	8	6.3	85.315
PFN1	24.44506	24.60501	24.54975	0.08123799	+		6	6	6	3.43E-39	825540000	89	60	15.054
PGAM1	25.20922	25.06858	25.04306	0.089480003			12	11	9	1.57E-138	834370000	138	64.2	28.804
PGD	22.16026	22.26668	22.16478	0.060179256	+		7	7	5	5.96E-32	153130000	23	31.9	51.872
PGK1	26.06827	26.0038	25.94186	0.06320922			17	17	17	2.09E-152	2265100000	202	60	44.614
PGLS	21.82131	21.92495	21.90522	0.055032431			5	6	5	3.99E-18	135720000	20	45.7	27.547
PGM1	20.22081	20.74583	20.1603	0.32201264			3	3	3	9.58E-07	33366000	4	6.8	61.448
PGRMC1	21.64152	21.51461	21.91838	0.206473493			3	3	3	3.00E-10	107100000	10	20	21.671
PHB	22.95688	22.83364	22.62417	0.168207076			5	5	5	2.23E-16	163810000	24	30.3	22.27
PHGDH	23.8826	24.00118	23.85671	0.077031476			10	10	9	9.28E-66	538200000	70	22.1	56.65

PI4K2A	26.38637	26.90357	27.18452	0.404860504			24	26	26	0	3565500000	312	72.7	54.022
PI4K2B	24.03641	24.12021	24.17957	0.071926855			12	14	12	9.25E-55	577570000	72	38.5	54.744
PIK3R4	22.57775	22.98003	23.12308	0.282747623	+		10	11	7	3.04E-44	300740000	41	13.8	153.1
PITPNB	20.20789	20.26109	20.26774	0.032803671	+		3	3	3	1.92E-06	35896000	5	11.8	31.54
PKM;PKM2	26.32163	26.48014	26.27764	0.106510399			21	21	22	4.64E-254	2726200000	244	55.4	57.936
PLGRKT	24.18359	23.97325	23.80134	0.191446697	+		4	5	5	8.84E-31	635760000	58	34	17.201
PLIN3	22.40239	21.93182	22.55999	0.326820908			6	4	6	6.89E-20	136670000	24	16.8	45.803
PLP2	25.64075	25.34871	26.00747	0.330084751			2	2	2	7.94E-58	2075200000	53	27	16.691
PLS3	26.24245	26.21271	25.95746	0.156661168			23	23	22	5.61E-154	2425400000	264	47.9	70.81
PLSCR1	25.93261	25.9129	26.44528	0.301840873			8	8	8	3.70E-91	1962900000	137	34.7	34.217
PLSCR3	23.04654	23.02748	23.34232	0.17652824			4	4	4	6.59E-22	271030000	36	26.1	31.648
PLXNB2	20.55459	20.6063	20.63644	0.041395987			3	3	3	1.67E-11	56497000	9	2.6	205.12
PMPCA	NaN	21.01924	20.90991	0.077307984			1	3	2	1.09E-26	71790000	14	9.1	58.252
PMPCB	20.78572	20.76474	20.7858	0.012135969			2	2	2	2.59E-10	61659000	9	6.7	54.366
PNP	21.18465	21.64856	20.84644	0.402698189			4	4	4	2.36E-15	72023000	13	25.3	32.118
PODXL	22.16272	22.06658	NaN	0.067981246			2	3	1	1.04E-07	131340000	10	5.5	55.385
PPA1	20.79907	20.9522	NaN	0.108279261			2	2	2	7.87E-12	63727000	8	16.3	32.66
PPA2	19.57472	19.48117	19.70153	0.11059755			2	2	2	3.48E-05	23105000	8	10.8	25.991
PPIA	24.92701	25.09317	24.7156	0.189236377			9	9	9	6.20E-36	1036100000	80	52.7	18.012
PPIB	21.29092	21.58242	NaN	0.206121627			2	2	1	1.24E-12	91776000	14	19	23.742
PPM1A	25.88358	25.71605	25.78845	0.084021602	+	+	14	15	14	1.82E-170	2207400000	201	52.9	42.447
PPM1B	25.16204	25.25101	25.02081	0.116084462	+	+	11	11	11	3.95E-127	1203800000	101	45.5	52.642
PPM1G	28.28154	28.20415	28.17074	0.056836139	+	+	22	21	22	0	10020000000	538	63.9	59.271
PPP2R1A	24.54539	24.64356	24.41931	0.112414099			16	16	15	1.89E-64	776840000	100	39.4	65.308
PPP3CA	21.03106	22.09071	21.94946	0.575364847			5	4	4	1.52E-21	183470000	26	17.2	57.658
PPP3R1	25.20251	25.32093	25.16488	0.081435983	+	+	9	8	8	8.28E-69	1209900000	105	84.7	19.3
PRAF2;WDR45	23.45224	23.11412	23.10442	0.198073209			3	2	3	1.65E-12	381670000	38	26.4	19.258
PRDX1	25.45026	25.77251	25.94708	0.252041617			8	9	8	1.50E-46	1924100000	93	53.3	22.11
PRDX2	22.82782	23.1741	23.26026	0.228887796			3	3	3	2.54E-15	263400000	43	19.2	21.892
PRDX3	21.96568	22.00357	21.95468	0.025647886			4	4	4	2.50E-16	136690000	20	31.5	25.838
PRDX4	23.07248	22.47003	22.62778	0.312407094			3	4	5	3.40E-26	160150000	25	35.4	30.54
PRDX6	23.65818	23.34123	23.73542	0.208889555			8	8	7	1.10E-51	437690000	73	50.9	25.035
PRKAA1	22.21762	22.52499	22.66275	0.227886979			6	7	6	8.35E-25	246730000	38	20	64.009
PRKAB1	24.58824	24.64861	24.28828	0.192984562	+		7	7	7	1.57E-105	851550000	74	51.5	30.382
PRKAB2	22.17091	21.93258	21.64939	0.261081365	+		2	2	2	4.10E-17	105020000	18	16.9	30.302
PRKACA	27.29026	27.31743	27.28551	0.017222359	+	+	16	16	17	1.49E-121	4921500000	322	63.2	40.589
PRKACB	23.28814	24.2832	23.57762	0.511824548	+		3	4	4	3.38E-84	462600000	55	62.7	40.622
PRKAG1	20.08031	20.29872	20.81127	0.375213928	+		4	4	3	1.49E-12	70729000	9	20.2	28.285
PRKDC	22.90189	22.68989	23.01691	0.165889361			13	13	13	3.00E-48	270730000	56	5.8	469.08
PRMT1	21.9117	21.6446	21.57999	0.175854289	+		4	4	4	5.30E-21	137900000	19	25.5	37.709
PRNP	22.85352	22.50693	23.05393	0.276736275			5	5	5	1.08E-22	197070000	30	21.5	26.885
PROCR	21.42234	22.94623	20.56449	1.20629134			2	3	3	4.74E-21	190300000	23	20.6	26.671
PRPF19	21.46049	20.60286	21.01229	0.428961028			3	4	4	4.07E-13	88949000	9	8.7	55.18
PRPF38B	21.57824	21.59245	21.39702	0.108961383			6	5	6	1.13E-13	95439000	16	10.3	64.467
PSAT1	19.84771	20.05629	19.82324	0.128073351			2	2	2	4.57E-06	28048000	4	7.6	40.422
PSMA1	20.85643	21.13965	22.17865	0.696180089			4	3	3	1.52E-13	121390000	13	21.3	29.555
PSMA3	NaN	21.33767	NaN	N/A			1	2	1	1.19E-12	93841000	16	15.3	27.647
PSMA4	21.66562	22.02624	21.96003	0.191966923			4	4	4	4.97E-12	139480000	23	23.2	24.526
PSMA6	21.46717	21.44253	21.6865	0.134309431			4	3	3	4.80E-16	125800000	22	27.7	16.645
PSMA7	21.45091	21.09462	21.22205	0.180535254			2	2	2	5.66E-07	70511000	6	12.9	27.887

PSMB2	22.14083	22.4967	21.81937	0.338810645			3	3	2	2.48E-07	112340000	12	14.4	22.836
PSMB5	22.11146	22.01045	22.22199	0.105805697			3	4	4	2.35E-23	134430000	24	30.4	28.48
PSMB6	21.47269	21.39754	21.75132	0.186387807			3	3	3	6.56E-10	100060000	16	13	25.357
PSMC1	28.44679	28.58545	28.35283	0.117023603	+	+	27	24	23	0	13212000000	755	63	49.184
PSMC2	24.3345	24.67562	24.4108	0.179031737			11	11	13	1.29E-82	1221900000	91	50.8	48.633
PSMC3	23.49741	23.17048	23.31755	0.163738831			4	3	4	1.26E-20	259810000	19	19.4	47.352
PSMC4	21.58256	21.44045	21.73616	0.1478922			4	4	4	2.15E-10	94116000	11	11.6	43.507
PSMC5	24.80853	25.08107	25.41464	0.303566667			5	5	5	3.54E-25	1281500000	61	18.3	44.784
PSMC6	21.47556	21.86446	21.72301	0.196842922			5	5	5	9.29E-26	135310000	23	24.2	44.172
PSMD1	22.38958	21.98305	22.30694	0.214864586			4	5	4	6.08E-17	138060000	18	7.3	102.26
PSMD11	21.45116	21.23742	21.55922	0.163766598			3	3	4	5.40E-15	95605000	20	14.2	47.463
PSMD12	21.99493	21.19244	21.59414	0.401245086			4	4	2	3.20E-17	109250000	28	14	52.904
PSMD13	22.80489	22.89643	22.32064	0.309411204			6	5	5	3.41E-30	182720000	35	24.7	42.945
PSMD14	20.30017	20.14967	20.33189	0.097348632			2	2	2	3.58E-07	39075000	6	6.5	34.577
PSMD2	24.29999	24.40022	24.2737	0.066763922			8	8	8	5.34E-103	620610000	92	31.1	100.2
PSMD3	21.21792	20.99948	NaN	0.154460405			3	3	1	1.43E-17	95938000	13	16	41.183
PSMD6	20.8777	21.51456	21.11509	0.321849072			3	3	4	2.35E-14	79084000	11	20.3	45.531
PSMD8	21.91313	21.82862	22.15416	0.168924881			3	3	3	1.70E-13	118280000	23	29.7	19.781
PSME1	19.43602	21.4139	NaN	1.39857236			3	2	2	1.33E-10	44860000	7	22.3	26.87
PTBP1	21.25903	22.32509	20.8768	0.750569519			3	3	3	3.30E-15	51454000	7	11.5	57.221
PTGES3	NaN	20.40904	NaN	N/A			1	2	1	3.72E-06	71886000	9	20.9	16.476
PTGFRN	26.76091	26.77505	26.60116	0.096572714	+		26	27	26	2.36E-238	3469900000	299	43.5	98.555
PTK7	24.33374	24.18639	24.01402	0.160023081	+		15	15	12	5.17E-82	662050000	83	25	118.39
PTPLAD1	24.14351	24.1404	24.17843	0.021116184			9	8	8	1.61E-45	668090000	68	29.8	43.159
PTRH2	21.36035	21.70421	NaN	0.243145738			3	3	1	8.83E-13	71580000	9	29.1	19.193
PTTG1IP	22.67057	22.53438	22.8064	0.13601004	+		2	2	2	2.29E-11	222190000	34	22.8	20.324
PVR	24.83297	24.57888	24.6479	0.131387717			5	5	5	3.01E-21	686980000	43	13.5	39.304
QKI	21.05219	21.57089	21.0312	0.305711074			3	3	3	1.21E-07	75631000	12	10.1	35.232
RAB14	20.94442	20.9167	20.55609	0.216644163			2	2	2	0.00045471	30018000	4	7.9	23.897
RAB1A;RAB1B;RAB1	22.85089	22.49629	22.83944	0.201504419			4	3	2	1.52E-14	211000000	17	37	19.018
RAC1;RAC3;RAC2	22.08839	21.87785	21.7485	0.171553552			2	2	2	7.78E-12	123560000	16	27.1	21.45
RAD23B	22.21071	22.36901	22.29008	0.079150102			5	7	4	2.04E-15	152290000	21	21	43.171
RAN	24.30765	24.48468	24.26649	0.115931476			5	6	6	1.60E-29	734100000	68	31.5	24.423
RANBP1	22.55035	22.28771	22.53938	0.148569792	+		3	3	2	1.70E-12	233550000	21	15.4	23.31
RANGAP1	22.5383	22.4315	22.75356	0.164045599			7	6	8	2.84E-35	218280000	44	21.3	63.541
RAP1B;RAP1A	NaN	20.70312	20.59423	0.076996857			1	2	2	1.28E-05	42316000	6	43.8	5.351
RAP2A	19.76424	20.92209	NaN	0.818723587			2	2	2	3.08E-34	52166000	3	45.4	20.615
RAP2B	25.32879	25.24619	25.1795	0.074786162			7	7	7	1.44E-47	1130000000	103	62.3	20.504
RAP2C	22.97486	22.6745	22.10463	0.442015874			5	5	5	8.60E-42	187260000	24	59	20.745
RARS	20.73967	20.88547	20.48895	0.200560161			4	4	3	1.70E-11	49153000	9	8.2	75.378
RBM39	NaN	NaN	20.95837	N/A			2	2	2	2.95E-09	43638000	11	13.7	36.514
RCC2	20.64974	20.51768	20.70616	0.096736558			2	2	3	5.35E-09	47052000	8	6.5	56.084
REEP5	23.72692	23.46725	23.87699	0.20729865			3	3	3	2.29E-30	367700000	49	15.9	21.493
RER1	21.58596	NaN	NaN	N/A			2	1	1	8.78E-10	108970000	10	23.4	18.388
RFC2	20.33919	20.35337	20.30686	0.02383793			3	2	3	1.32E-08	29878000	8	10	35.243
RFTN1	21.32163	21.27623	21.39373	0.059253439	+		6	4	5	1.10E-18	128460000	17	14	63.145
RGS19	22.50514	22.32641	22.34218	0.098952065			3	3	3	1.36E-08	177650000	16	16.1	24.635
RHBDD2	21.13483	22.27108	21.4706	0.583748488			2	1	2	2.05E-09	71679000	20	11.8	39.202
RHOA;RHOC	20.57145	21.31645	20.82283	0.37900695			2	2	2	1.34E-13	58289000	13	23.8	21.768
RHOB	24.0344	23.804	23.9911	0.122450983			5	4	4	5.78E-22	585470000	52	36.2	22.123

RNF11	21.24016	NaN	NaN	N/A	+			2	1	1	7.54E-10	50622000	5	18.8	17.444
RNF141	24.31755	24.51779	24.42861	0.100319036	+			5	5	6	2.54E-57	837650000	80	57.4	25.535
RNH1	19.92699	20.2105	20.43929	0.256636602				2	3	3	1.43E-08	32885000	5	11.1	49.973
RP2	25.24793	25.0554	25.39044	0.168141162	+			10	12	10	3.04E-38	1427400000	115	26.9	39.641
RPA2	20.73859	NaN	NaN	N/A	+			2	1	1	0.00026343	40789000	9	11.2	19.433
RPL10	23.92107	23.24037	24.38377	0.575153217	+			5	5	5	7.64E-22	459850000	24	35.5	22.975
RPL10A	23.44506	23.40549	23.07397	0.203788732				4	4	3	2.39E-14	292930000	30	26.7	24.831
RPL11	23.13924	23.10658	23.02731	0.057559728				3	3	3	1.67E-78	237010000	24	22	20.124
RPL12	22.78173	22.71502	22.60709	0.088127028				5	5	5	3.58E-35	219220000	29	54.5	17.818
RPL13	28.3135	27.87976	28.40617	0.281017676				12	13	11	0	11066000000	425	46.9	24.261
RPL13A	25.19216	25.16427	25.45895	0.162681211				8	8	8	3.89E-25	1724200000	144	30	23.577
RPL14	22.49592	22.69487	22.71394	0.120745937				3	3	3	1.42E-12	156110000	25	27.4	14.558
RPL15	27.25296	27.06188	27.49564	0.217390925	+			14	16	16	7.79E-90	6068900000	363	52.5	24.146
RPL18	28.39022	28.52391	28.87444	0.250071092	+			10	10	8	6.72E-251	14472000000	493	54.3	21.634
RPL18A	23.03636	23.53136	23.66395	0.330775431				4	4	4	1.25E-17	314340000	33	27.7	16.714
RPL19	24.72448	24.84336	25.20012	0.247535708	+			4	4	3	2.32E-38	1153000000	64	22.3	23.134
RPL21	22.11518	NaN	22.53429	0.296355523				2	1	2	3.16E-16	112990000	17	20.6	18.565
RPL22	23.23725	NaN	23.14146	0.067733759				2	1	2	8.96E-09	262420000	17	51.1	5.0827
RPL23	23.79729	23.77022	23.93921	0.090766818				5	5	5	9.67E-32	516530000	48	43.6	14.865
RPL24	22.929	22.99388	23.23317	0.16020222				5	4	4	1.08E-14	321030000	36	39.7	14.369
RPL27	22.08654	22.02827	22.02746	0.033878448	+			3	3	3	3.10E-10	147100000	28	20.6	15.798
RPL27A	25.00187	25.02132	25.54934	0.310619495				6	6	6	2.39E-23	1801400000	126	35.8	16.561
RPL28	27.20564	26.77555	27.74874	0.487687365				12	13	12	7.10E-92	4380000000	195	61.3	15.747
RPL29	25.19603	24.78102	25.95711	0.596470697				4	4	3	1.11E-21	1775800000	45	23.3	17.752
RPL3	24.46114	24.31927	24.44038	0.076622132				12	12	12	4.95E-48	626430000	72	33	46.108
RPL30	21.5552	21.30742	21.47833	0.126829382				2	2	2	1.65E-14	108060000	13	31.6	12.656
RPL31	24.94588	24.75827	25.27149	0.259683831				4	3	4	4.45E-157	1333500000	79	32.8	14.463
RPL32	29.43094	29.14513	29.9077	0.385248937				14	14	14	2.10E-291	28991000000	1376	60.2	15.616
RPL34	28.09763	27.71273	28.46123	0.374300508				9	9	8	6.28E-37	6206000000	189	35.9	13.293
RPL35	24.35783	24.07398	25.0842	0.521013219				5	5	5	5.74E-39	733910000	61	29.3	14.551
RPL35A	22.23298	22.08958	NaN	0.101399112				2	2	1	7.56E-07	113420000	9	20.9	12.538
RPL36	26.95859	26.97891	27.63672	0.385786468				6	8	8	2.39E-70	5545600000	202	43.8	12.254
RPL36A;RPL36AL	22.32314	21.45563	22.32699	0.501972222				2	3	2	1.62E-08	125080000	26	32.1	12.441
RPL37A;RPL37L	NaN	NaN	21.32279	N/A				2	2	2	0.00010104	38711000	5	29.3	6.5677
RPL4	25.99962	25.63118	26.01434	0.217093028				13	13	12	1.90E-66	1959600000	173	45.9	47.697
RPL5	24.49692	24.09136	23.99326	0.267013083	+			7	7	7	2.65E-37	470850000	36	35	34.362
RPL6	25.95897	25.48898	25.86738	0.249153864				12	12	10	2.23E-60	2273300000	215	42.7	32.728
RPL7	23.91013	24.13038	23.84871	0.148110582				8	8	7	4.70E-28	522110000	51	34.7	29.225
RPL7A	23.98146	24.16092	24.1368	0.097397965				8	8	6	4.33E-24	471840000	43	36.8	29.995
RPL8	23.87483	23.59491	23.87258	0.1609663	+			4	3	3	2.66E-24	370010000	47	20.2	28.024
RPL9	22.32638	22.50821	22.41258	0.090955745				3	3	2	7.12E-18	169980000	26	33.3	21.863
RPLP0;RPLP0P6	24.76289	25.11617	24.73195	0.213459227				8	8	8	5.74E-73	910410000	121	42.3	34.273
RPN1	NaN	20.75189	20.85255	0.071177369				2	3	3	6.79E-15	59327000	12	11.5	68.569
RPRD1B	19.64121	NaN	NaN	N/A				2	1	1	8.37E-09	38774000	6	11.7	36.899
RPS11	23.18123	22.86393	23.34232	0.243408528				6	8	5	3.49E-19	242910000	32	51.3	18.431
RPS12	22.26491	22.50237	22.0112	0.245629798				3	2	3	2.18E-18	177240000	28	31.8	14.515
RPS14	22.75681	22.79223	23.06434	0.16826226				3	3	3	3.13E-22	262850000	34	31.8	16.273
RPS15A	23.16783	23.27526	23.46874	0.152491827				4	4	4	2.46E-16	377120000	32	54	11.477
RPS16	23.01163	23.3692	23.06337	0.193246527				7	5	4	1.02E-20	275580000	41	42.6	14.419
RPS17L;RPS17	20.56616	20.92904	20.52229	0.223253232	+			2	3	2	1.09E-09	48831000	10	48.9	15.55

RPS2	25.01597	24.73288	24.7685	0.154191512				10	9	10	5.94E-44	874410000	96	42.7	31.324
RPS23	23.27711	22.81016	23.68271	0.436634318	+			4	4	4	3.95E-15	264280000	51	28.7	15.807
RPS25	23.74868	23.23961	23.33811	0.270006864				4	3	4	1.89E-10	392320000	48	28	13.742
RPS26;RPS26P11	23.31769	22.73818	23.23513	0.313477085				2	2	2	8.42E-07	246780000	17	20.9	13.015
RPS27L	22.86198	22.54609	22.64467	0.16162103				3	3	3	1.47E-16	212080000	17	38.1	9.4771
RPS3	25.65924	25.47364	25.27923	0.19002202				13	13	12	2.23E-58	1285800000	114	70.8	26.688
RPS3A	24.8811	25.25306	25.10893	0.187542976				12	10	12	5.59E-45	1149400000	95	52.7	29.945
RPS4X	24.28863	24.16346	24.42034	0.128453875				12	12	11	3.27E-46	652170000	84	47.1	29.597
RPS5	24.86773	24.52085	24.37994	0.251037994				9	9	10	2.45E-60	768870000	99	54	22.391
RPS6	23.53505	23.69158	24.12841	0.307516444				4	4	4	5.45E-46	643410000	66	18.9	28.68
RPS8	23.93805	23.79749	24.21215	0.210883396	+			7	7	7	3.91E-72	560150000	85	43.3	24.205
RPS9	27.34232	27.19112	27.55245	0.18146415				15	15	14	2.54E-61	5611600000	278	50.5	22.591
RPSA;RPSAP58	22.15035	22.77913	22.97491	0.430811887				3	3	3	1.00E-33	198990000	22	28.1	29.404
RRAS	19.67235	NaN	20.34992	0.479114342				2	2	2	2.33E-21	27662000	5	22.5	23.48
RRAS2	24.72713	24.37683	24.3864	0.199540559				8	8	7	3.27E-37	827620000	62	47.5	23.399
RRM1	22.06046	22.07627	22.4891	0.243040057				5	6	6	1.16E-22	153340000	25	13.5	79.219
RRM2	19.83212	19.71552	19.82656	0.065772785	+			2	2	2	9.50E-06	20192000	4	8.3	33.789
RTN3	22.22258	21.93326	NaN	0.204580134				2	2	1	2.06E-09	119590000	25	11	25.609
RTN4	22.85984	23.18042	22.84973	0.188073401				4	4	4	2.39E-18	379170000	33	20	37.144
RUVBL1	21.17767	21.18973	NaN	0.008527708				4	3	2	3.14E-17	79579000	14	18.6	50.227
RUVBL2	22.32748	21.96497	21.97607	0.206165669				6	4	4	2.07E-20	146310000	19	22.2	51.156
S100A11	21.73355	21.53929	21.99786	0.230174995				2	2	2	3.09E-09	100500000	12	37.1	11.74
SAE1	19.96183	20.96951	20.00922	0.568597936				4	2	3	2.38E-10	44714000	8	19.6	29.422
SAMM50	23.3073	23.78746	23.41467	0.252010028	+			10	9	8	9.07E-36	424550000	40	32.2	51.976
SC5D	NaN	NaN	28.78967	N/A				0	0	1	0.0057254	771800000	2	4.7	35.3
SCAMP1	25.31448	24.13014	24.33497	0.632989785				4	4	4	4.82E-64	569710000	61	27.2	37.873
SCAMP2	24.86169	24.9829	24.82789	0.081509104				3	3	3	1.88E-99	1023500000	65	13.7	36.648
SCAMP3	26.01595	26.54782	26.28028	0.265936614				7	7	7	3.89E-222	3124400000	222	38.9	38.287
SCARB1	23.2967	23.44556	22.81149	0.331570206	+			5	5	5	1.53E-27	275860000	34	19.8	53.579
SCARB2	26.20004	26.52135	25.95717	0.282997357	+			12	13	11	1.66E-132	2898400000	170	37.4	54.29
SCARF2	21.42173	20.8662	21.5855	0.377011645				3	3	2	2.19E-13	87420000	12	5.7	91.815
SCRIB	22.76081	23.12152	22.30977	0.406711783				9	8	9	9.44E-47	272650000	50	10.9	174.88
SCYL3	19.96028	21.62069	20.11235	0.917893985	+			3	3	2	2.18E-19	95085000	13	14.5	65.298
SDHA	21.08005	20.57311	20.84552	0.253705765				2	2	2	6.04E-08	46273000	8	4.5	63.566
SEC22B	NaN	20.3672	NaN	N/A				1	2	1	1.29E-07	30106000	6	14.9	24.593
SEC61A1;SEC61A2	23.28785	23.06878	23.57126	0.251925599	+			5	4	5	1.48E-18	322140000	35	12.4	52.264
SEC62	NaN	NaN	20.01658	N/A				1	1	2	0.00077966	27566000	5	20	9.8703
SEPT11	20.32608	20.27776	NaN	0.0341674				2	2	1	4.85E-07	38501000	5	9.4	49.005
SERBP1	22.13352	21.90845	22.87837	0.507642071				3	2	3	3.69E-17	57828000	12	12.9	42.426
SERINC1	24.49674	24.56023	24.53202	0.03181054	+			3	3	3	1.04E-99	1033300000	61	10.4	50.494
SERINC3	22.22016	22.51598	22.08793	0.219173085	+			3	3	2	2.88E-12	143080000	20	10	46.821
SERPINB6	22.74014	22.83208	22.64862	0.09173008				7	5	6	5.20E-31	277920000	38	39.4	42.621
SERPINH1	21.3849	NaN	21.50543	0.08522758				2	1	2	1.58E-28	73732000	13	16.5	46.44
SF1	NaN	19.60031	NaN	N/A				0	1	0	0.0081145	1630900	1	4.5	30.159
SFPQ	20.32345	NaN	20.56644	0.171819877				2	1	2	0.00024597	26672000	2	3.7	76.149
SFT2D2	21.28737	21.11483	NaN	0.122004204				1	1	1	4.92E-08	72172000	10	15.7	11.756
SFT2D3	23.9049	23.97998	24.06299	0.079078141				4	4	4	6.38E-56	527760000	46	27	21.789
SFXN1	23.04925	22.563	22.57741	0.276670591				6	6	5	3.46E-56	239560000	40	35.1	35.619
SGTA	21.91474	21.52066	21.40005	0.269181356				5	4	4	3.08E-10	51623000	9	17.3	34.063
SHISA2	21.49814	22.10655	22.13843	0.360820904				3	3	3	4.51E-12	96252000	9	15.6	31.375

SHMT2	23.98598	23.97754	23.78547	0.113406627	+		13	13	13	4.41E-48	546940000	84	35.6	53.454
SLC17A5	21.70324	22.27949	22.49219	0.408196737			5	5	4	1.10E-13	157670000	26	9.3	54.639
SLC19A1	20.51278	20.61117	20.70767	0.097446527			2	2	2	1.35E-05	54581000	9	3.9	64.868
SLC1A4	21.52784	21.79828	21.47497	0.173427404			4	3	3	4.31E-23	136400000	16	17.7	55.722
SLC1A5	25.84666	26.3181	25.8583	0.268888826			13	13	13	1.12E-218	2311700000	223	35.3	56.598
SLC25A11	21.66822	NaN	NaN	N/A			3	2	2	5.51E-15	70193000	14	19.9	32.182
SLC25A22	19.84649	NaN	19.47417	0.263269997			2	1	2	0.0014872	17154000	2	9.6	34.47
SLC25A24	20.61953	19.7125	NaN	0.641367064			3	3	2	3.71E-10	35974000	6	11.1	51.354
SLC25A3	23.71261	23.79213	23.51123	0.144788435			4	4	3	1.31E-19	560370000	45	19.7	39.958
SLC25A4	21.21502	21.42275	21.20675	0.122390186	+		2	2	2	4.96E-48	59818000	10	36.2	33.064
SLC25A5	26.58319	26.57831	26.432	0.085915508			13	14	11	8.87E-68	3072700000	207	42.3	32.852
SLC25A6	22.66902	23.32691	22.49069	0.440432501			4	3	3	6.07E-53	281390000	32	41.9	32.866
SLC29A1	21.26568	20.5383	20.80041	0.368388267			3	3	2	4.38E-15	44667000	7	9.4	50.219
SLC30A1	23.10119	23.20441	23.23719	0.070975227	+		5	3	5	2.42E-49	363320000	36	19.7	55.299
SLC35B2	25.25259	25.49674	25.5848	0.17210814			14	13	13	1.41E-51	1805100000	159	33.7	42.127
SLC35F6;C2orf18	21.19141	21.20789	21.00079	0.115107185	+		2	2	2	1.31E-17	67738000	7	5.7	40.214
SLC38A1	23.51244	23.33484	23.52193	0.105383813			6	5	4	2.42E-37	413470000	34	10.7	54.047
SLC38A2	24.63273	24.54044	24.35803	0.139792073			4	4	4	3.92E-116	894860000	80	17.6	56.025
SLC39A10	21.24098	20.76579	20.93919	0.240468397			3	2	2	1.34E-08	65422000	11	4.5	94.131
SLC39A14	21.16319	21.1142	21.17091	0.030756145			2	2	2	7.43E-09	41097000	5	21.3	17
SLC3A2	21.77929	22.37711	22.11124	0.299518061			6	5	5	8.84E-24	135560000	25	15.7	57.944
SLC41A3	21.82509	21.73781	22.3456	0.328622667			3	3	3	3.59E-19	102730000	17	30.3	16.43
SLC44A1	26.00734	26.23475	26.05424	0.120068576	+	+	15	12	13	5.52E-105	2309200000	198	28.3	73.301
SLC44A2	22.06927	22.13865	NaN	0.049059068	+		2	2	1	1.85E-18	218350000	28	8.5	79.845
SLC5A6	21.66679	22.37788	22.18553	0.367817595			4	4	2	7.31E-15	165610000	21	8.5	68.641
SLC7A1	24.08707	23.82036	23.5798	0.253747312	+		6	6	5	3.35E-89	466560000	33	14.8	67.638
SLC7A2	22.90202	22.50087	22.90178	0.23153481			6	5	6	8.12E-34	284510000	22	16.1	71.672
SLC7A5	22.35383	21.78895	21.93355	0.293437629			4	3	3	4.19E-17	148750000	19	11.2	55.01
SLC9A6	21.97816	21.7367	21.05345	0.47962174			4	3	4	4.50E-22	104130000	16	12.3	72.259
SMS	21.6802	22.042	21.9607	0.18981973			5	4	3	2.30E-10	137700000	20	14.7	35.278
SNAP23	26.09921	25.88409	26.02139	0.108921881			12	11	12	3.56E-114	1986900000	175	76.3	23.354
SND1	19.77828	NaN	19.80895	0.021686965			2	1	2	1.35E-11	24508000	10	6.7	102
SNRNP70	23.15413	23.50082	22.84011	0.330489592			8	7	7	3.76E-20	242320000	22	29.7	51.556
SNRPD2	20.98245	20.82073	20.96353	0.088414898			3	3	3	2.73E-09	58707000	10	31.4	13.527
SNRPN;SNRPB	20.94678	20.92296	20.41194	0.3021486			2	2	2	0.00019726	41760000	10	8.9	17.546
SPAG1	19.88044	NaN	19.95745	0.054454293			2	1	2	1.89E-06	29277000	7	3.2	103.64
SPECC1	20.50009	20.57681	21.44844	0.526781507	+		3	3	3	7.44E-09	68971000	8	5.5	79.014
SPPL2A	22.17442	21.24806	NaN	0.655035438	+		3	2	1	2.35E-08	79628000	16	6.5	58.143
SPRYD7	23.64548	23.06776	23.33497	0.289130317			5	6	6	2.07E-33	294390000	38	45.9	21.666
SRC	26.10358	26.01188	25.96069	0.072395739	+		21	20	21	2.08E-186	3108200000	243	51.5	59.834
SRI	21.03181	20.49003	20.85757	0.276577535			2	2	2	1.09E-06	49049000	8	19.4	17.605
SRP68	21.20562	20.99362	21.07431	0.107002486	+		4	3	4	1.48E-10	70576000	9	8.2	60.284
SRRM2	29.70408	29.51143	30.231	0.372500491			84	83	85	0	35497000000	2305	39.3	299.61
SRSF11	21.10578	21.61126	21.85935	0.384041322			2	2	2	2.51E-08	95271000	13	10	42.316
SRSF6;SRSF5;SRSF4	21.06645	NaN	21.66973	0.426583379			2	1	2	1.98E-05	42883000	5	5.4	38.418
SRSF7	19.57775	NaN	20.57515	0.705268304			2	1	2	0.00016403	15109000	4	15.9	15.257
ST13;ST13P5;ST13P4	22.7512	23.3332	23.00213	0.291918142	+		4	4	3	6.50E-32	306690000	44	19.5	41.331
STARD3NL	NaN	19.8178	22.40047	1.826223471			2	2	2	3.26E-07	80567000	13	11.2	23.505
STBD1	20.71129	20.77459	21.22099	0.277811069	+		2	2	2	9.06E-14	70906000	12	16.8	39.007
STIP1	24.41047	24.38443	24.18299	0.124501209			10	10	11	2.83E-57	644080000	74	30.9	62.639

STOM	24.56546	24.00024	24.30292	0.282847451			10	9	9	3.73E-83	780410000	70	57.3	31.73
STRAP	21.073	21.05039	21.18725	0.073365448			2	4	3	5.78E-13	78375000	12	16.3	38.438
STT3A	22.58052	22.75689	22.39315	0.181897719			4	4	4	2.44E-24	245150000	32	10.4	80.529
STX10	20.46483	20.07405	20.33647	0.199185656			2	2	2	4.33E-13	45904000	9	70.7	6.3891
STX12	24.1689	24.27014	23.74981	0.275871128			6	6	6	1.30E-55	606690000	74	35.5	31.642
STX6	20.87125	20.82353	20.99914	0.090803747			4	4	2	1.11E-22	68112000	12	25.1	29.176
STX7	24.78506	25.16802	25.01563	0.192805428			7	7	6	5.92E-173	1058100000	101	46.9	27.4
STX8	22.81114	22.46625	22.35727	0.236933088			4	5	5	6.42E-28	300370000	43	45.3	26.906
SURF4	23.63145	24.44158	23.02374	0.711324156	+		6	6	6	4.72E-28	422940000	97	39.8	21.127
SVIP	24.43377	24.06291	24.2988	0.187704614	+		2	2	2	8.99E-31	639530000	63	32.5	8.4426
SYNGR2	22.0097	22.18092	22.54743	0.274711795			2	3	3	1.06E-10	223740000	27	16.1	24.81
SYNJ2	NaN	26.4066	NaN	N/A			0	2	0	0.00070956	439200000	4	1.8	143.44
TAGLN2	25.31406	25.20068	25.06723	0.123550918			9	8	8	3.16E-65	1466600000	145	80.4	22.391
TALDO1	23.99559	24.49454	24.10693	0.261912584			11	11	10	1.04E-38	609410000	81	35	37.54
TARS	22.68646	22.2436	22.58405	0.231847288			6	6	5	1.24E-26	212400000	45	14.7	83.434
TBCD	NaN	19.12765	NaN	N/A			1	2	1	6.38E-05	7366700	1	2.8	84.262
TCP1	23.78417	24.1673	24.04618	0.195834918			18	19	17	1.25E-68	605240000	99	50.2	60.343
TECR	21.92857	21.68436	21.47764	0.225724592	+		4	4	4	4.07E-10	102970000	10	11.7	36.034
TESC	21.79162	22.11343	22.2392	0.230834582	+		2	3	2	1.32E-21	141630000	21	34.8	21.521
TFRC	27.14326	27.43102	27.02454	0.209016047			29	31	26	1.90E-297	5380000000	446	51.3	84.87
TKT	24.08278	23.9729	23.70187	0.196054118			8	8	9	3.65E-33	503470000	50	26.2	62.878
TLDC1	24.23659	24.40834	24.22986	0.101158676	+		8	8	7	5.97E-70	767030000	92	36.4	50.993
TLN1	20.93258	20.9132	21.39472	0.272583466			5	5	4	2.00E-13	76736000	11	3.7	258.08
TM9SF2	NaN	22.53509	22.54234	0.005126524			2	3	3	6.82E-12	114100000	9	6.3	75.775
TM9SF3	21.72238	22.09426	22.29606	0.29101166			4	5	5	1.12E-13	166180000	27	8.3	67.887
TMEM106B	24.85614	24.60682	25.22494	0.310978626	+		8	8	7	5.78E-78	1069200000	78	36.9	31.127
TMEM106C	21.78831	21.77981	22.42304	0.368939755	+	+	3	3	3	1.97E-19	177700000	20	14.4	27.875
TMEM115	NaN	22.27935	21.80247	0.337205082			1	2	2	7.90E-18	106060000	12	14.2	38.197
TMEM168	22.41738	22.73204	22.36172	0.199685525			5	4	4	2.84E-33	223970000	29	15.5	79.754
TMEM179B	23.28475	23.50215	23.21861	0.148342055			2	2	2	1.01E-15	270410000	32	15.5	23.55
TMEM181	22.10866	21.74694	21.95134	0.181369926	+		3	3	3	8.67E-13	133400000	13	6.5	69.324
TMEM184C	22.15983	NaN	22.42798	0.189610683			3	1	3	7.65E-76	224280000	13	9.6	50.141
TMEM192	NaN	19.92645	NaN	N/A			1	3	1	4.37E-16	69731000	12	21.7	30.549
TMEM222	NaN	21.02499	21.07927	0.038381756			1	2	2	1.01E-12	70079000	9	24.6	19.862
TMEM33	22.68207	22.71716	22.67068	0.024226124			4	4	4	1.04E-23	204970000	41	23.5	27.978
TMEM50A	23.21068	22.57194	22.68265	0.341335898			3	3	3	5.78E-18	279530000	32	29.3	17.4
TMEM50B	21.61095	NaN	NaN	N/A			2	1	1	1.31E-08	93251000	14	15.2	16.854
TMEM55B	22.99747	23.11009	23.12098	0.068381987	+		4	4	3	2.44E-23	300580000	36	26	29.469
TMEM59	20.91408	20.92426	22.54762	0.940199824			3	2	2	5.39E-11	58491000	3	19.8	21.86
TMEM63A	NaN	21.46757	21.001	0.329914811			2	2	3	4.58E-14	102570000	16	6.3	92.125
TMEM63B	23.15575	23.46862	23.46214	0.178794323			8	7	9	9.97E-31	377830000	50	11.7	94.957
TMEM65	20.66899	NaN	NaN	N/A			2	1	1	1.81E-05	17253000	3	8.8	25.498
TMEM87A	21.72787	22.15493	21.4864	0.338531231			2	3	3	1.28E-19	118680000	14	12.8	56.773
TMEM97	22.26442	22.1021	22.60463	0.256458907	+		2	2	2	0.00011607	175960000	14	10.2	20.848
TMPO	19.683	20.00744	20.04394	0.198692076			2	2	2	0.00010548	30332000	6	8.9	26.866
TMX1	25.45942	25.15163	25.3576	0.156804357			12	12	11	2.61E-59	1345300000	138	32.9	31.791
TMX3	21.99248	22.01754	21.9959	0.013589148			4	4	3	2.42E-18	165230000	30	18.1	51.871
TMX4	21.74821	21.41725	21.46204	0.17955219			3	2	3	3.34E-12	101070000	17	13.5	38.952
TNFRSF10A	24.00436	24.42496	23.94619	0.261249824			6	7	6	6.36E-32	539440000	62	19.2	50.089
TNPO1	22.22835	22.15613	22.28413	0.064175718			4	6	4	1.34E-30	188550000	41	15.6	101.31

TOMM40	23.53124	23.49814	23.60727	0.055954636	+		5	5	4	2.17E-68	340090000	26	16.6	37.893
TOMM70A	20.62676	NaN	NaN	N/A			2	2	2	5.92E-06	39208000	5	3	67.454
TPD52L2	21.28387	21.43125	21.39158	0.076262723			4	3	4	2.31E-15	92947000	19	25.2	22.237
TPI1	27.60513	27.74143	27.37451	0.185469477			15	16	15	0	5692200000	383	85.9	26.669
TPT1	22.44794	21.88353	22.32014	0.295950384	+		3	3	3	3.17E-17	115230000	14	25.6	19.595
TRAP1	23.28489	23.34854	23.27128	0.041242499			9	9	8	2.70E-72	474250000	68	24.9	74.267
TRAPPC3	24.90412	24.74016	24.50936	0.198320859	+		6	6	6	2.21E-32	966450000	75	41	15.005
TRIM28	22.70339	22.91262	22.55251	0.180841173			8	9	8	3.54E-41	187850000	37	12.6	88.549
TSN	NaN	20.91437	NaN	N/A			1	2	0	3.71E-07	53435000	12	13.5	21.01
TSPAN13	23.10732	22.74796	23.24229	0.25551212			5	5	5	8.27E-20	245730000	28	26	22.147
TSPAN14	22.11724	21.56532	22.21247	0.349401288			2	2	2	1.18E-11	151520000	20	14.6	28.876
TSPAN3	26.78481	26.90116	27.17665	0.201233963	+		4	4	4	4.69E-52	4316000000	77	38.6	25.183
TSPAN6	25.73317	25.68185	25.7034	0.025769484			6	7	7	2.38E-64	1773200000	128	37.1	27.563
TSPAN7	21.52473	21.52974	21.54976	0.013243875	+		2	2	2	1.34E-17	93774000	11	11.2	24.475
TSPAN9	21.51586	21.22664	NaN	0.204509423			2	2	1	5.39E-13	93473000	21	20.1	26.779
TTYH3	24.02488	23.63898	23.97325	0.209491806			6	7	6	2.75E-24	483680000	53	17.1	54.139
TUBA1B;TUBA4A	28.08441	28.15726	28.09874	0.038594174	+		19	18	19	0	9766700000	672	59.9	50.151
TUBA1C;TUBA1B	NaN	22.50661	22.39393	0.079676792	+		1	2	2	0	188330000	28	60.1	49.895
TUBB	25.84405	25.94069	25.73102	0.104941714			5	5	5	0	1743200000	133	58.2	47.766
TUBB2A	21.05404	20.80333	20.65561	0.20142127			3	3	3	0	52888000	15	47	49.906
TUBB4B;TUBB4A	27.53727	27.84822	27.5062	0.189135281			20	19	18	0	7070500000	705	59.6	49.83
TUBB8	NaN	NaN	22.7094	N/A			1	1	1	8.60E-137	179240000	8	24.3	49.775
TUFM	22.41007	22.67023	22.39218	0.155625127			6	6	6	2.31E-26	190850000	34	18.6	49.541
TXNDC5	21.17158	20.19832	21.85415	0.832156818			2	2	2	1.91E-12	51511000	12	16	36.177
UBA1	26.0044	26.12524	25.62611	0.260395976	+		21	20	22	2.88E-151	1914800000	205	37.9	117.85
UBB;RPS27A;UBC;UE	25.83959	25.21823	25.89631	0.376186544			4	4	4	4.96E-69	1894000000	149	50.5	10.469
UBE2I	21.38459	NaN	21.46931	0.059906087			2	1	2	2.17E-09	54769000	11	32.9	7.8891
UBE2L3	NaN	NaN	20.79384	N/A			1	1	2	7.75E-06	39457000	5	34.4	14.121
UBE2M	21.73938	NaN	21.88614	0.103774991			4	3	3	3.89E-13	128940000	17	35.5	20.9
UBE2V1;TMEM189;U	21.23631	20.85673	20.75507	0.253642685			2	3	2	2.16E-10	73551000	16	24.5	16.495
UBTD2	21.92426	22.02225	22.14074	0.108401653	+		3	3	2	7.70E-28	122810000	29	24.4	26.189
UCHL1	24.74037	24.75929	24.78611	0.022983423			8	8	8	2.42E-73	1134600000	117	59.6	24.824
UQCRC1	21.34803	21.16048	21.6309	0.236814068			4	3	3	1.12E-13	85573000	11	15.4	52.645
UQCRQ	24.04009	23.70388	24.31831	0.307670754	+		6	6	6	2.33E-24	558670000	102	74.4	9.9062
USMG5	21.04407	22.03073	21.68551	0.500686209			2	2	2	1.37E-06	76824000	6	43.1	6.4575
USP12;USP46	20.3368	20.59012	20.52612	0.131725146			2	2	2	1.09E-07	56852000	8	10.5	42.857
USP14	NaN	20.55291	NaN	N/A			2	2	1	6.12E-08	34911000	3	8.3	51.086
USP32	NaN	NaN	20.60729	N/A	+		3	2	3	4.55E-12	43917000	8	3.8	181.65
USP7	22.94038	22.94006	22.71982	0.1272481			6	6	5	1.18E-32	338080000	40	13.5	117
USP9X	19.69568	19.49422	19.77811	0.146044724			2	2	2	1.67E-05	22603000	6	0.9	290.46
VAMP3;VAMP2	25.0354	25.16315	25.221	0.094968455			2	2	2	1.86E-157	1276700000	43	40	11.309
VAMP7	24.81778	24.56162	24.48265	0.175198137			8	8	7	1.13E-47	920570000	82	45.5	24.935
VANGL1	21.14998	21.0561	21.18918	0.068386622			2	2	2	6.72E-13	69084000	7	12.3	59.747
VAT1	20.84476	21.16196	NaN	0.224294271			3	3	1	3.82E-10	63337000	7	19.6	41.92
VCL	24.35924	24.50488	24.31437	0.099597847			21	22	22	2.16E-84	795900000	139	29.1	116.72
VCP	22.68511	22.62781	22.39121	0.155799048			8	9	8	4.53E-46	242750000	50	16.1	89.321
VDAC1	22.11915	22.44789	22.31025	0.165092887			3	4	4	4.21E-21	192250000	37	23	30.772
VDAC2	25.80646	26.15021	26.03825	0.175321471			12	10	9	1.60E-124	2606900000	161	61.6	31.566
VDAC3	22.57163	23.30869	22.95594	0.368642596			4	3	4	8.87E-26	297110000	32	22.3	30.658
XPO1	23.884	23.79432	23.78776	0.053770614			12	11	11	2.45E-56	788680000	72	22.7	123.38

XPO5	19.88713	20.29502	20.10393	0.204080001			3	3	3	1.23E-08	34216000	8	2.7	136.31
XRCC5	23.52731	24.11934	23.12109	0.501998883			9	6	6	8.52E-59	515630000	64	25.3	82.704
XRCC6	23.90058	24.10797	23.84967	0.136821861			12	12	9	1.79E-51	525410000	71	29.9	69.842
XXYLT1	19.78141	19.56965	19.95985	0.195336962	+		2	2	2	2.54E-06	33447000	7	13.9	21.816
YARS	21.25718	21.87508	21.63414	0.311435209	+		7	7	7	1.69E-18	101840000	19	14.4	59.143
YES1	24.121	23.69307	23.14933	0.486983891	+		7	8	6	6.69E-79	431550000	52	43.3	60.801
YWHAB	24.53362	24.67487	24.44367	0.116544702			7	6	6	1.99E-123	1147100000	78	48.4	27.85
YWHAE	26.51034	26.51039	26.51081	0.000258134			13	14	13	4.56E-90	3481600000	215	61.2	29.174
YWHAG	21.96007	22.30098	21.92369	0.208122911			3	3	3	1.59E-39	109930000	25	35.2	28.302
YWHAH	22.74821	22.24176	22.62533	0.264170728	+		4	3	4	4.43E-29	208060000	27	26.8	28.218
YWHAQ	23.78407	23.78247	23.69487	0.051044033			7	7	7	1.08E-99	513720000	81	45.7	27.764
YWHAZ	26.04822	26.01154	25.97852	0.034866012			9	9	10	3.32E-163	2284800000	171	53.1	27.745
ZDHHC13	23.10188	23.6015	23.53231	0.270701984			7	7	7	6.79E-36	411090000	46	14.8	70.86
ZDHHC18	22.36119	21.96659	23.23402	0.648577954			3	4	5	1.32E-22	155850000	24	20.6	42.03
ZDHHC20	20.56058	20.93027	NaN	0.261410306			2	2	1	4.93E-15	82370000	14	17.3	42.277
ZDHHC3	21.16368	21.55918	21.53938	0.222846277			2	2	2	3.38E-14	120960000	10	9	34.17
ZDHHC4	NaN	21.13314	NaN	N/A			1	2	1	6.89E-07	80415000	8	11.6	39.786
ZDHHC5	22.50618	22.22429	22.13524	0.193644157			4	3	3	1.03E-23	157730000	29	14.7	71.951
ZDHHC6	22.26112	22.76217	22.72434	0.279002682	+		7	5	6	1.94E-30	278770000	35	18.8	47.205
ZMPSTE24	21.73236	21.43018	21.68799	0.163170384	+		3	3	3	2.57E-07	82939000	18	5.5	54.812
ZNRF2	22.95715	22.87532	23.45889	0.315962319	+		6	6	5	2.14E-28	253960000	27	59.5	24.115

Reagent 7

Gene names	log2 LFQ intensity_1	log2 LFQ intensity_2	log2 LFQ intensity_3	St dev log2 LFQ	MG protein	PTM peptide	Razor + unique peptides_1	Razor + unique peptides_2	Razor + unique peptides_3	PEP	Intensity	MS/MS Count	Sequence coverage [%]	Mol. weight [kDa]
ABHD17A	NaN	NaN	22.41664	N/A			2	2	2	0.000161	17896000	1	6.1	33.989
ACACA	27.80298	27.784	27.70603	0.051379			17	15	16	2.47E-97	823990000	37	9.9	257.24
ACTG1;ACTB;POTE	26.97104	27.26123	27.06168	0.148462			5	5	5	2.87E-40	504590000	11	24.8	41.792
ALDOA;ALDOC	24.10109	24.65382	24.05648	0.332745			3	3	3	1.83E-54	75039000	5	15.1	39.42
ANP32B;ANP32A	NaN	NaN	21.7556	N/A			1	1	1	0.01283	23942000	0	5.1	17.956
APRT	NaN	NaN	22.92738	N/A			1	1	1	0.000792	24090000	3	20.6	6.9189
ARF1;ARF3	31.23078	31.13418	31.21824	0.052528	+		7	7	6	7.52E-98	8820000000	43	32	20.697
ARF5;ARF4;ARF3	24.72198	25.00693	24.45369	0.276662	+		2	2	2	1.53E-94	102350000	4	32.2	20.529
ARF6	25.5461	26.31403	25.70345	0.405644	+		1	1	1	6.99E-55	225000000	3	16	20.082
ARL1	27.25881	27.11217	26.83425	0.215636	+		3	2	3	4.82E-37	514820000	10	26.2	18.565
ASAH2	NaN	NaN	21.85969	N/A			1	1	1	0.022758	10412000	1	2	81.717
ATP5G1;ATP5G2;A	NaN	NaN	23.84661	N/A			1	1	1	0.012074	66674000	1	21.4	10.038
ATP5H	NaN	24.24873	NaN	N/A			2	2	1	3.21E-17	92608000	2	18	18.491
ATP9A	22.63462	22.38837	NaN	0.174125			2	2	1	0.000362	18705000	3	1.9	105.13
B3GNT1	NaN	NaN	24.03145	N/A			1	1	1	0.015744	48549000	2	3.4	47.119
B4GALT3	NaN	NaN	22.22535	N/A			1	1	1	0.001771	18536000	1	15.2	15.654
BAG5	22.73591	23.12094	23.29376	0.285573			2	2	2	9.91E-12	31528000	4	5.6	51.199
BCLAF1	23.28475	23.01516	24.41687	0.74377	+		2	2	2	3.58E-12	47540000	3	4.1	83.134
C10orf129;ACSM6	NaN	NaN	29.28897	N/A			1	1	1	0.003044	1212100000	3	8.5	15.82
C10orf71	NaN	NaN	22.22161	N/A			1	1	1	0.019008	8545500	0	0.8	156.47
C2orf18;SLC35F6	NaN	24.37239	NaN	N/A	+		1	1	0	0.00087	43361000	1	5.2	31.096
CACFD1	NaN	24.23608	NaN	N/A			1	1	0	1.88E-06	31941000	2	17.7	13.63
CAND1;CAND2	23.16702	23.36266	23.91296	0.386767			2	2	2	2.72E-06	42150000	4	2.5	117.89
CANX	29.76488	29.67445	29.55939	0.102991			16	16	15	1.87E-171	3243800000	63	31.8	67.567
CAV1	25.35948	25.56993	25.68459	0.16489			3	3	3	4.59E-19	172510000	5	26.3	19.177
CCNY	NaN	24.72474	NaN	N/A	+		2	2	1	1.44E-06	42131000	3	56.9	5.8916
CCT4	22.19081	NaN	22.52468	0.236082			2	2	2	0.000379	22619000	2	6.7	54.719
CCT8	NaN	22.88764	NaN	N/A			0	1	0	0.00899	9936000	1	4	51.586
CD151	28.54951	28.16863	27.82929	0.36031	+		2	2	2	1.64E-53	1184000000	11	12.6	28.295
CD164	NaN	NaN	23.2142	N/A			1	1	1	0.020908	26719000	2	5.7	16.736
CD63	29.13658	28.22853	29.98604	0.878918			2	2	2	1.09E-143	2261400000	12	18.6	16.021
CD81	31.89309	31.42382	31.65233	0.234662	+		11	11	11	0	12355000000	77	37.3	25.809
CD9	29.32079	28.89122	29.66345	0.386929			7	7	7	1.93E-84	2356200000	31	25.4	25.416
CFL1	22.64656	22.1642	21.88084	0.387146			1	1	1	0.010334	18546000	3	13.9	9.0904
CHAT	NaN	NaN	23.09545	N/A	+		0	1	1	0.017868	30504000	0	1.9	70.393
CHCHD3	26.64842	26.83605	27.0802	0.216506	+		4	4	4	2.46E-23	441040000	10	14.5	26.152
CHMP6	NaN	NaN	22.39942	N/A	+		1	1	1	3.35E-09	42796000	3	10.6	12.939
CKAP5	21.84192	NaN	NaN	N/A	+		1	0	0	0.001818	4926800	1	1	218.52
CLN3	NaN	NaN	22.72071	N/A	+		1	1	1	1.01E-09	27154000	3	10.4	15.252
CMTM4	NaN	NaN	22.95848	N/A			1	1	1	0.002299	15732000	2	8.6	15.77
CNTN3	NaN	NaN	24.92388	N/A			1	1	1	0.000479	98037000	0	0.8	112.88
COX8A	NaN	NaN	24.21623	N/A			1	1	1	3.06E-18	62952000	3	11.6	7.579
CPD	23.35609	23.24399	22.60535	0.404977			2	2	2	6.91E-05	33294000	5	2.1	126.49
CPED1	NaN	NaN	23.98824	N/A			1	1	1	0.006057	43681000	0	0.9	117.49
CS	24.14942	24.31119	23.77987	0.272347			2	2	2	7.77E-64	65405000	5	7.7	50.431
CSE1L	NaN	NaN	22.78391	N/A			1	2	1	0.000191	15184000	2	3	103.88

CUL9	NaN	NaN	34.65549	N/A				1	1	1	7.43E-07	27230000000	4	9.7	10.917
CYB5R3	29.19385	29.17331	29.22476	0.025899	+			9	10	9	1.39E-87	2182300000	41	39.9	34.234
CYP2D6	25.91959	NaN	NaN	N/A	+			1	0	0	0.023203	83189000	1	3.8	50.06
DDX46	26.08343	25.98468	26.39785	0.215763	+			4	3	4	7.09E-21	260820000	6	4	117.36
DEGS1	NaN	NaN	24.25926	N/A	+			2	2	2	1.69E-07	80267000	3	9.6	30.124
DIRC2	NaN	NaN	23.53053	N/A	+			1	1	1	0.002191	35915000	2	18.3	12.672
DNAJC5	26.44659	27.60017	27.35035	0.606896				5	5	4	3.47E-56	563110000	23	36.5	18.801
DYM	NaN	NaN	21.71364	N/A	+			1	1	1	0.005064	16148000	1	3.5	54.425
EEF1A1P5;EEF1A1	27.15547	26.73415	27.29903	0.293601	+			6	5	5	7.87E-20	500950000	9	13.4	50.184
EEF2	24.86915	25.32165	25.45839	0.308399				5	5	4	1.02E-14	142820000	9	6.2	95.337
EIF3M	NaN	NaN	23.02264	N/A				1	1	1	0.02251	23502000	2	10.8	11.733
EIF4A1;EIF4A2	NaN	NaN	20.24109	N/A				1	1	1	0.004878	10829000	2	11.1	13.906
EMP2	NaN	NaN	24.36359	N/A				1	1	1	0.012423	48678000	1	8.4	19.198
ENO1	25.18779	25.06464	25.61313	0.287785				3	3	3	5.37E-10	149050000	9	9.7	47.168
ERGIC2	NaN	23.05894	NaN	N/A				1	1	0	7.27E-20	20494000	1	13.5	18.339
ERGIC3	23.17217	24.26485	23.01817	0.679691				2	3	2	8.31E-10	46508000	4	10.2	43.222
FAM129A	23.65665	23.76274	23.7213	0.053466	+			2	2	2	1.69E-08	49577000	4	3	103.13
FAM129B	26.48279	26.69166	26.47985	0.121449	+			4	4	2	7.06E-22	363110000	10	9.4	82.682
FAM49B	26.98598	26.60343	27.35683	0.376715	+			4	3	4	1.12E-71	465620000	18	22.2	36.748
FAM84B	NaN	NaN	23.36613	N/A	+			1	1	1	1.54E-16	28649000	2	7.4	34.474
FKBP4	23.17937	23.31493	23.79917	0.325837				2	2	2	7.99E-09	40561000	4	7.6	51.804
FLOT2	23.42624	23.64581	23.91944	0.247093	+			1	1	1	0.000295	47426000	6	5.2	27.86
FLVCR1	NaN	NaN	25.42461	N/A				1	0	1	0.017583	171940000	1	8.2	30.744
FTO	NaN	NaN	24.92755	N/A				1	1	1	0.022673	154470000	1	5.7	24.263
GAPDH	25.37504	25.11565	25.79015	0.340233	+			4	4	3	8.57E-19	164410000	7	17.3	36.053
GNAI1	NaN	NaN	24.05101	N/A	+			1	1	1	3.32E-19	45478000	1	20.2	34.774
GNAI2	27.40844	26.95715	27.90495	0.47408	+			6	5	5	5.60E-69	691550000	17	35.3	34.934
GNAI3	27.09315	27.07643	26.5513	0.308124	+			8	8	7	1.22E-51	464360000	16	25.4	40.532
GNAS	NaN	NaN	22.66536	N/A	+			1	1	1	0.019707	10164000	0	7.5	18.254
GNB2L1	NaN	NaN	22.96659	N/A				1	0	1	0.001565	24078000	1	12.9	16.403
GOLIM4	NaN	NaN	23.61941	N/A	+			1	0	1	0.003103	29904000	1	2.4	78.759
GORASP2	NaN	NaN	21.66427	N/A	+			1	1	1	0.003362	18912000	2	5.2	39.768
GPI	24.71843	22.61975	24.25947	1.103312				3	3	3	2.83E-09	62598000	3	7.7	63.146
GPR32	NaN	NaN	20.43777	N/A				1	1	1	0.019859	2964600	0	3.4	40.087
GPRC5A	24.25602	24.28419	24.98394	0.412373				3	3	3	2.71E-11	86704000	6	10.4	40.251
GSPT1;GSPT2	22.42734	NaN	22.0623	0.258122				1	1	1	0.001548	17396000	2	10.6	22.169
GSTP1	25.50118	25.85253	25.80326	0.190231				4	4	4	1.43E-20	203220000	7	24.8	23.356
HADHB	NaN	NaN	22.83109	N/A				1	1	1	0.001294	15564000	2	4.3	37.924
HCCS	25.7655	26.19599	26.08631	0.223708	+			5	6	5	3.40E-48	251560000	17	32.1	30.601
HIGD1B	23.81939	NaN	NaN	N/A				1	0	0	0.017365	19401000	0	14.8	6.2901
HPCA;HPCAL1	NaN	NaN	24.00599	N/A	+			1	1	1	1.03E-07	58676000	3	8.3	22.427
HSP90AA1	26.38597	25.8641	26.54975	0.358071	+			3	3	3	3.13E-68	293270000	11	5.6	84.659
HSP90AB1	NaN	NaN	22.41124	N/A				1	1	1	1.11E-45	12627000	2	5.5	83.263
HSPA1A	27.55186	26.75608	27.81195	0.550117				9	8	9	4.80E-34	655380000	17	26.2	70.051
HSPD1	24.70788	NaN	24.92293	0.152063				1	1	1	0.000462	72094000	3	23.4	10.279
IFITM2;IFITM3;IFIT	24.58841	25.29814	25.29607	0.409167				1	1	1	0.000223	127370000	7	25.7	8.0866
JAM3	23.8041	23.65949	24.368	0.374362				3	3	2	3.28E-06	58745000	3	14.3	29.223
KDELR1;KDELR2;KD	24.11545	23.85472	24.40145	0.273462				2	2	2	2.68E-13	65926000	4	17.3	17.486
KIAA2013	NaN	22.99814	NaN	N/A				1	2	1	1.59E-05	20192000	2	5.2	69.156
KPNB1	22.81705	22.85201	23.70651	0.503741				2	2	2	0.000183	33224000	3	5.7	81.178

LAMTOR1	NaN	NaN	25.1462	N/A	+			1	1	1	7.83E-28	157260000	3	7.5	17.745
LBR	NaN	NaN	22.68949	N/A				0	0	1	0.00606	6764200	1	9.4	14.743
LDHA	26.35335	NaN	NaN	N/A	+			3	2	2	3.73E-07	178640000	3	9.9	36.688
LDHB	NaN	24.78776	24.99477	0.146378				2	3	3	7.00E-10	93066000	6	15	36.638
LITAF	NaN	NaN	23.69594	N/A				1	1	1	8.68E-08	33320000	2	16.2	7.3728
LMTK3;LMTK2	NaN	NaN	23.13519	N/A				1	1	1	0.009624	26471000	2	0.5	153.66
LRRC57	NaN	NaN	24.50264	N/A	+			1	0	1	0.020468	62109000	1	5.9	26.754
LSR	25.53092	25.13273	NaN	0.281563				3	3	1	1.09E-26	120910000	6	9.7	54.498
LUC7L;LUC7L2	NaN	NaN	24.48561	N/A				1	1	1	0.001067	56968000	3	7.7	21.372
LUC7L3	25.83579	25.18945	25.48487	0.323567				2	2	2	1.02E-31	174640000	10	10.2	51.466
LYN	24.54197	NaN	24.17356	0.260505	+			2	1	2	1.98E-06	58963000	4	8.2	58.573
LZTFL1	NaN	28.60098	NaN	N/A				1	1	0	0.020728	1012000000	0	11.1	10.435
MAGI1	NaN	NaN	25.86344	N/A				1	1	1	0.005655	174020000	3	1.3	110.76
MARC2	NaN	NaN	22.32975	N/A	+			1	1	1	0.00909	12184000	3	3.3	38.023
MCCC1	26.00307	25.84062	24.87352	0.610677				4	5	4	5.14E-74	190910000	10	15.2	80.472
MGRN1	NaN	NaN	23.00828	N/A	+			1	1	1	0.002745	24833000	1	9.4	17.016
MGST2	NaN	NaN	22.32918	N/A				1	1	1	0.002167	21522000	2	22.1	8.3587
MGST3	NaN	NaN	20.93106	N/A				1	1	1	0.014769	7173300	0	7.8	14.379
MLEC	23.8447	23.55555	23.42406	0.215188				2	2	2	4.68E-09	46948000	6	14.7	23.9
MSRA	NaN	NaN	22.79931	N/A	+			1	1	1	0.001822	19820000	3	22	9.3986
NCAM1	25.40805	25.39839	25.31185	0.052973				2	2	2	1.09E-11	163770000	5	6.1	67.362
NCS1	25.51439	NaN	25.68798	0.122747	+			3	1	3	4.09E-49	152460000	6	25.8	21.878
NME2;NME1-NME	23.96746	24.11672	24.23754	0.135289				2	2	2	2.90E-08	64245000	6	12.5	17.298
NOL9	NaN	NaN	32.94677	N/A				1	1	1	0.00124	37448000000	16	2.6	79.322
NPM1	NaN	NaN	21.86903	N/A				1	1	1	0.013545	13994000	1	4.5	29.464
NRCAM	24.03011	NaN	NaN	N/A				1	0	0	0.02236	22453000	0	1	136.61
OR1D4;OR1D5	21.7737	22.20845	NaN	0.307415				1	1	1	0.00937	14772000	4	2.3	35.226
PAFAH2	NaN	NaN	21.76575	N/A	+			1	1	1	0.020044	8929400	1	9.7	18.506
PAICS	NaN	NaN	21.71364	N/A				1	1	2	3.05E-05	11981000	2	7	45.651
PASK	NaN	NaN	23.66319	N/A	+			1	1	1	0.019871	82294000	1	0.6	119.83
PC	28.77118	28.13453	28.46307	0.31838	+			11	11	11	0	1364900000	29	12.6	129.63
PCBP2	NaN	NaN	21.44091	N/A				1	1	1	0.011964	7492200	1	6	19.28
PCCA	26.44892	26.54442	26.33233	0.10622				4	4	4	1.05E-19	329890000	10	9.3	75.001
PCDHJ;DCHS2	NaN	NaN	31.28057	N/A	+			1	1	1	0.00538	11945000000	6	1.9	75.899
PCMTD2	22.98075	23.0091	23.08122	0.0518	+			2	2	2	0.000194	30488000	4	7.1	31.258
PERP	25.39454	25.80164	25.71343	0.214166				3	3	3	7.40E-44	187670000	12	21.8	21.386
PGD	NaN	NaN	19.62416	N/A	+			1	0	1	0.004688	2285700	1	8.8	17.293
PGK1	NaN	NaN	24.19393	N/A				1	1	1	7.84E-32	50896000	2	12.1	30.019
PI4K2A	24.02268	24.47389	24.08683	0.244104				3	2	2	9.28E-09	70609000	5	5	54.022
PIK3R4	NaN	21.35816	NaN	N/A	+			1	1	0	0.022193	5864800	2	0.6	153.1
PKM	24.66877	24.16231	23.97518	0.358838				3	3	3	5.67E-08	74587000	4	9.3	53.045
PLGRKT	NaN	NaN	23.56326	N/A	+			1	1	1	0.000561	34706000	3	8.8	17.201
PLP2	27.6502	26.64719	26.79848	0.540732				1	1	1	0.000441	525490000	5	14.5	16.691
PLS3	24.76415	24.61807	25.7202	0.598618				3	3	3	3.36E-18	125600000	6	7.4	65.632
PLSCR1	NaN	NaN	22.00007	N/A				1	1	1	0.009221	17813000	1	7.1	34.217
PLSCR3	NaN	NaN	21.63671	N/A				1	1	1	0.022624	31718000	1	14.4	12.18
PPIA	23.58532	24.48204	23.50252	0.543204				3	2	2	4.55E-22	60335000	5	30.9	18.012
PPIG	NaN	NaN	22.69846	N/A	+			1	1	1	0.006751	18754000	1	10.2	27.409
PPM1A	25.24659	25.48204	24.78047	0.357049	+			3	3	2	5.06E-16	144080000	6	18.5	35.957
PPM1B	25.59963	25.33146	25.59258	0.152834	+			3	4	4	5.13E-26	172700000	6	12.5	52.642

PPM1G	27.58671	27.93798	27.44174	0.255167	+		5	5	5	1.08E-73	781470000	20	12.6	59.271
PPP3R1	26.39074	26.52757	25.33695	0.651507	+		4	4	4	9.30E-76	280710000	11	36.9	18.208
PRDX2	NaN	NaN	22.54545	N/A			1	1	1	0.01068	14544000	2	8.6	21.892
PRKAB1	26.07684	26.17478	25.71442	0.242512	+		4	4	4	2.64E-13	249250000	10	14.4	30.382
PRKACA	27.6443	27.81617	28.22835	0.300152	+	+	8	9	8	5.66E-50	894610000	19	31.6	40.589
PRKACB	NaN	NaN	24.27498	N/A	+		2	2	2	6.21E-23	73174000	2	19.2	39.51
PRKDC	22.75634	23.20461	23.67088	0.4573			2	2	3	5.06E-12	34718000	5	1.2	465.38
PROCR	NaN	NaN	23.12986	N/A			1	1	1	1.59E-13	28848000	3	8	26.671
PRPF19	NaN	NaN	19.43669	N/A			0	0	1	0.008589	709620	0	1.6	55.18
PRPF38B	22.9006	NaN	NaN	N/A			2	1	1	6.85E-05	13675000	1	2.9	64.467
PSMB2	NaN	NaN	22.12278	N/A			1	1	1	0.004314	16112000	0	4	22.836
PSMC1	28.21438	28.48178	28.15282	0.174884	+		4	4	3	1.04E-31	1202700000	25	13.1	41.167
PTGFRN	NaN	NaN	24.72915	N/A	+		1	1	2	9.62E-05	43644000	2	2.8	98.555
PTPLAD1	NaN	NaN	22.94108	N/A			1	1	1	0.012546	19842000	1	3	40.069
PTPLB	NaN	NaN	19.92937	N/A			1	1	1	0.021404	3234100	0	13.1	6.8831
PVR	NaN	20.76409	NaN	N/A			1	1	0	1.46E-15	19046000	1	4.4	39.304
QKI	NaN	NaN	22.72771	N/A			1	1	1	3.33E-06	10460000	1	5.5	25.789
RAN	25.09309	25.45356	25.31174	0.181594			4	4	3	1.75E-11	148190000	8	25.9	24.423
RAP2A;RAP2C;RAP	NaN	NaN	23.20246	N/A			1	1	1	0.001964	57948000	1	22.2	12.286
RNASEK	NaN	NaN	22.48572	N/A	+		1	1	1	0.001816	20288000	2	9.7	14.9
RNF141	24.42329	26.71502	23.43135	1.684164	+		2	2	2	8.26E-19	191210000	6	16.5	25.535
RP2	27.5773	26.43229	26.70213	0.598579	+		2	3	3	7.99E-60	482360000	11	14.9	39.641
RPL10;RPL10L	NaN	NaN	21.72521	N/A	+		1	1	1	0.001287	13540000	3	11.1	12.285
RPL13	24.89657	25.31171	27.16394	1.207205			2	2	2	5.60E-39	272880000	23	43.6	8.9063
RPL13A	25.06201	25.07447	24.76056	0.177748			3	3	3	8.17E-07	119190000	9	15.3	23.577
RPL15	26.98468	27.31622	27.59676	0.306394	+		5	5	4	9.36E-17	607640000	17	27	24.146
RPL18	NaN	NaN	23.97666	N/A	+		1	1	1	0.005154	29055000	3	13.1	14.529
RPL18A	22.38774	22.74074	22.48338	0.18257			2	2	2	7.97E-05	21986000	3	16.8	16.177
RPL21	NaN	NaN	22.96453	N/A			1	1	1	0.016693	46449000	1	10	18.565
RPL23A	NaN	NaN	21.12956	N/A			1	1	1	0.002256	13789000	1	27.1	7.9232
RPL27	25.40227	25.43971	24.70504	0.413778	+		2	2	2	7.32E-20	145940000	5	26.5	15.798
RPL27A	24.91611	25.0217	23.93382	0.599934			2	2	2	5.89E-80	100960000	4	16.7	12.201
RPL28	NaN	NaN	24.94597	N/A			1	1	2	5.34E-15	72463000	3	31.9	7.888
RPL3	24.95855	24.8321	25.79949	0.525835			5	5	5	4.33E-19	142800000	7	26.1	31.243
RPL31	NaN	NaN	24.84919	N/A			1	1	1	1.60E-261	104610000	2	8.8	14.463
RPL32	NaN	NaN	27.68892	N/A			1	2	1	2.44E-60	1422300000	16	18.8	15.616
RPL36	26.13275	26.33113	26.49613	0.181945			2	2	1	7.36E-09	307440000	6	14.9	10.789
RPL37	26.18149	26.12502	27.99593	1.064244			2	2	2	1.47E-27	500150000	9	23.7	11.078
RPL37A	NaN	NaN	24.00547	N/A			1	1	1	0.001395	33551000	2	20	6.793
RPL4	NaN	NaN	24.3132	N/A			1	1	1	7.02E-08	64017000	2	10	18.818
RPL7A	NaN	23.91132	NaN	N/A			1	1	1	0.015869	43695000	2	7.9	21.545
RPLP0	NaN	NaN	22.52898	N/A			1	1	1	0.017657	20004000	1	10.3	11.057
RPS23	23.55579	22.50159	NaN	0.745432	+		1	1	1	0.015578	30604000	4	11.2	14.837
RPS26	NaN	NaN	21.60196	N/A			1	1	1	0.008141	29351000	1	17.4	13.015
RPS4X	NaN	22.50342	NaN	N/A			1	1	0	0.006734	14447000	1	4.6	29.597
RPS5	NaN	NaN	22.96469	N/A			1	1	1	1.00E-06	31435000	2	8.2	14.763
RPS9	24.64977	25.02521	24.99417	0.208379			3	2	3	1.29E-06	117520000	6	28.9	22.591
RTN2	NaN	24.33108	NaN	N/A	+		1	1	0	0.006275	54974000	0	2.9	30.102
RTN4	NaN	NaN	22.14083	N/A			1	1	1	0.023342	17248000	1	5.5	22.395
SCAMP2	24.75216	24.56808	24.98984	0.211447			3	3	2	9.42E-11	103870000	9	9.1	36.648

SCAMP3	24.20113	24.83658	24.03934	0.421419			1	1	1	8.44E-08	80835000	4	6.5	35.201
SCAMP4	24.99014	24.77455	NaN	0.152445			2	2	1	9.80E-05	85150000	4	8.7	25.728
SCARB2	26.2265	26.4403	25.72656	0.366305	+		2	3	3	1.28E-38	271670000	5	7.5	54.29
SERINC1	22.28757	24.56558	22.89262	1.179987	+		2	2	2	5.60E-09	45281000	3	3.5	50.494
SERPINB11	NaN	NaN	25.32855	N/A			1	1	1	0.023579	118660000	3	3.7	24.548
SERPINB7	NaN	NaN	26.2999	N/A			1	1	1	0.01854	346220000	0	2.2	41.174
SHMT2	NaN	NaN	21.22359	N/A	+		1	1	1	0.023196	6939600	0	2.9	44.616
SLC1A5	NaN	NaN	26.40492	N/A			2	2	2	1.09E-17	660990000	6	8	33.709
SLC25A5	25.57914	25.61807	24.68143	0.529889	+		6	6	5	8.46E-13	156360000	10	21.8	32.852
SLC35B2	NaN	NaN	22.7043	N/A			1	1	1	0.002808	21044000	1	3.7	32.663
SLC38A1	24.48148	24.53985	24.33946	0.103064			2	2	2	0.00029	87206000	3	4.1	54.047
SLC44A1	23.91834	24.21593	23.88176	0.183288	+		3	2	3	1.38E-06	61932000	4	4.9	73.011
SLC44A2	NaN	NaN	20.75768	N/A	+		1	1	1	6.29E-07	19335000	3	2.6	79.845
SLC7A1	23.79828	23.6936	23.49644	0.153263	+		3	3	2	4.68E-12	48493000	4	9.4	67.638
SLC9A6	NaN	NaN	22.70561	N/A			1	1	1	0.007058	18938000	2	1.5	72.259
SNRNP70	NaN	22.22911	NaN	N/A			1	1	0	0.02026	25024000	1	10.2	19.79
SRC	26.3123	25.90571	26.7571	0.425838	+		5	6	5	2.55E-53	310930000	12	15.1	59.834
SRP68	NaN	NaN	21.05881	N/A	+		1	0	1	6.32E-05	6703600	1	4.6	60.284
SRRM1	NaN	NaN	21.8506	N/A			1	1	1	0.014439	22544000	2	10.9	11.721
SRRM2	26.26012	26.04441	26.94827	0.47206			6	6	7	9.43E-63	320730000	13	4.2	256.64
SRSF4	NaN	NaN	22.54651	N/A			1	0	1	0.00015	13928000	2	4.3	56.678
SRSF6	24.89009	25.06604	25.04293	0.095614			3	3	3	9.25E-13	118810000	5	10.7	38.418
SRSF7	NaN	NaN	22.01859	N/A			1	1	1	0.001667	22513000	3	7.6	15.257
STAT2	NaN	NaN	23.76162	N/A			1	1	1	0.021418	45374000	1	2	52.998
STX12	NaN	NaN	22.94542	N/A			1	1	1	4.12E-06	27052000	3	6	24.576
STX7	NaN	NaN	23.31119	N/A			1	1	1	0.007469	39178000	1	4.6	27.4
TAGLN2	22.09558	23.89883	NaN	1.27509			1	1	1	0.000314	29893000	3	9.6	21.086
TESC	NaN	22.06313	NaN	N/A	+		0	1	0	0.000279	5610500	1	14.1	15.292
TFRC	27.42598	27.29509	27.58842	0.146948			8	8	8	4.22E-66	652970000	18	13.7	75.963
TKT	22.91903	22.92548	23.37862	0.263502			2	2	2	4.76E-07	31714000	3	8.1	36.449
TM9SF3	NaN	NaN	22.71406	N/A			1	0	1	0.000393	11621000	1	5.1	29.881
TMEM106B	NaN	NaN	24.09852	N/A	+		1	1	1	0.000488	78144000	4	4.4	31.127
TMEM147	NaN	NaN	21.4208	N/A			1	1	1	0.002148	6782800	0	4.6	19.543
TMEM168	NaN	NaN	21.88517	N/A			1	0	1	0.010511	8689000	1	5.4	29.105
TMEM184C	NaN	23.53315	NaN	N/A			1	1	0	0.011865	27471000	1	5.4	30.521
TMEM192	24.61122	22.97532	22.47311	1.118026			2	2	2	5.51E-14	49944000	3	13.1	30.549
TMEM33	NaN	NaN	21.82361	N/A			1	0	1	0.019789	6589700	2	7.7	17.806
TMEM50A	25.45497	25.41212	25.53386	0.061753			4	4	4	3.60E-16	163760000	10	33.1	17.4
TMEM63B	22.9771	22.84433	23.58955	0.397508			2	2	2	2.80E-06	33914000	3	6.9	56.307
TMEM97	NaN	NaN	23.09708	N/A			1	1	1	0.001881	32674000	3	14.5	8.2187
TMX1	22.93832	22.85377	22.80182	0.068896			3	2	2	1.09E-10	27175000	5	8.2	31.791
TPI1	24.92592	24.81827	25.12892	0.157745			3	3	3	1.36E-07	118520000	6	20.9	26.669
TRAPPC3	24.64169	24.83899	25.21148	0.289349	+		2	2	2	7.85E-17	112430000	5	27.6	15.005
TRIM28	NaN	NaN	21.88699	N/A			1	1	1	3.44E-07	7188800	1	13.6	18.098
TRPV3	NaN	NaN	24.00359	N/A			1	1	1	0.021858	60082000	1	2	87.66
TSPAN3	26.77731	26.04529	26.14194	0.397679	+		1	1	1	6.44E-16	312530000	7	8.5	21.183
TSPAN31;TSPAN13	NaN	NaN	25.19933	N/A			0	0	1	0.011834	38526000	1	11.1	13.317
TSPAN6	NaN	26.24231	26.4125	0.120343			2	4	3	1.54E-42	246130000	6	16.7	27.563
TTYH3	24.25205	23.99826	24.67293	0.340766			2	2	2	3.62E-12	73501000	3	11.4	38.486
TUBA1B	NaN	NaN	23.59537	N/A			1	1	1	4.07E-50	39286000	1	36.2	27.465

TUBA1C;TUBA1A;T	28.20088	28.03721	28.25291	0.112562	+			7	7	7	1.62E-48	1087800000	17	21.4	49.895
TUBB	25.67489	26.09287	24.88744	0.612079				2	2	2	5.47E-90	191640000	8	18.5	47.766
TUBB4B;TUBB4A	27.28056	26.99906	27.30077	0.168661				8	8	8	5.94E-46	554220000	23	22.2	49.83
TXNL1	NaN	NaN	23.72578	N/A				0	1	1	0.010541	28542000	1	5.4	18.934
UBA1	24.19551	24.29691	24.26935	0.052431	+			3	3	3	4.42E-11	72797000	6	3.6	117.85
UBC;UBA52;UBB;R	NaN	NaN	23.67949	N/A				1	1	1	0.00014	47169000	2	23	6.8758
UCHL1	24.40542	23.81294	24.14336	0.296897				2	3	3	2.56E-06	65246000	5	27.4	24.824
UQCRQ	NaN	NaN	23.48382	N/A	+			2	1	2	6.45E-05	29462000	2	26.8	9.9062
VAMP2;VAMP3	NaN	NaN	24.47785	N/A				1	1	1	1.97E-23	196290000	6	45.6	7.7752
VAMP7	24.19205	24.69285	24.06052	0.333652				2	2	2	1.60E-10	77309000	4	10.9	24.935
VDAC2	25.08331	24.64213	25.00599	0.235589				2	2	2	1.96E-18	111870000	5	18.6	22.159
XPO1	NaN	NaN	22.22202	N/A				1	1	1	1.83E-13	19352000	0	0.8	123.38
XRCC5	NaN	21.27145	22.74061	1.038853				2	2	2	0.000297	12730000	3	3.1	82.704
XRCC6	24.85519	25.231	NaN	0.265738				3	4	3	7.22E-14	113050000	4	11.3	64.283
YES1	25.05528	24.29593	23.8319	0.617602	+			2	2	2	2.38E-19	88394000	5	10.7	60.801
YWHAB	NaN	NaN	21.64548	N/A				1	1	1	0.001075	27258000	1	9.4	27.85
YWHAZ	25.98203	25.51265	26.18299	0.34401				3	3	3	1.41E-15	226590000	5	21.4	19.072

Table S4 : Identification of PTM peptides in Hek293, HeLa and MCF7 cells with MaxQuant and PEAKS

Sheet 1 : Summary of all detected PTM peptides
The table shows all proteins (gene names) for which a PTM peptide was detected as well as the number of appearences within a triplicate per cell line.

Sheets 2-4 : Total number of PTM peptide sequences per cell line (MQ)
A complete list of PTM peptides found in all replicates and their characterists, e.g. charge, retention time, score, delta score.
PTM peptides are ordered by their sequences.

Sheets 5-7 : Total number of PTM peptide sequences per cell line (PEAKS)
A complete list of PTM peptides found in all replicates and their characterists, e.g. probablity score (-10LogP), mass, retention time.
PTM peptides are ordered by their sequences,

In red - PTM peptides assigned as false positive (non-MG) or found in both YnMyr and Myr control
The false discovery rate (#non-MG PTM peptides/#MG PTM peptides + #non-MG PTM peptides) is **1%**.
Note: Interestingly two peptides from this data set, detected by both software packages, corresponded to Lypla1 and Lypla2, which contain *N* -terminal cysteine (rather than glycine) that was recently reported as palmitoylated (Kong et al., *J Biol Chem* **2013**, *288* , 9112). A possible explanation for this phenomenon is that YnMyr was transferred by palmitoyl transferases to the *N* -terminal cysteine of Lypla proteins, followed by an intramolecular S->N rearrangement to form an amide bond, which occurred either in situ or in vitro.

	Proteins with PTM peptide		PTM peptide seen within triplicate					
			MQ			PEAKS		
#	Gene names MQ	Gene names PEAKS	Hek	HeLa	MCF	Hek	HeLa	MCF
1	ABL1					3	3	3
2	AKAP12	AKA12	3	3		3	3	
3	ANKIB1	AKIB1		1			1	1
4	AP1AR				1	1	2	1
5	ARF1		3	3	3	3		3
6	ARF3		3	3	3	3	3	3
7	ARF4		3	3	3	3	3	3
8	ARF5		2	3	3	3	3	3
9	ARL5B					1	2	
10	AVPI1			2				
11	BCAS1				3			2
12	C8orf47	ERIC5	1			2		
13	CCNY					2		
14	CCNYL1	CCYL1				2		
15	CHCHD3	CHCH3				2	3	2
16	CHCHD6	CHCH6	3			3		
17	CHMP6			1	1	1	1	
18	CLN3		2			3	1	
19	DCUN1D3	DCNL3				1	1	2
20	DUSP23	DUS23					1	
21	DYM					1	1	1
22	EEPD1		3			3		
23	FAM129A	NIBAN	3	3		3	3	
24	FAM129B	NIBL1	3	3	3	3	3	3
25	FAM49B	FA49B				1	1	
26	FAM84B	FA84B			1	3		3
27	FKBP8			2				
28	FMNL1			2				
29	FMNL2		2	1		2	1	
30	FMNL3	F8W1F5					1	
31	FYN		1			3		
32	GNAI1, GNAI3		3	3	3	3	3	3
33	GNAI2		2	3	3	3	3	3
34	GNAO1			3			3	
35	GORASP2	GORS2	3	3	3	3	3	3
36	GREB1				3			3
37	HCCS	CCHL	2	3	1	3	3	1
38	HID1					1		3
39	LAMTOR1	LTOR1	3	3	3	3	3	3
40	LANCL2	LANC2					1	
41	LNP			1				1
42	LOH12CR1	L12R1		3	2		3	2
43	LRRC57	LRC57	1			1	1	1
44	LYPLA1	LYPA1		2	2	3	3	3
45	LYPLA2	LYPA2	1	3		3	3	1
46	MARC1			3			2	

47	MARC2		3	3	3	3	2	3
48	MARCKS	MARCS		1		3	3	3
49	MARCKSL1	MRP	1	2		2	2	1
50	MGRN1						1	1
51	MSRA		3	3	3	2	3	3
52	NDUFB7	NDUB7				3	3	3
53	NOL3			3	3		2	3
54	OGFRL1	OGRL1				2	2	
55	P2RX5						2	
56	PAFAH2	PAFA2					1	
57	PCMTD1	PCMD1	3	3	3	3	3	2
58	PCMTD2	PCMD2	3	2	2	3	3	3
59	PDE8A						2	
60	PHACTR2		3			3		
61	PLGRKT	PLRKT				2	2	1
62	POLR2A	RPB1						1
63	PPM1A, PPM1B		3	3	2	3	3	3
64	PPM1G		3	3	3	3	3	3
65	PPP3R1	CANB1	3	3	3	3	3	3
66	PRKAB1	AAKB1				2		
67	PRKACA	KAPCA	2	3	2	2	3	3
68	PRKACB	KAPCB	1	1		3	2	3
69	PSMC1	PRS4	3	3	3	3	3	3
70	RFTN1		1	2	1	2	2	3
71	RNF141	RN141	3	3	2	3	3	3
72	RP2	XRP2	1			3	3	
73	SAMM50	SAM50	3		1	3		3
74	SCYL3	X6RHX1	3	1	1	1		
75	SLC44A1	CTL1	3	2	3	3	1	2
76	SPECC1	CYTSB	3	2	3	3	2	2
77	SVIP					3	2	
78	TACC1		3	3		1	3	
79	TESC	CHP3	3	3	1	3	3	
80	TIAM1		1			1		1
81	TMEM106C	T106C	3	3	3	2	1	2
82	TOMM40L	TM40L	1	3		2	3	
83	TUSC2			1				
84	VKORC1	VKOR1		2			2	
85	ZDHHC6	ZDHC6					1	

HEK293 MQ

Replicate	Sequence	Gene Names	Protein Names	Charge	Length	m/z	Retention time	PEP	MS/MS Count	Score	Delta score	Intensity
Hek_1	CGNTMSVPLLTDAATVSGAER	LYPLA2	Acyl-protein thioesterase 2	3	21	871.7756	84.153	0.011375	1	34.217	32.809	2446400
Hek_1	GAAHSASEEVR	TESC	Calcineurin B homologous protein 3	3	11	526.2779	45.889	4.07E-05	1	108.47	91.239	17228000
Hek_2	GAAHSASEEVR	TESC	Calcineurin B homologous protein 3	3	11	526.2779	45.908	4.00E-07	1	138.22	115.1	18731000
Hek_3	GAAHSASEEVR	TESC	Calcineurin B homologous protein 3	3	11	526.2779	45.867	6.39E-07	1	126.39	107.72	9741700
Hek_1	GAFLDKPK	PPM1A;PPM1B	Protein phosphatase 1A;Protein phosphatase 1B	3	8	446.9346	67.685	0.071705	2	80.438	54.054	104950000
Hek_2	GAFLDKPK	PPM1A;PPM1B	Protein phosphatase 1A;Protein phosphatase 1B	3	8	446.9346	67.559	0.071872	2	77.923	58.146	87965000
Hek_3	GAFLDKPK	PPM1A;PPM1B	Protein phosphatase 1A;Protein phosphatase 1B	3	8	446.9346	67.473	0.071967	1	76.478	56.701	67222000
Hek_1	GAGSSTEQR	AKAP12	A-kinase anchor protein 12	2	9	678.3549	51.679	0.01281	1	90.15	50.932	32715000
Hek_1	GAGSSTEQR	AKAP12	A-kinase anchor protein 12	3	9	452.5724	51.656	0.006116	1	91.867	75.713	43442000
Hek_2	GAGSSTEQR	AKAP12	A-kinase anchor protein 12	3	9	452.5724	51.659	0.007924	1	84.615	68.461	36218000
Hek_2	GAGSSTEQR	AKAP12	A-kinase anchor protein 12	2	9	678.3549	51.669	0.028191	1	64.82	38.993	33741000
Hek_3	GAGSSTEQR	AKAP12	A-kinase anchor protein 12	3	9	452.5724	51.626	0.007954	1	82.645	68.973	27505000
Hek_1	GASSSSALAR	MARC2	MOSC domain-containing protein 2, mitochondrial	3	10	457.2564	58.709	0.001792	1	76.228	58.408	26251000
Hek_2	GASSSSALAR	MARC2	MOSC domain-containing protein 2, mitochondrial	3	10	457.2564	58.602	0.000572	1	96.143	78.323	25297000
Hek_2	GASSSSALAR	MARC2	MOSC domain-containing protein 2, mitochondrial	2	10	685.381	58.595	0.004577	1	71.03	47.058	17606000
Hek_3	GASSSSALAR	MARC2	MOSC domain-containing protein 2, mitochondrial	3	10	457.2564	58.533	0.000585	1	109.02	83.494	23358000
Hek_1	GAYLSQPNTVK	PPM1G	Protein phosphatase 1G	2	11	820.9596	72.121	6.15E-07	1	153.74	122.32	35956000
Hek_2	GAYLSQPNTVK	PPM1G	Protein phosphatase 1G	3	11	547.6421	72.029	0.00739	1	53.08	33.422	159640000
Hek_2	GAYLSQPNTVK	PPM1G	Protein phosphatase 1G	2	11	820.9596	72.027	9.98E-07	1	145.14	115.6	35684000
Hek_3	GAYLSQPNTVK	PPM1G	Protein phosphatase 1G	2	11	820.9596	72.005	8.00E-07	1	138.99	111.08	22682000
Hek_1	GCCSSASSAAQSSK	SLC44A1	Choline transporter-like protein 1	3	14	617.621	50.762	0.002534	1	64.842	60.314	20111000
Hek_2	GCCSSASSAAQSSK	SLC44A1	Choline transporter-like protein 1	3	14	617.621	50.658	0.002383	1	61.657	60.033	24265000
Hek_3	GCCSSASSAAQSSK	SLC44A1	Choline transporter-like protein 1	3	14	617.621	50.607	0.004714	1	46.68	45.005	18994000
Hek_1	GCCYSSENEDSDQDREER	LAMTOR1	Ragulator complex protein LAMTOR1	4	18	675.5319	51.076	1.50E-06	1	92.439	89.105	73033000
Hek_1	GCCYSSENEDSDQDREER	LAMTOR1	Ragulator complex protein LAMTOR1	3	18	900.3734	51.073	5.06E-35	1	149.73	131.5	85783000
Hek_2	GCCYSSENEDSDQDREER	LAMTOR1	Ragulator complex protein LAMTOR1	3	18	900.3734	50.959	1.88E-06	1	100.58	87.153	83386000
Hek_2	GCCYSSENEDSDQDREER	LAMTOR1	Ragulator complex protein LAMTOR1	4	18	675.5319	50.967	2.07E-06	1	77.506	73.385	73291000
Hek_3	GCCYSSENEDSDQDREER	LAMTOR1	Ragulator complex protein LAMTOR1	4	18	675.5319	50.957	5.08E-11	1	117.18	113.84	49232000
Hek_3	GCCYSSENEDSDQDREER	LAMTOR1	Ragulator complex protein LAMTOR1	3	18	900.3734	50.956	6.71E-06	1	90.379	83.531	52784000
Hek_2	GCFFSK	RP2	Protein XRP2	2	6	604.8159	80.885	0.072807	1	106.76	84.075	22367000
Hek_2	GCGLNK	RFTN1	Raftlin	2	6	556.3057	58.975	0.08065	1	107.73	39.968	7610800
Hek_1	GCSSSALNK	C8orf47	Uncharacterized protein C8orf47	3	9	462.9101	56.343	0.083379	1	44.98	34.843	9097900
Hek_1	GCSSSALNK	C8orf47	Uncharacterized protein C8orf47	2	9	693.8615	56.353	0.015607	1	76.064	56.278	4328400
Hek_1	GCTLSAEDK	GNAI3;GNAI1	Guanine nucleotide-binding protein G(k) subunit alpha;G	2	9	722.3667	63.556	0.00012	1	168.83	121.53	187590000
Hek_1	GCTLSAEDK	GNAI3;GNAI1	Guanine nucleotide-binding protein G(k) subunit alpha;G	3	9	481.9135	63.56	0.007976	1	81.263	66.252	58606000
Hek_2	GCTLSAEDK	GNAI3;GNAI1	Guanine nucleotide-binding protein G(k) subunit alpha;G	2	9	722.3667	63.491	0.008618	1	147.2	90.795	140300000
Hek_2	GCTLSAEDK	GNAI3;GNAI1	Guanine nucleotide-binding protein G(k) subunit alpha;G	3	9	481.9135	63.49	0.01105	1	70.399	57.362	48867000
Hek_3	GCTLSAEDK	GNAI3;GNAI1	Guanine nucleotide-binding protein G(k) subunit alpha;G	2	9	722.3667	63.392	0.000656	2	163.64	116.34	91699000
Hek_3	GCTLSAEDK	GNAI3;GNAI1	Guanine nucleotide-binding protein G(k) subunit alpha;G	3	9	481.9135	63.392	0.007292	1	87.806	72.869	29544000
Hek_1	GCTVSAEDK	GNAI2	Guanine nucleotide-binding protein G(i) subunit alpha-2	3	9	477.2416	58.835	0.019349	1	61.78	36.158	29620000
Hek_3	GCTVSAEDK	GNAI2	Guanine nucleotide-binding protein G(i) subunit alpha-2	2	9	715.3588	58.691	0.007338	1	101.66	63.097	80068000
Hek_3	GCTVSAEDK	GNAI2	Guanine nucleotide-binding protein G(i) subunit alpha-2	3	9	477.2416	58.686	0.007889	1	85.744	32.877	18793000
Hek_2	GCVQCK	FYN	Tyrosine-protein kinase Fyn	2	6	607.8103	56.785	0.081964	1	104.03	36.001	25756000
Hek_1	GDVLSTHLDDAR	FAM129B	Niban-like protein 1	3	12	587.9796	70.272	2.56E-11	2	142.1	126.11	52682000
Hek_2	GDVLSTHLDDAR	FAM129B	Niban-like protein 1	3	12	587.9796	70.247	2.89E-12	2	158.01	139.88	52534000
Hek_3	GDVLSTHLDDAR	FAM129B	Niban-like protein 1	3	12	587.9796	70.097	4.67E-09	2	118.87	103.72	36494000

Hek_1	GGAVSAGEDNDDLIDNLK	PCMTD1	Protein-L-isoaspartate O-methyltransferase domain-cont	3	18	756.0485	77.673	5.89E-06	1	72.583	69.825	7612200
Hek_2	GGAVSAGEDNDDLIDNLK	PCMTD1	Protein-L-isoaspartate O-methyltransferase domain-cont	3	18	756.0485	77.682	6.54E-05	1	61.127	58.159	8327900
Hek_3	GGAVSAGEDNDDLIDNLK	PCMTD1	Protein-L-isoaspartate O-methyltransferase domain-cont	3	18	756.0485	77.616	0.00189	1	45.618	40.244	5753300
Hek_1	GGAVSAGEDNDELIDNLK	PCMTD2	Protein-L-isoaspartate O-methyltransferase domain-cont	3	18	760.7204	76.376	7.75E-05	1	60.307	58.517	55608000
Hek_2	GGAVSAGEDNDELIDNLK	PCMTD2	Protein-L-isoaspartate O-methyltransferase domain-cont	3	18	760.7204	76.34	3.87E-06	1	80.361	77.56	51240000
Hek_3	GGAVSAGEDNDELIDNLK	PCMTD2	Protein-L-isoaspartate O-methyltransferase domain-cont	3	18	760.7204	76.231	0.000694	2	51.495	49.109	37741000
Hek_1	GGCAGSR	CLN3	Battenin	2	7	564.2906	52.425	0.034241	1	108.24	41.244	13147000
Hek_3	GGCAGSR	CLN3	Battenin	2	7	564.2906	52.329	0.032171	1	110.63	38.684	9413100
Hek_1	GGSASSQLDEGK	FAM129A	Protein Niban	3	12	533.6093	57.52	8.17E-05	1	99.5	72.714	28965000
Hek_1	GGSASSQLDEGK	FAM129A	Protein Niban	2	12	799.9103	57.507	3.69E-11	1	133.32	122.21	8769100
Hek_2	GGSASSQLDEGK	FAM129A	Protein Niban	3	12	533.6093	57.518	0.000566	1	67.749	45.722	27680000
Hek_2	GGSASSQLDEGK	FAM129A	Protein Niban	2	12	799.9103	57.526	0.000372	1	81.278	69.71	7206600
Hek_3	GGSASSQLDEGK	FAM129A	Protein Niban	3	12	533.6093	57.46	1.80E-09	1	124.39	102.36	19479000
Hek_3	GGSASSQLDEGK	FAM129A	Protein Niban	2	12	799.9103	57.454	0.000226	1	84.365	74.71	7067900
Hek_1	GGSHSQTPR	TACC1	Transforming acidic coiled-coil-containing protein 1	3	9	463.9164	39.465	0.005105	1	95.358	50.605	4460200
Hek_2	GGSHSQTPR	TACC1	Transforming acidic coiled-coil-containing protein 1	3	9	463.9164	39.378	0.008022	1	78.264	38.959	5126300
Hek_3	GGSHSQTPR	TACC1	Transforming acidic coiled-coil-containing protein 1	3	9	463.9164	39.338	0.004578	1	97.635	57.283	2596300
Hek_1	GLSPSAPAVAVQASNASASPPSGCPMHEGK	HCCS	Cytochrome c-type heme lyase	4	30	832.1659	63.438	3.72E-14	1	72.231	71.321	38229000
Hek_3	GLSPSAPAVAVQASNASASPPSGCPMHEGK	HCCS	Cytochrome c-type heme lyase	4	30	832.1659	63.168	9.59E-14	1	67.641	66.957	23416000
Hek_1	GLTISSLFSR	ARF4	ADP-ribosylation factor 4	3	10	515.3033	96.25	0.001875	2	74.841	65.883	25527000
Hek_2	GLTISSLFSR	ARF4	ADP-ribosylation factor 4	3	10	515.3033	96.178	0.001479	1	83.869	69.195	20679000
Hek_3	GLTISSLFSR	ARF4	ADP-ribosylation factor 4	3	10	515.3033	96.094	0.000568	1	96.342	69.556	39779000
Hek_3	GLTISSLFSR	ARF4	ADP-ribosylation factor 4	2	10	772.4514	96.091	0.003199	1	79.659	55.629	14708000
Hek_1	GLTVSALFSR	ARF5	ADP-ribosylation factor 5	3	10	505.2998	93.507	0.022453	1	64.82	48.682	3266000
Hek_3	GLTVSALFSR	ARF5	ADP-ribosylation factor 5	3	10	505.2998	93.396	0.001609	2	80.706	56.617	9823500
Hek_1	GNAAAAK	PRKACA	cAMP-dependent protein kinase catalytic subunit alpha	2	7	533.3118	52.708	0.03585	2	134.66	86.562	229320000
Hek_2	GNAAAAK	PRKACA	cAMP-dependent protein kinase catalytic subunit alpha	2	7	533.3118	52.659	0.037039	1	124.34	74.033	235840000
Hek_2	GNAATAK	PRKACB	cAMP-dependent protein kinase catalytic subunit beta	2	7	548.3171	52.65	0.029435	1	119.29	60.63	57105000
Hek_2	GNAESQHVEHEFYGEK	TIAM1	T-lymphoma invasion and metastasis-inducing protein 1	4	16	581.7818	52.022	0.000336	1	54.768	52.505	12321000
Hek_1	GNAGSMDSQQTDFR	FMNL2	Formin-like protein 2	3	14	659.6463	63.288	0.002356	1	66.246	59.764	5544000
Hek_2	GNAGSMDSQQTDFR	FMNL2	Formin-like protein 2	3	14	659.6463	63.211	0.001277	1	74.789	71.455	4611200
Hek_1	GNEASYPLEMCSHFDADEIKR	PPP3R1	Calcineurin subunit B type 1	4	21	737.8471	58.884	1.49E-14	1	114.75	108.51	30837000
Hek_2	GNEASYPLEMCSHFDADEIKR	PPP3R1	Calcineurin subunit B type 1	4	21	737.8471	58.843	2.36E-11	1	96.848	94.47	33540000
Hek_3	GNEASYPLEMCSHFDADEIKR	PPP3R1	Calcineurin subunit B type 1	4	21	737.8471	58.671	0.002003	1	34.65	33.47	17462000
Hek_1	GNEASYPLEMCSHFDADEIKR	PPP3R1	Calcineurin subunit B type 1	4	21	733.8484	63.694	2.15E-18	1	120.23	117.73	107310000
Hek_2	GNEASYPLEMCSHFDADEIKR	PPP3R1	Calcineurin subunit B type 1	4	21	733.8484	63.805	2.31E-19	1	127.01	119.91	152680000
Hek_3	GNEASYPLEMCSHFDADEIKR	PPP3R1	Calcineurin subunit B type 1	4	21	733.8484	63.674	1.20E-18	1	123.6	116.33	99206000
Hek_1	GNHSGRPEDPEPGAFTTTK	SPECC1	Cytospin-B	4	19	616.0609	45.88	1.18E-06	1	84.66	74.813	13943000
Hek_2	GNHSGRPEDPEPGAFTTTK	SPECC1	Cytospin-B	4	19	616.0609	45.861	4.06E-07	1	96.238	92.195	18317000
Hek_3	GNHSGRPEDPEPGAFTTTK	SPECC1	Cytospin-B	4	19	616.0609	45.909	1.17E-10	1	116.73	104.35	12000000
Hek_1	GNIFANLFK	ARF1	ADP-ribosylation factor 1	3	9	496.2891	101.25	0.061851	1	48.794	37.63	9590300
Hek_1	GNIFANLFK	ARF1	ADP-ribosylation factor 1	2	9	743.9301	101.35	0.01537	2	83.948	59.918	14544000
Hek_1	GNIFANLFK	ARF1	ADP-ribosylation factor 1	3	9	496.2891	101.4	0.023354	1	59.35	44.676	16540000
Hek_2	GNIFANLFK	ARF1	ADP-ribosylation factor 1	2	9	743.9301	101.28	0.028323	2	64.711	38.495	11369000
Hek_2	GNIFANLFK	ARF1	ADP-ribosylation factor 1	3	9	496.2891	101.28	0.023354	1	59.35	48.185	15687000
Hek_3	GNIFANLFK	ARF1	ADP-ribosylation factor 1	3	9	496.2891	101.22	0.008019	2	78.516	50.342	56324000
Hek_3	GNIFANLFK	ARF1	ADP-ribosylation factor 1	2	9	743.9301	101.22	0.002109	1	138.98	98.624	50287000
Hek_1	GNIFGNLLK	ARF3	ADP-ribosylation factor 3	2	9	719.9301	94.599	0.002381	1	128.38	78.343	19012000
Hek_2	GNIFGNLLK	ARF3	ADP-ribosylation factor 3	2	9	719.9301	94.562	0.005109	1	107.57	66.126	27016000
Hek_3	GNIFGNLLK	ARF3	ADP-ribosylation factor 3	2	9	719.9301	94.472	0.002286	1	126.07	80.7	35201000

Hek_3	GNIFGNLLK	ARF3	ADP-ribosylation factor 3	3	9	480.2891	94.473	0.010238	1	71.692	42.996	21676000
Hek_1	GNSALR	LRRC57	Leucine-rich repeat-containing protein 57	2	6	540.8173	59.382	0.066824	1	154.09	51.115	65274000
Hek_1	GNSASNIVSPQEALPGR	MSRA	Mitochondrial peptide methionine sulfoxide reductase	3	17	720.7222	67.946	7.65E-08	1	116.06	109.94	13200000
Hek_2	GNSASNIVSPQEALPGR	MSRA	Mitochondrial peptide methionine sulfoxide reductase	3	17	720.7222	67.998	0.000152	1	73.168	69.105	10232000
Hek_3	GNSASNIVSPQEALPGR	MSRA	Mitochondrial peptide methionine sulfoxide reductase	3	17	720.7222	67.876	5.14E-07	1	109.04	102.92	11655000
Hek_3	GNTLGLAPMGTLPR	TOMM40L	Mitochondrial import receptor subunit TOM40B	3	14	621.0207	81.994	0.015268	1	40.278	40.278	3279300
Hek_1	GQQISDQTQLVINK	RNF141	RING finger protein 141	2	14	1018.068	70.081	0.004859	1	65.043	54.69	2897100
Hek_2	GQQISDQTQLVINK	RNF141	RING finger protein 141	2	14	1018.068	70.087	0.00074	1	101.3	80.719	4806800
Hek_3	GQQISDQTQLVINK	RNF141	RING finger protein 141	3	14	679.048	69.967	0.00097	1	79.875	58.524	57770000
Hek_3	GQQISDQTQLVINK	RNF141	RING finger protein 141	2	14	1018.068	69.953	0.003913	1	55.724	43.666	4343800
Hek_1	GQSQSGGHGPGGGK	PSMC1	26S protease regulatory subunit 4	3	14	558.6204	38.934	5.64E-16	1	138.21	129.73	259720000
Hek_2	GQSQSGGHGPGGGK	PSMC1	26S protease regulatory subunit 4	3	14	558.6204	38.814	2.93E-10	2	127.32	127.32	188170000
Hek_3	GQSQSGGHGPGGGK	PSMC1	26S protease regulatory subunit 4	3	14	558.6204	38.801	0.00056	1	98.654	98.654	212900000
Hek_2	GQSQSGGHGPGGGKK	PSMC1	26S protease regulatory subunit 4	4	15	451.2409	30.887	0.000725	3	48.527	35.127	18264000
Hek_1	GQTSVSTLSPQPGSVDGLDK	PHACTR2	Phosphatase and actin regulator 2	3	20	812.7625	69.577	1.97E-10	1	97.57	91.454	9874000
Hek_2	GQTSVSTLSPQPGSVDGLDK	PHACTR2	Phosphatase and actin regulator 2	3	20	812.7625	69.57	1.57E-09	1	85.737	78.028	7386700
Hek_3	GQTSVSTLSPQPGSVDGLDK	PHACTR2	Phosphatase and actin regulator 2	3	20	812.7625	69.512	1.01E-10	1	102.07	94.628	6026200
Hek_1	GSENSALK	SCYL3	Protein-associating with the carboxyl-terminal domain o	2	8	634.8515	57.269	0.022925	1	104.2	54.839	13595000
Hek_2	GSENSALK	SCYL3	Protein-associating with the carboxyl-terminal domain o	2	8	634.8515	57.272	0.049525	1	94.309	46.068	13987000
Hek_3	GSENSALK	SCYL3	Protein-associating with the carboxyl-terminal domain o	2	8	634.8515	57.194	0.065164	1	82.287	45.7	6706400
Hek_1	GSQHSAAAR	TMEM106C	Transmembrane protein 106C	3	9	449.9129	38.881	0.011693	1	69.375	56.327	11764000
Hek_2	GSQHSAAAR	TMEM106C	Transmembrane protein 106C	3	9	449.9129	38.716	0.016682	1	63.397	47.516	9532500
Hek_3	GSQHSAAAR	TMEM106C	Transmembrane protein 106C	3	9	449.9129	38.697	0.001129	1	124.81	105.16	10633000
Hek_2	GSQSSK	MARCKSL1	MARCKS-related protein	2	6	528.7935	48.468	0.012882	1	163.34	69.178	172360000
Hek_1	GSSQSVEIPGGGTEGYHVLR	GORASP2	Golgi reassembly-stacking protein 2	3	20	831.7663	62.149	4.96E-19	1	127.59	119.09	258140000
Hek_1	GSSQSVEIPGGGTEGYHVLR	GORASP2	Golgi reassembly-stacking protein 2	4	20	624.0766	62.149	1.96E-13	1	107.41	102.11	151850000
Hek_2	GSSQSVEIPGGGTEGYHVLR	GORASP2	Golgi reassembly-stacking protein 2	3	20	831.7663	62.058	1.26E-17	1	121.82	116.85	224460000
Hek_2	GSSQSVEIPGGGTEGYHVLR	GORASP2	Golgi reassembly-stacking protein 2	4	20	624.0766	62.058	4.72E-18	1	121.82	117.6	136220000
Hek_3	GSSQSVEIPGGGTEGYHVLR	GORASP2	Golgi reassembly-stacking protein 2	3	20	831.7663	62.028	6.77E-32	2	144.72	137.07	137600000
Hek_3	GSSQSVEIPGGGTEGYHVLR	GORASP2	Golgi reassembly-stacking protein 2	4	20	624.0766	62.03	6.19E-11	1	99.092	92.318	76303000
Hek_1	GSTESSEGR	CHCHD6	Coiled-coil-helix-coiled-coil-helix domain-containing prot	2	9	686.8444	53.037	0.076829	1	52.725	30.488	28169000
Hek_2	GSTESSEGR	CHCHD6	Coiled-coil-helix-coiled-coil-helix domain-containing prot	2	9	686.8444	53.044	0.023646	1	68.557	36.203	23162000
Hek_3	GSTESSEGR	CHCHD6	Coiled-coil-helix-coiled-coil-helix domain-containing prot	2	9	686.8444	52.9	0.025637	1	66.92	48.131	29447000
Hek_3	GSTESSEGR	CHCHD6	Coiled-coil-helix-coiled-coil-helix domain-containing prot	3	9	458.232	52.905	0.007939	1	83.647	51.292	19229000
Hek_1	GSTLGCHR	EEPD1	Endonuclease/exonuclease/phosphatase family domain-	3	8	450.9068	52.826	0.025745	1	94.114	71.223	8271700
Hek_2	GSTLGCHR	EEPD1	Endonuclease/exonuclease/phosphatase family domain-	3	8	450.9068	52.752	0.023829	1	96.19	70.74	7918600
Hek_3	GSTLGCHR	EEPD1	Endonuclease/exonuclease/phosphatase family domain-	3	8	450.9068	52.656	0.024088	1	95.909	77.218	8571000
Hek_1	GTVHAR	SAMM50	Sorting and assembly machinery component 50 homolog	3	6	368.5526	46.5	0.053519	1	97.64	44.119	14816000
Hek_2	GTVHAR	SAMM50	Sorting and assembly machinery component 50 homolog	3	6	368.5526	46.397	0.039884	1	105.42	45.015	11954000
Hek_3	GTVHAR	SAMM50	Sorting and assembly machinery component 50 homolog	3	6	368.5526	46.435	0.063988	1	113.07	45.312	10166000
Hek_Myr2	GYEESEGHNTPKLKNQR	MORC4	MORC family CW-type zinc finger protein 4	3	17	817.4227	37.093	0.001381	1	48.405	41.652	84227000
Hek_1	GYEESEGHNTPKLKNQR	MORC4	MORC family CW-type zinc finger protein 4	3	17	817.4227	36.607	0.000309	3	58.885	48.511	108710000
Hek_2	GYEESEGHNTPKLKNQR	MORC4	MORC family CW-type zinc finger protein 4	3	17	817.4227	36.764	0.000891	1	51.03	41.219	115750000
Hek_3	GYEESEGHNTPKLKNQR	MORC4	MORC family CW-type zinc finger protein 4	3	17	817.4227	36.632	0.000891	3	51.03	41.973	62247000

HeLa MQ

Replicate	Sequence	Gene Names	Protein Names	Charge	Length	m/z	Retention time	PEP	MS/MS Count	Score	Delta score	Intensity
HeLa_2	CGNNMSTPLPAIVPAAR	LYPLA1	Acyl-protein thioesterase 1	3	17	744.7295	77.637	0.0017562	1	47.548	44.63	6038200
HeLa_3	CGNNMSTPLPAIVPAAR	LYPLA1	Acyl-protein thioesterase 1	3	17	744.7295	77.659	0.014254	1	35.473	33.17	4078600
HeLa_1	CGNTMSVPLLTDAATVSGAER	LYPLA2	Acyl-protein thioesterase 2	3	21	871.7756	84.186	0.0015373	1	47.499	44.353	3978100
HeLa_2	CGNTMSVPLLTDAATVSGAER	LYPLA2	Acyl-protein thioesterase 2	3	21	871.7756	84.103	0.0001873	1	57.484	54.064	4402600
HeLa_3	CGNTMSVPLLTDAATVSGAER	LYPLA2	Acyl-protein thioesterase 2	3	21	871.7756	84.21	0.010953	1	34.65	31.316	3358700
HeLa_1	GAAGSSALAR	MARC1	MOSC domain-containing protein 1, mitochondrial	3	10	441.9212	61.061	0.000131	1	119.68	69.656	47356000
HeLa_1	GAAGSSALAR	MARC1	MOSC domain-containing protein 1, mitochondrial	2	10	662.3782	61.059	0.001184	1	99.568	44.127	46261000
HeLa_2	GAAGSSALAR	MARC1	MOSC domain-containing protein 1, mitochondrial	3	10	441.9212	61.034	0.0004464	2	111.61	66.915	26292000
HeLa_2	GAAGSSALAR	MARC1	MOSC domain-containing protein 1, mitochondrial	2	10	662.3782	61.032	0.009323	1	62.78	34.606	34938000
HeLa_3	GAAGSSALAR	MARC1	MOSC domain-containing protein 1, mitochondrial	3	10	441.9212	61.096	0.0005709	1	109.29	69.627	35961000
HeLa_3	GAAGSSALAR	MARC1	MOSC domain-containing protein 1, mitochondrial	2	10	662.3782	61.086	0.001231	1	101.38	42.4	34744000
HeLa_1	GAAHSASEEVR	TESC	Calcineurin B homologous protein 3	3	11	526.2779	45.66	4.21E-12	2	170.89	135.23	385720000
HeLa_2	GAAHSASEEVR	TESC	Calcineurin B homologous protein 3	3	11	526.2779	45.545	2.84E-44	2	220.1	177.07	492830000
HeLa_3	GAAHSASEEVR	TESC	Calcineurin B homologous protein 3	3	11	526.2779	45.688	3.91E-70	2	236.77	179.33	537540000
HeLa_1	GAFLDKPK	PPM1A;PPM	Protein phosphatase 1A;Protein phosphatase 1B	3	8	446.9346	67.381	0.071967	1	76.478	56.701	73276000
HeLa_2	GAFLDKPK	PPM1A;PPM	Protein phosphatase 1A;Protein phosphatase 1B	3	8	446.9346	67.386	0.056134	1	93.429	67.045	97577000
HeLa_3	GAFLDKPK	PPM1A;PPM	Protein phosphatase 1A;Protein phosphatase 1B	3	8	446.9346	67.401	0.08043	1	73.665	38.309	55990000
HeLa_1	GAGSSTEQR	AKAP12	A-kinase anchor protein 12	3	9	452.5724	51.577	0.0015925	1	113.18	91.629	86262000
HeLa_2	GAGSSTEQR	AKAP12	A-kinase anchor protein 12	3	9	452.5724	51.456	0.0010705	1	123.35	101.81	85242000
HeLa_3	GAGSSTEQR	AKAP12	A-kinase anchor protein 12	3	9	452.5724	51.573	0.0014872	1	113.74	94.934	62295000
HeLa_3	GAQFSK	MARCKS	Myristoylated alanine-rich C-kinase substrate	2	6	550.8142	62.903	0.011417	1	164.1	81.961	1.173E+09
HeLa_3	GASGSK	TUSC2	Tumor suppressor candidate 2	2	6	485.2774	50.2	0.076544	1	107.22	32.505	12612000
HeLa_1	GASSSSALAR	MARC2	MOSC domain-containing protein 2, mitochondrial	3	10	457.2564	58.523	0.000607	1	99.257	81.437	26255000
HeLa_2	GASSSSALAR	MARC2	MOSC domain-containing protein 2, mitochondrial	3	10	457.2564	58.47	0.0005724	1	96.665	78.817	26023000
HeLa_3	GASSSSALAR	MARC2	MOSC domain-containing protein 2, mitochondrial	3	10	457.2564	58.512	0.0011889	1	88.392	70.572	20879000
HeLa_1	GAYLSQPNTVK	PPM1G	Protein phosphatase 1G	3	11	547.6421	71.96	0.0005279	1	76.843	57.185	187910000
HeLa_1	GAYLSQPNTVK	PPM1G	Protein phosphatase 1G	2	11	820.9596	71.961	4.35E-07	1	155.47	122.42	49263000
HeLa_2	GAYLSQPNTVK	PPM1G	Protein phosphatase 1G	3	11	547.6421	72.006	0.0008252	1	69.786	56.385	198360000
HeLa_2	GAYLSQPNTVK	PPM1G	Protein phosphatase 1G	2	11	820.9596	72.006	7.93E-07	1	138.76	115.44	60090000
HeLa_3	GAYLSQPNTVK	PPM1G	Protein phosphatase 1G	3	11	547.6421	72.044	2.17E-07	2	127.83	105.8	143870000
HeLa_3	GAYLSQPNTVK	PPM1G	Protein phosphatase 1G	2	11	820.9596	72.042	9.27E-05	2	106.62	79.191	44120000
HeLa_1	GCCSSASSAAQSSK	SLC44A1	Choline transporter-like protein 1	3	14	617.621	50.586	0.0050196	1	45.873	42.44	11654000
HeLa_2	GCCSSASSAAQSSK	SLC44A1	Choline transporter-like protein 1	3	14	617.621	50.547	0.0021465	1	57.794	55.134	11091000
HeLa_1	GCCYSSENEDSDQDREER	LAMTOR1	Ragulator complex protein LAMTOR1	4	18	675.5319	51.011	1.53E-06	1	92.264	88.929	86805000
HeLa_1	GCCYSSENEDSDQDREER	LAMTOR1	Ragulator complex protein LAMTOR1	3	18	900.3734	51.006	2.02E-18	1	127.83	117.01	94440000
HeLa_2	GCCYSSENEDSDQDREER	LAMTOR1	Ragulator complex protein LAMTOR1	3	18	900.3734	50.864	3.33E-26	1	141.86	123.63	122540000
HeLa_2	GCCYSSENEDSDQDREER	LAMTOR1	Ragulator complex protein LAMTOR1	4	18	675.5319	50.863	7.00E-14	1	125.98	122.64	96858000
HeLa_3	GCCYSSENEDSDQDREER	LAMTOR1	Ragulator complex protein LAMTOR1	4	18	675.5319	50.926	2.49E-06	1	83.758	77.944	57501000
HeLa_3	GCCYSSENEDSDQDREER	LAMTOR1	Ragulator complex protein LAMTOR1	3	18	900.3734	50.922	2.01E-05	1	69.081	62.599	67643000
HeLa_1	GCGLNK	RFTN1	Raftlin	2	6	556.3057	58.86	0.08065	1	107.73	39.089	19908000
HeLa_2	GCGLNK	RFTN1	Raftlin	2	6	556.3057	58.814	0.082803	1	103.79	36.497	24603000
HeLa_1	GCTLSAEDK	GNAI3;GNAI	Guanine nucleotide-binding protein G(k) subunit alpha;G	2	9	722.3667	63.383	2.37E-06	2	170.47	114.07	287050000
HeLa_1	GCTLSAEDK	GNAI3;GNAI	Guanine nucleotide-binding protein G(k) subunit alpha;G	3	9	481.9135	63.382	0.007482	1	87.149	69.636	85708000
HeLa_2	GCTLSAEDK	GNAI3;GNAI	Guanine nucleotide-binding protein G(k) subunit alpha;G	2	9	722.3667	63.442	0.0022688	2	142.43	95.599	307730000
HeLa_2	GCTLSAEDK	GNAI3;GNAI	Guanine nucleotide-binding protein G(k) subunit alpha;G	3	9	481.9135	63.443	0.0079848	1	80.688	63.458	90645000

HeLa_3	GCTLSAEDK	GNAI3;GNAI	Guanine nucleotide-binding protein G(k) subunit alpha;G	2	9	722.3667	63.461	0.0007051	2	163.16	115.27	204570000
HeLa_3	GCTLSAEDK	GNAI3;GNAI	Guanine nucleotide-binding protein G(k) subunit alpha;G	3	9	481.9135	63.46	0.0072916	1	87.806	72.191	63782000
HeLa_1	GCTLSAEER	GNAO1	Guanine nucleotide-binding protein G(o) subunit alpha	3	9	495.9208	63.809	0.015374	1	64.191	51.955	8798700
HeLa_2	GCTLSAEER	GNAO1	Guanine nucleotide-binding protein G(o) subunit alpha	3	9	495.9208	63.772	0.011354	1	69.915	56.938	10719000
HeLa_2	GCTLSAEER	GNAO1	Guanine nucleotide-binding protein G(o) subunit alpha	2	9	743.3776	63.807	0.04732	1	58.699	33.125	7301800
HeLa_3	GCTLSAEER	GNAO1	Guanine nucleotide-binding protein G(o) subunit alpha	3	9	495.9208	63.881	0.011354	1	69.915	54.033	7185500
HeLa_1	GCTVSAEDK	GNAI2	Guanine nucleotide-binding protein G(i) subunit alpha-2	2	9	715.3588	58.706	1.06E-10	1	183.67	99.89	181440000
HeLa_2	GCTVSAEDK	GNAI2	Guanine nucleotide-binding protein G(i) subunit alpha-2	2	9	715.3588	58.669	0.0025781	1	157.66	83.287	192760000
HeLa_3	GCTVSAEDK	GNAI2	Guanine nucleotide-binding protein G(i) subunit alpha-2	3	9	477.2416	58.693	0.0045045	1	98.009	41.631	37262000
HeLa_3	GCTVSAEDK	GNAI2	Guanine nucleotide-binding protein G(i) subunit alpha-2	2	9	715.3588	58.694	0.0006557	1	163.64	87.539	147090000
HeLa_1	GDVLSTHLDDAR	FAM129B	Niban-like protein 1	3	12	587.9796	70.112	1.13E-13	3	164.11	134.37	378530000
HeLa_1	GDVLSTHLDDAR	FAM129B	Niban-like protein 1	4	12	441.2365	70.094	0.0036864	1	45.28	40.386	5962400
HeLa_2	GDVLSTHLDDAR	FAM129B	Niban-like protein 1	3	12	587.9796	70.227	1.69E-17	3	176.05	146.31	512140000
HeLa_3	GDVLSTHLDDAR	FAM129B	Niban-like protein 1	3	12	587.9796	70.151	2.45E-17	2	174.05	144.31	371950000
HeLa_3	GDVLSTHLDDAR	FAM129B	Niban-like protein 1	4	12	441.2365	70.152	0.0012477	1	52.576	43.254	3847900
HeLa_1	GDVLSTHLDDARR	FAM129B	Niban-like protein 1	4	13	480.2618	62.099	1.10E-06	1	113.99	100.59	102110000
HeLa_2	GDVLSTHLDDARR	FAM129B	Niban-like protein 1	4	13	480.2618	62.142	1.53E-09	1	117.93	109.8	58744000
HeLa_3	GDVLSTHLDDARR	FAM129B	Niban-like protein 1	4	13	480.2618	62.222	0.0002532	2	74.987	66.855	69557000
HeLa_1	GGAVSAGEDNDDLIDNLK	PCMTD1	Protein-L-isoaspartate O-methyltransferase domain-cont	3	18	756.0485	77.745	0.0010924	1	49.537	46.986	18675000
HeLa_2	GGAVSAGEDNDDLIDNLK	PCMTD1	Protein-L-isoaspartate O-methyltransferase domain-cont	3	18	756.0485	77.749	8.83E-07	1	100.58	92.868	21950000
HeLa_3	GGAVSAGEDNDDLIDNLK	PCMTD1	Protein-L-isoaspartate O-methyltransferase domain-cont	3	18	756.0485	77.78	6.04E-06	1	72.433	69.674	14902000
HeLa_1	GGAVSAGEDNDELIDNLK	PCMTD2	Protein-L-isoaspartate O-methyltransferase domain-cont	3	18	760.7204	76.442	1.26E-05	1	66.022	60.127	7464300
HeLa_2	GGAVSAGEDNDELIDNLK	PCMTD2	Protein-L-isoaspartate O-methyltransferase domain-cont	3	18	760.7204	76.424	1.50E-06	1	96.208	91.304	10457000
HeLa_1	GGLFSR	LNP;KIAA17	Protein lunapark	2	6	550.3222	80.112	0.077263	1	105.42	34.352	20439000
HeLa_1	GGSASSQLDEGK	FAM129A	Protein Niban	3	12	533.6093	57.448	0.0002078	1	80.623	60.965	50374000
HeLa_1	GGSASSQLDEGK	FAM129A	Protein Niban	2	12	799.9103	57.454	4.19E-11	1	134.68	112.69	15292000
HeLa_2	GGSASSQLDEGK	FAM129A	Protein Niban	3	12	533.6093	57.358	0.0001133	1	95.094	73.066	36067000
HeLa_2	GGSASSQLDEGK	FAM129A	Protein Niban	2	12	799.9103	57.348	0.0002064	1	92.856	83.201	10969000
HeLa_3	GGSASSQLDEGK	FAM129A	Protein Niban	3	12	533.6093	57.464	5.22E-05	1	102.28	76.803	31884000
HeLa_3	GGSASSQLDEGK	FAM129A	Protein Niban	2	12	799.9103	57.478	4.19E-11	1	134.68	112.69	10228000
HeLa_1	GGSHSQTPR	TACC1	Transforming acidic coiled-coil-containing protein 1	3	9	463.9164	39.029	5.67E-11	1	183.43	125.84	38982000
HeLa_2	GGSHSQTPR	TACC1	Transforming acidic coiled-coil-containing protein 1	3	9	463.9164	38.952	1.20E-06	1	170.69	113.1	32421000
HeLa_3	GGSHSQTPR	TACC1	Transforming acidic coiled-coil-containing protein 1	3	9	463.9164	39.171	0.0003946	1	162.59	103.61	40714000
HeLa_1	GLSPSAPAVAVQASNASASPPSGCPMHEGK	HCCS	Cytochrome c-type heme lyase	4	30	832.1659	63.157	3.97E-58	1	143.07	142.6	204560000
HeLa_1	GLSPSAPAVAVQASNASASPPSGCPMHEGK	HCCS	Cytochrome c-type heme lyase	3	30	1109.219	63.157	1.80E-37	1	117.04	116.62	51967000
HeLa_2	GLSPSAPAVAVQASNASASPPSGCPMHEGK	HCCS	Cytochrome c-type heme lyase	4	30	832.1659	63.248	2.64E-49	1	134.21	133.12	263130000
HeLa_2	GLSPSAPAVAVQASNASASPPSGCPMHEGK	HCCS	Cytochrome c-type heme lyase	3	30	1109.219	63.25	9.31E-81	1	161.05	160.08	65671000
HeLa_3	GLSPSAPAVAVQASNASASPPSGCPMHEGK	HCCS	Cytochrome c-type heme lyase	4	30	832.1659	63.273	1.69E-25	1	92.258	90.358	128810000
HeLa_3	GLSPSAPAVAVQASNASASPPSGCPMHEGK	HCCS	Cytochrome c-type heme lyase	3	30	1109.219	63.271	8.28E-42	1	124.82	124.44	31049000
HeLa_1	GLSPSAPAVAVQASNASASPPSGCPMHEGK	HCCS	Cytochrome c-type heme lyase	4	30	836.1646	61.713	2.90E-05	1	34.861	30.336	23990000
HeLa_2	GLSPSAPAVAVQASNASASPPSGCPMHEGK	HCCS	Cytochrome c-type heme lyase	4	30	836.1646	61.72	1.26E-08	1	50.865	46.108	38785000
HeLa_3	GLSPSAPAVAVQASNASASPPSGCPMHEGK	HCCS	Cytochrome c-type heme lyase	4	30	836.1646	61.736	2.38E-11	1	60.029	55.503	51057000
HeLa_1	GLTISSLFSR	ARF4	ADP-ribosylation factor 4	3	10	515.3033	96.183	0.0005681	1	96.342	76.683	49979000
HeLa_1	GLTISSLFSR	ARF4	ADP-ribosylation factor 4	2	10	772.4514	96.178	0.0078661	1	63.624	45.805	16410000
HeLa_2	GLTISSLFSR	ARF4	ADP-ribosylation factor 4	3	10	515.3033	96.189	0.0006711	3	104.05	72.761	232410000
HeLa_3	GLTISSLFSR	ARF4	ADP-ribosylation factor 4	3	10	515.3033	96.203	0.0006688	3	103.88	88.014	138060000
HeLa_3	GLTISSLFSR	ARF4	ADP-ribosylation factor 4	2	10	772.4514	96.201	1.44E-14	1	190.57	163.47	59695000
HeLa_1	GLTVSALFSR	ARF5	ADP-ribosylation factor 5	3	10	505.2998	93.38	0.010493	1	56.404	46.033	1201700
HeLa_2	GLTVSALFSR	ARF5	ADP-ribosylation factor 5	3	10	505.2998	93.375	0.0006141	2	99.788	70.618	24553000
HeLa_3	GLTVSALFSR	ARF5	ADP-ribosylation factor 5	3	10	505.2998	93.45	0.038435	1	46.844	32.15	6500500

HeLa_1	GNAAAAK	PRKACA	cAMP-dependent protein kinase catalytic subunit alpha	2	7	533.3118	52.539	0.032383	2	138.37	88.058	432410000
HeLa_2	GNAAAAK	PRKACA	cAMP-dependent protein kinase catalytic subunit alpha	2	7	533.3118	52.43	0.029849	2	128.48	71.411	555200000
HeLa_3	GNAAAAK	PRKACA	cAMP-dependent protein kinase catalytic subunit alpha	2	7	533.3118	52.603	0.029835	2	124.34	74.033	287290000
HeLa_1	GNAAGSAEQPAGPAAPPPK	FMNL1	Formin-like protein 1	3	19	717.715	55.806	1.57E-05	1	55.437	52.381	10974000
HeLa_2	GNAAGSAEQPAGPAAPPPK	FMNL1	Formin-like protein 1	3	19	717.715	55.602	4.62E-09	1	62.658	54.316	15167000
HeLa_1	GNAATAK	PRKACB	cAMP-dependent protein kinase catalytic subunit beta	2	7	548.3171	52.53	0.072016	1	101.28	42.623	204960000
HeLa_1	GNAGSMDSQQTDFR	FMNL2	Formin-like protein 2	3	14	659.6463	63.177	0.0022484	1	67.102	65.104	5416900
HeLa_1	GNAQERPSETIDR	NOL3	Nucleolar protein 3	3	13	646.0047	48.096	0.0045687	2	58.32	42.73	106800000
HeLa_2	GNAQERPSETIDR	NOL3	Nucleolar protein 3	3	13	646.0047	48.017	0.0021822	1	76.759	58.623	145690000
HeLa_3	GNAQERPSETIDR	NOL3	Nucleolar protein 3	3	13	646.0047	48.068	0.0045687	2	58.32	42.691	67943000
HeLa_1	GNEASYPLEMCSHFDADEIKR	PPP3R1	Calcineurin subunit B type 1	4	21	737.8471	58.826	3.96E-08	1	73.672	72.004	21777000
HeLa_2	GNEASYPLEMCSHFDADEIKR	PPP3R1	Calcineurin subunit B type 1	4	21	737.8471	58.656	4.36E-14	1	109.85	107.47	38238000
HeLa_3	GNEASYPLEMCSHFDADEIKR	PPP3R1	Calcineurin subunit B type 1	4	21	737.8471	58.696	3.13E-12	1	105.46	101.25	28870000
HeLa_1	GNEASYPLEMCSHFDADEIKR	PPP3R1	Calcineurin subunit B type 1	4	21	733.8484	63.731	6.07E-26	1	138.03	127.68	142820000
HeLa_2	GNEASYPLEMCSHFDADEIKR	PPP3R1	Calcineurin subunit B type 1	4	21	733.8484	63.499	3.62E-33	1	147.25	140.45	280030000
HeLa_3	GNEASYPLEMCSHFDADEIKR	PPP3R1	Calcineurin subunit B type 1	4	21	733.8484	63.87	1.17E-24	1	132.01	121.46	102090000
HeLa_1	GNHSGRPEDPEPGAFTTTK	SPECC1	Cytospin-B	4	19	616.0609	45.651	5.47E-44	1	172.5	164.58	98129000
HeLa_2	GNHSGRPEDPEPGAFTTTK	SPECC1	Cytospin-B	4	19	616.0609	45.505	7.60E-20	1	135.26	131.21	71756000
HeLa_1	GNIFANLFK	ARF1	ADP-ribosylation factor 1	2	9	743.9301	101.31	0.025914	1	66.692	42.662	7906600
HeLa_2	GNIFANLFK	ARF1	ADP-ribosylation factor 1	2	9	743.9301	101.29	0.0010742	2	159.58	119.23	120970000
HeLa_2	GNIFANLFK	ARF1	ADP-ribosylation factor 1	3	9	496.2891	101.3	0.0071504	1	88.294	60.076	147610000
HeLa_3	GNIFANLFK	ARF1	ADP-ribosylation factor 1	2	9	743.9301	101.32	0.0023822	2	128.36	88.01	34961000
HeLa_3	GNIFANLFK	ARF1	ADP-ribosylation factor 1	3	9	496.2891	101.31	0.023354	1	59.35	37.39	58893000
HeLa_1	GNIFGNLLK	ARF3	ADP-ribosylation factor 3	2	9	719.9301	94.526	0.0017146	1	137.13	91.002	45654000
HeLa_1	GNIFGNLLK	ARF3	ADP-ribosylation factor 3	3	9	480.2891	94.524	0.0079578	1	82.426	49.533	29513000
HeLa_2	GNIFGNLLK	ARF3	ADP-ribosylation factor 3	2	9	719.9301	94.542	0.0078517	2	146.23	94.886	147230000
HeLa_2	GNIFGNLLK	ARF3	ADP-ribosylation factor 3	3	9	480.2891	94.545	0.0079154	1	85.161	57.337	98544000
HeLa_3	GNIFGNLLK	ARF3	ADP-ribosylation factor 3	2	9	719.9301	94.593	0.0022858	1	126.07	78.881	172710000
HeLa_3	GNIFGNLLK	ARF3	ADP-ribosylation factor 3	3	9	480.2891	94.593	0.0080235	1	78.192	49.495	118990000
HeLa_1	GNLFGR	CHMP6	Charged multivesicular body protein 6	2	6	563.8276	79.773	0.083106	1	103.7	63.723	33228000
HeLa_1	GNSASNIVSPQEALPGR	MSRA	Mitochondrial peptide methionine sulfoxide reductase	3	17	720.7222	67.992	1.94E-16	1	132.64	126.93	19785000
HeLa_2	GNSASNIVSPQEALPGR	MSRA	Mitochondrial peptide methionine sulfoxide reductase	3	17	720.7222	67.957	6.45E-07	1	106.93	101.21	17729000
HeLa_3	GNSASNIVSPQEALPGR	MSRA	Mitochondrial peptide methionine sulfoxide reductase	3	17	720.7222	67.963	5.55E-12	1	127.32	121.78	18704000
HeLa_3	GNTLGLAPMGTLPR	TOMM40L	Mitochondrial import receptor subunit TOM40B	3	14	626.3524	75.304	0.011452	1	38.995	33.454	2134400
HeLa_1	GNTLGLAPMGTLPR	TOMM40L	Mitochondrial import receptor subunit TOM40B	3	14	621.0207	82.062	0.0025035	1	52.527	44.117	6054300
HeLa_2	GNTLGLAPMGTLPR	TOMM40L	Mitochondrial import receptor subunit TOM40B	3	14	621.0207	82.078	0.0006762	1	85.163	72.532	7532200
HeLa_3	GNTLGLAPMGTLPR	TOMM40L	Mitochondrial import receptor subunit TOM40B	3	14	621.0207	82.084	0.015268	1	40.278	35.937	4352300
HeLa_1	GNTTTK	ANKIB1	Ankyrin repeat and IBR domain-containing protein 1	2	6	542.8091	51.137	0.078225	1	107.43	47.82	10297000
HeLa_1	GQQISDQTQLVINK	RNF141	RING finger protein 141	3	14	679.048	70.025	8.32E-18	1	146.77	100.7	31650000
HeLa_2	GQQISDQTQLVINK	RNF141	RING finger protein 141	3	14	679.048	70.142	2.84E-09	1	122.97	77.685	35023000
HeLa_3	GQQISDQTQLVINK	RNF141	RING finger protein 141	3	14	679.048	70.068	1.10E-17	1	144.8	106.5	32184000
HeLa_1	GQSQSGGHGPGGGK	PSMC1	26S protease regulatory subunit 4	3	14	558.6204	38.464	3.81E-14	2	128.22	111.9	974000000
HeLa_2	GQSQSGGHGPGGGK	PSMC1	26S protease regulatory subunit 4	3	14	558.6204	38.44	1.06E-17	1	145.1	126.12	1.363E+09
HeLa_3	GQSQSGGHGPGGGK	PSMC1	26S protease regulatory subunit 4	3	14	558.6204	38.704	2.06E-18	1	155.43	147.71	650400000
HeLa_1	GQSQSGGHGPGGGKK	PSMC1	26S protease regulatory subunit 4	4	15	451.2409	29.868	0.0003493	1	66.246	39.46	205860000
HeLa_1	GQSQSGGHGPGGGKK	PSMC1	26S protease regulatory subunit 4	3	15	601.3187	29.857	0.0002914	1	69.92	42.439	17732000
HeLa_2	GQSQSGGHGPGGGKK	PSMC1	26S protease regulatory subunit 4	4	15	451.2409	30.14	9.60E-05	1	89.507	62.721	149650000
HeLa_2	GQSQSGGHGPGGGKK	PSMC1	26S protease regulatory subunit 4	3	15	601.3187	30.106	9.57E-05	1	94.487	65.584	8054700
HeLa_3	GQSQSGGHGPGGGKK	PSMC1	26S protease regulatory subunit 4	4	15	451.2409	30.224	0.0001191	2	82.069	55.283	116530000
HeLa_3	GQSQSGGHGPGGGKK	PSMC1	26S protease regulatory subunit 4	3	15	601.3187	30.223	0.0001618	1	75.764	45.559	8330400

HeLa_2	GSENSALK	SCYL3	Protein-associating with the carboxyl-terminal domain of	2	8	634.8515	57.032	0.0033815	1	96.034	53.119	10219000
HeLa_1	GSEQSSEAESRPNDLNSSVTPSPAK	LOH12CR1	Loss of heterozygosity 12 chromosomal region 1 protein	4	25	760.126	50.089	9.98E-32	1	136.34	123.45	35222000
HeLa_2	GSEQSSEAESRPNDLNSSVTPSPAK	LOH12CR1	Loss of heterozygosity 12 chromosomal region 1 protein	4	25	760.126	50.11	1.38E-39	1	143.29	134.43	21964000
HeLa_3	GSEQSSEAESRPNDLNSSVTPSPAK	LOH12CR1	Loss of heterozygosity 12 chromosomal region 1 protein	4	25	760.126	50.109	8.83E-25	1	122.34	109.96	26033000
HeLa_1	GSQHSAAAR	TMEM106C	Transmembrane protein 106C	3	9	449.9129	38.346	0.0038686	1	101.25	84.698	185380000
HeLa_2	GSQHSAAAR	TMEM106C	Transmembrane protein 106C	3	9	449.9129	38.361	1.20E-06	1	170.69	147.57	220640000
HeLa_3	GSQHSAAAR	TMEM106C	Transmembrane protein 106C	3	9	449.9129	38.553	0.0043198	2	146.88	128.08	129590000
HeLa_2	GSQSSK	MARCKSL1	MARCKS-related protein	2	6	528.7935	48.261	0.079533	1	152.63	71.252	105610000
HeLa_3	GSQSSK	MARCKSL1	MARCKS-related protein	2	6	528.7935	48.352	0.012882	1	163.34	73.668	78654000
HeLa_1	GSSQSVEIPGGGTEGYHVLR	GORASP2	Golgi reassembly-stacking protein 2	3	20	831.7663	61.922	4.06E-24	1	135.38	126.37	443930000
HeLa_1	GSSQSVEIPGGGTEGYHVLR	GORASP2	Golgi reassembly-stacking protein 2	4	20	624.0766	61.921	7.06E-14	1	113.73	105.98	249270000
HeLa_2	GSSQSVEIPGGGTEGYHVLR	GORASP2	Golgi reassembly-stacking protein 2	3	20	831.7663	61.952	1.03E-25	1	138.28	131.51	287970000
HeLa_2	GSSQSVEIPGGGTEGYHVLR	GORASP2	Golgi reassembly-stacking protein 2	4	20	624.0766	61.952	5.02E-18	1	121.44	117.46	152900000
HeLa_3	GSSQSVEIPGGGTEGYHVLR	GORASP2	Golgi reassembly-stacking protein 2	3	20	831.7663	62.024	1.45E-41	2	154.26	142.22	151750000
HeLa_3	GSSQSVEIPGGGTEGYHVLR	GORASP2	Golgi reassembly-stacking protein 2	4	20	624.0766	62.023	5.10E-11	2	100.45	100.45	89813000
HeLa_1	GSTWGSPGWVR	VKORC1	Vitamin K epoxide reductase complex subunit 1	3	11	551.6267	77.164	0.0073898	1	53.08	46.176	1199700
HeLa_3	GSTWGSPGWVR	VKORC1	Vitamin K epoxide reductase complex subunit 1	3	11	551.6267	77.19	0.020561	1	46.73	37.075	2009500
HeLa_1	GTPASVVSEPPPWQAPIEAR	AVPI1	Arginine vasopressin-induced protein 1	3	20	851.4588	80.847	0.0067655	1	40.968	34.851	3018000
HeLa_2	GTPASVVSEPPPWQAPIEAR	AVPI1	Arginine vasopressin-induced protein 1	3	20	851.4588	80.868	1.83E-06	1	66.152	58.833	4042800
HeLa_2	GVGAGR	FKBP8	Peptidyl-prolyl cis-trans isomerase FKBP8	2	6	490.2934	59.4	0.042742	1	156.86	50.21	12970000
HeLa_3	GVGAGR	FKBP8	Peptidyl-prolyl cis-trans isomerase FKBP8	2	6	490.2934	59.436	1.88E-18	1	150	40.471	11391000
HeLa_Myr1	GYEESEGHNTPKLKNQR	MORC4	MORC family CW-type zinc finger protein 4	3	17	817.4227	36.322	0.0008911	1	51.03	42.73	176050000
HeLa_Myr2	GYEESEGHNTPKLKNQR	MORC4	MORC family CW-type zinc finger protein 4	3	17	817.4227	36.566	0.0008911	2	51.03	41.973	157210000
HeLa_1	GYEESEGHNTPKLKNQR	MORC4	MORC family CW-type zinc finger protein 4	3	17	817.4227	36.279	0.0003095	1	58.885	42.356	97117000
HeLa_1	GYEESEGHNTPKLKNQR	MORC4	MORC family CW-type zinc finger protein 4	3	17	817.4227	36.62	0.0003095	1	58.885	47.242	87356000
HeLa_2	GYEESEGHNTPKLKNQR	MORC4	MORC family CW-type zinc finger protein 4	3	17	817.4227	36.466	0.0003141	2	57.288	46.174	131260000
HeLa_3	GYEESEGHNTPKLKNQR	MORC4	MORC family CW-type zinc finger protein 4	3	17	817.4227	36.763	0.0022785	1	43.592	34.916	64255000

MCF7 MQ

Replicate	Sequence	Gene Names	Protein Names	Charge	Length	m/z	Retention time	PEP	MS/MS Count	Score	Delta score	Intensity
MCF_1	CGNNMSTPLPAIVPAAR	LYPLA1	Acyl-protein thioesterase 1	3	17	744.72951	77.728	0.0061457	1	41.088	38.428	2543900
MCF_3	CGNNMSTPLPAIVPAAR	LYPLA1	Acyl-protein thioesterase 1	3	17	744.72951	77.859	0.013451	1	36.029	33.555	2890400
MCF_3	GAAHSASEEVR	TESC	Calcineurin B homologous protein 3	3	11	526.27786	45.864	0.038418	1	45.829	33.666	1993200
MCF_1	GAFLDKPK	PPM1A;PPM1B	Protein phosphatase 1A;Protein phosphatase 1B	3	8	446.9346	67.284	0.07142	1	84.743	58.359	65651000
MCF_3	GAFLDKPK	PPM1A;PPM1B	Protein phosphatase 1A;Protein phosphatase 1B	3	8	446.9346	67.587	0.072016	2	75.738	60.698	84035000
MCF_1	GASSSSALAR	MARC2	MOSC domain-containing protein 2, mitochondrial	3	10	457.2564	58.507	0.001672	1	79.16	62.489	5040700
MCF_2	GASSSSALAR	MARC2	MOSC domain-containing protein 2, mitochondrial	3	10	457.2564	58.606	0.002835	1	67.334	49.514	4775200
MCF_3	GASSSSALAR	MARC2	MOSC domain-containing protein 2, mitochondrial	3	10	457.2564	58.672	0.0014107	1	85.533	67.714	5113100
MCF_1	GAYLSQPNTVK	PPM1G	Protein phosphatase 1G	3	11	547.64214	71.977	0.0063797	1	53.567	33.909	87322000
MCF_1	GAYLSQPNTVK	PPM1G	Protein phosphatase 1G	2	11	820.95957	71.976	7.52E-07	1	137.55	102.79	23525000
MCF_2	GAYLSQPNTVK	PPM1G	Protein phosphatase 1G	3	11	547.64214	72.145	0.022429	1	45.829	32.429	93545000
MCF_3	GAYLSQPNTVK	PPM1G	Protein phosphatase 1G	3	11	547.64214	72.176	0.0001608	1	87.568	71.704	106290000
MCF_3	GAYLSQPNTVK	PPM1G	Protein phosphatase 1G	2	11	820.95957	72.171	1.08E-06	1	147.62	107.27	24971000
MCF_1	GCCSSASSAAQSSK	SLC44A1	Choline transporter-like protein 1	3	14	617.62097	50.487	0.017252	1	39.587	36.802	11398000
MCF_2	GCCSSASSAAQSSK	SLC44A1	Choline transporter-like protein 1	3	14	617.62097	50.676	0.024176	1	37.177	36.161	13147000
MCF_3	GCCSSASSAAQSSK	SLC44A1	Choline transporter-like protein 1	3	14	617.62097	50.757	0.0018493	1	70.26	68.125	17031000
MCF_1	GCCYSSENEDSDQDREER	LAMTOR1	Ragulator complex protein LAMTOR1	3	18	900.3734	50.943	0.012247	1	40.968	37.095	54254000
MCF_1	GCCYSSENEDSDQDREER	LAMTOR1	Ragulator complex protein LAMTOR1	4	18	675.53187	50.941	3.64E-13	1	118.21	114.08	46556000
MCF_2	GCCYSSENEDSDQDREER	LAMTOR1	Ragulator complex protein LAMTOR1	4	18	675.53187	51.031	9.89E-05	1	54.103	52.695	35651000
MCF_2	GCCYSSENEDSDQDREER	LAMTOR1	Ragulator complex protein LAMTOR1	3	18	900.3734	51.029	8.03E-06	1	79.514	69.247	46315000
MCF_3	GCCYSSENEDSDQDREER	LAMTOR1	Ragulator complex protein LAMTOR1	4	18	675.53187	51.21	5.54E-06	1	69.081	66.303	41487000
MCF_3	GCCYSSENEDSDQDREER	LAMTOR1	Ragulator complex protein LAMTOR1	3	18	900.3734	51.208	0.0002524	1	57.525	54.19	40282000
MCF_1	GCGLNK	RFTN1	Raftlin	2	6	556.30568	58.87	0.081461	1	75.682	30.948	7929400
MCF_1	GCTLSAEDK	GNAI3;GNAI1	Guanine nucleotide-binding protein G(k) subunit alpha;G	2	9	722.36666	63.395	0.0022075	2	140.17	101.39	156160000
MCF_1	GCTLSAEDK	GNAI3;GNAI1	Guanine nucleotide-binding protein G(k) subunit alpha;G	3	9	481.91353	63.392	0.011265	1	70.056	57.02	48257000
MCF_2	GCTLSAEDK	GNAI3;GNAI1	Guanine nucleotide-binding protein G(k) subunit alpha;G	2	9	722.36666	63.495	0.0009708	2	160.59	104.18	140850000
MCF_2	GCTLSAEDK	GNAI3;GNAI1	Guanine nucleotide-binding protein G(k) subunit alpha;G	3	9	481.91353	63.492	0.0079759	1	81.263	64.033	43806000
MCF_3	GCTLSAEDK	GNAI3;GNAI1	Guanine nucleotide-binding protein G(k) subunit alpha;G	2	9	722.36666	63.65	0.0022075	2	147.2	99.597	128020000
MCF_3	GCTLSAEDK	GNAI3;GNAI1	Guanine nucleotide-binding protein G(k) subunit alpha;G	3	9	481.91353	63.649	0.0079848	1	80.688	60.168	61051000
MCF_1	GCTVSAEDK	GNAI2	Guanine nucleotide-binding protein G(i) subunit alpha-2	3	9	477.24165	58.694	0.01073	1	70.908	35.64	16086000
MCF_1	GCTVSAEDK	GNAI2	Guanine nucleotide-binding protein G(i) subunit alpha-2	2	9	715.35883	58.702	0.0083473	1	150.81	77.577	70038000
MCF_2	GCTVSAEDK	GNAI2	Guanine nucleotide-binding protein G(i) subunit alpha-2	3	9	477.24165	58.762	0.0079848	1	80.688	33.086	20011000
MCF_2	GCTVSAEDK	GNAI2	Guanine nucleotide-binding protein G(i) subunit alpha-2	2	9	715.35883	58.762	0.0074922	1	151.83	75.726	86033000
MCF_3	GCTVSAEDK	GNAI2	Guanine nucleotide-binding protein G(i) subunit alpha-2	3	9	477.24165	58.881	0.013208	1	66.962	31.81	18679000
MCF_1	GDVLSTHLDDAR	FAM129B	Niban-like protein 1	3	12	587.97959	70.115	6.44E-12	2	156.14	138	178290000
MCF_1	GDVLSTHLDDAR	FAM129B	Niban-like protein 1	4	12	441.23651	70.117	0.0012477	1	52.576	46.3	3297900
MCF_2	GDVLSTHLDDAR	FAM129B	Niban-like protein 1	3	12	587.97959	70.298	1.13E-13	2	164.11	148.97	124970000
MCF_3	GDVLSTHLDDAR	FAM129B	Niban-like protein 1	3	12	587.97959	70.47	6.44E-12	3	156.14	156.14	110560000
MCF_3	GDVLSTHLDDAR	FAM129B	Niban-like protein 1	4	12	441.23651	70.471	0.011251	1	39.006	33.907	2258300
MCF_1	GDVLSTHLDDARR	FAM129B	Niban-like protein 1	4	13	480.26179	62.143	0.0001438	1	83.53	63.872	23154000
MCF_2	GDVLSTHLDDARR	FAM129B	Niban-like protein 1	4	13	480.26179	62.277	0.0001623	1	82.069	73.937	19094000
MCF_3	GDVLSTHLDDARR	FAM129B	Niban-like protein 1	4	13	480.26179	62.427	0.0002532	1	74.987	61.587	15625000
MCF_1	GGAVSAGEDNDDLIDNLK	PCMTD1	Protein-L-isoaspartate O-methyltransferase domain-con	3	18	756.04851	77.81	1.10E-06	1	99.044	93.149	18159000
MCF_2	GGAVSAGEDNDDLIDNLK	PCMTD1	Protein-L-isoaspartate O-methyltransferase domain-con	3	18	756.04851	77.869	1.34E-05	1	65.231	62.473	12548000
MCF_3	GGAVSAGEDNDDLIDNLK	PCMTD1	Protein-L-isoaspartate O-methyltransferase domain-con	3	18	756.04851	77.972	4.47E-06	1	85.563	82.805	18278000
MCF_1	GGAVSAGEDNDELIDNLK	PCMTD2	Protein-L-isoaspartate O-methyltransferase domain-con	3	18	760.72039	76.444	0.0001186	1	57.525	54.47	9436100

MCF_3	GGAVSAGEDNDELIDNLK	PCMTD2	Protein-L-isoaspartate O-methyltransferase domain-con	3	18	760.72039	76.624	3.44E-06	1	76.586	71.682	17445000
MCF_1	GLSPSAPAVAVQASNASASPPSGCPMHEGK	HCCS	Cytochrome c-type heme lyase	4	30	832.16586	63.208	2.57E-14	1	73.13	71.805	26087000
MCF_1	GLTISSLFSR	ARF4	ADP-ribosylation factor 4	3	10	515.30335	96.274	0.0005681	1	96.342	80.477	66170000
MCF_1	GLTISSLFSR	ARF4	ADP-ribosylation factor 4	2	10	772.45138	96.275	0.0078661	1	63.624	45.805	25892000
MCF_2	GLTISSLFSR	ARF4	ADP-ribosylation factor 4	3	10	515.30335	96.299	0.0006761	2	104.42	75.467	59272000
MCF_2	GLTISSLFSR	ARF4	ADP-ribosylation factor 4	2	10	772.45138	96.302	0.0029803	1	82.417	64.597	17241000
MCF_3	GLTISSLFSR	ARF4	ADP-ribosylation factor 4	3	10	515.30335	96.429	0.001418	2	85.355	58.568	43374000
MCF_3	GLTISSLFSR	ARF4	ADP-ribosylation factor 4	2	10	772.45138	96.439	0.0061143	1	64.82	47.001	14710000
MCF_1	GLTVSALFSR	ARF5	ADP-ribosylation factor 5	3	10	505.29983	93.566	0.0008147	2	93.096	64.138	14638000
MCF_2	GLTVSALFSR	ARF5	ADP-ribosylation factor 5	3	10	505.29983	93.51	0.014381	1	53.567	34.252	11845000
MCF_3	GLTVSALFSR	ARF5	ADP-ribosylation factor 5	3	10	505.29983	93.696	0.001418	1	85.355	50.968	14148000
MCF_1	GNAAAAK	PRKACA	cAMP-dependent protein kinase catalytic subunit alpha	2	7	533.31182	52.57	0.031225	1	111.73	61.417	107160000
MCF_3	GNAAAAK	PRKACA	cAMP-dependent protein kinase catalytic subunit alpha	2	7	533.31182	52.719	0.04461	1	105.39	55.081	99367000
MCF_1	GNAQERPSETIDR	NOL3	Nucleolar protein 3	3	13	646.00469	47.948	0.0013256	1	95.264	77.128	65584000
MCF_2	GNAQERPSETIDR	NOL3	Nucleolar protein 3	3	13	646.00469	48.245	0.0013499	1	83.692	65.555	68758000
MCF_3	GNAQERPSETIDR	NOL3	Nucleolar protein 3	3	13	646.00469	48.446	0.0045518	1	57.434	45.166	59405000
MCF_3	GNAQERPSETIDR	NOL3	Nucleolar protein 3	4	13	484.75534	48.475	0.0028112	1	49.298	42.695	3787100
MCF_3	GNCCWTQCFGLLR	AP1AR	AP-1 complex-associated regulatory protein	3	13	712.34137	84.984	0.018822	1	40.475	37.717	932280
MCF_1	GNEASYPLEMCSHFDADEIKR	PPP3R1	Calcineurin subunit B type 1	4	21	737.8471	58.795	4.34E-12	1	104.95	102.57	25828000
MCF_3	GNEASYPLEMCSHFDADEIKR	PPP3R1	Calcineurin subunit B type 1	4	21	737.8471	58.861	1.96E-11	1	98.543	97.288	21896000
MCF_1	GNEASYPLEMCSHFDADEIKR	PPP3R1	Calcineurin subunit B type 1	4	21	733.84837	63.787	1.10E-11	1	65.438	60.892	45410000
MCF_2	GNEASYPLEMCSHFDADEIKR	PPP3R1	Calcineurin subunit B type 1	4	21	733.84837	63.828	2.89E-11	1	96.848	86.5	47211000
MCF_3	GNEASYPLEMCSHFDADEIKR	PPP3R1	Calcineurin subunit B type 1	4	21	733.84837	64.012	1.07E-17	1	75.064	72.136	55451000
MCF_1	GNHSGRPEDPEPGAFTTTK	SPECC1	Cytospin-B	4	19	616.06092	45.73	5.52E-07	1	94.313	89.965	21028000
MCF_2	GNHSGRPEDPEPGAFTTTK	SPECC1	Cytospin-B	4	19	616.06092	45.729	2.96E-06	1	69.081	64.464	12487000
MCF_3	GNHSGRPEDPEPGAFTTTK	SPECC1	Cytospin-B	4	19	616.06092	45.999	2.34E-09	1	106.75	106.75	15109000
MCF_1	GNIFANLFK	ARF1	ADP-ribosylation factor 1	2	9	743.93009	101.42	0.004763	2	155.07	112.47	36137000
MCF_1	GNIFANLFK	ARF1	ADP-ribosylation factor 1	3	9	496.28915	101.42	0.0079578	1	82.426	61.801	39570000
MCF_2	GNIFANLFK	ARF1	ADP-ribosylation factor 1	2	9	743.93009	101.45	0.0021676	2	128.36	88.01	30786000
MCF_2	GNIFANLFK	ARF1	ADP-ribosylation factor 1	3	9	496.28915	101.46	0.0067438	1	89.698	69.911	37500000
MCF_3	GNIFANLFK	ARF1	ADP-ribosylation factor 1	2	9	743.93009	101.56	8.96E-05	2	169.12	119.08	32871000
MCF_3	GNIFANLFK	ARF1	ADP-ribosylation factor 1	3	9	496.28915	101.56	0.0071504	1	88.294	67.669	57779000
MCF_1	GNIFGNLLK	ARF3	ADP-ribosylation factor 3	2	9	719.93009	94.688	0.004763	1	155.07	105.03	181730000
MCF_1	GNIFGNLLK	ARF3	ADP-ribosylation factor 3	3	9	480.28915	94.69	0.0079219	1	84.743	56.047	136730000
MCF_2	GNIFGNLLK	ARF3	ADP-ribosylation factor 3	3	9	480.28915	94.621	0.0079154	2	85.161	51.898	55626000
MCF_2	GNIFGNLLK	ARF3	ADP-ribosylation factor 3	2	9	719.93009	94.623	0.0052504	1	154.49	101.77	86543000
MCF_3	GNIFGNLLK	ARF3	ADP-ribosylation factor 3	2	9	719.93009	94.799	0.0031994	1	140.35	89.008	121730000
MCF_3	GNIFGNLLK	ARF3	ADP-ribosylation factor 3	3	9	480.28915	94.8	0.0063468	1	91.069	59.198	82755000
MCF_2	GNLFGR	CHMP6	Charged multivesicular body protein 6	2	6	563.82763	79.914	0.084087	1	103.41	50.82	26128000
MCF_1	GNQMSVPQR	BCAS1	Breast carcinoma-amplified sequence 1	3	9	499.26479	52.178	0.017018	1	56.432	35.911	1952200
MCF_3	GNQMSVPQR	BCAS1	Breast carcinoma-amplified sequence 1	3	9	499.26479	52.348	0.038638	1	48.423	34.844	3741000
MCF_3	GNQMSVPQR	BCAS1	Breast carcinoma-amplified sequence 1	3	9	493.93315	60.09	0.0047674	1	96.668	83.089	11132000
MCF_2	GNQVEK	FAM84B	Protein FAM84B	3	6	379.88401	53.502	0.043622	1	103.29	41.289	5215600
MCF_1	GNSASNIVSPQEALPGR	MSRA	Mitochondrial peptide methionine sulfoxide reductase	3	17	720.72218	67.925	2.44E-16	1	131.18	124.78	14405000
MCF_2	GNSASNIVSPQEALPGR	MSRA	Mitochondrial peptide methionine sulfoxide reductase	3	17	720.72218	68.069	3.22E-05	1	101.05	92.019	11879000
MCF_3	GNSASNIVSPQEALPGR	MSRA	Mitochondrial peptide methionine sulfoxide reductase	3	17	720.72218	68.221	0.0001028	1	79.393	73.852	16536000
MCF_1	GNSYAGQLK	GREB1;GREB1L	Protein GREB1;GREB1-like protein	2	9	700.88588	60.758	0.015367	1	84.054	47.497	5671200
MCF_1	GNSYAGQLK	GREB1;GREB1L	Protein GREB1;GREB1-like protein	3	9	467.59301	60.754	0.013642	1	66.27	40.444	9182700
MCF_2	GNSYAGQLK	GREB1;GREB1L	Protein GREB1;GREB1-like protein	2	9	700.88588	60.799	0.022772	1	69.275	49.499	4951700
MCF_3	GNSYAGQLK	GREB1;GREB1L	Protein GREB1;GREB1-like protein	3	9	467.59301	60.954	0.024865	1	58.433	43.769	5710500

MCF_1	GQQISDQTQLVINK	RNF141	RING finger protein 141	3	14	679.04801	70.039	1.36E-05	1	108.58	62.51	9628600
MCF_2	GQQISDQTQLVINK	RNF141	RING finger protein 141	3	14	679.04801	70.191	0.0006989	1	84.753	53.682	7642800
MCF_1	GQSQSGGHGPGGGK	PSMC1	26S protease regulatory subunit 4	3	14	558.6204	38.476	2.93E-10	1	127.32	121.32	428140000
MCF_2	GQSQSGGHGPGGGK	PSMC1	26S protease regulatory subunit 4	3	14	558.6204	38.686	1.51E-24	1	173.06	163.57	328220000
MCF_3	GQSQSGGHGPGGGK	PSMC1	26S protease regulatory subunit 4	3	14	558.6204	38.765	0.000195	2	104.09	86.86	275100000
MCF_1	GQSQSGGHGPGGGKK	PSMC1	26S protease regulatory subunit 4	4	15	451.24086	30.268	0.0003762	2	63.181	41.785	47720000
MCF_3	GQSQSGGHGPGGGKK	PSMC1	26S protease regulatory subunit 4	4	15	451.24086	30.254	0.00019	1	73.415	44.459	81750000
MCF_3	GSENSALK	SCYL3	Protein-associating with the carboxyl-terminal domain o	2	8	634.8515	57.278	0.083781	1	71.614	39.5	5457700
MCF_1	GSEQSSEAESRPNDLNSSVTPSPAK	LOH12CR1	Loss of heterozygosity 12 chromosomal region 1 protein	4	25	760.12599	50.039	3.99E-16	1	93.21	82.655	25525000
MCF_2	GSEQSSEAESRPNDLNSSVTPSPAK	LOH12CR1	Loss of heterozygosity 12 chromosomal region 1 protein	4	25	760.12599	50.324	6.80E-06	1	61.612	57.306	15314000
MCF_1	GSQHSAAAR	TMEM106C	Transmembrane protein 106C	3	9	449.91285	38.383	0.0009439	1	135.63	116	36956000
MCF_2	GSQHSAAAR	TMEM106C	Transmembrane protein 106C	3	9	449.91285	38.56	0.0010916	1	115.86	99.707	29073000
MCF_3	GSQHSAAAR	TMEM106C	Transmembrane protein 106C	3	9	449.91285	38.608	0.0022076	1	109.88	85.246	25061000
MCF_1	GSSQSVEIPGGGTEGYHVLR	GORASP2	Golgi reassembly-stacking protein 2	3	20	831.76634	61.932	1.21E-23	1	129.46	118.35	178450000
MCF_1	GSSQSVEIPGGGTEGYHVLR	GORASP2	Golgi reassembly-stacking protein 2	4	20	624.07657	61.931	1.12E-18	1	126.4	118.89	105150000
MCF_2	GSSQSVEIPGGGTEGYHVLR	GORASP2	Golgi reassembly-stacking protein 2	3	20	831.76634	62.09	1.21E-23	1	129.46	124.79	179680000
MCF_2	GSSQSVEIPGGGTEGYHVLR	GORASP2	Golgi reassembly-stacking protein 2	4	20	624.07657	62.089	5.42E-10	1	82.609	77.853	98841000
MCF_3	GSSQSVEIPGGGTEGYHVLR	GORASP2	Golgi reassembly-stacking protein 2	3	20	831.76634	62.195	9.75E-32	2	142.88	130.98	106960000
MCF_3	GSSQSVEIPGGGTEGYHVLR	GORASP2	Golgi reassembly-stacking protein 2	4	20	624.07657	62.198	2.32E-11	2	103.9	101.29	68645000
MCF_2	GTVHAR	SAMM50	Sorting and assembly machinery component 50 homolo	3	6	368.5526	46.299	0.080023	1	91.093	41.782	4322700
MCF_Myr1	GYEESEGHNTPKLKNQR	MORC4	MORC family CW-type zinc finger protein 4	3	17	817.42268	36.298	0.0048171	2	41.542	32.866	218980000
MCF_Myr2	GYEESEGHNTPKLKNQR	MORC4	MORC family CW-type zinc finger protein 4	3	17	817.42268	36.498	0.0095956	1	37.77	31.806	89394000
MCF_Myr2	GYEESEGHNTPKLKNQR	MORC4	MORC family CW-type zinc finger protein 4	3	17	817.42268	36.835	0.0003095	1	58.885	42.356	166150000
MCF_1	GYEESEGHNTPKLKNQR	MORC4	MORC family CW-type zinc finger protein 4	3	17	817.42268	36.193	0.0008911	1	51.03	41.987	92851000
MCF_1	GYEESEGHNTPKLKNQR	MORC4	MORC family CW-type zinc finger protein 4	3	17	817.42268	36.48	0.0008911	1	51.03	39.387	100590000
MCF_2	GYEESEGHNTPKLKNQR	MORC4	MORC family CW-type zinc finger protein 4	3	17	817.42268	36.644	0.0008911	1	51.03	41.556	185850000
MCF_3	GYEESEGHNTPKLKNQR	MORC4	MORC family CW-type zinc finger protein 4	3	17	817.42268	36.491	0.011082	1	36.597	30.46	83509000
MCF_3	GYEESEGHNTPKLKNQR	MORC4	MORC family CW-type zinc finger protein 4	3	17	817.42268	36.863	0.0008911	1	51.03	39.387	76800000

HEK293 PEAKS

Replicate	Sequence	Gene Name	-10LogP	Mass	ppm	m/z	RT	Scan						
Hek_1	C(+57.02)(+463.29)GNNMSTPLPAIVPAAR	LYPA1	47.09	2231.167	-2.8	744.7274	77.63	24290						
Hek_2	C(+57.02)(+463.29)GNNMSTPLPAIVPAAR	LYPA1	51.59	2231.167	-1.8	744.7282	77.59	24250						
Hek_3	C(+57.02)(+463.29)GNNMSTPLPAIVPAAR	LYPA1	65.09	2231.167	0.8	744.7301	77.52	24197						
Hek_1	C(+57.02)(+463.29)GNTMSVPLLTDAATVSGAER	LYPA2	67.6	2612.305	0.3	871.7759	84.11	26488						
Hek_2	C(+57.02)(+463.29)GNTMSVPLLTDAATVSGAER	LYPA2	50.22	2612.305	1.6	871.777	84.02	26436						
Hek_3	C(+57.02)(+463.29)GNTMSVPLLTDAATVSGAER	LYPA2	44.73	2612.305	0.6	871.7762	84.03	26376						
Hek_2	D(+463.29)(+57.02)AAAAK	BASP1	23.79	1065.593	-0.1	533.8038	56.32	16906						
Hek_1	G(+463.29)AAHSASEEVR	CHP3	55.81	1575.812	0.4	526.2781	45.9	13287						
Hek_2	G(+463.29)AAHSASEEVR	CHP3	59.1	1575.812	1.8	526.2788	45.85	13266						
Hek_3	G(+463.29)AAHSASEEVR	CHP3	59.48	1575.812	1.1	526.2784	45.89	13255						
Hek_1	G(+463.29)AFLDKPK	PPM1A, PPM1B	51.63	1337.782	-0.4	446.9344	67.47	20803						
Hek_2	G(+463.29)AFLDKPK	PPM1A, PPM1B	50.77	1337.782	0.7	446.9349	67.68	20850						
Hek_3	G(+463.29)AFLDKPK	PPM1A, PPM1B	51	1337.782	-0.1	446.9345	67.23	20683						
Hek_1	G(+463.29)AGSSTEQR	AKA12	46.93	1354.695	-1.6	452.5717	51.6	15223						
Hek_2	G(+463.29)AGSSTEQR	AKA12	42.69	1354.695	-0.1	452.5723	51.56	15215						
Hek_3	G(+463.29)AGSSTEQR	AKA12	37.71	1354.695	-0.6	452.5721	51.54	15181						
Hek_1	G(+463.29)AHLVR	NDUB7	44.26	1114.672	0.2	372.5648	54.03	16110						
Hek_2	G(+463.29)AHLVR	NDUB7	32.9	1114.672	-0.8	372.5644	53.98	16102						
Hek_3	G(+463.29)AHLVR	NDUB7	29.74	1114.672	-0.4	372.5646	53.87	16034						
Hek_1	G(+463.29)AQFSK	MARCS	30.49	1099.614	0.2	550.8143	62.78	19156						
Hek_2	G(+463.29)AQFSK	MARCS	26.6	1099.614	1.2	550.8148	63.01	19217						
Hek_3	G(+463.29)AQFSK	MARCS	33.57	1099.614	-0.1	550.8141	63.33	19326						
Hek_1	G(+463.29)ASSSSALAR	MARC2	53.75	1368.747	0.8	457.2567	58.61	17705						
Hek_2	G(+463.29)ASSSSALAR	MARC2	51.58	1368.747	0.4	457.2566	58.51	17674						
Hek_3	G(+463.29)ASSSSALAR	MARC2	52.33	1368.747	0.6	457.2567	58.47	17630						
Hek_1	G(+463.29)AYLSQ(+.98)PNTVK	PPM1G	30.56	1640.889	1.3	547.9708	73.45	22892						
Hek_1	G(+463.29)AYLSQPNTVK	PPM1G	59.11	1639.905	-0.7	820.959	72.15	22433						
Hek_2	G(+463.29)AYLSQPNTVK	PPM1G	60.21	1639.905	-0.4	820.9592	72.06	22383						
Hek_3	G(+463.29)AYLSQPNTVK	PPM1G	58	1639.905	-0.2	820.9594	72.08	22379						
Hek_1	G(+463.29)C(+57.02)C(+57.02)SSASSAAQSSK	CTL1	51.98	1849.841	1.3	617.6218	50.66	14883						
Hek_2	G(+463.29)C(+57.02)C(+57.02)SSASSAAQSSK	CTL1	48.08	1849.841	0.5	617.6213	50.56	14857						
Hek_3	G(+463.29)C(+57.02)C(+57.02)SSASSAAQSSK	CTL1	48.51	1849.841	2.5	617.6225	50.5	14804						
Hek_1	G(+463.29)C(+57.02)C(+57.02)YSSENEDSDQDREER	LTOR1	56.33	2698.098	-1.8	900.3718	51.04	15018						
Hek_2	G(+463.29)C(+57.02)C(+57.02)YSSENEDSDQDREER	LTOR1	57.04	2698.098	-1	675.5312	50.89	14976						
Hek_3	G(+463.29)C(+57.02)C(+57.02)YSSENEDSDQDREER	LTOR1	64.43	2698.098	-0.9	675.5312	50.86	14933						
Hek_1	G(+463.29)C(+57.02)FFSK	XRP2	28.56	1207.617	0.9	604.8164	80.76	25368						
Hek_2	G(+463.29)C(+57.02)FFSK	XRP2	27.61	1207.617	-0.4	604.8156	81.02	25430						
Hek_3	G(+463.29)C(+57.02)FFSK	XRP2	34.03	1207.617	-0.3	604.8157	80.98	25372						
Hek_1	G(+463.29)C(+57.02)GLNK	RFTN1	26.58	1110.597	1	556.3062	59	17847						
Hek_2	G(+463.29)C(+57.02)GLNK	RFTN1	27.65	1110.597	-1.5	556.3049	58.98	17836						
Hek_1	G(+463.29)C(+57.02)SSSALNK	ERIC5	30.87	1385.709	-0.7	693.861	56.39	16923						
Hek_2	G(+463.29)C(+57.02)SSSALNK	ERIC5	32.49	1385.709	2.7	462.9114	56.25	16881						
Hek_1	G(+463.29)C(+57.02)TLSAEDK	GNAI1, GNAI3	51.97	1442.719	0.1	481.9136	63.49	19407						
Hek_2	G(+463.29)C(+57.02)TLSAEDK	GNAI1, GNAI3	59.82	1442.719	-0.8	722.3661	63.32	19332						
Hek_3	G(+463.29)C(+57.02)TLSAEDK	GNAI1, GNAI3	53.71	1442.719	0.2	481.9136	63.28	19309						

Hek_1	G(+463.29)C(+57.02)TVSAEDK	GNAI2	44.27	1428.703	0	477.2416	58.72	17743						
Hek_2	G(+463.29)C(+57.02)TVSAEDK	GNAI2	45.01	1428.703	-0.8	477.2413	58.64	17720						
Hek_3	G(+463.29)C(+57.02)TVSAEDK	GNAI2	45.5	1428.703	0.9	477.2421	58.6	17677						
Hek_1	G(+463.29)C(+57.02)VQC(+57.02)K	FYN	34.2	1213.606	-0.3	607.8101	56.71	17041						
Hek_2	G(+463.29)C(+57.02)VQC(+57.02)K	FYN	41.67	1213.606	0.1	405.5426	56.83	17087						
Hek_3	G(+463.29)C(+57.02)VQC(+57.02)K	FYN	31.16	1213.606	-0.1	607.8102	56.61	16971						
Hek_1	G(+463.29)DVLSTHLDDAR	NIBL1	72.82	1760.917	0.3	587.9797	70.47	21846						
Hek_2	G(+463.29)DVLSTHLDDAR	NIBL1	68.1	1760.917	-1.6	587.9786	70.11	21705						
Hek_3	G(+463.29)DVLSTHLDDAR	NIBL1	68.63	1760.917	-0.3	587.9794	69.96	21638						
Hek_1	G(+463.29)DVLSTHLDDARR	NIBL1	38.88	1917.018	2	480.2628	62.48	19051						
Hek_1	G(+463.29)FIFSK	PLRKT	28.55	1160.671	0.4	581.3428	90.34	28439						
Hek_2	G(+463.29)FIFSK	PLRKT	34.13	1160.671	-1.1	581.3419	90.51	28464						
Hek_1	G(+463.29)GAVS(+79.97)AGEDNDELIDNLK	PCMD2	27	2359.106	0.6	787.3763	87.03	27420						
Hek_1	G(+463.29)GAVSAGEDNDDLIDNLK	PCMD1	75.76	2265.124	-0.2	756.0483	77.59	24277						
Hek_2	G(+463.29)GAVSAGEDNDDLIDNLK	PCMD1	63.71	2265.124	-0.6	756.048	77.58	24245						
Hek_3	G(+463.29)GAVSAGEDNDDLIDNLK	PCMD1	55.3	2265.124	-1.1	756.0476	77.5	24191						
Hek_1	G(+463.29)GAVSAGEDNDELIDNLK	PCMD2	79.2	2279.139	-0.3	760.7202	76.22	23814						
Hek_2	G(+463.29)GAVSAGEDNDELIDNLK	PCMD2	79.14	2279.139	-1	760.7197	76.19	23782						
Hek_3	G(+463.29)GAVSAGEDNDELIDNLK	PCMD2	84.67	2279.139	-1.1	760.7195	76.07	23724						
Hek_1	G(+463.29)GC(+57.02)AGSR	CLN3	30.09	1126.567	-0.8	564.2901	52.39	15511						
Hek_2	G(+463.29)GC(+57.02)AGSR	CLN3	26.25	1126.567	-0.2	564.2904	52.33	15494						
Hek_3	G(+463.29)GC(+57.02)AGSR	CLN3	24.21	1126.567	0.2	564.2906	52.32	15462						
Hek_1	G(+463.29)GSASSQLDEGK	NIBAN	72.59	1597.806	1.4	799.9113	57.5	17323						
Hek_2	G(+463.29)GSASSQLDEGK	NIBAN	59	1597.806	0	799.9102	57.55	17341						
Hek_3	G(+463.29)GSASSQLDEGK	NIBAN	60.65	1597.806	0	533.6093	57.38	17246						
Hek_1	G(+463.29)GSHSQTPR	TACC1	61.09	1388.727	0.5	463.9166	39.45	10961						
Hek_1	G(+463.29)GTTSTR	CHCH3	23.59	1141.62	0	571.8174	52.97	15720						
Hek_2	G(+463.29)GTTSTR	CHCH3	26.89	1141.62	-0.2	571.8173	52.91	15707						
Hek_1	G(+463.29)LC(+57.02)FPC(+57.02)PGESAPPTPDLEEK	SVIP	62.72	2663.272	-2.2	888.7627	83.15	26175						
Hek_2	G(+463.29)LC(+57.02)FPC(+57.02)PGESAPPTPDLEEK	SVIP	59.71	2663.272	-0.5	888.7642	83.15	26147						
Hek_3	G(+463.29)LC(+57.02)FPC(+57.02)PGESAPPTPDLEEK	SVIP	62.35	2663.272	0	888.7647	82.81	25980						
Hek_3	G(+463.29)LIFAK	ARL5B	23.08	1110.691	0.5	556.3532	87.52	27474						
Hek_1	G(+463.29)LSPSAPAVAVQASNASASPPSGC(+57.02)PMHEGK	CCHL	62.71	3324.634	-0.7	832.1653	63.45	19393						
Hek_2	G(+463.29)LSPSAPAVAVQASNASASPPSGC(+57.02)PMHEGK	CCHL	51.75	3324.634	0.9	1109.22	63.34	19338						
Hek_3	G(+463.29)LSPSAPAVAVQASNASASPPSGC(+57.02)PMHEGK	CCHL	88.81	3324.634	-1.6	832.1645	63.14	19258						
Hek_1	G(+463.29)LTISSLFSR	ARF4	49.81	1542.888	0.3	515.3035	96.11	30153						
Hek_2	G(+463.29)LTISSLFSR	ARF4	52.64	1542.888	0	515.3033	96.26	30161						
Hek_3	G(+463.29)LTISSLFSR	ARF4	46.31	1542.888	0.5	515.3036	96.19	30052						
Hek_1	G(+463.29)LTVSALFSR	ARF5	28.43	1512.878	1.3	505.3005	93.35	29342						
Hek_2	G(+463.29)LTVSALFSR	ARF5	43.14	1512.878	1.5	505.3006	93.3	29300						
Hek_3	G(+463.29)LTVSALFSR	ARF5	61.51	1512.878	0.9	505.3003	93.2	29174						
Hek_3	G(+463.29)N(+.98)IFANLFK	ARF1	24.22	1486.83	-0.6	496.6169	102.12	31753						
Hek_1	G(+463.29)NAAAAK	KAPCA	38.77	1064.609	0.8	533.3122	52.89	15691						
Hek_3	G(+463.29)NAAAAK	KAPCA	34.51	1064.609	1.2	533.3124	52.45	15511						
Hek_1	G(+463.29)NAATAK	KAPCB	36	1094.62	0.2	548.3172	52.58	15577						
Hek_2	G(+463.29)NAATAK	KAPCB	29.33	1094.62	0.4	548.3173	52.53	15569						
Hek_3	G(+463.29)NAATAK	KAPCB	29.4	1094.62	0.5	548.3174	52.49	15526						
Hek_2	G(+463.29)NAESQHVEHEFYGEK	TIAM1	50.54	2323.098	-1	581.7812	52.02	15381						
Hek_1	G(+463.29)NAGSMDSQQTDFR	FMNL2	55.24	1975.917	0	659.6462	63.28	19332						
Hek_2	G(+463.29)NAGSMDSQQTDFR	FMNL2	46.61	1975.917	-0.6	659.6459	63.16	19273						

Hek_2	G(+463.29)NC(+57.02)C(+57.02)WTQC(+57.02)FGLLR	AP1AR	29.53	2134.002	0	712.3414	84.7	26650							
Hek_1	G(+463.29)NEASYPLEM(+15.99)C(+57.02)SHFDADEIKR	CANB1	75.21	2947.359	0	737.847	58.87	17797							
Hek_2	G(+463.29)NEASYPLEM(+15.99)C(+57.02)SHFDADEIKR	CANB1	76.26	2947.359	-0.8	737.8465	58.85	17796							
Hek_3	G(+463.29)NEASYPLEM(+15.99)C(+57.02)SHFDADEIKR	CANB1	43.88	2947.359	-0.8	737.8465	58.78	17740							
Hek_1	G(+463.29)NEASYPLEMC(+57.02)SHFDADEIKR	CANB1	84.55	2931.364	0.1	733.8484	63.57	19437							
Hek_2	G(+463.29)NEASYPLEMC(+57.02)SHFDADEIKR	CANB1	86.53	2931.364	0.4	733.8486	63.81	19503							
Hek_3	G(+463.29)NEASYPLEMC(+57.02)SHFDADEIKR	CANB1	88.69	2931.364	0.2	733.8485	63.59	19417							
Hek_1	G(+463.29)NHSGRPEDPEPGAFTTTK	CYTSB	52.34	2460.215	0.8	616.0614	45.83	13259							
Hek_2	G(+463.29)NHSGRPEDPEPGAFTTTK	CYTSB	62.33	2460.215	-0.3	616.0607	45.82	13258							
Hek_3	G(+463.29)NHSGRPEDPEPGAFTTTK	CYTSB	80.61	2460.215	-0.3	616.0607	45.92	13265							
Hek_1	G(+463.29)NIFANLFK	ARF1	52.54	1485.846	-0.2	743.9299	101.51	31669							
Hek_2	G(+463.29)NIFANLFK	ARF1	42.1	1485.846	0.1	496.2892	101.11	31516							
Hek_3	G(+463.29)NIFANLFK	ARF1	63.32	1485.846	-0.5	743.9297	101.33	31504							
Hek_1	G(+463.29)NIFGNLLK	ARF3	41.89	1437.846	-0.2	480.289	94.43	29668							
Hek_2	G(+463.29)NIFGNLLK	ARF3	40.13	1437.846	0.9	480.2896	94.39	29623							
Hek_3	G(+463.29)NIFGNLLK	ARF3	43.37	1437.846	-0.5	719.9297	94.57	29589							
Hek_1	G(+463.29)NLFGR	CHMP6	25.25	1125.641	0.4	563.8279	80.01	25107							
Hek_1	G(+463.29)NLLGGVSFR	OGRL1	22.5	1481.847	0.1	494.9562	87.83	27673							
Hek_3	G(+463.29)NLLGGVSFR	OGRL1	48.08	1481.847	0.7	494.9565	87.69	27527							
Hek_1	G(+463.29)NLLK	FA49B	21.81	1006.629	-0.1	504.3216	75.46	23553							
Hek_1	G(+463.29)NQHPSISR	BAG5	22.73	1457.785	0.2	486.9358	45.58	13169							
Hek_2	G(+463.29)NQHPSISR	BAG5	26.16	1457.785	-0.2	486.9355	45.65	13194							
Hek_1	G(+463.29)NQVEK	FA84B	24.69	1136.63	-0.9	379.8837	53.53	15930							
Hek_2	G(+463.29)NQVEK	FA84B	28.72	1136.63	0	569.3224	53.52	15933							
Hek_3	G(+463.29)NQVEK	FA84B	25	1136.63	0	569.3224	53.36	15850							
Hek_3	G(+463.29)NSALR	LRC57	25	1079.62	1.3	540.818	59.07	17846							
Hek_1	G(+463.29)NSASNIVSPQEALPGR	MSRA	85.97	2159.145	2.4	720.7239	67.93	20955							
Hek_3	G(+463.29)NSASNIVSPQEALPGR	MSRA	82.07	2159.145	2.4	720.7239	67.81	20881							
Hek_2	G(+463.29)NTLGLAPM(+15.99)GTLPR	TM40L	42.75	1876.035	-0.6	626.352	75.37	23498							
Hek_3	G(+463.29)NTLGLAPM(+15.99)GTLPR	TM40L	43.02	1876.035	0.4	626.3526	75.3	23461							
Hek_3	G(+463.29)NTLGLAPMGTLPR	TM40L	48.28	1860.04	0.1	621.0208	81.9	25690							
Hek_2	G(+463.29)NTLTC(+57.02)C(+57.02)VSPNAS(+79.97)PK	CCYL1	67.07	2147.986	-1.3	717.0016	75.3	23469							
Hek_3	G(+463.29)NTLTC(+57.02)C(+57.02)VSPNAS(+79.97)PK	CCYL1	46.24	2147.986	1.8	717.0038	75.21	23432							
Hek_3	G(+463.29)NTSSER	AAKB1	26.39	1212.621	-0.8	405.214	51.2	15056							
Hek_1	G(+463.29)NTSSER	AAKB1	22.49	1212.621	0.5	405.2145	51.27	15101							
Hek_2	G(+463.29)NTTSC(+57.02)C(+57.02)VSSS(+79.97)PK	CCNY	36.95	1926.833	-0.3	643.2847	63.31	19326							
Hek_3	G(+463.29)NTTSC(+57.02)C(+57.02)VSSS(+79.97)PK	CCNY	56.36	1926.833	1.1	643.2856	63.25	19296							
Hek_2	G(+463.29)QC(+57.02)VTK	DCNL3	30.66	1154.623	0	578.3188	55.93	16768							
Hek_1	G(+463.29)QQISDQTQLVINK	RN141	47.55	2034.122	-1.5	679.047	69.92	21655							
Hek_2	G(+463.29)QQISDQTQLVINK	RN141	63.49	2034.122	0.8	1018.069	70.07	21690							
Hek_3	G(+463.29)QQISDQTQLVINK	RN141	67.71	2034.122	-0.6	679.0476	69.82	21586							
Hek_2	G(+463.29)QQPGK	ABL1	26.68	1076.609	0.3	539.312	51.07	15042							
Hek_3	G(+463.29)QQPGK	ABL1	28.46	1076.609	2.1	539.3129	51.05	15002							
Hek_1	G(+463.29)QQPGK	ABL1	25.37	1076.609	-0.1	539.3117	51.16	15062							
Hek_1	G(+463.29)QSQSGGHGPGGGK	PRS4	66.74	1672.839	0.1	558.6204	38.81	10723							
Hek_2	G(+463.29)QSQSGGHGPGGGK	PRS4	62.92	1672.839	0.1	558.6204	38.68	10688							
Hek_3	G(+463.29)QSQSGGHGPGGGK	PRS4	71.65	1672.839	0.2	558.6205	38.67	10665							
Hek_1	G(+463.29)QSQSGGHGPGGGKK	PRS4	43.89	1800.934	-0.2	451.2408	31.13	7924							
Hek_2	G(+463.29)QSQSGGHGPGGGKK	PRS4	42.3	1800.934	0.9	451.2412	30.93	7871							
Hek_3	G(+463.29)QSQSGGHGPGGGKK	PRS4	37.82	1800.934	0.4	451.2411	30.62	7741							

Hek_1	G(+463.29)QTSVSTLSPQPGSVDGLDK	PHAR2	72.45	2435.266	-0.6	812.762	69.58	21533							
Hek_2	G(+463.29)QTSVSTLSPQPGSVDGLDK	PHAR2	58.15	2435.266	0.8	812.7631	69.55	21505							
Hek_3	G(+463.29)QTSVSTLSPQPGSVDGLDK	PHAR2	71.14	2435.266	-1.6	812.7612	69.53	21480							
Hek_3	G(+463.29)SENSALK	X6RHX1	29.56	1267.689	0.3	634.8517	57.15	17164							
Hek_1	G(+463.29)SNSSR	DYM	25.14	1069.563	0.4	535.7889	49.16	14415							
Hek_1	G(+463.29)SQHSAAAR	T106C	24.17	1346.717	-0.5	449.9126	38.78	10712							
Hek_3	G(+463.29)SQHSAAAR	T106C	22.83	1346.717	-0.1	449.9128	38.65	10656							
Hek_1	G(+463.29)SQSSK	MRP	26.87	1055.572	-0.8	528.793	48.4	14133							
Hek_2	G(+463.29)SQSSK	MRP	30.52	1055.572	0.1	528.7935	48.68	14245							
Hek_1	G(+463.29)SSQSVEIPGGGTEGYHVLR	GORS2	84.16	2492.277	0.7	624.077	62.05	18901							
Hek_2	G(+463.29)SSQSVEIPGGGTEGYHVLR	GORS2	78.98	2492.277	0.3	624.0767	61.96	18854							
Hek_3	G(+463.29)SSQSVEIPGGGTEGYHVLR	GORS2	101.54	2492.277	0.5	831.7667	62.15	18913							
Hek_3	G(+463.29)STDSK	HID1	35.25	1056.556	1.4	529.2862	52.16	15406							
Hek_1	G(+463.29)STESSEGR	CHCH6	30.53	1371.674	0.1	686.8444	52.98	15724							
Hek_2	G(+463.29)STESSEGR	CHCH6	30.42	1371.674	-0.4	686.8441	52.96	15726							
Hek_3	G(+463.29)STESSEGR	CHCH6	48.66	1371.674	-0.3	458.2319	52.87	15670							
Hek_1	G(+463.29)STLGC(+57.02)HR	EEPD1	55.99	1349.699	-0.1	450.9068	52.77	15647							
Hek_2	G(+463.29)STLGC(+57.02)HR	EEPD1	61.79	1349.699	-1.9	450.906	52.71	15635							
Hek_3	G(+463.29)STLGC(+57.02)HR	EEPD1	64.45	1349.699	-0.8	450.9065	52.63	15576							
Hek_1	G(+463.29)TVHAR	SAM50	28.7	1102.636	-0.6	368.5524	46.37	13460							
Hek_2	G(+463.29)TVHAR	SAM50	29.56	1102.636	0.6	368.5528	46.37	13460							
Hek_3	G(+463.29)TVHAR	SAM50	32.21	1102.636	-0.4	368.5525	46.37	13429							
Hek_1	N(+463.29)LFSR	GANAB	22.66	1098.63	0.8	550.3226	80.36	25230							
Hek_2	S(+463.29)YQDKTIIMWK	D6R9Z1	26.74	1875.008	1	938.512	47.44	13792							

HeLa PEAKS

Replicate	Sequence	Gene Name	-10LogP	Mass	ppm	m/z	RT	Scan
HeLa_1	C(+57.02)(+463.29)GNNMSTPLPAIVPAAR	LYPA1	47.98	2231.167	-0.5	744.7292	77.52	24826
HeLa_2	C(+57.02)(+463.29)GNNMSTPLPAIVPAAR	LYPA1	73.32	2231.167	0.5	744.7299	77.64	24795
HeLa_3	C(+57.02)(+463.29)GNNMSTPLPAIVPAAR	LYPA1	64.28	2231.167	0.4	744.7299	77.57	24560
HeLa_1	C(+57.02)(+463.29)GNTMSVPLLTDAATVSGAER	LYPA2	59.88	2612.305	1.4	871.7769	84.11	27092
HeLa_2	C(+57.02)(+463.29)GNTMSVPLLTDAATVSGAER	LYPA2	80.22	2612.305	1.9	871.7773	84.05	27004
HeLa_3	C(+57.02)(+463.29)GNTMSVPLLTDAATVSGAER	LYPA2	70.4	2612.305	0.7	871.7762	84.11	26785
HeLa_1	G(+463.29)AAGSSALAR	MARC1	52.48	1322.742	-1.6	441.9205	61	19058
HeLa_2	G(+463.29)AAGSSALAR	MARC1	58.38	1322.742	-0.2	441.9211	60.94	18996
HeLa_3	G(+463.29)AAGSSALAR	MARC1	58.06	1322.742	1.1	441.9217	61.02	18840
HeLa_1	G(+463.29)AAHSASEEVR	CHP3	63.55	1575.812	-0.3	526.2777	45.79	13655
HeLa_2	G(+463.29)AAHSASEEVR	CHP3	74.2	1575.812	-0.9	526.2774	45.38	13442
HeLa_3	G(+463.29)AAHSASEEVR	CHP3	79.85	1575.812	-0.8	526.2775	45.57	13377
HeLa_1	G(+463.29)AFLDKPK	PPM1A, PPM1B	47.86	1337.782	-0.3	446.9344	67.16	21246
HeLa_2	G(+463.29)AFLDKPK	PPM1A, PPM1B	52.59	1337.782	0.8	446.9349	67.18	21191
HeLa_3	G(+463.29)AFLDKPK	PPM1A, PPM1B	54.09	1337.782	1	446.935	67.51	21114
HeLa_1	G(+463.29)AGSSTEQR	AKA12	48.42	1354.695	1.6	452.5731	51.51	15659
HeLa_2	G(+463.29)AGSSTEQR	AKA12	50.13	1354.695	-0.1	452.5723	51.37	15559
HeLa_3	G(+463.29)AGSSTEQR	AKA12	61.27	1354.695	-0.4	452.5722	51.46	15436
HeLa_1	G(+463.29)AHLVR	NDUB7	32.28	1114.672	-1	372.5644	53.71	16478
HeLa_2	G(+463.29)AHLVR	NDUB7	38.19	1114.672	-0.7	372.5645	53.54	16364
HeLa_3	G(+463.29)AHLVR	NDUB7	31.21	1114.672	-0.2	372.5647	53.73	16266
HeLa_1	G(+463.29)AQFSK	MARCS	25.93	1099.614	0.6	550.8145	63.72	20033
HeLa_2	G(+463.29)AQFSK	MARCS	30.11	1099.614	0.4	550.8144	62.58	19573
HeLa_3	G(+463.29)AQFSK	MARCS	34.44	1099.614	0.2	550.8143	62.68	19422
HeLa_1	G(+463.29)ASSSSALAR	MARC2	48.01	1368.747	0.3	457.2565	58.46	18173
HeLa_2	G(+463.29)ASSSSALAR	MARC2	54.63	1368.747	1.9	457.2572	58.41	18103
HeLa_3	G(+463.29)ASSSSALAR	MARC2	52.43	1368.747	0.4	457.2566	58.44	17933
HeLa_1	G(+463.29)AYLS(+79.97)QPNTVK	PPM1G	27.17	1719.871	1	574.2982	81.38	26167
HeLa_2	G(+463.29)AYLS(+79.97)QPNTVK	PPM1G	44.55	1719.871	0.9	574.2981	81.49	26139
HeLa_1	G(+463.29)AYLSQ(+.98)PNTVK	PPM1G	32.15	1640.889	0.2	547.9702	73.36	23422
HeLa_2	G(+463.29)AYLSQ(+.98)PNTVK	PPM1G	40.76	1640.889	0.3	547.9703	73.49	23409
HeLa_1	G(+463.29)AYLSQPNTVK	PPM1G	57.62	1639.905	0.4	820.9598	71.89	22902
HeLa_2	G(+463.29)AYLSQPNTVK	PPM1G	55.9	1639.905	0.2	547.6422	71.79	22804
HeLa_3	G(+463.29)AYLSQPNTVK	PPM1G	71.85	1639.905	0.8	547.6426	72.13	22724
HeLa_2	G(+463.29)C(+57.02)APSIHISER	PDE8A	31.05	1688.878	2.4	563.968	58.15	18007
HeLa_3	G(+463.29)C(+57.02)APSIHISER	PDE8A	32.91	1688.878	2	563.9678	58.2	17849
HeLa_1	G(+463.29)C(+57.02)C(+57.02)SSASSAAQSSK	CTL1	48.21	1849.841	1.8	617.6221	50.54	15305
HeLa_1	G(+463.29)C(+57.02)C(+57.02)YSSENEDSDQDREER	LTOR1	50.69	2698.098	0	675.5319	50.88	15430
HeLa_2	G(+463.29)C(+57.02)C(+57.02)YSSENEDSDQDREER	LTOR1	56.19	2698.098	-0.8	675.5313	50.78	15338
HeLa_3	G(+463.29)C(+57.02)C(+57.02)YSSENEDSDQDREER	LTOR1	54.27	2698.098	0.9	675.5325	50.75	15180
HeLa_1	G(+463.29)C(+57.02)FFSK	XRP2	35.64	1207.617	-0.5	604.8156	81.01	26036
HeLa_2	G(+463.29)C(+57.02)FFSK	XRP2	31.86	1207.617	0.5	604.8162	81.03	25978
HeLa_3	G(+463.29)C(+57.02)FFSK	XRP2	34.11	1207.617	-0.1	604.8158	80.71	25636
HeLa_1	G(+463.29)C(+57.02)GLNK	RFTN1	34.99	1110.597	-1.1	556.3051	58.8	18294
HeLa_2	G(+463.29)C(+57.02)GLNK	RFTN1	33.54	1110.597	-2.1	556.3045	58.76	18230
HeLa_1	G(+463.29)C(+57.02)TLSAED(+57.02)K(+57.02)AAVER	GNAI1, GNAI3	36.35	2083.048	-3.2	695.3544	56.06	17309

HeLa_1	G(+463.29)C(+57.02)TLSAEDK	GNAI1, GNAI3	53.06	1442.719	-0.4	722.3663	63.24	19863
HeLa_2	G(+463.29)C(+57.02)TLSAEDK	GNAI1, GNAI3	52.62	1442.719	0.4	481.9137	63.38	19856
HeLa_3	G(+463.29)C(+57.02)TLSAEDK	GNAI1, GNAI3	52.73	1442.719	0.2	481.9136	63.39	19671
HeLa_1	G(+463.29)C(+57.02)TLSAEER	GNAO	46.86	1484.741	0.9	495.9212	63.76	20050
HeLa_2	G(+463.29)C(+57.02)TLSAEER	GNAO	47.71	1484.741	0.1	495.9208	63.74	19987
HeLa_3	G(+463.29)C(+57.02)TLSAEER	GNAO	59.32	1484.741	0.3	495.921	63.83	19829
HeLa_1	G(+463.29)C(+57.02)TVSAEDK	GNAI2	46.94	1428.703	-1	715.3582	58.66	18249
HeLa_2	G(+463.29)C(+57.02)TVSAEDK	GNAI2	46.92	1428.703	0.6	477.2419	58.56	18156
HeLa_3	G(+463.29)C(+57.02)TVSAEDK	GNAI2	56.82	1428.703	0.2	715.3589	58.65	18013
HeLa_1	G(+463.29)DVLSTHLDDAR	NIBL1	62.72	1760.917	0.7	587.98	70.25	22333
HeLa_2	G(+463.29)DVLSTHLDDAR	NIBL1	64.8	1760.917	1	587.9802	70.38	22313
HeLa_3	G(+463.29)DVLSTHLDDAR	NIBL1	81.47	1760.917	0.2	587.9797	70.31	22091
HeLa_1	G(+463.29)DVLSTHLDDARR	NIBL1	50.28	1917.018	0.4	480.262	62.01	19425
HeLa_2	G(+463.29)DVLSTHLDDARR	NIBL1	59.88	1917.018	0	480.2618	62.04	19384
HeLa_3	G(+463.29)DVLSTHLDDARR	NIBL1	68.13	1917.018	-0.1	480.2617	62.41	19329
HeLa_2	G(+463.29)ETMSK	LANC2	26.05	1114.58	-0.4	558.2973	59.31	18423
HeLa_1	G(+463.29)FIFSK	PLRKT	24.55	1160.671	-0.9	581.342	90.47	29186
HeLa_2	G(+463.29)FIFSK	PLRKT	30.66	1160.671	0.2	581.3427	90.46	29126
HeLa_1	G(+463.29)GAVSAGEDNDDLIDNLK	PCMD1	59.9	2265.124	-0.7	756.0479	77.61	24854
HeLa_2	G(+463.29)GAVSAGEDNDDLIDNLK	PCMD1	73.65	2265.124	-0.3	756.0482	77.66	24803
HeLa_3	G(+463.29)GAVSAGEDNDDLIDNLK	PCMD1	72.09	2265.124	0	756.0485	77.67	24591
HeLa_1	G(+463.29)GAVSAGEDNDELIDNLK	PCMD2	69.45	2279.139	-1.5	760.7193	76.34	24430
HeLa_2	G(+463.29)GAVSAGEDNDELIDNLK	PCMD2	74.77	2279.139	0.7	760.7209	76.33	24357
HeLa_3	G(+463.29)GAVSAGEDNDELIDNLK	PCMD2	50.19	2279.139	-0.7	760.7198	76.32	24145
HeLa_1	G(+463.29)GC(+57.02)AGSR	CLN3	27.29	1126.567	-0.2	564.2904	52.16	15901
HeLa_1	G(+463.29)GSASSQLDEGK	NIBAN	60.52	1597.806	-1.6	799.9089	57.42	17803
HeLa_2	G(+463.29)GSASSQLDEGK	NIBAN	53.61	1597.806	0.4	533.6094	57.22	17695
HeLa_3	G(+463.29)GSASSQLDEGK	NIBAN	60.74	1597.806	2.6	799.9123	57.46	17591
HeLa_1	G(+463.29)GSHSQTPR	TACC1	57.09	1388.727	-0.3	463.9162	38.96	11126
HeLa_2	G(+463.29)GSHSQTPR	TACC1	60.81	1388.727	-0.6	463.9161	38.89	11023
HeLa_3	G(+463.29)GSHSQTPR	TACC1	68.63	1388.727	-0.2	463.9163	39.13	11040
HeLa_1	G(+463.29)GTTSTR	CHCH3	37.61	1141.62	-0.9	381.5471	52.91	16181
HeLa_2	G(+463.29)GTTSTR	CHCH3	41.23	1141.62	1.4	381.5479	52.84	16102
HeLa_3	G(+463.29)GTTSTR	CHCH3	41.89	1141.62	1.2	381.5479	52.97	15985
HeLa_2	G(+463.29)LC(+57.02)FPC(+57.02)PGESAPPTPDLEEK	SVIP	40.35	2663.272	-0.7	888.7641	82.92	26623
HeLa_3	G(+463.29)LC(+57.02)FPC(+57.02)PGESAPPTPDLEEK	SVIP	53.9	2663.272	-0.3	888.7644	83	26414
HeLa_2	G(+463.29)LIFAK	ARL5B	24.83	1110.691	1.2	556.3536	87.24	28073
HeLa_1	G(+463.29)LSPSAPAVAVQASNASASPPSGC(+57.02)PM(+15.99)HEGK	CCHL	49.31	3340.629	-1.8	836.163	61.63	19287
HeLa_2	G(+463.29)LSPSAPAVAVQASNASASPPSGC(+57.02)PM(+15.99)HEGK	CCHL	30.87	3340.629	-0.6	836.1641	61.65	19240
HeLa_3	G(+463.29)LSPSAPAVAVQASNASASPPSGC(+57.02)PM(+15.99)HEGK	CCHL	58.26	3340.629	0.3	836.1648	61.71	19077
HeLa_1	G(+463.29)LSPSAPAVAVQASNASASPPSGC(+57.02)PMHEGK	CCHL	73.76	3324.634	-1.2	832.1649	63.08	19805
HeLa_2	G(+463.29)LSPSAPAVAVQASNASASPPSGC(+57.02)PMHEGK	CCHL	93.9	3324.634	0.4	832.1661	63.2	19788
HeLa_3	G(+463.29)LSPSAPAVAVQASNASASPPSGC(+57.02)PMHEGK	CCHL	92.23	3324.634	-1.4	1109.217	63.22	19612
HeLa_1	G(+463.29)LTISSLFSR	ARF4	44.25	1542.888	0.1	515.3034	96.25	30956
HeLa_2	G(+463.29)LTISSLFSR	ARF4	46.98	1542.888	-1.2	772.4504	96.28	30970
HeLa_3	G(+463.29)LTISSLFSR	ARF4	56.09	1542.888	1.4	515.3041	96.59	30740
HeLa_1	G(+463.29)LTVSALFSR	ARF5	54.82	1512.878	0.2	505.2999	93.28	30053
HeLa_2	G(+463.29)LTVSALFSR	ARF5	63.35	1512.878	0.6	505.3001	93.48	30094
HeLa_3	G(+463.29)LTVSALFSR	ARF5	62.16	1512.878	0.7	505.3002	93.6	29829
HeLa_1	G(+463.29)NAAAAK	KAPCA	40.16	1064.609	0.4	533.312	52.74	16120

HeLa_2	G(+463.29)NAAAAK	KAPCA	36.57	1064.609	0.4	533.312	52.24	15883
HeLa_3	G(+463.29)NAAAAK	KAPCA	39.51	1064.609	1.8	533.3127	52.78	15912
HeLa_1	G(+463.29)NAATAK	KAPCB	34.71	1094.62	2.1	548.3182	52.38	15985
HeLa_2	G(+463.29)NAATAK	KAPCB	33.74	1094.62	0.3	548.3173	52.28	15896
HeLa_1	G(+463.29)NAGSMDSQQTDFR	FMNL2	42	1975.917	1.6	659.6473	63.17	19839
HeLa_3	G(+463.29)NAGSMDSQQTDFR	FMNL2	39.74	1975.917	0.9	659.6469	63.16	19591
HeLa_1	G(+463.29)NAQERPSETIDR	NOL3	30.34	1934.992	0.3	646.0049	47.93	14403
HeLa_2	G(+463.29)NAQERPSETIDR	NOL3	34.29	1934.992	0.1	646.0048	47.92	14341
HeLa_2	G(+463.29)NC(+57.02)C(+57.02)WTQC(+57.02)FGLLR	AP1AR	41	2134.002	-0.5	712.341	84.66	27213
HeLa_3	G(+463.29)NC(+57.02)C(+57.02)WTQC(+57.02)FGLLR	AP1AR	48.74	2134.002	-0.5	712.341	84.78	27005
HeLa_1	G(+463.29)NEASYPLEM(+15.99)C(+57.02)SHFDADEIKR	CANB1	66.6	2947.359	1.1	737.8479	58.86	18316
HeLa_2	G(+463.29)NEASYPLEM(+15.99)C(+57.02)SHFDADEIKR	CANB1	72.81	2947.359	-2.3	737.8453	58.65	18193
HeLa_3	G(+463.29)NEASYPLEM(+15.99)C(+57.02)SHFDADEIKR	CANB1	72.29	2947.359	-0.6	737.8466	58.56	17976
HeLa_2	G(+463.29)NEASYPLEMC(+57.02)SHFDADEIK	CANB1	42.99	2775.263	2.4	694.8247	70.85	22473
HeLa_1	G(+463.29)NEASYPLEMC(+57.02)SHFDADEIKR	CANB1	78.19	2931.364	-0.4	733.848	63.59	19987
HeLa_2	G(+463.29)NEASYPLEMC(+57.02)SHFDADEIKR	CANB1	90.77	2931.364	0	733.8483	63.39	19857
HeLa_3	G(+463.29)NEASYPLEMC(+57.02)SHFDADEIKR	CANB1	104.62	2931.364	0.1	733.8484	63.74	19798
HeLa_1	G(+463.29)NHSGRPEDPEPGAFTTTK	CYTSB	81.28	2460.215	-0.7	616.0605	45.65	13605
HeLa_2	G(+463.29)NHSGRPEDPEPGAFTTTK	CYTSB	74.25	2460.215	-1.3	616.0601	45.37	13441
HeLa_1	G(+463.29)NIFANLFK	ARF1	43.31	1485.846	-1	743.9293	101.46	32451
HeLa_2	G(+463.29)NIFANLFK	ARF1	48.47	1485.846	0	496.2891	101.4	32481
HeLa_Myr2	G(+463.29)NIFANLFK	ARF1	46.29	1485.846	1	496.2896	101.23	30487
HeLa_3	G(+463.29)NIFANLFK	ARF1	55.75	1485.846	-0.4	743.9297	101.46	32147
HeLa_1	G(+463.29)NIFGNLLK	ARF3	49.53	1437.846	0	480.2891	94.63	30474
HeLa_2	G(+463.29)NIFGNLLK	ARF3	45.96	1437.846	0	719.9301	94.57	30439
HeLa_3	G(+463.29)NIFGNLLK	ARF3	52.95	1437.846	0.4	480.2893	94.74	30181
HeLa_2	G(+463.29)NLESAEGVPGEPPSVPLLLPPGK	F8W1F5	50.41	2816.544	-2.5	939.8528	88.44	28476
HeLa_1	G(+463.29)NLFGR	CHMP6	34	1125.641	0.5	563.8279	79.92	25658
HeLa_2	G(+463.29)NLLGGVSFR	OGRL1	62.18	1481.847	-1.1	494.9556	87.73	28237
HeLa_3	G(+463.29)NLLGGVSFR	OGRL1	36.13	1481.847	0.8	494.9566	87.71	27967
HeLa_2	G(+463.29)NLLK	FA49B	21.38	1006.629	0	504.3216	75.18	23964
HeLa_2	G(+463.29)NSALR	LRC57	21.54	1079.62	1.7	540.8182	59.07	18339
HeLa_1	G(+463.29)NSASNIVSPQEALPGR	MSRA	75.5	2159.145	0.5	720.7226	67.93	21509
HeLa_2	G(+463.29)NSASNIVSPQEALPGR	MSRA	93.71	2159.145	0.4	720.7225	67.84	21416
HeLa_3	G(+463.29)NSASNIVSPQEALPGR	MSRA	94.24	2159.145	-0.4	720.7219	67.86	21230
HeLa_1	G(+463.29)NTLGLAPM(+15.99)GTLPR	TM40L	42.75	1876.035	-0.1	626.3523	75.43	24117
HeLa_3	G(+463.29)NTLGLAPM(+15.99)GTLPR	TM40L	73.27	1876.035	-1.3	626.3516	75.27	23789
HeLa_1	G(+463.29)NTLGLAPMGTLPR	TM40L	49.73	1860.04	0.5	621.0211	81.99	26374
HeLa_2	G(+463.29)NTLGLAPMGTLPR	TM40L	75.05	1860.04	0.3	621.0209	82.04	26321
HeLa_3	G(+463.29)NTLGLAPMGTLPR	TM40L	67.98	1860.04	0.2	621.0209	82.02	26088
HeLa_1	G(+463.29)NTTTK	AKIB1	27.79	1083.604	-0.8	542.8087	51.08	15501
HeLa_1	G(+463.29)QAGC(+57.02)K	P2RX5	32.91	1082.566	0.2	542.2902	50.82	15408
HeLa_3	G(+463.29)QAGC(+57.02)K	P2RX5	37.41	1082.566	2	542.2911	50.75	15177
HeLa_2	G(+463.29)QC(+57.02)VTK	DCNL3	24.69	1154.623	3.6	578.3209	55.7	17134
HeLa_1	G(+463.29)QQISDQTQLVINK	RN141	63.42	2034.122	0.6	679.0484	69.92	22216
HeLa_2	G(+463.29)QQISDQTQLVINK	RN141	68.88	2034.122	-0.6	679.0476	70.03	22196
HeLa_3	G(+463.29)QQISDQTQLVINK	RN141	78.61	2034.122	0.2	679.0482	70.01	21989
HeLa_1	G(+463.29)QQPGK	ABL1	27.16	1076.609	0.3	539.312	51.07	15498
HeLa_2	G(+463.29)QQPGK	ABL1	26.81	1076.609	-0.3	539.3116	50.93	15396
HeLa_3	G(+463.29)QQPGK	ABL1	29.61	1076.609	0	539.3118	51	15265

HeLa_2	G(+463.29)QSQ(+.98)SGGHGPGGGK	PRS4	36.87	1673.823	-0.6	558.9481	39.95	11420
HeLa_1	G(+463.29)QSQSGGHGPGGGK	PRS4	59.19	1672.839	1	558.621	38.33	10886
HeLa_2	G(+463.29)QSQSGGHGPGGGK	PRS4	63.27	1672.839	-0.8	558.6199	38.35	10814
HeLa_3	G(+463.29)QSQSGGHGPGGGK	PRS4	78.39	1672.839	-0.3	558.6202	38.55	10823
HeLa_1	G(+463.29)QSQSGGHGPGGGKK	PRS4	55.18	1800.934	-0.5	451.2406	29.79	7696
HeLa_2	G(+463.29)QSQSGGHGPGGGKK	PRS4	51.34	1800.934	-0.2	451.2408	30.01	7704
HeLa_3	G(+463.29)QSQSGGHGPGGGKK	PRS4	56.45	1800.934	0.4	601.319	30.18	7738
HeLa_1	G(+463.29)SEQSSEAESRPNDLNSSVTPSPAK	L12R1	69.84	3036.475	-0.2	760.1259	50.06	15128
HeLa_2	G(+463.29)SEQSSEAESRPNDLNSSVTPSPAK	L12R1	63.22	3036.475	-0.8	760.1254	50.09	15083
HeLa_3	G(+463.29)SEQSSEAESRPNDLNSSVTPSPAK	L12R1	66.12	3036.475	0.1	760.126	50.1	14939
HeLa_3	G(+463.29)SILSR	MGRN1	29.04	1094.656	0.3	548.3354	75.02	23701
HeLa_2	G(+463.29)SNSSR	DYM	25.75	1069.563	0.4	535.7889	49.08	14775
HeLa_2	G(+463.29)SQHSAAAR	T106C	24.35	1346.717	-1.1	449.9124	38.28	10790
HeLa_1	G(+463.29)SQSSK	MRP	31.29	1055.572	0.9	528.7939	48.45	14595
HeLa_2	G(+463.29)SQSSK	MRP	29.6	1055.572	0.6	528.7938	48.07	14396
HeLa_1	G(+463.29)SSQSVEIPGGGTEGYHVLR	GORS2	75.54	2492.277	1.6	624.0776	61.86	19369
HeLa_2	G(+463.29)SSQSVEIPGGGTEGYHVLR	GORS2	85.26	2492.277	-0.2	831.7662	61.82	19303
HeLa_3	G(+463.29)SSQSVEIPGGGTEGYHVLR	GORS2	81.93	2492.277	0.4	624.0768	61.88	19137
HeLa_1	G(+463.29)STWGSPGWVR	VKOR1	53.14	1651.858	0.1	551.6268	77.17	24717
HeLa_3	G(+463.29)STWGSPGWVR	VKOR1	56.04	1651.858	-1.3	551.626	77.21	24447
HeLa_1	G(+463.29)TFC(+57.02)SVIK	ZDHC6	23.92	1373.749	-2	687.8804	75.86	24261
HeLa_2	G(+463.29)VGAGR	J3KQ73	23.3	978.5723	-0.5	490.2932	59.38	18450
HeLa_2	G(+463.29)VNQSVGFPPVTGPHLVGC(+57.02)GDVMEGQNLQGSFFR	PAFA2	50.86	4049.999	-3	1013.504	81.98	26300
HeLa_3	G(+463.29)VQPPNFSWVLPGR	DUS23	45.8	2016.106	-1	673.0419	91.94	29309
HeLa_1	N(+463.29)LFSR	E9PKU7	23.38	1098.63	0.3	550.3223	80.25	25772
HeLa_2	N(+463.29)LFSR	E9PKU7	26.55	1098.63	-0.6	550.3218	79.94	25591

MCF7 PEAKS

Replicate	Sequence	Gene Name	-10LogP	Mass	ppm	m/z	RT	Scan
MCF_1	C(+57.02)(+463.29)GNNMSTPLPAIVPAAR	LYPA1	54.26	2231.167	-1.8	744.7282	77.73	23983
MCF_3	C(+57.02)(+463.29)GNNMSTPLPAIVPAAR	LYPA1	55.57	2231.167	0.4	744.7299	77.84	24009
MCF_2	C(+57.02)(+463.29)GNNMSTPLPAIVPAAR	LYPA1	46.97	2231.167	0	744.7296	77.71	23967
MCF_2	C(+57.02)(+463.29)GNTMSVPLLTDAATVSGAER	LYPA2	54.56	2612.305	2.9	871.7782	84.32	26160
MCF_1	G(+463.29)AFLDKPK	PPM1A, PPM1B	53.02	1337.782	0.2	446.9346	67.07	20390
MCF_3	G(+463.29)AFLDKPK	PPM1A, PPM1B	47.29	1337.782	0.2	446.9347	67.4	20496
MCF_2	G(+463.29)AFLDKPK	PPM1A, PPM1B	51.26	1337.782	-0.1	446.9345	67.63	20582
MCF_1	G(+463.29)AHLVR	NDUB7	30.1	1114.672	-0.3	372.5646	53.65	15773
MCF_3	G(+463.29)AHLVR	NDUB7	23.9	1114.672	0.3	372.5648	53.87	15837
MCF_2	G(+463.29)AHLVR	NDUB7	27.94	1114.672	0.3	372.5648	53.79	15836
MCF_1	G(+463.29)AQFSK	MARCS	26.37	1099.614	0.8	550.8146	62.94	18943
MCF_3	G(+463.29)AQFSK	MARCS	26.87	1099.614	0.6	550.8145	63.22	19040
MCF_2	G(+463.29)AQFSK	MARCS	29.06	1099.614	1	550.8147	62.7	18883
MCF_1	G(+463.29)ASSSSALAR	MARC2	62.78	1368.747	-0.8	457.256	58.48	17416
MCF_3	G(+463.29)ASSSSALAR	MARC2	55.88	1368.747	-0.5	457.2562	58.64	17460
MCF_2	G(+463.29)ASSSSALAR	MARC2	51.33	1368.747	-1.7	457.2556	58.65	17489
MCF_3	G(+463.29)AYLS(+79.97)QPNTVK	PPM1G	37.03	1719.871	1	574.2982	81.8	25350
MCF_1	G(+463.29)AYLSQPNTVK	PPM1G	64.86	1639.905	0.1	820.9597	72.01	22092
MCF_3	G(+463.29)AYLSQPNTVK	PPM1G	61.95	1639.905	0.5	820.96	72.2	22150
MCF_2	G(+463.29)AYLSQPNTVK	PPM1G	46.67	1639.905	1.6	547.643	71.9	22059
MCF_1	G(+463.29)C(+57.02)C(+57.02)SSASSAAQSSK	CTL1	46.27	1849.841	-2.6	617.6194	50.4	14602
MCF_3	G(+463.29)C(+57.02)C(+57.02)SSASSAAQSSK	CTL1	52.76	1849.841	1.4	617.6218	50.7	14697
MCF_1	G(+463.29)C(+57.02)C(+57.02)YSSENEDSDQDREER	LTOR1	67.5	2698.098	-0.3	675.5317	50.87	14769
MCF_3	G(+463.29)C(+57.02)C(+57.02)YSSENEDSDQDREER	LTOR1	50.92	2698.098	-0.3	675.5317	51.06	14824
MCF_2	G(+463.29)C(+57.02)C(+57.02)YSSENEDSDQDREER	LTOR1	47.96	2698.098	0.2	675.532	50.89	14782
MCF_1	G(+463.29)C(+57.02)GLNK	RFTN1	29.05	1110.597	0.3	556.3058	58.81	17531
MCF_3	G(+463.29)C(+57.02)GLNK	RFTN1	30.86	1110.597	0	556.3057	58.99	17582
MCF_2	G(+463.29)C(+57.02)GLNK	RFTN1	26	1110.597	-0.8	556.3052	58.88	17575
MCF_1	G(+463.29)C(+57.02)TLSAEDK	GNAI1, GNAI3	52.76	1442.719	-0.2	481.9134	63.3	19074
MCF_3	G(+463.29)C(+57.02)TLSAEDK	GNAI1, GNAI3	52	1442.719	0.2	481.9136	63.52	19145
MCF_2	G(+463.29)C(+57.02)TLSAEDK	GNAI1, GNAI3	50.11	1442.719	-0.2	481.9135	63.4	19128
MCF_1	G(+463.29)C(+57.02)TVSAEDK	GNAI2	44.91	1428.703	-1.4	715.3578	58.69	17489
MCF_3	G(+463.29)C(+57.02)TVSAEDK	GNAI2	49.02	1428.703	-0.3	477.2415	58.78	17511
MCF_2	G(+463.29)C(+57.02)TVSAEDK	GNAI2	48.24	1428.703	-0.5	477.2414	58.69	17505
MCF_1	G(+463.29)DVLSTHLDDAR	NIBL1	83.78	1760.917	0.1	587.9796	70.3	21504
MCF_3	G(+463.29)DVLSTHLDDAR	NIBL1	71.2	1760.917	-0.1	587.9795	70.55	21579
MCF_2	G(+463.29)DVLSTHLDDAR	NIBL1	86.09	1760.917	0.1	587.9796	70.45	21560
MCF_1	G(+463.29)DVLSTHLDDARR	NIBL1	65.26	1917.018	1.1	480.2623	62.05	18637
MCF_3	G(+463.29)DVLSTHLDDARR	NIBL1	61.46	1917.018	-0.6	480.2615	62.29	18720
MCF_2	G(+463.29)DVLSTHLDDARR	NIBL1	79.57	1917.018	-0.2	480.2617	62.18	18706
MCF_1	G(+463.29)FIFSK	PLRKT	31.31	1160.671	-0.5	581.3422	90.61	28171
MCF_3	G(+463.29)GAVSAGEDNDDLIDNLK	PCMD1	76.91	2265.124	0.6	756.0489	77.87	24021
MCF_2	G(+463.29)GAVSAGEDNDDLIDNLK	PCMD1	71.05	2265.124	0.8	756.0491	77.76	23984
MCF_1	G(+463.29)GAVSAGEDNDELIDNLK	PCMD2	76.23	2279.139	-0.8	760.7198	76.34	23528
MCF_3	G(+463.29)GAVSAGEDNDELIDNLK	PCMD2	73.93	2279.139	-0.5	760.72	76.55	23590
MCF_2	G(+463.29)GAVSAGEDNDELIDNLK	PCMD2	69.11	2279.139	0	760.7204	76.4	23544

MCF_3	G(+463.29)GLFSR	LNP	26.33	1098.63	0.3	550.3223	80.47	24899
MCF_1	G(+463.29)GTTSTR	CHCH3	24.68	1141.62	-1.2	571.8168	52.85	15482
MCF_2	G(+463.29)GTTSTR	CHCH3	25.65	1141.62	0.4	571.8177	52.9	15510
MCF_3	G(+463.29)LSPSAPAVAVQASNASASPPSGC(+57.02)PMHEGK	CCHL	25.62	3324.634	-4.9	832.1618	63.57	19162
MCF_1	G(+463.29)LTISSLFSR	ARF4	50.91	1542.888	0.5	515.3036	96.36	29905
MCF_3	G(+463.29)LTISSLFSR	ARF4	47.21	1542.888	0.8	515.3038	96.53	29961
MCF_2	G(+463.29)LTISSLFSR	ARF4	49.88	1542.888	1	515.3038	96.05	29761
MCF_1	G(+463.29)LTVSALFSR	ARF5	58.19	1512.878	-0.1	505.2998	93.71	29118
MCF_3	G(+463.29)LTVSALFSR	ARF5	59.1	1512.878	-0.1	505.2998	93.79	29158
MCF_2	G(+463.29)LTVSALFSR	ARF5	58.08	1512.878	-0.4	505.2996	93.66	29060
MCF_1	G(+463.29)NAAAAK	KAPCA	37.58	1064.609	0.1	533.3118	52.8	15463
MCF_3	G(+463.29)NAAAAK	KAPCA	42	1064.609	-0.3	533.3116	52.56	15365
MCF_2	G(+463.29)NAAAAK	KAPCA	28.48	1064.609	-0.3	533.3116	52.79	15468
MCF_1	G(+463.29)NAATAK	KAPCB	34.72	1094.62	0.8	548.3175	52.46	15341
MCF_3	G(+463.29)NAATAK	KAPCB	33.1	1094.62	1.1	548.3177	52.61	15380
MCF_2	G(+463.29)NAATAK	KAPCB	34.63	1094.62	0.6	548.3174	52.5	15366
MCF_1	G(+463.29)NAESQHVEHEFYGEK	TIAM1	62.53	2323.098	0.1	581.7819	52.07	15196
MCF_1	G(+463.29)NAQERPSETIDR	NOL3	43.47	1934.992	0.5	646.005	47.89	13772
MCF_3	G(+463.29)NAQERPSETIDR	NOL3	60.84	1934.992	-1.1	484.7548	48.55	14000
MCF_2	G(+463.29)NAQERPSETIDR	NOL3	37.36	1934.992	0.6	646.0051	48.16	13870
MCF_3	G(+463.29)NC(+57.02)C(+57.02)WTQC(+57.02)FGLLR	AP1AR	39.24	2134.002	-1.2	712.3405	84.99	26392
MCF_1	G(+463.29)NEASYPLEM(+15.99)C(+57.02)SHFDADEIKR	CANB1	83.47	2947.359	0.6	737.8475	58.77	17519
MCF_3	G(+463.29)NEASYPLEM(+15.99)C(+57.02)SHFDADEIKR	CANB1	67.72	2947.359	0.6	737.8475	58.82	17524
MCF_2	G(+463.29)NEASYPLEMC(+57.02)SHFDADEIKR	CANB1	74.09	2931.364	-0.4	733.848	63.71	19235
MCF_1	G(+463.29)NHSGRPEDPEPGAFTTTK	CYTSB	68.46	2460.215	-1.2	616.0602	45.66	13013
MCF_3	G(+463.29)NHSGRPEDPEPGAFTTTK	CYTSB	71.7	2460.215	-0.4	616.0607	45.97	13119
MCF_1	G(+463.29)NIFANLFK	ARF1	46.05	1485.846	-0.7	743.9296	101.53	31365
MCF_3	G(+463.29)NIFANLFK	ARF1	53.09	1485.846	0.1	743.9301	101.67	31430
MCF_2	G(+463.29)NIFANLFK	ARF1	52.33	1485.846	0.7	496.2895	101.56	31335
MCF_2	G(+463.29)NIFGN(+.98)LLK	ARF3	26.68	1438.83	-0.7	480.6168	97.55	30187
MCF_1	G(+463.29)NIFGNLLK	ARF3	51.26	1437.846	-0.2	480.289	94.79	29447
MCF_3	G(+463.29)NIFGNLLK	ARF3	46.27	1437.846	-0.1	719.93	94.87	29475
MCF_2	G(+463.29)NIFGNLLK	ARF3	48.79	1437.846	0.1	480.2892	94.76	29385
MCF_2	G(+463.29)NLM(+15.99)GK	RPB1	30.37	1097.602	-2.6	549.8066	96.21	29810
MCF_1	G(+463.29)NQM(+15.99)SVPQR	BCAS1	50.86	1494.773	0.4	499.265	52.17	15233
MCF_3	G(+463.29)NQM(+15.99)SVPQR	BCAS1	55.24	1494.773	1.5	499.2656	52.38	15297
MCF_3	G(+463.29)NQMSVPQR	BCAS1	58.21	1478.778	2.2	493.9342	60.07	17951
MCF_1	G(+463.29)NQVEK	FA84B	22.91	1136.63	-0.7	569.322	53.25	15629
MCF_3	G(+463.29)NQVEK	FA84B	29.8	1136.63	-0.7	379.8838	53.49	15701
MCF_2	G(+463.29)NQVEK	FA84B	34.71	1136.63	0.3	569.3226	53.4	15692
MCF_3	G(+463.29)NSALR	LRC57	31.69	1079.62	0.5	540.8176	59.36	17713
MCF_1	G(+463.29)NSASNIVSPQEALPGR	MSRA	62.45	2159.145	1.1	720.723	67.84	20645
MCF_3	G(+463.29)NSASNIVSPQEALPGR	MSRA	48.66	2159.145	0.1	720.7223	68.1	20731
MCF_2	G(+463.29)NSASNIVSPQEALPGR	MSRA	68.87	2159.145	-1	720.7215	67.93	20680
MCF_1	G(+463.29)NSYAGQLK	GREB1	55.03	1399.757	0.4	467.5932	60.7	18186
MCF_3	G(+463.29)NSYAGQLK	GREB1	54.1	1399.757	1.5	467.5937	60.87	18238
MCF_2	G(+463.29)NSYAGQLK	GREB1	53.21	1399.757	0.9	467.5934	60.72	18209
MCF_3	G(+463.29)NTTTK	AKIB1	26.32	1083.604	0.1	542.8091	51.24	14888
MCF_1	G(+463.29)QC(+57.02)VTK	DCNL3	30.05	1154.623	1.4	578.3196	55.69	16449
MCF_3	G(+463.29)QC(+57.02)VTK	DCNL3	29.25	1154.623	-0.1	578.3187	56.05	16570

MCF_1	G(+463.29)QQISDQTQLVINK	RN141	73.54	2034.122	0	679.048	70.02	21413
MCF_3	G(+463.29)QQISDQTQLVINK	RN141	56.49	2034.122	0.1	679.0481	70.32	21503
MCF_2	G(+463.29)QQISDQTQLVINK	RN141	75.44	2034.122	1.1	679.0488	70.18	21469
MCF_1	G(+463.29)QQPGK	ABL1	29.19	1076.609	2.2	539.313	51.09	14849
MCF_3	G(+463.29)QQPGK	ABL1	30.41	1076.609	0.1	539.3118	51.31	14912
MCF_2	G(+463.29)QQPGK	ABL1	23.37	1076.609	1.3	539.3125	51.07	14850
MCF_1	G(+463.29)QSQSGGHGPGGGK	PRS4	71.11	1672.839	-0.1	558.6204	38.38	10406
MCF_3	G(+463.29)QSQSGGHGPGGGK	PRS4	63.97	1672.839	-0.3	558.6202	38.6	10485
MCF_2	G(+463.29)QSQSGGHGPGGGK	PRS4	67.64	1672.839	0.2	558.6205	38.57	10505
MCF_1	G(+463.29)QSQSGGHGPGGGKK	PRS4	49.38	1800.934	0.1	451.2409	30.44	7539
MCF_3	G(+463.29)QSQSGGHGPGGGKK	PRS4	55.86	1800.934	-0.7	451.2405	30.16	7455
MCF_2	G(+463.29)QSQSGGHGPGGGKK	PRS4	47.14	1800.934	0.2	451.241	30.16	7472
MCF_1	G(+463.29)SEQSSEAESRPNDLNSSVTPSPAK	L12R1	75.88	3036.475	0.5	760.1264	50.07	14485
MCF_2	G(+463.29)SEQSSEAESRPNDLNSSVTPSPAK	L12R1	48.12	3036.475	1.7	760.1273	50.39	14598
MCF_3	G(+463.29)SILSR	MGRN1	26.51	1094.656	-1.6	548.3344	75.24	23159
MCF_3	G(+463.29)SNSSR	DYM	24.54	1069.563	-1.1	535.7881	49.26	14257
MCF_1	G(+463.29)SQHSAAAR	T106C	35.98	1346.717	-0.4	449.9127	38.32	10385
MCF_2	G(+463.29)SQHSAAAR	T106C	24.02	1346.717	0.2	449.9129	38.48	10472
MCF_3	G(+463.29)SQSSK	MRP	27.08	1055.572	0.3	528.7936	48.44	13959
MCF_1	G(+463.29)SSQSVEIPGGGTEGYHVLR	GORS2	71.76	2492.277	0.5	624.0768	61.85	18567
MCF_3	G(+463.29)SSQSVEIPGGGTEGYHVLR	GORS2	67.45	2492.277	-0.8	624.076	62.07	18639
MCF_2	G(+463.29)SSQSVEIPGGGTEGYHVLR	GORS2	71.04	2492.277	-0.1	624.0765	61.98	18638
MCF_1	G(+463.29)STDSK	HID1	22.96	1056.556	0.2	529.2856	51.92	15145
MCF_3	G(+463.29)STDSK	HID1	27.19	1056.556	0.7	529.2858	52.17	15220
MCF_2	G(+463.29)STDSK	HID1	24.46	1056.556	2.4	529.2867	52.01	15184
MCF_1	G(+463.29)TVHAR	SAM50	25.03	1102.636	-2.2	368.5518	46.15	13198
MCF_3	G(+463.29)TVHAR	SAM50	25.53	1102.636	-0.4	368.5525	46.38	13270
MCF_2	G(+463.29)TVHAR	SAM50	27.53	1102.636	-0.3	368.5525	46.23	13238

Table S5: Label-free quantification of YnMyr-tagged proteins in Hek, HeLa and MCF cells

Sheet 1 : Summary

The table is showing a total number of protein IDs found in all 3 cell lines, the corresponding number of MG proteins and their percentage based on the number of requested valid values (1 valid value in any YnMyr treated sample). In addition, significance (both total and MG proteins) as well as number of proteins with PTM peptide (both MQ and PEAKS analysis) is indicated per cell line.

Sheet 2 : Total protein IDs

Cells were treated for 24 h with 20 µM YnMyr or 20 µM Myr. Cells were lysed, tagged proteins were captured with reagent **2**, enriched, digested with trypsin and analyzed by LC-MS/MS. The data were analyzed with MaxQuant (version 1.5.0.25) and Perseus (version 1.5.0.9). The data are filtered (1 valid value in any YnMyr treated samples) and are displayed without any additional processing (total protein IDs). Colunm MG proteins indicates where the MG signature is present, column t-test significance shows proteins significantly enriched in YnMyr experiments (modified t-test FDR = 0.01 and s0 = 2) and column PTM peptides indicates if such peptides were found for a given ID with either MaxQuant or PEAKS software. The column t-test parameters gives the value for the standard t-test and t-test difference (difference between logarithmized label-free intensities (corresponds to fold change)). Protein grouping feature was enabled in MaxQuant, therefore when unique peptides could not be assigned to one unique protein they were assigned instead to a group of closely related proteins. Proteins are ordered by gene names.

<u>Note</u>: due to the database (human complete fasta file, uniprot) redundancy, protein TACC1 appears as 2 entries from the reviewed and not-reviewed part of human proteome

cell line	# total proteins	# MG proteins	% MG proteins	# proteins t-test significant	# MG prteins t-test significant	# proteins with PTM peptide MQ	# proteins with PTM peptide PEAKS	# proteins with PTM peptide PEAKS + MQ
**HEK 293**	2050	403	20%	452	170	46	65	65
**HeLa**				439	163	50	65	69
**MCF7**				375	145	37	50	50

											t-test parameters					
		t-test Significance			PTM peptides MQ			PTM peptides PEAKS			Hek		HeLa		MCF	
Gene names	MG protein	HEK	HeLa	MCF	Hek	HeLa	MCF	Hek	HeLa	MCF	-Log t-test p value	t-test Difference	-Log t-test p value	t-test Difference	-Log t-test p value	t-test Difference
A4GALT											0.03779518	-0.5611331	0.011303	-0.5249602	0.3312715	0.070319494
AAMDC											0.33319613	0.03290558	0.1513455	-0.28447978	0.0441461	-1.043377558
AARS											0.08683495	-0.1312466	0.00306573	-0.37020556	0.0050972	-0.232659658
AASDHPPT											0.78915779	0.60988935	0.3607466	0.0852534	0.2105162	-0.229929606
ABAT											0.41060999	0.21350225	0.06294529	-0.56878853	1.2743692	0.140023549
ABCB6		+	+	+							2.51846523	2.17844327	1.97493433	3.54628944	4.2610581	5.350556056
ABCB7			+								2.01668754	1.42354139	3.6771481	2.10581907	0.1527921	-0.510014216
ABCC1;ABCC3											0.09790453	-0.4363314	2.0152303	1.08059247	0.9597226	0.686009725
ABCC4											1.14254426	1.27296448	0.77522384	0.63726362	0.1724958	-0.319731394
ABCD1											0.04069515	-0.6984615	0.36076533	0.11235619	0.0507972	-0.920393626
ABCD3			+								0.36346794	0.14136759	2.11120601	2.22678693	0.1963078	-0.106569926
ABCE1											0.13430307	-0.2084382	0.00790288	-0.28253746	0.4846606	0.141005198
ABCF2											0.03127708	-0.723629	0.41355254	0.26607768	0.0721214	-0.857228597
ABHD10											0.39772121	0.07745171	0.06682489	-0.78973325	0.1709399	-0.313048681
ABHD11											0.03762576	-0.6718108	0.29249435	-0.0098877	0.5592532	0.559690475
ABHD16A											0.31263745	0.02279218	0.0569471	-0.66030121	0.0886571	-0.686218898
ABHD17A		+		+							4.59905307	5.16623751	0.79306307	0.72044945	4.3650788	4.223765691
ABHD17B		+	+	+							4.57258776	5.92467626	5.63074505	5.15748533	4.9033066	5.044546127
ABHD17C		+		+							3.23508948	2.46668879	0.02701588	-1.03730647	5.5930599	5.616771062
ABL1	+	+	+	+				+	+	+	3.5821083	4.20324707	3.2633333	4.3977108	2.6205713	3.772357941
ABL2	+	+									3.59518725	2.67057864	2.19231681	1.53854243	0.0015564	-1.196763357
ACAA1											0.28563036	-0.0324949	0.42277773	0.18865013	0.6748671	0.712481181
ACAA2	+	+	+	+							5.86160164	4.49472491	2.75793442	5.81632868	2.8912364	3.166614532
ACACA											3.09E-05	-2.6281732	1.06E-06	-2.7948335	2.33E-05	-1.887030919
ACAD9		+									2.91749066	2.01844088	2.44659882	0.71169853	1.1600877	1.539813995
ACADM											0.40805532	0.08297157	0.75499371	1.03061231	0.0742848	-0.806451797
ACADSB	+										0.17690165	-0.11947	0.0866882	-0.70150312	0.0884865	-0.450357437
ACADVL		+									2.01200812	1.82968903	1.82594991	1.40077337	2.6450748	0.441076914
ACAT1											3.27548826	1.07087199	5.54928406	1.08341471	0.2581844	-0.183478673
ACAT2	+										1.00257748	0.74224663	2.32881881	1.27367846	1.79125	1.48833847
ACBD3											0.3367442	0.07033157	0.10543087	-0.30371475	0.113476	-0.609753927
ACLY											0.00601838	-0.6025861	0.00011452	-0.61633937	0.0133945	-0.385114034
ACO2											0.60205575	0.10882187	0.39860848	0.0295016	0.3826549	0.034912745
ACOT7											0.40137315	0.24141502	0.49230358	0.42163658	1.0515849	1.647788366
ACOT9		+	+								4.16195352	2.63827578	1.44326927	2.7130324	0.1098409	-0.372526805
ACOX1			+								2.28208327	1.30496152	3.93374293	2.12990061	1.4198941	1.520145416
ACP1											0.82722201	1.11647606	0.10859621	-0.2356987	0.1841421	-0.233605067
ACP2	+										0.40968154	0.15308825	0.1884141	-0.1504701	0.2748549	-0.014357885
ACSL1											0.05849031	-0.8244101	1.54332704	1.08121173	0.5154971	0.509389242
ACSL3		+									3.07401978	2.95485751	1.93752685	0.56092898	0.8889345	1.078093211
ACSL4											0.24259999	-0.1085726	0.28084819	-0.04958534	0.1276913	-0.546410243
ACTA1;ACTC1;ACTG2											0.91020736	2.78590965	0.12925892	-0.35713132	0.7327842	2.003770192
ACTA2;ACTG2											0.03155706	-4.0550105	7.22E-05	-7.60238393	0.0225373	-5.089892069
ACTG1;ACTB											0.09886468	-0.1348972	0.00085763	-0.30806732	0.1123903	-0.028399785
ACTN1	+	+									3.63486779	2.39097659	0.08269597	-0.27518972	0.0041176	-0.648585637
ACTN4	+										0.01180997	-0.2466113	0.00089945	-0.60375277	0.0117668	-0.206798553

ACTR1A;ACTR1B		+									3.41171562	3.21907489	0.17133825	-0.20082347	0.0926217	-0.554255803
ACTR2			+								0.04184564	-0.5391655	3.66261391	3.75649516	0.0863443	-0.770856222
ACTR3	+										1.69150502	0.30212021	0.01103966	-0.2398421	0.140853	-0.101759593
ACVR1											0.04178751	-0.7460721	0.0187313	-0.78632355	0.2319626	-0.123052597
ADAM10											0.32016253	0.02200317	0.0058428	-0.868131	0.357489	0.092336019
ADCY3	+										1.03171003	1.07664045	0.13382119	-0.28169378	0.3783873	0.180404027
ADH5		+									3.58575577	3.2057635	1.18629723	2.54511261	0.9537672	1.948115667
ADK											1.16867254	0.90585772	0.34962014	0.02340253	0.744367	0.155429204
ADO											0.71512361	0.71778234	0.4581077	0.24833934	0.028619	-0.919577281
ADRA2A	+										0.75102429	0.51227252	0.09314466	-0.38628197	0.0564388	-0.854913076
ADSL											0.13150838	-0.3412507	0.79219882	1.68784587	0.3204758	0.051373164
ADSS											0.92991507	0.71315384	1.28726742	0.16664696	0.548149	0.27428627
AGK											0.07559452	-0.2391033	0.71314714	0.23944092	0.1271487	-0.540903727
AGPAT1		+									4.66583218	3.89548556	1.75256436	1.88934517	0.9033381	0.779717127
AGPAT2											0.15778177	-0.3570296	0.3281636	0.06346893	0.0409863	-0.960228602
AGPS											0.96403692	0.52269681	1.39802651	1.38353666	0.4600552	0.201131821
AGTRAP											0.42563564	0.20112864	0.08659901	-0.33880107	0.5470724	0.45731926
AHCY											0.43537172	0.027469	0.03398114	-0.11959902	0.0181622	-0.201853434
AHNAK											0.34235019	0.01591746	0.00201202	-1.10939916	8.09E-05	-0.538436254
AHSA1											0.07747924	-0.6564331	0.14672972	-0.32125791	0.9473781	1.031533559
AIFM1	+										0.22235706	-0.0833708	0.24671856	-0.137537	1.470929	0.538801193
AIFM2	+	+	+	+							3.48094241	5.31787046	4.69355266	8.21990522	4.9316053	6.841719309
AIG1			+								1.67214873	1.68521563	4.82712823	1.86149979	2.2154007	1.265858332
AIMP2		+									3.17049792	2.00898679	1.25090114	1.82202593	1.1544534	1.665159225
AIP											0.83048224	0.29723549	0.44774441	0.05027135	0.1880054	-0.388310115
AK1	+										0.16441934	-0.4793282	0.60089589	0.33287303	0.0061998	-1.042487462
AK2				+							2.32398896	1.06859271	1.48600972	0.91336886	2.5792574	2.332227071
AK3	+										0.63681337	0.46514893	0.38968967	0.24018542	0.2334923	-0.111087163
AKAP12	+	+	+		+	+		+	+		5.09952845	8.00682386	5.16564277	9.6329422	0.4058556	0.581253052
AKR1A1											1.1265133	0.59428406	0.7171578	0.11212985	0.685627	0.335892359
AKR1B1											0.12226331	-0.2950281	0.93189827	0.54662704	0.1528409	-0.344774882
AKR1C1			+								0.04329158	-0.8882262	2.05555504	2.46470896	0.5066365	0.213303884
AKR1C2;AKR1C1											0.12211955	-0.2139734	1.75695206	1.42903392	0.4101968	0.063658396
AKR1C3	+										0.14452063	-0.23504	0.22853801	-0.03821437	0.5459737	0.430688858
AKR7A2											0.67426271	0.65697352	1.5093127	1.21331533	0.168482	-0.143351237
ALDH16A1	+										0.38033336	0.1441377	0.17067897	-0.15158653	0.6495772	0.440104167
ALDH18A1		+									3.0303686	3.30190595	0.76353838	0.57643763	0.1812217	-0.215716044
ALDH1B1			+								0.10459487	-0.3650462	2.11507312	1.97068787	0.0824301	-0.364927928
ALDH2											3.76100996	1.01730537	2.83430715	0.77359772	0.0020372	-1.09768041
ALDH3B1;ALDH3B2			+								0.0834763	-0.426541	2.89531191	3.12592697	0.1118952	-0.355981191
ALDH5A1		+									3.4435309	3.21243604	0.22315945	-0.18491554	0.1959877	-0.160112381
ALDH6A1											0.24563637	-0.1397807	1.01087537	0.58960787	0.8187657	1.476306915
ALDH7A1	+										2.01162639	0.63645426	1.40492113	0.33595594	2.6247305	0.763038635
ALDH9A1											0.14986625	-0.5150172	1.89518939	1.31728554	0.4032226	0.068404516
ALDOA											0.33587878	0.01361911	0.00205344	-0.23762385	0.3861571	0.01717186
ALDOC		+									3.65593935	3.40244865	1.48835074	0.85483487	1.6588982	0.486457189
ALG1		+									1.97514772	2.01589139	3.02748646	1.34171677	1.8667466	1.391681671
ALG10B;ALG10											1.82065323	1.72195943	0.09551159	-0.79177666	0.2181382	-0.131493251
ALG3											0.96787656	0.40882111	0.58545284	0.27941322	0.1857824	-0.237945557
ALG6											0.62858023	0.92624982	0.78301049	0.19107755	0.0809811	-0.696163813

ALG8			+								0.79733867	0.57342911	2.33806909	2.33338674	2.0371835	1.156042099
ALPL			+								0.19804456	-0.206192	3.42305365	4.73187828	0.1784369	-0.190820694
AMIGO2			+	+							0.35401677	0.08543841	5.00146143	4.67135493	3.4571218	2.113826752
ANKIB1	+	+	+	+		+			+	+	2.43693112	2.90562884	3.00663212	4.37430318	3.2000106	3.132746379
ANKRD22	+			+							0.04994945	-0.4718634	0.58271076	0.64222463	4.0487929	3.067455292
ANO3											0.52707331	1.41812706	0.33848849	0.33605448	0.0623113	-3.771570841
ANO6		+	+	+							3.32397555	3.14084752	2.90808319	2.60281754	3.4130332	1.773375193
ANP32A											0.76034195	1.06804276	2.4769501	0.55224927	0.7590078	0.179554621
ANP32B											0.55143425	0.23555374	2.00773956	1.21767044	1.4006558	0.425900777
ANTXR1			+								1.50224307	1.27154477	2.93050185	3.82054075	0.0464421	-0.597076416
ANTXR2											0.11681461	-0.5419019	0.66655675	0.4533062	0.0995454	-0.142831802
ANXA1											1.7393112	1.0753816	4.06230915	1.30679321	0.3638217	0.205343246
ANXA11											3.09546324	1.19143105	2.58541718	0.4390138	0.5139822	0.10696284
ANXA2;ANXA2P2	+	+									4.07985278	1.53248914	4.06251092	0.91006152	3.2533836	0.691138585
ANXA3		+	+								2.07520428	2.03106562	2.88408807	1.89944267	1.5264387	1.038192113
ANXA4											1.23378276	0.54059474	0.50068691	0.20359802	0.5006883	0.075057348
ANXA5		+	+	+							4.78836422	3.68685722	5.50361056	3.13207054	6.4574826	2.228404363
ANXA6											2.13289974	0.34934235	1.42202669	0.23539607	1.4578002	0.070748647
ANXA7		+									1.39583432	2.11675199	0.0780871	-0.126894	1.1320669	0.226024628
AP1AR	+	+	+	+			+	+	+	+	4.80234452	4.0037988	4.07215041	3.41833433	2.4474349	3.49740092
AP1B1											0.38635562	0.1821874	0.60848625	0.35180728	1.8388087	0.723636627
AP1G1											1.95272118	1.1408062	0.93835721	1.5605011	2.9284757	1.116622925
AP1M1;AP1M2											0.10279395	-0.5479495	1.04816715	0.4515241	1.0377949	0.638807933
AP2A1;AP2A2											0.45663235	0.39217822	0.70570491	0.90629005	0.8542315	0.943045934
AP2B1											0.68000137	0.24187724	1.33484425	0.35191536	0.3052569	0.000996908
AP3D1											1.52147932	1.61499977	0.72446834	0.18504969	0.6780566	0.352433523
AP3M2;AP3M1											0.29648972	-0.0077572	0.20982084	-0.18642743	0.5456292	0.41290156
APEH											0.14613342	-0.3703562	0.17140001	-0.17986107	0.0994261	-0.713120143
APEX1											0.5182602	0.57581838	0.8831022	0.30008062	1.0286197	0.244924545
API5											2.0785651	1.06004651	1.24634851	1.41410955	0.7737305	0.402146657
APIP											0.00190024	-0.9392846	0.01618797	-1.48002052	0.2751926	-0.026021957
APMAP		+	+	+							4.63104247	3.48412895	2.58128192	4.17921702	3.0691377	2.891007106
APOA1BP			+								1.68231355	1.67063459	2.02852841	2.52482859	0.0368563	-0.567715327
APRT											2.60331901	0.91941198	3.04252682	0.64411481	2.9936522	0.763263067
ARCN1											1.24599766	0.92766571	1.09064353	1.60639763	0.8701014	0.915498734
ARF1	+	+	+	+	+	+	+	+		+	5.95404306	10.0092818	2.93022276	11.0979939	3.3752418	9.826437632
ARF3	+	+	+	+	+	+	+	+	+	+	1.9690251	3.47522608	3.38478653	5.03282992	4.6987253	7.099402746
ARF4	+	+	+	+	+	+	+	+	+	+	6.35602175	9.7343057	7.09771972	8.03198814	4.4387776	7.465930303
ARF5	+	+	+	+	+	+	+	+	+	+	4.43378309	7.56195323	4.00617093	7.75269763	5.6621108	7.905403773
ARF6	+	+	+	+							5.14196308	7.7795016	6.1808749	9.65566127	6.330534	9.315905253
ARHGAP1											1.06552333	1.53843625	0.82386937	0.75145149	0.8563554	1.12593015
ARHGAP12											0.01297254	-0.8811773	0.01650246	-1.29585584	0.7760691	2.015624364
ARHGAP22	+	+									1.91210153	2.31130409	0.74497979	0.62214788	0.1908579	-0.267473857
ARHGDIA											0.6931999	0.50190226	1.81166289	1.12707392	1.3562699	0.482842127
ARHGEF40											0.60540685	0.29776255	0.10488094	-0.40373611	0.0755163	-0.502674103
ARL1	+	+	+	+							6.28722772	9.40346654	4.68741405	9.15243149	6.1769787	9.910179138
ARL13B		+									3.66677918	2.76143138	0.30043424	-0.00162252	0.1519108	-0.399866104
ARL15		+		+							5.36411802	2.6179231	0.00122724	-0.94513702	2.5101909	1.944393158
ARL3	+										1.45036437	0.84214147	0.01623554	-1.37359238	0.3807329	0.117500941
ARL4A	+		+								0.23257341	-0.0828063	2.91415469	3.5570399	0.504811	0.327296575

ARL4C;ARL7	+	+									4.20083965	3.67048073	4.27771658	1.285731	0.1005209	-0.597494125
ARL4D	+		+								0.45883013	0.2837162	2.5691637	2.03561401	0.0582361	-0.937009811
ARL5A	+	+									2.48903565	2.55032857	0.76426621	1.01058578	0.8420004	0.752020518
ARL5B	+	+	+	+				+	+		4.97589325	5.94819069	3.8560906	5.81676165	3.9579197	4.690758387
ARL6IP4											0.32078544	0.0228049	0.09829296	-0.19392459	0.4296776	0.201370875
ARL6IP5			+	+							1.63226286	1.4146169	2.13084364	2.25293859	2.5067487	2.633310318
ARL6IP6											0.0651477	-0.8822136	0.33095405	0.06269519	0.2515614	-0.036778768
ARMC4	+										0.29940064	-0.0030034	0.45197179	0.11454837	0.1310485	-0.457539241
ARPC2											0.47458107	0.299764	0.01652629	-0.39731916	0.0184241	-1.04725647
ARPC3											0.59751216	0.57975133	0.4997682	0.60804176	1.6394057	0.938933055
ARPC4;ARPC4-TTLL3											1.34205965	0.57057889	3.15706014	0.66577339	1.3988178	0.351054509
ARPC5		+									1.51095709	2.02169545	0.49915313	0.67219035	0.1877213	-0.204456965
ASMTL	+										0.00078294	-2.3887335	0.7634121	0.27046267	1.7530689	1.571650823
ASNA1											2.13271264	1.08416176	1.78554031	1.7335345	1.2264704	1.148646673
ASNS											1.60727699	0.74162483	0.18826713	-0.02949715	0.0349414	-0.22718811
ASPH											0.04642873	-0.7203979	0.46450401	0.32138824	0.0910536	-0.7439092
ASPHD1											0.1387027	-0.1140219	2.298953	1.74435679	0.1789824	-0.290082296
ASS1											0.09754762	-0.5830523	0.46005628	0.28480212	0.0059907	-0.79129982
ATAD3A											0.26692305	-0.1153819	0.03961058	-0.85862033	0.0366534	-0.463739395
ATAD3B		+									1.43452403	2.16803296	1.05830222	1.38758214	0.1806995	-0.15977033
ATIC											0.16906065	-0.0710233	0.00297015	-0.30958303	0.1111855	-0.050981522
ATL3											0.76166824	0.77074051	1.20296145	0.23230044	1.3144292	0.538087209
ATP11B		+									3.24205756	2.93887011	1.27690798	1.4699192	2.277613	1.100528081
ATP13A1											0.21453336	-0.1648	0.14561746	-0.39440091	0.3932034	0.112925212
ATP13A3											1.79911768	1.26164118	0.93206214	0.73179817	0.5425219	0.576687495
ATP1A1	+										1.67323915	0.23976135	0.25395703	-0.01926804	2.1240565	0.519935608
ATP1B3											1.34322555	0.66292572	2.43520681	1.3502833	0.0521369	-0.610875448
ATP2A2	+										2.6707681	0.6972065	2.87551105	0.40893555	2.5614209	0.971970876
ATP2B4;ATP2B1;ATP2	+										1.09303628	0.3044618	0.45532588	0.12471326	0.0673809	-0.513978958
ATP2C2											0.05374398	-0.6406333	0.24765035	-0.10549418	0.9108078	1.280286789
ATP5A1											4.14074243	0.58142853	2.58683743	0.28671201	2.6572036	0.23644193
ATP5B											0.5542113	0.06811333	0.01284907	-0.19817416	0.0263093	-0.164197922
ATP5C1											2.07967158	0.6124541	3.20752092	0.83346367	3.0970101	0.479060491
ATP5F1											0.86894335	1.09585063	2.31558611	1.10739708	1.1024927	1.538358053
ATP5H		+	+								4.07863485	2.71242587	3.77710755	2.35666021	3.4030725	1.685893377
ATP5J2;PTCD1;ATP5J2-PTCD1											3.53403214	1.19657199	4.84089313	0.86955007	0.4782817	0.671292623
ATP5O		+									2.87395934	1.66275915	2.89957225	1.2657973	2.1406133	0.564613342
ATP6V0A1	+										1.08924542	0.72225444	0.2151098	-0.15419261	0.0910048	-0.403207779
ATP6V0A2	+	+	+	+							2.73150768	1.74644216	2.06123465	2.24242083	3.0081257	1.797245026
ATP6V0C											0.7084703	1.0050017	0.06313136	-0.73808225	0.0745078	-0.467309316
ATP6V0D1											0.89721947	0.83995756	0.46942556	0.26885541	1.4379271	1.257695516
ATP6V1A			+								0.19634385	-0.3220552	2.754192	2.12679482	0.267011	-0.070813497
ATP6V1B2											1.14763707	1.05355581	0.80577845	0.6827596	0.0294449	-1.306293488
ATP9A		+	+	+							3.95093696	5.09946632	3.94960303	5.00816154	6.105471	5.196394602
ATXN10		+									2.2513262	1.78607178	2.55180204	1.35743332	3.9328481	0.977364222
ATXN2L											0.37488663	0.07244364	0.57801768	0.31779416	0.138269	-0.180772781
AUP1		+	+	+							3.37559839	3.98823929	3.59753432	3.60334078	3.8138097	2.977296193
AVPI1	+					+					0.01502919	-0.9610519	0.48754139	0.32000097	0.3545861	0.080654144
B3GNT1		+	+	+							6.29194744	5.55998166	7.57223802	4.36181577	3.0411033	2.374353409
B4GALT1		+	+	+							4.05709472	4.88101641	4.67139584	8.28691864	4.007776	4.726100922

B4GALT3											1.69262756	1.20540746	0.96843868	1.01887067	0.7448461	0.481730779
B4GALT7											0.96370343	0.95501328	0.31481047	0.02246157	0.2093523	-0.212259293
BAG2											0.01406826	-0.9763947	0.14569349	-0.25170517	0.0128781	-0.852409363
BAG5		+	+	+							4.92592699	4.88014793	5.99570129	6.60200882	4.7268577	7.475664775
BAG6		+	+								1.73281157	2.30070941	1.9112339	2.26889737	3.4051314	1.192783356
BANF1		+									1.75332877	1.8960336	1.33839447	2.02143161	1.7710862	1.289609909
BASP1	+	+	+	+							3.85497055	6.00072225	4.20628765	8.91018486	3.4994718	4.75399526
BAX											0.50403594	0.52345848	0.05171062	-1.14929771	0.1195163	-0.489160538
BCAM		+	+	+							4.91899779	4.65402985	5.60641801	4.1616319	5.2683205	6.807722727
BCAP31	+			+							1.74713461	1.0002346	1.5168127	1.79382197	2.6632839	2.319754283
BCAS1	+			+			+			+	0.18850998	-0.2349854	0.4332937	0.4385802	5.1480962	7.09513855
BCAT1											0.02386807	-1.2945957	0.25783152	-0.06140264	0.0208086	-1.304463704
BCAT2											0.81887131	0.90839005	0.00340502	-1.02705193	0.9136481	1.526206334
BCKDK											0.55696753	0.32371775	0.60481696	0.62631671	0.0625605	-0.65275383
BCL2L12	+	+		+							4.27910861	2.2464002	0.70627937	0.78543027	4.4545302	2.693609238
BCLAF1	+										0.08774175	-0.7631963	0.00177886	-1.01534907	0.0441014	-1.148639043
BET1;DKFZp781C0425											0.09483659	-0.3632902	0.35525595	0.20664342	0.0861245	-0.763814926
BET1L		+	+	+							5.55174224	4.04198074	2.58129914	2.38296954	5.3566546	3.077030182
BID			+								0.1768879	-0.0705007	2.34793807	2.46452586	0.1522082	-0.452875773
BLMH											0.10955696	-0.7897867	0.11917923	-0.56040827	1.2276461	0.874945958
BLVRA											0.80658766	0.8856767	0.09009619	-1.31728554	0.3051328	0.002475103
BLVRB											0.29475833	-0.0067094	1.31084705	1.28558095	1.4892923	0.775024414
BOLA2											0.18857004	-0.2527625	0.53999626	0.60202217	0.0776645	-0.250925064
BPNT1			+								0.25430007	-0.0471319	3.11849702	1.77920405	0.5371528	0.431243896
BRD3;BRD4											0.06157324	-0.9312337	0.68404089	0.68612734	0.1493438	-0.364627202
BRI3											0.75005325	0.51220322	0.05309338	-0.31672796	0.7042768	0.464522044
BSG	+										0.00960729	-0.9198074	0.56840083	0.14056269	0.1417021	-0.384269079
BST2			+								0.15474924	-0.30992	5.33606709	6.71978124	0.0292188	-0.805404663
BUB3											0.70348511	0.29006322	0.00064433	-0.68662516	0.6087424	0.671128591
BYSL											0.46737025	0.26172129	0.31239797	0.01770973	0.1078041	-0.624924342
BZW1											2.29868928	0.84101677	1.75086718	0.51156044	0.8835646	0.313061396
BZW2			+								0.38742174	0.10611852	1.59151614	2.22636668	0.3825081	0.143873215
C11orf52	+			+							0.55893803	0.27369817	0.12323353	-0.37229665	4.2699456	4.772192637
C12orf10	+										0.12217211	-0.6333218	0.41314418	0.15492376	0.020936	-1.102549235
C12orf23											0.0033497	-1.1305682	0.00109367	-0.82837359	0.000321	-0.88531367
C14orf166											1.03902384	1.16549619	1.03711999	0.14187876	0.6933812	1.092611949
C16orf55	+			+							0.7825925	0.51705488	0.50253671	0.16018422	3.8394213	3.280143102
C1orf172				+							0.47877688	0.24528186	0.16379527	-0.35161273	3.3069618	4.505901337
C1orf58;BROX											1.03375956	0.65248235	0.23113103	-0.19301669	0.1968903	-0.271550496
C1QBP			+								1.3476516	0.46716436	5.09440377	3.18016307	0.7776044	0.990910848
C21orf33											0.56986046	0.3886795	0.20044466	-0.12789218	1.3099926	1.821142832
C22orf28											0.00387947	-0.5728709	0.02985592	-0.42249044	0.0451865	-0.187311808
C5orf28											0.05530961	-0.6049538	0.31504047	0.02773158	0.0425548	-0.962607702
C6orf211											0.77549714	1.00293668	2.02677842	1.91694005	1.1239572	0.504858017
C8orf47	+	+			+			+			4.45885238	3.29605802	0.13697251	-0.25934982	0.1406522	-0.63879331
C9orf114											0.28889688	-0.0163542	0.42743847	0.30115573	0.5348049	0.958161672
C9orf123	+	+									3.7104209	5.77831395	0.97187811	2.66463025	0.0326799	-1.243821462
C9orf169			+	+							0.29388669	-0.0073903	3.54891244	4.37708982	4.6330795	6.149560293
C9orf91											0.00390347	-0.6232179	0.13384597	-0.3105011	0.0991916	-0.529754639
CA2											0.88962979	1.17751503	0.01982912	-0.86287181	1.405338	0.73998642

CA5B											0.97679763	1.38916397	0.3409505	0.07172267	0.2094968	-0.33993276
CACNA2D1			+								0.09476348	-0.4459171	5.95179122	3.6907107	0.192173	-0.370224635
CACYBP											0.8868296	0.64187876	1.20633852	1.36164411	0.7841634	1.192828496
CAD											0.1520213	-0.2838726	0.00424157	-1.45826022	0.2004667	-0.460849126
CADM4	+										0.93985618	1.0519015	0.02579609	-1.29167875	0.0765427	-0.768300374
CALD1											0.13398779	-0.1999995	0.25893004	-0.03735352	0.2099303	-0.232570012
CALHM2		+		+							3.28003105	2.74288877	0.28193189	-0.03242493	3.732616	3.304164886
CALR											0.06818118	-0.7823607	0.02359142	-0.3267676	0.0218633	-0.889717102
CAND1											1.90667209	0.34610558	1.33475278	0.11153603	1.4567769	0.296005249
CANX		+	+	+							5.77279757	4.23757044	6.8085234	3.97018687	6.6210205	3.306780497
CAP1											0.02419175	-0.2912458	0.0458801	-0.2815272	0.0148613	-0.498563766
CAPN1											0.42933234	0.09251658	0.05746136	-0.20910136	0.170302	-0.056634267
CAPN2											0.35587053	0.09758822	0.71840935	0.32664426	0.5132374	0.098925908
CAPNS1	+										3.32466979	1.07613881	2.75053837	0.69904709	0.0911316	-0.128395716
CAPRIN1											0.12837838	-0.3621438	0.65801608	0.46955808	0.0420487	-0.461870829
CAPZA1											0.56194933	0.59197299	1.1726892	1.33144252	0.5477866	0.679561615
CAPZB											0.09059917	-0.0927315	0.06240437	-0.12934621	0.4462722	0.085343679
CARS											0.31706512	0.00926908	0.99822983	0.22149086	0.2675304	-0.015576045
CAT											0.47308326	0.07652346	1.00368846	0.3809433	0.0017355	-0.981461207
CAV1		+	+	+							3.68805191	3.55153465	3.46181023	5.39175224	4.2496597	7.12888209
CAV2	+		+	+							0.28846156	-0.0363121	3.55052125	3.55698077	2.8449931	3.339197795
CBR1											3.27635466	0.55460676	1.55736322	0.56898435	0.845329	0.665637334
CBS											0.66883576	0.73769633	0.11853135	-0.71791522	0.053812	-0.954768499
CBX3	+										1.68955451	1.27503713	0.86992242	0.38536517	2.8160942	0.939325968
CCDC47											0.78514636	0.75152397	0.48993121	0.44730186	0.0054428	-1.520040512
CCNB1											0.19342738	-0.3200569	0.30322775	0.0045681	0.2720677	-0.040889104
CCND3											0.11285772	-0.5027847	0.08559518	-0.88945262	0.0853565	-0.560624441
CCNY	+	+	+	+				+			4.22747233	5.60869026	3.52734745	4.86237208	2.812488	4.160552979
CCNYL1	+	+	+					+			5.20827028	3.83037567	5.14592276	2.95775795	2.9963244	1.649326324
CCRN4L		+	+	+							4.1783031	2.76542409	5.41237133	5.35171064	4.253768	2.60360082
CCT2											0.40372181	0.02778625	0.16081279	-0.04375966	2.6829067	0.2598025
CCT3											1.76739224	0.31312943	1.07119035	0.1005923	2.9187934	0.315788905
CCT4											1.10859133	0.31796201	2.09469605	0.14848836	2.2396457	0.37141037
CCT5											0.16689012	-0.0639572	0.07933789	-0.09748395	0.2544082	-0.013931274
CCT6A											0.19794884	-0.0349426	0.0673837	-0.07673963	2.5057823	0.30674998
CCT6B											1.42272755	0.84310786	0.13158166	-0.45623461	0.6567085	0.751440048
CCT7											0.47286711	0.04916382	0.28220515	-0.00415611	0.7675209	0.076481501
CCT8											1.25487833	0.25846799	0.12785553	-0.04077021	0.9095361	0.068286896
CD151	+	+	+	+							4.58179927	6.49497986	3.93413216	7.2220726	4.9650674	7.307088852
CD276		+	+	+							5.60157433	7.24960772	5.56583449	6.08704503	4.881228	5.900683085
CD44		+	+	+							3.66913145	4.20615451	5.65146018	6.89719899	5.0918635	4.592794418
CD46			+								1.97709175	1.63382657	1.69260042	3.32129478	0.1823609	-0.304182053
CD47			+	+							1.6912869	1.78018188	4.26486569	2.9079984	4.5580645	3.578428268
CD55			+								0.01925893	-1.1388747	5.68324755	5.83608119	0.035997	-0.587333043
CD58											0.0850298	-0.7813206	0.03737482	-0.91111819	0.0513099	-0.701100032
CD59	+		+								0.12823071	-0.5111898	5.5425757	5.24240812	1.2562226	0.630559921
CD63		+	+	+							4.81626488	7.60439301	4.58646654	7.91180929	4.879751	8.799007416
CD81	+	+	+	+							3.60391393	10.2234484	5.45769282	7.67405891	5.5988958	8.514958064
CD82	+		+	+							1.01882467	1.63766289	4.62200304	4.85793495	5.7578651	3.448679606
CD9		+	+	+							4.50437352	7.00129445	5.03166116	8.08529154	6.2807598	9.515175502

CDC37											1.38844059	1.55617332	0.43948838	0.34326108	0.2865558	-0.056776683
CDC42			+	+							2.241913	1.6714344	2.61338598	1.92045657	3.249177	1.920135498
CDCA3	+	+	+	+							4.21913087	6.03343964	4.19216541	7.57803472	4.3767811	3.832825979
CDCP1			+								0.30558746	0.00293668	5.15326219	5.56780306	0.0297798	-1.368038177
CDH13											0.85268618	0.43357976	0.42359884	0.227911	0.7128181	0.540867488
CDIPT											0.2486026	-0.1097902	0.50888672	0.29961268	1.0292692	0.760305405
CDK1	+	+									3.99384763	1.83131345	3.66858742	1.50371869	2.4481006	1.246000926
CDK2;CDK3											1.01561061	0.65955734	0.97866081	0.8785375	1.2460995	0.678037643
CDK4											2.14799542	0.84367752	0.11272002	-0.64207331	0.0266786	-0.702757517
CDK6											0.03004476	-0.3070037	0.94581757	0.65966543	0.0357124	-1.626260757
CDKN2A;CDKN2B			+								0.69047835	0.84244029	4.28865226	2.54248873	0.0329962	-0.884096781
CECR5		+									3.82628292	2.36007563	1.09705905	1.20306651	1.6553456	1.279061635
CELF1											0.81186608	0.59442774	0.03306194	-1.80257098	0.2480895	-0.047611872
CELSR2				+							0.10117212	-0.340655	0.10029609	-0.39259974	2.8212442	3.759860357
CEPT1											0.27913033	-0.0296625	0.09542753	-0.72144699	0.2886126	-0.020345688
CERS2		+	+	+							3.72555775	2.39452362	4.27058721	4.9432284	4.6053456	2.402774811
CFL1											1.84623627	0.88769595	1.48391416	0.54428101	2.8653641	0.597730001
CHCHD3	+	+	+	+				+	+	+	5.79678974	8.0970389	4.44556182	9.42184703	7.3147538	7.453032811
CHCHD6	+	+	+	+	+			+			3.98885121	6.66784986	4.08474621	4.59390259	3.6791913	5.011208216
CHIC2											0.08234133	-0.6484718	1.24494256	1.16377258	0.0854101	-0.901118596
CHMP6	+	+	+	+		+	+	+	+		5.94526922	7.02952449	4.05103085	6.78173256	5.8202302	6.006587346
CHORDC1											0.8168061	0.67067655	2.31244114	0.59183184	0.1680295	-0.294761658
CHP1	+	+	+	+							3.81586042	6.28078524	5.52243937	8.64180628	4.7311033	6.834328969
CIB1	+	+	+	+							3.1664711	2.53191249	3.42321397	4.32869466	5.9186055	4.667348226
CISD1											0.10319763	-0.4196402	0.13240978	-0.47367795	0.1260826	-0.364789327
CKAP4		+	+	+							4.49960454	5.01556714	6.99983901	4.18709501	4.2158528	4.553815842
CKAP5	+										1.82985696	0.35777982	1.05044019	1.66394615	0.5817858	0.818995158
CKB											0.88316256	0.14413706	1.43919898	0.14117877	0.0348513	-0.837610245
CKLF											0.11080457	-0.8518442	0.38251342	0.21187528	0.1907016	-0.177440643
CKMT1B;CKMT1A											0.01927158	-0.3319263	0.3461422	0.10602824	0.0235778	-0.248516083
CLASP1;CLASP2	+										0.35462013	0.06147703	0.02547487	-0.47397931	0.0064043	-1.518911362
CLCC1											0.03384314	-0.5918477	1.00495809	0.64662298	1.5968232	0.984207153
CLCN6											0.62613745	0.4318854	0.14111594	-0.22708257	0.1781976	-0.241442362
CLDN1		+	+	+							5.25277789	2.15332095	4.29579823	5.55862427	2.9522218	2.762903849
CLDN12	+										0.1327188	-0.5920849	0.20902263	-0.15833092	0.5196919	0.615479151
CLDN3				+							0.04482195	-0.8355954	1.23483856	0.98304113	5.2710705	7.684064865
CLDN7				+							0.02968424	-0.7964452	0.51912258	0.34790738	4.3020811	6.881729762
CLDN9											0.39704953	0.10039711	0.2396579	-0.17817243	0.6634619	0.895901362
CLDND1	+	+	+	+							3.08753137	4.38695208	3.88951448	4.57239469	5.7832442	3.998105367
CLEC11A		+									1.31291577	2.5130043	0.05728285	-2.65917079	0.9817092	2.04352061
CLGN											0.83662412	0.80322393	1.66049804	1.77925173	0.2603459	-0.080378215
CLIC1											0.4221093	0.07855988	2.52232426	0.30270131	0.7780989	0.091655731
CLIC3											0.75177608	0.6182696	0.57096833	0.3306764	0.8014111	1.146028519
CLIC4		+									4.7944096	2.87295151	0.68241857	0.1804498	1.6784933	1.371072769
CLN3	+	+	+	+	+			+	+		4.01329032	4.37639618	4.81647541	5.14382617	3.3377044	4.249161402
CLNS1A											0.69271961	0.33558909	0.70996884	0.2294	0.4109458	0.16825676
CLPTM1											1.39405262	0.48051325	1.15112409	0.39346504	0.0467774	-0.953478495
CLPTM1L			+								1.83558093	1.80538495	2.07053966	1.99348259	3.4390168	1.00261879
CLTA			+								1.40748353	1.86142413	3.12429357	2.2555453	0.008471	-0.905010223
CLTC											0.00457023	-0.3157088	0.00030105	-0.50272814	0.0185986	-0.177436829

CLUH;PCDHAC2											0.23625392	-0.1305218	0.20188512	-0.10257594	0.1404128	-0.67360878
CMBL											1.2048933	1.06746991	1.69151539	0.56508764	0.2101362	-0.116208394
CMPK1											0.12834733	-0.1475404	2.38313519	1.23831495	0.2522641	-0.089313507
CMTM3		+									2.75673261	2.39919535	0.16707921	-0.41963196	0.036082	-1.284596761
CMTM6											0.4235321	0.22345225	1.48871029	0.9475867	1.1196635	1.168662389
CMTM7		+									4.04021297	2.35165215	0.2413017	-0.16235034	0.0551367	-0.305290858
CNDP2											0.04722226	-0.5969168	0.6933601	0.33345922	0.0075293	-1.184268316
CNIH											0.42066213	0.13095919	1.79819766	1.93006961	0.1690138	-0.345648448
CNIH4											0.00993775	-0.332159	0.01611748	-0.60883967	0.3173606	0.044716517
CNN2			+								0.53759541	0.46621831	3.57777146	1.89787928	0.0415064	-0.762072245
CNN3											1.39582741	0.40314356	0.50639333	0.42347463	0.38962	0.061601003
CNP											0.17534052	-0.3230871	0.30287664	0.00300598	0.024503	-1.036441803
COASY											0.06976634	-0.8494708	0.02752867	-1.08962186	0.2113555	-0.138308207
COMT	+	+									2.72558126	2.69966888	2.94147883	1.10702197	1.2499002	0.544422785
COPA											0.00649719	-0.3366216	0.00629164	-0.23839188	0.0424336	-0.093261719
COPB1											0.23558497	-0.0176029	0.13126487	-0.113451	0.189219	-0.032739003
COPB2											0.08915728	-0.1645508	0.03404065	-0.24397214	0.0435424	-0.252612432
COPE											1.14612962	0.8494161	1.06096577	0.154356	0.8202554	0.198079427
COPG1											1.56710352	0.29465485	0.63846784	0.05267906	0.9099296	0.102033615
COPS2											0.3503822	0.10148748	1.16293693	0.57671102	0.2255053	-0.275982539
COPS3											0.9944406	0.72716268	0.72395532	0.36372185	0.1068558	-0.658460617
COPS4											0.06207337	-0.6559626	0.48274893	0.30951182	0.2607818	-0.067452113
COPS8											0.07483886	-0.2184588	0.42273765	0.18831317	3.52E-05	-1.442747752
COPZ1	+										0.3360758	0.08620453	0.22740161	-0.10839272	0.7860142	0.438138326
CORO1B											0.15020218	-0.4415512	0.18669565	-0.31798299	0.341804	0.125920614
CORO1C											0.06093182	-0.4752935	0.30170855	0.00160853	0.4719845	0.244723002
COTL1											0.49797249	0.23199145	0.6541718	0.56858444	0.0355747	-0.748286565
COX15											1.90572476	1.82119497	1.53618746	1.73839315	0.9306933	1.280286789
COX20											0.01850974	-0.8876171	0.10266544	-0.38037809	0.0690678	-0.381317774
COX4I1											0.59629258	0.24110985	0.01750741	-0.74108442	0.2301097	-0.136350632
CPD		+	+	+							4.90548541	5.93365733	3.18708509	5.03083038	2.7956521	3.127709071
CPM			+								0.31185834	0.012345	4.32499092	3.32647959	0.0467988	-0.624624252
CPNE1											0.04515335	-0.1961066	0.10156685	-0.18659782	0.225613	-0.377563477
CPNE3											0.1572122	-0.1061643	0.02099878	-0.32314364	0.3611971	0.026726405
CPOX											1.7439464	1.0178051	0.67661097	0.23469035	0.5481358	0.232554118
CPS1											0.0620368	-0.9924914	2.82E-05	-0.48699188	0.1691243	-0.428326289
CPSF6											0.05285122	-1.2273553	0.00493828	-2.54683749	0.0384857	-1.893102646
CPSF7	+										0.85730845	0.43416913	0.62273373	0.3565534	0.012011	-0.675865173
CPT1A		+									2.93092503	2.06757545	3.92495861	1.47366524	2.3275534	0.914255142
CRABP2											0.07907406	-0.5044759	2.30523516	1.42488925	0.0919028	-0.320184708
CRBN											2.3942455	1.15538406	0.85864452	0.78221893	0.3187395	0.038175583
CRIP2											1.64742698	0.42250443	0.40822333	0.17710495	0.4697754	0.481534322
CRLF1											0.1883382	-0.4464442	1.77278479	0.7157383	0.1494221	-0.349704742
CROCC											0.13604819	-0.349205	0.07118916	-0.43888982	0.4130246	0.15863355
CS											0.98958873	0.35858154	0.00892056	-0.21951612	0.2856909	-0.003405889
CSDE1											0.48364961	0.36362012	0.01784152	-1.24926186	0.0023183	-3.093427658
CSE1L											3.2990787	0.42665863	0.78996608	0.10061518	3.6808963	0.461090088
CSNK1G3;CSNK1G2;CSNK1G1				+							1.28608495	2.12697665	1.15267588	2.20098241	4.145812	2.90031751
CSRP1											0.154168	-0.2715912	0.02809656	-0.49548022	0.3046416	0.016916275
CSTB			+								0.94557238	0.65579732	1.44441073	2.47417831	0.0509472	-0.35583051

CTBP1	+										0.04095011	-0.2902597	0.55579399	0.32109133	1.163078	0.893660863
CTBP2											0.51394012	0.5009861	0.25676455	-0.11201223	0.3247651	0.043113708
CTDSP1											2.07239127	1.43195089	0.0282747	-0.96969287	0.2659363	-0.078482946
CTDSP2	+										0.46101783	0.19811122	0.51057429	0.34741656	0.1382943	-0.423281352
CTNNA1											0.95309469	0.45814768	1.86407579	0.34376335	0.187573	-0.087674459
CTNNA2											0.1704315	-0.2688001	0.2202945	-0.1564153	0.2360843	-0.162595113
CTNNB1											0.01674325	-1.0972716	0.2654987	-0.09894689	0.1419634	-0.297571818
CTNND1											1.18878622	1.5805734	1.53211821	0.2614975	1.5645926	0.705663045
CTPS1											2.71740133	0.87161128	2.19684628	0.60849253	1.2094353	0.887631098
CTSD											0.2940269	-0.0159149	0.17391291	-0.1461436	0.5180342	0.085308711
CTTN											0.03640021	-1.2837156	0.16587248	-0.10946655	0.0106379	-1.172997793
CUL1											0.15012864	-0.3516432	0.26582286	-0.05783081	0.0441569	-0.886403402
CUL2											1.10280318	0.37034925	0.00028076	-1.01858711	1.5110909	0.442237854
CUL4B											2.869412	0.5571448	1.32548045	0.34625944	0.7430114	0.43651708
CUTA											1.0453455	1.2253863	3.19408922	1.13033867	1.2331565	0.920174281
CXADR		+	+								3.39603694	4.62053998	4.53828676	4.91746839	0.0247862	-1.037650426
CXCR7											0.19017081	-0.2156048	0.006242	-1.0745519	0.4033032	0.257515589
CYB561				+							0.92532782	0.65561867	1.96721148	1.39406459	3.8743744	3.902064641
CYB5B		+	+	+							4.23551978	5.78228188	5.8343391	6.10887527	2.614084	5.30642128
CYB5D2											2.60366773	1.15372467	0.03092499	-0.90294266	0.2099324	-0.239960353
CYB5R3	+	+	+	+							7.33923801	8.5927906	8.22225046	9.07913272	5.5268773	8.579743703
CYBA	+										0.10805668	-0.6456095	0.36993182	0.06050428	0.2299331	-0.182408015
CYC1											0.0373473	-0.8397681	1.31341878	0.35442543	0.6915925	0.284346263
CYCS	+										0.78321365	0.24651909	1.26500589	0.28690656	0.0139356	-0.405326207
CYFIP1;CYFIP2											0.41030619	0.15789922	0.24951754	-0.03026009	0.411285	0.138050079
CYP51A1											0.73786625	0.51528676	1.00237147	0.79371071	0.3012057	0.000385284
CYS1	+										0.16353335	-0.2512004	0.76570153	0.59568342	0.351063	0.085473378
CYSTM1											0.10576743	-0.4263363	0.15621637	-0.25718498	0.2917566	-0.021250407
DAAM1											0.15962922	-0.3138593	0.05842423	-0.84786479	0.721891	0.673617681
DAB2											0.90168229	0.67781703	0.02169157	-0.64171727	0.0334767	-0.970160166
DAD1		+									1.49064646	2.04776446	0.34392263	0.07319895	0.5159503	0.309850693
DAG1		+	+	+							3.59453462	2.30135409	4.77981638	3.04027112	2.2366342	2.19382604
DAGLA											1.61825306	0.91860771	0.10724211	-0.4281311	0.0109953	-0.935201009
DAGLB		+	+	+							4.26258489	6.84906387	4.83127647	6.8218956	5.4729212	6.822306315
DAK											1.19143161	0.46743011	0.43472969	0.25016403	1.2940374	1.581992467
DAP3											0.09505074	-0.5565472	1.86596227	1.47265879	0.0341502	-1.034950892
DARS;DKFZp781B11202											0.02066025	-0.4436684	0.02531206	-0.26922417	0.035459	-0.236953735
DARS2											0.54492097	0.41783714	0.00111595	-0.86274719	0.3840767	0.131634394
DBNL											0.63835781	0.37564341	1.07283603	0.56085078	0.1579323	-0.273307165
DCAF11	+	+	+	+							3.60381433	4.11435191	4.93533373	5.47918447	3.9923015	4.269292196
DCTN1											0.0056403	-1.4154625	0.19838241	-0.18614578	0.4034594	0.056284587
DCTPP1											0.67842613	0.38394038	2.86388761	1.00825818	0.9487018	0.574563344
DCUN1D3	+	+	+	+				+	+	+	4.64401926	3.18788656	3.92299407	3.92090797	3.8339484	3.254378637
DCXR											1.61545435	1.04055023	0.19520357	-0.21111679	0.6705106	0.43769455
DDB1											0.05695078	-0.248757	4.09E-05	-0.69243495	0.0094071	-0.438695908
DDOST	+										1.43527737	1.22072792	0.0122318	-0.41484515	0.0749434	-0.207343419
DDT;DDTL			+								0.54466734	0.50467745	1.71288961	2.25002734	2.0725781	0.541796366
DDX1											0.01149303	-0.3920905	0.0037922	-0.55759112	0.0308238	-0.453013738
DDX17											0.05311005	-0.2076467	0.00345744	-0.66471418	0.0323903	-0.351477305
DDX19A;DDX19B											0.47007733	0.37034162	0.38301871	0.08499273	0.5706789	0.383972804

DDX39B;DDX39A	+										2.00153639	0.71346664	1.25302182	0.77001699	1.2595653	0.411661784
DDX3X;DDX3Y	+										0.0498984	-0.1703142	5.08E-05	-0.78314145	0.0189627	-0.430010478
DDX46	+										0.00584483	-0.5933355	0.00885054	-0.3567454	0.0001601	-0.952947617
DDX5											4.28E-05	-0.7554499	0.00672171	-0.62817701	0.3688712	0.185720444
DDX6											0.65054322	0.76982562	0.90231109	0.75493495	1.317103	0.291421254
DECR1			+								1.21320031	1.94683901	2.19244161	2.51371956	1.0853557	1.653394699
DEGS1	+	+	+	+							4.87122316	7.36611112	5.10769578	8.32110532	3.2945378	5.309862773
DEGS2	+			+							0.55051163	0.34666443	0.19292213	-0.23357264	3.6525571	3.407246908
DEK											0.68318507	0.62092018	0.39152336	0.17897733	0.0950288	-0.624544779
DERL1				+							0.07567583	-0.4285444	0.08620961	-0.4327666	4.0685719	3.446873983
DESI2	+										1.0191327	1.5755628	0.04583284	-0.82194138	0.0424768	-0.778270086
DHCR24	+										0.04394536	-0.8874842	0.22116618	-0.14171982	0.6575077	0.707271576
DHCR7		+	+								3.13802531	3.00027911	3.04648383	2.65757879	2.5725763	1.49349912
DHRS7											0.51888026	0.21691004	4.56359693	0.70711962	0.3149956	0.030141195
DHRS7B											0.68123313	0.43677839	0.16381751	-0.39011256	0.16489	-0.461075465
DHTKD1											0.29446524	-0.008639	0.30823329	0.01210976	0.1717928	-0.318141937
DHX15											0.00383892	-0.6696618	0.79252157	0.17484601	0.0007067	-0.570421219
DHX9	+										0.01541243	-0.3770065	0.05102381	-0.1325709	0.5291788	0.05255572
DIAPH1											0.0183155	-0.6406364	0.04931903	-0.33545431	0.0718973	-0.68178304
DIRC2	+	+	+	+							3.82853829	5.14263344	3.54950566	4.66897519	3.3710182	4.386709849
DIS3											0.91596636	0.59180323	0.89373852	0.67952156	0.0628555	-0.802796682
DKFZp686F20250;FAM114A1											0.14001231	-0.3762703	0.30152996	0.00068919	0.0218879	-0.886959712
DLAT		+	+	+							1.85539063	3.17266019	4.48547575	3.64627775	3.2950164	2.967154185
DLD											0.01999951	-0.5841637	0.66525667	0.10788282	0.0016498	-0.539093653
DNAJA1											0.02175985	-0.6236248	1.89955172	0.41447767	0.6469791	0.516917547
DNAJB11											0.16863223	-0.42847	0.36982523	0.14074453	0.3200578	0.054052353
DNAJC5		+	+	+							4.0658691	7.00448736	6.08043165	7.76905505	4.1482757	7.110827128
DNAJC7											0.50400236	0.40571276	0.08677233	-0.71018855	0.0041601	-1.221027374
DNAJC9	+										0.04897717	-0.1917381	0.04749105	-0.52821668	0.3523975	0.046182632
DNER											0.20792449	-0.1177432	0.88082333	0.61062431	0.0251729	-0.525681178
DNM1L											2.05220498	1.06920052	0.33129216	0.02296066	0.0023784	-0.676457087
DNM2	+	+									3.62700103	2.06391207	3.1304349	1.01807531	2.6856545	0.484430949
DNPEP											0.21546425	-0.1959979	0.08523244	-0.36022822	0.1171181	-0.131726583
DNPH1											0.44589444	0.21994209	0.06229624	-0.75270398	0.300839	-0.000631968
DOLK											0.45906053	0.28096517	0.42721818	0.14925639	0.0199669	-1.146958669
DPM1											0.12131828	-0.4403203	0.36877997	0.14469147	0.0642594	-0.62019666
DPP3	+		+								0.00249472	-0.5426044	3.50318665	2.63893191	0.2756467	-0.041674932
DPY19L1											0.28673165	-0.0417067	0.24193853	-0.1652991	0.3394809	0.070170085
DPYSL3											0.00258567	-0.7700926	0.0062467	-0.56704458	0.0986383	-0.572755814
DSC1											0.26800632	-0.0667343	0.00211232	-0.84857051	0.1384892	-0.280949275
DSC2											1.79444748	1.7654349	0.13135269	-0.34627088	2.2454434	1.571960449
DSG2		+	+	+							3.62960023	3.70492744	5.82472211	4.42943573	3.7878326	4.023339589
DSTN											0.1471474	-0.5516307	0.09881181	-1.38289706	1.7407861	0.497028987
DTYMK			+								1.73584233	1.63460223	3.05420276	2.41323789	0.2472692	-0.133305232
DUSP22	+	+	+	+							2.7290014	1.88420677	2.91140647	2.46934954	3.6665536	2.92414856
DUSP23	+		+						+		0.05433985	-0.3591995	3.26304362	2.58203634	0.7287306	0.542890549
DUT											1.464733	1.72729747	2.05397126	0.74283854	1.1615117	1.611291885
DYM	+	+	+	+				+	+	+	5.20325708	4.58321953	3.85776966	5.18287214	4.168761	4.62507693
DYNC1H1											0.06315993	-0.5969512	0.00628452	-0.9566892	0.034383	-0.496664683
DYNC1I2											0.99172744	0.67070834	1.72987986	2.02308464	0.6992512	0.521675746

DYNC1LI1											0.10053947	-0.385355	0.4823195	0.32453346	0.0444973	-0.275753021
DYNLL2;DYNLL1											0.36152191	0.0719223	0.04334699	-0.7218647	0.7665757	0.926176071
EARS2											0.3449158	0.06099892	0.8228765	0.7759196	0.3825217	0.080337524
EBAG9;PDAF		+		+							3.11675414	3.83617147	1.2904277	2.02607218	5.8081439	3.574359258
EBF2											1.36492563	1.03361193	0.22538671	-0.17420324	0.1599558	-0.350111008
EBP											0.94346043	0.76635615	0.56249441	0.26418877	0.9564646	1.486168543
ECE1			+	+							0.03482327	-1.2664801	3.71414028	6.1791509	3.8713357	2.525982539
ECH1											0.05169573	-0.6355921	0.40621033	0.26418813	0.1107864	-0.665414174
ECHS1											1.00038328	0.45997874	3.29531649	0.77254613	2.0365962	0.468419393
ECI1;DCI											1.94785398	1.10070992	0.49954866	0.6627636	1.3784355	1.633416494
ECI2	+	+									2.2412826	2.72890218	1.52181744	2.13559341	0.1659444	-0.591279348
ECM29;KIAA0368											0.6353672	0.12742996	1.23141142	0.67707062	1.6285379	1.885674795
EDC4											0.08657202	-0.6480001	0.27338846	-0.05894089	0.1563752	-0.452161153
EEF1A1P5;EEF1A1	+										2.36875928	0.28769302	2.96800066	0.22678757	0.4708972	0.052353541
EEF1A2	+										0.62014733	0.54754766	0.00783507	-0.53037771	0.4898495	0.181163788
EEF1B2	+										2.21210616	0.37404315	1.61251121	0.32104683	0.7283444	0.097576777
EEF1D											1.71662086	0.2015082	0.93549383	0.11147817	0.1706587	-0.061527252
EEF1E1;hCG_2043275											0.10913658	-0.5768878	1.04158513	0.5671285	0.4288723	0.129259745
EEF1G											0.31089396	0.00302951	0.09661191	-0.08453878	0.1295392	-0.079174042
EEF2											0.21375844	-0.0282415	0.0001921	-0.31329282	0.2247999	-0.014842351
EEPD1	+	+			+			+			4.46025859	4.03264046	0.33417856	0.09530195	0.7260801	0.372432709
EFCAB14		+									2.14953157	1.93841298	0.51551831	0.22445552	0.2902073	-0.020136515
EFHD2											0.0912563	-0.2569834	0.0152084	-0.50114568	0.0651709	-1.146800359
EFNA5			+								0.14938801	-0.2173932	2.51605191	2.29914602	0.0563963	-0.896668752
EFNB3	+										0.30356358	0.00363986	0.70036264	0.55416171	0.0342409	-0.848694483
EFR3A		+	+	+							4.29953428	5.54570071	5.39836513	5.13780467	4.0653752	4.783201853
EFTUD2											0.01481273	-0.5174389	0.01033538	-0.612957	0.1625852	-0.129833221
EGFR											0.0659964	-0.6408806	0.02898096	-0.84103584	0.018367	-1.197313309
EIF1AY;EIF1AX											0.72914154	0.24356842	0.11167013	-0.47079722	0.2464571	-0.115442276
EIF2B1											0.93125954	1.01835187	1.00022894	0.52755292	2.1748213	0.179385503
EIF2B2											0.32692327	0.04970996	0.41114662	0.18232536	0.0931326	-1.172308604
EIF2B4											0.05296935	-0.5190976	0.13589679	-0.27847799	0.0674674	-0.502717336
EIF2S1											1.22274641	0.41689364	0.58270571	0.12703514	1.2571773	0.242832184
EIF2S2											0.55522336	0.42767207	0.38665927	0.15878105	0.0345707	-0.704488754
EIF2S3;EIF2S3L											0.0140052	-0.688268	0.12180669	-0.19088173	0.1992988	-0.054486593
EIF3A											0.5612519	0.10315641	0.05743276	-0.2132562	0.1833995	-0.092374166
EIF3B											0.00081258	-0.5281067	0.01367973	-0.5606823	0.0004756	-0.385317485
EIF3C;EIF3CL											0.285261	-0.0063445	0.19027193	-0.03999901	0.9607028	0.229520162
EIF3D											0.18305513	-0.1040262	0.00108474	-0.3928744	0.0919891	-0.095608393
EIF3E											2.74591164	0.4691995	0.17461832	-0.04160754	0.700952	0.112440109
EIF3F											3.21429968	0.87235006	3.00627858	0.87987836	1.5912159	1.612701416
EIF3H;EIF3S3											2.67604736	0.96903674	0.71880786	0.33975474	0.2685423	-0.062076569
EIF3I											0.16187865	-0.3873056	0.33407487	0.07005374	0.1768897	-0.386501948
EIF3J											0.01184467	-0.9133561	0.8029913	1.09279442	0.1369484	-0.40242068
EIF3K											0.6543073	0.6944116	0.78280361	0.36528015	0.0238829	-0.768689473
EIF3L											0.22762893	-0.0578391	0.15437812	-0.12544632	0.8115597	1.221512477
EIF3M											1.00011066	1.28257942	2.71508629	0.52324359	0.4094768	0.394803365
EIF4A1	+										2.372386	0.23662567	1.01336738	0.15723928	2.7464599	0.24431928
EIF4A3											0.70838728	0.77452278	0.66432241	0.53969574	0.013746	-1.759051005
EIF4G1											2.51633817	0.3684686	1.30413549	0.18836021	2.4845343	0.431952159

EIF4G2											0.84829017	0.19699415	1.54357055	0.22047997	0.7638149	0.748166402
EIF4H											0.03112216	-0.6358159	0.0755892	-0.61178652	0.3786674	0.092586517
EIF5											1.91480208	0.76680501	1.61233979	0.46061071	1.7234565	0.44707044
EIF5A;EIF5AL1											1.34033395	0.38308652	2.42725226	0.65674019	2.2948913	0.574405034
EIF6											0.0118747	-1.695495	1.3793757	1.98825963	0.7996406	0.520844142
ELOVL1											0.06526572	-0.4080365	1.09691389	1.89094861	0.5043273	0.667263031
ELOVL5											0.19721614	-0.2737223	0.9321107	1.51055209	0.0509201	-0.759203593
ELOVL6											1.05236368	0.77334849	1.11152189	0.79583104	0.257447	-0.033725739
Em:AP000351.3;GSTT	+										1.81022801	0.71903229	0.3098112	0.01400566	0.3865575	0.216133753
ENDOD1	+										0.06706369	-0.7463932	0.00097419	-1.2912178	0.1432339	-0.360841751
ENO1											0.73031187	0.20708593	1.27395929	0.18449529	1.7696716	0.137098948
EPHB4											0.55229115	0.59403547	3.0534452	0.82725334	2.0433901	1.808689117
EPHX1											1.06558511	0.97512118	2.60249495	0.83559863	1.3129399	1.335350037
EPPK1											0.01984586	-0.8008944	0.12482473	-0.34197172	0.0151052	-0.885667801
EPRS											0.04172444	-0.3509897	0.00037612	-0.58310572	0.0050264	-0.668391546
ERGIC1	+										0.50610996	0.44904709	3.02760205	0.48318164	1.330373	1.581051509
ERGIC2		+	+	+							3.7677171	5.827027	4.27081984	6.56314214	4.5984587	5.517553965
ERGIC3		+	+								4.05938507	2.32732073	3.65312815	3.0852712	0.286396	-0.039210002
ERI3;PRNPIP											0.41544285	0.06125196	0.01567801	-0.85453733	0.0654138	-0.938330968
ERLIN2											0.45671967	0.17673556	0.39280127	0.18628883	0.0720633	-0.607223511
ERMP1		+		+							2.15364436	2.60555204	1.13094435	1.14684741	2.4289217	1.910587947
ERP29		+	+								2.93439342	2.13887151	1.3857927	2.45809174	1.7151865	0.497577667
ESD											0.26356462	-0.0205701	0.00588764	-0.75825564	0.0679144	-0.44637235
ESRP1;ESRP2											0.47165656	0.24963252	0.18549304	-0.33968925	0.1877764	-0.195738475
ESYT1											0.02758375	-0.248092	0.00209161	-0.26527913	0.0849754	-0.018426895
ESYT2											0.70821773	0.25218646	0.07407334	-0.10330009	0.0514977	-0.48646609
ETF1											0.69992841	0.66165288	0.53698492	0.18581518	0.8554901	0.308172862
ETFA											3.18724163	0.8372434	2.92920421	0.84323057	1.6425024	0.460214615
ETFB											1.49731137	0.66041692	0.06794112	-0.18077469	0.5948554	0.122647603
EXTL2	+	+	+								4.22892003	3.62811534	2.76700733	2.74325752	0.0389967	-0.984426498
EZR											0.37520952	0.02340062	0.07909283	-0.11540794	0.0209796	-0.398588181
F2R	+										0.36248677	0.10895538	0.30452565	0.00765673	0.0476582	-0.815196991
FABP5	+										0.86420731	1.10134697	0.79431173	1.04468218	0.2300941	-0.15137736
FADS1											2.51951163	1.64076614	1.7393256	1.75343513	0.0737443	-0.771643957
FADS2	+		+								1.50135438	0.82716242	2.288283	2.50047684	2.4665517	1.124749502
FADS3	+										0.25140394	-0.1044502	0.0034974	-1.14460754	0.2096008	-0.301359812
FAF2											0.24471653	-0.1718286	0.37831623	0.13655472	0.2201204	-0.130696615
FAM105B											0.32221256	0.03882726	0.19554947	-0.32313347	0.2009117	-0.236090342
FAM120A	+										0.12159073	-0.1176548	0.3207802	0.0121727	0.594563	0.147778829
FAM129A	+	+	+		+	+		+	+		4.47699809	7.13248062	5.96121583	7.61046727	0.1537654	-0.304320653
FAM129B	+	+	+	+	+	+	+	+	+	+	6.78327047	7.75010681	6.68232605	5.66187032	2.7751117	6.495426178
FAM134C											0.21682694	-0.0229219	0.59709603	0.26327833	0.1551567	-0.371114731
FAM162A	+		+								1.62616286	0.91156006	2.46259485	1.89546267	0.6229073	0.155930201
FAM171A1											0.07499964	-0.2625071	0.34828721	0.08911641	0.0422272	-0.8860569
FAM213A	+	+									2.7269901	1.73079872	0.64338562	0.35800997	2.5465077	1.198824565
FAM219B											0.62645996	0.28458977	0.90919764	0.35267957	0.1067748	-0.583304087
FAM219B											0.1877862	-0.1427129	0.79772282	0.51599566	0.0830802	-0.706588745
FAM26E											0.81909193	0.6947422	0.11374169	-0.40577761	0.0988532	-0.381781896
FAM49A	+	+									4.16075024	4.68658574	0.08382696	-0.44600105	0.0946172	-0.786404928
FAM49B	+	+	+	+				+	+		4.98032551	10.1367327	6.48733702	11.2944819	5.4277392	7.982671102

FAM69B		+									3.95468896	5.07363256	0.48240068	0.32192993	0.1833278	-0.314163844
FAM84B	+	+		+			+	+		+	5.02188646	5.42408816	0.10437285	-0.37600072	5.6827319	7.251331965
FAM98B	+										0.3364271	0.08565776	0.17251915	-0.26857694	0.4262718	0.154608409
FANCI											0.76346866	0.35679754	0.31422614	0.01913961	0.0095798	-0.855333328
FARSB											0.02644986	-0.2988949	0.04098121	-0.20885785	0.0810023	-0.0866038
FAS		+									1.72728016	1.95282364	0.00477331	-0.7399985	0.1452647	-0.215178808
FASN											0.02258241	-0.2188791	0.0003326	-0.48363304	0.003631	-0.243562063
FASTKD2											0.15124725	-0.3808142	0.23634649	-0.10370636	0.2861774	-0.034872691
FBP1											0.71376961	0.28568077	0.02172588	-0.5733312	2.8613053	0.260986964
FBP2											0.01662213	-0.5134907	0.30229983	0.00280062	0.9523944	1.556576411
FBXL2											0.42308389	0.11882337	0.35468898	0.15061124	0.2964444	-0.006267548
FBXL20		+									3.01414895	2.57860184	0.73728351	0.42209053	0.0082317	-0.59083875
FBXO17	+	+	+	+							3.24636676	4.79778163	2.77540443	3.67856598	3.5423387	3.768613815
FBXO22											0.46738079	0.35876592	0.22799016	-0.12835693	0.1725418	-0.32087326
FDFT1											0.37513025	0.0707283	0.97666675	0.69032733	0.0124249	-1.091578166
FDPS											0.48357066	0.03147125	0.99954643	0.17839686	1.6293686	0.454104106
FECH											0.37551487	0.12471771	0.26856969	-0.06742795	0.0032263	-1.571501414
FEN1	+										0.5349291	0.05395508	0.08047838	-0.17214839	0.5663823	0.625286738
FERMT2											0.69712262	0.74540456	0.09273031	-0.31762314	0.0020988	-1.029793421
FH		+									2.53990524	1.9722379	0.88339273	0.22851817	0.7100197	1.026570002
FHL1											0.04357196	-0.650486	0.01316681	-1.06877009	0.0581964	-0.929964066
FIS1											0.26072022	-0.0544326	0.06879755	-0.5675602	0.0052967	-1.211688995
FITM2											0.13903021	-0.3230311	0.19064721	-0.21922112	0.6381716	0.472173691
FKBP10	+										0.02821826	-0.4039497	1.05131278	1.10074997	0.5492111	0.523005803
FKBP1A	+										0.41366246	0.23511632	0.31065779	0.0148646	0.0866808	-0.892053604
FKBP3											0.07177236	-0.6660538	0.62127368	0.34905497	0.0348418	-0.440725962
FKBP4											1.05674873	0.80046336	1.60990317	0.67858696	2.3074272	0.301994324
FKBP8	+	+	+	+		+					3.70385869	3.52238655	3.89131564	5.19484647	2.6304552	1.819066366
FKRP											0.15881465	-0.1991653	0.00169354	-1.58453115	0.0708649	-0.565284093
FLNA											3.51E-06	-1.0962671	7.53E-07	-1.15804799	1.97E-05	-0.717666626
FLNB											0.0003179	-2.7183247	8.49E-05	-1.66112773	1.35E-05	-1.617539088
FLNC											0.31005007	0.02183278	0.01271489	-1.20504888	0.1636694	-0.282691956
FLOT1	+	+	+	+							3.59391919	5.62279765	5.12474142	5.08776983	3.0988449	4.122532527
FLOT2	+	+	+	+							3.2941455	8.12080129	6.12685804	6.9195315	5.1162525	6.643678665
FMNL1	+		+			+					0.06075338	-0.7401409	2.67861679	5.33307139	0.0054361	-1.291004817
FMNL2	+	+	+	+	+	+		+	+		4.38197011	4.820666	4.63973526	5.40697416	4.419052	3.88178126
FMNL3	+	+	+	+					+		4.11175648	3.31604576	4.28669536	2.81344541	3.1953071	2.027724584
FOLR1			+								0.00469051	-0.4372075	4.97051888	3.71756299	0.1336258	-0.549876531
FRK											0.58958632	0.2760334	0.01608601	-0.9503142	0.5699494	0.677031835
FRS2	+	+		+							2.85246321	2.49786504	0.99397551	1.20901934	4.2330637	3.166891098
FSCN1											0.34918851	0.06045532	0.02394749	-0.35798009	0.6793687	0.269050598
FTH1											0.0610639	-0.7309373	0.12921332	-0.56922913	0.1250358	-0.682053884
FUBP1											0.07441455	-0.1153768	0.10027707	-0.09932772	0.002724	-0.476458867
FUBP3											0.07239795	-0.2673461	0.362726	0.10069211	0.0991186	-0.492425283
FUS;TAF15											1.49112705	0.92083422	0.30934671	0.00602468	0.1908995	-0.117577871
FXYD3	+										0.12533079	-0.3512185	0.10525311	-0.49878438	0.5779185	1.292738597
FYN	+	+	+		+			+			4.35563426	5.44122887	3.33414257	2.50769615	0.06205	-0.719819387
FZD6											0.72315473	0.56835874	0.00814984	-0.96076902	0.3369742	0.11209933
G3BP1											0.00420758	-0.7520434	0.01918539	-0.40479088	0.0007031	-0.485544205
G6PD	+	+									1.72675009	2.60231082	0.22017278	-0.04449209	1.2948332	0.102420807

GAA	+										0.52292563	0.31841405	0.13149347	-0.30585035	0.3696749	0.170804977
GALK1											0.68410242	0.43641345	0.1597094	-0.43937874	0.3700735	0.10651207
GALNT1	+	+	+	+							6.55233351	4.23638598	4.01357947	6.00776927	5.0123854	3.895788829
GALNT2		+	+	+							2.47933138	2.62194316	4.41587686	4.35594813	2.0173362	2.415880203
GALNT3											0.22122281	-0.1141478	0.0491406	-0.48535665	1.7933823	0.706566493
GANAB											0.00338613	-0.5588913	0.00104158	-0.59520531	0.0041471	-0.391438166
GAPDH	+										2.31121662	0.71650441	3.08358792	0.60067495	4.1133359	0.568647385
GAPVD1											0.02127127	-1.2158623	0.10148317	-0.56113879	0.0684233	-0.76108551
GARS											0.49257525	0.06786919	0.04615567	-0.25167656	0.0929276	-0.154322306
GART											0.23425241	-0.0397434	0.02386173	-0.14625804	1.0893531	0.24610583
GBE1											0.45040443	0.180336	0.21115814	-0.06593641	0.0165935	-0.42191124
GBP2;GBP1											1.05078425	0.3783048	0.07700071	-0.51541265	0.5516559	0.193101247
GCAT											0.08347762	-0.2876561	0.46323998	0.30853335	0.2463892	-0.112920761
GCN1L1											1.13989494	0.74140867	1.9278205	0.36403847	1.8119891	0.710345586
GDA											0.49988061	0.14040693	1.02756004	0.64730072	0.0184337	-1.271627426
GDI1											1.14339547	1.21493022	0.27629746	-0.05622037	0.0915419	-0.754275004
GDI2											1.53642953	0.31522814	0.50506994	0.05424182	2.3526641	0.197141647
GDPD3											0.27447631	-0.0447191	0.5099117	0.50890414	1.1642755	1.507785161
GEMIN4											0.00883182	-0.8103091	0.42882923	0.15228589	0.1049879	-0.331583023
GEMIN5	+										0.18465887	-0.1559073	0.07793824	-1.92775281	0.3886267	0.57961146
GFM1											0.07540845	-0.4700947	0.60104118	0.36521975	0.0167088	-1.095241547
GFPT1;GFPT2											0.19232125	-0.3172003	0.08457755	-0.95517667	0.0066595	-1.474719365
GFRA1				+							0.70970702	0.49721209	0.12940188	-0.35279592	1.5872312	2.200182597
GGH											0.71446016	0.62494723	0.09002429	-0.6373717	0.0468513	-1.026980718
GGT1;GGT2;GGT3P			+								0.4380358	0.25673866	3.0729633	2.72571691	0.0029732	-0.553499222
GHITM											0.10892317	-0.4307912	0.19136061	-0.35136223	0.1932592	-0.316895803
GIPC1											0.06312856	-0.5609595	0.07156061	-0.77948443	0.1330261	-0.478380203
GLCE		+	+								3.00409126	3.55502955	2.31215486	1.90390778	1.2704642	1.461478551
GLG1		+	+	+							2.92050275	3.6289374	2.83157168	2.31325658	3.1951329	3.979815801
GLIPR2	+	+	+	+							3.09267908	3.92315038	2.73859702	4.96560733	4.0110337	2.269611994
GLO1											0.57857908	0.13533592	2.69371467	0.76220767	1.2426392	0.090297699
GLOD4											0.03410777	-0.9911353	2.47727492	1.73232587	0.612611	0.837867101
GLRX3											1.89981434	0.53678703	0.11927507	-0.1121006	0.0813132	-0.336661657
GLS	+										2.14441375	1.34406471	2.41364027	0.92166265	0.0376818	-1.192524592
GLUD1;GLUD2											0.79722501	0.20941162	0.27310792	-0.01944606	0.0058869	-0.313387553
GMCL1;GMCL1P1	+										1.47837041	1.37646039	2.31246377	1.00416692	1.9806211	1.381787618
GMPS											0.28092565	-0.0052624	0.01047413	-0.26210912	0.6220793	0.039857864
GNA11		+	+	+							4.4314023	6.27983729	3.5315019	4.65686035	4.5048709	4.945361455
GNA13		+	+	+							3.34418267	4.79170863	1.99993541	2.36041196	3.6431361	5.373849869
GNAI1	+	+	+		+	+	+	+	+	+	5.08363374	5.68108686	3.64380089	5.9722964	0.0182498	-1.406394323
GNAI2	+	+	+	+	+	+	+	+	+	+	9.31370067	10.1209405	4.04526751	9.30962563	4.911369	7.63193957
GNAI3	+	+	+	+	+	+	+	+	+	+	6.268279	7.60951805	4.10571393	8.24841118	5.0347129	8.972011566
GNAO1	+		+			+			+		0.79704441	0.26836014	5.9552134	5.68412908	0.0004594	-1.222032547
GNAQ	+	+	+	+							3.13536022	2.57167244	3.30648998	1.93555705	3.2823565	2.828659693
GNAS	+										1.83052121	1.29098765	0.96595186	0.73052216	2.9233333	1.372346878
GNAZ	+	+									3.07755157	4.0822614	0.00207609	-2.07109133	0.5990614	0.320489883
GNB1											0.02505883	-1.1432228	0.04465225	-0.49092293	1.2609622	0.630425771
GNB2											2.16228958	1.43829664	1.91597623	1.10256449	3.6239524	1.506601334
GNB2L1											0.25703099	-0.0307636	0.00036575	-0.39436086	0.0012604	-0.151842117
GNG12											0.03860825	-0.762516	0.06845567	-0.6154925	0.0162928	-1.314345042

GNPDA1											1.49955744	1.91841189	0.29770161	-0.01203028	0.0462851	-0.614861806
GNPNAT1											0.75399994	0.42662938	0.32803308	0.0419534	0.7707168	0.784063975
GNPTAB											0.25028138	-0.0868594	0.55467223	0.22233582	0.2585075	-0.052130381
GNPTG											0.19619754	-0.1704865	0.05392509	-0.79312007	0.45103	0.258252462
GOLGA7		+	+	+							4.17891264	4.52689234	4.79522018	3.25000509	3.7433301	4.253088633
GOLGB1											1.34248339	0.97407595	0.45527372	0.28732808	0.1344801	-0.514966965
GOLIM4	+	+	+	+							3.96861408	5.47024536	5.71327729	6.73948924	3.513413	2.733587901
GOLT1B											0.63697345	0.28158251	2.27568018	1.58476702	0.9722063	0.52358373
GORASP1	+	+	+	+							4.94967819	4.34984907	5.16563293	4.47263654	4.3463068	4.736963908
GORASP2	+	+	+	+	+	+	+	+	+	+	7.41582407	8.80909411	4.55277458	8.49254672	3.8050366	8.679632187
GOT1											1.09554272	0.31047885	1.80597738	0.40171305	0.114682	-1.044118881
GOT2											2.05939144	0.46137555	2.03650542	0.28905869	0.3564261	0.025526047
GPAA1	+		+								0.85168826	0.83273633	2.02574557	1.97244072	0.1821457	-0.451859792
GPC1			+								0.9049104	0.22341283	3.5217528	2.85770035	0.1176848	-0.662508647
GPD1L											0.66820218	0.51671727	0.04608351	-0.5135053	1.2964962	0.806688944
GPD2											0.02707519	-0.7863185	0.35313166	0.14325714	1.094234	0.194588979
GPHN			+								0.32817151	0.03002548	3.10236811	2.25838407	0.1025373	-0.681810379
GPI											2.17143742	0.63913155	2.23599555	0.42472458	1.8585701	0.408471425
GPM6A	+	+									5.74474845	3.63138771	0.51113463	0.31004333	0.216332	-0.213371277
GPM6B	+										0.18404748	-0.3047314	0.35828036	0.06336149	0.0545553	-0.764411926
GPR157											0.51612977	0.25567055	0.11116853	-0.34901047	1.2452904	1.085009257
GPR37											1.03859042	0.92830149	1.20663002	0.80713463	0.1533589	-0.266992569
GPR89C;GPR89A;GPR89B			+	+							0.10658144	-0.4620482	3.57090432	2.22958755	3.1313661	2.258241653
GPRC5A		+	+	+							4.25179595	4.90094185	5.63046523	3.40947405	2.9979229	4.058506012
GPRC5C	+	+									2.62024886	2.32784462	0.07518098	-0.40365028	2.5668671	0.801980337
GPS1											0.15511896	-0.7058589	0.00900013	-1.29999097	0.794294	0.861548742
GPX8			+								0.63895875	0.94920158	3.58476859	4.13194466	0.2779367	-0.048212687
GREB1	+			+			+			+	0.79328917	0.45888201	0.68338178	0.6241188	5.0184479	4.650143941
GRHPR											0.8493502	0.96385892	1.1292763	0.90685399	0.1728553	-0.366333008
GRPEL1											0.30741481	0.01035881	1.5528872	1.12805494	0.306174	0.013565699
GSK3B											1.09534843	1.01113955	0.68108572	0.10152245	0.6936976	0.606488546
GSPT1;GSPT2											0.16592329	-0.090498	0.20603325	-0.13538933	0.210357	-0.076087316
GSR											0.43815877	0.07121531	0.67456978	0.18851789	0.1285844	-0.233560562
GSTK1	+		+								0.79217428	0.67271614	3.51707517	2.49600474	0.1733464	-0.216487249
GSTM1;GSTM5;GSTM	+										0.02022354	-0.6683083	0.54034961	0.10126813	0.0499984	-1.174870173
GSTM2											0.95545741	1.11577161	0.10311484	-0.61788686	0.0222683	-1.082190832
GSTM3											0.83797739	0.49812444	0.71325621	0.45450783	2.8218841	0.623782476
GSTO1											2.30596914	0.83684285	1.55970157	1.00345039	1.0947885	0.600323995
GSTP1											2.76856077	0.63439178	0.88786975	0.20249494	0.0580405	-1.213240941
GTF2I											0.72909458	1.0300204	0.50299544	0.43615595	0.0779149	-0.43384552
HADH	+	+									1.69916561	2.06105169	0.42153018	0.29390272	1.0681383	1.503864924
HADHA											3.6003254	0.90464083	2.02540676	0.56910642	2.6915664	0.554059347
HADHB		+	+	+							4.13357846	7.39025815	4.60174812	7.73169327	5.8130535	3.834447861
HAGH											1.39141355	1.38620122	0.19222743	-0.19467799	0.5509038	0.286287944
HARS											0.50153524	0.1148955	0.925772	0.18773969	0.6887042	0.093955994
HCCS	+	+	+	+	+	+	+	+	+	+	3.85464864	6.29779371	5.98763813	8.87877401	4.6887078	6.124697367
HDHD3		+									2.9640913	2.15610504	0.78125254	0.32909203	0.0192371	-1.043015798
HDLBP											0.15852973	-0.3667247	0.38443802	0.12332726	0.0233074	-0.489572525
HEATR2											0.62556869	0.13970311	3.09965726	0.31371752	0.0010542	-0.45013682
HEATR6											0.12146747	-0.2547925	0.58445118	0.40274302	1.1108639	0.406549454

HIBCH	+										0.04696671	-0.4887842	0.00559571	-0.68312263	0.0080952	-1.823669434
HID1	+	+		+				+		+	3.79145918	3.75686264	0.30734629	0.00619634	3.070682	5.037316004
HINT1				+							0.45533849	0.41923523	1.25828375	2.49507395	2.5912481	2.334988912
HIST1H4A											0.93800293	0.70968946	0.00039837	-0.89717166	0.3167184	0.052054723
HK1	+										0.12916882	-0.1788394	0.01114682	-0.47472509	0.0040186	-0.438290914
HLA-A											0.41681518	0.17155202	0.13553604	-0.18518575	0.0017747	-1.479075114
HLA-A											0.27138458	-0.0366847	0.19039865	-0.21349271	0.0304452	-0.802918752
HLA-B		+	+								2.45087817	3.25694656	4.03195658	4.93816821	0.0694261	-0.796258926
HLA-B			+								0.22473007	-0.1452198	3.02686448	2.24069468	0.2099434	-0.284390767
HLA-C											0.08479735	-0.6702302	0.24939397	-0.11671575	0.0740216	-0.346232096
HLA-C											0.70597398	0.54769389	0.84114244	1.47267469	0.0613428	-0.659070969
HLA-C			+								1.64895024	1.13612302	4.13917462	8.00323423	0.0341661	-1.52835083
HLA-C											0.52318385	0.25349744	0.72750679	1.47137388	0.0578964	-0.777512868
HLA-G											0.46999443	0.38553492	0.63342699	0.9569912	0.2487283	-0.138451258
HM13		+	+	+							1.51318441	2.47542254	2.09965507	2.71278191	3.3225885	3.579339345
HMGB1	+										0.05099016	-0.5748895	1.99583922	0.82263565	0.0566188	-0.343058268
HMGB2	+										0.94888368	1.4538428	0.09010116	-0.90030162	0.0195234	-0.462620417
HMGB3											1.01041444	0.85383733	0.08555153	-0.45424398	0.9057666	0.712926229
HMGCS1											0.19089332	-0.1304506	0.42332541	0.28281021	1.2841056	1.700280507
HMOX2		+									3.46277613	2.25895691	2.69711609	1.52414195	2.3298584	1.415018717
HNRNPA1;HNRNPA1L2											0.04618686	-0.2661215	0.00353486	-0.49687958	0.2476761	-0.024890264
HNRNPA2B1											0.01330052	-1.2265644	2.17166631	1.18310738	0.7594586	0.634511312
HNRNPA3											0.20497177	-0.2823175	0.03968399	-0.50822639	0.048644	-1.037352244
HNRNPAB											0.25406587	-0.0352834	0.0233336	-0.3837471	0.8688979	1.283812841
HNRNPD											0.87156597	0.22538884	1.28607431	0.27883212	0.0110628	-0.367201487
HNRNPF											0.76608886	0.20836004	0.88628842	0.45483144	0.7577759	0.289342244
HNRNPH1	+										0.081047	-0.3370756	0.73344526	0.14467812	0.0001583	-0.709625244
HNRNPK											1.03079989	0.06758499	0.00029081	-0.44717471	0.0044356	-0.314374924
HNRNPL											0.00373503	-2.041153	0.44258965	0.47766113	0.0005504	-2.083780289
HNRNPM											3.04321576	0.43492254	0.5027161	0.07068443	1.63451	0.438317617
HNRNPU	+										0.00120852	-0.920948	9.35E-05	-1.53387451	0.0037081	-0.881766001
HNRNPUL1											0.02374476	-0.8302492	1.61984866	0.48698997	0.0678789	-0.985623042
HPCAL1	+	+	+	+							4.93876533	6.44671822	4.25866038	10.6755428	4.4298711	8.979324341
HPRT1											2.25282706	0.47237841	1.02974368	0.1799914	2.4934969	0.238583247
HRAS											0.01141562	-0.7027645	0.08481857	-0.29031817	0.0094165	-0.828627904
HS6ST2											1.06614502	1.15905253	0.21357052	-0.11361567	0.1861613	-0.25422287
HSD17B10				+							2.07771737	1.50456429	3.23647464	1.61381658	1.7211054	2.819775899
HSD17B11											0.23991079	-0.1554317	0.3910197	0.09142049	0.2348709	-0.241964976
HSD17B12		+		+							4.03377434	1.68545659	1.65824158	1.92188454	3.5340989	2.730750402
HSD17B4	+										4.44056969	0.88772265	3.20624583	0.66321627	1.7188066	0.53268369
HSDL1											0.1415462	-0.5590776	1.51703702	0.47481346	0.7216576	0.514274597
HSDL2		+									1.64915514	3.95054436	2.33190281	1.22477086	0.454887	0.317022324
HSP90AA1	+										0.22323775	-0.0377909	0.26390519	-0.00482241	0.9173616	0.078255971
HSP90AB1											0.37602403	0.04215304	1.2172368	0.06952413	1.4096603	0.216312408
HSP90AB2P											0.25407078	-0.0963955	0.00153518	-0.73860041	0.5683311	1.030577977
HSP90AB4P											0.34958802	0.04416847	0.63630829	0.22380511	0.1065548	-0.661131541
HSP90B1											0.00103109	-0.3292599	1.03E-05	-0.5567805	0.0029244	-0.396656036
HSPA1A											1.15084131	0.23447609	1.54962293	0.19451523	1.937131	0.262213389
HSPA4											0.30254632	0.00089836	0.14295746	-0.10251172	0.1885753	-0.029506683
HSPA4L		+									1.79390941	2.4919459	0.70257488	1.1152668	0.2685535	-0.104415894

HSPA5											0.14864562	-0.0960147	0.01523602	-0.23049609	0.0070031	-0.297583262
HSPA8											0.47369404	0.05751864	1.10089613	0.09555626	1.4676702	0.111654282
HSPA9											1.59841408	0.30593554	0.26103867	-0.00918134	0.0040123	-0.153834025
HSPB1											0.81437574	1.43396568	0.00541917	-0.25444857	0.0139563	-0.255418142
HSPBP1	+										1.60284474	0.94538498	1.44102065	1.35532824	1.5978028	1.676843643
HSPD1											4.33833858	0.7544651	1.7583511	0.30384318	1.7914965	0.269582113
HSPE1	+										0.05190972	-0.8038241	1.80131446	1.3231945	0.0577619	-0.851074219
HSPH1											0.17088302	-0.0815207	0.18140691	-0.10332553	0.7076096	0.271602631
HUWE1											0.09429678	-0.6283627	0.61171036	0.42125384	0.0313372	-1.00440979
HYOU1											0.99343308	0.66259956	1.83019495	0.28660711	0.066764	-0.183389028
IARS	+										0.00232761	-0.646019	0.00194225	-0.65336863	0.0030741	-0.410315196
IARS2		+									3.51530116	3.05298869	0.7535955	1.10603333	0.0153986	-0.229042053
ICOSLG											0.51925275	0.35995547	0.15173243	-0.36775144	0.1664411	-0.424248377
IDE											1.3392494	1.11913045	0.11132505	-1.0064373	0.3828248	0.282445908
IDH1		+									3.08737192	3.16271337	0.02768704	-0.27922249	0.512639	0.12112236
IDH2											0.56867275	0.1354154	1.25923664	0.45948855	0.2464648	-0.020498276
IDH3A											1.0728205	0.39842733	0.67388577	0.25043488	1.0584049	0.320230484
IDH3B											0.18640182	-0.2025038	0.00326972	-1.35793304	0.1495278	-0.292936961
IDH3G											0.06808162	-0.591177	2.27646738	0.97993787	0.0788946	-1.001606623
IDI1											0.42179182	0.13839086	1.32012639	1.20952797	0.5186094	0.102424622
IER3IP1											0.04307248	-0.452076	0.08443126	-0.52570089	0.2337537	-0.128123601
IFITM1;IFITM2											0.13998652	-0.347386	0.00297573	-1.16136551	0.8952004	1.344120661
IFITM3		+	+	+							4.60164976	6.96433703	5.18059062	7.95887629	5.5625121	6.59176
IGF2BP1											0.01805147	-1.029583	0.33894725	0.07881419	0.001361	-0.913405101
IGF2R	+										0.43644675	0.16017977	0.21391616	-0.33657201	0.4428493	0.229853948
IGHMBP2											0.38018287	0.16589101	0.02032053	-0.58871269	0.0319236	-0.916496277
IGSF8	+										0.07483172	-0.6268628	0.78315569	0.50531705	0.5072186	0.393955231
IKBKAP											0.00320728	-0.6057854	0.83762384	0.64974403	0.1025205	-0.45783488
IL27RA											0.10214294	-0.4933109	0.01805863	-0.89004072	0.0638024	-0.821365992
ILF2											1.54110643	0.39400991	1.72394374	0.36492856	1.1552078	0.319038391
ILF3											0.0020379	-0.785003	0.26481543	-0.01959419	0.0995132	-0.223464966
ILK											0.22272524	-0.0712026	0.66879121	0.40562185	0.0824773	-0.576644262
ILVBL				+							1.34584571	1.46690241	2.28513879	1.86665535	1.7152696	2.277128855
IMMT											0.51741826	0.10715548	1.73886965	0.48971939	2.9058179	0.626241048
IMPA1	+										0.75728203	0.15973091	0.44219387	0.20720673	0.0109244	-0.7416598
IMPDH2											0.0108585	-0.6276925	0.001143	-0.40648715	0.0226229	-0.158159892
INF2											0.12872052	-0.2643433	2.12545203	1.33396594	1.1797535	1.191595713
INPP4B	+			+							0.44307551	0.19993782	1.09489061	0.93000793	1.1951421	2.629969279
IPO4											1.0524419	1.50580661	2.71789393	0.47377141	2.2934957	0.572877248
IPO5											1.38457005	0.62030411	2.46386557	0.26061948	2.2728415	0.716913859
IPO7											2.31814304	0.64154689	0.36908164	0.02109591	0.5927264	0.190209707
IPO8											0.58756147	0.42372576	0.06923872	-0.81378619	0.0215267	-1.072859446
IPO9											0.75741217	1.25512886	0.92460887	0.08839544	0.778308	1.226725896
IQGAP1	+										0.06035538	-0.1497472	1.59628625	0.14689382	0.0873263	-0.11177063
ISOC1											0.04666515	-0.5888983	0.0490523	-0.84983508	0.2061026	-0.215583165
ISOC2											0.16663089	-0.28523	0.15033721	-0.38386981	0.224356	-0.094287872
ISYNA1											1.44327944	0.22653707	0.01878124	-1.17666562	0.1028828	-0.422449748
ITFG3		+	+	+							3.97128097	2.86708387	3.51751799	3.8582147	3.4557258	4.052039464
ITGA3	+		+								0.91969691	0.5743796	3.53990937	2.61924553	0.2905085	-0.019210815
ITGB1											0.32717308	0.04140981	1.25182412	0.6051801	0.1629044	-0.397158305

ITGB4			+	+							0.45493987	0.16292445	3.33318244	3.48405774	4.1497142	5.836261749
ITM2B		+	+	+							4.79153798	4.76523145	3.75632046	5.61729622	3.845197	4.154172897
ITM2C	+	+									3.21710666	2.44760895	0.0989031	-0.46250661	0.2141239	-0.274885813
ITPRIP											1.26317138	1.01190631	1.4603813	1.04598427	0.1730268	-0.3865846
JAM3		+									3.13516527	2.80952644	0.38417488	0.10012563	0.118592	-0.435419718
JUP											0.23837807	-0.1013298	0.18635712	-0.39191246	0.2424376	-0.036872864
KALRN											0.39906572	0.47149022	0.83521525	2.15197563	0.4173183	0.888299306
KARS											0.01582539	-0.4995918	0.00883112	-0.59447797	0.0163137	-0.34575208
KATNAL2											0.43546481	0.14486504	0.07011383	-0.73894501	0.5202676	0.426019669
KCNB1											0.23974316	-0.0979646	0.44466844	0.58443705	0.0128226	-1.284200033
KCNK1											0.23852394	-0.0671844	0.08856613	-0.63793246	0.2097579	-0.399623871
KCNK1											0.08306261	-0.4969076	0.10826213	-0.28984578	0.3485813	0.195427577
KCTD12											0.05182768	-0.8621833	0.39735207	0.0459315	0.3243144	0.053423564
KDELR1											0.08971855	-0.402078	0.84691639	1.14298884	1.0214992	1.046150208
KDELR2											0.61437324	0.35773722	0.11725533	-0.36085129	0.4834135	0.160139084
KHDRBS1											0.12986532	-0.3013121	0.00314858	-1.20445251	0.0552775	-1.329264959
KHSRP											0.2526597	-0.0216732	0.16930213	-0.08735212	0.2107906	-0.065047582
KIAA0319L		+	+								4.18270443	4.22057978	2.60006065	2.77339554	0.0265307	-1.04996109
KIAA0391											0.04127597	-0.8992399	2.08955374	1.32965279	0.7175432	0.384751002
KIAA1324				+							0.53461064	0.33642324	0.36252638	0.11517398	5.09606	4.054335912
KIAA1522	+										0.62079934	0.35018412	0.00092119	-1.30914179	0.2198302	-0.223823547
KIAA1967											0.61385876	0.3460331	0.18937642	-0.24766032	0.0477064	-0.620564143
KIAA2013		+	+	+							4.86585451	4.52311071	3.33299909	4.11973699	4.476649	4.294262568
KIF11		+									2.71975601	3.02303696	1.86613328	1.10844231	0.0101001	-1.645659765
KIF16B											0.0485162	-0.5042082	0.48491529	0.21789996	0.7063274	2.78133138
KIF5B											0.00456126	-0.7516162	0.41719932	0.18779055	0.0054632	-0.320532481
KPNA2											2.62263265	1.08185387	2.88611076	0.66492844	3.1344995	0.754588445
KPNA3											0.39556012	0.12255732	2.55240379	1.46395493	1.2957667	0.994021734
KPNA4											0.46840173	0.30394046	0.55679888	0.25438436	0.0078666	-0.533994039
KPNA6;KPNA5											1.66991553	1.51425298	2.03884152	1.47798411	0.4192094	0.266920726
KPNB1											2.06333307	0.69036674	3.46591119	0.29214795	2.0404046	0.468262355
KRTCAP2											0.2007615	-0.3004112	0.57087379	0.3214124	0.2784238	-0.06226031
KRTCAP3				+							0.02068787	-0.9049829	0.28191633	-0.04138629	2.7306363	2.496193568
KTN1											0.05307022	-0.7178485	0.7131936	1.49824079	0.213853	-0.170120875
KYNU											0.16958068	-0.2766864	0.96997978	0.52067312	0.0543381	-0.731241226
L1CAM			+	+							0.09642186	-0.3805904	4.34760961	5.90914154	3.8376278	3.634588242
LAMP2											0.29397308	-0.0149479	1.08851355	0.43089549	0.1008875	-0.442792257
LAMTOR1	+	+	+	+	+	+	+	+	+	+	6.47375112	8.93641917	6.04900013	9.51441701	5.5335043	8.87793986
LANCL1		+	+								1.34432822	2.20770454	3.38404233	1.95739555	1.0613334	0.925773621
LANCL2	+								+		1.83941566	1.31433996	0.64645963	0.42431005	1.5086481	1.014919917
LAP3											0.49622754	0.3056221	1.03562904	0.14957428	0.0284031	-0.253377279
LAPTM4A											0.24223255	-0.1641394	0.21524947	-0.19430542	0.0699741	-1.009725571
LAPTM4B											1.20058031	1.28087743	0.66457969	0.47228368	1.5443074	0.863245646
LARP1											0.51779116	0.32505989	0.04121826	-0.88783646	0.0210971	-1.272411346
LARS											0.58262301	0.09030596	0.00222388	-0.46627998	0.2160576	-0.0554142
LASP1											0.04822081	-0.782397	1.02192915	0.79734039	0.0106202	-1.183109283
LBR				+							3.18684023	1.09272766	2.56047967	1.29583041	2.7788803	2.187222163
LCLAT1											1.04201759	0.46887589	0.58452457	0.84250387	0.3154875	0.019926707
LCP1											1.9726636	1.02668826	0.35746213	0.11880366	0.0067324	-0.363320033
LDHA	+										4.73478777	0.8528614	3.9922203	0.62451045	2.8881378	0.58667193

LDHB											2.98164911	0.73479907	2.97272762	0.47172038	2.3518965	0.382830302
LEMD3											0.28819071	-0.0269737	0.00593103	-1.59495989	0.1217007	-0.394499461
LGALS1											0.11986345	-0.2830118	3.18540868	1.42093913	0.0490371	-0.317265193
LGALS3											0.02725226	-0.8986855	1.20178502	1.60842705	0.4042437	0.093779246
LHFPL2											0.69227002	0.29378764	0.11168427	-0.28458722	0.6602546	0.87303861
LITAF											0.04671455	-0.6294346	0.93882598	0.87872823	0.1975677	-0.277767181
LMAN1		+									3.31996569	2.36275927	0.51649774	0.22387314	0.0697313	-1.225779215
LMAN2											4.26551104	1.22537549	2.70102053	0.8049132	2.1193935	0.449120204
LMBRD1		+	+	+							3.8035014	5.75817108	4.22100074	3.37301954	3.5505385	5.098573685
LMBRD2											0.09695794	-0.6447716	0.43248908	0.24539693	0.6955322	0.861684799
LMF2											1.57170141	1.4508934	1.8658168	0.63171832	0.0312524	-1.42694219
LMNA	+	+									2.3502934	1.95625432	0.01833321	-1.38193194	0.2474186	-0.136542002
LNP;KIAA1715	+	+	+	+		+				+	5.75074033	7.10627683	5.13063926	6.95152855	5.2745033	5.874843597
LNPEP		+	+	+							3.73076805	5.17953173	3.61289948	6.47251511	4.662415	6.557732264
LOH12CR1	+	+	+	+		+	+		+	+	5.5393568	6.06525803	3.83972634	5.52510961	3.6098147	4.600874583
LONP1											0.96487631	0.36851501	0.44274362	0.05327606	0.2522113	-0.040003459
LPAR1											0.63234158	1.27621078	0.00634413	-0.3701992	0.3841198	0.120534897
LPAR2				+							0.07153209	-0.8818423	0.02050633	-1.45820872	2.2846096	2.082605362
LPCAT1											0.31786751	0.03825188	0.02338045	-0.45878855	1.2086815	0.635445913
LPCAT2											0.05750779	-0.3448753	0.50978023	0.49723752	0.0071235	-1.174327215
LPCAT3											0.88067832	1.03651428	0.4971886	0.33944194	0.5084868	0.467777888
LRPPRC											1.57003209	0.4336071	2.29114123	0.29047648	3.1839993	0.420895894
LRRC1		+									3.85562543	3.89818001	0.00927017	-1.12109884	2.2080031	1.363084157
LRRC15			+								0.14790186	-0.1778037	3.48262626	4.81896655	0.041775	-1.178532283
LRRC47											0.14363748	-0.3470815	0.43638365	0.26624044	0.2155805	-0.12489446
LRRC57	+	+	+	+	+			+	+	+	4.93101003	6.93420664	4.8540418	7.90079308	4.1423864	6.115076065
LRRC59											1.17087644	0.46568298	2.23858866	0.58579826	1.2586071	0.336746216
LSR											0.30175167	0.00170453	0.24081895	-0.10972341	0.1424456	-0.712493896
LSR		+		+							4.17233086	7.64582189	0.37968148	0.14040375	5.0433068	9.017170588
LTA4H											1.25703219	0.16676648	0.28413056	-0.00856209	1.8582294	0.192620595
LTBR	+		+	+							1.56984816	1.91077423	2.72085234	3.20270157	2.6560385	2.06153361
LUC7L											1.15247464	0.45868047	0.13988919	-0.31890233	0.0651287	-0.561273575
LUC7L2											0.17238225	-0.5401427	0.05205965	-0.93955294	0.0082837	-0.907981237
LUC7L3											0.0009926	-2.5667292	0.11487357	-0.88176473	0.0181478	-1.980040232
LXN											0.18268351	-0.3064823	0.0572515	-0.80441221	0.233682	-0.039987564
LYN	+	+	+	+							5.35328914	6.75140699	4.3514704	6.26036644	3.3422397	5.767993291
LYN	+										0.01940005	-0.8833148	0.5352196	0.21340243	0.1609477	-0.164607366
LYPLA1		+	+	+		+	+	+	+	+	3.04931534	3.61111895	3.93936452	4.54141998	1.933543	3.01680692
LYPLA2		+	+	+	+	+		+	+	+	3.24906659	4.13658841	3.54837497	5.30210368	4.5861018	4.916671753
LYST		+	+	+							4.52327071	4.45294571	4.47102557	5.17369397	3.3154017	3.853932063
LZTS1	+	+									2.09873456	2.64598401	0.00038308	-0.7907505	0.0143699	-0.728366216
M6PR		+	+	+							3.05485472	3.38504728	4.04719961	2.64159584	4.9626118	5.876724879
MACROD1											0.63175592	0.49461683	0.44422473	0.22023392	0.1461986	-0.549970627
MAGED2											0.32851197	0.05412992	0.05724982	-1.11813672	0.0889248	-0.632977168
MAGT1											0.15463905	-0.2916718	2.37600699	1.44770749	0.0403343	-1.023736954
MAL2											0.02736732	-0.402146	0.02805337	-0.78856277	0.7436111	1.41007487
MAN1B1		+	+	+							3.81079316	5.59074084	3.40921701	5.52575366	4.3602161	5.493925095
MANEA		+									2.49190751	3.21825536	0.41093412	0.15194384	0.1852532	-0.239210765
MANF											0.29580757	-0.0135797	1.02392399	1.35087013	0.0271835	-1.331272125
MAP2K1											1.02438517	0.78070323	1.21497216	0.61020851	0.5578312	0.295293808

MAP4											0.06685154	-0.653169	0.18302453	-0.39918582	0.0908272	-0.293501536
MAPK15											0.40575424	0.16937637	0.39314767	0.13147163	0.4528394	0.148155848
MAPK3											1.19280245	1.13277944	1.20109504	0.90181669	0.0557188	-1.247820536
MAPRE1		+									2.14434184	1.82352002	2.19134501	1.4439106	0.5640011	0.44603157
MARC1	+	+	+	+		+			+		3.60664469	4.27683322	6.0700162	7.78646914	4.2863587	2.843940099
MARC2	+	+	+	+	+	+	+	+	+	+	5.17147448	6.07596842	5.45167494	5.95340093	3.2718784	4.67837588
MARCH5											0.27181904	-0.0292937	0.14424006	-0.2549572	0.2217905	-0.178391139
MARCKS	+	+	+	+		+		+	+	+	4.68362472	10.7238903	5.47973245	9.74790382	6.125119	8.027525584
MARCKSL1	+	+	+	+	+	+		+	+	+	3.65100287	7.39850044	4.63578085	6.31986872	4.9295848	5.51997757
MARS				+							1.16676179	1.98648135	0.43756253	0.34441503	3.111454	3.0930837
MARVELD3				+							0.46503362	0.31885974	0.17845425	-0.13954989	4.9886906	3.790260951
MARVELD3											0.24753302	-0.0784709	1.0617535	1.0757192	1.0467874	1.129301071
MAT2A											0.59871997	1.03044001	0.09354573	-0.08984693	0.2341504	-0.043160756
MAT2B											0.86354018	0.88647525	0.23512547	-0.14533869	0.0845105	-0.863737742
MATR3	+										0.07129757	-0.9396483	0.17238471	-0.26347351	0.099221	-0.65649732
MBLAC2		+	+	+							5.66249289	4.6233902	4.03034507	3.82241376	5.7777215	2.963743846
MBOAT7	+		+	+							3.54954011	1.40752538	3.90234088	2.50257174	3.6019738	2.558966955
MBP	+		+	+							0.92244365	1.35317421	2.20162864	3.42306709	2.2903918	2.657636007
MCAM	+	+	+								2.37970604	3.8057874	4.29413071	5.22687403	0.0914053	-0.677944819
MCAT											0.08640559	-0.9135787	0.56169155	0.20438703	0.0944407	-0.59947904
MCCC1											0.00010683	-2.5509351	7.03E-06	-2.62201118	6.40E-05	-1.60724322
MCCC2		+									2.5909416	2.47574997	0.48633469	0.41400782	0.4963488	0.5051651
MCM2											0.12716448	-0.1335646	0.00192373	-0.77281443	0.0319823	-0.157625198
MCM3											0.11281504	-0.1140677	0.00057186	-0.52955055	0.0149093	-0.382765452
MCM4											0.11771583	-0.2994671	0.10342658	-0.33061409	0.0065557	-0.773474375
MCM5											0.20898687	-0.0564919	0.04407439	-0.17923482	0.0166103	-0.135240555
MCM6											0.00635404	-0.2164281	0.40102269	0.03646596	0.0058232	-0.318674088
MCM7											0.10518572	-0.2231102	0.12195517	-0.15301323	0.0042191	-0.141233444
MCMBP											0.25396572	-0.0573928	0.0741561	-0.67988586	0.0756851	-0.316055934
MCOLN1		+	+								2.70033907	3.28184446	3.23865337	3.46015612	0.4270099	0.378112793
MCTS1	+										0.79571512	0.42491468	0.24798006	-0.02916908	0.8864746	0.524090449
MDFIC											0.06960959	-0.4435139	0.32985573	0.05781047	0.0457103	-0.75482432
MDH1											2.29199203	0.48768298	1.26121284	0.25932185	1.2337432	0.236783346
MDH2											3.30300812	0.67751185	3.1884044	0.42747053	2.7689142	0.24276034
ME1	+										0.7968603	0.55019442	0.00207334	-0.4703242	0.0389218	-0.130964279
ME2											1.54320992	1.00034205	1.06198404	1.64303589	0.0434519	-0.57015419
MECR											0.17068421	-0.1332391	0.01508253	-0.92036184	0.393093	0.14860789
METAP2											0.03897557	-0.4831187	0.00046686	-0.95927048	0.0170183	-0.869365692
METTL13											0.06019065	-0.5779076	0.30797495	0.00937398	0.2443641	-0.119582494
METTL7A											1.247488	1.16713969	0.19995828	-0.17273585	0.4466217	0.283754349
METTL7B		+									3.75482877	3.42484601	2.31668589	1.82691701	2.4537308	1.487662633
MFSD10	+										0.22603337	-0.1782417	0.8979732	1.29412079	0.4892041	0.410829544
MFSD12	+		+								0.32819404	0.03354263	4.54213927	4.54830488	0.2326621	-0.169645945
MFSD5		+	+	+							2.76673747	2.96955363	2.99188438	2.06843567	4.2822875	4.278423945
MGRN1	+	+	+	+					+	+	3.41775456	6.60598818	3.57733381	5.8167909	4.3097553	4.812980652
MGST1			+	+							0.20470357	-0.0834796	1.58388636	2.89479256	2.2513213	2.787483851
MGST2											0.64775995	0.4147892	0.14054577	-0.52102216	0.4557722	0.204147339
MGST3		+									2.62123469	2.57082812	1.77187151	1.98235003	0.2500801	-0.182765325
MICA	+		+								0.31565427	0.01656342	2.95119681	3.20887884	1.2264007	1.109831492
MIF			+								1.35371946	0.47420375	1.46976756	4.08033117	0.916681	2.104787191

MLEC		+	+	+							5.52591126	6.24643771	5.07766944	4.52403069	3.9856289	5.701106389
MLKL											1.34594732	1.05927149	0.02281017	-0.55133502	0.3693835	0.108732224
MMAB											0.20364002	-0.1590157	0.08311547	-0.28630892	0.1260949	-0.329537074
MMRN2											0.04064086	-1.0599505	0.10915887	-0.36417135	0.103477	-0.619380315
MMS19											0.23633739	-0.0812683	0.02434536	-0.80509186	0.0908417	-0.572213491
MNF1											0.09667109	-0.6007258	0.15943469	-0.23760923	0.0807142	-0.370713552
MOB1B;MOB1A											0.45633128	0.25058937	0.14352118	-0.24222501	0.193952	-0.148167928
MOGS											0.24437815	-0.0802313	1.88948191	0.57498423	0.7588502	0.29441007
MORC4											0.02138553	-0.787049	0.2862691	-0.02195358	0.6702611	1.653278351
MPDU1		+	+	+							2.04120216	2.57801946	5.03836291	4.13099988	5.7643549	3.601086299
MPI	+										0.24999546	-0.1074142	0.48327393	0.20655441	0.051615	-0.742405574
MPST											0.40610767	0.22657394	1.37653724	1.55266825	0.4417297	0.373093287
MREG	+	+	+	+							2.52264015	2.49457932	2.64827104	2.62550354	5.7578892	6.365106583
MRI1											0.10529955	-0.3553969	2.27583244	0.5241731	0.3539565	0.112798691
MRPL10											1.00496061	0.65725454	0.5933372	0.36197281	0.028229	-1.144856135
MRPL15											0.371692	0.09790039	0.70863303	0.18510373	0.0101163	-0.588260015
MRPL35											0.02859941	-0.3985825	0.43205252	0.18313344	0.1180539	-0.414738337
MRPL37											0.18712781	-0.0275141	0.74400912	0.46897697	0.0687302	-0.822900772
MRPS23											0.21609046	-0.1341858	0.49582977	0.39378611	0.2044019	-0.225817998
MRPS27											1.51382543	0.87205505	1.12842354	0.76854451	1.3610591	1.64139239
MRPS34											0.19249015	-0.1759268	1.09906308	0.88666852	0.0345145	-1.345164617
MRPS36		+	+	+							4.67556703	4.64820035	5.61979155	6.57828458	4.0916576	4.016384761
MRPS7											0.71193754	0.37926165	2.22657619	1.31769244	0.0747867	-0.298774083
MSH2	+										0.72365453	0.14305941	0.0259139	-0.35848173	0.0434761	-0.279987971
MSH6											0.05526692	-0.9495672	0.07332423	-1.15286001	0.8424904	1.123666763
MSLN			+								0.92752619	0.71723811	2.18099859	3.07908694	0.9015683	0.489780426
MSN											0.50009895	0.07172648	0.4791419	0.06943639	0.2263832	-0.014817556
MSRA	+	+	+	+	+	+	+	+	+	+	2.68267119	4.76456388	4.29357959	5.46602122	6.0802731	6.048743566
MTA2;DKFZp686F2281											0.08587148	-0.4193865	0.44811283	0.24344063	0.3003822	-0.001447042
MTAP											0.0412894	-0.8429712	0.03789486	-0.56931369	0.0840663	-1.135178884
MT-ATP6											0.2043728	-0.1672974	0.16862683	-0.25749143	1.4502622	1.182484309
MTCH1	+		+								1.7268671	0.68682098	3.07548988	2.69087855	0.4319256	0.395870209
MTCH2											0.78187718	0.88602893	2.72978367	1.24228986	3.6196818	0.534484863
MT-CO1											0.72646427	0.39789708	0.26389977	-0.11232503	0.4211739	0.374022166
MT-CO2		+									4.34766669	2.06412697	2.62610254	1.22479439	2.0368836	1.824385961
MTDH		+	+	+							4.24476554	4.47084173	6.04065539	5.29586283	4.5537873	3.805036545
MTHFD1											0.28262845	-0.010952	0.00168423	-0.32625262	1.0725762	0.136190414
MTHFD1L	+	+		+							3.53911126	2.86892064	3.17168868	1.37160365	3.1059314	2.377358754
MTHFD2											0.40250218	0.17693901	0.55204472	0.25349998	0.100764	-0.584079107
MT-ND4		+	+	+							2.51467488	2.35892741	2.30958396	2.31770325	3.243627	2.440434774
MT-ND5		+	+	+							2.89695422	2.59965833	3.59389934	2.32055537	3.072042	4.92316246
MTPAP		+									3.50930435	2.33437157	1.63155174	1.2713356	0.0023943	-0.685074488
MTPN											0.19769836	-0.1960411	0.06633117	-0.57895343	0.0390678	-0.661700567
MTRR	+										0.15940736	-0.467186	0.2520645	-0.15115738	0.0944989	-0.970603943
MTUS1	+										0.32318686	0.03331629	0.05612271	-0.78419431	0.3885009	0.207063675
MUC1				+							1.07052908	0.66016388	0.08293064	-0.71724765	3.3558344	4.63476944
MVK											0.58595234	0.3140742	0.09888471	-0.28092066	0.1071076	-0.43391482
MYADM		+	+	+							3.92152109	3.51346143	3.45940561	4.20442645	3.4237444	4.386623383
MYBBP1A											0.74778666	0.53286489	0.90480981	0.82928594	1.634661	1.028526942
MYH14											0.29505771	-0.0142651	0.5073394	0.35372098	0.0961644	-0.485874176

MYH9											0.00184365	-0.7477163	8.27E-05	-0.84974416	6.92E-05	-0.472232819
MYL1;MYL3											0.32606213	0.0322717	0.29768391	-0.00813166	0.0071961	-1.237068812
MYL12A;MYL12B		+									2.90742636	1.63213158	5.41847463	1.33602969	1.8226922	1.395575841
MYL6;MYL6B											1.66052779	0.53374799	1.93279729	0.33654849	1.8891632	0.282486598
MYL9;MYL12A											0.00120295	-2.2442029	0.00025697	-3.64251709	0.5659974	0.303457896
MYO1B											0.88156135	0.31035487	0.25580707	-0.09984652	2.8594248	1.335002263
MYO1C											0.07151193	-0.3716005	0.11517867	-0.57276662	0.8200912	0.712492625
MYO6											0.14376399	-0.364577	0.88673668	0.27134959	0.0218944	-1.287611008
MYOF				+							1.39134995	0.23521296	2.41379796	1.0556132	3.0141498	2.094333013
N4BP3			+	+							0.85570285	0.45349503	3.32713508	2.4713885	5.1004898	4.729222616
NAA15											0.70873107	0.23593394	0.22778542	-0.05064329	0.3917873	0.019892375
NAA50											0.56538177	0.30098025	0.27532862	-0.0499649	0.45554	0.253664653
NAALADL2	+										1.11967677	1.12643433	0.19230208	-0.2002182	0.9813691	0.743501027
NACA											0.25179316	-0.1404444	0.48754374	0.28380521	0.0512679	-0.949689229
NADK2											0.07904091	-0.4291344	0.1786142	-0.26177915	0.6619455	0.382642746
NAE1											0.52496145	0.24643008	0.29169251	-0.01641401	0.07555	-0.433213552
NAGK											0.2380297	-0.1298854	0.11773099	-0.38164584	0.0690169	-1.012070338
NAMPTL;NAMPT											0.12431818	-0.472374	0.56152396	0.07375018	0.397454	0.193068186
NANS											1.2754119	0.95165253	0.56607256	0.58064715	0.8964409	1.029760361
NAP1L1											0.42929292	0.08310636	0.09738315	-0.15991338	0.0685189	-0.258835475
NAP1L4											0.0480161	-0.5533314	0.32708019	0.03622182	0.1676562	-0.20165952
NAPA											1.31537184	1.67173767	2.39063557	0.84558169	0.414071	0.069367727
NARS											0.0445278	-0.2531516	0.06801687	-0.39554278	0.0705239	-0.307144165
NASP			+								1.44079589	0.98087629	2.52379667	3.20817375	0.1539579	-0.472643534
NAT14		+	+								3.12526136	3.84464137	4.12640467	3.97479057	1.1833035	1.879403432
NCALD	+	+									3.17696326	2.52158801	0.08114968	-0.57681402	0.1407323	-0.568187714
NCAM1		+									3.62190114	6.06489817	0.08501109	-0.35930506	0.4579839	0.30172348
NCAM2											0.81700181	0.30007998	0.00312391	-1.76185036	0.6039284	0.392278671
NCAPG	+										0.42852875	0.07191213	1.2339112	1.27962875	1.8328566	0.264692942
NCBP1											0.0792112	-0.5745379	0.34151737	0.05806796	0.4080202	0.217737198
NCDN											0.31155157	0.01342265	0.87889037	0.25856463	0.5457408	0.785482407
NCEH1			+								0.2372594	-0.1029714	2.4954938	3.00078456	1.7520187	0.779649099
NCL											0.01525016	-0.7381941	0.00107794	-0.69786771	0.0014605	-0.738127391
NCLN											0.19163612	-0.2770379	0.32935341	0.01798375	0.0361109	-1.230714798
NCR3LG1		+									1.99705255	2.35203743	0.49475694	0.51185735	0.1729118	-0.273550034
NCS1	+	+	+	+							5.00913766	6.21789742	5.19968274	7.37866084	4.222494	5.657565435
NCSTN			+	+							2.02048237	1.22780037	3.50425441	4.62770398	4.379859	3.24032402
NDC1											1.13002279	0.95513089	0.76813081	0.49412028	0.07313	-0.710988363
NDFIP1											0.27070509	-0.045695	0.1304627	-0.43022537	0.5628077	0.264831543
NDFIP2											0.88875207	0.32793045	0.81618802	0.83971278	0.4390547	0.18620046
NDRG1											0.02604801	-0.7925739	0.19723342	-0.23464521	0.0797355	-0.692146937
NDRG3											0.49538126	0.32664935	0.05105898	-0.82985814	0.2367884	-0.073891958
NDST1											0.93500245	0.73532867	1.03258533	0.77536265	0.6762272	0.28030777
NDUFA10											0.2550978	-0.0756187	0.18512324	-0.17931366	0.1468268	-0.096598307
NDUFA4											2.01322769	0.92391078	1.68263792	1.45333354	1.4240831	0.357362111
NDUFA5;DKFZp781K1356											0.02269148	-0.9670595	0.01001446	-0.97406069	0.1467802	-0.197945277
NDUFA9											0.18892484	-0.2659454	0.84709232	0.70780055	0.5529236	0.62832578
NDUFAF4	+	+	+	+							3.91851085	6.53049469	4.38214531	7.46940676	4.3481534	4.788241069
NDUFB1											0.11542036	-0.4872386	0.00160701	-1.14278412	0.0994106	-0.623524984
NDUFB10											0.58995021	0.34099388	1.51414081	0.79837608	0.2140754	-0.116348902

NDUFB3											0.10323484	-0.4246642	0.2352965	-0.08326594	0.3246406	0.069530487
NDUFB6											0.28321436	-0.0365842	1.38225787	1.46974818	0.0910681	-0.495921453
NDUFB7	+	+	+	+				+	+	+	4.78062166	5.47031784	4.0105821	6.47827085	4.7361218	5.76009051
NDUFS1											0.79982213	0.71974881	1.07911589	1.17031034	0.3947065	0.300299327
NDUFS2											1.19786245	2.06418101	1.1779079	1.56927999	1.9664031	0.419473648
NDUFV1	+										0.86099519	0.68414434	1.13305347	1.04323133	0.8720552	0.624119441
NDUFV2											0.40479141	0.07419523	0.19511494	-0.11799939	0.1372489	-0.500102997
NEDD8;NEDD8-MDP1											0.05856244	-0.453783	0.0163235	-0.95504443	0.3343787	0.117794037
NEGR1											0.01703912	-1.6087284	0.79552441	1.08022436	0.0493301	-0.938154856
NELFB											0.20383695	-0.1862011	0.44115033	0.17716599	0.073756	-0.773293177
NELFCD;TH1L											0.57320596	0.20339076	0.77615272	0.51250394	2.3264931	1.06672287
NEU3											0.13460551	-0.2077096	0.09278387	-0.78889783	0.0274948	-0.498717626
NFU1											0.03471338	-0.9092986	0.01991691	-0.64414469	0.1535942	-0.444273631
NIPSNAP1											0.03683892	-0.9397799	0.17271852	-0.37442398	0.5881185	0.559396108
NIT2											0.03139281	-0.4582971	1.11006263	1.02187792	0.1704917	-0.457877477
NMD3											0.03384472	-0.7596029	0.50371314	0.43120893	0.1653374	-0.068073273
NME2;NME1-NME2;NME1											0.09965818	-0.115221	0.32939821	0.00414276	0.2340553	-0.010627747
NME3											0.10655946	-0.3841349	0.15345101	-0.45799955	0.0631334	-0.463242213
NMRAL1			+	+							0.15904381	-0.3497728	4.405027	2.73389498	2.5467788	3.009905497
NMT1;NMT2											2.71383949	1.55082766	0.16263564	-0.52616692	0.9772539	0.52565066
NNMT											0.97269553	0.29818535	2.22325012	1.34210205	0.836393	0.74345843
NNT											1.69077762	0.79040464	2.88630287	0.95696322	1.9124132	1.130865097
NOC2L											0.52694614	0.08022245	0.14899172	-0.2876784	0.0130154	-1.44115448
NOL3	+		+	+		+	+		+	+	1.68248246	0.86831093	6.12589132	7.60262871	5.3844344	7.702938716
NOP9	+										0.21007423	-0.1750221	0.33513406	0.05250549	0.0693611	-0.348116557
NOTCH1											0.12806068	-0.3221715	0.02950247	-0.15246201	0.0856628	-0.682677587
NPC1		+	+	+							2.59223976	4.52725156	3.06171174	5.97317759	3.8209518	4.727672577
NPDC1											0.05610524	-0.4636777	0.21237325	-0.17345556	0.0456505	-0.759724299
NPEPPS											0.11227902	-0.1324412	0.00228149	-0.42471822	0.263563	-0.008771896
NPHP3	+										0.8690108	0.5669117	0.30956787	0.01219749	1.021772	0.517929077
NPM1											1.40148486	0.61599731	0.52653485	0.04229482	0.8786927	1.264718374
NQO1											0.05562664	-0.6540407	0.6294823	0.86526171	0.3313042	0.124897639
NR3C1			+								1.53616201	1.45932579	2.72000337	2.69730886	0.0187544	-0.786497752
NRAS;KRAS		+	+	+							3.93487199	5.15334956	3.90362795	4.44223913	2.480567	5.002920787
NRM		+									1.92564695	2.52155304	3.18542205	1.57765261	0.390139	0.167125702
NRP1		+	+	+							3.51274797	4.58460617	1.96962123	2.22418149	4.1583669	4.285817464
NRSN2											0.17319316	-0.3129139	0.39872671	0.23551814	0.6659835	0.247281392
NSDHL											0.16900625	-0.2535057	0.74705433	0.36765862	0.9354523	1.232114792
NSMF	+										1.76834224	0.72189077	0.41577213	0.26078415	0.0246373	-0.53841273
NSUN2	+										0.26614655	-0.0165138	0.23265755	-0.03443909	0.02098	-0.279623667
NT5C3A											0.13085952	-0.3414574	0.23743824	-0.10294342	0.0772043	-1.029521942
NT5DC1											0.15032676	-0.1739305	1.02027982	0.87209956	0.3393503	0.068577449
NT5DC2		+									2.48961087	1.79471715	1.31943605	1.1914463	0.6514426	0.406042735
NUDC	+										0.36723975	0.09995842	0.5045627	0.2222449	0.034295	-1.063083013
NUDT21											0.87237229	0.5401535	0.07185495	-0.13145892	0.0006702	-0.853990555
NUDT5											0.11116811	-0.2312794	0.34851292	0.01538785	0.1155255	-0.153282801
NUDT8		+	+	+							3.41829565	4.68824768	3.4647693	2.67525991	3.17529	4.76339976
NUP155											0.09055426	-0.7071953	0.66031623	0.20716222	0.047698	-0.865578969
NUP93											1.31690089	1.21344439	3.32541692	1.22124227	0.0808277	-0.438690186
NUTF2	+										0.06263752	-0.3658282	0.22892006	-0.13611285	0.2883565	-0.031293233

OAT											1.40094974	0.38051478	0.7794171	1.07577515	0.9664212	0.244642893
OCIAD2											0.4716369	0.22247632	0.2404655	-0.19740041	0.0968736	-0.501720428
OGDH											0.2386637	-0.1224944	0.00534329	-0.81072299	0.129136	-0.652692795
OGFRL1	+	+	+					+	+		5.59906312	5.14160538	3.11676637	5.00341034	2.0218179	1.87865003
OLA1											1.1375678	1.10134697	0.73448677	0.39510028	1.5773454	0.789108912
OSBP											3.13955175	0.70845604	0.08313852	-0.56007957	0.1283936	-0.594381968
OSGEPL1											0.3712977	0.0953668	0.66461234	0.30270131	0.1315417	-0.534971237
OTUB1											0.7849943	0.23325793	0.0052731	-0.36517207	0.0387078	-0.506810506
OXA1L											0.01393509	-1.0651805	0.58667463	0.0881265	0.0122985	-1.0569369
OXCT1		+									3.27568725	2.86843872	1.68941497	0.3044529	0.0304809	-0.792228699
OXSR1			+								0.22632833	-0.1793327	4.15660593	2.78185908	0.7830968	0.629042943
P2RX4				+							1.03030155	0.70152346	0.13709495	-0.39065552	5.9045947	4.389010111
P2RX5	+		+						+		0.05581506	-0.5114498	4.78574378	4.28408813	0.001173	-0.70076561
P4HA1											0.01637741	-0.4735012	0.19028938	-0.10010846	0.5294711	0.338287354
P4HB											0.19020797	-0.0597197	1.61826341	0.17245611	0.0971545	-0.104322433
P4HTM											0.29249819	-0.0088488	0.08428903	-0.66114998	0.5952798	0.563522339
PA2G4											0.97400896	0.31027603	0.35598241	0.04800606	0.9518539	0.226144791
PABPC1;PABPC3											0.00126574	-0.5860322	0.01197255	-0.44027074	0.0037695	-0.227848053
PABPC4											0.04400015	-1.5091286	0.93580194	1.27202733	0.5945314	0.409472783
PACSIN3											0.00033388	-1.3910751	0.65039992	0.46097946	0.9053119	0.583602269
PAFAH1B1											1.48574078	0.72165108	0.21857453	-0.19165993	0.2788225	-0.063378652
PAFAH1B2											0.52840267	0.30247752	2.38793972	1.23059845	0.2661494	-0.083997091
PAFAH1B3											0.1135247	-0.5481339	0.03113819	-0.60097504	0.5667675	0.692836761
PAFAH2	+	+	+	+					+		2.94993546	3.33177058	7.93796278	4.21866798	4.4351449	3.78956159
PAICS											1.31551721	0.18496831	0.03577704	-0.2270031	0.0420524	-0.089912415
PAIP1											0.96001337	0.27030627	0.28387746	-0.00883993	0.1784071	-0.045085907
PAK2;PAK3											0.1373761	-0.4705321	0.31621587	0.06104914	0.2338562	-0.198141734
PALD1	+	+									4.05581212	5.16585795	1.31571115	1.75658417	0.2154012	-0.258788427
PALLD											0.11094787	-0.4233793	0.02412422	-0.77403831	0.0193879	-0.868971507
PALM		+	+								4.05473849	5.50061798	4.71731582	2.95223808	0.0746664	-0.363265355
PALM2;PALM2-AKAP2											0.0864156	-0.6793766	1.48507495	0.80561447	0.0047163	-1.916852315
PARK7											2.64059473	0.39015579	0.61501358	0.16799927	0.7408217	0.316599528
PARP1											0.00661117	-0.7771339	0.00776031	-1.06905683	0.0539431	-0.180533727
PC	+										0.00017388	-2.4672871	1.00E-06	-2.78267543	3.46E-05	-1.625013987
PCBP1											0.01984655	-0.1679624	0.03774732	-0.27776019	0.1808129	-0.088198344
PCBP2	+										0.55937537	0.11355464	2.94386905	0.24695969	1.652983	0.432343165
PCCA											0.00010137	-2.3034967	8.57E-06	-2.62257322	1.37E-05	-1.837729136
PCCB											0.00031022	-2.2430509	1.89E-06	-2.38427226	1.81E-05	-1.738293966
PCDH10		+									4.38861392	2.98644257	0.23426301	-0.11103503	0.1713732	-0.394654592
PCDH7											0.12416892	-0.1773885	0.11114342	-0.18655332	0.8734813	0.639116923
PCID2											0.31484642	0.02134641	1.34434561	1.63208898	0.1354313	-0.548453013
PCK2											1.01998067	0.73346837	0.0831759	-0.65433757	0.4886094	0.213245392
PCMT1		+									2.0327347	2.60250854	0.2020839	-0.12290764	1.0732895	1.508822759
PCMTD1	+	+	+	+	+	+	+	+	+	+	5.23053272	3.96355247	5.74177666	4.96446228	3.3248435	5.431502024
PCMTD2	+	+	+	+	+	+	+	+	+	+	5.71720482	7.38635635	4.76533273	4.32198016	5.5535409	5.948863347
PCNA											2.0641799	0.36451149	0.19551585	-0.05004628	2.1537343	0.363164902
PCYOX1	+										0.46184369	0.33315277	0.47806278	0.20004781	0.045003	-0.994407654
PDCD4											0.67210169	1.03950119	0.04297382	-0.50897344	0.2586532	-0.131708145
PDCD6			+								1.99337172	1.54067357	3.79185687	2.25145658	0.0188746	-1.206738154
PDCD6IP											0.07231361	-0.1857821	0.10232395	-0.08742205	0.0518057	-0.165981929

PDE12											0.5977039	0.28260231	0.00256498	-1.19941966	0.1289509	-0.456802368
PDE8A	+		+	+					+		2.10056534	1.66713905	1.83069971	2.69749832	3.2987605	2.162345886
PDHA1											0.84704223	0.26443736	0.06977798	-0.1775945	0.2773011	-0.07323583
PDHB				+							1.22691674	1.52143606	3.42925359	0.58688736	3.7472763	1.999279022
PDIA3											1.2941172	0.23690605	0.27075616	-0.00871277	0.0062238	-0.216061274
PDIA3											6.26E-06	-5.4528033	2.63E-05	-5.92588679	5.98E-05	-4.204325358
PDIA4											0.91499121	0.29853503	0.00206302	-0.2980957	0.0876639	-0.103401184
PDIA6											1.0597646	0.20240529	0.35937149	0.02003288	0.5974933	0.103507996
PDK3											0.18502848	-0.1350269	0.56685383	0.2929891	0.2146395	-0.112163544
PDLIM1											0.92581127	0.53977203	0.23876603	-0.0493482	0.137719	-0.547485352
PDLIM5											1.44128074	1.07459831	0.07101708	-0.48212369	0.6115411	0.761518478
PDLIM7											0.77482414	0.72320048	1.64844715	1.31637192	0.2136219	-0.282188416
PDS5A											0.20933115	-0.111908	0.16815105	-0.15817642	0.3968345	0.089179357
PDXDC1	+										0.20736106	-0.1649017	0.44783937	0.1218001	0.9532656	0.146243413
PDXK	+										0.20893129	-0.2135633	2.42126599	0.84463247	0.0747539	-0.114547729
PDYN											0.52323573	0.39646975	0.01398504	-0.78288269	0.0196329	-0.587080638
PEBP1			+	+							0.60728578	0.23946571	4.99025734	5.09333356	1.1570476	3.169841131
PEMT											0.12868367	-0.14372	0.14858055	-0.52206294	0.4835442	0.365038554
PERP											0.05042257	-0.7944291	0.00133216	-0.71335983	0.7884555	1.974311193
PEX11B	+										0.12661085	-0.3256785	0.04496463	-0.65428225	1.5128672	1.422446569
PFAS											0.03424154	-0.1412837	0.00298849	-0.6774133	0.0123541	-0.579341888
PFDN2											1.03453317	0.38241005	1.71315714	0.81452052	0.2804317	-0.049551646
PFDN5			+								1.59586111	0.82311948	2.93877413	1.82490603	0.5340328	0.075183233
PFKL											0.69414152	0.28801282	0.37825365	0.18689791	1.4652471	0.314800898
PFKM											1.34156907	1.73569679	1.15103242	0.34164619	0.6117904	0.659406026
PFKP	+										1.22789317	0.36121941	0.82428954	0.1193161	3.058884	0.347803116
PFN1	+										1.72096067	0.50755755	2.7148342	0.31835365	0.9663929	0.145768483
PGAM1											2.48182979	0.57131004	2.16637545	0.55760129	1.6248823	0.288558324
PGD	+										1.72616446	0.1834596	0.70899321	0.04508146	4.088012	0.322681427
PGK1											2.60025011	0.67059453	1.56307543	0.29174487	2.0373074	0.272743861
PGLS			+								2.97733675	1.14304034	3.71843267	3.70939445	1.1512782	1.295130412
PGM1											0.64651134	0.21568425	0.31708781	0.00294495	0.2103257	-0.204731623
PGM2											2.49531839	0.45988592	0.68097506	0.08488019	1.1860118	0.479082743
PGP											0.89799218	1.16121101	0.51368378	0.52025731	0.698192	0.602221171
PGRMC2	+										1.85155644	0.88951429	2.33745153	0.82421239	1.8447216	1.243110021
PHACTR2	+	+	+		+			+			2.32551853	1.9031264	2.73876965	3.53144455	0.9090023	1.590363185
PHACTR4	+										0.1316203	-0.3950272	0.20942131	-0.30070051	0.1105644	-0.440009435
PHB											1.47760463	0.29500771	1.8885028	0.72237968	4.4291227	0.703247706
PHB2											0.77356623	0.71251488	1.23086127	1.85643196	1.00155	0.717606862
PHGDH											5.15268145	0.72952525	3.18932095	0.65563075	1.8882723	0.308031082
PI4K2A		+	+	+							2.65137016	6.16177177	1.67563155	3.79728444	1.6959119	4.414013545
PI4K2B		+	+	+							5.40536708	5.64176178	4.0632663	4.22329458	3.8556241	4.842420578
PIGU		+	+								2.71285316	1.94585292	3.23173356	2.69036802	2.6769583	1.775272369
PIK3R4	+	+	+	+							5.30045627	5.32244364	5.54419643	7.50146675	3.8504845	4.652011871
PIR	+										0.30609912	0.00952403	1.01515113	0.90261587	0.4357398	0.413263957
PITPNB	+										0.09676043	-0.3354314	0.53416383	0.38294856	0.4503608	0.324713389
PKM;PKM2											0.33015714	0.01096217	0.0015338	-0.25788752	0.0461852	-0.126078924
PKP3											0.03285717	-0.9257291	0.45013562	0.17090162	0.6319714	0.392136892
PLA2G4A											0.32314831	0.02440008	0.00309154	-0.35637792	0.2028982	-0.122101466
PLAUR	+										0.32018704	0.03507106	1.75782665	1.57134755	0.2731818	-0.037590027

PLD1											0.50024075	0.19516754	0.32321225	0.02390607	0.0293705	-1.26292038
PLD3											0.00753632	-1.266229	1.45711268	0.68274689	0.3839591	0.115381241
PLEKHN1	+										0.62596844	0.3598779	0.03002155	-1.13869985	1.0136968	1.663065592
PLGRKT	+	+	+	+				+	+	+	3.14258371	5.33557447	4.79826679	4.21012942	4.7407115	3.528661092
PLIN3		+	+								2.28378641	3.39477475	2.1778134	4.19363403	3.5135265	1.391642888
PLOD1											0.15896858	-0.1905886	0.06755719	-0.3352108	0.0978896	-0.620832443
PLOD2	+										1.07847517	0.72864087	0.01399414	-0.54868126	0.441366	0.431589762
PLP2		+	+	+							1.28446565	3.07412148	3.08380848	5.34487152	4.9691259	5.117419561
PLS3											0.23638229	-0.0192388	0.68864681	0.07597478	0.2171747	-0.025889079
PLSCR1		+	+	+							4.80224984	6.38903427	4.92506235	5.87978236	4.8664935	5.69774437
PLSCR3		+	+	+							3.54689787	3.81843185	4.75073861	4.06563695	6.5388635	4.753132502
PLSCR4											0.02600261	-0.4454422	0.12651581	-0.3444322	0.1058531	-0.549160004
PLXNB2		+	+								3.80612754	1.9422849	4.21118781	3.3228728	2.5555894	1.580003738
PM20D2											1.20342894	0.579518	2.27613782	0.72679456	0.0159988	-0.832993825
PMM2											0.19223217	-0.1666711	0.24305588	-0.13552729	2.3214823	0.621834437
PMPCA											1.41413758	1.9576486	0.19570249	-0.31359927	0.4590901	0.445972443
PMPCB											2.63678769	1.39093717	0.91129636	0.32850456	0.0536434	-0.588537852
PMVK											0.10849776	-0.4513353	0.9187695	0.4255263	0.5552005	0.324727376
PNP											2.90938578	1.13865026	3.6232479	1.23006884	3.2380824	0.842449188
PNPT1											0.28918994	-0.0159467	0.61726664	0.30592219	0.152436	-0.270849228
PODXL			+	+							0.62837824	0.6780599	3.80190015	4.68178876	3.2010134	3.269297282
POLD1											1.99837259	0.76600711	0.05588814	-0.6303819	0.6015156	0.199630737
POLDIP2											0.06363673	-0.611721	0.06814962	-0.75457891	0.0243088	-0.429935455
POM121;POM121C			+								1.35905724	0.89937592	2.55175581	2.68496768	0.0833902	-0.728123347
POM121C											0.19945825	-0.1981564	0.4334669	0.25530688	0.2919358	-0.025206884
POMGNT1		+									2.60583988	1.75327682	0.279279	-0.04000791	0.0491041	-1.009273529
PON2	+										0.12084114	-0.3666337	0.8079651	0.59986178	0.0915698	-0.792678197
POR	+										0.06012492	-0.431743	0.18678115	-0.38292567	0.1361955	-0.272220612
PPA1											0.07813068	-0.2305298	0.00152784	-0.32441966	0.0410369	-0.261493683
PPA2											0.92970544	0.33258883	2.15538162	0.69919904	0.0215541	-0.176997503
PPIA											0.96056138	0.38098653	0.83374425	0.09114265	0.9183656	0.097608566
PPIB											0.80365832	0.44535764	0.98880218	0.27383804	0.1571572	-0.105995814
PPID											0.0079966	-0.9361871	0.061092	-0.62070465	0.0237787	-0.815547943
PPIH											0.94174742	0.76661046	0.42319811	0.18430901	0.1152882	-0.727513631
PPM1A	+	+	+	+	+	+	+	+	+	+	4.48539476	8.03221766	5.89775819	8.50407028	6.8285641	8.638414383
PPM1B	+	+	+	+	+	+	+	+	+	+	6.20293546	7.78252602	3.86839259	7.14182854	5.161541	7.04610316
PPM1G	+	+	+	+	+	+	+	+	+	+	5.4011526	9.50939178	4.53135844	10.4265404	4.6340143	10.21605428
PPME1											1.2065096	0.39126078	2.60263395	0.51883125	0.6366899	0.508453369
PPP1CA;PPP1CC;PPP1CB											0.60097314	0.07347616	0.66434615	0.2929643	0.8558103	0.211104711
PPP2CB;PPP2CA			+								1.07623946	1.50794347	2.51722861	2.33151881	0.39604	0.307301839
PPP2R1A											1.47743708	0.4292717	2.87021541	0.48911858	2.7131502	0.786116918
PPP2R4	+										0.80532391	1.21148809	1.35307194	1.84073257	2.014487	0.388498942
PPP3CA		+	+								2.66205978	2.78497823	5.48208567	1.98709106	3.0478234	1.305123011
PPP3R1	+	+	+	+	+	+	+	+	+	+	4.30485399	7.85159365	3.24851148	7.0073789	3.4817723	6.81257693
PPP5C											1.41460373	1.56782023	0.76575083	0.65658379	0.3326568	0.04838562
PPT1											0.44020665	0.2450072	1.32431879	1.05623436	0.0271202	-0.54360199
PQLC3											0.12579183	-0.3459428	0.09303023	-0.42358398	0.3074054	0.007899602
PRAF2;WDR45		+	+	+							4.52434073	4.95693525	3.66381578	4.71139717	4.8181835	2.637910843
PRDX1											0.27205911	-0.020263	0.00180227	-0.31177775	0.0069063	-0.223774592
PRDX2											1.08576347	0.5941747	2.05134234	1.847826	1.1796069	0.317325592

PRDX3											2.75180075	0.77534294	3.25357671	0.74529266	0.5957234	0.106656392
PRDX4											0.00213397	-0.5504799	0.141571	-0.13221232	1.1802278	1.796015422
PRDX5	+										2.27929828	1.20436605	3.55220186	1.42554855	2.6188899	0.66752243
PRDX6											2.80770274	0.7717495	3.12561272	0.77024142	2.1350988	0.488433202
PREX1	+										0.6144828	0.25749906	0.31320676	0.02906164	0.2411055	-0.052832921
PRIM2											0.15455124	-0.3201682	0.03739327	-0.61801974	0.1584603	-0.293903351
PRKAA1		+	+	+							3.8076853	4.83915393	3.68485744	3.60692406	3.1039856	3.912742615
PRKAB1	+	+	+	+				+			5.83715338	6.81500816	4.10191121	6.48538144	5.1158984	7.559259415
PRKAB2	+	+	+	+							4.23914257	4.46021907	3.7872874	4.76321983	4.6779126	5.016141891
PRKACA	+	+	+	+	+	+	+	+	+	+	5.64206866	9.06105042	4.68039788	9.73997498	4.4741159	8.212789536
PRKACB	+	+	+	+	+	+		+	+	+	4.93138816	6.09754372	4.64676288	7.41508738	4.1565923	5.960465113
PRKACG	+										0.12540124	-0.3577118	0.03497035	-0.40040334	0.568079	1.330233892
PRKAG1											0.38950723	0.15899277	0.20839573	-0.13675372	0.2364122	-0.11856397
PRKAR1A											0.01683465	-0.6146615	0.72801588	0.77331734	0.0364374	-0.418566386
PRKAR2A		+	+								3.21582026	1.80595779	3.50659296	3.10236804	0.3497144	0.152823766
PRKDC	+										0.01740614	-0.1613413	0.0053796	-0.29045804	1.1741299	0.172273
PRMT1	+										0.01560061	-0.3101215	0.00523884	-0.5075175	0.001233	-0.649600983
PRMT5											0.13396273	-0.8055286	0.41667063	0.12046878	0.2170994	-0.172585169
PRNP			+	+							0.19330938	-0.2055893	4.30338041	5.04839643	4.7185601	4.0564092
PROCR		+	+								4.06335264	3.93729591	4.02168508	5.06844012	0.0232318	-1.063133876
PRPF19											1.08104515	0.13648097	0.0013676	-0.48451742	0.7284581	0.144421895
PRPF38B											0.75679213	0.54031626	0.14764319	-0.49377314	0.0070711	-0.996080399
PRPF4B											0.00448196	-1.2787177	0.15800951	-0.42718379	1.5415449	0.767047246
PRPH											0.86922092	0.76601982	1.28326428	0.60383542	0.8209851	0.680135727
PRPS2;PRPS1											0.00709971	-0.683574	0.37314696	0.20822144	0.0037877	-0.576324463
PRPSAP1	+										0.06560564	-0.9053605	0.24936123	-0.08162562	0.2057772	-0.204618454
PRSS21	+		+								0.11447544	-0.3967476	5.28463338	5.03153865	0.0459247	-0.60014979
PSAT1											2.38652336	0.68474897	0.06064704	-0.25121307	0.0572232	-0.387372335
PSEN1											0.70717506	0.51025518	0.00047082	-1.70366287	0.6463321	0.731079102
PSMA1											0.00028783	-1.6354656	0.05385748	-0.37246895	0.0393101	-0.248969396
PSMA2											0.24873929	-0.1752205	0.16564185	-0.56566238	0.9418549	1.030496597
PSMA3											0.47049496	0.04899979	0.30546768	0.00157611	0.0898831	-0.107526143
PSMA4											1.86472954	0.89905993	1.60224333	0.38028399	0.9106604	0.360899607
PSMA5											0.03477964	-0.285511	0.12556647	-0.12513479	0.0352734	-0.280023575
PSMA6											2.02824344	0.2726682	2.57478502	0.53691673	1.2107086	0.202018738
PSMA7											1.57719223	0.26471901	1.52333924	0.11746661	0.4622239	0.056159973
PSMB1											0.19561664	-0.2005177	0.32905008	0.00963529	0.6044113	0.205191294
PSMB2											1.18107606	0.37746557	0.56505427	0.15703138	0.6287085	0.909877141
PSMB3											0.26463281	-0.0954367	0.58909489	0.66987546	0.2449104	-0.06663386
PSMB4		+									3.62747087	1.64428202	1.03354887	1.03865178	1.0151943	1.410060247
PSMB5											0.66282627	0.62946892	0.11517286	-0.10942777	0.7241089	0.100240707
PSMB6											0.16910794	-0.2079983	1.23822384	0.85145187	0.4226879	0.284105937
PSMC1	+	+	+	+	+	+	+	+	+	+	3.55118532	8.88243675	7.81230643	11.1749064	5.5115093	9.715284348
PSMC2		+	+	+							3.64407733	4.65997251	3.17856148	4.67845027	4.1617813	4.353047053
PSMC3											3.63247279	0.88292567	3.30802399	1.46244558	1.1494931	0.409464518
PSMC4											0.93715999	0.78001849	1.54860072	1.72300657	1.6597168	0.558958689
PSMC5		+	+	+							1.88362022	4.31811015	2.39393587	6.724425	1.9597364	5.44271787
PSMC6											4.40298072	0.90317218	4.20684049	1.16930072	4.2736489	0.477105459
PSMD1											0.34822366	0.01736768	1.7338634	0.18438021	0.8146603	0.130264282
PSMD11											0.8406274	1.26348877	3.36664777	0.63719495	0.2960148	-0.001314163

PSMD12											0.49248122	0.07337189	1.10114988	0.29125341	1.310778	0.315052668
PSMD13											2.36961201	0.58503914	1.98728613	0.46421814	0.4333938	0.082146327
PSMD14											2.17680312	0.38448079	1.46303914	1.72249158	1.0669051	0.904056549
PSMD2											1.69379839	0.63586553	3.66166373	1.58712959	3.393903	1.340033849
PSMD3											3.34702828	0.68970426	1.18771555	0.39316813	1.526992	0.464485804
PSMD4											0.6990202	0.42272313	0.95997752	0.75778453	0.6213471	0.349686305
PSMD5											0.28478484	-0.0279783	2.22484415	0.94565519	1.4224008	0.21526591
PSMD6											1.20490653	0.49780464	1.68009881	0.73275693	1.4553317	0.295530319
PSMD7											0.1440264	-0.3257548	1.20773853	0.74145253	1.3718478	0.578713099
PSMD8											2.28574409	0.71892103	0.88225322	0.29246902	0.1633576	-0.062466939
PSME1			+								1.73175685	1.23377673	1.77552947	2.58602142	1.9762646	0.958571116
PSME2		+									2.84053381	2.44889005	2.4185648	1.74948692	2.859569	0.997554143
PSME3			+								2.23576056	1.56105359	2.71441611	3.07555262	0.0432007	-0.964749654
PSMF1											0.20753009	-0.1515554	0.09964972	-0.28569539	0.0741707	-0.634969711
PSMG1											0.37968652	0.17890803	0.46400036	0.49827703	1.1347406	0.756296794
PSMG2											1.59361261	0.98624357	0.01928393	-0.62149556	0.4143029	0.240056356
PTBP1											0.01934299	-0.3670959	0.01729776	-0.2854379	0.0994279	-0.124950409
PTDSS1											0.05819484	-0.6713339	0.61428906	0.62432988	0.5628208	0.274766922
PTDSS2		+									3.80868371	1.63416608	0.11526627	-0.48627218	0.2000854	-0.27282842
PTGES											0.28079606	-0.026439	0.4455497	0.29084587	0.1149483	-0.309021632
PTGES3											0.52403503	0.09651566	0.38814601	0.04277611	0.5467184	0.066631953
PTGFRN	+	+	+	+							4.99937586	8.12168248	2.45853427	6.00249036	4.7800294	8.342305501
PTGS2;COX-2											0.00429728	-0.4800638	0.38476097	0.18636386	0.0977596	-0.497881571
PTK7	+	+	+	+							5.27039902	5.26184209	4.62589116	4.9186554	4.2482152	4.805984497
PTPLAD1		+	+	+							3.50909744	2.26554171	4.53849721	2.58814112	1.6710754	2.922632217
PTPLB			+	+							0.19947494	-0.3348694	1.99780863	2.07139015	4.7917547	3.120976766
PTPN1		+		+							1.55441196	2.07817332	1.26684899	2.0124925	2.7566408	2.872603099
PTRH2		+	+	+							3.74080861	3.69624647	3.60576017	4.39416377	2.0325074	3.454886754
PTRHD1											0.03376176	-0.4313456	0.17926121	-0.23680878	0.4313994	0.279179255
PTTG1IP	+	+	+	+							3.39207176	4.55287743	2.8344553	3.52306747	3.7610043	4.871233622
PUF60											0.5655781	0.67847125	1.18805505	1.57478142	0.7193512	0.862325033
PUS1	+										0.28509743	-0.0314522	0.8086712	0.51076953	0.8929478	0.706682205
PVR		+	+								2.92428168	2.34546407	3.87967911	4.26790174	1.5507836	2.149511337
PYCR1											1.08036794	1.04511134	3.94993405	0.87023481	2.6806165	0.338493347
PYCR2											3.97458799	0.96609688	1.69733019	1.11581802	1.2156009	1.54762586
PYCRL											0.88485726	0.66803296	1.06032372	0.9758091	0.0202486	-0.510115306
PYGB											0.48300606	0.12824694	0.04243264	-0.96202787	0.7202913	0.856864929
PYGL				+							0.16404568	-0.081529	0.17790936	-0.05654907	3.7490909	2.46363767
QARS											1.09862119	0.20681636	0.01059453	-0.46367137	0.4124182	0.068609873
QDPR											0.14251305	-0.3834565	0.18147432	-0.27449481	0.2557634	-0.047123591
QKI		+	+								2.30949131	3.72485224	3.27004438	1.76289431	1.6760455	1.782091777
RAB10											0.01023142	-0.4514586	0.94003985	2.05267906	0.6013384	0.891322454
RAB14											0.53345558	0.43314679	0.75660251	0.71918233	0.04437	-0.568862915
RAB1B;RAB1C			+								0.75478083	1.59673754	1.29534869	2.84510867	1.6855425	1.491238912
RAB21											0.02039878	-1.5121129	0.79815776	0.49426651	2.6997872	1.461900075
RAB31;RAB22A											0.08395026	-0.4616299	0.79140167	0.83959961	0.4310911	0.229663849
RAB35											0.65434885	0.32967186	1.47661102	0.84886678	1.5398555	1.245645523
RAB5B											0.05831621	-0.9704018	0.02907108	-1.51889801	0.016381	-1.867886225
RAB5C;RAB5B;RAB5A											0.67079481	0.58630943	0.74910291	0.71217155	1.4383722	1.826674143
RAB6B;RAB6A;RAB39A											1.79915623	0.94928106	0.07954093	-0.54137929	0.7402601	1.567661285

RAB7A											1.59028137	0.72160594	3.20026671	0.78048452	1.7495472	0.482369105
RAC1;RAC3;RAC2											2.58322034	1.09457207	2.66214998	0.80035082	1.5133557	0.232357661
RAD23A											0.46561998	0.10942586	0.33044918	0.05440521	0.0163115	-0.613276164
RAN											1.5288143	0.54290263	2.45747889	0.64781125	1.5789883	0.387294769
RANBP1	+										0.96158202	1.81808599	1.33202258	2.35290337	0.9194838	1.569248835
RANBP3											0.19886755	-0.2181104	1.93762575	1.10646121	0.0250333	-1.238896688
RANBP6											0.90499827	0.57290077	0.01826382	-0.75211652	0.0006656	-1.163546244
RANGAP1											2.58145933	0.84756152	3.28578756	0.59337552	0.1729602	-0.174105326
RAP1B;RAP1A											0.77960059	0.2428112	1.56427078	0.3093001	1.5266496	0.479056676
RAP1GDS1											2.93543565	1.15611966	2.43048133	1.77285258	0.6374706	0.66841952
RAP2A		+	+								1.93077239	2.58724403	4.13744063	2.05133947	0.4529722	0.181064606
RAP2B		+	+	+							3.29180618	5.0179704	5.60002181	4.06819153	5.0756622	4.90997378
RAP2C		+	+	+							4.00516682	6.14101601	4.64176046	5.52757136	3.5714433	5.060958227
RARS											1.58784831	0.41813787	0.43247223	0.04964701	0.5935168	0.094642003
RBBP7;RBBP4											0.59079782	0.83151118	0.11005592	-0.24113655	0.0286412	-0.941814423
RBM39											0.01474773	-0.4010175	0.00059979	-0.97551282	0.0085786	-0.460009893
RCC1											0.0927221	-0.3192476	0.56386885	0.22963715	0.1516696	-0.283174515
RCC2											0.0076426	-0.7080822	0.06972369	-0.18834496	0.0073179	-0.690225601
RCE1											1.81124773	1.71196938	1.52291713	0.72494189	2.1177868	1.655808131
RDH11											0.7057024	0.24539757	0.94903511	0.89392726	0.0339658	-0.189612071
RDX											0.09680909	-0.2469355	0.02468785	-0.99720637	0.0112854	-1.12335523
RECQL											0.31974057	0.03179868	0.75306382	0.94542758	0.3633058	0.100442886
REEP5											0.0773272	-0.5807654	0.0836925	-0.2203668	0.0204784	-1.775858561
RELL1											0.05908516	-0.7797298	0.11107167	-0.32693164	0.4260302	0.131006877
REPIN1	+										0.05977744	-0.7460244	0.11699328	-0.77676264	0.4240896	0.254271189
RER1		+	+	+							3.64645962	2.35624949	2.40225096	3.14954885	3.4214402	2.242008209
RFC2											4.73592563	0.40687625	0.88054695	0.26113065	0.0224462	-1.053431829
RFC3											0.51752951	0.45931625	1.15702048	0.79231453	0.4237806	0.17354393
RFT1	+										0.24202837	-0.1324457	0.53937779	0.44687208	0.2627201	-0.091320674
RFTN1	+	+	+	+	+	+	+	+	+	+	3.15939318	5.70600637	3.813388	6.59962273	5.7014165	5.558599472
RFX1		+	+								3.01795083	2.95284653	2.87402546	2.50693703	0.0298452	-0.830662409
RGS17											0.45409501	0.20461718	0.24591839	-0.07285817	1.2022356	0.687699
RGS19		+	+	+							3.16987231	5.09794426	3.38690912	4.12015661	2.8628513	4.379569372
RHBDD2		+	+	+							5.69116095	4.31684812	3.54616913	4.01725642	3.2525844	3.728707631
RHOA;RHOC			+								1.90941677	1.78636805	4.82023021	2.46588453	0.5673148	0.787497203
RHOB		+	+	+							3.9458841	5.2064635	4.34225959	6.16626612	4.4638502	3.344495773
RHOG		+		+							1.93641715	3.3085645	0.63022912	1.86015129	1.3555506	2.547997793
RHOQ											1.45089124	0.91153844	0.21531086	-0.1333402	0.8847748	0.795535405
RHOT2											0.5399566	0.18712807	0.14481565	-0.28222974	0.648613	0.579053243
RNF11	+	+	+	+							3.9576693	3.47097969	1.64681829	2.59180133	1.7934167	2.337239583
RNF141	+	+	+	+	+	+	+	+	+	+	5.72259473	6.67756844	3.77891512	4.92775599	3.0547402	3.745675405
RNF167			+								0.33254226	0.043986	2.93776622	2.68403562	0.7879279	0.7119929
RNF223				+							0.30209532	0.0017217	0.73565505	0.98102951	2.7917098	2.01110967
RNF34											0.0055407	-0.7986234	0.28195066	-0.02434603	0.9775001	0.919017792
RNH1											1.16355423	0.32753118	0.00502242	-0.5032889	0.0181573	-0.314580917
RNPEP											0.03561703	-0.3061028	0.47437869	0.14270655	0.0260848	-0.307029724
ROR2											0.62746327	0.4324131	0.83952995	1.06793086	0.0461488	-0.475979487
RP2	+	+	+	+	+			+	+		5.13261744	7.19534874	5.63763417	6.57977931	3.8674947	3.366534551
RPA1											0.28617504	-0.0529963	0.66587413	0.34516589	0.3698397	0.135730108
RPA3											1.63026735	1.06620916	0.25286818	-0.11888949	0.8758171	0.607540766

RPL10	+										0.45598025	0.07466571	0.04294035	-0.22778002	0.7769428	1.384840647
RPL10A											0.26904397	-0.0180531	1.47297322	0.62667084	0.198492	-0.051422755
RPL11											1.63495449	0.77198219	0.45502146	0.0702006	0.1980134	-0.03107961
RPL12											0.54710942	0.11574872	0.905461	0.15198644	0.1950535	-0.044643402
RPL13											0.00054955	-1.0228246	0.00020395	-1.52586683	0.0002941	-1.476921082
RPL13A											0.0040234	-0.7804674	0.0002422	-1.50447591	0.0001433	-1.496429443
RPL14											0.27744667	-0.0168101	0.01834219	-0.30518977	0.00049	-0.48470815
RPL15	+										0.00851987	-0.9151096	0.0003101	-1.26632055	0.0167218	-0.715289434
RPL17											0.3122305	0.01101875	0.014422	-0.41974767	0.0042885	-0.355304718
RPL18	+										0.00043399	-1.0942523	0.00105531	-1.465765	0.0001012	-1.309942881
RPL18A											0.40317794	0.09634145	5.25E-05	-0.93391927	0.0060118	-0.711090088
RPL19	+										0.02913551	-0.3815797	0.00571393	-0.83183352	0.0081941	-0.828573227
RPL21											0.01157662	-2.3383115	0.52008513	0.15534274	0.5107548	0.67199262
RPL22											2.46407373	1.17352168	4.79699449	0.83154869	2.5565171	0.773678462
RPL23											0.67324846	0.13652547	0.51088116	0.10065079	0.8729484	0.096230189
RPL23A											0.2669749	-0.0602303	0.00029729	-0.56926155	0.0049804	-0.321785609
RPL24											0.12342551	-0.1506837	0.00102646	-0.63045692	0.0029463	-0.592549006
RPL26;KRBA2;RPL26L1											0.61442923	0.66194026	0.61082184	0.39653842	0.0420062	-1.606728872
RPL27	+	+									2.96999761	2.15447362	1.11134143	0.95317014	2.8702948	1.460292816
RPL27A											0.05436395	-0.3060277	0.00030515	-0.64736112	0.0006897	-0.649194717
RPL3											0.11507104	-0.2318745	0.00010455	-0.57735634	0.0011455	-0.560805639
RPL30											1.10783935	2.06214333	1.01664657	1.7537384	0.8842655	0.799543381
RPL32											0.00039489	-1.6653697	0.001467	-1.3135732	0.0023844	-1.303119024
RPL34											0.45044915	0.18313026	0.13615468	-0.24643453	0.0618779	-0.305300395
RPL35											0.07021938	-2.404026	0.02758074	-1.08602269	0.0049892	-1.479029338
RPL36											1.16E-05	-1.1093922	0.00178547	-1.05971654	0.0001676	-1.029904683
RPL37A											0.04238871	-0.4049778	0.06402956	-0.26287333	0.0256871	-0.676525116
RPL38		+									1.78378843	3.44387118	2.81233249	0.99481201	2.4292902	0.99971199
RPL39P5;RPL39											0.01656244	-3.0993563	0.30534242	0.00965627	0.0826341	-0.689846039
RPL4											0.59487486	0.12666257	0.00034357	-0.48989423	2.27E-06	-0.370765686
RPL5	+										0.00254082	-0.4984665	0.00262592	-1.01101748	0.0040819	-0.74405098
RPL6											0.00958812	-1.0914307	0.0001041	-1.45066961	8.19E-05	-1.35528628
RPL7											0.21233882	-0.0452964	0.00055207	-0.62805049	0.0042694	-0.624293009
RPL7A											0.46302751	0.18430964	0.41896566	0.07607396	0.1352391	-0.095369975
RPL8	+										0.0548466	-0.5006618	0.00270923	-1.57008934	0.0723342	-1.304972331
RPL9											1.00908009	0.23478826	2.11268201	0.45673688	0.5767195	0.184434891
RPLP0;RPLP0P6											3.96570703	0.85754585	3.00356274	0.51895905	2.1058304	0.478434245
RPLP1		+	+								1.92978798	2.31647873	4.27793504	2.67675591	0.0253095	-0.63217481
RPLP2											0.14881384	-0.2058385	0.20723308	-0.22413254	0.0717604	-0.981230418
RPN1											0.28848216	-0.0062733	0.25570207	-0.01711337	0.2397261	-0.025671641
RPN2											2.3633271	1.60771688	0.22801947	-0.24151103	1.6436386	1.128644943
RPRD1B											0.26550504	-0.0362523	0.2601979	-0.06676801	0.0357415	-0.91217804
RPS10											0.11514182	-0.3097935	0.02187918	-0.50297991	0.0455483	-1.306358337
RPS11											0.14717717	-0.1238073	0.03507052	-0.14744568	0.4951067	0.045389175
RPS12											1.43751879	0.60516866	3.11191	0.73221334	1.0670947	0.242195765
RPS13	+										0.41853894	0.0458018	0.0687715	-0.12774722	0.0089712	-0.619223277
RPS14											0.85231901	1.30712255	0.01906733	-0.21607399	0.3127746	0.007832845
RPS15A											2.67749472	0.384758	2.58691542	0.98192533	1.3625561	0.524208069
RPS15A											0.00030691	-4.2147986	1.78E-05	-3.98962021	0.0153591	-2.786168416
RPS16											0.64494779	0.20954704	0.97552692	0.11947441	0.2636446	-0.014559428

RPS17L;RPS17	+										0.28118524	-0.014335	0.9258391	0.45727921	0.1462382	-0.086081187
RPS18											0.02702779	-0.2777532	0.00376758	-0.59242058	0.5206689	0.437360128
RPS19											0.59100852	0.56931432	0.69873618	1.01070595	0.0512897	-0.346597036
RPS2											1.20580764	0.31556384	0.00421126	-0.33710353	0.1564181	-0.077707926
RPS20											0.21217366	-0.4403909	0.03406796	-1.46297264	0.06811	-1.207049688
RPS23	+										0.51044039	0.13204002	0.00083912	-0.54379336	0.0598357	-0.19348526
RPS24											0.08695131	-0.1296431	0.02878425	-0.83840879	0.0365826	-0.35697492
RPS25											0.33432114	0.0547816	2.08344171	0.43995349	0.4862487	0.077107747
RPS26;RPS26P11											0.03276202	-1.3669758	0.23773545	-0.22777621	0.0026404	-3.097171148
RPS27A;UBB;UBC;UBA52;UBBP4			+	+							1.82951488	1.72601636	4.5965066	3.75433922	4.0899144	2.681430817
RPS27L;RPS27											1.90128301	1.0981547	0.00089808	-1.99868329	0.0815611	-0.814568202
RPS28											0.15738572	-0.2508767	0.07421762	-0.83942731	0.0253263	-1.491978963
RPS3											2.46427905	0.50236956	2.10761417	0.3723437	1.0371366	0.23852857
RPS3A											2.26830308	0.67257055	0.83930284	0.13347816	0.5487869	0.072378794
RPS4X											1.01117006	0.23699188	1.95354936	0.31962331	0.9348238	0.17838796
RPS5											2.32467855	0.95947393	2.16785373	0.74114672	2.4244984	0.333944956
RPS6											0.035936	-0.5174363	0.00039215	-0.93986448	0.0028691	-0.66901652
RPS6KB1											0.04509754	-0.9340801	0.00463288	-1.02546183	0.1549908	-0.245318731
RPS7											0.92872455	1.35563596	0.31295129	0.01227442	0.0888836	-0.767843246
RPS8	+										0.07379569	-0.242211	0.00014095	-0.97211266	0.0033381	-0.55876414
RPS9											0.02056682	-0.3589293	0.00030695	-1.16790136	0.0005786	-1.308356603
RPSA;RPSAP58											1.13826379	0.31024806	0.36715154	0.05172984	0.0149251	-0.386887232
RQCD1											0.83208014	0.43748665	0.36571538	0.14592616	0.1439075	-0.185736338
RRAS		+	+	+							3.69831672	3.32968267	3.41371862	3.97017543	2.963222	3.059521993
RRAS2		+	+	+							4.19592447	5.99429639	4.18921535	4.34729131	2.6375613	3.483658473
RRM1											0.03392345	-0.3369204	0.00378058	-0.37767855	0.0182106	-0.350418727
RRM2	+										0.00348529	-1.3239168	0.72877612	1.1768411	1.2481695	1.496761958
RRP12	+										0.24573706	-0.0803547	0.12186458	-0.4261659	0.0144416	-0.935934067
RSPH10B2;RSPH10B											1.75061776	1.05552038	0.01532915	-0.61386998	0.4729989	0.93210729
RSPRY1		+	+	+							4.1950629	4.0650959	3.66324495	3.97590383	3.0518066	3.610448837
RSRC1	+										0.13282889	-0.4221579	0.00760684	-0.88544846	0.3027868	0.003503164
RTN3		+	+	+							1.64483723	3.22581609	4.54256898	5.70472972	1.555492	3.247399648
RTN4		+	+	+							3.31278584	3.9218146	3.66298983	3.32906596	3.2004233	3.327266057
RUVBL1											0.3528231	0.02578672	0.07420734	-0.18176651	0.0082084	-0.328200658
RUVBL2											0.75096302	0.32176654	0.69962399	0.98016675	0.1774121	-0.758349737
S100A10											0.07186069	-0.8273907	0.284602	-0.03459358	0.4819137	0.531084696
S100A11											2.116337	1.06507174	2.32679693	1.85368665	3.6093616	1.310735703
S100A14	+			+							0.42296485	0.14702225	1.01392605	0.71832339	3.6884604	7.565485001
S100A16											0.25717304	-0.0728938	1.50070055	1.67202822	1.149167	1.650019964
S100A4											0.10562012	-0.3278999	1.72842357	0.52371979	0.2059216	-0.123677572
S100A6											0.99564521	0.73298772	0.92656544	1.79956118	0.2025475	-0.191403707
S1PR3											0.27297066	-0.0245984	1.34454029	0.66193453	0.1790374	-0.333850861
SAE1											0.97426579	0.41624133	0.04343081	-0.64925575	0.5473028	0.873629252
SAMM50	+	+	+	+	+		+	+		+	4.27645672	4.8495636	3.00673457	3.86980883	3.3596905	4.426814397
SAR1A		+									2.63260894	2.45498403	1.41496402	1.76334826	1.3321402	2.302431742
SARS											2.24521837	0.74098015	1.26748573	0.28991699	0.8578921	0.102893194
SARS2											0.08750954	-0.6537787	0.05682419	-0.82728704	0.0107819	-2.056672414
SART3											0.34699427	0.11454837	0.14549122	-0.40298525	0.3759878	0.181605657
SBDS											0.54195331	0.4876887	0.9280345	1.21160316	0.1396938	-0.821536382
SCAMP1		+	+	+							2.38224241	4.89661789	3.33503056	5.54314486	2.3451204	3.839703878

SCAMP2											0.78282016	0.35669136	0.29853655	-0.00539017	0.0726295	-1.293164571
SCAMP2		+	+	+							3.48220496	4.18701808	3.94097369	4.71871312	4.8860352	4.695318858
SCAMP3		+	+	+							4.06436162	7.01676687	5.64468071	7.71009763	5.2214162	7.683427811
SCAMP4		+	+	+							4.57693361	6.04057058	3.7691129	3.66357803	3.7955794	5.341930389
SCAP											0.77587318	0.83764013	0.68616313	0.50604184	0.217924	-0.151643117
SCARB1	+		+	+							0.4367288	0.15580368	4.07535967	4.34377543	5.6297069	5.444201152
SCARB2	+	+	+	+							4.58660268	8.31153933	7.00749178	8.66787275	4.2823526	8.063446045
SCARF2		+									5.32273359	2.15855281	0.07348943	-0.45335897	0.0223336	-1.261780421
SCD				+							0.29759234	-0.0040862	0.70174438	1.13987986	3.5020223	4.852065404
SCFD1	+										0.88590956	0.60737228	1.07621482	1.03565598	0.0586906	-0.664567947
SCP2	+										0.19594423	-0.1383286	0.04232004	-0.71648534	0.3506856	0.110230128
SCPEP1											0.11406137	-0.3220406	1.81389897	0.99786758	0.0296236	-1.16388003
SCRIB		+	+	+							3.07480844	4.81693014	2.63720613	2.59542338	4.3046132	3.608307521
SCRN1											0.14911987	-0.3802929	0.22331982	-0.12852732	0.0582978	-0.399529139
SCYL1											0.03811332	-0.7803103	0.01627874	-0.64980634	1.046157	0.764734904
SCYL3	+	+	+	+	+	+	+	+			4.73035028	4.89751244	3.53880097	4.40996297	4.8732731	4.561777751
SDC1				+							0.91861017	1.05744298	0.22038244	-0.18450546	3.3164739	3.295544306
SDHA											0.27826362	-0.0202065	0.03395391	-0.29028384	0.025564	-0.293166478
SDHB											0.28342545	-0.0335356	1.41479383	0.68762716	0.0852972	-0.789841334
SDHC		+	+								2.39457537	3.63971519	5.01567261	3.56880569	0.314146	0.061437607
SEC13											0.46761942	0.19265366	0.06731341	-0.96054141	0.001078	-1.169830322
SEC22B											0.09532363	-0.3339373	0.49993324	0.62788264	0.0531106	-0.538344065
SEC23A											0.00634365	-1.2150313	0.56470842	0.14536667	0.0506743	-0.521790187
SEC23B											0.23358543	-0.1151466	0.00664848	-0.76063093	0.6174326	0.418732325
SEC24C											0.15971417	-0.4149647	0.40896468	0.15126483	0.0290586	-0.577329
SEC31A											0.53927218	0.66420237	0.04529526	-0.61698977	0.299034	-0.001634598
SEC61A1;SEC61A2	+	+	+	+							3.87348032	2.64694722	1.89091266	2.17311478	2.4970026	2.237707774
SEC61G											0.1621441	-0.1409817	0.26748319	-0.05062358	0.0681227	-0.60132281
SEC63											0.40424857	0.11777242	1.07786476	1.8150959	1.2496368	1.389618556
SELENBP1											2.14469687	0.79046249	0.78373286	0.53365962	0.0034108	-1.169563929
SEPT11;SEPT6											0.16666862	-0.0623023	0.63850359	1.0141805	0.6543102	0.547347387
SEPT2		+									2.8795765	2.86976306	0.57369855	0.30148125	1.8071681	0.667486827
SEPT7											0.80204312	0.18310229	0.74053386	0.78569349	0.3109566	0.023742676
SEPT9	+										0.10696716	-0.8712298	0.23959966	-0.03100522	1.8682652	0.251296997
SERBP1											0.02837202	-1.3243351	0.42653715	0.31530253	0.0011899	-0.694447835
SERINC1	+	+	+	+							6.01809795	7.22791545	5.76479575	6.11979802	5.6618082	5.726558685
SERINC3	+	+	+	+							7.056823	5.75237465	5.48279539	7.08974139	4.3115525	6.045027415
SERINC5		+		+							3.05809536	4.21793938	0.2557032	-0.08883031	3.1058124	5.210983276
SERPINB5											0.23353497	-0.1314348	0.95564042	0.05222638	0.1540979	-0.572540283
SERPINB6		+	+	+							4.02962375	4.36473211	4.69195401	5.79154015	5.7536296	5.653251012
SERPINH1											0.56119527	0.23323631	0.52813642	0.06989161	0.6938974	0.129470189
SET											0.12499292	-0.2980213	0.02869648	-1.76811409	0.0084435	-1.071156184
SF1											0.0047908	-1.3546658	0.23964181	-0.04991849	0.1495579	-0.460468292
SF3A3											0.58475	0.77467028	0.75059664	1.46586037	0.7198334	1.035083135
SF3B1											0.47117412	0.31090228	0.85642488	0.30659866	0.1158154	-0.902908325
SF3B2											0.1529494	-0.3546486	0.03330962	-0.51591047	0.0200921	-0.88489151
SF3B3											0.0008741	-0.9499944	0.00043069	-0.86957741	0.0011231	-0.479959488
SF3B4											0.05512659	-0.6409327	0.62553876	0.2684803	0.0265175	-0.660858154
SFN											2.01710366	1.05387688	0.88409102	0.42357254	0.5319776	0.481477102
SFPQ											0.01148756	-1.4565353	0.00048833	-1.130874	0.4570434	0.379933675

SFRS3;SRSF3											0.30641721	0.01013947	0.19325073	-0.12535477	0.121152	-0.404010137
SFT2D1											0.2921513	-0.0116584	0.09145511	-0.41562589	0.4201812	0.107748032
SFT2D2											0.40596207	0.19134267	0.34503298	0.08464877	0.3376247	0.088972727
SFT2D3		+	+	+							4.988295	5.60947673	3.96782946	3.31941923	4.2033227	4.818337123
SFXN1		+	+	+							2.92993522	2.28415426	5.25099214	2.50899951	3.5219972	2.391708374
SFXN3	+		+								0.05911492	-0.5962474	1.93918668	2.13793691	0.7073191	0.709442139
SGCE		+	+								3.9563793	2.50916672	3.93040354	3.681029	1.6004167	0.906633377
SGMS2											0.25060444	-0.1322187	0.38114894	0.08607992	0.2455766	-0.106397629
SGTA											1.5422178	1.25023524	0.46182225	0.22092756	0.0009893	-2.150942485
SHISA2		+									2.49927011	2.69562912	0.06082456	-0.87870534	0.2915078	-0.014268239
SHMT1	+										1.1580767	0.7441349	0.82732517	1.03487905	1.6697609	0.514607747
SHMT2	+										2.34855692	0.58539836	0.49960031	0.05574989	1.3505301	0.155289332
SHPK											0.94163434	0.44365565	0.14591231	-0.33080355	1.2895884	1.355978012
SIGIRR											0.01652469	-0.9136054	0.10987512	-0.44340897	0.6689939	0.643011729
SIGMAR1	+										0.43126543	0.27614339	0.24580301	-0.14976756	0.1390735	-0.371753057
SKIV2L2											0.40859806	0.16639392	0.4100653	0.14365641	0.2765254	-0.063604355
SKP1			+								0.47791171	0.35355059	4.00005797	1.94749324	0.2617967	-0.140349706
SLC11A2		+									2.45770015	3.40447744	0.22221147	-0.10840416	1.334667	1.505605062
SLC12A9											0.02208351	-0.9079583	0.02586702	-1.0274264	0.0458596	-0.415416082
SLC15A4											1.41383605	0.88943926	0.45123622	0.20844332	0.8580805	0.450581868
SLC16A1		+	+								1.59544558	2.01703962	1.93116443	2.91357104	0.0603126	-0.827091853
SLC16A3	+		+								0.32166735	0.0354379	4.0445468	3.03857231	0.114969	-0.638776143
SLC17A5		+									4.28439229	3.17189153	1.5658564	1.20416069	0.6264367	0.583447774
SLC19A1				+							0.0656266	-0.5282942	0.80345312	1.02507909	3.865479	2.492242177
SLC19A2											0.0308365	-1.2056967	0.32680424	0.0511392	0.3086605	0.012149175
SLC1A4		+	+								4.87702978	4.41437785	3.71652096	1.8294576	0.0086019	-0.771312078
SLC1A5		+	+	+							4.62267312	7.27588463	6.90299064	6.04178111	3.3252737	5.865706126
SLC22A18											0.01491543	-0.7455228	0.91847366	0.61082013	0.074855	-0.386206945
SLC23A2		+									2.79421256	1.65854963	1.53101369	1.3579305	1.1321132	0.896448135
SLC25A1		+	+								1.52899078	3.20154254	3.99068135	2.78600311	2.4578861	1.09341685
SLC25A10											2.27252687	0.87706502	0.34581286	0.09341621	1.9202216	0.708271027
SLC25A11		+									1.75695609	2.04338201	3.45061408	1.30767441	1.1144809	1.918961207
SLC25A13											1.38662606	0.4139328	2.14827285	0.40959167	1.70364	1.94262886
SLC25A22		+		+							3.17495331	2.74585215	1.04300786	1.33747419	2.7363215	1.958544413
SLC25A24		+	+								2.7634999	2.87141037	3.61678784	2.88892428	5.4955229	1.586128871
SLC25A3											3.24779216	1.27684784	2.83026765	0.91051038	1.9905657	0.705645243
SLC25A32			+	+							0.19129894	-0.1453762	3.78411469	4.14052582	2.9275383	2.21991984
SLC25A4	+										2.42926284	1.43341573	0.86915156	0.98136584	2.3015145	1.835066477
SLC25A5											2.08849656	1.15300369	3.44362642	0.87082863	4.4634945	0.894790014
SLC25A6											3.26784064	1.28157361	3.55324865	0.97539965	3.89359	0.977200826
SLC26A11		+									1.84582667	2.48796527	0.30344614	0.00522168	0.4103974	0.134592056
SLC27A2				+							0.45043919	0.16305161	2.70692798	1.69650714	3.3981352	3.250563304
SLC27A3	+			+							0.1444728	-0.4428647	0.11104077	-0.33400027	6.2876456	5.405802409
SLC27A4		+		+							2.14696313	1.80869993	3.65201335	1.63935407	3.2522907	1.812241872
SLC29A1			+	+							2.30319784	1.50039228	1.88331109	2.68042437	1.7502749	2.085154851
SLC29A2											0.49851571	0.11987813	0.01653607	-1.10031382	0.0343227	-0.906038284
SLC2A1		+	+	+							2.71286107	2.67049154	4.02188099	3.05921173	3.0083039	3.437772115
SLC2A13											0.03131906	-0.5867538	0.13950939	-0.35935847	0.0975265	-0.530741374
SLC2A3;SLC2A14	+	+									2.01562861	2.16452471	0.38203109	0.06638972	0.1435455	-0.429259618
SLC30A1	+	+	+	+							4.62397273	5.29838053	3.78456097	6.53709666	4.006486	5.324124654

SLC30A6	+	+		+							2.69009875	2.01298141	2.74837449	1.48651059	4.2882945	2.241281509
SLC35B2		+	+	+							4.72494024	5.75720088	6.63769797	5.40222804	4.2329569	5.872968038
SLC35C1		+									3.46898487	2.88774172	0.11579541	-0.68671481	0.0628577	-0.43905894
SLC35C2	+										0.21220972	-0.0865078	0.16385256	-0.4409523	0.272084	-0.05076472
SLC35E1				+							2.37906354	1.11712519	1.52662473	1.14558156	5.3296945	3.398106893
SLC35E2B;SLC35E2		+									2.98799955	3.88391304	0.82812619	1.22774315	0.1235533	-0.322905858
SLC35F6;C2orf18	+	+	+	+							2.86620935	2.20429866	3.96021327	2.88677597	3.9291134	2.568404516
SLC36A1											0.15051742	-0.2393386	0.3221771	0.02548154	0.5674861	0.450380961
SLC37A4											0.76549303	0.36064021	0.14772502	-0.51163101	0.0543473	-0.389096578
SLC38A1		+	+	+							3.64045736	4.16651917	4.39928889	5.06181717	4.3678471	5.674921036
SLC38A2		+	+	+							4.83804148	7.06088956	4.83685875	5.96241951	4.3763014	6.162672679
SLC38A5			+								0.72778757	0.60573069	1.91159979	3.02531942	0.3371552	0.055985769
SLC39A10											1.45872136	1.2087129	1.44214233	1.27037239	0.0172023	-1.193857193
SLC39A14			+								0.84717228	0.71667926	3.89372985	2.62700272	0.0924888	-0.822247823
SLC3A2		+									2.3077332	3.35633723	5.57007387	1.42109044	4.7528391	1.430803935
SLC41A1											0.53223324	0.22919401	0.1286636	-0.3682607	0.0544231	-0.757600784
SLC41A3		+									4.32986893	3.29881732	1.6751805	1.02831586	3.2884291	1.316111247
SLC43A3											0.37232699	0.05749766	0.77593395	1.24312655	1.3976929	0.414822896
SLC44A1	+	+	+	+	+	+	+	+	+	+	5.29165213	6.98991966	3.84958964	6.09973653	5.2899128	8.397553126
SLC44A2	+	+	+	+							3.75118395	4.54690552	3.50705734	4.75163333	5.142964	6.121195475
SLC46A1											0.02639641	-1.1086337	0.00476298	-0.55779203	0.5395734	0.268420537
SLC5A6		+	+	+							2.82887688	3.37196922	4.47481131	4.48071734	6.0695964	6.777966181
SLC6A14											0.11452353	-0.4345951	0.01019672	-1.12126287	0.3789608	0.194854736
SLC7A1	+	+	+	+							5.27274941	3.95763715	2.41140704	4.20736376	5.0154241	3.477181753
SLC7A2		+	+	+							4.97429951	4.96040408	5.51169377	5.45964686	3.977674	6.341187159
SLC7A5		+	+	+							2.95553123	2.2294515	4.00599766	2.23842049	4.6975768	2.484886805
SLC9A3R1											1.53699103	0.82828776	0.87037664	1.30268606	0.3269367	0.015272776
SLC9A6		+	+								5.7027071	3.88403002	4.19833148	3.33572006	1.9813262	1.501326879
SLC9A7											0.00591205	-0.7106686	0.15759171	-0.25830841	0.2340255	-0.106567383
SLCO3A1			+								0.13460566	-0.4334895	2.83705938	2.41727257	0.383691	0.161629995
SLCO4A1											0.88481325	0.56683477	1.96071631	1.88370641	0.206295	-0.205095291
SLFN11											0.15680874	-0.3558394	0.01663077	-0.88575808	0.0368787	-0.783089956
SLIRP											0.83588995	0.84910647	1.50129532	2.11041069	0.8265107	1.261222839
SLITRK6;SLITRK5											0.25674759	-0.0570234	0.21566217	-0.1405913	0.7524615	0.380909602
SMC4											0.02769112	-0.5947984	0.39850633	0.1680336	0.255564	-0.083288193
SMDT1											0.00699609	-0.9625696	0.65265515	2.28700193	0.5171743	0.204949697
SMIM1											0.15309364	-0.3073279	0.05537253	-0.76101557	0.0810098	-0.464868546
SMS											0.59593788	0.16361809	1.35515581	2.33557765	0.0205883	-1.250815709
SNAP23		+	+	+							5.65652751	6.14810308	4.0747328	5.28097534	3.2816933	4.573660533
SNAP25											0.39910422	0.21670405	1.17728683	1.93717829	0.0330982	-0.938527425
SND1											0.00069323	-0.3944842	0.00047029	-0.80542437	0.0153341	-0.427726746
SNRNP200											0.29162497	-0.112896	0.06867279	-3.5172418	0.2637526	-0.475111008
SNRNP70											0.35815325	0.11580594	0.00021039	-1.77143478	0.001386	-1.425158819
SNRPD2											0.08932231	-0.505064	0.38196796	0.09311358	0.4901602	0.204371134
SNRPD3											0.95718188	0.60519791	1.04401552	0.61599541	0.0583333	-0.632301966
SNRPE											0.21932388	-0.1124763	0.71273535	0.41551272	0.04448	-0.924553553
SNRPN;SNRPB		+	+								1.87033338	2.2120622	3.81914478	1.9077301	1.0979169	1.132752736
SNX27	+										0.00498687	-1.289636	0.04516696	-0.88016256	0.3381758	0.041550318
SNX6											0.35341449	0.05055364	1.70528769	0.50799243	0.1934193	-0.379315694
SOAT1		+	+								2.36958311	2.56921069	1.53346197	2.86252467	0.0806203	-0.778799693

SORT1		+		+							5.39704652	3.84103584	1.74620287	2.05647278	5.2660304	6.480529149
SPAG1			+	+							1.18887872	1.05265808	4.37663426	4.71212769	4.990539	4.871581395
SPCS1											0.66473559	0.58433787	0.20648569	-0.17912865	0.0207783	-1.170722961
SPCS2											0.37388432	0.11098989	0.79041398	0.56528155	0.4794652	0.279311498
SPCS3											0.04737544	-0.8493481	0.28925752	-0.01839765	0.386894	0.158601761
SPECC1	+	+	+	+	+	+	+	+	+	+	3.20491019	3.95191511	3.82586121	6.48650424	5.3727769	4.383079529
SPINT1				+							0.36040448	0.1131738	0.72835717	0.49475479	5.3092313	3.311370214
SPNS1											0.24985401	-0.0541083	0.65958314	0.49406052	0.1497364	-0.296966553
SPPL2A	+	+	+	+							3.02686315	3.6330471	4.66331069	3.67673683	5.0850673	5.208098094
SPPL3											1.7298552	1.77175903	0.96426654	0.88110034	1.0229431	0.645318985
SPR											0.61244128	0.38504219	0.1759206	-0.22126134	0.0223527	-0.754259109
SPRED1											0.2319666	-0.237449	0.10359069	-0.23005168	0.0993007	-0.434369405
SPRED2		+		+							2.6174641	1.77098974	0.82446191	0.79149119	2.6353174	2.590473175
SPRY4											0.95389336	0.66215769	0.40415669	0.15630404	0.6693775	0.622635523
SPRYD7		+	+	+							5.41473299	5.41049067	3.66483794	4.31619008	3.4417085	3.947374344
SPTBN5											0.5161126	0.47922389	0.83626434	1.26963743	0.3799151	0.117354075
SQLE				+							0.04810753	-0.589674	0.12719893	-0.59922091	4.0568459	2.591344198
SRC	+	+	+	+							5.31735661	8.01279767	5.54541649	6.95490456	4.7747203	7.08919843
SRI											1.39279214	0.85225423	1.56076572	0.60606702	0.9323943	0.301628749
SRM	+										0.11533012	-0.4576257	0.10885799	-0.35810089	0.2191187	-0.224308014
SRP14											3.85606063	0.50283623	2.31570948	0.66312663	1.221707	1.294291178
SRP54											0.25544453	-0.1306585	0.27668754	-0.04329554	0.0306394	-0.446963628
SRP68	+										2.04888343	0.43781662	0.08582581	-0.19778379	0.0781287	-0.873540243
SRP72		+									3.10785483	1.77262561	0.31691736	0.03845024	0.5955695	0.499679565
SRP9											0.08966869	-0.4686483	0.28827387	-0.02366257	0.3261257	0.048645655
SRPR											0.23460044	-0.0737597	0.81196331	0.21047783	1.0482208	0.729345957
SRPRB			+	+							0.21322538	-0.1530488	3.20202181	1.87754695	2.4182213	2.146887461
SRRM1	+										0.48039655	0.38480377	0.14586168	-0.74126498	0.041144	-0.595195134
SRRM2											0.0010678	-0.8535652	1.12E-05	-1.65956752	0.0002744	-0.892201742
SRRT	+										0.27293192	-0.0334765	1.48485	0.9152209	0.0520082	-1.225811005
SRSF11											0.01507593	-0.7683226	0.00369741	-0.9034481	0.0007739	-1.28044637
SRSF6											0.09188732	-1.2861691	0.07165074	-0.96616236	0.021335	-1.356245041
SRSF7											0.00359279	-2.1772582	0.00566341	-1.83770752	0.0190401	-0.769986471
SSB											2.07139706	1.01493454	0.96912922	1.49226189	0.5179235	0.48559761
SSR4											0.48378617	0.31933912	0.4781659	0.257267	0.116335	-0.46521314
SSRP1											0.24838999	-0.1341267	0.00971759	-0.71898142	0.0630388	-1.096312205
SSSCA1	+										0.99483717	0.50774256	1.5039423	1.60216586	0.1612622	-0.317484538
ST13;ST13P4;ST13P5	+										2.83265229	0.53250058	1.15675328	0.29972267	0.6250295	0.125855764
STAM2;STAM											0.1055905	-0.4290759	0.56205029	0.42306391	0.007134	-1.24434789
STAMBPL1											0.45338535	0.22812589	0.34276378	0.05553691	1.5079622	0.869561513
STARD10											0.54463905	0.42591985	0.09881878	-0.46397654	0.0089486	-1.07185936
STARD3NL		+	+								3.97786545	3.25203705	5.03610358	2.83806992	1.8903548	1.68454361
STAT1											0.02778998	-0.8554948	0.39820605	0.10816065	0.601778	0.636824926
STAT3											0.02806035	-0.6432101	2.06058861	0.46630414	0.7570768	0.186150869
STBD1	+	+	+	+							3.55271323	3.0337251	4.16514521	5.02030309	2.8515765	3.033400853
STEAP3		+	+	+							4.40630773	3.86051432	4.09198845	5.11191114	3.5221424	4.725072225
STIP1											0.59640272	0.08195051	0.00466143	-0.27336502	0.0133612	-0.219834646
STK16	+	+	+	+							2.02845774	2.10631943	4.56506174	3.69979413	4.1570824	2.812885284
STK24;STK25;MST4											1.64306544	0.55427996	1.05909567	0.92750486	0.0386996	-1.009097417
STOM		+	+	+							5.27362142	5.95503553	4.22718541	7.64365387	5.2887629	6.940309525

STOML1											1.62578991	1.13318062	0.96409147	0.41137632	0.4877105	0.123395284
STOML2											0.34869294	0.10238902	0.05989074	-0.46613693	0.7802046	0.535471598
STRAP											0.90930197	0.10842959	0.30931124	0.00627391	0.5518798	0.072999318
STT3A				+							2.76590948	1.45945803	3.20834532	1.35219765	3.7150428	1.737044017
STT3B		+		+							2.44999768	2.87489446	1.22922954	2.09464518	1.5383775	2.510045369
STUB1											0.81443422	1.05962817	0.00357575	-0.95869319	0.3270335	0.039207458
STX10		+	+	+							2.23412885	3.12508011	2.70012077	3.55420431	3.8427547	2.48216629
STX11											0.125739	-0.3065987	0.04773846	-0.77707227	0.1047798	-0.411708832
STX12		+	+	+							3.68693315	5.92159589	4.84139678	4.97038523	5.2291424	4.818850835
STX4	+		+								0.07061821	-0.9476198	2.67940956	2.79959997	2.5689808	1.444378535
STX6		+	+	+							3.44279589	3.69374657	3.58922372	3.93579865	4.8412429	5.367488225
STX7		+	+	+							4.08823268	5.92034531	3.57367116	3.87382189	5.2754095	4.720450719
STX8		+	+	+							3.7479859	4.50311406	3.69878219	4.34795252	4.1373471	3.641438802
SUCLA2											0.60393319	0.37559128	0.18653046	-0.26470248	0.0922545	-0.574678421
SUCLG2											0.74369095	0.90640831	0.47162097	0.23599625	0.3921971	0.219364166
SULF1											0.06578698	-0.4074345	0.00893896	-0.57906024	0.8572317	1.901134491
SULT1A1;SULT1A2;SULT1A3											0.28956181	-0.0221888	0.72217058	0.23607699	0.1179436	-0.305330276
SULT2B1											0.68801469	0.32706833	0.03408991	-0.74547577	0.4808614	0.53626887
SUMO1											0.53444658	0.39675268	0.17501052	-0.35742251	0.1727388	-0.382059097
SUN2											0.06366045	-0.8068651	0.22871066	-0.08740679	0.0216769	-2.096890132
SUPT16H											0.60400904	0.74130758	0.042786	-1.61526934	1.2371546	1.630673091
SURF4	+	+	+	+							3.34301935	2.94843419	4.187497	2.76237297	4.809636	2.812932968
SVIP	+	+						+	+		5.31901594	6.37938309	0.66213917	0.43083827	0.1829918	-0.277613322
SYNCRIP											0.01090026	-0.5350424	0.00059567	-0.64325651	0.003117	-0.564777374
SYNGR2											0.3371493	0.06490771	0.06268754	-0.61857859	0.8970024	1.623058955
SYPL1											0.44949939	0.26672173	0.16549821	-0.37915738	0.5138908	0.453195572
SYT1		+		+							3.97511309	3.61468569	0.2885461	-0.014534	3.1886065	4.667097727
SYT7											2.10307315	1.03834534	0.36397853	0.09072367	0.0353791	-0.540538152
SYVN1	+										0.16689175	-0.3344167	0.5130386	0.50813293	1.5098443	1.819517136
TACC1	+		+		+	+		+	+		0.20053296	-0.2056586	2.83471147	3.45720545	0.062549	-0.868892034
TACC1		+	+		+	+					3.20315882	2.27514903	4.79733715	5.682621	0.183496	-0.242815653
TACC2	+										0.2184181	-0.1360582	0.11609232	-0.83010928	0.0625796	-0.691865921
TACO1											0.03449968	-1.0240688	1.07181515	0.67358971	0.0481377	-0.297376633
TAGLN											0.61215757	0.37378184	3.72100723	1.56112925	0.0497666	-1.114957809
TAGLN2											3.8769786	0.88025729	4.55123	0.96733602	1.9201556	0.294389089
TALDO1											1.69174941	0.44518344	2.42707606	0.6064949	0.8828189	0.214901606
TARS											0.0050457	-0.3643068	3.58E-05	-0.54629453	0.1346218	-0.065474828
TBCB;CKAP1											0.31373742	0.01979192	0.5111535	0.32079379	0.5130738	0.190364838
TBCD	+										0.44025544	0.15044785	1.15948961	0.48643939	0.965935	0.344226201
TBRG4											0.06584951	-0.8254611	0.54506364	0.31560135	1.7848715	1.180684408
TCEB1											0.40972796	0.13734182	0.1157843	-0.4038105	0.344465	0.142402013
TCEB2											0.17176734	-0.2152812	0.49690832	0.3004907	0.0954929	-1.144407908
TCP1											0.01920412	-0.1709099	0.00196087	-0.27383677	0.0362736	-0.101224264
TEAD1	+										1.75588528	1.26564407	0.56139469	0.46227074	0.0008552	-1.327283223
TECR	+										2.22590217	0.83933767	2.27113247	1.12058767	3.9226979	1.399028142
TELO2											1.4082045	1.03943507	1.64697334	1.20823987	0.3933587	0.103612264
TESC	+	+	+	+	+	+	+	+	+		5.43165558	4.61861865	5.41382452	9.92431259	3.630009	2.153181076
TFB2M			+								0.18619698	-0.2544403	3.03739712	1.75567945	0.6123681	0.508815765
TFPI											0.21477628	-0.2812983	0.03595372	-0.58589236	0.1398761	-0.67976888
TFRC		+	+	+							5.34210717	8.10798836	7.59016461	5.27082634	5.3465169	7.453898748

TGM2											0.20981752	-0.1532745	0.01417965	-0.30112966	0.0110122	-1.023036321
THEM6				+							0.6195437	0.74106916	1.4265835	1.44845454	3.8091317	2.263555527
THOP1											0.34886835	0.06404177	0.01125285	-0.50200335	0.5700138	0.237656911
THUMPD3											0.230861	-0.1856715	0.27668513	-0.04606883	1.02954	0.511914571
THY1											0.33181807	0.04251417	0.96564235	0.96210861	0.1059397	-0.66141065
TIAM1	+				+			+		+	0.47309293	0.15123876	0.50989755	0.162817	0.6208126	0.541821798
TICAM2;TMED7	+										0.43185094	0.20794741	2.92931539	0.87139511	0.5528557	0.513662338
TIMM44											0.19486474	-0.080403	1.49965038	0.70662944	0.5315506	0.323984782
TKT											1.70806264	0.22270203	0.80486971	0.09960365	2.4308996	0.332078298
TLCD1	+	+									3.08047054	1.98014132	0.34878983	0.06332906	0.032319	-0.921300252
TLDC1	+	+	+	+							5.86676004	6.40593274	4.16745755	6.53279368	4.3453492	7.126946767
TLE2;TLE3;TLE4;TLE1											0.11871404	-0.3663737	0.89220438	0.44707998	0.0258508	-0.555712382
TLN1											0.34248097	0.03153928	0.13554776	-0.02694702	1.290126	1.041836421
TM4SF1			+								1.36765163	0.65437253	3.48022524	2.33501625	0.1962353	-0.105117798
TM7SF2											0.16325536	-0.412789	0.59174787	0.39941279	0.8157023	0.730965932
TM9SF1			+	+							0.33309201	0.0410703	3.38092385	2.41179466	2.1304166	3.052522024
TM9SF2			+								1.69337693	1.08217176	2.98292442	2.52501043	1.9925767	1.513713837
TM9SF3											2.36188417	1.25877825	2.28973462	0.7218132	3.7946931	1.014087041
TM9SF4		+	+	+							3.18942359	2.05268224	2.9413514	4.15583483	2.7143892	3.357418696
TMBIM1											0.58579057	0.39977773	0.39978131	0.28433545	0.05605	-0.953838348
TMBIM6											0.21842776	-0.1020069	0.03101512	-1.1674811	0.571953	0.690014521
TMCO1											0.41361009	0.15733528	0.75692263	0.27571932	0.3028262	0.003576279
TMED1											0.78502175	1.7481041	0.33533589	0.10181681	0.175526	-0.291535695
TMED10			+								3.31828179	1.4558061	1.85763679	2.42129517	2.540288	1.056999842
TMEM106A	+										0.35960369	0.10346603	1.42489653	0.91165352	0.1003061	-0.763699214
TMEM106B	+	+	+	+							4.15097163	5.39608637	4.96531933	5.35558573	2.925344	3.868050893
TMEM106C	+	+	+	+	+	+	+	+	+	+	3.58261799	3.21940613	4.87560701	6.42437045	5.3703839	5.524436951
TMEM115		+	+	+							4.77629448	4.10944303	2.97957028	3.04555003	2.6769354	2.491577784
TMEM120B											0.38796192	0.1049099	0.2108841	-0.11213048	0.0363741	-0.36782074
TMEM123	+										0.36125153	0.05110614	0.01158433	-1.02129618	0.3960151	0.263496399
TMEM134											1.66479593	0.90133413	0.01926178	-0.73351288	0.5178273	0.417528152
TMEM161A											0.18685093	-0.2662284	0.08033198	-0.60754204	0.0508868	-0.693297704
TMEM165			+	+							1.16874199	1.23571523	2.3999344	3.02837753	2.4863038	1.904936473
TMEM167A											0.24813566	-0.1522834	1.64302085	1.75669797	0.2883895	-0.042256673
TMEM168		+	+	+							5.19258674	4.56701914	3.73443924	5.03205554	3.8654181	4.152671814
TMEM179B											0.77241344	0.50175985	0.05760328	-0.77871704	0.2955743	-0.015314738
TMEM181	+	+	+								6.13329666	3.88772774	3.49957313	2.4991862	0.1189245	-0.533920288
TMEM184B											0.71663655	0.4452947	0.49001645	0.38948568	0.0684006	-0.898466746
TMEM184C		+	+	+							3.0773783	3.36724472	4.81571987	2.86913236	1.9710542	3.428288142
TMEM192		+		+							4.85800463	3.96173668	2.17763109	1.66739591	2.8040454	3.809595108
TMEM2			+	+							0.01958083	-0.7896004	2.81257452	2.82530912	3.8033842	2.205177307
TMEM205	+										0.1998487	-0.288681	0.89344895	0.39848455	0.6596134	0.636821747
TMEM206											0.48842867	0.31190872	2.44039945	1.48695183	0.0027623	-0.716218948
TMEM214											0.4805449	0.24485524	2.77943713	1.75044568	0.0668307	-0.921668371
TMEM219	+										0.01439913	-0.7564405	0.72500432	1.3038667	0.7436596	0.554230372
TMEM222		+									4.28861666	3.14602343	0.0172021	-0.7698822	1.6524056	2.10911878
TMEM238											0.09699788	-0.5068366	0.07687853	-0.5927639	0.3847986	0.154394786
TMEM245				+							1.02607592	0.42241478	0.25781765	-0.04285431	3.1170506	1.918774287
TMEM251				+							0.57012936	0.37642097	0.99179628	0.63676198	2.8141935	3.793890635
TMEM33											2.38150059	1.27053642	2.16431	0.87318675	1.3211407	2.190328598

TMEM41A											0.31282427	0.02605756	0.51493486	0.46026039	0.8532825	0.534482956
TMEM43		+	+	+							2.28137154	1.87256622	4.0604884	3.0042909	3.5094944	2.696844737
TMEM50A		+	+	+							3.00690642	3.58495585	2.94019349	3.64599228	3.2663564	2.244272868
TMEM50B											0.10085283	-0.3384069	0.27038353	-0.03568649	0.1865632	-0.327823639
TMEM54											0.0566673	-1.2140121	0.21727305	-0.12611198	0.0114749	-1.082500458
TMEM55A		+									3.53226104	2.06118711	0.23972658	-0.15108744	0.2929666	-0.012149175
TMEM55B	+	+	+	+							4.83747812	4.41587448	3.31335777	3.18658956	4.2303072	4.655915578
TMEM56		+									2.61352817	1.84953372	0.11129605	-0.6301384	0.0012959	-1.177157084
TMEM59		+	+	+							3.15098451	3.04733149	3.29435253	3.28307597	2.1787146	2.755045573
TMEM63A		+	+	+							3.07015342	2.79156876	2.52681728	2.08526421	3.9806108	2.615618388
TMEM63B		+	+	+							4.1555494	3.90458743	3.96713018	3.81847318	5.1017175	4.17960612
TMEM64	+			+							0.81541218	0.56197548	0.07364787	-0.35330518	3.7205803	3.033768972
TMEM65		+									2.68134885	1.81865565	0.51760091	0.46485138	0.02136	-0.790980657
TMEM87A		+	+	+							3.29651568	3.92333794	3.80254739	5.89248339	4.5134601	4.720147451
TMEM87B											0.54540217	0.38755671	0.02326371	-0.67513847	0.6805266	1.218378703
TMEM8A	+										0.59425741	0.39356613	0.10424006	-0.42486509	0.1226699	-0.265642802
TMEM97	+	+									5.12830174	4.54939143	0.83704729	1.12882741	0.983595	1.636841456
TMPO											0.00025628	-2.7214788	0.02925996	-2.27338219	0.0001919	-2.803539276
TMPO											1.04185159	0.44427745	0.6237263	0.79694812	0.0276989	-0.285455704
TMPPE				+							0.08717058	-0.4646282	0.04817794	-1.15656153	3.1554473	1.993339539
TMX1		+	+	+							2.41592216	4.82559331	2.31216388	4.84332784	4.6050023	5.00965182
TMX2;TMX2-CTNND1											0.31341906	0.01173846	0.65324642	0.75306892	0.0152085	-0.856840769
TMX3		+	+	+							2.65667746	2.65351295	3.39766405	4.60348193	2.7729805	1.96620814
TMX4		+		+							5.12068351	2.27814929	0.20639293	-0.26547368	3.294003	2.725634893
TNFRSF10A		+	+	+							4.36646808	6.67482821	3.57403251	5.71513685	2.8561086	2.830532074
TNFRSF10B		+									3.32048881	3.24247805	0.35459245	0.05328051	0.1605014	-0.207115173
TNFRSF10D	+										0.67314248	0.49820773	0.22094246	-0.16353861	0.1168141	-0.379329046
TNFRSF12A											0.8018218	0.61842982	2.26315951	0.81434123	0.7739743	0.607844035
TNPO1											1.27805566	0.44373703	1.21004561	0.21833801	2.2407688	0.559797287
TNPO2											0.01979076	-1.2096691	0.02125105	-0.99965922	0.1015994	-0.526973089
TNPO3											0.48407125	0.3047123	0.52427243	0.47800573	0.2366588	-0.166899999
TOLLIP											0.12192683	-0.5110563	1.03280746	0.56937345	0.0012586	-1.199768066
TOMM22											0.26764005	-0.0451647	0.05229725	-0.97617404	0.3730174	0.151250839
TOMM40	+	+	+	+							2.06543111	2.96847661	2.62269068	3.42644946	1.634588	2.956447601
TOMM40L	+	+	+		+	+		+	+		2.09076914	2.522885	3.82439249	3.22908783	2.5063398	1.313240051
TOMM70A											0.74575317	1.00678126	0.27363012	-0.08507029	0.517964	0.44029363
TPBG			+	+							1.76151076	1.55089887	4.96916697	3.73595047	4.3640983	5.28137207
TPD52L2											1.36581646	0.69796499	4.62246138	1.32800992	0.2336463	-0.074560165
TPD52L2											0.00571476	-1.835158	0.00032714	-2.6293939	0.0001827	-3.901983261
TPI1											2.3006955	0.53160222	2.40130877	0.41772779	1.1532949	0.11318779
TPM3											1.09239996	0.89833132	0.08644016	-0.43145116	0.0885344	-0.330963135
TPP1	+										1.58073695	0.93094508	0.05263976	-1.06665675	0.0050322	-0.912094752
TPP2											0.33250109	0.0540301	0.06835179	-0.96735827	0.0961137	-0.81456693
TPT1	+										1.10697413	1.97596931	1.59251051	0.83064524	0.7547359	1.405510585
TRAM1			+								0.66488246	0.82527987	3.71293322	3.62788963	0.2407062	-0.214031855
TRAP1											1.58191413	0.27230072	0.13648801	-0.04731623	0.0284492	-0.147345861
TRAPPC3	+	+	+	+							4.04307242	5.69044113	3.53356137	6.15260569	4.0011661	5.243242264
TRIM23											0.0478014	-0.6936296	0.21528483	-0.07876968	0.7896112	2.571427663
TRIM25											0.90017698	0.51404889	0.0204684	-0.62524796	0.0673872	-0.41827774
TRIM28											0.02590749	-0.2738031	0.01062931	-0.56047821	0.0041857	-0.380976359

TRIM33											0.03820681	-0.9452489	0.1919008	-0.22878011	0.6423424	0.153427124
TRIP13											1.51728631	0.77753321	2.00395103	0.86512947	1.6238919	0.693026225
TRMT10C											0.60220258	0.11236254	0.31168448	0.01125781	0.0535653	-0.948486964
TRNT1											0.3480557	0.0403525	0.14886668	-0.37354596	0.700581	0.696385701
TROVE2											0.43408541	0.19158363	1.22633785	1.3284626	0.0783915	-0.253290176
TRPM4				+							0.10474758	-0.4675579	0.31428625	0.01706251	4.701111	4.737295151
TSFM											0.69661261	0.56208738	0.49941668	0.54255295	1.2205578	0.533709844
TSN											1.05465952	1.01952235	1.37869591	1.18361092	2.7324482	1.394467672
TSNAX;DISC1											2.09202381	1.29351934	0.89966751	0.2655805	0.1239355	-0.37456131
TSPAN1				+							0.14900837	-0.2746614	0.30720477	0.00730642	3.5964121	3.546279907
TSPAN13		+		+							3.65121827	4.61178462	1.97485267	1.92748515	3.6115058	5.591509501
TSPAN14		+	+	+							2.61986703	3.24123192	4.64811631	3.37511571	4.2702437	3.976378759
TSPAN15		+		+							4.32263323	2.21652921	0.30039276	-0.00077311	3.6289383	4.458089828
TSPAN18											0.54314775	0.3579642	0.38255414	0.40766017	0.2293391	-0.1417497
TSPAN3	+	+	+								4.72562259	7.02455266	2.83942585	5.19442685	1.5016676	0.436311086
TSPAN33											1.99840925	0.8908844	0.10980461	-0.67001025	0.1998644	-0.33801206
TSPAN4											0.01607389	-1.1418807	0.04930592	-0.61836243	0.871314	0.974476496
TSPAN6		+	+	+							4.34524529	6.40379969	4.4798031	6.76253001	3.3306588	5.339508692
TSPAN7	+	+									4.14974784	3.44122187	0.62742993	0.46414185	0.2707377	-0.041807175
TSPAN9		+	+	+							5.46077347	4.49844742	6.34355808	3.70278168	3.8640143	2.632166545
TSPO											0.48885665	0.16714732	0.22202068	-0.14645513	0.208646	-0.121665319
TSTA3	+										1.05439672	0.86073939	2.63675771	0.45514743	1.1666944	2.346942902
TTC1	+										0.7370888	0.39488347	0.142202	-0.4070638	0.0130334	-1.422571818
TTLL12											0.24130466	-0.0955086	0.01775795	-0.48342832	1.0806526	0.70839564
TTYH3		+	+	+							4.03442413	2.86825307	3.86636281	3.9404494	4.0904319	3.762369156
TUBA1B		+	+	+							4.27659817	2.99274699	5.40314726	2.61130778	5.7221594	2.892435074
TUBA1C;TUBA1B											3.09E-05	-2.777633	0.00012813	-3.00142543	0.0001938	-2.703407288
TUBA4A	+										0.14205717	-0.4312909	0.11408926	-0.09462293	0.0004489	-0.715272268
TUBAL3											0.31051957	0.00927099	0.57828635	0.29717318	0.0481212	-0.433645884
TUBB											1.91357199	0.45533117	0.27669372	-0.01443354	2.6636063	0.261583328
TUBB2A;TUBB2B		+									1.50017356	2.07900556	0.39975109	0.21444893	0.2826776	-0.042413712
TUBB3											0.54954239	0.2816054	0.0595673	-0.6331075	0.6509973	0.870475133
TUBB4B											1.32660923	0.29842504	1.67667528	0.13178762	2.8287476	0.301262538
TUBB6											3.53590656	1.26110077	1.59543607	0.46572431	0.1023113	-0.790297826
TUBB8											0.72625667	1.52133497	0.47387149	0.18890381	0.8263803	2.116608302
TUFM											0.15155751	-0.0360292	0.33612353	0.008962	0.0065797	-0.244479497
TUSC2	+	+	+	+		+					4.37019071	2.98615901	3.46130491	4.44919459	4.0919811	4.786469777
TWF1											0.0207239	-0.8431543	0.08353439	-0.46434402	0.1016042	-1.294617971
TXN											0.01558865	-2.2227408	0.90492697	0.74517377	0.1357121	-0.168965022
TXNDC12											0.12611841	-0.4132353	1.09260255	0.63693237	0.0520254	-0.790608088
TXNDC17											0.64905365	0.34586716	0.04456069	-1.1937205	0.1437185	-0.362433751
TXNDC5											0.14470953	-0.1568089	0.11476273	-0.16485151	0.0036079	-0.861640294
TXNRD1	+										1.47666822	1.71059481	0.3130171	0.00972366	0.2220102	-0.303635279
TYMS											0.24149558	-0.1126143	0.08827817	-0.41527748	0.0721522	-0.53243192
TYSND1		+	+	+							2.40728982	2.51340485	4.20007567	3.05707359	2.6570292	3.024616877
U2AF2											1.1658905	1.55443255	0.78558085	0.53404681	0.3298197	0.07756424
UBA1	+										0.02741107	-0.1896477	0.00036348	-0.32368596	0.2592263	-0.008996328
UBA2											1.55006118	0.31495476	0.54426325	0.52509753	0.4319735	0.406670252
UBA6											0.00210754	-0.7593632	0.15486887	-0.22223473	0.2725314	-0.028081894
UBE2D2;UBE2D3		+									8.91841386	2.59180005	0.87012381	1.11473974	0.8027399	1.674163818

UBE2I											0.03999885	-0.5620817	2.62277326	1.50181834	1.2667613	0.478761673
UBE2K											0.8449194	0.42788951	0.71590296	0.63604291	0.5382755	0.282479604
UBE2M		+									2.5230649	1.80569967	0.04338063	-0.72911962	0.0398468	-0.549616496
UBE2N;UBE2NL											0.5642899	0.10407194	2.74172496	1.24035772	1.4600565	0.428597132
UBE2V1;TMEM189;UBE2V2											2.1949634	1.02494748	0.74026887	0.89856466	1.3280569	0.227321625
UBIAD1											0.00977334	-0.7969214	1.99639714	0.9305884	0.0560601	-0.789981206
UBL3											0.77516834	0.46850268	0.03732965	-0.45290693	0.1398453	-0.525019964
UBTD1	+	+	+	+							3.64959199	3.81351217	3.88089298	3.4469649	3.8901009	2.681990306
UBTD2	+	+									1.58871648	2.19754219	1.87378108	1.63021787	1.6783037	1.168991725
UCHL1											2.38516488	0.55677096	0.18328512	-0.42450587	0.0078629	-1.068138123
UCHL3											0.64725917	0.41632207	0.07670493	-0.748703	0.1742356	-0.442001979
UCHL5											0.06547978	-0.7606614	0.01300474	-1.23940531	0.7440148	0.482741038
UFC1			+								0.35473894	0.07226245	1.71331788	2.16325315	0.2298612	-0.141639709
UGDH	+										1.24349523	1.81586901	0.007985	-0.60060946	0.264509	-0.021816254
UGGT1;UGGT2	+										0.0658936	-0.8413734	0.01182171	-0.78879929	0.0161854	-1.178078969
UGP2											0.05581585	-0.2757772	0.1550621	-0.31551997	0.1708309	-0.226893107
ULBP2											0.33180342	0.07713318	0.28657762	-0.02325439	0.3459797	0.080533346
ULBP3											0.64567417	0.52438736	0.09166845	-0.5502828	0.0262445	-2.168010076
UMODL1											0.35942999	0.07906723	0.01322402	-1.23741531	0.3188226	0.060804367
UMPS											0.22814202	-0.1134332	0.44473112	0.05448532	0.4110275	0.042536418
UNC13D											0.15227078	-0.2761211	0.12440195	-0.5500927	0.2063116	-0.278308233
UNC45A				+							0.70055444	0.60165914	1.49422316	1.7830073	3.0810231	2.246619542
UPF1											0.12337645	-0.1189524	0.09581259	-0.98917961	0.0466967	-0.419636408
UQCR10											0.28262386	-0.0716972	0.79063711	1.41915703	0.5265632	0.791576385
UQCRC1											0.78814956	0.08492788	0.1632682	-0.11501058	0.9512141	0.182981491
UQCRC2											0.70877015	1.19339689	0.02111014	-0.4783268	0.0985733	-0.168409348
UQCRQ	+										0.0067091	-0.919555	0.00020038	-1.54658953	0.0036287	-1.90524737
USMG5											0.53033283	0.32572873	0.88269051	0.75031598	0.1119805	-0.760276159
USO1											0.64107936	0.6663545	0.39026857	0.12506358	0.2697012	-0.029389064
USP12											0.2461973	-0.1046308	0.07374581	-0.61113103	0.0685052	-0.628083547
USP14											0.38567194	0.04162979	0.49203327	0.13958104	0.5336252	0.100403468
USP32	+	+	+	+							4.53288085	4.55791728	3.67491988	4.99994914	6.266798	7.707603455
USP46		+	+	+							4.21693926	3.27078311	3.98663288	3.18373299	3.1361319	3.148617427
USP5											0.00302539	-0.3243796	0.00662686	-0.55878258	0.0024946	-0.460807165
USP7			+								2.78224074	1.30201976	3.65085651	2.70431074	2.3997684	1.655572891
USP9Y;USP9X											0.04942207	-0.4866517	0.66051145	0.43889999	0.2216573	-0.152301788
VAMP3;VAMP2		+	+	+							3.55359169	5.75217565	4.98216231	5.89391645	5.6049139	5.084294637
VAMP4		+	+								3.51369328	2.78718567	2.33429772	2.158535	0.7014912	0.526573181
VAMP7		+	+	+							4.92273653	5.59950066	3.16886203	4.98051135	5.2827443	4.032155991
VANGL1		+	+	+							3.64478494	3.16997973	3.15686466	2.83052572	3.9433158	5.580887477
VANGL2		+									2.72398095	2.20845477	0.25878394	-0.06814321	0.3781186	0.144374847
VAPB											0.30951699	0.02325567	0.10001259	-0.57252566	1.4527155	2.126856486
VARS											0.40542656	0.14052391	0.00096632	-0.87921715	0.0283478	-0.478830973
VASN			+	+							1.29093198	0.89074707	5.39431098	5.1690108	4.9283455	2.603122075
VASP											0.06386737	-0.4246616	0.75933111	0.52883085	0.0395161	-0.823614756
VAT1											1.11248542	0.14357249	2.39561531	0.54590543	1.161368	0.419993083
VBP1											0.24077855	-0.0903753	0.10763042	-0.70215098	0.1490968	-0.462387721
VCL											2.0720452	0.53423882	2.63012215	0.41338348	1.7477915	0.521541595
VCP											0.03457617	-0.2838796	3.22E-05	-0.49590619	0.015014	-0.235346476
VDAC1				+							2.22618744	0.77757708	1.00802508	1.4168733	3.6525844	3.763499578

VDAC2		+									3.01608683	1.83052444	2.75955647	1.22012266	3.5694007	1.341637929
VDAC3			+								4.19237547	1.19521713	2.2887573	2.30260022	3.163486	1.033741633
VKORC1	+	+	+	+		+			+		3.19230955	2.70831553	2.62164317	2.06430753	3.762924	2.482686996
VKORC1L1			+	+							1.00552828	1.25798861	3.59339592	2.76123174	4.043408	2.00313441
VMA21											0.16900842	-0.1890074	0.39742751	0.1872565	0.5250301	0.194526037
VMP1											0.18020414	-0.1313489	0.55964732	0.66140111	0.7541598	2.017450968
VPS35											0.08131601	-0.1345285	0.28115255	-0.00454267	0.3608845	0.017545064
VPS4A											0.71665144	0.703029	0.87621448	0.68675232	0.4923247	0.412409465
VPS52											0.1291725	-0.3848159	0.81982251	0.16139603	0.1081601	-0.502554576
VTA1											0.82362058	0.89393616	3.22101261	1.62488174	0.0194102	-0.556308746
WARS											1.03804595	1.20269267	1.81214317	0.44844755	0.1631248	-0.100648244
WDR1											0.16293482	-0.0856533	5.53E-05	-0.79793421	0.0002044	-0.762400945
WDR5											0.13124489	-0.280817	0.36558724	0.11969121	0.0103086	-1.684065501
WLS											0.11312496	-0.435627	2.87075609	0.87062645	0.5674563	0.469097773
XPNPEP1											0.95662805	0.62278493	0.05892353	-0.43125025	0.0753143	-0.108414968
XPO1											1.11560585	0.33523941	2.37296011	0.1040465	2.5127341	0.22620519
XPO5											2.81131795	0.85907618	0.79851628	0.31886164	2.2202374	0.666456223
XPO7				+							1.09017901	0.57342148	1.49038294	0.40943146	3.3822005	2.60066096
XPOT											0.5009915	0.11875661	0.00339563	-0.2184035	1.1366908	0.235773722
XRCC5											0.10430389	-0.1351681	0.01476088	-0.17265447	0.5176033	0.068682988
XRCC6											1.40570624	0.15520287	1.96937501	0.1782697	0.9857003	0.077960332
XXYLT1	+		+								2.20620544	1.40996297	1.64231977	2.25876999	0.225312	-0.186327616
YARS	+										1.36025289	0.56477038	1.69233029	0.32222366	1.7542135	0.397497813
YBX1											0.07393359	-0.9540666	0.00366839	-1.45900599	0.035799	-0.59185346
YES1	+	+	+	+							4.40717536	6.64455605	4.34789986	7.05010859	4.3367133	6.829088847
YIPF3			+								1.4853975	0.86648051	4.2116836	1.78912163	0.0071188	-1.376675924
YIPF6											0.15930077	-0.2075462	1.31181635	0.71851985	0.0123455	-1.259684881
YKT6											0.26250896	-0.0554473	0.05855326	-1.16495832	0.6446509	0.2568175
YRDC											0.91835433	0.43053118	0.63978186	0.28282102	0.0138967	-0.763163249
YTHDF2			+								0.83274711	0.6843249	3.38192056	1.86021678	0.5966325	0.498515447
YWHAB											3.94631818	1.35056114	2.71894341	0.90233167	0.4008332	0.041979472
YWHAE											2.32711482	0.85288048	4.10080806	0.77056503	3.2214799	0.550562541
YWHAG											0.99111039	0.28512446	1.3450636	0.30808385	0.7532989	0.151379903
YWHAH	+										1.66844204	0.99663862	0.0244424	-0.48553212	0.0387245	-0.93731753
YWHAQ											2.40828969	0.94200134	2.67539308	1.06618182	0.986311	0.208713531
YWHAZ											3.07385281	0.91790899	2.80090289	1.02697309	3.0617123	0.523761749
ZDHHC13	+	+	+	+							5.81783792	5.92924627	2.05842339	2.59063848	4.2309641	4.640251795
ZDHHC14			+								0.59911034	0.21059545	2.86655649	3.00870641	1.0854496	0.802103678
ZDHHC17		+	+	+							4.17860954	2.88801893	3.37908676	2.65140088	3.0973324	2.248273214
ZDHHC18		+	+	+							3.52589861	3.72319349	3.608381	2.08722115	3.4350255	3.581525803
ZDHHC20		+	+	+							3.61422136	2.61089071	3.82600106	3.23570061	6.2467144	5.146009445
ZDHHC20											0.56608413	0.39803251	0.04652338	-0.53382874	0.4675245	0.428263982
ZDHHC21	+										0.87999073	0.67848714	0.1049397	-0.27279218	0.6770757	0.565515518
ZDHHC24	+										1.59710961	1.37611326	0.86802131	1.2228349	0.0064689	-1.689606984
ZDHHC3		+	+	+							3.98177186	4.3988355	3.2348437	3.34277471	3.151026	3.215759913
ZDHHC4											0.68833417	1.35602125	0.04092996	-0.65842756	0.2451264	-0.186623255
ZDHHC5		+	+	+							3.50305547	2.74714979	4.85927611	4.1748689	3.0663026	3.560382843
ZDHHC6	+	+	+	+					+		3.1073798	5.02516047	3.4979708	5.2048289	3.7423023	3.927830378
ZDHHC7			+	+							0.31227447	0.02375984	3.61940797	2.97741572	2.6271011	3.917298635
ZMPSTE24	+		+								0.6847226	1.03406207	2.17173344	2.75695483	1.8655288	1.623500824

ZNF207	+										0.07804358	-0.7407265	2.49312181	1.565087	0.0990177	-0.381534576
ZNRF1	+	+	+	+							3.068477	3.1166083	2.94332403	3.66347186	4.4544787	5.84520785
ZNRF2	+	+	+	+							5.38764804	5.98996162	4.22904943	6.18304189	4.1412066	6.257630666
ZZEF1	+		+	+							2.0586167	1.58549118	4.02980033	3.51294708	3.4517731	3.798852921

Table S6 : Identification of PTM peptides in zebrafish embryos with PEAKS

Sheet 1 : Summary of all detected PTM peptides
The table shows all proteins (gene names) for which a PTM peptide was detected as well as the number of appearences within a triplicate per experiment.

Sheets 2-3: Total number of PTM peptide sequences found in 72h long YnMyr pulse (2) and 24h long mixed YnMyr pulse (3) experiments

A complete list of PTM peptides found in all replicates and their characterists, e.g. probablity score (-10LogP), mass, retention time.
PTM peptides are ordered by their sequences.

In red - PTM peptides assigned as false positive (non-MG)
The false discovery rate (#non-MG PTM peptides/#MG PTM peptides + #non-MG PTM peptides) is **1%**.

	All proteins with PTM peptide		PTM peptide seen within triplicate	
#	Gene name	Accesion	72h pulse	24h mix
1	anxa13	tr|F1REH8|F1REH8_DANRE		3
2	anxa13l	tr|A8E7L1|A8E7L1_DANRE		1
3	arf1	tr|Q803D1|Q803D1_DANRE		3
4	arf1l	tr|Q7ZUZ7|Q7ZUZ7_DANRE		3
5	arf2	tr|Q6NYD8|Q6NYD8_DANRE	2	3
6	arf3a	tr|Q6DHP3|Q6DHP3_DANRE		2
7	arf4a	tr|Q6DC20|Q6DC20_DANRE		2
8	arf5	tr|Q7ZUW4|Q7ZUW4_DANRE	1	3
9	chchd3	tr|Q5RH04|Q5RH04_DANRE	1	1
10	chmp6b	tr|F8W3M3|F8W3M3_DANRE		1
11	DKEY-238C7.11	tr|Q6P5M5|Q6P5M5_DANRE		1
12	fam129aa	tr|Q5RG84|Q5RG84_DANRE	1	1
13	fam129ab	tr|E7FC82|E7FC82_DANRE	2	
14	fam129ba	tr|A1L1T1|A1L1T1_DANRE	2	2
15	fam129bb	tr|F1R7A2|F1R7A2_DANRE	1	3
16	flot2a	Q98TZ8|FLOT2_DANRE	2	3
17	flot2b	tr|Q6PFT0|Q6PFT0_DANRE	1	
18	fmnl2b	tr|E7F0M4|E7F0M4_DANRE	2	2
19	fmnl3	tr|E9QG32|E9QG32_DANRE	2	2
20	fynb, fyna	F1RDG9|FYNB_DANRE, tr|F1QHX3|F1QHX3_DANRE	2	1
21	gnai1, gnai3	tr|Q7T3D3|Q7T3D3_DANRE, tr|A9JTC8|A9JTC8_DANRE	3	3
22	gnai2a	tr|Q6TNT8|Q6TNT8_DANRE	3	3
23	gnai2b	tr|X1WDQ5|X1WDQ5_DANRE	2	2
24	gnao1a, gnao1b	tr|Q6PBP1|Q6PBP1_DANRE, tr|Q6P967|Q6P967_DANRE	3	3
25	gnat1	tr|Q90WX6|Q90WX6_DANRE		2
26	gnat2	tr|Q90WX5|Q90WX5_DANRE		3
27	gorasp1	tr|B0S774|B0S774_DANRE	2	1
28	gorasp2	tr|F1RBF9|F1RBF9_DANRE	3	3
29	greb1	tr|F1QAG4|F1QAG4_DANRE		2
30	lamtor1	tr|E7FBZ6|E7FBZ6_DANRE	1	
31	LOC100536777	tr|E7F846|E7F846_DANRE	1	
32	loh12cr1	Q5XIX8|L12R1_DANRE	1	
33	lrrc57	tr|E9QEY4|E9QEY4_DANRE		1
34	marcksa	tr|F1Q7Q6|F1Q7Q6_DANRE	1	1
35	marcksl1a	tr|Q6PD99|Q6PD99_DANRE		1
36	ndufb7	tr|Q6P6E5|Q6P6E5_DANRE	2	3
37	npas3	tr|E7FEE2|E7FEE2_DANRE	1	
38	pald1	Q803E0|PALD_DANRE	2	3
39	pcmtd1	tr|F1QAE7|F1QAE7_DANRE	1	3
40	polr2a	tr|F1Q9K4|F1Q9K4_DANRE	1	1
41	ppm1aa, ppm1bb	tr|Q6NYP6|Q6NYP6_DANRE, tr|Q5U386|Q5U386_DANRE	3	3
42	ppm1g	tr|F1QJE5|F1QJE5_DANRE	3	2
43	ppp3r1a, ppp3r1b	tr|E9QG20|E9QG20_DANRE, tr|Q66HZ0|Q66HZ0_DANRE	2	3
44	prkacaa	tr|A3KMS9|A3KMS9_DANRE	2	3
45	prkacab	tr|Q6DBV8|Q6DBV8_DANRE	2	3
46	prkacbb	tr|Q3ZB92|Q3ZB92_DANRE	2	3

47	psmc1a	tr|A8KC30|A8KC30_DANRE	3	3
48	psmc1b	tr|Q6IQ72|Q6IQ72_DANRE	3	3
49	rapsn	tr|Q8QGW4|Q8QGW4_DANRE	2	
50	rnf141	tr|A3KQA9|A3KQA9_DANRE	2	3
51	si:dkeyp-67a8.4	tr|F1QL32|F1QL32_DANRE		2
52	slc44a1a	tr|F1Q9M4|F1Q9M4_DANRE	2	3
53	stk16	tr|A2CEW6|A2CEW6_DANRE	1	
54	tldc1	Q1LWV7|TLDC1_DANRE	1	
55	uqcrq	tr|F1QEJ5|F1QEJ5_DANRE	1	
56	zgc:73075	tr|Q1LUD3|Q1LUD3_DANRE		3

72 h YnMyr pulse

Replicate	Sequence	Accesion	-10LogP	Mass	ppm	m/z	RT	Scan
1	G(+463.29)AEQSGDADHK	Q5XIX8|L12R1_DANRE	44.66	1576.759	0.2	526.5939	39.53	11494
1	G(+463.29)AFLDKPK	tr|Q6NYP6|Q6NYP6_DANRE, tr|Q5U386|Q5U386_DANRE	46.46	1337.782	0.2	446.9346	62.71	19539
2	G(+463.29)AFLDKPK	tr|Q6NYP6|Q6NYP6_DANRE, tr|Q5U386|Q5U386_DANRE	47.39	1337.782	0.2	446.9347	62.72	18873
3	G(+463.29)AFLDKPK	tr|Q6NYP6|Q6NYP6_DANRE, tr|Q5U386|Q5U386_DANRE	45.98	1337.782	0	446.9346	62.71	19501
1	G(+463.29)AHLVR	tr|Q6P6E5|Q6P6E5_DANRE	37.11	1114.672	-0.9	372.5644	49.29	14891
3	G(+463.29)AHLVR	tr|Q6P6E5|Q6P6E5_DANRE	30.19	1114.672	0.7	372.565	49.17	14813
3	G(+463.29)ANNSTR	tr|Q5RH04|Q5RH04_DANRE	41.84	1181.627	-1.1	394.8823	45.18	13388
3	G(+463.29)AQFTK	tr|F1Q7Q6|F1Q7Q6_DANRE	30.2	1113.63	0.1	557.8221	58.85	18170
3	G(+463.29)ASSSLLDETQSNYIK	tr|Q5RG84|Q5RG84_DANRE	33.41	2175.117	-0.4	726.046	69.18	21751
1	G(+463.29)ASSSSLLDESK	tr|E7FC82|E7FC82_DANRE	57.72	1642.853	1.1	548.6254	62.49	19465
3	G(+463.29)ASSSSLLDESK	tr|E7FC82|E7FC82_DANRE	53.8	1642.853	0.3	548.6249	62.49	19425
1	G(+463.29)AYLSQPN(+.98)TEK	tr|F1QJE5|F1QJE5_DANRE	41.37	1670.863	0.3	557.9617	69.18	21813
3	G(+463.29)AYLSQPN(+.98)TEK	tr|F1QJE5|F1QJE5_DANRE	39.21	1670.863	0.7	557.9619	67.68	21233
1	G(+463.29)AYLSQPNTEK	tr|F1QJE5|F1QJE5_DANRE	63.06	1669.879	0.3	557.6337	65.87	20643
2	G(+463.29)AYLSQPNTEK	tr|F1QJE5|F1QJE5_DANRE	63.68	1669.879	0	557.6335	66	19989
3	G(+463.29)AYLSQPNTEK	tr|F1QJE5|F1QJE5_DANRE	58.26	1669.879	0.3	557.6337	65.81	20574
3	G(+463.29)C(+57.02)C(+57.02)FSSDSETTDPDGDEVKPLIPDPNQER	tr|E7FBZ6|E7FBZ6_DANRE	56.06	3727.673	-1.1	932.9244	65.41	20436
1	G(+463.29)C(+57.02)C(+57.02)GSTQK	tr|F1Q9M4|F1Q9M4_DANRE	38.36	1359.639	-1.8	454.2194	45.39	13487
3	G(+463.29)C(+57.02)C(+57.02)GSTQK	tr|F1Q9M4|F1Q9M4_DANRE	34.04	1359.639	-1.1	454.2197	45.45	13474
1	G(+463.29)C(+57.02)C(+57.02)LTVGPNEALVVSGAC(+57.02)C(+57.02)GSDAK	tr|Q6PFT0|Q6PFT0_DANRE	55.75	2944.367	1.6	982.4644	71.53	22612
1	G(+463.29)C(+57.02)TLSAEER	tr|Q6PBP1|Q6PBP1_DANRE, tr|Q6P967|Q6P967_DANRE	58.57	1484.741	-0.4	495.9206	58.97	18243
2	G(+463.29)C(+57.02)TLSAEER	tr|Q6PBP1|Q6PBP1_DANRE, tr|Q6P967|Q6P967_DANRE	55.47	1484.741	-1.1	495.9203	58.83	17556
3	G(+463.29)C(+57.02)TLSAEER	tr|Q6PBP1|Q6PBP1_DANRE, tr|Q6P967|Q6P967_DANRE	44.96	1484.741	-0.2	495.9207	58.95	18205
1	G(+463.29)C(+57.02)TLSTEDK	tr|Q7T3D3|Q7T3D3_DANRE, tr|A9JTC8|A9JTC8_DANRE	54.24	1472.729	-1.3	737.371	57.94	17885
2	G(+463.29)C(+57.02)TLSTEDK	tr|Q7T3D3|Q7T3D3_DANRE, tr|A9JTC8|A9JTC8_DANRE	50.03	1472.729	0.5	491.9173	58.08	17304
3	G(+463.29)C(+57.02)TLSTEDK	tr|Q7T3D3|Q7T3D3_DANRE, tr|A9JTC8|A9JTC8_DANRE	52.15	1472.729	0.2	491.9172	58.23	17955
1	G(+463.29)C(+57.02)TVSAEDK	tr|X1WDQ5|X1WDQ5_DANRE	47.76	1428.703	1.4	477.2423	53.73	16417
3	G(+463.29)C(+57.02)TVSAEDK	tr|X1WDQ5|X1WDQ5_DANRE	40.75	1428.703	0.2	477.2417	53.75	16399
1	G(+463.29)C(+57.02)TVSQEDK	tr|Q6TNT8|Q6TNT8_DANRE	59.33	1485.725	-0.9	496.2484	52.7	16065
2	G(+463.29)C(+57.02)TVSQEDK	tr|Q6TNT8|Q6TNT8_DANRE	39.69	1485.725	-0.2	496.2487	52.5	15418
3	G(+463.29)C(+57.02)TVSQEDK	tr|Q6TNT8|Q6TNT8_DANRE	56.52	1485.725	0.9	496.2493	52.47	15951
1	G(+463.29)C(+57.02)VQC(+57.02)K	F1RDG9|FYNB_DANRE, tr|F1QHX3|F1QHX3_DANRE	33.89	1213.606	0	607.8102	51.68	15704
3	G(+463.29)C(+57.02)VQC(+57.02)K	F1RDG9|FYNB_DANRE, tr|F1QHX3|F1QHX3_DANRE	38.13	1213.606	0	607.8102	51.66	15663
3	G(+463.29)DLISTHLDESKR	tr|F1R7A2|F1R7A2_DANRE	58.27	1933.038	0.9	484.2672	59.23	18304
3	G(+463.29)DVISTNLDDAK	tr|A1L1T1|A1L1T1_DANRE	41.78	1709.895	2.5	570.9736	72.13	22753
1	G(+463.29)DVISTNLDDAKR	tr|A1L1T1|A1L1T1_DANRE	32.06	1865.996	-0.3	623.0057	62.47	19459
1	G(+463.29)GAVSAGEDNDDLIDNLK	tr|F1QAE7|F1QAE7_DANRE	42.87	2265.124	1.3	756.0494	72.57	22972
1	G(+463.29)GAVSAGEDNDELIDNLK	tr|E7F846|E7F846_DANRE	49.24	2279.139	-3	760.7181	71.23	22507
1	G(+463.29)GSQSVEIPGGGSEGYHVLR	tr|F1RBF9|F1RBF9_DANRE	78.81	2448.251	0.3	613.0702	56.99	17552
2	G(+463.29)GSQSVEIPGGGSEGYHVLR	tr|F1RBF9|F1RBF9_DANRE	75.26	2448.251	-0.3	613.0698	57.15	16988
3	G(+463.29)GSQSVEIPGGGSEGYHVLR	tr|F1RBF9|F1RBF9_DANRE	82.25	2448.251	-0.5	613.0697	57.11	17565
3	G(+463.29)LHFGNLAK	tr|F1QEJ5|F1QEJ5_DANRE	31.2	1418.815	-0.4	473.9453	62.12	19292
3	G(+463.29)LTISSLFGR	tr|Q7ZUW4|Q7ZUW4_DANRE	43.68	1512.878	0.3	505.3	91.41	28513
1	G(+463.29)LTQSSGDGPEGGTEGYHVHGVQEDSPAER	tr|B0S774|B0S774_DANRE	45.13	3515.63	-0.8	879.9141	48.02	14440
3	G(+463.29)LTQSSGDGPEGGTEGYHVHGVQEDSPAER	tr|B0S774|B0S774_DANRE	61.64	3515.63	-1	879.9139	48.21	14467
3	G(+463.29)N(+.98)GDSVVAQK	Q1LWV7|TLDC1_DANRE	36.09	1437.758	-0.4	480.2596	56.05	17213

1	G(+463.29)NAATAK	tr|Q3ZB92|Q3ZB92_DANRE	35.19	1094.62	0.2	548.3172	47.33	14191
3	G(+463.29)NAATAK	tr|Q3ZB92|Q3ZB92_DANRE	34.1	1094.62	0.4	548.3173	47.53	14221
1	G(+463.29)NAESMDAQLTDFR	tr|E7F0M4|E7F0M4_DANRE	63.67	2016.969	-0.6	673.3298	70.82	22367
3	G(+463.29)NAESMDAQLTDFR	tr|E7F0M4|E7F0M4_DANRE	57.68	2016.969	-0.4	673.3299	70.58	22220
1	G(+463.29)NAPTAK	tr|A3KMS9|A3KMS9_DANRE	32.91	1120.635	0	561.3249	47.72	14334
3	G(+463.29)NAPTAK	tr|A3KMS9|A3KMS9_DANRE	34.92	1120.635	0.2	561.325	47.86	14339
1	G(+463.29)NC(+57.02)YTVGPNEALVVSGGC(+57.02)C(+57.02)GSDGK	Q98TZ8|FLOT2_DANRE	79.26	2920.327	1	974.4505	65.78	20610
3	G(+463.29)NC(+57.02)YTVGPNEALVVSGGC(+57.02)C(+57.02)GSDGK	Q98TZ8|FLOT2_DANRE	89.83	2920.327	1.5	974.4509	65.61	20505
2	G(+463.29)NEASYPLEM(+15.99)C(+57.02)SHFDADEIKR	tr|E9QG20|E9QG20_DANRE, tr|Q66HZ0|Q66HZ0_DANRE	57.63	2947.359	0.1	737.8472	54.32	16028
3	G(+463.29)NEASYPLEM(+15.99)C(+57.02)SHFDADEIKR	tr|E9QG20|E9QG20_DANRE, tr|Q66HZ0|Q66HZ0_DANRE	57.93	2947.359	-0.8	737.8464	54.22	16566
2	G(+463.29)NEASYPLEMC(+57.02)SHFDADEIK	tr|E9QG20|E9QG20_DANRE, tr|Q66HZ0|Q66HZ0_DANRE	35.7	2775.263	0.2	694.8232	65.91	19956
2	G(+463.29)NEASYPLEMC(+57.02)SHFDADEIKR	tr|E9QG20|E9QG20_DANRE, tr|Q66HZ0|Q66HZ0_DANRE	75.71	2931.364	0.4	733.8486	59.41	17758
3	G(+463.29)NEASYPLEMC(+57.02)SHFDADEIKR	tr|E9QG20|E9QG20_DANRE, tr|Q66HZ0|Q66HZ0_DANRE	77.41	2931.364	-0.4	733.848	59.18	18287
1	G(+463.29)NIESVDGQSEM(+15.99)K	tr|E9QG32|E9QG32_DANRE	46.34	1871.905	0.7	624.976	60	18603
3	G(+463.29)NIESVDGQSEM(+15.99)K	tr|E9QG32|E9QG32_DANRE	32.33	1871.905	0.5	624.9758	60.14	18620
1	G(+463.29)NLM(+15.99)GK	tr|F1Q9K4|F1Q9K4_DANRE	40.77	1097.602	-2.8	549.8065	90.06	28502
1	G(+463.29)NM(+15.99)FAGLFK	tr|Q6NYD8|Q6NYD8_DANRE	38.98	1462.776	0.1	488.5992	78.89	25095
3	G(+463.29)NM(+15.99)FAGLFK	tr|Q6NYD8|Q6NYD8_DANRE	42.81	1462.776	0.3	488.5992	78.82	24944
3	G(+463.29)NMFAGLFK	tr|Q6NYD8|Q6NYD8_DANRE	56.81	1446.781	0.5	483.2677	90.47	28260
1	G(+463.29)NTPAAK	tr|Q6DBV8|Q6DBV8_DANRE	31.72	1120.635	0.3	561.3251	47.45	14232
3	G(+463.29)NTPAAK	tr|Q6DBV8|Q6DBV8_DANRE	41.12	1120.635	0.2	561.325	47.61	14250
3	G(+463.29)QALC(+57.02)IC(+57.02)SR	tr|A2CEW6|A2CEW6_DANRE	49.22	1526.781	0.8	509.9347	66.97	20984
3	G(+463.29)QC(+57.02)VTK	tr|E7FEE2|E7FEE2_DANRE	33.73	1154.623	0.3	578.319	50.82	15370
1	G(+463.29)QDQTK	tr|Q8QGW4|Q8QGW4_DANRE	35.31	1138.61	-1.2	570.3113	45.5	13527
3	G(+463.29)QDQTK	tr|Q8QGW4|Q8QGW4_DANRE	32	1138.61	0.6	570.3124	45.46	13481
1	G(+463.29)QQLSGQAVTR	tr|A3KQA9|A3KQA9_DANRE	57.1	1606.89	-1.6	536.6365	56.65	17441
3	G(+463.29)QQLSGQAVTR	tr|A3KQA9|A3KQA9_DANRE	53.08	1606.89	0	536.6374	56.62	17409
2	G(+463.29)QSQSGGHGPGGGK	tr|Q6IQ72|Q6IQ72_DANRE	58.52	1672.839	0.4	558.6206	33.67	9046
3	G(+463.29)QSQSGGHGPGGGK	tr|Q6IQ72|Q6IQ72_DANRE	67.79	1672.839	0.4	558.6206	33.51	9356
1	G(+463.29)QSQSGGHGPGGGKK	tr|Q6IQ72|Q6IQ72_DANRE	41.19	1800.934	-1	451.2404	25.69	6567
3	G(+463.29)QSQSGGHGPGGGKK	tr|Q6IQ72|Q6IQ72_DANRE	35.93	1800.934	-0.6	451.2406	26.21	6755
1	G(+463.29)QSQSGGQGPGGGK	tr|A8KC30|A8KC30_DANRE	66.53	1663.839	-0.3	555.6201	43.26	12802
2	G(+463.29)QSQSGGQGPGGGK	tr|A8KC30|A8KC30_DANRE	50.07	1663.839	0.1	555.6203	43.69	12476
3	G(+463.29)QSQSGGQGPGGGK	tr|A8KC30|A8KC30_DANRE	73.87	1663.839	0.4	555.6205	43.31	12775
1	G(+463.29)QSQSGGQGPGGGKK	tr|A8KC30|A8KC30_DANRE	48.57	1791.934	2	598.3198	32.84	9147
3	G(+463.29)QSQSGGQGPGGGKK	tr|A8KC30|A8KC30_DANRE	62.16	1791.934	1.1	598.3193	33.2	9240
1	G(+463.29)TTASAAPQATLHER	Q803E0|PALD_DANRE	61.11	1973.044	-0.8	658.6882	46.78	13994
3	G(+463.29)TTASAAPQATLHER	Q803E0|PALD_DANRE	64.17	1973.044	0.1	658.6887	46.71	13926
1	Q(+463.29)QITK	tr|F1RAC4|F1RAC4_DANRE	31.37	1079.645	0.3	540.83	56.73	17470

24 h mixed YnMyr pulses

Replicate	Sequence	Accesion	-10LogP	Mass	ppm	m/z	RT	Scan
1	A(+463.29)ISGLFTR	tr|E7FD71|E7FD71_DANRE	27.01	1326.777	-0.8	443.266	57.73	17875
1	G(+463.29)AFLDKPK	tr|Q6NYP6|Q6NYP6_DANRE, tr|Q5U386|Q5U386_DA	40.65	1337.782	0.5	446.9348	84.06	26435
2	G(+463.29)AFLDKPK	tr|Q6NYP6|Q6NYP6_DANRE, tr|Q5U386|Q5U386_DA	43.35	1337.782	0.6	446.9348	84.08	26352
3	G(+463.29)AFLDKPK	tr|Q6NYP6|Q6NYP6_DANRE, tr|Q5U386|Q5U386_DA	35.66	1337.782	1.2	446.9351	84.16	26172
1	G(+463.29)AGASAEEK	tr|Q90WX6|Q90WX6_DANRE	30.73	1281.668	0.3	428.23	67	20843
2	G(+463.29)AGASAEEK	tr|Q90WX6|Q90WX6_DANRE	40.09	1281.668	0.4	428.23	66.98	20715
1	G(+463.29)AHLVR	tr|Q6P6E5|Q6P6E5_DANRE	29.55	1114.672	-0.2	372.5647	67.79	21118
2	G(+463.29)AHLVR	tr|Q6P6E5|Q6P6E5_DANRE	33.93	1114.672	-1.1	372.5643	67.41	20868
3	G(+463.29)AHLVR	tr|Q6P6E5|Q6P6E5_DANRE	30.79	1114.672	0.4	372.5649	67.47	20777
1	G(+463.29)ANNSTR	tr|Q5RH04|Q5RH04_DANRE	39.5	1181.627	0.1	394.8828	63.41	19630
3	G(+463.29)AQFTK	tr|F1Q7Q6|F1Q7Q6_DANRE	26.94	1113.63	-1.6	557.8212	80.07	24836
3	G(+463.29)AQLTK	tr|Q6PD99|Q6PD99_DANRE	25.82	1079.645	-0.4	540.8297	77.91	24133
2	G(+463.29)ASESLQEK	tr|F1QL32|F1QL32_DANRE	37.24	1410.747	-0.9	471.2557	73.63	22908
3	G(+463.29)ASESLQEK	tr|F1QL32|F1QL32_DANRE	29.51	1410.747	0.4	471.2563	73.68	22772
2	G(+463.29)ASSSLLDETQSNYIK	tr|Q5RG84|Q5RG84_DANRE	39.65	2175.117	-1	726.0456	93.4	29365
1	G(+463.29)AYLSQPNTEK	tr|F1QJE5|F1QJE5_DANRE	55.18	1669.879	0.1	557.6336	88.95	28046
2	G(+463.29)AYLSQPNTEK	tr|F1QJE5|F1QJE5_DANRE	65.06	1669.879	0	557.6335	88.9	27935
1	G(+463.29)C(+57.02)C(+57.02)GSTQK	tr|F1Q9M4|F1Q9M4_DANRE	27.86	1359.639	-0.7	454.2199	63.82	19771
2	G(+463.29)C(+57.02)C(+57.02)GSTQK	tr|F1Q9M4|F1Q9M4_DANRE	33.11	1359.639	0.6	454.2205	63.84	19632
3	G(+463.29)C(+57.02)C(+57.02)GSTQK	tr|F1Q9M4|F1Q9M4_DANRE	26.93	1359.639	-0.3	454.2201	63.86	19570
1	G(+463.29)C(+57.02)TLSAEER	tr|Q6PBP1|Q6PBP1_DANRE, tr|Q6P967|Q6P967_DAN	51.7	1484.741	-1	495.9203	80.2	25163
2	G(+463.29)C(+57.02)TLSAEER	tr|Q6PBP1|Q6PBP1_DANRE, tr|Q6P967|Q6P967_DAN	44.65	1484.741	1.7	495.9217	80.55	25178
3	G(+463.29)C(+57.02)TLSAEER	tr|Q6PBP1|Q6PBP1_DANRE, tr|Q6P967|Q6P967_DAN	46.99	1484.741	-0.7	495.9205	80.25	24896
1	G(+463.29)C(+57.02)TLSTEDK	tr|Q7T3D3|Q7T3D3_DANRE, tr|A9JTC8|A9JTC8_DANF	40.24	1472.729	0.3	491.9172	79.43	24905
2	G(+463.29)C(+57.02)TLSTEDK	tr|Q7T3D3|Q7T3D3_DANRE, tr|A9JTC8|A9JTC8_DANF	44.26	1472.729	0.6	491.9174	79.42	24800
3	G(+463.29)C(+57.02)TLSTEDK	tr|Q7T3D3|Q7T3D3_DANRE, tr|A9JTC8|A9JTC8_DANF	29.13	1472.729	1.1	491.9176	79.47	24630
1	G(+463.29)C(+57.02)TVSAEDK	tr|X1WDQ5|X1WDQ5_DANRE	34.92	1428.703	0.7	477.242	74.11	23175
2	G(+463.29)C(+57.02)TVSAEDK	tr|X1WDQ5|X1WDQ5_DANRE	38.17	1428.703	-0.2	477.2416	74.09	23065
1	G(+463.29)C(+57.02)TVSQEDK	tr|Q6TNT8|Q6TNT8_DANRE	49.72	1485.725	2.3	496.2499	72.66	22688
2	G(+463.29)C(+57.02)TVSQEDK	tr|Q6TNT8|Q6TNT8_DANRE	45.39	1485.725	0.4	496.249	72.65	22569
3	G(+463.29)C(+57.02)TVSQEDK	tr|Q6TNT8|Q6TNT8_DANRE	42.71	1485.725	-0.2	496.2487	72.67	22426
2	G(+463.29)C(+57.02)VQC(+57.02)K	F1RDG9|FYNB_DANRE, tr|F1QHX3|F1QHX3_DANRE	32.03	1213.606	1.7	607.8113	71.47	22190
1	G(+463.29)DLISTHLDESKR	tr|F1R7A2|F1R7A2_DANRE	56.99	1933.038	-1.2	484.2662	80.05	25114
2	G(+463.29)DLISTHLDESKR	tr|F1R7A2|F1R7A2_DANRE	57.57	1933.038	-1.2	484.2662	79.97	24991
3	G(+463.29)DLISTHLDESKR	tr|F1R7A2|F1R7A2_DANRE	41.6	1933.038	-2.6	484.2655	80.01	24819
1	G(+463.29)DVISTNLDDAKR	tr|A1L1T1|A1L1T1_DANRE	56.76	1865.996	-0.2	623.0058	84.86	26702
3	G(+463.29)DVISTNLDDAKR	tr|A1L1T1|A1L1T1_DANRE	35.68	1865.996	-0.5	623.0056	84.89	26418
1	G(+463.29)GAVSAGEDNDDLIDNLK	tr|F1QAE7|F1QAE7_DANRE	40.36	2265.124	-0.3	756.0482	98.09	30879
2	G(+463.29)GAVSAGEDNDDLIDNLK	tr|F1QAE7|F1QAE7_DANRE	40.51	2265.124	-0.4	756.0482	97.99	30770
3	G(+463.29)GAVSAGEDNDDLIDNLK	tr|F1QAE7|F1QAE7_DANRE	39.21	2265.124	0.3	756.0487	98.1	30550
1	G(+463.29)GSQSVEIPGGGSEGYHVLR	tr|F1RBF9|F1RBF9_DANRE	66.66	2448.251	-0.5	613.0697	78.18	24488
2	G(+463.29)GSQSVEIPGGGSEGYHVLR	tr|F1RBF9|F1RBF9_DANRE	78.9	2448.251	0.9	613.0706	78.25	24420
3	G(+463.29)GSQSVEIPGGGSEGYHVLR	tr|F1RBF9|F1RBF9_DANRE	66.13	2448.251	-2	613.0688	78.13	24204
1	G(+463.29)LTISSLFGR	tr|Q7ZUW4|Q7ZUW4_DANRE	45.4	1512.878	-0.1	505.2998	104.25	32877
2	G(+463.29)LTISSLFGR	tr|Q7ZUW4|Q7ZUW4_DANRE	60.71	1512.878	-0.2	505.2997	104.26	32783
3	G(+463.29)LTISSLFGR	tr|Q7ZUW4|Q7ZUW4_DANRE	50.19	1512.878	-0.1	505.2998	104.24	32510

1	G(+463.29)LTISSLFSR	tr|Q6P5M5|Q6P5M5_DANRE	38.42	1542.888	1.4	515.3041	104.23	32871
1	G(+463.29)LTISSVFSR	tr|Q6DC20|Q6DC20_DANRE	56.12	1528.873	2	510.6325	103.93	32749
2	G(+463.29)LTISSVFSR	tr|Q6DC20|Q6DC20_DANRE	56.77	1528.873	1.5	510.6322	103.93	32656
1	G(+463.29)LTQSSGDGPEGGTEGYHVHGVQEDSPAER	tr|B0S774|B0S774_DANRE	50.29	3515.63	-4.7	879.9107	67.52	21027
1	G(+463.29)NAATAK	tr|Q3ZB92|Q3ZB92_DANRE	31.91	1094.62	-1.7	548.3162	65.98	20499
2	G(+463.29)NAATAK	tr|Q3ZB92|Q3ZB92_DANRE	29.31	1094.62	0.6	548.3174	66.02	20388
3	G(+463.29)NAATAK	tr|Q3ZB92|Q3ZB92_DANRE	30.27	1094.62	2.5	548.3185	66.05	20297
1	G(+463.29)NAESMDAQLTDFR	tr|E7F0M4|E7F0M4_DANRE	52.58	2016.969	-0.4	673.33	95.37	30060
2	G(+463.29)NAESMDAQLTDFR	tr|E7F0M4|E7F0M4_DANRE	38.74	2016.969	-0.9	673.3296	95.44	29995
1	G(+463.29)NAPTAK	tr|A3KMS9|A3KMS9_DANRE	27.23	1120.635	-1.9	374.5516	66.61	20711
2	G(+463.29)NAPTAK	tr|A3KMS9|A3KMS9_DANRE	34.74	1120.635	-0.7	561.3245	66.61	20588
3	G(+463.29)NAPTAK	tr|A3KMS9|A3KMS9_DANRE	32.87	1120.635	-0.5	561.3246	66.71	20517
1	G(+463.29)NAQSGGIPR	tr|Q1LUD3|Q1LUD3_DANRE	42.66	1418.774	0.7	473.9323	72.78	22728
2	G(+463.29)NAQSGGIPR	tr|Q1LUD3|Q1LUD3_DANRE	46.12	1418.774	0.1	473.932	72.79	22615
3	G(+463.29)NAQSGGIPR	tr|Q1LUD3|Q1LUD3_DANRE	44.21	1418.774	1.4	473.9326	72.78	22467
1	G(+463.29)NC(+57.02)QPTIVPYEDFDVIADIK	tr|F1REH8|F1REH8_DANRE	65.6	2756.384	1.1	919.803	103.83	32710
2	G(+463.29)NC(+57.02)QPTIVPYEDFDVIADIK	tr|F1REH8|F1REH8_DANRE	82.42	2756.384	-1	919.8011	103.83	32617
3	G(+463.29)NC(+57.02)QPTIVPYEDFDVIADIK	tr|F1REH8|F1REH8_DANRE	42.52	2756.384	1.7	919.8036	103.86	32369
1	G(+463.29)NC(+57.02)YTVGPNEALVVSGGC(+57.02)C(+57.02)	Q98TZ8|FLOT2_DANRE	72.11	2920.327	2.5	731.0908	89.68	28280
2	G(+463.29)NC(+57.02)YTVGPNEALVVSGGC(+57.02)C(+57.02)	Q98TZ8|FLOT2_DANRE	79.85	2920.327	0.8	731.0895	89.59	28162
3	G(+463.29)NC(+57.02)YTVGPNEALVVSGGC(+57.02)C(+57.02)	Q98TZ8|FLOT2_DANRE	59.56	2920.327	-1.5	731.0878	89.6	27953
2	G(+463.29)NEASYPLEM(+15.99)C(+57.02)SHFDADEIKR	tr|E9QG20|E9QG20_DANRE, tr|Q66HZ0|Q66HZ0_DAI	71.51	2947.359	0.3	737.8473	74.63	23241
3	G(+463.29)NEASYPLEM(+15.99)C(+57.02)SHFDADEIKR	tr|E9QG20|E9QG20_DANRE, tr|Q66HZ0|Q66HZ0_DAI	76.34	2947.359	-1.3	737.8461	74.57	23063
1	G(+463.29)NEASYPLEMC(+57.02)SHFDADEIKR	tr|E9QG20|E9QG20_DANRE, tr|Q66HZ0|Q66HZ0_DAI	69.12	2931.364	1.1	733.8492	80.61	25293
2	G(+463.29)NEASYPLEMC(+57.02)SHFDADEIKR	tr|E9QG20|E9QG20_DANRE, tr|Q66HZ0|Q66HZ0_DAI	79	2931.364	1.1	733.8491	80.68	25226
3	G(+463.29)NEASYPLEMC(+57.02)SHFDADEIKR	tr|E9QG20|E9QG20_DANRE, tr|Q66HZ0|Q66HZ0_DAI	76.16	2931.364	0.5	733.8487	80.5	24972
1	G(+463.29)NIESVDGQSEMK	tr|E9QG32|E9QG32_DANRE	31.98	1855.91	0.7	619.6443	85.56	26936
3	G(+463.29)NIESVDGQSEMK	tr|E9QG32|E9QG32_DANRE	33.1	1855.91	1.5	619.6448	85.59	26646
1	G(+463.29)NIFANLFK	tr|Q803D1|Q803D1_DANRE	42.54	1485.846	-0.1	496.2891	104.42	32943
2	G(+463.29)NIFANLFK	tr|Q803D1|Q803D1_DANRE	44.9	1485.846	-0.3	743.9299	104.39	32835
3	G(+463.29)NIFANLFK	tr|Q803D1|Q803D1_DANRE	46.7	1485.846	-0.6	743.9296	104.39	32571
1	G(+463.29)NIFGNLLK	tr|Q6DHP3|Q6DHP3_DANRE	44.23	1437.846	0.2	480.2892	104.1	32816
2	G(+463.29)NIFGNLLK	tr|Q6DHP3|Q6DHP3_DANRE	46.05	1437.846	-2.2	480.2881	104.09	32715
3	G(+463.29)NLFGK	tr|F8W3M3|F8W3M3_DANRE	25.58	1097.635	-0.8	549.8241	97.38	30338
3	G(+463.29)NLM(+15.99)GK	tr|F1Q9K4|F1Q9K4_DANRE	30.85	1097.602	-3.1	549.8063	104.63	32662
1	G(+463.29)NM(+15.99)FAGLFK	tr|Q6NYD8|Q6NYD8_DANRE	42.94	1462.776	-1.4	732.394	103.4	32550
2	G(+463.29)NM(+15.99)FAGLFK	tr|Q6NYD8|Q6NYD8_DANRE	43.59	1462.776	0.5	488.5993	103.33	32430
3	G(+463.29)NM(+15.99)FAGLFK	tr|Q6NYD8|Q6NYD8_DANRE	39.94	1462.776	0	488.5991	103.41	32198
1	G(+463.29)NMFAGLFK	tr|Q6NYD8|Q6NYD8_DANRE	46.71	1446.781	0.3	483.2676	104.17	32845
2	G(+463.29)NMFAGLFK	tr|Q6NYD8|Q6NYD8_DANRE	51.39	1446.781	-3.2	483.2659	104.14	32739
3	G(+463.29)NMFAGLFK	tr|Q6NYD8|Q6NYD8_DANRE	47.32	1446.781	-0.8	724.397	104.13	32473
3	G(+463.29)NSALK	tr|E9QEY4|E9QEY4_DANRE	27.13	1051.614	-0.3	526.814	72.88	22502
2	G(+463.29)NSYTGHLQSTR	tr|F1QAG4|F1QAG4_DANRE	60.09	1782.913	-2.1	595.3102	64.55	19883
3	G(+463.29)NSYTGHLQSTR	tr|F1QAG4|F1QAG4_DANRE	47.03	1782.913	1.4	595.3123	64.48	19782
1	G(+463.29)NTPAAK	tr|Q6DBV8|Q6DBV8_DANRE	28.45	1120.635	1	561.3254	66.27	20596
2	G(+463.29)NTPAAK	tr|Q6DBV8|Q6DBV8_DANRE	28.04	1120.635	-0.2	561.3248	66.27	20473
3	G(+463.29)NTPAAK	tr|Q6DBV8|Q6DBV8_DANRE	27.95	1120.635	0.4	561.3251	66.37	20407
1	G(+463.29)NVFANLFK	tr|Q7ZUZ7|Q7ZUZ7_DANRE	46.29	1471.83	0.2	736.9224	104.25	32878
2	G(+463.29)NVFANLFK	tr|Q7ZUZ7|Q7ZUZ7_DANRE	50.07	1471.83	0.2	491.6174	104.22	32770
3	G(+463.29)NVFANLFK	tr|Q7ZUZ7|Q7ZUZ7_DANRE	45.49	1471.83	0.7	491.6176	104.22	32504

2	G(+463.29)NVQPTITPFEDFDVVADIK	tr|A8E7L1|A8E7L1_DANRE	44.06	2667.391	-3	890.1349	104.05	32702
1	G(+463.29)QQLSGQAVTR	tr|A3KQA9|A3KQA9_DANRE	50.72	1606.89	1	536.6379	77.72	24337
2	G(+463.29)QQLSGQAVTR	tr|A3KQA9|A3KQA9_DANRE	46.27	1606.89	0.4	536.6376	77.67	24227
3	G(+463.29)QQLSGQAVTR	tr|A3KQA9|A3KQA9_DANRE	39.87	1606.89	2.9	536.639	77.71	24068
1	G(+463.29)QSQSGGHGPGGGKK	tr|Q6IQ72|Q6IQ72_DANRE	37.27	1800.934	-0.4	451.2407	37.95	10945
2	G(+463.29)QSQSGGHGPGGGKK	tr|Q6IQ72|Q6IQ72_DANRE	39.94	1800.934	-0.3	451.2407	37.98	10835
3	G(+463.29)QSQSGGHGPGGGKK	tr|Q6IQ72|Q6IQ72_DANRE	42.2	1800.934	0.5	451.2411	37.97	10833
1	G(+463.29)QSQSGGQGPGGGK	tr|A8KC30|A8KC30_DANRE	64.68	1663.839	-1.3	555.6196	61.58	19038
2	G(+463.29)QSQSGGQGPGGGK	tr|A8KC30|A8KC30_DANRE	60.18	1663.839	0.6	832.9273	61.58	18872
3	G(+463.29)QSQSGGQGPGGGK	tr|A8KC30|A8KC30_DANRE	58.12	1663.839	-0.4	555.6201	61.53	18807
1	G(+463.29)QSQSGGQGPGGGKK	tr|A8KC30|A8KC30_DANRE	47.83	1791.934	0.9	598.3192	47.91	14476
2	G(+463.29)QSQSGGQGPGGGKK	tr|A8KC30|A8KC30_DANRE	54.93	1791.934	1.3	598.3194	48.06	14358
3	G(+463.29)QSQSGGQGPGGGKK	tr|A8KC30|A8KC30_DANRE	41.59	1791.934	1.1	598.3193	48.12	14393
1	G(+463.29)SGASAEDK	tr|Q90WX5|Q90WX5_DANRE	40.52	1283.647	0.7	428.8899	65.59	20367
2	G(+463.29)SGASAEDK	tr|Q90WX5|Q90WX5_DANRE	43.42	1283.647	-0.7	642.8303	65.51	20214
1	G(+463.29)SGASAEDKEM(+15.99)AK	tr|Q90WX5|Q90WX5_DANRE	51.86	1758.857	-2.6	587.2914	52.8	16195
2	G(+463.29)SGASAEDKEM(+15.99)AK	tr|Q90WX5|Q90WX5_DANRE	62.87	1758.857	-1.1	587.2923	52.86	16024
3	G(+463.29)SGASAEDKEM(+15.99)AK	tr|Q90WX5|Q90WX5_DANRE	49.65	1758.857	-1.2	587.2922	52.83	16018
1	G(+463.29)SGASAEDKEM(+15.99)AKK	tr|Q90WX5|Q90WX5_DANRE	35.92	1886.952	-0.5	472.745	43.01	12747
3	G(+463.29)SGASAEDKEM(+15.99)AKK	tr|Q90WX5|Q90WX5_DANRE	31.62	1886.952	1.4	472.7459	43.14	12657
1	G(+463.29)SGASAEDKEMAK	tr|Q90WX5|Q90WX5_DANRE	60.41	1742.862	1	581.9619	58.04	17963
2	G(+463.29)SGASAEDKEMAK	tr|Q90WX5|Q90WX5_DANRE	68.44	1742.862	0.1	581.9614	58.02	17762
3	G(+463.29)SGASAEDKEMAK	tr|Q90WX5|Q90WX5_DANRE	56.12	1742.862	-1.4	581.9605	58.02	17751
1	G(+463.29)TTASAAPQATLHER	Q803E0|PALD_DANRE	43.96	1973.044	-0.5	658.6884	65.42	20309
2	G(+463.29)TTASAAPQATLHER	Q803E0|PALD_DANRE	44.35	1973.044	-0.3	658.6885	65.36	20162
3	G(+463.29)TTASAAPQATLHER	Q803E0|PALD_DANRE	39.94	1973.044	-0.6	658.6883	65.47	20105
3	M(+463.29)SLSAK	tr|Q7ZT21|Q7ZT21_DANRE	30.45	1098.622	0.9	367.2149	25.47	6631

Table S7: Label-free quantification of YnMyr-tagged proteins in zebrafish

Sheet 1 : Summary

The table is showing a total number of protein IDs, the corresponding number of MG proteins and their percentage based on the number of requested valid values (1 valid value in any YnMyr treated sample). In addition, significance (both total and MG proteins) as well as the number of proteins with PTM peptide is indicated.

Sheet 2 : Total protein IDs

Zebrafish embryos were pulsed for 24 h with 20 µM YnMyr (or Myr control) after 5, 48 and 96 hours post fertilisation. The embryos were then lysed, tagged proteins were captured with reagent **2**, enriched, digested with trypsin and analyzed by LC-MS/MS. The data were analyzed with MaxQuant (version 1.5.0.25) and Perseus (version 1.5.0.9). The data are filtered (1 valid value in any YnMyr treated samples) and are displayed without any additional processing (total protein IDs). The LFQ intensity is displayed for each replicate (N = 3) for the YnMyr and control samples. In addition PEP and molecular weight are shown for each protein ID. Colunm MG proteins indicates where the MG signature is present, column t-test significance shows proteins significantly enriched in the YnMyr experiment (modified t-test FDR = 0.01 and s0 = 2) and column PTM peptides indicates if such peptides were found for a given ID. The column t-test parameters gives the value for the standard t-test and t-test difference (difference between logarithmized label-free intensities (corresponds to fold change)). Protein grouping feature was enabled in MaxQuant, therefore when unique peptides could not be assigned to one unique protein they were assigned instead to a group of closely related proteins. Proteins are ordered by gene names.

<u>Note</u>: due to the database (zebrafish complete fasta file, Uniprot) redundancy, protein gnao1a appears as 2 entries from the reviewed and not-reviewed part of the proteome

# total proteins	# MG proteins	% MG proteins	# proteins t-test significant	# MG proteins t-test significant	# proteins with PTM peptide
574	134	23%	95	72	51

		Log2 LFQ intensity											t-test					
Gene names	Protein IDs	YnMyr_1	YnMyr_2	YnMyr_3	Myr_1	Myr_2	Myr_3	MG protein	t-test Significant	PTM pep	PEP	Mol. weight [kDa]	-Log t-test p value	t-test Difference				
aars	Q1LVL2;Q1	22.71611	22.2083	22.46396	22.41398	22.72242	22.34533				8.13E-08	106.94	0.2502096	-0.0311165				
aatkb	E7F2C8	20.58724	18.82076	26.3727	20.29424	20.54585	19.15635				0.003428	182.16	0.6451942	1.92808723				
acaa2	B0S5C5	26.68646	26.44973	26.25961	19.32665	24.11188	23.50652		+		3.70E-56	41.786	1.590087	4.15025012				
acaca	F1QH12;F6	29.17305	29.60929	29.47769	30.02472	30.23158	29.30628				0	265.91	0.0536303	-0.4341838				
acacb	F1QM37;F1	28.48528	29.25232	28.70859	29.56249	29.64274	28.42049				0	254.47	0.1070524	-0.3931764				
acadm	A2CG95	20.77435	20.13166	21.4767	20.79758	21.0336	21.2696				3.15E-08	46.351	0.1524565	-0.2393557				
aco2	F8W4M7	22.82582	23.50009	20.29902	20.01998	19.46836	21.37759				2.71E-10	85.589	1.0866357	1.91966756				
acsf2	F1R1C0;Q0	24.21941	23.71544	23.48247	20.81591	19.88074	20.53331		+		1.35E-20	67.573	3.4904591	3.39578692				
acta1b	Q6XNL8	21.15501	24.97705	18.37418	20.41978	19.58011	20.27816				1.31E-209	41.974	0.5968714	1.4093984				
actba;actbb;act	Q7ZVI7;Q7	28.26087	28.40418	27.98923	27.90173	27.96063	28.2122				9.29E-85	41.766	0.8551346	0.19323985				
actc1b;actc1a;a	Q9I8V1;Q6	30.95858	31.00558	30.6465	30.89742	30.88555	30.83989				1.53E-262	41.972	0.2895669	-0.0040677				
actn1	F1QVZ2;B8	20.11517	19.42095	20.71808	18.96983	20.03846	20.82559				1.03E-41	103.94	0.3761326	0.14010429				
actn3a	D1GJ56	26.23105	26.57226	25.98144	26.04654	26.29626	26.42482				4.09E-79	103.59	0.3102402	0.00571124				
actn3b	Q8AX99	22.81308	22.66036	22.42558	22.55983	22.63823	22.42044				3.09E-78	103.86	0.5934063	0.09350268				
adh5	Q6NXA6	19.86228	22.43903	22.21041	22.41664	20.87261	23.40795				1.04E-06	40.032	0.1384464	-0.7284902				
adprhl1	Q5XJB9	27.22628	19.89343	17.51276	19.62919	19.24263	20.71284				0.010033	39.2	0.523138	1.68260384				
ahcy	Q803T5	24.58365	24.4106	24.8292	24.46438	24.7239	24.57184				4.37E-29	47.964	0.3517863	0.02110863				
ahnak	F1R1J9;F1C	20.83435	19.52732	23.11658	20.08016	19.75283	20.74073				0.000861	607.11	0.673728	0.96817589				
ak1	Q68EH2	21.49527	21.67681	19.29476	18.98853	18.99763	20.83993				0.000494	21.442	0.8472576	1.21358172				
akap12b	F2Z4T4	24.18056	24.12139	24.5538	20.91356	19.95798	20.67944		+		7.30E-11	165.55	3.8390007	3.7682546				
akap8l	R4GEY4;R4	20.47976	19.00563	20.6082	19.06891	21.77708	20.48428				0.011733	16.719	0.1812336	-0.4122276				
aldh18a1	A4IGC8;E7I	24.98802	25.47568	25.35491	23.66666	24.94481	24.57097				4.14E-30	85.376	1.3148952	0.87872632				
aldh2.2;aldh2l;a	F2Z4R7;A5	20.71822	20.07114	21.4493	20.34339	20.59347	20.84675				0.00181	56.563	0.4326895	0.15168317				
aldh3a2b	E9QH31	20.72875	19.8768	20.76223	20.91972	21.15196	20.99546				0.007093	55.059	0.0291444	-0.5664514				
aldh9a1a;aldh9	Q7ZVB2;B0	22.77096	23.0158	22.45164	22.05497	22.68265	22.92671				1.50E-09	55.261	0.5477948	0.19135984				
aldoaa;aldoab	Q803Q7;Q	24.99624	24.71062	24.71392	25.13022	24.94891	25.17509				9.72E-23	39.74	0.0170101	-0.2778123				
aldob	Q8JH71;E9	22.59015	22.33625	22.21966	22.29415	22.49375	22.11019				1.30E-09	39.287	0.5061826	0.0826575				
ampd1;ampd3a	Q6P3G5;E7	22.00522	21.38411	21.14967	21.79102	21.45784	21.42212				0.00039	82.936	0.2530394	-0.0439955				
and1	E7F5V5	20.95844	20.38659	20.73579	19.5802	21.28411	22.10072				2.51E-08	58.082	0.1932171	-0.2947413				
and2	A9JRX1	22.42399	21.99931	19.30827	22.98447	22.22391	22.51085				3.87E-06	56.542	0.0591565	-1.3292179				
ank2b	A2BIB8;A2I	19.65958	18.92974	20.19063	18.72418	19.02442	19.33924				0.001216	34.197	0.9255057	0.56403542				
ankrd22	F1QM15	20.95458	18.45903	19.60635	19.82796	19.2451	19.55855	+			0.01066	21.88	0.3616451	0.12945112				
anp32a	E9QB42;Q7	22.92343	22.41287	23.08352	22.60406	22.43365	22.78075				1.80E-07	24.673	0.6730342	0.20045662				
anxa13	F1REH8	28.65595	28.89313	28.00142	20.9825	22.02408	21.22873	+	+	+	1.08E-210	35.249	4.4785318	7.10506185				
anxa13l	A8E7L1;A8	25.4674	25.1671	24.60235	21.75829	19.61147	22.16404	+	+	+	1.35E-31	29.211	2.3293372	3.90102005				
anxa1a	Q804H2;A2	23.33851	22.94367	22.75591	20.72451	22.79374	22.38769				4.13E-07	37.754	1.0300244	1.04405085				
anxa2a	Q6P603	21.35361	19.71106	21.52416	19.79429	21.29745	20.51772				0.000337	38.138	0.4717625	0.32645607				
anxa4	Q804G7	20.16591	20.06799	21.70696	18.32584	19.08378	20.80214				9.85E-05	35.634	0.91833	1.24302928				
ap2b1;ap1b1	E9QC84;A5	21.19574	21.24551	21.62721	21.51326	21.809	21.42859				0.001001	67.651	0.0633875	-0.2274647				
apoa1	O42363	23.74294	23.06892	24.6084	22.98716	23.06298	25.19186				2.23E-12	30.256	0.3244249	0.0594209				
apoa1b	E7FES0	25.47113	25.98126	26.30588	25.0857	25.17287	27.04396				6.19E-37	30.139	0.379361	0.1519146				
apobb	Q5TZ29;F1	26.60045	26.4352	26.01434	26.17062	26.20494	25.90663	+			1.35E-102	412.59	0.8755312	0.2559363				
arf1	Q803D1	25.73107	26.93506	26.30266	20.69002	20.96279	19.91277	+	+	+	4.17E-256	20.611	3.9113416	5.80106926				
arf1l	Q7ZUZ7;E9	26.29801	27.64065	25.9476	18.21086	20.92937	20.61815	+	+	+	6.00E-276	20.608	2.8879902	6.70929337				
arf2	Q6NYD8;F8	29.039	28.75627	28.64712	21.71179	20.2931	19.46202	+	+	+	7.95E-285	20.599	3.9260618	8.3251578				
arf3b	Q78AP9	19.66778	26.41101	20.03324	19.29763	20.6732	20.91787	+			5.55E-211	20.601	0.6183229	1.74111176				

arf4a	Q6DC20;E7	26.88413	27.41965	26.21106	21.33716	19.15481	20.03636	+	+	+	7.85E-186	20.477	3.4120815	6.66217105				
arf5	Q7ZUW4	30.39151	30.54628	30.35741	19.34021	21.23187	20.55314	+	+	+	4.79E-247	20.342	4.5601738	10.0566635				
arf6b	Q6P2U5	24.83089	24.78701	24.57346	18.47036	20.06138	20.28913	+	+		1.32E-11	20.128	3.3505988	5.12349574				
arl1	Q6DGK1	28.0392	28.65526	27.92858	20.79983	20.30972	21.26801	+	+		5.52E-96	20.323	4.7980729	7.4151624				
arl8;arl5c;arl5a	Q6ZM32;Q	20.05517	20.18411	23.90903	20.62262	21.78954	20.57413	+			0.008445	20.354	0.4064296	0.38734436				
atic	A2CEW4	23.34164	23.54439	20.35402	19.82033	20.08662	20.35977				7.28E-05	64.056	1.3479832	2.32444445				
atp1a1	Q9DGL6	24.79273	24.49978	24.42573	24.30257	24.6198	24.60224	+			3.23E-50	113.38	0.4601367	0.06454213				
atp1a1b	B0R068	22.67193	22.94833	22.76947	22.53979	22.72761	22.49497	+			1.33E-43	112.74	1.2059581	0.20912488				
atp1a3a;atp1a3	Q6P271;F8	26.0968	25.75213	25.8574	25.7318	25.54613	25.85117	+			7.19E-53	112.52	0.9435876	0.19241079				
atp2a1	Q642Z0;Q5	29.22988	29.14931	28.51661	28.95191	28.92835	28.69352				8.88E-228	108.76	0.4697829	0.10733795				
atp2a1l	A0JMP4	22.92649	22.90207	19.76885	22.58371	22.45317	22.2287				1.46E-126	108.93	0.1629121	-0.556057				
atp5a1	Q08BA1	26.89342	26.56401	26.19022	26.37323	26.81103	26.69988				1.26E-102	59.743	0.2079117	-0.0788314				
atp5c1	Q5RH26	19.59192	19.44556	21.3921	19.40934	20.70825	21.3701				0.000723	32.148	0.1869676	-0.3527031				
atp5f1	B8JIS1	18.62258	17.81168	22.22046	19.11005	19.26246	20.31351				0.000226	28.169	0.2986204	-0.0104351				
atp5o	Q6DRD1	21.83302	22.2451	19.06917	20.03727	20.74429	20.34258				0.001413	22.482	0.5655467	0.67438444				
atp6v1ab;atp6v	E7FCD8;A2	22.91428	22.0776	18.63267	22.44718	22.70872	22.23163				4.90E-09	68.377	0.0955462	-1.2543271				
atrx	F1QJ36;E7	23.51881	18.97863	21.23794	19.35993	19.64048	20.34714				0.001265	184.59	0.7728982	1.4626166				
basp1	Q1RM09	24.37802	23.34286	23.0579	20.01801	21.58713	20.09324	+	+		2.65E-21	19.996	2.3191302	3.02680206				
baz1b	F1RAR2;A2	27.43165	27.25647	27.15112	27.77731	26.85091	26.8662				3.60E-99	176.26	0.4345822	0.11494255				
bhmt	F1QU55;Q	25.65499	25.4197	25.90635	25.02931	25.63384	26.03402				8.99E-52	44.082	0.4062642	0.09462611				
calrl2;calr;calrl	F1Q8W8;F	20.35536	19.56908	19.65577	20.62806	20.86933	21.23596				4.33E-05	48.64	0.0057311	-1.0510515				
cap1	Q6YBS2;B8	20.60763	19.64622	22.72269	20.08342	20.37794	19.23161				0.000278	49.396	0.7919421	1.09452438				
cast	F1QZT8;E9	26.03967	25.43157	26.50126	20.24681	20.78171	20.54414	+	+		1.63E-33	85.363	4.3277113	5.46661504				
cat;trio	F8W2C4;Q	20.11626	19.42212	20.00854	20.0886	20.34658	20.50865				0.000335	57.123	0.0300229	-0.4656366				
ccdc124	Q6PHE8	19.9287	19.14829	26.38555	19.56419	19.72433	20.98113				0.001398	25.187	0.6020818	1.73096275				
ccny	E9QHJ2;E7	20.75327	19.5344	22.55969	19.80676	20.07347	20.04984	+			0.003171	25.027	0.779173	0.97243182				
ccnyl1	Q08CI4	22.96309	19.58938	18.79136	19.0241	19.32116	19.74516	+			6.92E-06	38.783	0.648171	1.08446948				
cct2	F1QN02	21.037	18.66534	21.33925	18.95192	20.10484	20.89556				0.001655	33.003	0.4323923	0.36308924				
cct3	Q7T2P2	22.80347	19.80939	22.28876	18.7357	22.15872	22.20467				1.85E-15	60.282	0.4531512	0.60084216				
cct4	Q6PH46	23.65261	23.58325	23.90766	22.97412	23.54428	23.93921				4.00E-19	56.973	0.6158975	0.22863642				
cct5	Q6NVI6	21.42936	21.35347	20.09971	19.56428	20.11697	19.84375				0.007744	59.394	1.4459622	1.11918004				
cct6a	Q7ZYX4;E9	22.47941	22.05112	22.01114	21.83495	22.11203	21.95851				0.000101	57.628	0.8535844	0.21205902				
cct7	B3DKJ0	22.75794	22.61964	22.77121	22.62647	22.52542	22.47497				2.43E-06	59.706	1.5421505	0.17397499				
cct8	Q7ZU96	23.28687	22.95874	23.1092	22.96517	23.04143	23.44177				3.08E-08	59.3	0.2470577	-0.0311877				
cd81	Q6PFU1	26.4349	26.08192	26.93461	23.32759	23.50446	24.38614	+	+		8.84E-22	26.198	2.8862837	2.74441592				
cd9b	Q1L9H0;Q	21.90503	23.85833	22.86531	18.65586	19.8092	19.94661		+		8.46E-06	17.142	2.3917549	3.40566699				
cdh13	F1R6D1	20.27139	19.60839	25.49911	20.16333	19.71022	21.02553				0.012004	72.906	0.6230728	1.49326833				
cdk5;cdk4;cdk2	Q9DE44;F1	21.05416	20.43334	24.74689	19.11424	19.4024	20.28994				0.002082	33.399	1.1251482	2.47594007				
cfl1	Q6TH32	23.63821	23.42547	23.25407	22.82429	23.35877	23.89652				4.45E-08	18.77	0.3866296	0.07939084				
chchd3	F1QJD0;Q5	23.93589	23.33347	23.61695	19.43407	20.26518	20.20816	+	+	+	9.82E-15	35.878	3.7801396	3.65963236				
chchd6;chchd6	Q63ZW2;E	24.387	23.70049	24.14274	19.34704	19.9014	18.91438	+	+		8.23E-14	27.54	4.0507207	4.68913651				
chchd6b	F1RBH3	25.95507	25.69347	25.99051	19.6145	20.20757	20.67911	+	+		4.20E-33	25.924	4.5283655	5.71262296				
chmp6b;chmp6	F8W3M3;G	24.9649	24.36546	25.70086	19.07703	19.3938	20.3723	+	+	+	6.48E-17	19.301	3.5231182	5.39603106				
chp1	Q6TH29;Q	26.08211	25.81556	26.53138	19.73267	20.6675	21.04437	+	+		4.68E-30	21.561	3.9690966	5.66150093				
chpfb	I3ISR1;E7F	20.76522	18.89796	23.71576	20.54141	17.76149	20.36546				0.011881	70.989	0.6998021	1.57018852				
cib1	F1Q5E1;F1	22.7667	22.54099	23.13031	21.23056	20.91555	21.00486	+			4.00E-09	21.41	3.3766921	1.76234754				
ckbb;ckba	Q8AY63;R4	25.63486	25.17375	25.62893	25.16672	25.75022	25.142				3.36E-36	42.883	0.494121	0.12620227				
ckma	A2BHA3;B8	27.9673	27.75958	27.60781	27.76047	27.65697	27.94676	+			3.25E-118	42.814	0.277837	-0.0098362				
ckmb	Q7T306	27.94022	28.56997	28.27066	28.02205	27.99037	28.99167				6.79E-120	42.851	0.2413173	-0.0744139				
ckmt1	Q7ZUN7	21.30335	20.55157	22.2212	19.27861	20.88751	20.16173				0.00013	46.706	1.1680327	1.24942144				

cldn7b	Q9YH92	19.69713	20.63019	22.01941	20.51268	20.75752	19.63956				0.000162	22.865	0.552554	0.47898801				
cldne	Q90XR9	23.13787	23.85823	24.30709	20.91297	20.66689	20.98385		+		1.96E-07	22.593	3.2267053	2.9131635				
cldni	Q7ZTS2;Q9	21.46234	21.71833	23.45876	20.02802	19.56167	19.34997	+			2.85E-05	20.9	2.05635	2.56658999				
cltca;cltcb	F1R966;E9	24.86008	24.63229	24.65037	24.72957	24.6826	24.81748				5.72E-29	191.82	0.2023686	-0.0289733				
col1a2	Q6IQX2;E9	21.71754	21.30719	21.74714	21.83113	21.65036	23.28334				2.49E-08	127.21	0.0662432	-0.6643181				
col2a1a	F1QP85;B3	20.21948	19.35687	22.4108	20.14171	19.90612	19.0662				0.00032	119.59	0.7240943	0.95770772				
copa	F1QDQ1;F1	19.62664	21.94163	17.9248	19.33186	19.61328	20.79033				0.000301	138.12	0.2804362	-0.0808004				
cox6a1	A9C462	24.20307	22.87461	23.34665	23.35944	23.86183	24.03002				9.97E-17	12.226	0.1436275	-0.2756564				
CR385063.1	F1Q4P0	21.43559	19.59487	31.50834	20.08157	20.65614	19.8875				0.011893	285.02	0.76357	3.97119331				
CR847973.1	E7EYE1	24.43879	23.50724	23.32773	24.14942	24.02749	23.83856				3.60E-25	150.57	0.1324281	-0.2472343				
cryba1b	Q6DGY4	24.01504	23.61571	23.98902	24.34679	24.29642	24.4891				3.66E-11	23.276	0.005115	-0.5041796				
cryba1l1	Q52JI6;F1Q	24.64889	24.22199	24.2793	24.15885	24.46538	24.08627				4.20E-17	24.537	0.6433828	0.14656321				
cryba4	F1R761	23.95287	24.05159	23.52157	23.21108	21.07641	20.5148				1.05E-11	18.743	1.5574195	2.24124591				
crybb1	F1QT01	26.0005	26.19695	25.57816	25.69716	25.83841	25.83072				3.49E-36	28.984	0.5946058	0.13644028				
crybb1l1	E7F8M1;F1	23.01747	22.5838	18.30666	22.80294	22.98513	22.08638				1.94E-15	25.696	0.1067092	-1.3221728				
crybb1l2	A7E2K5;E7	24.73138	25.24685	24.20978	24.76218	25.10125	25.41674				4.94E-17	25.958	0.0866975	-0.3640563				
crygm2d11	B0UY86;Q4	25.4921	24.80228	24.30111	24.93832	25.41922	25.04556				2.62E-17	21.425	0.1285037	-0.2692032				
crygnb	Q6DGY7	24.43816	24.50264	24.21667	24.4075	24.2839	24.60947				4.96E-21	21.745	0.1967806	-0.0478007				
cs	Q7ZVY5;B2	25.87832	26.15074	26.04824	26.09017	26.3106	25.31572				3.87E-29	51.751	0.4440202	0.12027168				
ctnnd1;CT5836	F1R6C8;F1	19.60278	19.95324	21.85221	20.12984	20.32565	20.50111				6.11E-05	100.08	0.3758312	0.15054131				
ctrb1	F1QFX9;E7	19.6301	18.75769	21.91641	20.61517	20.78537	19.77886				9.83E-06	28.245	0.2158422	-0.2917315				
ctsl1b;im:6910	A7MCR8;A	23.29221	22.70267	23.4008	23.2363	19.94411	23.41622				1.57E-07	38.081	0.6359944	0.93301837				
CU207301.4	E7EXW8;X1	20.40165	18.86851	22.01485	19.97215	20.58393	19.56795				1.54E-05	119.68	0.452168	0.38699659				
CU457819.3	E7EZH8	19.83105	19.95754	24.79625	20.81533	20.32392	20.64793				1.00E-35	16.717	0.5227556	0.93255552				
CU929052.1	E7F0F1	23.02518	24.13132	22.89369	19.3012	20.49711	20.14249		+		1.72E-11	71.765	2.8077918	3.3697993				
cyc1	F1R377;Q3	21.16222	20.70817	23.13146	20.53542	21.19848	20.03908				7.33E-05	13.98	0.8900204	1.07628632				
cyt1;zgc:92533	E9QDY3;Q	20.53797	19.08916	20.34191	19.73923	20.00803	20.55112				2.78E-42	46.294	0.2369292	-0.10978				
cyt1l	E7FCX7;Q1	26.36491	26.04183	26.00084	26.1524	25.97358	26.51391				5.61E-43	46.256	0.1915469	-0.0774371				
ddx39aa	Q8AW05;A	22.94373	22.17231	22.52776	22.34411	21.10433	22.58086				2.43E-10	39.961	0.7567217	0.53816541				
ddx39ab	Q803W0	22.93774	21.74571	19.58363	21.55291	22.19123	22.23795				4.62E-10	48.947	0.1549999	-0.5716705				
degs1	B8A4A2;E7	20.05574	22.79729	19.89161	19.19071	20.39804	19.62664	+			0.006208	38.117	0.8134515	1.17641703				
desma	F1R8W4;F1	23.49008	23.02318	23.20526	23.30647	23.36693	23.50749				2.09E-12	54.149	0.0855249	-0.1541189				
dixdc1a	F1QIN5	20.10809	21.12573	21.95674	20.10523	20.13431	20.19657	+			0.000587	50.332	1.0930737	0.91814995				
dldh	Q6TNU6	22.04926	22.36234	22.38848	22.40616	22.36432	22.33985				1.29E-09	53.62	0.0977325	-0.1034145				
dnm2a;dnm1b	Q4V8Z7;E7	20.51627	19.65823	19.71822	19.10633	18.97314	19.46452	+			0.003666	86.082	1.4768267	0.78291194				
dpysl2b	A8DZ95	22.72854	22.6611	22.06434	22.40291	22.71553	22.59505				4.60E-09	58.284	0.1957849	-0.0865021				
dpysl3	Q52PJ5;E7	20.24297	20.10893	21.81296	21.0561	19.41307	19.88009				0.000532	61.543	0.642306	0.60519791				
dpysl5a;dpysl5	Q52PJ7;Q5	20.29474	19.68223	18.57172	19.13421	19.76163	20.60299				0.011653	61.29	0.1727223	-0.3167165				
dym	E9QFK7;E7	23.0005	22.54964	22.50475	20.067	20.23373	19.69849		+		1.84E-08	59.265	3.8596109	2.68522072				
eef1a;eef1a1l1	Q92005;F1	28.72429	28.22573	28.5346	28.30709	28.46463	28.35132	+			1.30E-121	50.047	0.6252496	0.12053108				
eef1g	Q6PE25	24.81098	24.61291	24.66194	24.91392	24.69848	24.96609				1.64E-19	50.456	0.0410056	-0.1642253				
eef2b;eef2l2	Q6P3J5;A2	27.95986	27.89897	27.73602	27.29141	27.64093	27.57939				5.97E-120	95.497	1.6337304	0.36104329				
eif2s3	F1QGW6;F	22.20967	21.27384	18.68134	21.29075	20.36269	21.59737				0.000218	51.221	0.2084719	-0.3619862				
eif3a	Q6PCR7	22.89308	21.88933	19.52763	22.32858	22.22022	22.2903				1.07E-08	151.29	0.109538	-0.8430195				
eif3ea	F1R279;Q6	19.97792	20.29145	19.86044	19.85809	19.43902	21.26061				0.006208	18.622	0.2266999	-0.1426392				
eif3l	Q7T2A5	21.90135	21.38453	22.03711	22.46229	22.47351	21.88948				3.49E-05	67.992	0.0326564	-0.5007585				
eif4a1b;eif4a2	Q7ZU67;F1	22.82718	22.35859	22.84774	22.53279	22.42306	23.31548				1.95E-15	46.122	0.2285272	-0.0792751				
ela2	F1Q5I4	19.71018	19.09307	21.76158	19.48496	21.45317	20.90695				0.004866	28.799	0.1840118	-0.4267502				
elavl1	Q7ZTS8;F1	19.93534	20.33675	19.78062	18.8736	20.09401	20.4056				0.000951	35.936	0.4739221	0.22650592				
eno1a	Q6PC12	21.16381	19.7949	23.41918	19.62072	18.99179	21.52311				1.25E-61	47.073	0.7714174	1.41409556				

eno3	F1QBW7;E	26.05846	25.97058	26.25357	25.84427	25.86594	26.41622				3.15E-65	51.208	0.39145	0.05205663				
eppk1	I3ISA6	22.25038	21.94967	22.413	19.16018	22.28531	20.54564				1.98E-06	611.71	1.0777181	1.54063543				
fam129aa	Q5RG84	20.29615	19.4331	22.13571	20.46183	19.75569	19.81137	+		+	0.003138	95.259	0.6010891	0.61202304				
fam129ab	E7FC82	22.73316	22.53253	22.79041	20.54842	19.39473	21.77196	+		+	5.16E-05	91.24	1.725081	2.11366272				
fam129ba	A1L1T1	24.91989	23.41828	24.52283	19.14183	20.53485	19.72808	+	+	+	2.23E-28	95.578	3.0565917	4.48541705				
fam129bb	F1R7A2;H9	25.71416	25.52295	25.69171	19.95095	20.99106	20.6529	+	+	+	1.12E-65	85.001	4.3899986	5.11130142				
fam49a	Q6DC29	19.98624	23.10819	18.84984	19.80187	20.15573	19.3135	+			1.15E-06	36.787	0.5770761	0.89105606				
fam49ba	Q6TLE5;B0	25.95593	26.47763	25.48392	18.60857	20.34156	20.76432	+	+		2.70E-37	37.075	3.2670861	6.06767464				
fam49bb	Q6NYL6;F1	24.24627	23.69869	18.52419	19.80103	20.06791	21.52835	+			9.55E-20	36.904	0.6737301	1.69061915				
faua;faub	Q6PC01;F1	18.06293	23.22187	18.81671	20.86262	21.09662	19.59074				0.002003	14.625	0.2173585	-0.4828231				
fbl	Q7ZTZ4	20.30156	19.77501	21.0349	20.09786	18.527	20.57052				0.007491	33.633	0.6739294	0.63869794				
fetub	E7FE90	24.00684	22.36637	25.31741	23.53445	25.61445	25.46631				4.56E-12	56.75	0.1023173	-0.9748631				
flna	E9QI62;F1F	20.27648	21.02526	20.88204	20.89896	20.87613	22.10821				8.90E-07	272.55	0.0685433	-0.5665042				
flot1b	Q6TH07;F1	25.62843	25.92495	25.75165	19.44646	21.01122	20.12131		+		5.10E-35	47.208	3.8719055	5.57534726				
flot2a	Q98TZ8;Q9	30.25034	30.48932	29.43498	21.12565	21.79035	19.2851	+	+	+	3.44E-215	46.92	3.7797414	9.32451439				
flot2b	Q6PFT0;A8	26.9566	26.70912	26.40751	20.48773	19.83842	20.98508	+	+	+	2.99E-102	46.969	4.4541239	6.25400352				
fmnl2b	E7F0M4;F1	26.34181	26.17341	26.22011	19.98024	19.58356	20.75416	+	+	+	2.37E-38	124.41	4.5213663	6.13912265				
fmnl3	F1R4P0;E9	23.41455	22.63456	23.66036	20.13518	21.00025	20.16197	+	+	+	3.03E-19	113.71	2.8839236	2.80401993				
FP325123.1	E7F2T3	20.22039	19.92992	22.64152	18.01711	18.33925	20.80099				7.50E-33	16.503	0.9970759	1.87816493				
fscn1a	F1QET7	20.70312	20.98329	21.15752	21.34158	21.6787	21.97778				1.69E-07	54.697	0.0074106	-0.7180449				
fth1a	Q9DDT0	19.62575	19.95155	22.83291	20.37414	20.62327	20.0246				0.000357	20.719	0.4700519	0.46273104				
fubp1	Q6P2A9	20.47545	19.35626	20.86808	19.98998	19.41715	20.35627				0.000795	66.726	0.5324931	0.31213252				
gapdh	Q5XJ10;E9	25.99721	25.97797	25.99319	25.7982	26.01478	26.09762				3.80E-44	35.784	0.3766939	0.01925405				
gapdh-2;gapdh	Q5MJ86;F1	20.76616	19.03125	21.89256	20.96774	20.24797	19.93149				3.46E-15	36.107	0.3724749	0.1809241				
gcat	F6P2R4	22.37955	19.27297	22.21596	19.68758	20.9651	19.73631				0.00012	46.453	0.7591506	1.15983136				
gdi2	Q6TNT9;A2	23.50337	23.45098	23.53517	23.12084	23.25911	23.72796				1.64E-21	50.586	0.5765538	0.12720235				
glipr2l	Q5XJ73	21.90757	20.85689	21.71377	20.33607	19.59984	20.3597	+			3.05E-05	16.932	1.8722417	1.39420636				
glrx2	F2Z4R4;F1	22.20792	21.521	22.17892	21.3854	21.02423	20.05588				5.07E-09	16.633	1.4842699	1.14744059				
glud1b;glud1a	Q6P3L9;B8	23.35367	23.69147	23.3692	23.41429	23.77886	23.26284				2.68E-11	59.977	0.2777107	-0.013888				
gnai1	Q7T3D3	27.30435	26.99486	26.95737	19.60761	20.35555	20.92334	+	+	+	7.39E-115	40.329	4.4669857	6.79002508				
gnai2a	Q6TNT8	27.61175	27.84757	27.03685	20.77448	19.69581	20.01169	+	+	+	3.60E-105	40.836	4.5829728	7.33806038				
gnai2b	X1WDQ5;F	24.66671	24.13884	23.92425	20.08225	20.34043	20.54543	+	+	+	8.68E-54	40.453	4.2614959	3.92055829				
gnai3	A9JTC8;B8	23.26957	22.91623	22.75513	18.02411	19.90498	19.70239	+	+	+	2.61E-48	40.646	2.7457142	3.76981862				
gnaia	Q7ZW82	25.03486	25.40918	25.53937	20.64683	18.63842	19.85542		+		2.36E-59	43.655	3.4304664	5.61424828				
gnao1a	Q6PBP1	29.29248	29.21523	27.80008	22.22084	21.956	19.96994	+	+	+	4.04E-192	39.976	3.2967554	7.38700676				
gnao1a	F8W442	21.45358	18.78218	25.03691	22.22084	21.956	21.44177	+			6.63E-92	40.259	0.2809529	-0.1153126				
gnao1b	Q6P967	26.46125	26.50039	25.06106	21.06986	19.89318	19.71656	+	+	+	8.72E-59	39.956	3.3913234	5.78102938				
gnat1	Q90WX6	25.19152	26.20747	24.82022	20.01975	21.14541	21.20157	+	+	+	2.46E-49	39.87	3.2122424	4.6174895				
gnat2	Q90WX5;B	30.5106	30.38854	30.36995	19.04675	22.37812	22.62959	+	+	+	1.54E-221	40.189	3.1481066	9.07154338				
gnb1b;gnb1;CA	Q803H5;Q	22.39851	22.00261	22.18583	22.71257	22.53642	22.27153				3.10E-05	37.303	0.0325246	-0.3111928				
gnb2l1	O42248	24.14149	23.74735	23.58703	24.09644	24.48173	24.29207				2.13E-17	35.122	0.0176208	-0.4647897				
golim4a	H9GXX9	20.91511	20.65105	21.39932	18.53512	20.821	19.66775				2.68E-05	75.438	1.1810876	1.31387011				
gorasp1	B0S774;E7	25.88423	25.81487	26.20486	19.87935	20.4248	19.90015	+	+	+	5.34E-23	46.995	5.2794339	5.89988645				
gorasp2	F1RBF9	27.76028	27.47885	27.5847	20.16369	19.4377	21.6024	+	+	+	5.85E-82	45.531	3.7480107	7.2066803				
got2a	Q7SYK7	22.13999	22.39605	18.59732	18.76877	20.30597	19.9843				0.001157	47.586	0.745792	1.35811106				
got2b	F1QCD4	22.97512	23.15191	19.36703	20.82202	21.05728	19.7191				4.10E-08	47.41	0.7275773	1.29855537				
gpm6aa	Q8UUT4	25.428	24.86924	25.35753	22.56962	19.337	23.78806				1.43E-11	30.895	1.4664113	3.32003212				
gpm6ab	Q6DI17;F8	23.25883	22.73554	19.55389	19.98247	19.36634	20.47598				7.34E-11	31.884	1.028115	1.90782102				
greb1	F1QM27;F1	21.98989	24.20262	23.44594	19.56355	19.8378	21.11178	+	+	+	5.70E-16	24.004	2.0107073	3.04177284				
greb1l	B8JKP6	20.12017	19.42634	22.47068	20.33354	19.39834	19.98332	+			8.50E-05	214.83	0.6296717	0.76732763				

gstm;zgc:17399	B8JIS8;A8K	20.20661	19.51954	21.12943	18.81441	20.01393	19.90326				0.007217	26.062	0.8157242	0.70799573				
gstp1;gstp2	Q9DDU5;Q	24.19663	24.13038	24.40093	24.00804	24.10302	24.52594				1.44E-15	23.526	0.3597214	0.0303154				
gyg1a	Q6NV37;Q	23.37172	23.55988	24.90017	23.42291	23.59935	23.92379				4.94E-12	36.966	0.5310583	0.29523786				
h2afv;h2afvb	Q71PD7;Q	23.68442	22.74179	23.56523	23.50312	24.59651	23.79184				3.88E-09	13.509	0.0517485	-0.6333421				
h2afy2	Q4V914;B8	24.97482	23.99378	24.60896	25.04702	25.10881	24.68303				3.79E-21	39.864	0.0588817	-0.4204311				
h3f3a;h3f3b.1	Q6PI20;G1	20.41026	24.43473	20.81861	20.55565	20.00167	18.77865				1.12E-33	15.328	0.9961656	2.10920715				
hadhab;hadhaa	F1QYX8;A7	20.93191	20.49255	23.66319	20.22825	20.3384	20.12506				4.47E-07	82.846	0.969928	1.46531423				
hadhb	H9GXI2	29.03664	30.60752	29.72933	21.04247	21.13859	21.20379		+		7.09E-130	50.216	4.6421146	8.66288185				
hbae1	Q7ZT21;F6	23.40639	23.54828	23.37955	23.34272	23.49412	23.97141				2.44E-06	15.588	0.1153402	-0.1580105				
hbbe1	O93548;F6	27.43873	27.95355	27.36595	27.38435	27.77153	27.84338				2.80E-48	16.162	0.2034636	-0.0803439				
hbbe2;ba1	Q7T1B0;Q	20.40487	20.29188	18.16412	20.42566	20.04368	19.87313				7.74E-08	16.533	0.1380504	-0.4938704				
hbbe3	Q5BLF6	23.54745	22.68699	22.83877	22.50763	22.9167	22.95676				6.79E-08	16.601	0.6136616	0.23070653				
hdlbp	F1R9Y8;E7	22.28359	20.12181	19.05785	17.42089	20.15585	21.09978				0.000277	141.39	0.5544242	0.92891184				
histh1l	A3KPR3;X1	27.39885	26.43175	27.91707	27.91673	27.37898	27.8662				2.64E-37	20.672	0.0890017	-0.4714158				
hmgb1a	Q6NX86	19.9695	20.07043	22.79275	18.85605	19.15221	19.81894	+			3.55E-12	23.695	1.0976659	1.66849136				
hmgb2a	B8JL30;B8J	24.62645	19.72983	24.58474	24.28489	24.42541	24.55912	+			8.88E-16	19.835	0.1038472	-1.4427973				
hmgb2b	Q66IB6	20.71756	19.79277	24.27412	20.79586	19.62799	19.54889				3.73E-08	24.253	0.791703	1.60390472				
hnrnpa0a	F1QS28	21.46782	19.80653	20.71699	21.12043	20.99414	19.66019				8.18E-05	31.994	0.3375361	0.07219696				
hnrnpa0b	F1QTL9;X1	24.11688	24.73283	24.16967	24.37099	24.46961	24.54168				3.35E-14	32.486	0.1498808	-0.1209672				
hnrnpa1	F1QFV2	20.54564	21.85631	19.23456	18.33873	18.6509	19.98841				5.49E-06	40.529	1.0881429	1.55282402				
hnrnpab	F8W4E3;F8	23.12769	22.84038	23.07024	23.17644	23.11514	23.86391				2.26E-08	40.005	0.0503222	-0.3723946				
hnrnpr;syncrip;	X1WH05;Q	20.28902	21.15004	18.90566	20.78792	20.48191	19.31066				0.00042	20.377	0.2699819	-0.0785891				
hnrnpub	Q5RHQ7	22.74727	22.09976	22.06217	22.59571	22.79352	21.92318				3.66E-10	88.549	0.1926294	-0.1344039				
hnrnpul1	Q1L9A1	20.55339	19.35159	20.65228	19.53405	20.63087	19.93128				0.000206	88.961	0.4061588	0.15369161				
hp1bp3	E7FAZ5	24.37981	23.82687	23.99766	24.20479	24.10133	23.82309				3.06E-13	65.168	0.3438778	0.02504412				
hpca	Q6PC34	25.0653	24.5569	25.09039	19.93751	20.73745	20.14545	+	+		2.13E-29	22.441	4.3119208	4.63072332				
hpcal4	A3KPW8	22.94995	23.15058	23.89855	19.93827	20.2009	20.46957	+	+		7.30E-28	22.099	3.4786364	3.13011551				
hsc70;hsp70;m	Q6PGX4;Q	20.6073	19.86835	22.26178	18.12205	20.79917	19.80093				1.60E-31	70.945	0.8649381	1.33842913				
hsdl2	X1WFF1;Q	20.95252	20.01776	20.62623	19.12946	19.68087	21.29286				0.00402	19.046	0.5860172	0.49777031				
hsp90ab1	E7EZ16;O5	25.86583	25.73019	25.92669	25.81343	25.76284	26.21819				6.43E-39	81.567	0.1521625	-0.0905838				
hsp90b1	Q7T3L3	23.01313	22.15041	23.51473	22.14597	20.51737	23.94521				2.17E-11	91.281	0.5581721	0.6899039				
hspa5	Q6P3L3	23.15666	22.93812	24.05374	23.13422	23.29278	24.60575				6.44E-27	71.99	0.1665	-0.2947419				
hspa8;hspa8l	Q6NYR4;F1	25.99499	26.04533	26.21924	25.73053	25.79072	26.40758				1.10E-46	71.179	0.4865959	0.11024666				
hspd1	Q803B0	24.28023	24.08489	24.40782	23.99878	24.09628	24.50652				8.16E-40	61.196	0.4151239	0.05712128				
hspe1	F1QDM6;Q	20.96457	20.40353	20.09905	21.07549	21.3029	20.12071				0.00619	9.1869	0.1191718	-0.3439827				
idh1	B0UXL2;A8	23.1168	23.68175	19.4257	18.43091	21.76371	20.68469				0.000134	48.504	0.7660525	1.78164673				
igf2bp1	Q08CK7	21.447	18.9201	20.57385	20.48889	19.31505	19.45688				0.005119	65.529	0.5718884	0.5600427				
ildr1a	R4GEN6;Q	20.71088	20.32376	22.60098	20.04669	19.88049	20.63967				0.003992	33.48	0.9223076	1.02292379				
ilf2	Q6NZ06	20.32411	19.28194	22.93693	20.9614	21.0578	22.12075				0.000307	42.852	0.1762605	-0.5323238				
ilf3;ilf3b	Q6NXA4;F1	23.96464	23.61919	23.23323	23.22498	23.83836	23.34367				9.68E-16	90.435	0.4854499	0.13667933				
jupa	F1QTT3;Q7	20.92255	20.3197	21.66718	20.40229	20.47853	20.72234				7.01E-06	80.019	0.7706866	0.4354248				
khdrbs1a	A2CE73;Q6	24.68303	24.39034	24.27959	24.81411	25.17234	24.59121				2.49E-09	40.219	0.0270521	-0.4082317				
khsrp	E9QIA6;H0	19.86192	20.0825	20.55122	19.31306	19.59506	21.38332				0.004747	14.105	0.3348821	0.0680631				
kpnb1	F8W2W2;C	23.01693	22.96393	19.61646	22.83621	23.02313	20.78306				1.77E-09	97.466	0.2243541	-0.3483594				
kpnb3	B8JHR9	22.17017	20.02028	19.21977	19.41599	20.57146	19.77358				3.93E-05	122.74	0.5287976	0.54973348				
krt18	Q7ZTS4;A8	22.19814	20.7562	18.70822	22.33612	19.62136	22.32369				3.90E-09	48.63	0.1411547	-0.8728714				
krt4	F1QK60;F1	27.33492	27.06897	26.98794	26.67667	26.96268	27.31683				9.60E-73	53.997	0.574827	0.14521662				
krt5	F1R5A5;F1	23.25465	23.63367	23.46625	23.19248	23.49814	23.86297				5.71E-43	58.786	0.2148854	-0.0663382				
krt8	Q6NWF6	22.58495	22.59204	23.36145	22.19111	19.8385	22.95209				2.01E-25	57.759	0.8393359	1.18558121				
ldha	Q9PVK5	23.56825	22.80778	23.13118	23.37385	23.56023	23.61874				8.61E-13	36.246	0.0477035	-0.3485355				

ldhba	Q9PVK4;E9	25.26985	24.55999	24.6653	24.62048	24.74766	25.04989				3.80E-22	36.246	0.3350722	0.02570852				
lnpa	F1R5C8	24.2344	23.8145	24.37723	21.15503	20.41899	20.5468	+	+		2.17E-11	44.332	3.8790133	3.43510373				
lnpb	Q6PFM4;F1	23.17571	17.20828	19.1228	19.42145	19.7001	21.29236	+			1.17E-12	45.218	0.2511529	-0.3023726				
LOC100004590	E7F8H8;X1	19.91393	19.20398	27.47491	19.19637	20.09254	21.51029				0.005955	44.436	0.5862861	1.93120893				
LOC100333818	F1R915	19.65063	20.19635	24.39421	20.84176	20.41934	21.23757				0.004277	54.033	0.4429216	0.58084361				
LOC100334800	E7FBT1;F1(	20.57623	19.8552	23.66687	18.85874	19.17757	24.19821				1.30E-09	28.757	0.4084941	0.62125969				
LOC100535398	X1WC28;F1	21.10908	19.88507	19.44544	21.56482	20.53416	19.47835				0.011653	16.327	0.1715987	-0.379247				
LOC557250	E7FD36	23.78047	23.36746	24.55075	19.2177	19.173	21.03763		+		8.99E-24	155.34	2.6577734	4.09011714				
LOC557507	E7FFC4	25.87704	26.49034	25.85472	19.62526	20.27851	19.48199	+	+		1.70E-73	40.47	4.692352	6.27878062				
LOC560910	E7F5A4	22.58213	22.62676	23.25241	23.02861	22.98794	22.94297				1.48E-29	20.653	0.1215389	-0.1660741				
loh12cr1	Q5XIX8	22.41222	21.97904	19.78016	18.94937	18.58221	19.97713	+		+	0.000301	23.544	1.4418502	2.22090022				
lrrc57	E9QEY4;Q6	26.15114	26.10129	26.15493	20.34219	20.59232	21.11031	+	+	+	2.68E-30	24.787	5.0518725	5.45418549				
lypla2	I3ISH4;F1Q	19.66951	19.81404	20.02553	19.92291	19.61337	21.57018				0.001469	23.836	0.1068347	-0.5324624				
marcksa	F1Q7Q6	26.33541	26.12881	26.38637	20.33456	20.58492	19.72099	+	+	+	2.68E-10	24.487	4.9458549	6.07004293				
marcksb	F6NXD5	19.88924	22.76786	23.3396	21.81972	20.24374	20.16814	+			9.90E-07	23.255	0.7530971	1.25503349				
marcksl1a	Q6PD99	26.01493	25.65728	25.18382	19.22008	19.70935	19.30788	+	+	+	2.77E-48	22.093	4.8873822	6.20623906				
marcksl1b	Q6NWH2	29.93058	29.61823	29.88553	18.26644	19.52371	21.63956	+	+		4.34E-227	20.979	3.5701362	10.0015443				
matn1	A5WWJ4;A	23.84709	23.42098	23.62064	23.35555	23.70377	23.79174				2.80E-14	53.316	0.3241579	0.01255226				
matn4	F1R4G5;F1	21.75389	21.4768	19.65149	21.7319	19.28619	21.67272				0.000165	72.403	0.3214445	0.06378937				
mccc1	F1QHE1;Q1	31.50233	31.94883	31.47687	32.04189	32.2795	31.52739				0	78.016	0.0753881	-0.3069146				
mdh1a	E7EZF9;Q8	24.2798	24.76238	25.04185	24.67034	24.88795	24.91853				1.81E-14	33.316	0.1575299	-0.1309268				
mdh2	Q7T334	25.63437	26.04306	25.92168	25.8642	26.41522	25.96688				3.34E-19	35.42	0.0857735	-0.2157326				
meis4.1a;meis2	H0WEE7;X1	21.89477	17.95197	19.16184	19.58695	20.55979	21.28613				0.001532	13.262	0.1420415	-0.8080953				
mgrn1b;mgrn1	F1QIN7;Q7	20.15263	20.68506	21.13834	19.08062	20.2042	18.68746	+			5.11E-07	48.377	1.4710227	1.33458519				
mreg	Q6GQM0;I	22.10425	20.97565	22.54809	19.54395	20.29684	21.99589	+			1.17E-06	27.149	0.9646023	1.26376979				
mthfd1b	F1QXP5;K7	21.73422	20.3689	19.25879	19.33195	20.26724	20.88543				0.000229	100.52	0.4276394	0.29242706				
mybphb	A8KBU7;A6	23.11888	23.04379	23.0608	22.68859	23.20799	23.41751				2.56E-08	52.856	0.2582242	-0.0302092				
myh7bb	E9QGC3;E9	19.08984	24.92687	17.51973	21.09084	21.31777	21.18264				2.89E-54	183.83	0.2133943	-0.6849346				
myh9a	F1R889;F8V	23.37928	23.35097	23.59081	23.08427	23.46389	23.67648				2.13E-17	227.33	0.359901	0.03214073				
myhb	X1WF87;F1	19.75372	20.32598	22.03523	20.22952	20.18603	20.78976				0	222.6	0.4607503	0.30321058				
myhz1.1	B8A568;F1	32.4076	32.37974	31.75839	32.44675	32.15068	31.98169	+			0	222.15	0.286873	-0.0111268				
myhz1.2	B8A561	26.21334	25.46799	24.95069	25.92755	25.93629	26.10658				0	222.16	0.0694495	-0.4461308				
myhz1.3	B8A569;F1	25.01129	24.78536	24.4861	24.90293	24.75144	25.04756				0	222.21	0.1158006	-0.1397273				
myhz2	Q6IQX1;F1	25.74399	25.50124	25.18177	25.53517	25.46076	25.63514				0	221.88	0.1905221	-0.0680218				
myl1	Q6P0G6;E9	25.04389	24.90173	24.63073	24.73273	24.40302	24.77535				1.19E-20	20.932	0.8869407	0.22175407				
mylpfa	O93409;E9	26.71148	26.91411	26.42837	26.23803	26.04416	26.77039				1.90E-44	18.865	0.8750929	0.33378919				
mylpfb	E9QG51;Q6	23.39165	24.00556	22.96496	23.13159	22.70407	23.94771				7.31E-29	15.343	0.4530973	0.19293658				
myom1a	E7EYD0;F1	22.17035	20.31562	20.12939	19.59416	20.54811	20.83758				2.14E-08	166.32	0.5946237	0.5451711				
myom2	H9GZB8	19.919	20.41642	21.22717	20.57818	19.44691	19.69413				0.000637	37.612	0.8279962	0.61445808				
naca	Q8JIU7;F1F	20.71813	21.0191	23.15562	19.83379	21.14599	21.31074				2.88E-05	23.4	0.7111561	0.86744626				
ncalda	F1QMZ6;A9	25.83738	25.60648	26.06231	19.63828	18.34603	20.74308	+	+		6.56E-33	22.21	3.3520199	6.25959587				
ncaldb	Q6AXL4	20.8129	18.7476	21.05709	19.89165	19.28058	19.32991	+			8.91E-14	21.984	0.693514	0.70514679				
ncam1a	E9QDC6;X1	19.35581	19.53342	21.31871	19.03288	18.74024	20.41472				0.000839	32.135	0.6443409	0.67336782				
ndufaf4	E7FDX3	24.54716	23.8188	24.35097	18.87751	20.35459	19.69502	+	+		2.03E-13	20.246	3.4805884	4.5966053				
ndufb7	Q6P6E5	26.2578	25.5942	25.36926	19.40162	19.86355	19.10293	+	+	+	4.14E-25	14.483	4.5656806	6.28439204				
neb	B0UY61;B0	26.39862	25.88644	25.70859	26.26798	26.25884	25.83181				3.76E-82	710.09	0.1721308	-0.1216577				
nme2b.2	Q7SXL4;E7	20.61286	21.33967	21.21252	20.30862	21.0508	20.58776				0.0056	17.123	0.881868	0.40595373				
nnt	Q6NYQ7	20.09196	20.17323	22.37812	19.81657	20.01309	20.0915				7.41E-06	112.78	0.8315148	0.90737915				
npepps	F1QRM9	20.21383	18.88981	19.89937	19.83913	21.34645	21.63228				0.001428	103.92	0.0307874	-1.2716134				
ogdha	B8JI08;F1Q	21.77398	21.88945	20.08204	21.80869	19.6948	21.91426				7.31E-06	115.67	0.3409876	0.10924021				

opn1lw2;opn1l	Q8AYN0;Q	22.1077	23.09189	22.26539	18.78544	22.22329	21.16947				1.22E-05	39.518	1.0647934	1.76226234				
opn1mw1;opn1	Q9W6A5;C	23.69339	24.88358	24.68822	23.93058	23.82017	24.66546				1.33E-21	38.844	0.5475202	0.28299141				
opn1sw1	Q9W6A9;C	23.91707	19.11992	24.59838	24.43065	24.36339	24.73908				1.43E-10	37.268	0.0753873	-1.965915				
or137-4	Q2PRG9	19.49064	19.17736	21.97855	19.71246	20.27818	19.27563				0.006485	35.024	0.4897383	0.46009318				
p4hb	B0S564	23.5412	23.39598	24.31292	23.49473	23.12016	24.28842				1.08E-10	56.633	0.3933011	0.11559995				
pa2g4a	Q8AW82	22.41181	23.11251	23.43974	23.14035	23.36653	23.15196				1.51E-09	43.323	0.1246845	-0.2315922				
pa2g4b	Q6PHD8;E	22.3152	19.26389	22.44164	19.431	20.50768	22.37504				1.63E-07	43.475	0.4591853	0.56900533				
padi2	F1QTZ4;E7	20.60887	19.11462	20.30908	20.06522	20.38654	20.58943				0.006971	72.953	0.1317291	-0.3362052				
pald1	Q803E0	24.02563	23.66807	24.31375	19.06135	20.73617	18.86133	+	+	+	2.18E-14	98.884	2.9927346	4.4495341				
pc	F1QYZ6;B0	33.11457	34.14952	33.26919	34.01247	34.47985	32.89858				0	129.99	0.1680765	-0.2858709				
pcca	F1QPL7;B0	33.37503	33.88322	33.58139	34.15934	34.35001	33.65042				0	79.041	0.0362948	-0.4400457				
pcdh2ac	F1QDV3	19.22299	20.32975	25.26939	20.4951	20.74049	19.95855				0.000882	102.41	0.5577875	1.20933151				
pcl	E7F1R1;RE	32.61221	33.22317	33.01192	33.17112	33.47	32.76173				0	129.83	0.1349015	-0.1851819				
pcloa	F6NYB5;F1	19.41872	19.61456	20.80081	19.38938	19.66901	20.34202				0.002114	441.25	0.4016007	0.14456113				
pcmtd1	F1QAE7;Q	24.12707	24.51863	24.45501	19.13219	19.41979	21.33016	+	+	+	1.82E-18	41.188	2.7877533	4.40618896				
pcna	Q9PTP1	22.041	19.73911	19.11403	22.12654	22.4879	22.434				3.21E-06	28.61	0.0186678	-2.0514329				
pdia3	F1QUR3;X1	24.03313	23.5285	24.33858	19.49392	23.53124	24.61526				6.02E-12	51.135	0.6792888	1.41993014				
pdip5	Q90WA5	19.59946	20.94355	21.61509	19.66363	20.27986	18.96838				1.76E-05	48.008	1.0027483	1.08207512				
pgam1a;pgam1	Q7SZR4;B8	20.67105	20.82439	19.00151	19.77064	19.4765	20.29085				0.0007	28.912	0.49566	0.31965065				
pgk1	F1QXV8;E9	23.86882	24.02428	24.6397	24.03825	24.18359	23.95872				2.03E-19	44.732	0.4841068	0.11741257				
pgm1	F1QF00	19.72996	21.52301	19.62828	19.64503	19.91675	19.905				5.25E-05	61.188	0.6104433	0.47148768				
pkma	Q7ZVT2;E7	22.35391	21.86215	18.82003	22.15422	19.85393	22.4371	+			1.05E-06	58.057	0.2040591	-0.4697196				
pla2g7	Q5RHM0;U	25.63148	25.72614	25.6811	19.82025	19.68835	21.33874		+		4.19E-13	50.429	3.5811382	5.39712524				
plgrkt	F1R9U9	24.20911	23.93463	23.84029	20.75368	19.88717	20.78748		+		2.78E-12	13.94	3.7394844	3.51856613				
plscr3b	Q6NY24	22.81073	22.76205	23.36373	20.63145	19.8074	18.91307		+		0.000586	34.599	2.7121772	3.19485982				
pnisr	E9QDD5;F1	22.47425	22.38337	19.12163	20.62648	23.78067	20.75683				9.53E-11	91.902	0.224206	-0.3949083				
pom121	F1R543	21.9025	21.93272	23.10356	20.27973	21.37998	19.73757				0.000173	123.09	1.6818147	1.84716543				
pon3	F1R346;F1	20.07255	20.60375	20.46603	20.01836	19.52167	20.61723	+			0.005025	39.589	0.6917595	0.32835452				
postnb	F1QHK3;Q	23.03154	22.82594	22.42956	23.01558	23.0722	23.1263				3.63E-19	82.911	0.036286	-0.3090153				
ppiaa	B8JKN6;B8	24.0264	23.53848	23.81841	23.63931	24.06332	23.53848				3.02E-10	17.404	0.3789993	0.04738998				
ppiab	Q6PC53	20.71529	21.17399	22.79331	18.6905	20.73334	20.46887				0.000298	17.489	1.1235549	1.5966231				
ppm1aa	Q6NYP6;B8	28.52219	28.4155	28.74522	20.03342	20.08298	21.11494	+	+	+	1.66E-63	42.089	4.921048	8.1505305				
ppm1ba	Q68FN4;B8	25.98072	25.84699	26.35595	20.73093	21.03193	20.18182	+	+		2.45E-54	42.669	4.6047136	5.41299566				
ppm1bb	Q5U386;A	28.74538	26.84589	28.89807	19.31608	19.70322	20.95702	+	+	+	7.14E-70	42.198	3.5346984	8.17100906				
ppm1g	F1QJE5	29.6692	29.77566	30.22894	19.86955	19.25268	21.32899	+	+	+	1.87E-216	53.222	4.2674055	9.74086253				
ppp2r1a;ppp2r	F1R9L6;Q6	18.9452	20.58599	22.62196	20.64498	20.88572	21.12753				1.80E-10	53.171	0.2529259	-0.1683585				
ppp3r1b;ppp3r	Q66HZ0;E9	27.75207	28.20662	28.42394	18.85894	19.15501	20.84563	+	+	+	3.33E-173	19.3	4.0079608	8.50768153				
prdx2	Q6DGJ6	22.89317	21.82788	22.75742	21.94306	22.77975	22.9668				1.26E-08	21.851	0.2540194	-0.0703818				
prkab1a	Q6DHM2;E	19.24361	19.74222	20.78835	20.49903	21.03578	20.42618	+			1.62E-06	30.361	0.0491879	-0.7289302				
prkab1b	Q6NY31;F1	24.69163	23.76223	24.14203	19.53218	20.329	20.58929	+	+		6.87E-09	29.4	3.5015439	4.04847082				
prkacaa	A3KMS9	26.21362	26.40419	25.13414	18.37185	18.683	19.01121	+	+	+	2.10E-46	40.592	4.4100671	7.22863261				
prkacab	Q6DBV8	21.15796	23.97421	23.2627	20.05057	20.96901	19.91913	+		+	2.96E-39	40.87	1.5849727	2.48538653				
prkacbb	Q3ZB92;X1	27.94268	28.00619	27.77913	20.76312	19.98209	20.48123	+	+	+	3.00E-49	40.649	5.5186999	7.50052007				
prmt1;CABZ010	F1R3J9;E7	21.25608	22.09648	18.60104	19.98684	20.39172	21.28667	+			0.000148	40.116	0.329847	0.09612274				
prpf38b	F1Q7F0;Q6	23.26184	24.29271	25.24627	24.45098	25.05283	23.586				2.85E-13	59.512	0.2593072	-0.096337				
psma1	Q6DGX8	21.20681	20.54302	18.79526	20.81503	21.86015	19.58535				2.24E-05	29.248	0.1514523	-0.5718117				
psma6a;psma6	F1R2U4;Q	19.65251	18.64677	21.29978	19.15203	20.28128	20.73956				6.08E-05	25.892	0.2377291	-0.1912683				
psmb5	B0R028	21.28763	20.95385	21.95681	20.16502	19.36981	20.19028				4.46E-05	29.601	1.9948512	1.49106089				
psmb6	F1RCM3;E	21.31893	20.46413	21.8053	20.27498	21.21279	22.10802				0.000282	25.028	0.2998082	-0.0024738				
psmc1a	A8KC30;F8	30.94566	29.39084	29.55468	19.56883	22.8786	20.932	+	+	+	1.22E-193	49.145	3.2170565	8.83724785				

psmc1b	Q6IQ72	26.29784	27.00089	26.25927	20.67912	21.88588	20.56175	+	+	+	3.45E-187	49.211	3.7495003	5.4770813				
psmd1	F1QY43;F1	19.81844	20.41484	21.99642	20.00673	21.60321	21.23169				1.77E-06	106.53	0.2267327	-0.2039763				
psmd2	F1R334	19.52271	21.1295	18.46141	20.02122	20.77956	20.60167				0.00042	55.325	0.0966003	-0.7629414				
psmd3	Q6NYV1	20.67822	20.54526	16.91909	19.74446	21.19234	20.59265				1.29E-05	57.921	0.106385	-1.1289565				
ptgr1	F1QVB1	21.30906	20.03506	20.99245	19.56822	20.48313	20.37728				0.004277	35.862	0.8934853	0.6359787				
pvalb1	Q804W0	20.4395	24.54422	23.95358	25.17448	18.96807	25.28824				1.65E-10	11.428	0.2797113	-0.1644936				
pvalb2	Q9I8V0;Q7	25.0135	25.092	25.4623	24.93764	24.81118	25.01975				2.21E-18	11.622	1.1173494	0.26641083				
pvalb4	Q6IMW7	20.38425	19.88794	20.53875	21.6771	20.70784	20.99165				0.000101	11.581	0.0154809	-0.8552163				
rab6bb;rab6ba;	F1QV30;Q5	21.01003	20.38577	21.94478	19.74532	19.84291	19.42325				0.000951	21.094	1.7283592	1.44303513				
rac1;rac3a;rac3	Q7ZSZ9;Q6	23.0184	22.10454	22.11502	22.21098	22.40083	22.30714				5.79E-06	21.534	0.4276254	0.1063385				
ran	P79735	20.13799	23.43708	23.34001	23.44834	23.82231	19.16322				6.93E-07	24.46	0.3302458	0.16040548				
ranbp1	Q6NWK3;C	21.77459	22.10255	23.47445	21.69153	20.32551	20.36457				0.000269	24.626	1.4342865	1.65665436				
rapsn	Q8QGW4	24.0648	23.09088	22.91046	19.46033	19.93477	19.65315	+	+	+	1.01E-11	46.91	3.4761612	3.67263222				
rars	F1QLT9	19.74818	18.67006	20.06408	20.39582	21.41108	20.00015	+			0.01152	75.755	0.0306408	-1.1082452				
rbm4.2	F1Q7F8	20.95808	20.84459	21.12149	20.76145	20.99858	20.04753				1.53E-06	42.332	0.8565943	0.37219683				
rbm4.3	Q6NXC1	23.3317	22.12003	22.34733	22.51744	23.09323	22.53671				3.91E-09	38.235	0.2198595	-0.1161079				
rcv1	Q6PC38	27.90236	28.30913	28.12736	19.17736	19.50496	20.17393	+	+		1.65E-150	22.618	5.2447652	8.49420039				
rcvrna	Q4V8S2;E7	26.69883	26.64403	26.64224	20.90552	19.51582	19.84601	+	+		3.80E-25	23.376	4.3142222	6.57257843				
retsatl	B0S6C5;F1(	24.01478	23.72858	25.13762	23.66828	24.29642	24.82435				6.31E-15	67.93	0.3197459	0.03064601				
rho	P35359	22.0127	21.47818	22.23836	19.93427	21.00655	21.28354				2.40E-07	39.706	1.4719768	1.16829364				
rnf141	A3KQA9;Q(	22.29109	22.40587	22.39639	20.40618	20.65432	20.9052	+		+	8.27E-07	14.383	3.7866695	1.70921961				
rp2	F1QC45	19.39693	18.9843	21.82974	20.28279	20.53476	20.25293	+			0.000307	41.872	0.2090832	-0.2865028				
rpl10	E9QIR9;Q7	22.45076	22.0348	22.6518	19.82027	22.68517	22.31352	+			0.000544	18.236	0.6506324	0.77279981				
rpl10a	Q6PC69	24.65152	24.32113	24.99399	24.2803	24.77535	24.61223				1.04E-14	24.622	0.4542672	0.09958903				
rpl11	Q6IQI6	21.7411	18.8568	22.93234	19.85166	20.91881	20.10246				0.006516	20.376	0.5864351	0.88577334				
rpl12	Q6DRE6	20.6687	18.66143	22.54142	20.46141	20.86405	21.2717				0.007706	17.685	0.2376778	-0.2418709				
rpl13	Q90Z10;F8	28.25413	27.52432	28.93528	28.34448	28.49933	28.63409				2.60E-47	24.364	0.1467079	-0.2547213				
rpl13a	Q1LYB7	26.60527	26.42077	27.1278	27.22113	27.46505	26.75187				2.46E-23	23.677	0.0515478	-0.4280707				
rpl15	Q6DHS3	26.17936	25.46201	26.35318	26.04827	26.56766	26.34289	+			6.73E-26	24.064	0.0862876	-0.32142				
rpl17	E9QHB7;Q7	18.61609	21.84406	19.4291	19.24331	19.52746	19.46471	+			0.001599	17.829	0.5219029	0.55125872				
rpl18	F1QN76;Q6	29.77194	29.2467	29.61879	30.28879	30.26327	29.48066	+			1.10E-102	19.05	0.0470039	-0.4651				
rpl18a	Q7ZWJ4;F8	27.13585	26.82432	27.63138	27.89285	27.55076	27.73705				1.65E-34	20.68	0.0237183	-0.5297025				
rpl19	Q6P5L3	27.7845	27.01265	28.17655	28.09391	28.5007	28.19065				1.22E-59	23.453	0.0389998	-0.6038526				
rpl21	Q6IQQ0	23.50252	18.73977	19.31436	23.26728	23.99792	23.2839				1.93E-11	18.531	0.0268554	-2.9974836				
rpl22	F1QG80	19.62254	19.26097	22.26579	21.0684	21.29603	20.3418				0.000959	16.289	0.163608	-0.5189762				
rpl23a	F1Q9T9;F1	19.96679	22.72392	21.03156	22.88271	23.80164	22.95351				7.99E-05	17.257	0.0182829	-1.9718615				
rpl26	Q7SXA1	23.47902	22.34188	20.71264	22.17512	22.89833	22.56405				1.88E-08	17.341	0.1805446	-0.3679854				
rpl28	F1QFV6	26.11603	25.78404	26.63755	26.49026	26.36503	26.2265				1.58E-32	15.625	0.1318086	-0.1813927				
rpl3	Q5BJJ2;F1C	26.09857	25.16902	26.19867	25.3123	25.6064	25.60289				1.05E-27	46.255	0.6890093	0.31489118				
rpl30	Q1L8Q7;Q7	23.62868	23.07521	23.63865	23.48996	24.09273	23.25072				6.74E-17	10.656	0.1636149	-0.163627				
rpl31	Q24JV3	24.65709	24.3684	25.19903	25.21434	25.02221	25.16948				3.51E-07	14.338	0.0435213	-0.393837				
rpl32	Q24JV1	29.35454	28.73185	29.74574	29.60451	30.27364	29.88585				5.41E-80	15.813	0.0320728	-0.643959				
rpl34	Q7ZWJ7	24.48265	23.63134	24.21993	24.61891	24.6732	24.95509				8.67E-10	13.369	0.0175633	-0.6377576				
rpl35	Q8JHJ1	27.09305	26.21619	27.10199	27.04989	27.33211	27.77046				1.80E-29	14.43	0.0416945	-0.580403				
rpl36	Q6Q415	26.13794	25.83519	26.576	26.11506	26.30466	26.52151				2.93E-27	12.179	0.1617768	-0.1307004				
rpl36a	P61485	20.27117	19.49365	20.6465	19.36009	20.19703	20.29237				0.002944	12.528	0.4565805	0.18727875				
rpl37	Q6IQJ7	21.18678	20.31208	18.68698	19.82549	21.41866	22.08845				0.00542	11.042	0.0836951	-1.0489222				
rpl39	Q6IMW8	27.70951	27.15875	27.44356	27.75136	27.81042	27.42118				2.09E-06	6.3906	0.0772053	-0.223711				
rpl4	Q7ZW95	25.83702	25.34719	25.82379	25.31741	25.9983	25.64182				3.34E-27	42.552	0.3231121	0.01681836				
rpl5b	Q6IQB1	20.46201	19.61608	20.95688	21.19495	19.54953	20.95663	+			0.008421	34.054	0.2034995	-0.2220459				

rpl6	Q567N5	29.06152	28.8925	29.82249	29.75318	29.51775	29.6531				2.90E-119	30.541	0.0612758	-0.382508				
rpl7	Q6Q417	29.19784	28.9476	28.96502	29.47688	29.45546	29.35296				6.62E-120	28.445	0.0025726	-0.3916162				
rpl7a	Q6PBZ1	25.12904	24.99542	25.0498	25.14137	25.05503	24.99977				1.36E-14	30.01	0.2610482	-0.007302				
rpl8	Q6P0V6	24.22457	23.70957	24.12005	24.15731	24.08367	23.90288	+			2.35E-14	28.066	0.2488117	-0.0298913				
rpl9	Q5BKV9;Q(	22.07002	19.20945	22.29407	21.6706	21.94145	22.18314				5.41E-05	21.654	0.1254216	-0.7405485				
rplp0	F8W4I2;Q6	23.81499	23.91798	23.73314	23.88707	23.36786	23.76497				1.27E-12	17.218	0.6779115	0.1487395				
rps10	F8W2R0;Q	21.48173	18.92212	21.54232	20.72689	21.31816	21.26218				7.05E-05	8.0015	0.1658239	-0.453687				
rps11;si:dkey-1	Q7ZV05;A9	22.14406	22.34952	18.36948	20.29548	19.35114	20.77363				0.000258	18.405	0.5364726	0.81426748				
rps12	Q6PC90	23.55309	24.03154	23.6698	23.69243	23.64713	24.40788				2.30E-15	14.466	0.153529	-0.1643403				
rps13	Q6IMW6;F	23.87764	23.75011	24.18918	24.35756	24.00058	23.86032	+			1.97E-11	17.194	0.1351013	-0.1338425				
rps14	Q6PBW3	20.58118	23.10026	18.96238	23.18723	19.46356	23.41326				4.02E-08	16.245	0.1402378	-1.1400763				
rps15a	Q6Q420	21.23673	20.60433	22.12016	20.25485	18.91239	19.9973				1.60E-05	14.839	1.5479618	1.59889158				
rps16	Q1LWH1	23.66622	23.57681	23.33715	23.23361	23.39493	23.21196				3.55E-11	16.356	1.3166665	0.24656169				
rps18	E9QJF2;Q8	19.81106	17.80468	20.67323	20.51321	20.08656	19.82255				0.005065	11.861	0.1138354	-0.7111168				
rps19	Q6PBW7;E	19.98022	18.31547	21.63063	19.15253	19.4395	19.48045				8.00E-05	15.941	0.5560909	0.61794345				
rps2	Q6NWC3;E	27.37791	27.49335	27.61989	27.71227	27.71352	27.39067				7.93E-60	30.29	0.1093178	-0.1084385				
rps23	A8KB78	23.56011	24.19985	23.17435	24.1325	23.88995	24.19054	+			6.74E-12	15.848	0.056704	-0.4262225				
rps25	Q6PBI5;G1	22.94778	20.54985	19.79448	19.18231	20.01646	22.22123				0.001879	13.86	0.4818121	0.62403234				
rps26;rps26l	Q6P6E2;F1	23.5122	19.82881	19.80647	23.9402	19.71232	23.54369				0.00013	13.021	0.1251872	-1.3495763				
rps27.1;rps27.2	Q4VBV2;Q(	22.3588	23.05195	18.02121	21.30898	21.10556	22.99869				2.20E-06	9.5311	0.1921369	-0.6604207				
rps3	Q6TLG8;I3I	25.76676	25.49945	25.48379	25.42643	25.66251	25.80479				6.30E-33	26.878	0.2059741	-0.0479101				
rps3a	Q6PBY1	25.64031	24.99244	25.42528	25.32529	25.47908	25.7657				1.39E-45	30.199	0.1247724	-0.1706823				
rps4x	Q642H9	24.57594	23.96834	24.56151	24.42124	24.75287	24.38903				3.93E-17	29.688	0.1385153	-0.1524512				
rps5	Q6PC80	23.94584	20.57633	24.12581	23.32251	23.70651	23.86543				1.40E-15	22.872	0.1413236	-0.7488257				
rps6	Q6DHL6	25.70833	25.31513	26.47951	26.0074	26.22396	26.29579				2.23E-36	28.708	0.0936643	-0.3413938				
rps7	P62084	20.12644	20.22858	19.07589	19.37655	19.64063	19.76976				0.007955	22.25	0.5171972	0.21465619				
rps8	P62247;B5	24.66378	24.66259	24.46594	24.54304	24.96768	24.836	+			1.81E-22	24.093	0.0610034	-0.1848005				
rps9	Q6P5M3;A	27.52081	27.04874	27.75602	27.68879	27.73912	27.62094				1.13E-34	22.466	0.0748039	-0.241092				
rpsa	Q803F6	23.66069	23.33142	23.92143	23.64988	23.89273	24.06308				4.70E-10	34.012	0.0785125	-0.2307123				
rras;rras2	Q5XIZ2;Q4	20.41315	20.86399	20.54906	20.87184	21.13819	21.80142				0.008005	13.273	0.0216666	-0.6617546				
rrp12	B8A5Y5;B8	19.63398	18.90214	21.9577	20.32067	20.01488	20.65898				0.011161	31.24	0.2470515	-0.1669057				
rspry1	Q1LX67	22.17518	21.38606	21.51711	19.26851	21.01392	20.98222				8.98E-06	64.873	1.2511004	1.27123388				
rsrc1	E9QJH4	20.06629	19.27545	22.66625	20.40806	20.75804	19.73804	+			0.000439	35.73	0.4273661	0.36794853				
rtn1a;rtn1b	Q6PBQ4;Q	20.1826	19.10103	22.2946	20.76317	20.53179	20.39493				0.011881	23.053	0.2883767	-0.0372194				
rtn3	Q7ZUD6;Q	22.91914	20.61044	23.37557	22.248	22.57274	23.83364				9.63E-07	24.495	0.1502714	-0.583079				
rtn4a	F1QT89;Q4	23.38667	23.52229	23.57565	20.38494	19.77032	22.89428				1.83E-07	22.051	1.5176315	2.47835668				
ruvbl2	P83571	18.91448	23.75807	19.73007	19.0304	19.65659	21.06534				0.008779	51.25	0.5134541	0.88342603				
scamp5	Q6P0C7	19.66904	18.48126	20.75516	20.82255	20.25101	20.55816				0.007866	25.568	0.058156	-0.9087563				
sec61al1;sec61	Q90ZM2;Q	22.35092	21.50325	21.74768	20.41915	21.35151	19.39627				1.67E-07	52.297	1.4254808	1.47830327				
serpina1;serpin	Q5SPJ4;Q5	22.28565	22.27952	22.68648	20.19956	22.30663	21.74573				0.00089	47.653	1.0096458	0.99991035				
sfpq	I3ITB4;Q1L	23.02562	22.24336	22.31255	22.18906	22.24397	22.51203				1.65E-07	68.519	0.6246046	0.21215566				
shmt1	E9QEK5;E9	19.80743	19.37499	20.66787	19.43906	20.49989	20.17815				0.008648	26.523	0.2462624	-0.0889371				
si:ch211-103n1	B0R0K9	22.4807	22.32279	22.34486	22.71398	22.29547	22.23734				6.25E-06	18.818	0.2387001	-0.0328121				
si:ch211-113a1	E7FE06;R4(	20.51793	19.76031	23.49838	20.04063	21.55646	20.42629				7.25E-38	16.831	0.4821268	0.58441353				
si:ch211-12e13	E9QE93;A2	22.82813	22.72315	23.11862	23.27213	24.2454	23.7043	+			0.011353	111.13	0.0108925	-0.8506387				
si:ch211-154a2	F1QA36	21.69635	18.91316	23.24025	19.80127	20.06814	23.44973				2.86E-06	18.573	0.3356664	0.1768748				
si:ch211-157c3	E7FAA1;I3I	25.5688	25.83675	26.57398	19.89178	19.75	21.03111		+		7.41E-54	16.651	3.7769297	5.76888084				
si:ch211-241e1	A8B6E8	20.39849	18.15952	24.99942	19.67327	21.12957	20.22286				0.001127	247.04	0.4542454	0.84391212				
si:ch211-251f6.	F1R5B8;E7	19.00001	18.21861	21.48881	22.81577	20.24157	19.87645				0.000254	27.485	0.0851858	-1.4087906				
si:ch211-253b1	F1QV75	19.41281	19.22707	24.00462	18.69349	19.84232	21.98392				0.00328	53.123	0.4441103	0.7082545				

si:ch211-278a6	F1QL76;E7	24.14965	24.12424	21.55782	18.95974	20.64428	21.52174	+			1.75E-18	127.2	1.4954737	2.90198135				
si:ch211-288g1	A3KPI6	29.17612	28.72991	28.66584	29.16883	28.76929	29.50469				1.22E-44	41.227	0.0801305	-0.2903112				
si:ch211-69g19	F1QZD2	20.53145	22.11051	23.44101	19.47588	19.64085	20.45528	+			1.02E-05	48.455	1.4429982	2.1703186				
si:ch73-52e5.1	F1QG62;B0	19.83415	18.60223	22.15317	19.63406	20.14381	21.97108				0.008218	23.452	0.2126509	-0.3864689				
si:ch73-78o10.1	E7FBV5	24.44025	24.51551	25.54613	18.88223	20.79215	20.56487	+	+		1.34E-19	19.394	2.9109532	4.7542127				
si:dkey-108k21	E9QG44;X1	27.31121	26.78756	28.51822	18.87585	19.17139	21.54263		+		1.28E-44	17.103	3.1318876	7.67570305				
si:dkey-108k21	F1QFS5	19.41434	19.67992	20.36003	19.65749	19.92882	20.98799				0.001218	164.11	0.1225968	-0.3733368				
si:dkey-156n14	I3ISW2;B0J	22.05023	19.46134	19.44718	19.94168	19.91021	20.18526				0.00079	46.961	0.4307767	0.30720266				
si:dkey-190g11	A8WHV7	24.09973	23.56488	24.45004	19.63289	20.48557	20.12562	+	+		6.56E-11	12.144	3.7256932	3.95685514				
si:dkey-21n12.3	F1Q6Y6;F1	21.02651	19.36825	21.14507	18.99431	19.42175	21.15619				0.000457	154.64	0.6061915	0.65585772				
si:dkey-240e12	A3KQR6;A8	19.27209	18.51197	24.44689	19.73145	20.00049	20.27703	+			0.000228	15.342	0.4482791	0.74065971				
si:dkey-241l7.6	Q5RG14;Q	21.55715	19.58914	21.46448	19.47882	19.91139	21.23752				0.010221	17.063	0.6280003	0.66101329				
si:dkey-28b4.8	E9QCZ3;F1	20.43743	20.03218	23.00076	20.02586	20.8678	20.00481				2.16E-34	115.65	0.6700619	0.85730362				
si:dkey-29p10.4	A3KQE0;F1	24.88507	24.39369	24.67271	19.51225	19.78808	20.07294	+	+		3.07E-37	80.209	4.9410027	4.85940043				
si:dkey-67c22.2	B0UYQ8;B0	25.29632	25.94539	25.87479	26.78781	26.64746	24.9936				1.93E-35	121.07	0.1290201	-0.4374612				
si:dkey-9i23.11	F1QJU0	20.4728	18.64657	22.53716	20.0046	20.21621	18.98038				0.002828	49.516	0.5780954	0.81844521				
si:dkeyp-113d7	Q1LXJ9;I3I	20.46003	21.46737	19.5754	21.3688	19.55188	20.88267				3.05E-16	49.934	0.260752	-0.1001848				
si:dkeyp-67a8.4	B0S638;B0	22.08706	22.08932	22.67672	21.55103	19.60823	19.67109	+		+	9.54E-05	30.731	1.704044	2.00758807				
slc1a2b	Q6PH15;F8	21.21841	22.32103	19.55211	19.10537	20.28008	21.62827				8.35E-05	61.266	0.5542062	0.69260915				
slc25a12	F1R5Y8;F1	21.08272	20.13047	19.46792	19.36031	21.05136	19.90509				0.006394	31.039	0.3626504	0.12144661				
slc25a3b;slc25a	E9QJ49;F1	22.71108	23.09806	22.86157	22.6964	22.88813	23.18006				2.24E-05	30.83	0.2481587	-0.0312932				
slc25a4;slc25a6	Q6NX10;B0	22.80369	23.15721	22.45189	21.17268	21.39708	20.12138				1.13E-24	32.688	2.2015759	1.90721766				
slc25a5	Q8JHI0;A2	28.05072	27.47499	27.40844	27.27888	27.27817	27.53148				3.28E-42	32.763	0.8687687	0.28186925				
slc44a1a	B8A445;F1	26.49007	26.85484	27.41683	19.74631	19.32355	20.18635	+	+	+	5.75E-37	71.938	4.6934181	7.16850789				
smyhc1	F1QIR4;F1	26.4615	26.43781	25.90832	26.40876	26.31864	25.94338	+			0	223.04	0.3703504	0.04561806				
snrnp70	Q6DRE8;E7	25.71798	24.83128	26.58635	25.43059	25.94085	26.3734				7.61E-42	57.786	0.2013148	-0.2030767				
sod1	O73872	22.54128	22.57515	23.08159	22.68419	22.94596	23.05161				2.11E-05	15.953	0.1185106	-0.1612453				
spag1a	F1RBN2	23.95065	23.41622	19.45944	19.15067	20.52718	19.99814				7.27E-18	46.984	1.043986	2.38343875				
specc1	E7F789;A2	19.26099	18.5	22.02212	20.87057	19.42913	21.33757				1.20E-05	112.29	0.1667212	-0.6180534				
spna2	F1R446;F1	23.74612	22.81849	24.91566	22.88764	23.95908	24.76532				7.10E-24	284.93	0.2838266	-0.043925				
src	Q1JPZ3	25.16457	25.24529	24.86278	20.38198	20.73447	20.46585	+	+		6.78E-42	60.147	5.3727398	4.56344668				
srl	F1RCU5;F1	22.22084	22.21166	18.2049	18.71222	22.2496	19.3281				7.63E-09	44.415	0.4725266	0.78249677				
srrm1	F8W2X4;F8	19.55849	22.14523	20.37996	18.52774	18.83406	20.60954				0.002663	9.2636	0.9154149	1.37077967				
srsf11	F1RE68;F8	26.1305	26.2781	26.62742	26.16789	26.25997	26.14129				8.74E-84	52.108	0.741316	0.15562057				
srsf5b;srsf6a;sr	Q5SPH2;Q	20.83261	19.72642	22.43781	20.89662	21.38996	21.36182				0.007361	15.96	0.2219329	-0.217186				
stk16	A2CEW6	21.26346	20.65901	21.49726	19.95984	20.2218	20.48965	+		+	2.79E-09	34.884	1.7539643	0.91614914				
stxbp1a	F1QM13	20.68475	18.89423	19.82491	21.18712	19.35847	20.53133				0.006208	67.074	0.1235346	-0.557677				
sumo3l;sumo3;	Q6NV25;Q	20.77324	19.41331	22.27344	20.28218	20.63374	20.36687				0.000139	10.683	0.4801482	0.39240328				
synpo2b	F1RDE4;E9	18.36436	19.7684	19.127	20.50312	20.74826	20.28426				0.006467	64.19	0.0063367	-1.4252955				
syt1a	Q5TZ27;Q6	23.24732	22.70649	22.79891	19.898	20.08654	20.57958		+		2.98E-06	47.396	3.6136529	2.72953415				
tcp1	Q803P2	22.87884	22.42719	22.40385	22.06917	22.37369	22.31205				5.77E-07	60.204	1.1181154	0.31832314				
tfa	B8JL43;F1R	23.11383	23.66514	24.03674	23.77122	23.24144	23.90894				7.43E-15	73.282	0.2682192	-0.0352974				
tgm1	E7F8E0;F8	21.3405	19.55874	21.62055	19.12526	19.48199	20.13621				0.000372	81.958	1.1218941	1.25877889				
tgm1l1	E9QB19;F1	20.68209	20.09689	18.44984	19.23733	19.71716	19.69092				0.006489	87.2	0.4028966	0.19447009				
tkt	Q6PHI8	24.45789	24.18571	24.74786	24.45732	24.45193	24.82522				2.69E-22	67.838	0.1564603	-0.1143386				
tldc1	Q1LWV7	20.33474	21.48458	19.4675	20.77728	20.21304	19.61847	+		+	0.000221	49.949	0.4236885	0.22601128				
tmed10	Q6NWI7	20.53013	19.62725	21.1925	20.06686	18.58086	20.38894				0.000944	24.441	0.7651623	0.7710762				
tmem106b	Z4YHQ6;Q	19.93071	20.07105	22.34995	19.95079	18.95822	19.62434	+			0.000213	29.888	0.9938399	1.27278709				
tmem263	Q7T352	24.32396	24.75073	25.37395	19.77602	20.28788	19.38075		+		3.82E-11	11.865	3.9192524	5.00132561				
tnni2a.4	Q0D2W2;C	23.95855	24.25846	24.65693	23.70398	23.36145	24.38851				7.16E-18	20.084	0.8815979	0.47333272				

tnnt3a	Q9I8U9	20.4743	19.2199	22.41129	20.25982	20.5125	20.21388				0.003378	27.786	0.449989	0.37309647				
tnnt3b	E9QDI4;E7	24.30466	24.28926	25.08695	20.23204	24.17173	25.46441				7.04E-07	26.424	0.6286527	1.27089755				
tnw	B0S6K6;F1	21.89881	22.28484	20.28134	22.32647	22.11063	20.68068				1.53E-10	92.87	0.2216982	-0.2175992				
tomm20b	Q6DH66;E	25.32848	25.11906	25.55672	21.57768	20.06873	21.20755		+		7.57E-61	16.292	3.4296692	4.38343239				
top1	E7FE49	21.21221	19.91805	17.27858	20.09687	19.39045	19.28997				0.000493	61.169	0.2685882	-0.1228186				
tpi1b	Q90XG0;E	23.59855	23.40536	23.98963	23.76314	23.56941	23.87811				6.33E-29	26.827	0.196565	-0.0723769				
tpma;tpm3;tpm	P13104;F1	24.63284	24.62984	24.32471	24.55824	24.65141	25.7496				1.32E-17	32.722	0.0737693	-0.4572856				
trim23	Q08C70;F1	19.6442	19.14791	23.0113	20.67576	20.33977	20.35971				0.000358	47.719	0.3408455	0.14272308				
trim33	E9QIK9;Q6	21.53693	21.54699	23.47828	20.16932	20.42063	20.47275				0.010412	118.01	1.6172468	1.83316739				
ttna	F1R7N8;F1	25.36976	25.4603	24.89634	25.70472	25.67104	25.46778				2.50E-57	3486.7	0.0271944	-0.3723806				
ttnb	B0S6Y0;F6	21.82714	19.11796	17.86598	23.15262	23.88288	22.90263				2.79E-09	626.77	0.0081188	-3.7090136				
ttnb	F1Q6U3;G	23.45501	23.41957	23.22433	23.15262	23.88288	22.90263				2.54E-55	3026.1	0.3624762	0.05359395				
tuba1b;tuba1c;	B8A516;B8	27.86148	27.79334	27.36018	27.72806	27.55954	27.83473				2.04E-77	49.969	0.2399692	-0.0357774				
tuba2	Q6P972	20.44356	21.59528	19.37941	19.59013	20.20017	20.33553				2.93E-56	50.036	0.5523663	0.43080648				
tuba4l	Q6TGS5	22.95399	19.39593	18.3508	22.93542	23.16494	23.93724				6.58E-43	50.214	0.0210097	-3.1122958				
tuba8l2	Q6PC95	21.52737	20.01649	23.87718	20.49197	18.44094	19.76116				6.82E-53	50.158	1.1160999	2.2423261				
tuba8l4	Q6NWJ5	23.91807	24.53285	24.09844	23.93292	24.10125	24.27647				3.43E-73	50.034	0.4430461	0.07957331				
tubb4b	F1R6Y8;F1	22.79808	20.30154	19.81929	27.09668	27.15132	27.37824				3.30E-81	49.788	0.000553	-6.235775				
tubb4b	Q6P5M9;Q	27.04218	27.28965	26.9741	27.09668	27.15132	27.37824				5.25E-98	49.786	0.1118643	-0.1067657				
tufm	Q5BJ17	19.41072	20.28342	22.05745	19.6395	20.3154	20.25935				0.002217	49.244	0.552451	0.51244609				
uba1	F1RCA1;E9	23.73211	22.95738	23.56034	22.97748	23.48775	23.10514				1.61E-12	118.25	0.6338449	0.22648112				
ubb;uba52;rps2	B0R193;Q3	25.27863	24.71015	25.83586	23.8424	23.93355	24.87502				2.76E-17	9.9152	1.3745682	1.05788867				
uchl1	Q6YI49	20.09811	18.93994	21.74004	19.41704	19.06763	21.2481				4.00E-05	24.247	0.4212555	0.34844271				
uqcrc1;pmpcb	F1QUE3;Q	19.39712	19.02527	22.58816	20.75686	18.99467	19.55127				4.49E-05	55.45	0.4742122	0.56924947				
uqcrc2b	Q6IQ59	19.83597	22.73954	18.61617	19.26607	19.8188	21.10671				0.001304	48.122	0.3895271	0.33336767				
usp12a	A4FUN7	20.28907	19.60845	18.9467	20.59742	20.83964	21.08325				0.004327	43.026	0.0089978	-1.2253621				
utp3	Q66I02	20.30651	19.65768	25.86917	18.69653	19.01471	19.89209				0.0015	54.75	0.9158801	2.74334335				
vat1	Q8JFV8	21.13016	22.21493	18.59293	18.66603	19.73776	22.89221				1.85E-06	53.562	0.3449899	0.21400515				
vcp;zgc:136908	Q7ZU99;Q	23.7544	23.65655	23.61336	23.53552	23.87923	24.0164				2.59E-18	89.423	0.1007723	-0.1356169				
vdac1	Q6NWC1	20.66753	20.27128	20.02627	19.23943	20.09162	21.15932				0.000978	30.626	0.3976769	0.15823873				
vdac2	Q8AWD0;F	22.35386	23.02442	22.64143	20.49065	22.13317	21.12925				2.66E-07	30.284	1.5930619	1.42221069				
vsnl1a	Q5TZ35	19.48338	18.1264	24.41067	18.26784	20.4541	20.19142	+			1.21E-32	22.185	0.4970648	1.03569539				
vsnl1b	E7FCW3	28.3803	28.58613	28.51702	18.67126	19.50265	19.48638	+	+		2.43E-57	22.261	5.5995852	9.27438736				
vtg1	Q1LWN2;F	32.21257	32.53832	31.71624	31.57691	31.06272	31.62205				0	149.23	1.4578416	0.7351532				
vtg2	Q1MTC4;F	30.73223	31.11836	30.31581	30.48836	29.79054	30.22871				0	179.89	1.1321215	0.55292765				
vtg3	F1QQQ3;F	27.98799	27.97826	27.77361	27.37857	26.78955	27.11744				2.61E-157	139.68	2.2472921	0.81809934				
vtg4	E9QFD8	33.41097	33.85183	33.02504	30.79355	29.80922	30.83425		+		0	149.35	2.9991267	2.95027097				
vtg4	F1Q7L0;F1	29.33287	29.47812	29.07938	30.79355	29.80922	30.83425				0	149.24	0.0063531	-1.182216				
vtg5	F1R2S5;F1	30.54894	30.82487	30.17188	29.87571	28.84995	29.75017				0	149.26	1.5827836	1.02328873				
vtg6	F1QV15;F1	29.10057	29.67108	28.76465	28.8496	27.52088	28.59413				0	149.9	1.1186184	0.8572286				
vtg7	Q1MTC6;F	28.98566	29.5729	28.77653	28.63031	27.71856	28.61828				0	147.18	1.2617608	0.78931173				
wu:fc28f08	E7EZT8	20.34383	19.66748	31.54315	20.24394	19.86046	20.81438				0.008701	56.408	0.687785	3.54522324				
wu:fe37d09;LO	A3KPR4;X1	31.84464	31.80008	32.0631	32.37176	32.45655	32.13437				2.37E-128	11.367	0.0064451	-0.4182822				
wu:fj41e11;tub	F1R4K1;Q6	21.03847	18.94045	20.66327	27.09668	27.15132	27.37824				2.32E-88	49.646	9.27E-05	-6.994681				
yes1	A1A5H8;F1	27.47923	27.36986	27.42726	20.28288	19.01283	20.79042	+	+		5.03E-72	61.268	4.1179752	7.39674314				
ywhaba;ywhab	E9QJ96;Q5	25.40999	25.28118	25.47268	25.17581	25.12664	25.55614				1.45E-19	25.757	0.5794095	0.10175578				
ywhabl	Q6P102	22.55682	22.50978	22.67297	22.23377	18.77846	23.06554				5.20E-11	27.907	0.6932202	1.22059886				
ywhah;ywhag1	Q7T3G2;Q	19.70492	21.18204	20.64752	19.48743	19.76403	18.68094				2.01E-09	27.932	1.3447886	1.20069186				
ywhaqa	Q7ZUM0	21.36731	19.9234	21.36057	20.26485	20.54705	20.31852				1.06E-10	27.764	0.7477791	0.50694911				
zgc:101566	Q5XJU2	21.47956	19.22207	18.9768	18.71416	20.58638	20.43209				0.004592	12.85	0.2951688	-0.0180677				

zgc:110425	F1R314;Q6	22.82918	18.66368	24.17326	23.13353	22.94061	23.11207				1.07E-23	20.24	0.1303526	-1.1733646				
zgc:112425	Q566W6	20.823	19.11983	21.84153	20.05643	21.52916	20.54381				0.00036	18.454	0.2615937	-0.1150119				
zgc:112994	E9QCQ1;F1	25.22509	24.50882	24.55888	20.53764	20.28578	19.81699	+	+		1.07E-15	37.001	4.1873607	4.55079269				
zgc:113358	E7FAF6;Q5	19.5	18.06076	19.23359	19.82644	20.85684	19.71265				0.010584	14.409	0.0232034	-1.2005278				
zgc:113984	Q4QRF4;X1	28.30069	27.98212	27.79495	19.23129	21.0002	21.2507		+		1.76E-86	15.388	3.7937545	7.53185527				
zgc:114037;zgc	R4GE02;X1	29.05375	28.93078	29.07449	29.26548	29.241	29.28505				2.28E-83	27.149	0.0013818	-0.2441692				
zgc:123103	A8E4Z6;F1	20.00324	20.19447	20.52736	21.16533	21.96455	21.61199				0.001255	35.972	0.0018445	-1.3389339				
zgc:136220	F1R6Y6	24.60891	23.40341	23.30034	19.4319	20.2132	21.42802		+		1.64E-14	23.79	2.3520467	3.41317813				
zgc:136380	F1Q9F6;F1	24.65179	25.41313	25.94635	25.17813	25.76608	25.90228				3.81E-56	55.416	0.142129	-0.2784081				
zgc:153405	X1WGK9	20.2219	21.75099	19.50545	19.93828	20.20091	21.37703				4.26E-38	20.656	0.2958972	-0.0126247				
zgc:153675	F1QZ92	24.33395	24.82479	24.85391	24.80302	25.26137	25.24322				7.66E-21	18.05	0.0287888	-0.4316552				
zgc:153722;zgc	F1QLE3;F1	21.99652	21.55848	21.41235	19.95406	19.16921	20.62502	+			0.000195	32.659	2.0260826	1.7396876				
zgc:162618	F1Q9X8	20.49346	23.86467	24.00161	19.70557	19.97541	20.83852	+			2.90E-12	49.884	1.3260412	2.61341031				
zgc:163061	E7F225;A4	24.1916	23.65524	25.02458	24.62561	24.67573	24.27469				9.25E-14	11.732	0.1561669	-0.2348741				
zgc:163069	F1R2V7	24.75781	24.77836	24.8292	24.5131	24.62199	25.40662				1.00E-22	56.474	0.2385264	-0.0587788				
zgc:173552	A8KBJ5	26.62478	19.30027	18.21681	19.32999	19.11186	25.91027				3.59E-25	15.404	0.2944763	-0.0700849				
zgc:195633;zgc	F1Q909;A4	20.4185	19.748	26.81836	29.26548	29.241	29.28505				2.08E-15	13.632	0.008112	-6.9355558				
zgc:56072	E9QDK6;Q	20.80491	20.24737	21.62925	20.70417	20.94308	21.0276				0.001097	18.376	0.3027904	0.00222715				
zgc:63569;slc44	H9GX71;Q	23.0913	23.52229	24.05159	19.74793	19.47252	20.23618		+		5.49E-13	73.128	3.6310649	3.73618253				
zgc:63695	Q7SXX4;Q5	26.35568	25.7335	26.71122	20.12097	19.03559	20.60471	+	+		3.05E-20	22.379	3.8088928	6.34638087				
zgc:66127	E7FAQ8;E7	20.42992	20.57967	18.6052	19.47296	20.30009	18.12585				0.000133	38.531	0.5541634	0.57196299				
zgc:73075	I3ITF4;Q1L	26.15513	26.13401	26.19363	18.85054	19.14687	19.76576	+	+	+	8.39E-41	21.685	5.157719	6.90652847				
zgc:77052	Q6NYA1	21.21275	20.90894	22.35485	20.07046	21.9195	21.61883				4.56E-05	34.248	0.4501792	0.28924942				
zgc:77262	Q6P0G2	22.97963	22.16604	22.49392	22.8364	23.54922	22.6868				4.80E-08	37.257	0.0581621	-0.477609				
zgc:86599	F1R1J3;F1R	21.59745	20.87934	20.56272	19.60787	20.12321	21.81687				0.012086	11.405	0.572364	0.49718412				
zgc:86723;cryg	B0V191;A7	23.00707	22.77239	19.82909	22.70637	22.94127	22.95878				6.67E-10	21.459	0.0928833	-0.9992879				
zgc:86725	F1RCB6	20.05084	19.86977	23.18852	19.94514	20.09966	20.48138				3.37E-150	41.976	0.6257766	0.86097972				
znf395	Q7ZW35;Q	23.49045	23.90582	23.11916	19.318	20.16188	20.04182	+	+		9.76E-12	37.135	3.6383906	3.66457558				

Table S8: Quantification of myristoylation during zebrafish development

Sheet 1 : Raw dataset of triplex dimethyl labelled samples

Zebrafish embryos were pulsed for 24 h with 20 µM YnMyr (or Myr control) after 5, 48 and 96 hours post fertilisation. The embryos were then lysed, tagged proteins were captured with reagent 2, enriched, digested with trypsin, dimethyl-labelled (please see methods' section for details) and analyzed by LC-MS/MS. The data were analyzed with MaxQuant (version 1.5.0.25). The data are unfiltered. The table is showing a total number of protein IDs together with avarage logarithmized intensities of light (24h), medium (72h), and heavy (120h) unique and razor peptides. Given is also the number of unique and razor peptides found in each sample. Proteins are ordered by gene names.

Sheet 2 : GO Annotations

The data were further processed in Perseus (version 1.5.0.9). Two out of three valid values per time point were required and proteins without N-terminal glycine were removed. Subsequently, entries with no significant enrichment in YnMyr/Myr feeding experiments were removed from the list. For the residual proteins a heat map of myristoylation levels in developing zebrafish (Figure 2c) was prepared following normalisation (z-scorring) and basic gene onthology annotations were gathered. Proteins are ordered by gene names.

Gene names	Log2 Intensity Light (24h)			Log2 Intensity Medium (72h)			Log2 Intensity Heavy (120h)			# Unique and Razor Peptides		
	Sample1	Sample2	Sample3	Sample1	Sample2	Sample3	Sample1	Sample2	Sample3	Sample1	Sample2	Sample3
acaa2	20.61702	23.36826	21.73149	19.52449	24.14833	20.17694	19.75215	24.75108	23.6202	1	4	1
acaca	22.64792	23.26871	24.24721	21.10066	22.64258	19.98315	20.91729	21.20854	23.33729	1	2	1
acadm	NaN	NaN	20.64253	NaN	NaN	NaN	NaN	NaN	NaN	0	0	1
actba;actbb;actb1	25.17272	25.27498	26.23562	24.68314	26.03231	23.90812	24.69636	25.52187	24.51581	3	5	3
actc1b	25.86783	26.76699	27.14734	26.9173	29.1497	26.25406	27.06301	28.79408	27.82317	7	13	8
actn3a	NaN	NaN	NaN	NaN	20.76636	NaN	NaN	NaN	NaN	0	1	0
adh5	18.56078	NaN	NaN	NaN	NaN	NaN	NaN	NaN	NaN	1	0	0
ahcy	NaN	21.30352	20.53174	NaN	22.46446	17.1138	NaN	21.97418	19.49746	0	1	1
ahnak	NaN	18.97085	21.03517	NaN	20.72567	20.46483	NaN	19.84866	19.61947	0	1	1
ak1	NaN	18.16957	NaN	NaN	21.59819	NaN	NaN	21.09552	NaN	0	1	0
akap12b	20.55075	20.24237	23.12286	22.57499	21.06178	23.35286	21.85121	21.02952	16.51522	1	1	2
aldh18a1	22.51989	22.0693	23.06745	20.2247	19.87398	19.7835	18.88298	20.12994	19.88481	3	2	3
aldh6a1	21.86491	NaN	NaN	18.94829	NaN	NaN	16.89936	NaN	NaN	2	0	0
aldoaa	NaN	19.82907	NaN	NaN	22.14672	NaN	NaN	21.93776	NaN	0	1	0
anxa13	24.62617	24.39872	24.63006	23.92886	25.48965	23.32003	23.3692	25.31122	23.22164	6	8	4
anxa13l	NaN	20.82376	NaN	NaN	20.91656	NaN	NaN	20.13107	NaN	0	1	0
anxa1c	NaN	NaN	20.81143	NaN	NaN	19.6206	NaN	NaN	18.31079	0	0	1
apoa1b	19.3059	NaN	NaN	19.84233	NaN	NaN	21.72771	NaN	NaN	2	0	0
apobb	21.36196	24.03616	21.40041	20.86204	25.56209	19.98579	18.83121	24.04018	19.49508	1	14	1
arf1	25.10153	24.26406	25.6045	21.98597	23.17557	21.78377	21.76065	22.56697	21.62453	1	1	1
arf1l;arf3b;arf2	26.60752	26.18764	27.3784	24.66476	25.57782	25.3655	24.36393	24.97285	24.31748	2	2	2
arf4a	26.66085	26.44172	26.80033	23.88334	25.32687	23.45951	24.14203	24.6053	23.60546	4	3	3
arf5;DKEY-238C7.11	28.99145	27.7465	28.79219	26.85186	27.36085	25.82216	26.82214	26.92014	25.9459	6	4	5
arf6b	24.86089	24.49515	24.87446	22.93414	23.70504	22.10415	21.91287	23.24086	21.62176	3	4	3
arl1	26.08361	25.37832	26.38752	23.36011	24.30285	23.37836	22.90266	23.70683	23.46239	6	5	5
arl8;arl5c;arl5a	23.28164	20.53602	23.37013	17.55382	17.94266	17.70077	NaN	17.10728	NaN	2	1	2
atic	NaN	NaN	21.59919	NaN	NaN	NaN	NaN	NaN	NaN	0	0	1
atp1a1	20.45051	18.34069	22.58224	21.14208	21.67827	20.70498	22.67898	22.61688	22.31133	2	2	2
atp2a1	20.91371	25.45271	21.70147	21.8055	27.25791	22.1545	21.59723	25.87427	23.01306	2	16	2
atp5a1	NaN	NaN	23.44607	NaN	NaN	NaN	NaN	NaN	NaN	0	0	2
basp1	20.65018	21.69073	20.08939	25.03082	24.29929	23.27838	24.6097	24.71183	22.021	3	2	1
baz1b	23.17779	23.13452	21.68286	23.66427	24.40347	21.00546	22.10894	21.62149	20.38195	3	7	1
bcam	19.49457	NaN	21.1121	NaN	NaN	NaN	NaN	NaN	NaN	1	0	1
bhmt	NaN	20.67452	NaN	NaN	21.79015	NaN	NaN	22.70276	NaN	0	1	0
cast	21.85836	22.10204	24.41738	22.80513	23.3048	24.87141	22.02479	22.28797	21.62382	3	2	5
ccnyl1	21.8423	22.13113	21.72071	19.80622	21.10226	20.12855	19.63996	19.86104	18.78309	1	1	1
cct3	NaN	NaN	21.37342	NaN	NaN	NaN	NaN	NaN	NaN	0	0	1
cct6a	NaN	19.6934	NaN	NaN	20.12502	NaN	NaN	19.13212	NaN	0	1	0
cd81	24.65633	25.4989	26.52876	23.90921	23.77384	24.21327	25.74932	24.55871	24.48056	5	4	5
cd9b	NaN	20.63041	21.37263	NaN	18.7604	NaN	NaN	15.60484	NaN	0	1	1
chmp6b;chmp6	22.49077	23.32182	23.13948	22.04697	23.12951	21.01502	21.18586	22.30502	21.40151	1	1	1
chp1	22.66536	NaN	23.38166	NaN	NaN	20.45151	NaN	NaN	19.88991	1	0	2
ckma	22.64535	24.1934	23.01357	25.02149	27.60724	23.28292	25.13817	27.0357	24.98936	5	10	4
ckmb	20.56746	22.808	NaN	21.93124	25.61557	NaN	22.07006	25.8169	21.59355	1	5	1
cldne	NaN	NaN	21.74932	NaN	NaN	NaN	NaN	NaN	NaN	0	0	1
cldni	20.75426	21.62484	22.49617	20.90021	20.95433	20.60277	18.52263	20.01591	19.7022	1	1	1

cryba1b	15.53223	19.16619	NaN	18.38486	23.51641	NaN	21.78676	23.53112	21.35515	2	2	1
cryba1l1	NaN	NaN	NaN	NaN	NaN	NaN	21.08537	NaN	NaN	1	0	0
cryba4	NaN	NaN	NaN	NaN	NaN	NaN	NaN	22.55269	NaN	0	1	0
crybb1	NaN	18.94037	NaN	NaN	23.15532	NaN	20.63068	22.09239	21.1881	1	2	1
crygm2d3;crygm2d7;si:ch	NaN	21.14171	NaN	NaN	23.85776	NaN	NaN	22.51357	NaN	0	3	0
cs	22.22305	22.9831	NaN	19.1123	24.01614	NaN	19.29343	23.7291	NaN	2	5	0
ctnnd1;CT583626.2	NaN	NaN	20.69804	NaN	NaN	17.16497	NaN	NaN	14.29383	0	0	1
CU929052.1	NaN	NaN	NaN	NaN	NaN	NaN	21.26058	20.53469	NaN	1	1	0
cyt1	21.69969	20.98447	21.9451	21.44481	22.23301	20.44607	22.24858	21.86106	21.11121	1	1	1
ddt	16.79195	NaN	NaN	17.47048	NaN	NaN	21.48817	NaN	NaN	1	0	0
ddx39aa	19.97827	19.83039	NaN	18.56074	20.50087	NaN	18.97449	19.6829	NaN	1	1	0
eef1a	25.70372	25.7397	26.01468	24.05648	26.55076	23.85871	24.10765	25.62154	24.40737	4	5	3
eef1b2	NaN	20.72692	NaN	NaN	20.30486	NaN	NaN	19.70126	NaN	0	1	0
eef1g	NaN	22.28811	23.20674	NaN	21.76219	19.76273	NaN	21.63462	21.50925	0	2	3
eef2b;eef2l2	25.29067	25.36199	24.79734	21.1237	25.30438	21.3626	20.39331	24.28898	21.81679	6	11	6
eif4a1a	NaN	21.75471	21.70303	NaN	21.42808	18.82246	NaN	21.13151	19.92217	0	1	1
eno3	21.38427	20.43797	23.37106	19.88344	NaN	19.54561	20.23433	NaN	20.79305	1	1	3
ephx1	NaN	21.34191	NaN	NaN	16.75094	NaN	NaN	17.34117	NaN	0	1	0
fabp11a	24.86576	NaN	31.39018	23.42931	NaN	23.40561	23.64097	NaN	16.02287	1	0	1
fam129bb;fam129ba	21.94489	21.65984	21.50028	20.56997	22.44599	20.21857	20.61396	21.65905	20.18628	2	2	1
fam49a	NaN	20.45774	NaN	NaN	21.25822	NaN	NaN	20.87912	NaN	0	1	0
fam49ba	23.77324	NaN	23.849	21.26499	NaN	21.57851	21.01788	NaN	21.13358	3	0	3
flot1b	21.746	22.50741	23.44012	NaN	20.35993	21.39404	NaN	20.24922	19.81222	1	1	2
flot2a	27.33194	27.14812	27.72793	26.18149	27.38303	25.72148	25.35134	26.67344	25.05205	9	12	11
flot2b	21.76369	19.4628	21.13796	21.52745	21.9102	20.30452	20.55178	21.52583	18.85571	2	2	1
fmnl2b	21.74505	NaN	NaN	18.90301	NaN	NaN	17.72773	NaN	NaN	1	0	0
fubp3	NaN	NaN	22.41738	NaN	NaN	NaN	NaN	NaN	NaN	0	0	1
fyna	19.20263	NaN	19.80821	18.61712	NaN	17.69187	17.54653	NaN	16.48721	1	0	1
gapdh	20.56932	19.92493	22.74417	17.66442	20.85316	21.10521	19.59107	19.51563	23.63345	1	1	2
gdi2	NaN	20.61495	NaN	NaN	20.6868	NaN	NaN	19.51054	NaN	0	1	0
gnai1;gnai3;gnai2b	24.22391	22.26608	24.21934	23.04808	23.54851	22.92665	22.86663	22.92571	21.68932	2	2	2
gnai2a	23.81714	22.94849	23.79867	22.65007	22.95355	21.87882	22.34733	22.76324	21.09905	1	2	1
gnaia	24.61184	22.87489	24.79352	21.99213	22.21386	21.89807	20.99335	21.67314	20.11921	4	3	4
gnao1a	25.96987	25.27473	25.45951	25.71201	26.04579	24.42905	25.09124	25.91982	24.18495	4	6	3
gnao1b	NaN	20.6479	NaN	NaN	22.68581	NaN	NaN	22.62201	NaN	0	2	0
gnat1	NaN	18.63336	NaN	NaN	18.06245	NaN	21.04166	24.34252	NaN	1	5	0
gnat2	25.63425	25.25735	25.3762	24.49149	25.14433	23.57992	26.52228	28.16663	27.05258	5	6	5
gnb3b	12.90552	NaN	NaN	16.10942	NaN	NaN	18.65903	NaN	19.2632	1	0	1
gorasp2	23.75786	23.05626	23.49314	22.15743	23.29446	21.47035	21.48454	22.40626	20.87245	2	2	1
got2b	NaN	NaN	21.0125	NaN	NaN	NaN	NaN	NaN	NaN	0	0	1
gpm6aa	NaN	NaN	23.46064	NaN	NaN	22.63059	24.32155	NaN	25.45284	1	0	1
gpm6ab	19.7703	19.86195	NaN	22.65211	21.30168	NaN	24.56349	22.63806	NaN	2	2	0
gstp1	NaN	22.49678	22.47028	NaN	22.34522	19.72915	NaN	22.74876	20.17962	0	2	2
h2afv;h2afvb	22.93789	22.41604	23.47346	22.53626	23.27056	22.33301	23.74335	22.65193	22.58687	2	1	1
h3f3a;h3f3b.1	NaN	NaN	23.32773	NaN	NaN	21.18725	NaN	NaN	18.30879	0	0	1
hadhb	27.60392	27.45552	27.25341	26.33301	27.69458	24.93468	26.61709	28.14258	26.20618	9	12	8
hbbe1	22.25859	23.89606	23.11816	23.70683	26.31206	23.52409	24.11084	25.73892	25.28828	3	6	5
histh1l	17.93053	NaN	18.58167	18.33407	NaN	19.41627	21.18562	NaN	20.2049	2	1	1
hmgb1a	NaN	19.18853	NaN	NaN	21.0921	NaN	NaN	20.65962	NaN	0	1	0
hmgb2a	NaN	21.37613	NaN	NaN	21.48449	NaN	NaN	20.90984	NaN	0	1	0

hmgb2b	22.50164	22.66763	NaN	21.69422	26.25994	NaN	20.10904	21.45583	NaN	1	1	0
hnrnpa0b	NaN	20.61306	NaN	NaN	21.69004	NaN	NaN	20.59669	NaN	0	1	0
hnrnpub	19.5765	19.24832	19.75491	16.12475	20.09288	NaN	NaN	18.22256	NaN	1	1	1
hpca	25.12892	24.84556	25.6332	24.58503	25.17798	24.80351	25.15777	25.35944	24.34462	5	5	5
hpcal4	20.2995	NaN	NaN	22.13649	NaN	NaN	22.97339	NaN	NaN	2	0	0
hsp90ab1	22.71284	23.01151	24.18986	21.12319	23.48235	19.80663	21.01917	22.81857	20.32575	2	3	4
hspa5	NaN	NaN	20.917	NaN	NaN	17.90201	NaN	NaN	16.94866	0	0	1
hspa8;hspa8l	NaN	22.61286	24.8606	NaN	22.38269	22.10559	NaN	21.95794	21.39341	1	2	4
hspd1	NaN	NaN	20.79931	NaN	NaN	18.39674	NaN	NaN	18.3653	0	0	1
hspe1	NaN	21.00038	NaN	NaN	19.1541	NaN	NaN	19.19075	NaN	0	1	0
khdrbs1a	NaN	20.57801	21.07254	NaN	21.28805	18.82938	NaN	19.66809	18.36807	0	1	1
kpnb3	NaN	NaN	20.61018	NaN	NaN	20.02052	NaN	NaN	15.38987	0	0	1
krt4	24.3507	24.03162	23.67023	25.00367	25.77666	22.1254	24.5418	24.99049	24.3575	6	9	3
krt5	NaN	20.8631	21.82676	NaN	21.59905	18.70046	NaN	21.25055	19.26547	0	1	1
ldhba	NaN	21.28726	22.70531	NaN	21.74497	19.56704	NaN	21.42645	20.53117	0	2	2
lnpa	NaN	21.1038	22.44066	NaN	19.77836	19.3467	NaN	20.65587	18.42267	0	1	1
lnpa	24.09788	22.83642	24.4095	22.28923	21.94956	21.63019	19.46152	20.68782	16.83257	3	2	3
LOC100148604	NaN	NaN	27.43261	21.0654	20.44738	26.90999	NaN	NaN	25.57219	2	1	1
LOC557507	23.92406	21.76737	23.29292	21.29249	21.0818	20.68097	21.14221	21.50218	20.62694	3	1	2
LOC561593	24.62232	22.03245	24.50736	22.09697	19.38518	21.86944	20.41328	17.67707	20.34863	3	1	3
lrrc57	23.6698	22.8834	23.55999	20.86386	21.66401	21.05384	21.37454	21.92278	20.82811	3	2	2
marcksb	25.04623	25.56037	26.09607	24.11323	23.28093	24.20202	22.47647	22.25231	20.921	2	2	2
marcksl1a	22.59366	21.68603	24.92121	21.4405	20.02012	22.86488	22.04913	20.39215	20.63945	3	1	3
marcksl1b	29.31092	29.29215	30.799	29.37997	28.82065	30.10225	28.97454	27.80858	26.9476	5	3	3
mccc1	27.82941	27.44063	27.88838	28.1693	29.68616	27.46762	27.12573	28.19427	27.81623	13	17	10
mcm3	NaN	NaN	19.66607	NaN	NaN	NaN	NaN	NaN	NaN	0	0	1
mdh2	21.23736	22.01321	23.27412	19.34419	21.19713	19.93301	19.84749	21.36528	21.32097	1	1	2
mybphb	NaN	18.39837	NaN	NaN	21.2794	NaN	NaN	21.6759	NaN	0	1	0
myh6	NaN	NaN	22.13987	NaN	NaN	22.45538	NaN	NaN	21.36169	0	0	1
myh7bb	22.74993	23.53362	22.9393	24.60885	27.08618	24.47717	25.0312	26.24585	24.83244	1	1	1
myhz1.1	26.50913	27.29588	26.27491	28.3697	30.51606	27.27088	28.116	29.62838	27.88518	34	56	24
myhz1.3	NaN	22.4138	NaN	NaN	23.05915	NaN	NaN	22.28037	NaN	0	3	0
myhz2	18.61673	22.07738	NaN	21.17652	24.37398	NaN	20.97274	24.02454	NaN	1	5	0
myl1	17.34992	17.84512	NaN	20.50978	23.22792	NaN	19.69168	20.8119	NaN	1	2	0
mylpfa	19.98022	22.25848	19.54374	21.80629	24.26464	20.16349	22.41743	23.66373	21.8266	2	4	2
mylz3	NaN	NaN	NaN	NaN	NaN	NaN	NaN	20.45372	NaN	0	1	0
myo5aa;myo5b;myo5ab;	NaN	27.54621	29.64385	NaN	28.18782	28.51807	NaN	28.05258	28.37915	0	1	1
ncalda	25.38067	25.27064	25.85505	24.20404	25.87366	23.64163	24.2776	25.07182	23.42842	2	3	3
ndufb7	24.44752	24.90862	24.83186	24.3579	25.4921	23.99602	23.86344	24.37518	23.93553	2	3	1
nme2b.2	NaN	23.07573	23.42329	NaN	22.01947	NaN	NaN	20.05126	NaN	0	2	1
nme3	21.22541	22.21122	NaN	21.2715	22.22341	NaN	22.08965	22.00666	NaN	1	1	0
nop58	NaN	26.31095	27.93444	NaN	26.94246	26.93382	NaN	26.72988	26.89377	0	1	1
opn1sw1	NaN	20.72501	NaN	NaN	20.3673	NaN	NaN	22.56772	22.163	0	2	1
p4hb	NaN	23.15912	24.77937	NaN	22.4278	21.01536	NaN	21.3093	19.47657	0	5	6
pc	27.95837	27.39795	27.67559	28.62373	29.97624	27.13114	27.07419	30.15786	28.29636	19	24	18
pcca	29.51709	29.01255	29.93846	29.73675	30.95588	28.93396	28.67428	29.76063	29.2617	26	33	27
pcdh2ac	25.23059	26.10955	NaN	26.66966	26.89064	NaN	26.02147	26.81042	NaN	1	1	0
pcl	30.85103	30.14637	30.56502	30.28173	31.61881	29.27353	28.83632	30.85244	29.71555	36	38	31
pkma	NaN	NaN	21.95059	NaN	NaN	NaN	NaN	NaN	NaN	0	0	1
plgrkt	NaN	20.37878	20.60159	NaN	20.05497	19.13711	NaN	18.94512	17.88435	0	1	1

plscr3b	21.2678	22.97414	23.62723	19.94492	22.37997	20.59076	20.59049	21.97638	19.84769	1	2	2
ppiaa	21.24342	22.85129	NaN	20.23058	22.15085	NaN	20.81956	21.7177	NaN	1	1	0
ppm1aa	26.20124	25.85897	25.67793	24.46264	25.50494	23.58451	23.54156	24.90899	23.10023	10	9	6
ppm1ba	23.44847	21.18447	22.78674	21.2083	20.40447	20.37518	20.10034	19.10121	20.01971	2	1	2
ppm1bb	25.09378	24.82216	25.20758	23.65567	24.68475	23.65851	22.85584	23.95686	22.54614	4	4	4
ppm1g	27.91991	27.86189	27.6064	25.28072	25.83925	24.45482	24.49314	24.13289	23.04303	5	4	6
ppp3r1b;ppp3r1a	24.95096	24.92397	24.507	25.51965	25.97784	24.7684	25.59101	25.32361	23.7685	2	6	1
prkacaa	26.08799	25.00744	25.91878	24.29985	25.3133	23.61706	23.94458	24.69737	23.55731	5	5	6
prkacbb;prkacab	21.42481	21.91514	22.63575	19.27045	21.28184	20.13094	18.93105	20.90102	20.38564	1	1	1
psmb5	NaN	NaN	20.75393	NaN	NaN	17.84236	NaN	NaN	18.75447	0	0	1
psmc1a	28.00201	27.08548	28.49098	26.45782	27.03517	26.23821	24.81627	25.52402	24.75618	14	12	13
psmc1b	24.84062	25.20888	24.59001	23.57623	24.47209	23.39702	23.66557	24.08513	23.50724	2	2	2
pvalb1	NaN	NaN	NaN	22.55574	NaN	NaN	NaN	NaN	NaN	1	0	0
pvalb2	NaN	21.88148	NaN	NaN	22.89447	NaN	22.0696	23.29614	NaN	1	2	0
ran	20.57154	20.92071	22.10894	NaN	20.15906	NaN	NaN	NaN	NaN	1	1	1
rars2	20.77354	20.42634	20.44889	19.5098	18.75483	19.5762	28.79801	27.66939	28.42834	1	1	1
rcv1	21.86891	25.25976	22.00237	24.47988	24.94114	23.4837	26.75366	27.08821	25.73068	6	8	5
rcvrna	23.60479	23.82094	23.22904	22.00089	23.25263	23.76577	24.20672	25.72012	24.21697	1	4	2
retsatl	21.6888	20.92325	23.98198	16.13715	18.16595	18.97304	16.50275	16.23444	NaN	2	1	4
rpl12	NaN	NaN	21.30368	NaN	NaN	NaN	NaN	NaN	NaN	0	0	1
rpl13	25.36693	24.30473	26.40553	25.9205	24.33946	25.31924	26.26385	24.70852	25.24478	3	3	4
rpl13a	27.11486	24.87558	25.46198	23.37518	24.13641	22.29272	23.57727	23.71796	24.36085	5	4	4
rpl15	21.6868	NaN	23.52049	20.34104	NaN	20.41473	20.41844	NaN	21.91053	2	0	1
rpl17	20.91685	21.28399	21.68603	20.31041	21.44834	22.05106	20.29558	20.54934	19.68574	1	1	1
rpl18	28.5862	26.06862	26.84577	25.80978	26.17085	24.43536	25.54428	25.35451	24.73805	2	2	2
rpl18a	23.65064	22.69092	23.69604	21.71741	21.90036	20.85407	21.51215	22.07633	22.62228	2	1	1
rpl19	26.31173	24.39748	24.53641	24.74597	24.43663	23.62623	24.34564	24.71539	24.0592	2	2	1
rpl22	20.59805	21.64077	22.08703	19.35419	21.31063	19.18268	20.43441	20.75369	19.35847	1	1	1
rpl23	NaN	22.3738	21.41648	NaN	22.70398	19.96888	NaN	21.67861	17.93635	0	1	1
rpl24	NaN	NaN	20.97442	NaN	NaN	NaN	NaN	NaN	NaN	0	0	1
rpl28	25.44266	23.12942	24.4501	22.70664	23.34408	22.88453	24.22788	23.49497	23.47445	1	1	1
rpl3	22.34673	22.31551	23.45048	21.31512	21.70852	20.53953	20.62996	21.35283	21.99862	2	2	2
rpl32	25.44894	24.37696	25.99867	24.4139	24.45362	24.00247	23.73603	23.66112	24.7006	2	3	2
rpl34	22.819	21.08499	22.83258	21.40883	21.14992	20.12591	22.3347	20.84353	21.2939	1	1	1
rpl35	25.71361	23.26284	24.13391	23.04135	23.96455	22.77838	23.25262	23.09613	23.30814	2	1	1
rpl35a	NaN	20.50978	21.20353	NaN	19.84374	17.68791	NaN	20.04674	17.59631	0	1	1
rpl36	25.64075	23.74253	25.43838	24.14313	24.28426	23.3041	24.26342	23.51292	23.74571	4	3	2
rpl39	24.46289	23.73221	25.68782	23.57473	24.28588	23.62868	24.58652	23.99118	24.37643	1	1	1
rpl4	20.37952	NaN	24.31617	20.24423	NaN	20.90624	20.8671	NaN	22.15684	1	0	3
rpl5b	22.13571	22.60535	23.74273	20.47961	22.59876	20.28546	20.84483	21.93117	20.85742	1	3	4
rpl6	26.62422	25.96283	27.42053	26.40785	25.22953	25.32248	26.26324	25.58222	26.20631	4	4	4
rpl7	28.56576	26.48746	27.15509	26.29295	26.80354	25.04398	26.61484	25.34736	26.59733	8	4	6
rpl7a	20.9869	22.88021	23.57196	19.27556	21.20461	19.67173	19.94006	20.91539	20.94735	1	2	2
rpl8	21.13477	21.61491	23.48419	20.56913	21.24586	20.66014	21.05828	20.77322	21.45056	1	1	1
rpl9	NaN	NaN	20.07535	NaN	NaN	18.13761	NaN	NaN	18.60552	0	0	1
rplp0	NaN	NaN	20.95518	NaN	NaN	NaN	NaN	NaN	NaN	0	0	1
rps12	NaN	20.43033	20.73405	NaN	18.432	NaN	NaN	19.20192	NaN	0	1	1
rps14	NaN	21.50436	21.92998	NaN	21.25914	20.18991	NaN	20.30441	20.69523	0	1	1
rps16	NaN	NaN	21.55225	NaN	NaN	19.96395	NaN	NaN	19.33344	0	0	1
rps17	NaN	NaN	21.77052	NaN	NaN	NaN	NaN	NaN	NaN	0	0	1

rps2	24.16821	24.84168	24.29327	22.34673	23.82542	22.25868	21.92154	23.37637	23.1158	5	6	5
rps26;rps26l	NaN	20.48797	NaN	NaN	19.33239	NaN	NaN	17.67059	NaN	0	1	0
rps27.1	20.99231	21.02992	21.60711	18.57378	21.00148	19.68936	21.16515	20.15561	21.0452	1	1	1
rps27a;ubb;uba52;si:ch21	23.09721	21.9117	23.7679	22.22565	21.90819	21.55459	22.0496	21.95511	22.07783	2	1	2
rps3	20.37061	22.26095	23.05909	18.64987	21.97358	19.25511	19.42255	21.16723	19.74014	1	2	2
rps3a	20.88524	23.20184	24.32547	19.48569	23.26742	21.60182	19.64114	22.35912	22.19931	1	3	3
rps4x	NaN	22.74936	22.5015	NaN	22.22179	19.92503	NaN	22.11794	20.89186	0	1	1
rps5	NaN	21.33772	NaN	NaN	21.32778	NaN	NaN	20.86507	NaN	0	1	0
rps6	NaN	22.17792	23.62008	NaN	21.47511	22.18782	NaN	22.17063	22.58834	0	1	1
rps8	21.43405	NaN	24.36946	20.25419	NaN	19.58151	20.38701	NaN	20.74468	2	0	4
rps9	25.75075	22.67312	26.05951	23.62478	22.54106	24.01316	24.05117	22.84477	24.87225	4	2	5
rpsa	NaN	21.36565	21.35019	NaN	20.84491	18.48006	NaN	20.06723	18.94678	0	1	1
rrm1	NaN	NaN	20.60277	NaN	NaN	NaN	NaN	NaN	NaN	0	0	1
s100a1	NaN	NaN	22.44339	NaN	NaN	NaN	NaN	NaN	NaN	0	0	1
si:ch211-103n10.5	NaN	NaN	NaN	NaN	NaN	NaN	22.03366	NaN	NaN	1	0	0
si:ch211-113a14.18;si:ch2	22.6289	21.56941	22.39679	22.60107	21.63458	22.08923	23.60818	21.2898	21.99662	2	1	1
si:ch211-288g17.3	23.03937	20.96219	23.23643	24.66031	22.52032	23.79412	24.52815	22.28808	22.99521	4	1	2
si:dkey-238c7.16	21.20634	NaN	NaN	19.77187	NaN	NaN	18.88599	NaN	NaN	1	0	0
si:dkey-28b4.8	NaN	22.11956	NaN	NaN	24.97364	NaN	NaN	23.20098	NaN	0	1	0
slc25a5	22.90189	24.36633	24.53955	21.79729	25.29498	22.72823	22.31271	24.91693	23.59184	2	6	3
slc44a1a	23.63843	23.50567	23.72307	21.48331	21.36346	19.61558	21.23975	20.28974	19.09954	4	4	2
slco4a1	NaN	23.27028	NaN	NaN	21.00175	NaN	NaN	28.48788	NaN	0	1	0
smyhc1	NaN	18.93825	NaN	NaN	20.85369	NaN	NaN	19.35988	NaN	0	1	0
snrnp70	22.63995	22.33118	23.61212	24.1757	22.96233	23.84422	24.01026	23.06763	22.91185	3	3	3
srsf11	25.09985	24.65671	25.08655	25.62428	25.16592	23.88539	23.9865	23.94065	22.54736	3	4	3
ssx2ip	21.81425	22.67736	NaN	23.06083	20.72684	NaN	21.35558	22.95757	NaN	1	1	0
syt1a;syt1b	16.72347	17.59215	NaN	17.41576	18.5597	NaN	23.49814	21.6999	NaN	3	2	0
tkt	NaN	20.70514	22.03406	NaN	21.10361	19.7073	NaN	21.06894	19.16627	0	1	1
tnika;tnikb;mink1	NaN	NaN	NaN	22.26058	NaN	NaN	NaN	NaN	NaN	1	0	0
trim23	NaN	21.8405	NaN	NaN	20.55375	NaN	NaN	20.53032	NaN	0	1	0
tuba1b	23.14261	25.09708	24.40178	22.8853	26.06275	22.4998	22.88122	25.47445	23.60014	2	7	3
tuba4l	22.99279	22.69198	24.42387	21.69064	23.51713	21.72949	21.67094	22.8182	22.52378	3	2	3
tuba8l4	NaN	NaN	22.61746	NaN	NaN	18.18679	NaN	NaN	19.09195	0	0	1
tubb4b	NaN	20.2781	NaN	NaN	20.96445	NaN	NaN	20.07627	NaN	0	1	0
tubb4b	22.50191	25.30515	24.6732	22.26121	25.65417	22.36645	21.55997	24.59679	22.98557	2	8	4
uchl1	20.98593	NaN	21.89604	18.92056	NaN	17.48526	18.11294	NaN	NaN	1	0	1
uqcrq	NaN	NaN	21.36062	NaN	NaN	20.20455	NaN	NaN	20.20407	0	0	1
vcp	NaN	19.64176	NaN	NaN	20.97463	NaN	NaN	19.89491	NaN	0	1	0
vdac2	NaN	21.34706	21.45091	NaN	21.86427	NaN	NaN	19.44253	NaN	0	1	1
vsnl1a	23.80666	23.46064	23.12997	24.4472	24.12731	23.10548	24.61263	24.46563	22.64051	4	4	2
vsnl1b	24.03381	24.38949	24.83894	27.09889	27.20564	27.01808	27.66472	27.85691	27.11128	10	11	7
vtg1	30.11843	27.79123	29.43269	26.73337	26.36815	25.06196	23.76334	25.57488	23.91223	9	7	9
vtg2	30.92174	29.56333	30.34244	27.23526	28.21206	25.80196	26.10966	27.62443	25.05685	39	36	38
vtg3	26.26958	23.78596	25.48837	22.52991	22.466	20.53744	21.47189	22.05653	22.7376	9	3	7
vtg4	26.10853	25.32742	26.38482	19.5547	23.50712	20.71389	17.56733	23.63733	21.51567	2	2	2
vtg4	32.5586	30.7336	32.11623	28.71999	29.29761	27.25431	26.2222	28.60332	26.26673	45	30	45
vtg5	28.96072	27.01945	29.11553	25.36436	25.41687	26.12688	24.68961	25.32107	25.27558	13	9	11
vtg6	29.8246	28.2964	29.4577	26.23134	26.84158	24.77671	23.65905	26.24154	23.83152	12	8	12
vtg7	30.8178	28.87836	30.48328	27.01084	26.91934	25.49448	24.8306	26.62575	25.22589	22	15	20
wu:fe37d09;LOC1005381	30.19084	27.9332	29.79562	29.4973	29.74626	29.0583	29.55017	29.6526	29.50631	6	7	6

yes1	24.90421	28.12509	24.43771	22.85844	22.69523	21.69825	22.18671	21.19592	20.50271	5	4	5
ywhaba	NaN	NaN	21.52655	NaN	NaN	19.53611	NaN	NaN	19.71386	0	0	1
ywhabb	17.6202	21.24156	NaN	17.97175	22.11544	NaN	19.05457	25.68105	NaN	1	1	0
ywhabl	NaN	NaN	21.43079	NaN	NaN	18.89134	NaN	NaN	19.72868	0	0	1
ywhae1	NaN	NaN	20.71431	NaN	NaN	18.38005	NaN	NaN	19.7963	0	0	1
ywhaqb	19.98635	23.00992	24.17577	18.51368	23.66731	19.96437	16.64723	23.36933	19.10224	1	2	2
ywhaz	NaN	NaN	20.86703	NaN	NaN	NaN	NaN	NaN	NaN	0	0	1
zgc:112994	23.40237	21.75079	21.88241	NaN	NaN	NaN	NaN	NaN	NaN	1	1	1
zgc:114037;zgc:171759;z	25.84597	24.64927	25.83904	25.31552	25.72507	24.57664	26.18221	24.92007	25.0566	3	1	1
zgc:136380	22.93736	22.71616	21.26585	22.733	22.80548	21.32773	21.3118	21.64447	20.8662	2	3	1
zgc:153440	NaN	20.88874	21.02174	NaN	19.76513	NaN	NaN	19.68563	NaN	0	1	1
zgc:153675	NaN	NaN	22.3126	NaN	NaN	22.34112	NaN	NaN	19.25981	0	0	1
zgc:162618	21.23567	NaN	NaN	21.23397	NaN	NaN	18.60274	NaN	NaN	1	0	0
zgc:63695	20.40072	21.38759	NaN	20.58353	21.85647	NaN	21.42116	22.36912	NaN	1	2	0
zgc:73075	20.85939	19.10229	20.63414	19.72376	21.71209	18.73615	23.31879	26.15219	25.21119	3	7	4
zgc:92533	22.14168	22.49148	22.85886	21.44491	24.2981	21.64205	22.07064	23.63134	21.97323	2	6	2
znf395	21.19886	NaN	NaN	18.40495	NaN	NaN	NaN	NaN	NaN	1	0	0

Gene names	GOMF name	GOBP slim name	GOCC slim name	KEGG name
anxa13	binding;calcium ion binding;calcium-dependent phospholipid binding;cation binding;ion binding;lipid binding;metal ion binding;phospholipid binding			
arf1	binding;GTP binding;guanyl nucleotide binding;guanyl ribonucleotide binding;nucleotide binding;purine nucleotide binding;purine ribonucleoside triphosphate binding;purine ribonucleotide binding;ribonucleotide binding	anatomical structure development;biological regulation;cellular process;developmental process;response to stimulus;signal transduction	cell part;intracellular	
arf1l;arf3b;arf2	binding;GTP binding;guanyl nucleotide binding;guanyl ribonucleotide binding;nucleotide binding;purine nucleotide binding;purine ribonucleoside triphosphate binding;purine ribonucleotide binding;ribonucleotide binding	biological regulation;cellular process;response to stimulus;signal transduction	cell part;intracellular	Vibrio cholerae infection
arf4a	binding;GTP binding;guanyl nucleotide binding;guanyl ribonucleotide binding;nucleotide binding;purine nucleotide binding;purine ribonucleoside triphosphate binding;purine ribonucleotide binding;ribonucleotide binding	biological regulation;cellular process;response to stimulus;signal transduction	cell part;intracellular	
arf5;DKEY-238C7.11	binding;GTP binding;guanyl nucleotide binding;guanyl ribonucleotide binding;nucleotide binding;purine nucleotide binding;purine ribonucleoside triphosphate binding;purine ribonucleotide binding;ribonucleotide binding	biological regulation;cellular process;response to stimulus;signal transduction	cell part;intracellular	
arf6b	binding;GTP binding;guanyl nucleotide binding;guanyl ribonucleotide binding;nucleotide binding;purine nucleotide binding;purine ribonucleoside triphosphate binding;purine ribonucleotide binding;ribonucleotide binding	biological regulation;cellular process;response to stimulus;signal transduction	cell part;intracellular	Endocytosis;Fc gamma R-mediated phagocytosis
arl1	binding;catalytic activity;enzyme activator activity;enzyme regulator activity;GTP binding;GTPase activity;guanyl nucleotide binding;guanyl ribonucleotide binding;hydrolase activity;hydrolase activity, acting on acid anhydrides;hydrolase activity, acting on acid anhydrides, in phosphorus-containing anhydrides;nucleoside-triphosphatase activity;nucleotide binding;purine nucleotide binding;purine ribonucleoside triphosphate binding;purine ribonucleotide binding;pyrophosphatase activity;ribonucleotide binding	biological regulation;cellular component organization;cellular localization;cellular metabolic process;cellular process;endosome transport;establishment of localization;Golgi organization;localization;metabolic process;organelle organization;response to stimulus;secondary metabolic process;signal transduction;toxin metabolic process;transport;vesicle-mediated transport	cell part;Golgi apparatus;intracellular membrane-bounded organelle;intracellular organelle;organelle	

basp1			cell part;membrane;plasma membrane	
cast	calcium-dependent cysteine-type endopeptidase inhibitor activity;cysteine-type endopeptidase inhibitor activity;endopeptidase inhibitor activity;endopeptidase regulator activity;enzyme inhibitor activity;enzyme regulator activity;peptidase inhibitor activity;peptidase regulator activity			
cd81			cell part	B cell receptor signaling pathway;Hepatitis C;Malaria
chmp6b;chmp6		establishment of localization;protein transport;transport	cell part;endomembrane system;membrane	Endocytosis
fam129bb;fam129ba	binding;lipid binding;phospholipid binding			
fam49ba				
flot2a		biological adhesion;cell adhesion;cellular process	cell part;cytoplasmic membrane-bounded vesicle;endosome;intracellular membrane-bounded organelle;intracellular organelle;membrane;organelle;vesicle	Insulin signaling pathway
flot2b				Insulin signaling pathway
fyna	adenyl nucleotide binding;adenyl ribonucleotide binding;ATP binding;binding;catalytic activity;cation binding;ion binding;kinase activity;metal ion binding;non-membrane spanning protein tyrosine kinase activity;nucleotide binding;phosphotransferase activity, alcohol group as acceptor;protein kinase activity;protein tyrosine kinase activity;purine nucleotide binding;purine ribonucleoside triphosphate binding;purine ribonucleotide binding;ribonucleotide binding;transferase activity;transferase activity, transferring phosphorus-containing groups	anatomical structure morphogenesis;biological regulation;cellular homeostasis;cellular ion homeostasis;cellular metabolic process;cellular process;developmental process;homeostatic process;macromolecule metabolic process;macromolecule modification;metabolic process;phosphorus metabolic process;primary metabolic process;protein metabolic process;protein modification process;protein phosphorylation;regulation of protein modification process	cell part;cytosol;intracellular membrane-bounded organelle;intracellular organelle;nucleus;organelle	Adherens junction;Axon guidance;Fc epsilon RI signaling pathway;Focal adhesion;Measles;Natural killer cell mediated cytotoxicity;Osteoclast differentiation;Pathogenic Escherichia coli infection;Prion diseases;T cell receptor signaling pathway;Viral myocarditis

gnai1;gnai3;gnai2b	binding;catalytic activity;G-protein beta/gamma-subunit complex binding;G-protein-coupled receptor binding;GTP binding;GTPase activity;guanyl nucleotide binding;guanyl ribonucleotide binding;hydrolase activity;hydrolase activity, acting on acid anhydrides;hydrolase activity, acting on acid anhydrides, in phosphorus-containing anhydrides;metabotropic serotonin receptor binding;molecular transducer activity;nucleoside-triphosphatase activity;nucleotide binding;protein binding;protein complex binding;purine nucleotide binding;purine ribonucleoside triphosphate binding;purine ribonucleotide binding;pyrophosphatase activity;receptor binding;ribonucleotide binding;signal transducer activity	biological regulation;cellular process;response to stimulus;signal transduction	cell part;macromolecular complex;protein complex	Axon guidance;Chagas disease (American trypanosomiasis);Chemokine signaling pathway;Gap junction;Gastric acid secretion;Leukocyte transendothelial migration;Long-term depression;Melanogenesis;Progesterone-mediated oocyte maturation;Tight junction;Toxoplasmosis
gnai2a	binding;catalytic activity;G-protein beta/gamma-subunit complex binding;G-protein-coupled receptor binding;GTP binding;GTPase activity;guanyl nucleotide binding;guanyl ribonucleotide binding;hydrolase activity;hydrolase activity, acting on acid anhydrides;hydrolase activity, acting on acid anhydrides, in phosphorus-containing anhydrides;molecular transducer activity;nucleoside-triphosphatase activity;nucleotide binding;protein binding;protein complex binding;purine nucleotide binding;purine ribonucleoside triphosphate binding;purine ribonucleotide binding;pyrophosphatase activity;receptor binding;ribonucleotide binding;signal transducer activity	biological regulation;cellular process;response to stimulus;signal transduction	cell part;macromolecular complex;protein complex	Axon guidance;Chagas disease (American trypanosomiasis);Chemokine signaling pathway;Gap junction;Gastric acid secretion;Leukocyte transendothelial migration;Long-term depression;Melanogenesis;Progesterone-mediated oocyte maturation;Tight junction;Toxoplasmosis

gnao1a	binding;catalytic activity;G-protein beta/gamma-subunit complex binding;G-protein-coupled receptor binding;GTP binding;GTPase activity;guanyl nucleotide binding;guanyl ribonucleotide binding;hydrolase activity;hydrolase activity, acting on acid anhydrides;hydrolase activity, acting on acid anhydrides, in phosphorus-containing anhydrides;metabotropic serotonin receptor binding;molecular transducer activity;nucleoside-triphosphatase activity;nucleotide binding;protein binding;protein complex binding;purine nucleotide binding;purine ribonucleoside triphosphate binding;purine ribonucleotide binding;pyrophosphatase activity;receptor binding;ribonucleotide binding;signal transducer activity	biological regulation;cellular process;response to stimulus;signal transduction	cell part;macromolecular complex;protein complex	Chagas disease (American trypanosomiasis);Long-term depression;Melanogenesis;Toxoplasmosis
gnat2	binding;catalytic activity;G-protein beta/gamma-subunit complex binding;G-protein-coupled receptor binding;GTP binding;GTPase activity;guanyl nucleotide binding;guanyl ribonucleotide binding;hydrolase activity;hydrolase activity, acting on acid anhydrides;hydrolase activity, acting on acid anhydrides, in phosphorus-containing anhydrides;molecular transducer activity;nucleoside-triphosphatase activity;nucleotide binding;protein binding;protein complex binding;purine nucleotide binding;purine ribonucleoside triphosphate binding;purine ribonucleotide binding;pyrophosphatase activity;receptor binding;ribonucleotide binding;signal transducer activity	biological regulation;catabolic process;cellular metabolic process;cellular nitrogen compound metabolic process;cellular process;detection of stimulus;heterocycle metabolic process;metabolic process;nitrogen compound metabolic process;nucleobase-containing compound catabolic process;nucleobase-containing compound metabolic process;nucleobase-containing small molecule metabolic process;nucleotide metabolic process;primary metabolic process;response to abiotic stimulus;response to chemical stimulus;response to external stimulus;response to stimulus;signal transduction;small molecule metabolic process	cell part;cell projection;cilium;macromolecular complex;photoreceptor inner segment;protein complex	Phototransduction
gorasp2				
hpca	binding;calcium ion binding;cation binding;ion binding;metal ion binding			
lnpa				
LOC557507	binding;catalytic activity;coenzyme binding;cofactor binding;flavin adenine dinucleotide binding;oxidoreductase activity			
lrrc57				
marcksl1a				Fc gamma R-mediated phagocytosis
marcksl1b				Fc gamma R-mediated phagocytosis;Leishmaniasis
ncalda	binding;calcium ion binding;cation binding;ion binding;metal ion binding			

ndufb7	catalytic activity;NADH dehydrogenase (quinone) activity;NADH dehydrogenase (ubiquinone) activity;NADH dehydrogenase activity;oxidoreductase activity;oxidoreductase activity, acting on NADH or NADPH;oxidoreductase activity, acting on NADH or NADPH, quinone or similar compound as acceptor		cell part;intracellular membrane-bounded organelle;intracellular organelle;mitochondrion;organelle	Alzheimer's disease;Huntington's disease;Oxidative phosphorylation;Parkinson's disease
ppm1aa	binding;catalytic activity;cation binding;hydrolase activity;hydrolase activity, acting on ester bonds;ion binding;magnesium ion binding;manganese ion binding;metal ion binding;phosphatase activity;phosphoprotein phosphatase activity;phosphoric ester hydrolase activity;protein serine/threonine phosphatase activity;transition metal ion binding	cellular metabolic process;cellular process;macromolecule metabolic process;macromolecule modification;metabolic process;phosphorus metabolic process;primary metabolic process;protein dephosphorylation;protein metabolic process;protein modification process		MAPK signaling pathway
ppm1ba	binding;catalytic activity;cation binding;hydrolase activity;hydrolase activity, acting on ester bonds;ion binding;magnesium ion binding;manganese ion binding;metal ion binding;phosphatase activity;phosphoprotein phosphatase activity;phosphoric ester hydrolase activity;protein serine/threonine phosphatase activity;transition metal ion binding	cellular metabolic process;cellular process;macromolecule metabolic process;macromolecule modification;metabolic process;phosphorus metabolic process;primary metabolic process;protein dephosphorylation;protein metabolic process;protein modification process		MAPK signaling pathway
ppm1bb	binding;catalytic activity;cation binding;hydrolase activity;hydrolase activity, acting on ester bonds;ion binding;magnesium ion binding;manganese ion binding;metal ion binding;phosphatase activity;phosphoprotein phosphatase activity;phosphoric ester hydrolase activity;protein serine/threonine phosphatase activity;transition metal ion binding	biological regulation;cellular metabolic process;cellular process;macromolecule metabolic process;macromolecule modification;metabolic process;phosphorus metabolic process;primary metabolic process;protein dephosphorylation;protein metabolic process;protein modification process		MAPK signaling pathway
ppm1g	binding;catalytic activity;cation binding;hydrolase activity;hydrolase activity, acting on ester bonds;ion binding;metal ion binding;phosphatase activity;phosphoprotein phosphatase activity;phosphoric ester hydrolase activity;protein serine/threonine phosphatase activity	cellular metabolic process;cellular process;macromolecule metabolic process;macromolecule modification;metabolic process;phosphorus metabolic process;primary metabolic process;protein dephosphorylation;protein metabolic process;protein modification process		

ppp3r1b;ppp3r1a	binding;calcium ion binding;cation binding;ion binding;metal ion binding			Alzheimer's disease;Amyotrophic lateral sclerosis (ALS);Apoptosis;Axon guidance;B cell receptor signaling pathway;Calcium signaling pathway;ko05152;Long-term potentiation;MAPK signaling pathway;Natural killer cell mediated cytotoxicity;Oocyte meiosis;T cell receptor signaling pathway;VEGF signaling pathway;Wnt signaling pathway
prkacaa	adenyl nucleotide binding;adenyl ribonucleotide binding;ATP binding;binding;catalytic activity;kinase activity;nucleotide binding;phosphotransferase activity, alcohol group as acceptor;protein kinase activity;protein serine/threonine kinase activity;purine nucleotide binding;purine ribonucleoside triphosphate binding;purine ribonucleotide binding;ribonucleotide binding;transferase activity;transferase activity, transferring phosphorus-containing groups			Amoebiasis;Apoptosis;Bile secretion;Calcium signaling pathway;Chemokine signaling pathway;Dilated cardiomyopathy;Endocrine and other factor-regulated calcium reabsorption;Gap junction;Gastric acid secretion;GnRH signaling pathway;Hedgehog signaling pathway;Insulin signaling pathway;Long-term potentiation;MAPK signaling pathway;Meiosis - yeast;Melanogenesis;Olfactory transduction;Oocyte meiosis;Prion diseases;Progesterone-mediated oocyte maturation;Salivary secretion;Taste transduction;Vascular smooth muscle contraction;Vasopressin-regulated water reabsorption;Vibrio cholerae infection;Wnt signaling pathway

prkacbb;prkacab	adenyl nucleotide binding;adenyl ribonucleotide binding;ATP binding;binding;catalytic activity;kinase activity;nucleotide binding;phosphotransferase activity, alcohol group as acceptor;protein kinase activity;protein serine/threonine kinase activity;purine nucleotide binding;purine ribonucleoside triphosphate binding;purine ribonucleotide binding;ribonucleotide binding;transferase activity;transferase activity, transferring phosphorus-containing groups			Amoebiasis;Apoptosis;Bile secretion;Calcium signaling pathway;Chemokine signaling pathway;Dilated cardiomyopathy;Endocrine and other factor-regulated calcium reabsorption;Gap junction;Gastric acid secretion;GnRH signaling pathway;Hedgehog signaling pathway;Insulin signaling pathway;Long-term potentiation;MAPK signaling pathway;Meiosis - yeast;Melanogenesis;Olfactory transduction;Oocyte meiosis;Prion diseases;Progesterone-mediated oocyte maturation;Salivary secretion;Taste transduction;Vascular smooth muscle contraction;Vasopressin-regulated water reabsorption;Vibrio cholerae infection;Wnt signaling pathway
psmc1a	adenyl nucleotide binding;adenyl ribonucleotide binding;ATP binding;binding;catalytic activity;hydrolase activity;hydrolase activity, acting on acid anhydrides;hydrolase activity, acting on acid anhydrides, in phosphorus-containing anhydrides;nucleoside-triphosphatase activity;nucleotide binding;purine nucleotide binding;purine ribonucleoside triphosphate binding;purine ribonucleotide binding;pyrophosphatase activity;ribonucleotide binding	catabolic process;macromolecule metabolic process;metabolic process;primary metabolic process;protein catabolic process;protein metabolic process	cell part;cytoplasm;macromolecular complex;proteasome complex;protein complex	
psmc1b	adenyl nucleotide binding;adenyl ribonucleotide binding;ATP binding;binding;catalytic activity;hydrolase activity;hydrolase activity, acting on acid anhydrides;hydrolase activity, acting on acid anhydrides, in phosphorus-containing anhydrides;nucleoside-triphosphatase activity;nucleotide binding;purine nucleotide binding;purine ribonucleoside triphosphate binding;purine ribonucleotide binding;pyrophosphatase activity;ribonucleotide binding	catabolic process;macromolecule metabolic process;metabolic process;primary metabolic process;protein catabolic process;protein metabolic process	cell part;cytoplasm;macromolecular complex;proteasome complex;protein complex	Proteasome
rcv1	binding;calcium ion binding;cation binding;ion binding;metal ion binding			
rcvrna	binding;calcium ion binding;cation binding;ion binding;metal ion binding			Phototransduction
slc44a1a			cell part	

vsnl1b	binding;calcium ion binding;cation binding;ion binding;metal ion binding			
yes1	adenyl nucleotide binding;adenyl ribonucleotide binding;ATP binding;binding;catalytic activity;kinase activity;non-membrane spanning protein tyrosine kinase activity;nucleotide binding;phosphotransferase activity, alcohol group as acceptor;protein kinase activity;protein tyrosine kinase activity;purine nucleotide binding;purine ribonucleoside triphosphate binding;purine ribonucleotide binding;ribonucleotide binding;transferase activity;transferase activity, transferring phosphorus-containing groups	anatomical structure morphogenesis;biological adhesion;biological regulation;cell adhesion;cell cycle;cellular metabolic process;cellular process;developmental process;macromolecule metabolic process;macromolecule modification;metabolic process;phosphorus metabolic process;primary metabolic process;protein metabolic process;protein modification process;protein phosphorylation;regulation of protein modification process	cell part;cytoskeleton;cytosol;intracellular membrane-bounded organelle;intracellular non-membrane-bounded organelle;intracellular organelle;membrane;microtubule organizing center;nucleus;organelle;plasma membrane	Adherens junction;Bacterial invasion of epithelial cells;Endocytosis;Epithelial cell signaling in Helicobacter pylori infection;ErbB signaling pathway;Focal adhesion;Gap junction;GnRH signaling pathway;ko05152;Tight junction;VEGF signaling pathway
zgc:63695	binding;calcium ion binding;cation binding;ion binding;metal ion binding			
zgc:73075	binding;calcium ion binding;cation binding;ion binding;metal ion binding			

4. References

[1] S. T. Laughlin, J. M. Baskin, S. L. Amacher, C. R. Bertozzi, *Science* **2008**, *320*, 664-667.

[2] V. Link, A. Shevchenko, C.-P. Heisenberg, *BMC Dev. Biol.* **2006**, *6*, 1-9.

[3] F. I. Hinz, D. C. Dieterich, D. A. Tirrell, E. M. Schuman, *ACS Chem. Neurosci.* **2011**, *3*, 40-49.

[4] J. Rappsilber, Y. Ishihama, M. Mann, *Anal. Chem.* **2003**, *75*, 663-670.

[5] P. J. Boersema, R. Raijmakers, S. Lemeer, S. Mohammed, A. J. Heck, *Nat. Protoc.* **2009**, *4*, 484-94.

[6] J. Cox, M. Mann, *Nat. Biotechnol.* **2008**, *26*, 1367-1372.

[7] J. Cox, N. Neuhauser, A. Michalski, R. A. Scheltema, J. V. Olsen, M. Mann, *J. Proteome Res.* **2011**,*10*, 1794-1805.

[8] J. Zhang, L. Xin, B. Shan, W. Chen, M. Xie, D. Yuen, W. Zhang, Z. Zhang, G. A. Lajoie, B. Ma, *Mol. Cell. Proteomics* **2012**, *11*, M111 010587.

[9] R. Srinivasan, L. P. Tan, H. Wu, P.-Y. Yang, K. A. Kalesh, S. Q. Yao, *Org. Biomol.Chem.* **2009**, *7*, 1821-1828.

[10] a) M. H. Wright, B. Clough, M. D. Rackham, K. Rangachari, J. A. Brannigan, M. Grainger, D. K. Moss, A. R. Bottrill, W. P. Heal, M. Broncel, R. A. Serwa, D. Brady, D. J. Mann, R. J. Leatherbarrow, R. Tewari, A. J. Wilkinson, A. A. Holder, E. W. Tate., *Nat. Chem.* **2014**, *6*, 112-121. b) E. Thinon, R. A. Serwa, M. Broncel, J. A. Brannigan, U. Brassat, M. H. Wright, W. P. Heal, A. J. Wilkinson, D. J. Mann, E. W. Tate, *Nat. Commun.* **2014**, *5*, 4919.

Full list of authors for ref. 14 from the main text:

[14] J. A. Vizcaíno, E. W. Deutsch, R. Wang, A. Csordas, F. Reisinger, D. Ríos, J. A. Dianes, Z. Sun, T. Farrah, N. Bandeira, P. A. Binz, I. Xenarios, M. Eisenacher, G. Mayer, L. Gatto, A. Campos, R. J. Chalkley, H. J. Kraus, J. P. Albar, S. Martinez-Bartolomé, R. Apweiler, G. S. Omenn, L. Martens, A. R. Jones, H. Hermjakob, *Nat. Biotechnol.* **2014**, *30*, 223-226.
